# Flexible conservatism in the skull modularity of convergently evolved myrmecophagous placental mammals

**DOI:** 10.1186/s12862-022-02030-9

**Published:** 2022-06-30

**Authors:** Sérgio Ferreira-Cardoso, Julien Claude, Anjali Goswami, Frédéric Delsuc, Lionel Hautier

**Affiliations:** 1 Institut des Sciences de l’Evolution de Montpellier (ISEM), CNRS, IRD, EPHE, Université de Montpellier, Montpellier, France; 2 Mammal Section, Life Sciences, Vertebrate Division, The Natural History Museum, London, UK

**Keywords:** Modularity, Skull evolution, Myrmecophagy, Mammals, Geometric morphometrics, Tooth loss

## Abstract

**Background:**

The skull of placental mammals constitutes one of the best studied systems for phenotypic modularity. Several studies have found strong evidence for the conserved presence of two- and six-module architectures, while the strength of trait correlations (integration) has been associated with major developmental processes such as somatic growth, muscle-bone interactions, and tooth eruption. Among placentals, ant- and termite-eating (myrmecophagy) represents an exemplar case of dietary convergence, accompanied by the selection of several cranial morphofunctional traits such as rostrum elongation, tooth loss, and mastication loss. Despite such drastic functional modifications, the covariance patterns of the skull of convergently evolved myrmecophagous placentals are yet to be studied in order to assess the potential consequences of this dietary shift on cranial modularity.

**Results:**

Here, we performed a landmark-based morphometric analysis of cranial covariance patterns in 13 species of myrmecophagous placentals. Our analyses reveal that most myrmecophagous species present skulls divided into six to seven modules (depending on the confirmatory method used), with architectures similar to those of non-myrmecophagous placentals (therian six modules). Within-module integration is also similar to what was previously described for other placentals, suggesting that most covariance-generating processes are conserved across the clade. Nevertheless, we show that extreme rostrum elongation and tooth loss in myrmecophagid anteaters have resulted in a shift in intermodule correlations in the proximal region of the rostrum. Namely, the naso-frontal and maxillo-palatine regions are strongly correlated with the oro-nasal module, suggesting an integrated rostrum conserved from pre-natal developmental processes. In contrast, the similarly toothless pangolins show a weaker correlation between the anterior rostral modules, resembling the pattern of toothed placentals.

**Conclusions:**

These results reveal that despite some integration shifts related to extreme functional and morphological features of myrmecophagous skulls, cranial modular architectures have conserved the typical mammalian scheme.

**Supplementary Information:**

The online version contains supplementary material available at 10.1186/s12862-022-02030-9.

## Introduction

Modularity is one of the most ubiquitous intrinsic characteristics of biological systems [1, 2]. Modules represent semi-independent units that generate coordinated phenotypic variation, potentially driven by functional or developmental processes [3, 4]. As a result, covariance/correlation between phenotypic traits has been considered as reflecting the processes driving morphological integration and has been used as a proxy for estimating or testing modularity hypotheses [5, 6]. Moreover, the patterns of trait/module associations are often considered to be a primary constraint on morphological variation and, therefore, evolution [7]. Specifically, structural partitions in well-integrated modules theoretically promote the evolutionary potential of some functional units, while limiting the effects of drift or the action of directional selection on uncorrelated characters [8–11]. This axiom was the basis of several studies that aimed at defining modules and testing modularity hypotheses on a broad range of organisms and body parts such as insect wings [12–14], mammalian mandibles [15, 16], limbs [17], and vertebrae [18].

Trait correlations within the cranium have been intensively studied in the vertebrate skull and explained in terms of functional and ontogenetic constraints. Investigations of the skull modularity and integration patterns have been conducted in fish [19–21], birds [22, 23], amphibians [24–26], reptiles [27–29], and mammals [30–32]. The mammalian skull presents a modular pattern with a major separation between the face and the neurocranium [6, 33, 34], as well as between the rostrum, the vault, and the basicranium (e.g., [6, 31, 35, 36]). Further regionalization of its modular architecture has been proposed with empirical evidence for six modules corresponding to the oro-nasal, molar-palate, orbit, zygomatic-pterygoid, vault, and basicranium regions [6, 31, 35]. These phenotypic modules have been suggested to correspond to both developmental and functional constraints, some being specifically related to food detection and capture (oro-nasal), food processing (molar-palate), and origin of adductor muscles. Namely, the pterygoid and the masseter take their origins on the pterygoid fossa and zygomatic arch, respectively, while the superficial temporal arises from the sagittal and occipital crests of the squamosal and occipital bones (cranial vault).

Dietary shifts have a major impact on skull shape evolution and were often considered to explain the phenotypic diversity across vertebrates (e.g., [37–44]). The morphological diversification of the feeding apparatus has played a major role in mammal radiations, varying from strong jaws with bone-crushing teeth to edentulous and elongated jaws [45]. The loss or reduction of teeth has occurred in all major mammalian clades [46, 47] and is often associated with a specialized myrmecophagous diet [48, 49]. Myrmecophagy evolved independently in divergent placental lineages such as anteaters, giant armadillos, pangolins, aardwolves, and aardvarks. The evolution of these taxa toward this specialized diet led to an extensive rearrangement of their skull morphology [47, 50–54]. Given the conserved development of the vertebrate skull [55, 56], these morphological changes likely occurred in a context of highly conserved modularity.

**Fig. 1:**
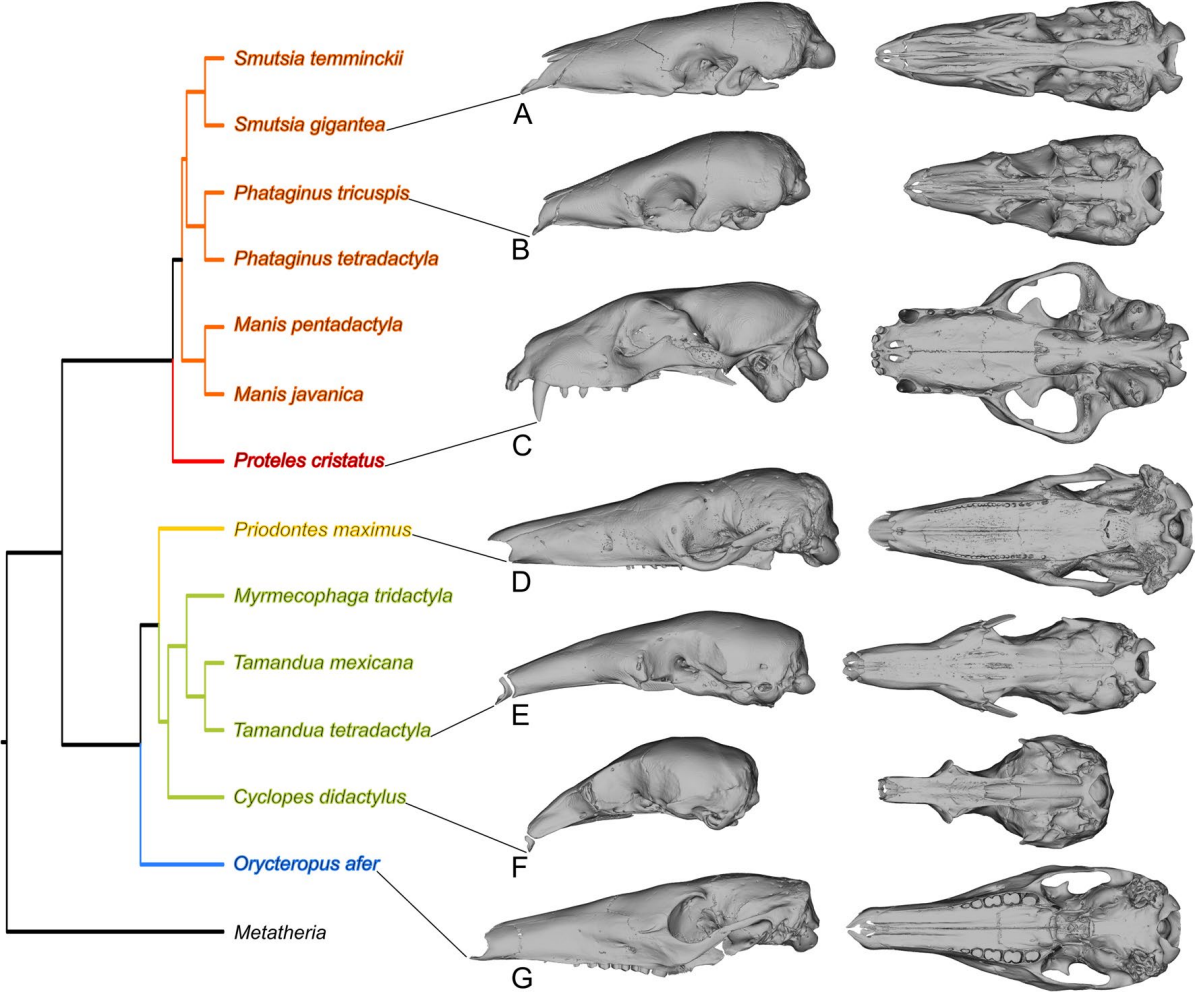
Phylogenetic relationships of 13 myrmecophagous placentals. Lateral (left) and ventral (right) views of the giant pangolin (**A**), the white bellied pangolin (**B**), the aardwolf (**C**), the giant armadillo (**D**), the collared anteater (**E**), the pygmy anteater (**F**), and the aardvark (**G**) display the morphological diversity within this convergent set of species. Branch colors correspond to the following clades: orange—Pholidota; red—Carnivora; yellow—Cingulata; green—Vermilingua; blue—Tubulidentata

**Fig. 2:**
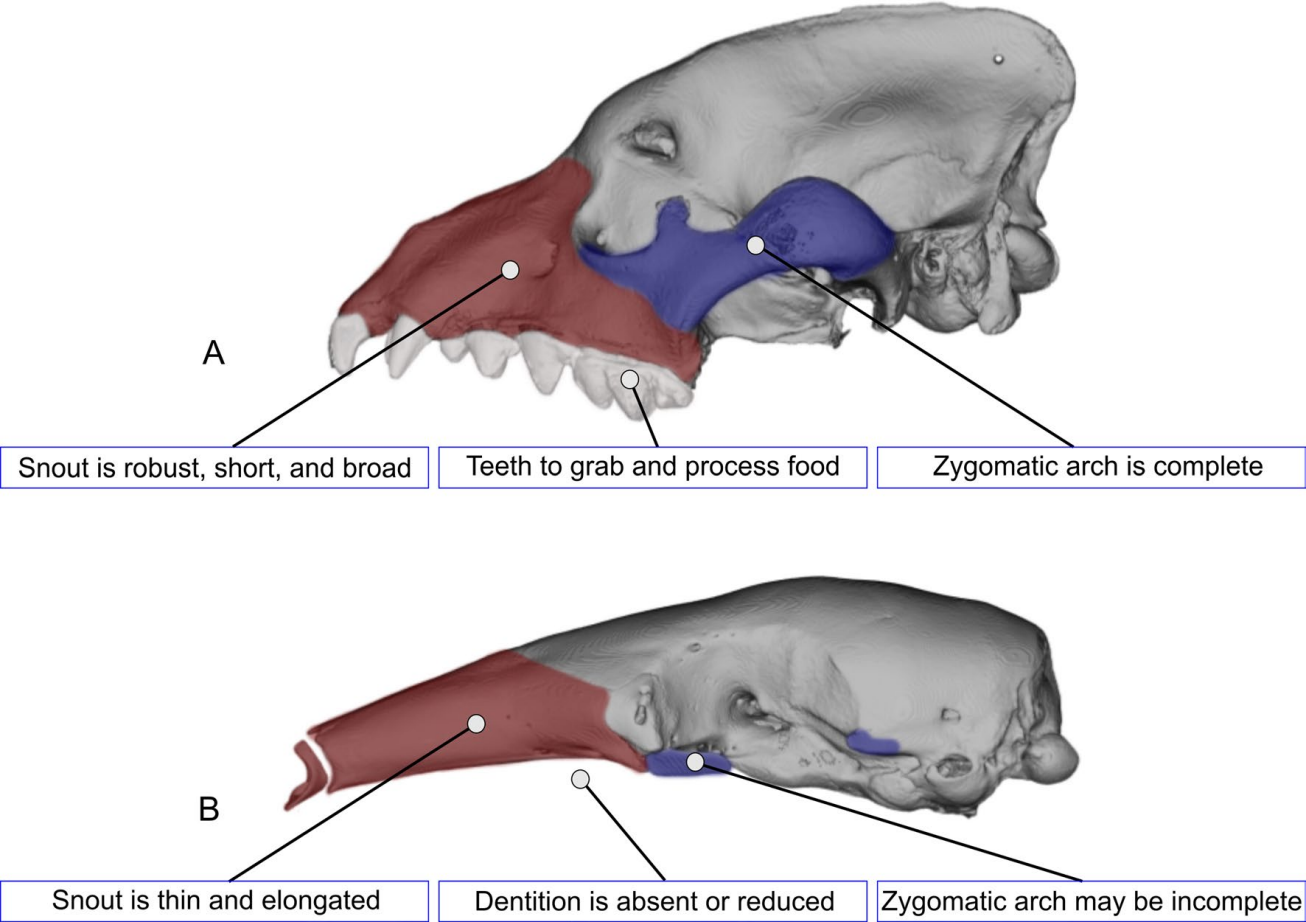
Key differences between the feeding apparatus of non-myrmecophagous and myrmecophagous placentals. Lateral views of the striped hyena (*Hyaena hyaena;* **A**) and the collared anteater (*Tamandua tetradactyla; ***B**). The snout is colored in red and the zygomatic arch elements are colored in blue

Functional and developmental constraints were proposed to act as generators of increased correlation between anatomical traits (e.g., [6, 31]). Therefore, the loss of function could result in an alteration of correlation. Compared to toothed placentals, it could be hypothesized that myrmecophagous mammals present three main differences in covariance-generating processes. First, tooth reduction or absence should decrease the variance generated by tooth development and eruption (e.g., [16, 57]). Second, the loss of masticatory function, adductor muscle insertion areas (e.g., zygomatic arch), and reduction of masticatory muscle volume [58–60] might have induced shifts in the covariance patterns influenced by mandibular adduction. Indeed, in chicken and mice, bone formation depends on functional muscles that perform embryonic muscle contraction [61, 62]. Third, the extreme snout elongation of some myrmecophagous placentals, particularly giant and collared anteaters, might have required changes in integration of traits in the rostrum related to myrmecophagy [5, 63, 64], as somatic growth of bone tissues is one of the main processes contributing to structural covariation on the mammalian skull [3, 4, 65, 66]. To test these predictions, we used geometric morphometrics and both exploratory and confirmatory methods to investigate the previously unexplored modular architecture of the skull in 13 myrmecophagous placental species (Fig. 1 and Additional file 2: Fig. S1; Additional file 3: Table S1 and S2). We used Euclidean Distance Matrix Analysis (EDMA; [67]) to first explore skull covariation without a priori modularity hypotheses and to identify possible common trends in the covariance patterns associated with myrmecophagy (Additional file 3: Table S3). We then tested several a priori-defined modular architectures for each species and compared integration patterns in order to test if the three main morpho-functional shifts associated with myrmecophagy (tooth loss, mastication loss, and snout elongation; Fig. 2 and Table 1) had a quantitative impact on cranial covariation/correlation patterns (e.g., extremely low within-module correlations; presence of less complex modular architectures resulting from growth).

**Table 1:** Cranial features associated with the adaptation to myrmecophagy

Common name	Clade	Tooth loss	Long rostrum	Zygomatic arch
Anteaters	Xenarthra	Yes	Yes	Absent
Giant armadillo	Xenarthra	Partial	Yes	Present
Aardvark	Tubulidentata	Partial	Yes	Present
Pangolins	Pholidota	Yes	Yes/no	Absent
Aardwolf	Carnivora	Partial	No	Present

## Results

### Exploratory analysis of modularity—EDMA

EDMA is an alternative for estimating mean shape, forms, and variance–covariance structure relying on interlandmark distances ([67, 68]). Inter-trait Euclidean distance matrices were used to cluster landmarks and the Gap statistic was used to find the optimal number of clusters (see ‘Materials and Methods’). The Gap test revealed that the skull of myrmecophagous placentals was optimally divided in four to nine clusters (Additional file 3: Table S3), with the exception of *Smutsia gigantea* (Giant pangolin) and *Orycteropus afer* (Aardvark), which were considered to optimally consist of one single cluster. However, this result might be related with a lower Gap statistics value for a partition in two clusters, given that further subdivision highly improved this value (Additional file 2: Fig. S2). For these two species, we defined the compartmentalization of the skull as the lower number of clusters (*k*) corresponding to the plateau of the Gap value distribution (Additional file 2: Fig. S2). According to this criterion, *O. afer* was first divided in six clusters, while *S. gigantea* was divided in five (Additional file 2: Figs. S2 and S3). The calculation of Jaccard coefficients showed that, with the exception of *Phataginus tetradactyla* (Black-bellied pangolin) and *Manis pentadactyla* (Chinese pangolin), the optimal number of clusters retrieved from Gap statistics included unstable groups (< 0.70; Additional file 3: Table S3). When we considered stable clusters only, their final number varied between three and seven (Additional file 3: Table S3). Changing the Jaccard threshold to 0.60 increased the number of clusters retained in nine out of 13 species (Additional file 3: Table S3).

**Fig. 3:**
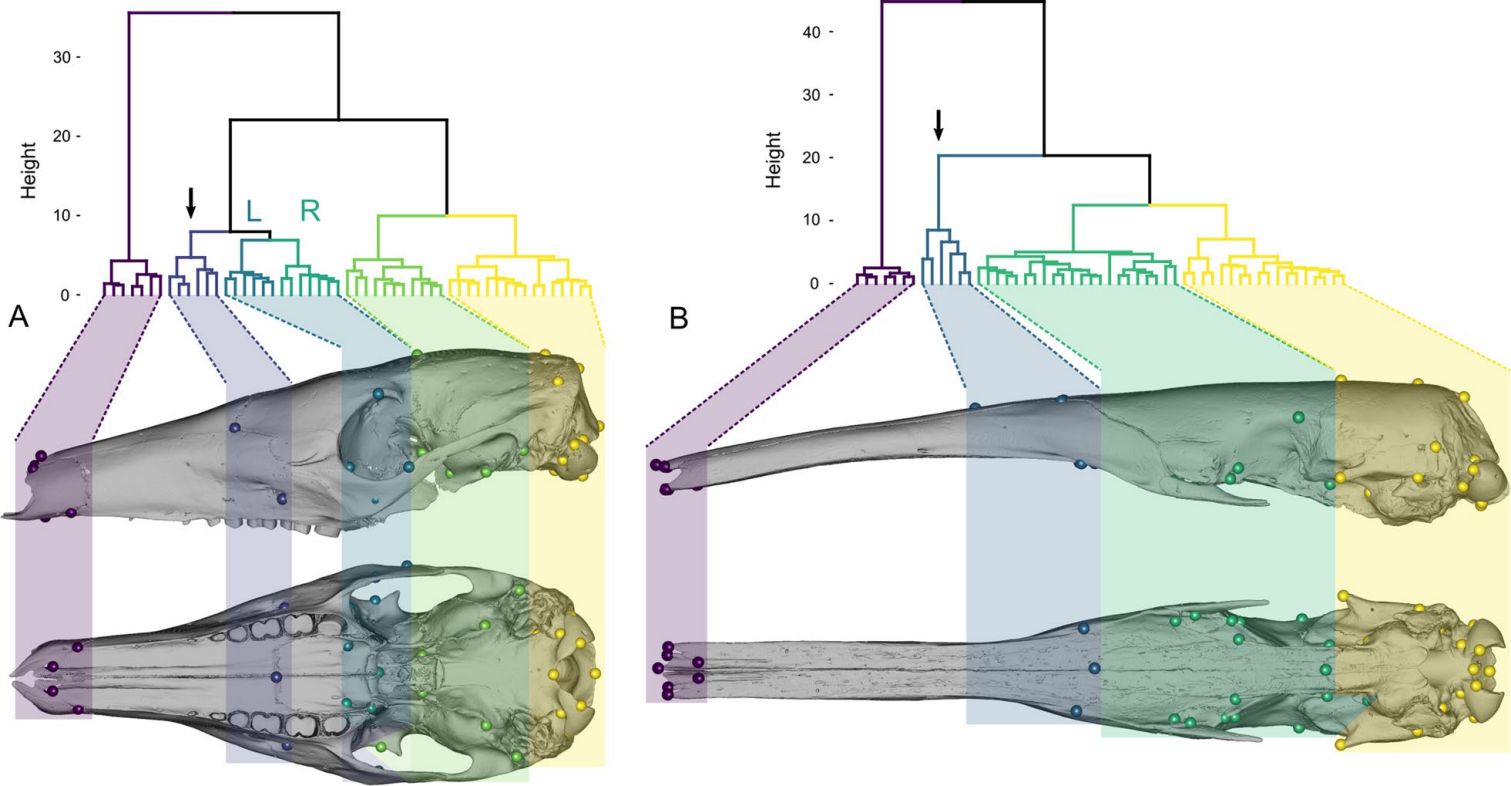
Hierarchical clustering (above) and respective anatomical units (below) resulting from Euclidean distance covariance matrices. *O. afer* presented six clusters (**A**) while *M. tridactyla* presented four clusters (**B**). In both species, a naso-palatine cluster was detected (black arrow). Colors do not correspond to a specific anatomical region and differ from those used throughout the rest of this paper. “L” and “R” represent bilaterally symmetric anatomical clusters

The oro-nasal module was conserved across all taxa. It was composed of the landmarks at the anterior parts of the maxilla and nasal in all species (Fig. 3 and Additional file 2: Fig. S3). In *Priodontes maximus* (Giant armadillo), the oro-nasal module also included the landmarks from the naso-frontal suture, as well as those from the infraorbital foramina (landmarks #3, #23; Additional file 2: Fig. S3).

The molar region of the skull also presented some variability, and was either: (1) a small region including the maxillary foramina (#4, #24), palatal foramina (#6, #25, #7, #26), and the zygomatic process of the maxilla (#41, #52); (2) merged with the naso-palatine region; (3) merged with the orbital region. *Tamandua tetradactyla* (Collared anteater) (Additional file 2: Fig. S4) presented the first condition of an individualized molar module. *Cyclopes didactylus* (Pygmy anteater), *Phataginus tricuspis* (White-bellied pangolin), *Manis javanica* (Sunda pangolin), *M. pentadactyla*, *Smutsia temminckii* (Ground pangolin), and *Proteles cristatus* (Aardwolf) presented the second condition, with a large naso-palatine/molar module, with this cluster also presenting landmarks from the anterior and superior parts of the orbit in *P. tricuspis*, *S. temminckii*, and *P. cristatus* (Additional file 2: Fig. S3). *Myrmecophaga tridactyla* (Giant anteater; Fig. 3B), *Tamandua mexicana* (Northern tamandua), *P. maximus*, *O. afer*, and *S. gigantea* showed the third condition, with a merged molar/orbit region, although a small maxillo-palatine module was present anteriorly in *T. mexicana* and *O. afer* (Additional file 2: Fig. S3). All pangolins, *P. cristatus*, *O. afer*, and *P. maximus* presented maxillary (#41, #52) and squamosal (#12, #30) components of the zygomatic arch within the same cluster, either as a component of the molar region or as part of a molar-orbit cluster (Additional file 2: Fig. S3).

The naso-frontal/maxillo-palatine intersection region presented different partitions among myrmecophagous placentals, with three main patterns: (1) the naso-palatine (#39, #50 and #42), the intermaxillary-interpalatine intersection (#5), and the infraorbital foramina (#3, #23; here treated as part of the maxillo-palatine landmarks for simplicity) formed a separate cluster; (2) the naso-frontal/maxillo-palatine landmarks (hereafter, naso-palatine) were partially merged with the posterior cluster(s); (3) the naso-palatine landmarks clustered with the oro-nasal landmarks; (4) the naso-palatine landmarks were all included in a posterior cluster. The naso-palatine cluster was present in *T. tetradactyla*, *T. mexicana*, *M. tridactyla*, and *O. afer* (Additional file 2: Figs. S3, S4, and 3). In *T. mexicana*, both the maxillary foramina (#4, #24) and the zygomatic processes of the maxillae (#41, #52) clustered with the naso-palatine (Additional file 2: Fig. S3). In *P. tetradactyla*, the naso-frontal landmarks formed an independent cluster, while the maxillo-palatine landmarks grouped with the posterior molar module (Additional file 2: Fig. S3). *P. maximus* presented a merged oro-nasal/naso-palatine cluster (with the exception of landmark #5). On the other hand, the remaining species showed the naso-palatine landmarks grouped with the molar module (Additional file 2: Fig. S3).

*O. afer*, *P. tetradactyla*, and *P. cristatus* presented a bilateral separation of the posterior part of the rostrum and orbit (and the basicranium in *P. tetradactyla*), but all bilateral counterparts were merged within the same large cluster (Additional file 2: Fig. S3).

### A priori modular architecture hypothesis testing

**Fig. 4:**
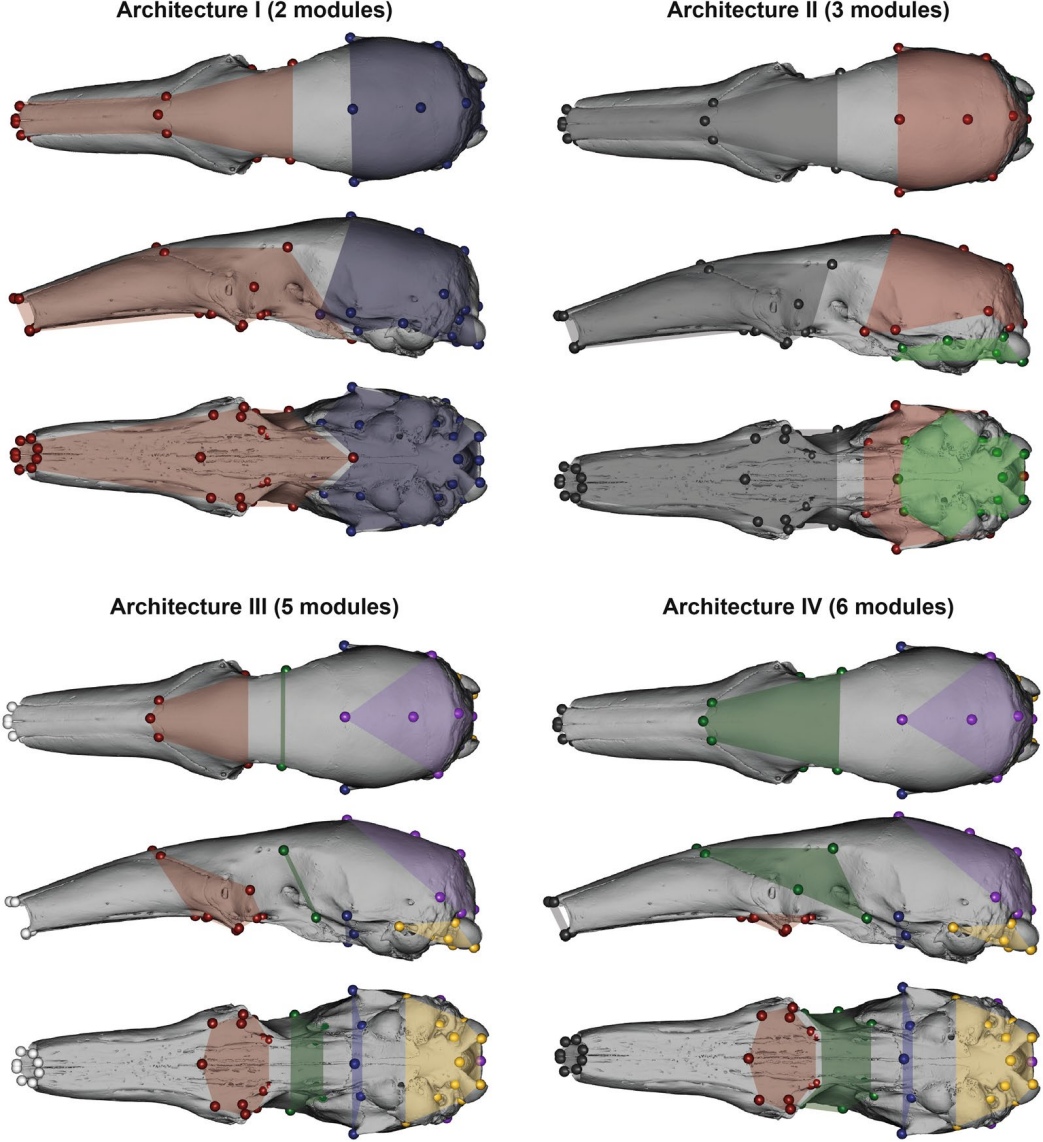
First four modular architectures (I to IV) tested in this paper. See Table 2 for more details. In the face-neurocranium two-module architecture (e.g., Drake and Klingenberg [34]; I): red—face/rostrum; blue—neurocranium and posterior part of the zygomatic arch. In the three-module architecture (Hallgrímsson et al. [30]; II): dark grey—rostrum; red—vault and sphenoid; green basicranium. In the five-module with unintegrated oro-nasal (adapted from Goswami [31]; III): white—unintegrated; red—molar/molar-palate module; green—orbit; blue—zygomatic-pterygoid; purple—cranial vault; yellow—basicranium. In therian six-module (Goswami [31]; IV): dark grey—oro-nasal; remaining colors as in architecture III

**Fig. 5:**
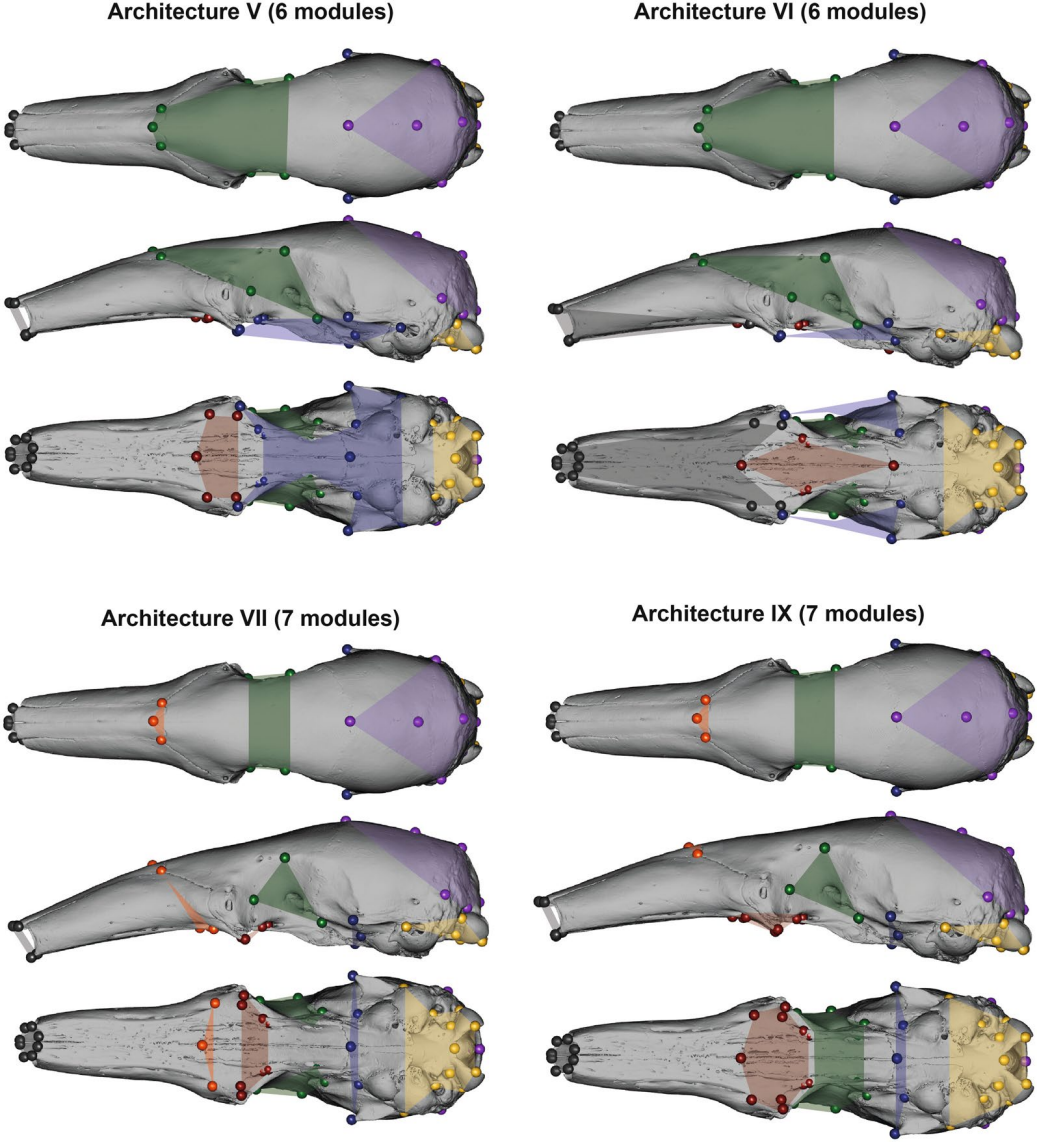
Modular architectures (V, VI, VII, and IX) tested in this paper. In the six-module with fused ptrygoid and zygomatic maxilla (adapted from Goswami [31]; V) and the alternative six-module (adapted from Hallgrímsson et al. [30]; VI): colors are as in architecture IV (Fig. 4). In the seven-module with naso-palatine module (this study; VII) and the seven-module with naso-frontal module (this study; IX): orange—naso-palatine / naso-frontal. Modular architectures VIII and X are not illustrated here as they correspond to modifications of architectures IV and VI by including the naso-palatine and the naso-frontal modules (as in VII and IX), respectively

The interlandmark measurement-based exploratory analysis retrieved different modular architectures per species (Fig. 3 and Additional file 2: Fig. S3). However, the facial region showed a trend towards the clustering of the landmarks of the maxillo-palatal and naso-frontal regions (e.g., *Tamandua*, *M. tridactyla*, and *O. afer*) into a naso-palatine module (Fig. 3 and Additional file 2: Fig. S3). Additionally, *P. tetradactyla* showed an individualized nasal module, similar to the condition found in rhesus macaques [6]. Therefore, this justified the inclusion of four additional seven-module architectures with the naso-palatine module (VII, VIII) and a small nasal module (IX, X; Table 2), corresponding to modified versions of the therian and *macaque phen-gen* architectures, respectively (see Figs. 4 and 5 and Additional file 3: Table S3). Such additions to the previously proposed hypotheses allow for the testing of the detected patterns in a confirmatory framework, which is often considered statistically more robust than exploratory methods [69, 70].

**Table 2:** Modular architectures evaluated with a maximum likelihood method (*EMMLi*)

Architecture	Number of modules	Other designations
I	2	Face-neurocranium
II	3	Face-neurocranium-basicranium
III	5	Rostrum unintegrated
IV	6	Therian
V	6	Therian zyg-zygpte
VI	6	Macaque phen-gen
VII	7	Therian EDMA
VIII	7	Macaque phen-gen EDMA
IX	7	Therian EDMA2
X	7	Macaque phen-gen EDMA2

Architectures are ordered by number of modules

The results of the modular architecture selection by maximum-likelihood (EMMLi) are summarized in Table 3 and Additional file 3: Table S4. Before correcting data for static allometry, EMMLi retrieved a seven-module architecture for all species (VII, VIII, and IX) except for *P. maximus* which showed the six-module therian architecture with a complete zygomatic arch (V; Fig. 5). Architecture VII was the most likely for all anteaters but *M. tridactyla* (IX), for all pangolins except for *S. gigantea* (VIII), as well as for *O. afer*. Static allometry was detected for all species but *P. maximus* and *P. cristatus*. Apart from these species, results reported below relate to the allometry-corrected data (Table 3). Static allometry did not affect the number of retrieved modules except in *S. temminckii*, which presented a therian six-module architecture with a zygomatic arch (V; Table 3 and Additional file 3: Table S4)*.* Allometric-correction also changed the modular architecture of *O. afer*, but without changing the number of modules. Posterior to static allometry correction, the most likely architecture for *O. afer* was the seven-module VIII, which contrasts with architecture VII in presenting a complete zygomatic arch and palatine modules (Fig. 5 and Additional file 2: Fig. S3; Table 3). EMMLi results were consistent when the sample size was reduced to 30 and 15 in the two tested species (Additional file 3: Table S5). Integration values generally increased with lower sample sizes, but these changes are within the previously reported variation ranges [71].

**Table 3:** Static allometry-corrected modular architectures of 13 myrmecophagous mammals

	*N*	*MLi*	*CR*	*ρ* 1	*ρ* 7	*ρ* 2	*ρ* 3	*ρ* 4	*ρ* 5	*ρ* 6	*ρ abs* 1–2	Mean *ρ*
*T. tetradactyla*	74	VII (7)	0.59	0.75	0.18	0.49	0.32	0.27	0.19	0.40	− 0.48	0.37
*T. mexicana*	47	VII (7)	0.68	0.73	0.19	0.47	0.30	0.31	0.21	0.25	− 0.44	0.35
*M. tridactyla*	35	IX (7)	0.59	0.73	0.62	0.39	0.40	0.27	0.28	0.49	− 0.49	0.45
*C. didactylus*	60	VII (7)	0.71	0.67	0.16	0.43	0.22	0.42	0.20	0.21	− 0.25	0.33
*P. maximus*	14	–	–	–	–	–	–	–	–	–	–	–
*O. afer*	40	VIII (7)	0.67	0.34	0.15	0.47	0.29	0.22	0.18	0.33	− 0.17	0.28
*M. javanica*	28	VII (7)	0.66	0.51	0.28	0.38	0.29	0.30	0.16	0.25	− 0.30	0.31
*M. pentadactyla*	27	VII (7)	0.73	0.50	0.24	0.27	0.29	0.15	0.17	0.20	− 0.15	0.26
*S. temminckii*	15	V (6)	0.83	0.52	–	0.27	0.25	0.16	0.22	0.20	− 0.19	0.23
*S. gigantea*	12	VIII (7)	0.80	0.47	0.32	0.69	0.38	0.46	0.24	0.27	− 0.29	0.40
*P. tricuspis*	72	VII (7)	0.63	0.41	0.22	0.40	0.22	0.31	0.17	0.20	− 0.18	0.28
*P. tetradactyla*	17	VII (7)	0.79	0.53	0.27	0.34	0.27	0.40	0.26	0.24	− 0.23	0.33
*P. cristatus*	24	–	–	–	–	–	–	–	–	–	–	–

Number of specimens (*N*), most likely modular architectures recovered with EMMLi (*MLi*), covariance ratio (*CR*), within-module absolute correlations (*ρ*), correlation between oro-nasal and molar-palate modules (*ρ abs*), and mean within-module correlation (Mean *ρ*). (1) Oro-nasal/rostrum, (2) molar-palate, (3) orbit, (4) zygomatic-pterygoid, (5) vault, (6) basicranium, (7) naso-palatine (VII/VIII) / nasal (IX/X). All CR values were significant

*Tamandua* spp., *C. didactylus*, *Phataginus* spp., and *Manis* spp. presented architecture VII (Table 4). This consists of the therian functional modules [31] with an additional naso-palatine module derived from the EDMA analyses (seven modules in total; Fig. 5). The most likely architecture recovered for *P. maximus* and *S. temminckii* (V) differs from architecture VII in which the naso-palatine module is split between the orbit (naso-frontal landmarks; #39/50, #42) and the molar-palate (maxillo-palatine midline and infraorbital foramen landmarks; #5, #3/23). Additionally, the posterior tip of the zygomatic process of the maxilla (#41/52) is included in the zygomatic-pterygoid module (Fig. 5). *O. afer* and *S. gigantea* seven-module architecture (VIII) differs from architecture VII in presenting the landmarks of the maxillary foramen (#4/24) included in the oro-nasal module, the posterior limit of the interpalatine suture (#9) included in the molar-palate module, and the zygomatic process of the maxilla (#41/52) is integrated with the zygomatic process of the squamosal (#12/30) and the foramen ovale (#13/31).

**Table 4:** Static allometry-corrected (when detected) integration magnitudes of the oro-nasal (1), naso-palatine or nasal (7), and molar-palate (2) modules in myrmecophagous mammals with a 7-module architecture

	*N*	*ρ* 1	*ρ* 7 (VII / IX)	*ρ* 2 (VII / IX)	*ρ* 1–7 (VII / IX)	*ρ* 2–7 (VII / IX)
*T. tetradactyla*	74	0.75	0.18	0.49	0.30	0.12
*T. mexicana*	47	0.73	0.19	0.47	0.31	0.15
*M. tridactyla*	35	0.73	0.35 / 0.62	0.45 / 0.39	0.45 / 0.37	0.19 / 0.10
*C. didactylus*	60	0.67	0.16	0.43	0.17	0.15
*O. afer*	40	0.34	0.15	0.47	0.16	0.14
*M. javanica*	28	0.51	0.28	0.38	0.19	0.11
*P. pentadactyla*	27	0.50	0.24	0.27	0.17	0.12
*S. gigantea*	12	0.47	0.32	0.69	0.33	0.37
*P. tricuspis*	72	0.41	0.22	0.40	0.18	0.12
*P. tetradactyla*	17	0.53	0.27	0.34	0.21	0.16
*P. cristatus*	24	0.46	0.23 / 0.49	0.31 / 0.28	0.13 / 0.13	0.19 / 0.13

Number of specimens (*N*), within-module absolute correlations (*ρ*), and correlation between oro-nasal, and molar-palate modules with the naso-palatine (*ρ* 1–7, *ρ* 2–7)

For each species, CR modularity tests [72] showed that the most likely architectures retrieved by EMMLi were significantly modular (Table 4). Significant modular signals were obtained both prior to and after allometric correction (Table 3 and Additional file 3: Table S4). The strongest modular signal, as obtained from the calculation of *Z*_*CR*_ [73], corresponded to the most likely architectures retrieved by EMMLi (Table 5 and Additional file 3: Table S6), except in *P. maximus*, *M. pentadactyla*, *S. temminckii*, and *P. tetradactyla*. In these four species, the lowest *Z*_*CR*_ values did not coincide with the most likely architecture (Table 5 and Additional file 3: Table S6), corresponding instead to architectures VII (*P. maximus*) and VIII (*M. pentadactyla*, *S. temminckii*, *P. tetradactyla*). For all species, the most likely architecture presented significantly larger modular strength when compared to a non-modular hypothesis (Additional file 3: Table S6), as expected based on previously calculated CR significance (Table 3 and Additional file 3: Table S4). The most likely architectures (V-VIII) presented a significantly (or marginally significant) lower *Z*_*CR*_ than architectures I and II in *Tamandua* spp., *C. didactylus*, *O. afer*, *M. javanica*, *Smutsia* spp., *P. tetradactyla*, and *P. cristatus* (Additional file 3: Table S6). The difference between the most likely architecture and architecture I was nearly significant in *P. tricuspis*. In *M. tridactyla*, *P. maximus*, and *M. pentadactyla* no significant difference was found among the modular architectures (Additional file 3: Table S6).

**Table 5:** Static allometry-corrected (when detected) effect size (Z_CR_) for the most likely architecture retrieved by EMMLi compared to each other and  to the therian six-module [31] and Macaque phen-gen [30] hypotheses

	*MLi*	*Z*_*CR*_	IV	V	VI	VII	VIII
*T. tetradactyla*	VII (7)	**−** **9.65**	5.74 × 10^–1^	1.76 × 10^–1^	3.68 × 10^–1^	1.00	7.33 × 10^–1^
*T. mexicana*	VII (7)	**−** **8.76**	3.87 × 10^–1^	1.72 × 10^–1^	3.72 × 10^–1^	1.00	8.48 × 10^–1^
*M. tridactyla*	VII (7)*	**−** **5.92**	8.70 × 10^–1^	5.87 × 10^–1^	7.77 × 10^–1^	1.00	6.35 × 10^–1^
*C. didactylus*	VII (7)	**−** **9.04**	7.02 × 10^–1^	6.61 × 10^–1^	1.83 × 10^–1^	1.00	5.21 × 10^–1^
*P. maximus*	V (6)	− 5.22	9.40 × 10^–1^	1.00	9.16 × 10^–1^	6.26 × 10^–1^	7.82 × 10^–1^
*O. afer*	VIII (7)	**−** **8.47**	4.85 × 10^–1^	4.82 × 10^–1^	7.74 × 10^–1^	6.48 × 10^–1^	1.00
*M. javanica*	VII (7)	**−** **8.37**	6.31 × 10^–1^	8.14 × 10^–1^	8.57 × 10^–1^	1.00	8.42 × 10^–1^
*M. pentadactyla*	VII (7)	− 6.51	8.10 × 10^–1^	7.88 × 10^–1^	7.40 × 10^–1^	1.00	4.76 × 10^–1^
*S. temminckii*	V (6)	− 5.85	7.90 × 10^–1^	1.00	6.87 × 10^–1^	3.46 × 10^–1^	3.43 × 10^–1^
*S. gigantea*	VIII (7)	**−** **7.07**	1.76 × 10^–1^	1.69 × 10^–1^	5.40 × 10^–1^	6.67 × 10^–1^	1.00
*P. tricuspis*	VII (7)	**−** **8.04**	7.79 × 10^–1^	8.55 × 10^–1^	7.01 × 10^–1^	1.00	9.44 × 10^–1^
*P. tetradactyla*	VII (7)	− 7.28	5.10 × 10^–1^	6.73 × 10^–1^	5.69 × 10^–1^	1.00	8.90 × 10^–1^
*P. cristatus*	VII (7)*	**−** **7.40**	6.04 × 10^–1^	7.24 × 10^–1^	3.62 × 10^–1^	1.00	9.10 × 10^–1^

For each most likely architecture, the *p*-values of the modular signal strength comparison are given. Most likely modular architectures (*MLi*), effect size calculated with the compare. CR function (*Z*_*CR*_, [73]), *p*-value for the comparison with the remaining modular hypothesis (IV–VIII). The lowest ZCR values are in bold. *VII is presented here instead of IX; see discussion on architecture IX

### Within- and between-modules integration

**Fig. 6:**
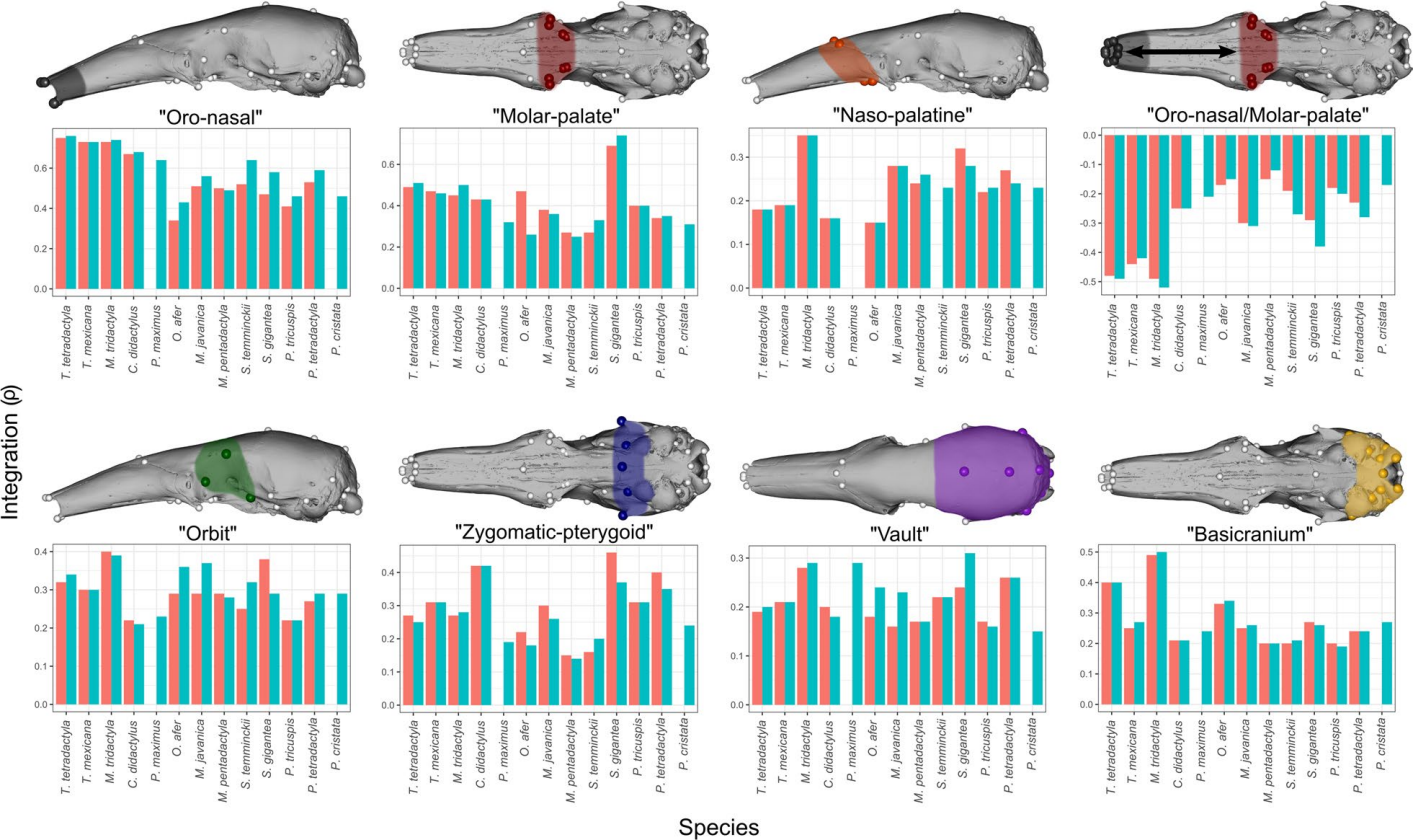
Bar-plots of the within-module correlations (integration) for the seven modules recovered from the maximum-likelihood approach. Blue bars correspond to raw values and coral bars correspond to static allometry-corrected values. Empty cases mean absence of modules or lack of static allometry in a species. The anatomical region corresponding to each module is depicted on a skull of *T. mexicana* in dark grey (oro-nasal), red (molar-palate), orange (naso-palatine), green (orbit), blue (zygomatic-pterygoid), purple (cranial vault), and yellow (basicranium). The correlation between the oro-nasal and the molar-palatine modules is represented by a twin-headed black arrow. White spheres represent the set of landmarks used in this study (Additional file 3: Table S2)

Regarding integration strength (Fig. 6), the oro-nasal region displayed the strongest integration values relative to overall skull integration (Table 3 and Additional file 3: Table S3; Fig. 6). Anteaters presented the highest relative integration values for this module (0.67 < ρ < 0.75). *P. tetradactyla* (ρ = 0.53) showed the highest value among pangolins, while *P. tricuspis* showed the lowest (ρ = 0.41). *O. afer* was the species with the least integrated oro-nasal module (ρ = 0.34).

*S. gigantea* presented the highest integration for the molar-palate module (ρ = 0.69; Table 4), above the average for the remaining species (Table 4; Fig. 6). This value is especially high when compared with the remaining pangolins, for which integration for the molar-palate model varied between 0.27 < ρ < 0.40 (Table 4). Anteaters showed a relatively well-integrated molar-palate (0.39 < ρ < 0.49), with the smaller integration value corresponding to the molar-palate module of *M. tridactyla* including the interfrontal-intermaxillary (#5) and the inferior alveolar canal landmarks (#3, #23). *P. cristatus* (ρ = 0.31) and *P. maximus* (ρ = 0.32) displayed molar-palate integrations close to the lower levels of our sample range found for *M. pentadactyla* and *S. temminckii* (ρ = 0.27).

The orbit module was generally less integrated than the oro-nasal and the molar-palate modules (Table 4). Nevertheless, *S. gigantea* (ρ = 0.38) and *M. tridactyla* (ρ = 0.40) presented relatively well-integrated orbits, showing ρ values close to their species mean integration (Table 4; Fig. 6). *C. didactylus* and *P. tricuspis* showed the least integrated orbits (ρ = 0.22; Table 3).

The zygomatic-pterygoid module was generally poorly integrated, except for *C. didactylus*, *S. gigantea*, and *P. tetradactyla* that show levels of integration (0.40 < ρ < 0.46) well above the average module integration (Table 4; Fig. 6). *M. pentadactyla* and *S. temminckii* were the species with the lowest integration magnitudes (ρ = 0.15, ρ = 0.16) for this module.

The vault was the least integrated module, overall (Table 3 and Additional file 3: Table S4; Fig. 6). *P. maximus* and *M. tridactyla* showed the higher integration magnitudes for the vault (ρ = 0.29, ρ = 0.28). The species with the least integrated cranial vault was *P. cristatus* (ρ = 0.15). This module often presented between-module integration magnitudes close to or higher than the within-module integrations, but these results are most likely related to an artefact resulting from the low sampling of this region [25, 74].

The basicranium was relatively well-integrated in *T. tetradactyla* and *M. tridactyla* (Table 3; Fig. 6), with the latter showing the highest magnitude (ρ = 0.49). Pangolins revealed relatively low integration magnitudes for the basicranium, with *P. tricuspis*, *M. pentadactyla*, and *S. temminckii* presenting the lowest values of the whole sample (ρ = 0.20). *C. didactylus* also showed a very weakly integrated basicranium (ρ = 0.21).

In species presenting a naso-palatine module, this region was weakly integrated, with anteaters presenting lower magnitudes when compared to pangolins (Fig. 6). Nevertheless, integration values were below the average intra-module integration in both clades. In all species, except *S. gigantea*, the naso-palatine module was more strongly correlated with the oro-nasal than with the molar-palate module (Table 4). This pattern was especially pronounced in *Tamandua* spp. (Fig. 6), in which the correlation between the naso-palatine and the oro-nasal was at least 30% higher than the naso-palatine intra-modular correlation (Table 4). Additionally, the correlation between the naso-palatine and oro-nasal modules was twice as high as between the former and the molar-palate in *Tamandua* spp. (Table 4). In *O. afer* and *C. didactylus*, integration magnitudes of both naso-palatine, oro-nasal/naso-palatine, and molar-palate/naso-palatine were near equal (Table 4). *M. tridactyla* and *P. cristatus* presented a nasal module which was relatively strongly integrated in both cases (ρ = 0.62 and ρ = 0.49, respectively). While in *P. cristatus* the nasal module was weakly integrated with both oro-nasal and molar-palate modules (ρ = 0.13), *M. tridactyla* showed a pattern similar to that of *Tamandua* spp., with the nasal module being much more strongly correlated to the oro-nasal (ρ = 0.37) than to the molar-palate (ρ = 0.10). Intermodular correlations for the most likely hypotheses for each species are provided on Additional file 3: Tables S7 to S13.

When the absolute correlations between the oro-nasal and molar-palate regions were considered, they were by far more negative in the long-snouted *Tamandua* spp. and *M. tridactyla* (− 0.44 ≥ ρ ≥ − 0.49) than in the rest of the sample (− 0.15 ≥ ρ ≥ − 0.30; Table 3; Fig. 6). *M. pentadactyla* presented the least negative correlations (ρ < − 0.13), while *M. tridactyla* showed the most negative (− 0.49). *S. gigantea* was the only species in which allometric correction drastically reduced absolute correlation values (ρ = − 0.38 vs ρ = − 0.29).

## Discussion

### Modular architectures across myrmecophagous species

Exploratory methods were used in the early studies to address the modularity of the mammalian skull [6, 31]. However, hypothesis testing methods were then increasingly used to assess if sets of traits exhibit modular structures compared to a random organization [13, 72, 75, 76]. Given that confirmatory methods are considered statistically powerful tools (e.g. [71, 72, 77]), the combination of methodologies used here enabled us to test the strength of the hierarchical clustering patterns, as well as to assess the validity of patterns from non-superimposed data (EDMA) in a Procrustes alignment framework (e.g., EMMLi, CR, RV). Overall, all clusters obtained from EDMA were spatially coherent, with adjacent structures being integrated with each other and separated from the more remote landmarks, as in previous studies using clustering methods [6, 31, 78]. Although the optimal number of clusters varies within a range of thresholds for the Jaccard similarity index (Additional file 3: Table S3), our option for a relatively high threshold level (J > 0.70) allows for a good degree of confidence concerning the biological validity of the recovered modules. Using lower thresholds (e.g., J > 0.60) may give insights on finer subdivisions of the skull, but the landmark composition of these modules would be highly doubtful (i.e., low stability of these subdivisions with 10,000 permutations [79]).

Modular architectures recovered for myrmecophagous placentals with the confirmatory maximum-likelihood approach are concordant with previously published studies of non-myrmecophagous species [6, 31, 71, 80–83]. We showed that the modular organization of their skull varies between six to seven modules. Most of the species presented seven-module architectures, seven of them corresponding to the therian-based seven-module architecture (VII; Fig. 5), two to the modified version of the *macaque phen-gen* seven-module architecture (VIII), and two others to the therian seven-module architecture with a separate nasal module (IX; Fig. 5). While the CR test found significant modular signals for all most-likely architectures, the strongest modular signal as measured by *Z*_*CR*_ did occasionally differ from the ones recovered from the maximum-likelihood approach (Table 5 and Additional file 3: Table S6). These exceptions were *P. maximus* (Giant armadillo), *M. tridactyla* (Giant anteater), and three pangolin species *M. pentadactyla* (Chinese pangolin), *S. temminckii* (Ground pangolin), and *P. tetradactyla* (Black-bellied pangolin). Although contrasting, the results from EMMLi and *Z*_*CR*_ are not contradictory, as the two tests perform a model choice based on two different criteria [71, 73]. However, unlike maximum-likelihood, CR effect sizes were not statistically different between modular architectures with six and seven modules (Table 5 and Additional file 3: Table S6). These apparently more conservative results obtained with *Z*_*CR*_ might be explained by two factors, one associated with the dataset, and the other related to how *Z*_*CR*_ determines the favoured modularity hypothesis [72, 139]. Firstly, the differences between architectures III to X, varying between six to seven modules, try to capture significant changes in covariance associated with a few landmarks, instead of large modules (see Additional file 3: Table S2; in *Z*_*CR*_ analyses, architecture III = architecture IV, as ‘NAs’ are interpreted as a module). In fact, these eight architectures (III–X) are all variations of two previously proposed hypotheses [30, 31]. Given that major modules are conserved, it is not surprising that modular strength is imperceptible to *Z*_*CR*_, provided that a few landmarks are likely not enough to increase the modular strength (lower *Z*_*CR*_) in one specific module. On the other hand, and most importantly, a six-module architecture might separate two highly correlated landmark clusters and still yield a lower than or significantly indistinguishable *Z*_*CR*_ than any other architecture with a finer modular architecture (e.g., 7 modules; [139], p. 16), which gives a better account of the between-modules correlations.

Although the number of clusters is not straightforward to determine [84–86], hierarchical clustering enabled us to pinpoint landmark associations from the naso-palatine region that consistently clustered apart from our a priori defined molar-palate module. The merging of the maxillo-palatine (landmark #5) and infraorbital canal (#3/23) landmarks with the naso-frontal region in architectures VII and VIII was missing from previously tested hypotheses, with the naso-frontal landmarks being typically associated with the orbit module [31, 80, 83] or a nasal bone module [6, 71, 82]. A similar arrangement was retrieved for *P. tetradactyla*, with the naso-frontal landmarks forming a completely separate cluster (Additional file 2: Fig. S3). This nasal cluster resembles the small nasal module present in rhesus macaques [6] and dolphins [82]. However, our identified nasal module differs in consisting solely of the naso-frontal landmarks, while those in rhesus macaques and dolphins also include the anterior part of the nasals. This is probably explained by the small length of nasals in rhesus macaques and dolphins, in which all landmarks are spatially close and therefore more susceptible to covary and cluster together (e.g., [69, 78]). In all our taxa, landmarks delimiting the anterior edge of the nasals were found to consistently cluster with anterior maxillary landmarks (Fig. 3 and Additional file 2: Fig. S3). *M. tridactyla* and *P. cristatus* (Aardwolf) displayed a small naso-frontal module and an intact molar-palate region (IX; Table 3), but such architecture selection should, however, be taken with caution. In our dataset, the nasal module consists of only three landmarks, two of them bilateral (#39/50). Therefore, pronounced variance on a single landmark may have a disproportionate impact on the estimated likelihood of the modular architecture. The hierarchical clustering resulting from interlandmark distance correlations (Fig. 3A and Additional file 2: Fig. S3) shows that, in *P. tetradactyla*, the naso-frontal cluster is closely associated with the remaining molar-palate landmarks (unlike in *M. tridactyla*; Fig. 3B). Curiously, *P. tetradactyla* was found to fit a seven-module architecture with a naso-palatine module (VII; Table 4). When naso-frontal bearing architectures (IX, X) were removed from the analyses, the maximum likelihood approach recovered a naso-palatine module in both *M. tridactyla* and *P. cristatus* (VII; Table 3).

Overall, our results showed that the use of exploratory methods is a good complement to a priori hypothesis testing. These methods allow us to reveal patterns that are not predicted in developmental, functional, or even physiological hypotheses [78]. Specifically, the presence of a naso-palatine module recovered from EDMA clusters was validated by two confirmatory methods (maximum-likelihood and covariance ratio). Our two-pronged approach enabled us to propose a new modular architecture that could be tested in other mammalian datasets. Additionally, the naso-palatine module is potentially useful to explain integration patterns in the rostrum of myrmecophagous placentals. Nevertheless, in view of results yielded based on the *Z*_*CR*_ (which showed that the modular strength of architectures IV to X are similar; Table 5 and Additional file 3: Table S6) and the statistical robustness of this method (Adams & Collyer, 2019), we recommend caution when testing a modularity hypothesis including the naso-palatine module and encourage the analysis of intermodular correlations (Table 4) to support such a decision.

### Flexible conservatism in myrmecophagous placentals

Given that the skull and the masticatory apparatus perform virtually the same functions across tetrapods [45], especially in mammals [87], the maintenance of a functional partition of the skull across all mammals is beneficial in promoting the evolvability of functionally related structures [8, 88]. Anteaters are a good example of modular conservatism, with *T. tetradactyla* (Collared anteater) and *C. didactylus* (Pygmy anteater) showing completely different skull shapes (Fig. 1E and F), but a similar underlying seven-module cranial architecture (VII). In fact, considerable shape change can occur while phenotypic modularity patterns are maintained, as a result of extreme directional selection acting on conserved partitions [89]. Therefore, our findings that modularity patterns are mostly conserved across our sample are in line with previous findings in mammals [32] and the common functional and developmental constraints applying to the mammalian clade [3, 6]. However, the modular architectures retrieved for the myrmecophagous species also reflect slight changes in covariation patterns in the anterior half of the skull. Our results thus suggest that extreme rostrum elongation and tooth loss might have affected covariance patterns in some myrmecophagous placentals.

*Tamandua* spp. present naso-palatine landmarks more correlated to the oro-nasal module than with the molar-palatine region (Table 4). *M. tridactyla* presents similar between-modules integration patterns as *T. tetradactyla*, both when considering architectures with a naso-frontal (IX) or a naso-palatine module (VII). Most importantly, in *Tamandua* spp., the correlation between the naso-palatine and the oro-nasal modules is higher than the naso-palatine intramodular correlation, with *M. tridactyla* showing a similar trend when considering a naso-palatine module (Table 4). In contrast, the naso-palatine of *C. didactylus* displays similarly low intra- and intermodular correlations. This implies that the oro-nasal and naso-palatine modules could be considered as a single functional/developmental unit [2] in long-snouted myrmecophagid anteaters. A well-integrated rostrum module (oro-nasal + naso-palatine) might translate the preservation of covariance generated early during ontogeny (orofacial region; [90]). The primary and secondary palates originate from the bilateral maxillary prominences and their interaction with the lateral nasal prominences, which in turn form from the splitting of the fronto-nasal prominence [91]. Covariance introduced by such interactions might have persisted into adulthood in myrmecophagous species, given the absence of covariance generated by dental eruption [92] through bone resorption and osteogenesis [93, 94]. Therefore, the association of the maxillo-palatal region landmarks with those of the naso-frontal region could be the result of facial prominences outgrowth and fusion [3]. In addition, the loss of mastication in myrmecophagous placentals [60, 95] likely reduces the mechanical stress applied to the molar region during adduction. Such reduced strain might have also reduced covariances generated by bone remodelling as a response to masticatory stress [96]. However, the absence of a strong oro-nasal/molar-palatine integration in similarly toothless pangolins mitigates the assumption of a simple link between shifts of covariance patterns and tooth loss or reduction of masticatory function. In this clade, the between-module correlations of the oro-nasal/naso-palatine complex are weaker than the within-module correlation of the naso-palatine (Table 4; Fig. 6). In *S. gigantea* (Giant pangolin), the naso-palatine is more strongly correlated with the molar-palatine, a pattern similar to that of *P. cristatus* (Table 4). Additionally, *S. temminckii* exhibits a six-module architecture in which the nasopalatine landmarks are merged with the orbit and molar-palate modules (e.g., Fig. 2), similar to previously published hypotheses on placental mammals [31]. While development might release toothless placentals from constraints related to tooth eruption, the myrmecophagid pattern of integration may stem from completely different developmental processes, such as those involved in the allometric growth of the rostrum in mammals (see ‘Morphological integration and allometry’).

Another major feature of the convergent skulls of anteaters and pangolins is the absence of an ossified zygomatic arch (e.g., [95, 97]). Mandibular adductor muscles, like the masseter [98], apply significant mechanical strain, increasing the rate of periosteal deposition [99, 100] and generating mastication-induced osteogenesis [3]. We show that the absence of a module including a complete zygomatic arch is a frequent pattern among myrmecophagous placentals (Table 3). Such absence is concordant with the evolution of adduction-less mandibular motion in anteaters and probably pangolins too [95], which contrasts with some previous studies on mammalian modularity [31, 80]. Although masticatory function appears to explain part of the variation of the zygomatic region, it is hardly the sole explanatory variable justifying the presence/absence of a zygomatic-pterygoid module with a complete zygomatic arch. Several myrmecophagous species (*Smutsia* spp., *O. afer*, *P. maximus,* and *P. cristatus*) presented a complete zygomatic-pterygoid module, despite displaying contrasting masticatory biomechanics. In ground pangolins (*Smutsia* spp.), mandibular movement is reduced and mastication likely absent [101], while *O. afer* (aardvark) is known to perform mandibular adduction [50]. The biomechanics of *P. maximus* remains elusive but the masseter might still have a role in anterior direction movements, although its mandibular movements hardly correspond to those on a typical mammalian mastication cycle [53]. *P. cristatus* displays mandibular adduction and an ossified zygomatic arch [102]. Its zygomatic-pterygoid module did not include the anterior part of the arch (Table 3), which is similar to that in other carnivores [31, 83, 103]. Such an exclusion—and subsequent absence of a zygomatic arch module—in chewing placentals could be explained by two interacting factors: (1) an exclusion of the jugal from the landmarked structures (e.g., this study; [31]) and (2) genetically-underlied variation patterns that vary across taxa [104]. Parr et al. (2016; [80]) showed that the jugal bone could consist of an individual module in dogs. While this result highlights an artefact of a non-landmarked jugal in our dataset, it also suggests that the dissociation observed between the zygomatic processes of the maxilla and the squamosal in *P. cristatus* could be due to the presence of an additional jugal module. On the other hand, a landmarked jugal bone would likely be integrated within the zygomatic arch module of *P. maximus* and *O. afer*. The absence/presence of a zygomatic arch module is, therefore, likely to be a result of phylogenetically constrained developmental processes [104] rather than function-related factors (i.e., mastication).

Our results show that morphological changes linked to dietary convergence did not directly translate into convergent modular patterns in the skull of ant- and termite-eating placentals. In particular, the naso-palatine module in myrmecophagid anteaters is twice more strongly integrated with the oro-nasal than with the molar-palatine, while the equally toothless pangolins presented either a less patent difference or a stronger integration between the naso-palatine and the molar-palate (*S. gigantea*; Table 4). Furthermore, the presence of a zygomatic arch module in non-chewing species (*P. maximus*, *S. temmincki*, *S. gigantea*) and its absence in those presenting a typically mammalian mandibular adduction (*P. cristatus*) suggests that changes in covariation patterns putatively linked to functional shifts (e.g., loss of mastication) may not translate into detectable quantitative differences in correlation matrices. Given that skull development is mostly conserved across mammals (e.g., [105]), changes in modular architectures and integration likely result from the interaction between developmental processes [4]. Therefore, modularity can be conserved and flexible at the same time [89]. This flexible conservatism of the placental skull can explain why tooth loss and snout elongation may have contributed to a less modular rostrum in myrmecophagid anteaters, while resulting in a typical therian pattern in pangolins. Such slight deviations of the therian pattern were previously found in other long snouted species, such as the common dolphin (*Delphinus delphis*; [82]). In the latter, a separate nasal module is present, with the maxillo palatine landmarks being unintegrated (based of a hierarchical clustering approach; [82]). Further investigations using our methodological approach (EDMA + hypothesis testing) and including all long snouted placental taxa might be key to reveal developmental shifts underlying this flexibility in the mammalian skull.

### Morphological integration and allometry

Previous studies highlighted the role of allometry as a major component of phenotypic integration (e.g., [32, 103]). Our results confirm that size variation is an important factor influencing integration magnitude in the mammal skull, and suggest that the impact of allometric growth is differentially distributed in the skull. As in other mammal clades (e.g., [31, 32]), the oro-nasal module of myrmecophagous species is more integrated than the remaining ones. The stronger integration of the oro-nasal module is explained by ontogenetic allometry and represents a pattern common to all mammals [63, 64]. In our dataset, the strong correlation of this module could also result from its landmarks being spatially contiguous (except for *P. cristatus*). Nevertheless, the extreme rostrum elongation of the skull in myrmecophagous species was corroborated by the negative correlation between the oro-nasal and the molar-palate modules (sensu [31]; Fig. 6). These correlations were more negative in *M. tridactyla* and the two *Tamandua* species compared to all the remaining species (Tables 4 and 5; Figs. 5 and 6). Reeve [107] showed that myrmecophagid anteaters (especially *M. tridactyla*) present distinct allometric trajectories when compared to their sister clade *C. didactylus*. A previous study also associated the long rostrum in *M. tridactyla* with a covariance structure that diverged from the remaining xenarthrans [106]. The negative correlation between the oro-nasal and the molar-palate modules suggests a strong ontogenetic allometric effect on the rostrum region, reflecting the opposite directions of variation in its anterior and posterior parts. While somatic growth is one of the main generators of phenotypic covariance across mammals [3, 4, 108], we show that within- and between-modules integration levels of the myrmecophagid snout remained practically unchanged after allometric correction (Table 3 and Additional file 3: Table S4). Main changes related to size variation often occur early in ontogeny [3, 109], but studies on prenatal and early postnatal cranial development of anteaters are currently missing. However, given the ubiquity of developmental processes and allometric patterns across mammals [64], explanations for myrmecophagid integration patterns may be tentatively extrapolated from other clades.

Although early stages of tooth development occur in anteaters [110, 111], osteogenesis associated with that process (e.g., trabecular bone) is much reduced [47]. Therefore, the reduction of covariance introduced by tooth development might have released the naso-palatine module to co-vary more with the oro-nasal region (e.g., anteaters), under the influence of the midfacial fetal growth. Experimental work in mice showed that endochondral ossification and interstitial growth of the nasal septum promote facial growth away from the neurocranium [112]. A similar relationship between midfacial growth and snout length was observed in rabbits [113, 114]. In addition, Smith et al. [105] suggested that the direction of chondrocyte proliferation from the posterior midface differs between short and long-snouted mammals. All these studies suggest that midface growth is an important covariance generator during the development of a long rostrum. Recently, Camacho et al. [115] showed that rostrum elongation in long-faced nectar-eating bats (Glossophaginae) represents a peramorphic change (i.e., hypermorphosis) resulting from the extension of the progenitor cell proliferation period of the midface region. Glossophaginae were also shown to present highly divergent skull covariation patterns among phyllostomid bats [116]. Interestingly, nectar-eating bats share a number of convergent traits with anteaters, such as extremely elongated rostra, reduced dentition, and extensible tongues attached to the xiphisternum [117, 118]. We therefore hypothesize that the strong negative correlations between the oro-nasal and molar-palatine modules in myrmecophagid anteaters might result from a “glossophagine-like” midface hypermorphosis during myrmecophagid snout development. This could also explain why the strong between-module integration of the oro-nasal/naso-palatine regions is present in myrmecophagid anteaters (*M. tridactyla* + *Tamandua*). Other mammal clades, such as carnivores, include clades with divergent covariation patterns [119] putatively associated with less extreme face elongation [120]. Although the sole study on anteater allometric patterns [107] appears to suggest that myrmecophagid snout elongation evolved by peramorphosis through acceleration (e.g., speeding up cell proliferation; [121]), that study lacks a comparison with other xenarthrans. Until further investigation, we propose that the hypothetical scenario of snout elongation by hypermorphosis might explain the unique integration patterns revealed in myrmecophagid anteaters. Following this scenario, the different rostral integration patterns in pangolins could reflect a less pronounced midfacial growth in this clade.

## Conclusions

We showed that morphological changes in convergently-evolved lineages did not radically change the modular patterns in the skull of myrmecophagous placentals. Our data fails to detect a convergent shift in cranial modularity and integration among myrmecophagous placentals. This confirms previous results suggesting that changes in the morphospace and adaptation to functionally demanding ecologies (e.g., fossoriality) do not mandatorily impact structural integration [122]. In the case of myrmecophagous placentals, the loss of covariance generated by tooth eruption (anteaters and pangolins) did not have a noticeable effect in either modularity or integration. On the other hand, the presence or absence of a zygomatic arch module, as well as its integration values, appear to be independent of the masticatory capabilities associated with each of the species in our dataset. While the results regarding the zygomatic arch could be related to some instability of the zygomatic-pterygoid module at the evolutionary level (e.g., [31, 103, 104]), our inability to reveal covariance shifts associated with the absence of tooth development may result from two factors: (1) the fact that the current knowledge about the influence of tooth eruption on phenotypic variation during development is limited to rodent model species [92], while none exists for the sister taxa of myrmecophagous placentals; (2) being the major factor of generation of covariation in the skull [3], allometry in the anterior part of the rostrum may obscure any slight variations associated with tooth loss, when comparing toothless (anteaters and pangolins) and toothed (giant armadillo, aardvark, aardwolf) species. The latter factor additionally offers a putative explanation for the developmental affinities between the oro-nasal and the naso-palatine regions in myrmecophagid anteaters, setting them apart from pangolins and other placentals. Such unique integration patterns observed in myrmecophagid anteaters may provide an example of generation of strong trait correlation while preserving phenotypic organizational modules (canalization; [123, 124]). Under this scenario, myrmecophagid midface growth might represent an incipient process of decanalization [125, 126] resulting from strong selective pressures and extreme ecological specialization.

## Methods

### Biological sampling

Skull digitization included specimens from the collections of the Natural History Museum (BMNH) in London (United Kingdom), the Museum für Naturkunde (MfN) in Berlin (Germany), the Muséum National d’Histoire Naturelle (MNHN) in Paris (France), the Institut des Sciences de l’Evolution in Montpellier (France), the Royal Museum for Central Africa (KMMA/RMAC) in Tervuren (Belgium), the American Museum of Natural History (AMNH) in New York (United States of America), the National Museum of Natural History (USNM) in Washington DC (United States of America), and the Museum of Vertebrate Zoology (MVZ) in Berkeley (United States of America). Our dataset is composed of cranial landmarks of 466 specimens from 13 extant species of myrmecophagous placentals (Additional file 1; Additional file 3: Table S1). Our study excluded two myrmecophagous placental species—*Otocyon megalotis* (bat-eared fox) and *Manis crassicaudata* (Indian pangolin)—due to very few specimens collected.

We placed 54 three-dimensional landmarks on skulls using a Revware MicroScribe M 3D digitizer (Additional file 3: Table S2). We selected homologous anatomical landmarks for our morphologically diversified sample based on previous works [31, 97, 127, 128]. In this paper, landmark numbers are always preceded by “#” (e.g., #3 and #23 are the ventral margin of the left and right infraorbital canals). Given that modularity analyses are based on the covariation/correlation structure of the data, we opted out the placement of semilandmarks to avoid autocorrelation issues [129]. In a significant number of pangolin and anteater specimens (82% of our myrmecophagous sample) premaxillae were either absent, loosely attached, or broken, and were therefore not landmarked. Other missing landmarks were estimated via thin plate spline interpolations [76], for each species, using the ‘estimate.missing’ function in *geomorph* v.3.2.1 in R [130].

### Modularity

We used three different methods to assess patterns of modularity within each of the 13 species studied. First, we used Euclidean Distance Matrix Analysis on non-superimposed landmark configurations (EDMA; [67, 68]) as an exploratory method without a priori hypotheses to visualize the structure of the data and their raw patterns of covariation and correlation. Second, we used the maximum likelihood approach (EMMLi; [71]) and, third, the Covariance Ratio (CR; [72]), two widely recognized methods to test for a priori hypotheses of modularity.

We performed EDMA using pairwise interlandmark distances to detect modular structures while avoiding the potentially undesirable effects of Procrustes superimposition [129]. The EDMA procedure does not require landmarks to be aligned with Procrustes superimposition. This avoids the issues related to estimations of variability of the landmarks that are placed further from the centroid of shape [67]. EDMA consisted of four steps: (1) reconstructing the mean shape coordinates obtained from the average form matrix using the ‘mEDMA2’ function from Claude (2008)[76]; (2) constructing a covariance matrix based on all configurations; (3) extracting the eigenvectors with corresponding positive eigenvalues resulting from the covariance matrix; (4) scaling the eigenvectors by using the eigenvalues; (5) calculating the inter-trait Euclidean distance matrix from the scaled eigenvectors; (6) identifying trait clusters using Ward’s clustering method. For the last step, we used the Gap statistic [131] as implemented in the function ‘clusGap’ of the *cluster* v.2.1.0 R package [132]. For each species, we tested the splitting of the tree in two to ten clusters. The preferred number of clusters was selected and we then determined the cluster composition with bootstrap resampling using the ‘clusterboot’ function in the *fpc* R package [79, 133]. This function applied the Jaccard similarity coefficient [134] to compare landmark affiliation between bootstrap runs, in order to appraise the stability of each cluster. A cluster was considered unstable when the Jaccard coefficient was below the 0.70 threshold [133]. The number of clusters was reduced until all resulting units presented a Jaccard coefficient equal or superior to the defined threshold. A supplemental stability assessment was carried on using a less conservative threshold (0.60) in order to understand the effect of threshold choice in the final number of clusters. This study aims at identifying shifts in covariance patterns associated with rostrum elongation, tooth and mastication loss, and we therefore mostly focus on divisions of the anterior half of the skull. In some species (e.g., Fig. 3), a reduced number of landmarks was excluded because some linear distances presented a variance larger than their mean (see [76]). This happened when landmarks were placed exceptionally close to each other (short distances; [76]) and never involved the removal of bilateral landmarks (all anatomical regions were integrally present in the analysis). In some cases, small asymmetries might be present in the EDMA-retrieved architectures likely due to a combination of residual differences in the calculation of covariances for bilaterally symmetric landmarks. These asymmetries might have been generated by the shape perturbation model within EDMA (see [67], p. 128 “Step 2”), but further details were not investigated here. Nonetheless, results were consistent with previous hierarchical clustering based studies (e.g., [6, 31, 80]; see ‘Results’).

In order to test a priori hypotheses of modular partitioning of the skull, landmark sets were subjected to species-specific Generalized Procrustes Analysis (GPA; [135, 136]). The GPA scales to centroid size, optimally translates, and rotates the specimens using the least-squares criterion [135]. A matrix of landmark correlations (congruent coefficient) was then constructed for each species using the ‘doctorr’ function of the *paleomorph* v0.1.4 R package [137]. Multiple modular architectures were tested using the ‘EMMLi’ function in the *EMMLi* v0.0.3 R package [71]. This approach selects the best model based on maximum likelihood, comparing models with different numbers of modules and different variations within the same model related to inter- and intra-modular correlation pattern heterogeneity [71]. Additionally, EMMLi enables the analyses of architectures with unintegrated landmarks (landmarks with no affiliation to a module; NAs in Additional file 3: Table S2). EMMLi was performed twice for each species, with option *abs* set to FALSE in the second analysis in order to allow negative inter-modular correlations to be retrieved [24]. For each species, we checked for high between-module correlations that could favor the merging of two modules (as suggested in [25]). In this study, the architectures tested are referred to in roman numerals as presented in Table 2. We tested eleven different modular architectures (Table 2) varying from a fully integrated skull (one single module) to the classical therian mammal six-module architecture (Architecture IV; Fig. 4; [28]). Our a priori architectures additionally included a variant of Goswami (2006)’s therian six-module architecture with the zygomatic process landmarks added to the zygomatic-pterygoid module (Architecture V), analogues of those previously tested by Hallgrímsson et al*.* [30] (three and six modules; respectively Architecture II and VI), and a variant of the therian six-module architecture with the oro-nasal module coded as unintegrated [82] (Architecture III). To this set of hypotheses, we added a classical division of the skull in two modules (face-neurocranium; Architecture I; Fig. 4), as well as four seven-module architectures (e.g., Architecture VII to X; Fig. 5) modified according to the exploratory results from EDMA (see ‘Results’).

Sample size is known to be an important factor when measuring integration, with heterogeneous sampling having possibly spurious effects when covariance structures are compared across species (e.g., [138]). However, the maximum likelihood approach used here has shown to be robust to variation in sample sizes regarding both architecture selection and modular integration measurements [31]. Therefore, we assume integration values (ρ) to be comparable across species with different sample sizes. In order to confirm the robustness of our architecture selection and integration measurement procedures, we selected our two best sampled species (*Tamandua tetradactyla*—*n* = 74; *Phataginus tricuspis*—*n* = 72) and reported results for EMMLi with sample sizes of 30 and 15 in Additional file 3: Table S5.

In addition to the correlation matrices calculated from the Procrustes-aligned coordinates, modular architecture was tested on static allometry-corrected residuals. When a significant relationship with size was found, allometry-corrected shapes were extracted from a Procrustes ANOVA (shape ~ centroid size) performed with the ‘procD.lm’ function in the *geomorph* R package.

We additionally used the covariance ratio (CR; [72]) to test for a priori hypotheses of modular architecture using the ‘modularity.test’ function in the *geomorph* R package. For each species, we submitted all tested modular architectures to the CR test and then performed a pairwise comparison using *Z*_*CR*_ [73]. The models with the most negative *Z*_*CR*_ values represented those with the strongest modular signals for each individual species. This method enabled us to provide a more robust comparison of modularity hypotheses, considering the EMMLi trend towards the selection of the most parametrized architectures [23, 73].

## Supplementary Information

**Additional file 1:** Dataset composed of 54 three-dimensional landmarks placed on 466 skulls.

**Additional file 2: Figure S1.** Homologous anatomical landmarks used across the skull in (A) ventral, (B) dorsal, and (C) lateral views. A detailed list of landmarks, as well as their affiliation to the ten a priori architectures tested, is given in Table S2. The architecture presented here corresponds to architecture 1. **Figure S2.** Clusters retrieved with EDMA for *O. afer* (A) and *S. gigantea* (B). Colors of landmark configurations do not correspond homologous anatomic clusters (left). Gap statistics estimation (y-axis) according to the number of defined clusters (*x*-axis) for *O. afer* (A, right) and *S. gigantea* (B, right). Black arrows indicate the lowest *k* value for which the Gap statistics stabilize. **Figure S3.** Results of EDMA for all 13 myrmecophagous placentals included in this study. Spheres (left) represent the landmark configuration for each species, colored according to the recovered hierarchical structure (right). Colors do not correspond to homologous units between species or to the colors adopted in the main text figures. The species name and the number of cluster identified by the Gap statistics is given below each item. **Figure S4.** EDMA results (above) and the naso-palatine module (below) in *T. tetradactyla* (A) and *O. afer* (B). Landmark color corresponds to the therian six-module (Goswami, 2006; A) and macaque phen-gen (Hallgrímsson, 2004; B) six-module architectures to which the nasopalatine was added, resulting in architectures VII (A) and VIII (B), respectively.

**Additional file 3: Table S1.** List of myrmecophagous species included in this study. Number of specimens (*N*). **Table S2.** List of homologous anatomical landmarks used across the data set. Columns I to X show the affiliation of each landmark to the ten a priori architectures tested in this study (Table 2). **Table S3.** Number of clusters resulting from the EDMA for each myrmecophagous species and minimum Jaccard coefficient per number of modules (*k*) calculated from a 10,000 bootstrap. Number of specimens (*N*), value of Gap statistics (Gap) for the optimal number of clusters (Cluster), and final number of clusters after removing biologically meaningless cluster (Final). **Table S4.** Modular architectures of 13 myrmecophagous species. Number of specimens (*N*), most likely modular architectures recovered with EMMLi (*MLi*), covariance ratio (*CR*), within-module absolute correlations (*ρ*), correlation between oro-nasal and molar-palate modules (*ρ abs*) and mean within-module correlation (Mean *ρ*). (1) Oro-nasal/rostrum, (2) molar-palate, (3) orbit, (4) zygomatic-pterygoid, (5) vault, (6) basicranium, (7) naso-palatine. All *CR* values were significant. **Table S5.** Static allometry-corrected modular architectures of two myrmecophagous mammals with three different sample sizes. Number of specimens (*N*), most likely modular architectures recovered with EMMLi (*MLi*), covariance ratio (*CR*), within-module absolute correlations (*ρ*), and correlation between oro-nasal and molar-palate modules assuming a therian six-module (V) architecture (*ρ abs*). (1) Oro-nasal/rostrum, (2) molar-palate, (3) orbit, (4) zygomatic-pterygoid, (5) vault, (6) basicranium, (7) naso-palatine. All *CR* values were significant. **Table S6.** Static allometry-corrected (when detected) effect size (*ZCR*) for the most likely architecture retrieved by EMMLi. For each most likely architecture, the *p*-values of the modular signal strength comparison are given. Most likely modular architectures (*MLi*), effect size calculated with the compare. CR function (*ZCR*, Adams & Collyer, 2019), p-value of the comparison with non-modular hypothesis (0), *p*-value for the comparison with the remaining modular hypothesis (I-X). The lowest *ZCR* values are in bold. Significant and marginally significant p-values are in red and orange, respectively. **Table S7.** Between-modules correlations of *C. didactylus* and *M. tridactyla*. Between-modules absolute correlations (*ρ*) for the most-likely architecture in *C. didactylus* (upper triangle; values in black) and *M. tridactyla* (lower triangle; values in blue). (1) Oro-nasal/rostrum, (2) molar-palate, (3) orbit, (4) zygomatic-pterygoid, (5) vault, (6) basicranium, (7) naso-palatine. **Table S8.** Between-modules correlations of *T. mexicana* and *T. tetradactyla*. Between-modules absolute correlations (*ρ*) for the most-likely architecture in *T. mexicana* (upper triangle; values in black) and *T. tetradactyla* (lower triangle; values in blue). (1) Oro-nasal/rostrum, (2) molar-palate, (3) orbit, (4) zygomatic-pterygoid, (5) vault, (6) basicranium, (7) naso-palatine. **Table S9.** Between-modules correlations of *S. gigantea* and *S. temminckii*. Between-modules absolute correlations (*ρ*) for the most-likely architecture in *S. gigantea* (upper triangle; values in black) and *S. temminckii*. (lower triangle; values in blue). (1) Oro-nasal/rostrum, (2) molar-palate, (3) orbit, (4) zygomatic-pterygoid, (5) vault, (6) basicranium, (7) nasopalatine/ naso-frontal. **Table S10.** Between-modules correlations of *M. pentadactyla* and *M. javanica*. Between-modules absolute correlations (*ρ*) for the most-likely architecture in *M. pentadactyla* (upper triangle; values in black) and *M. javanica* (lower triangle; values in blue). (1) Oro-nasal/rostrum, (2) molar-palate, (3) orbit, (4) zygomatic-pterygoid, (5) vault, (6) basicranium, (7) nasopalatine. **Table S11.** Between-modules correlations of *P. tricuspis* and *P. tetradactyla*. Between-modules absolute correlations (*ρ*) for the most-likely architecture in *P. tricuspis* (upper triangle; values in black) and *P. tetradactyla* (lower triangle; values in blue). (1) Oro-nasal/rostrum, (2) molar-palate, (3) orbit, (4) zygomatic-pterygoid, (5) vault, (6) basicranium, (7) naso-palatine. **Table S12.** Between-modules correlations of *O. afer* and *P. maximus*. Between-modules absolute correlations (*ρ*) for the most-likely architecture in *O. afer* (upper triangle; values in black) and *P. maximus* (lower triangle; values in blue). (1) Oro-nasal/rostrum, (2) molar-palate, (3) orbit, (4) zygomatic-pterygoid, (5) vault, (6) basicranium, (7) naso-palatine. **Table S13.** Between-modules correlations of *P. cristata*. Between-modules absolute correlations (*ρ*) for the most-likely architecture, (1) Oro-nasal/rostrum, (2) molar-palate, (3) orbit, (4) zygomatic-pterygoid, (5) vault, (6) basicranium, (7) naso-palatine.

## Data Availability

The datasets used and/or analysed during the current study are available on:10.6084/m9.figshare.15802323.

## Abbreviations

EDMA: Euclidean distance matrix analysis
EMMLi: Evaluating modularity with maximum likelihood
CR: Covariance ratio
