# Supplementary material for: Flexible conservatism in the skull modularity of convergently evolved myrmecophagous placental mammals
Source: BMC Ecol Evol. 2022 Jun 30;22:87. doi: 10.1186/s12862-022-02030-9 (PMC9248141; doi:10.1186/s12862-022-02030-9)
Supplement: Supplementary file 1 — Additional file 1: Dataset composed of 54 three-dimensional landmarks placed on 466 skulls. [file 12862_2022_2030_MOESM1_ESM.pdf]

Additional file 1 - Dataset composed of 54 three-dimensional landmarks placed on 466 skulls

LM3=54
2.3362999999999 -0.0451000000001603 -2.34960000000005
1.04439999999975 -2.52670000000025 -2.52360000000007
11.0355000000001 -0.792800000000102 -0.285199999999963
10.9064000000001 -0.680100000000088 -0.21849999999995
12.1887 2.19959999999993 -1.40779999999994
15.8965000000001 1.23559999999995 0.11540000000006
17.3532942783229 1.92464058046048 -0.254565417487922
19.8519000000001 -0.0166000000000053 8.96620000000008
17.4892000000001 3.67290000000001 -1.30309999999994
24.2617000000002 3.01810000000006 0.38120000000007
25.7830000000002 2.65520000000005 -1.17039999999994
29.5921 -4.16860000000003 -0.257699999999935
32.3245 1.72480000000001 -3.87439999999995
35.1611 -0.646600000000011 -3.75939999999995
38.5099999999999 3.48319999999998 -4.25849999999995
41.2548999999999 4.98919999999997 -3.63309999999994
38.9088999999998 7.84359999999997 -5.37249999999994
43.2368999999997 8.49779999999994 -1.04509999999993
42.3250999999998 3.39669999999993 -3.95649999999995
42.6482999999998 4.91539999999996 -5.23339999999994
2.50829999999995 1.24549999999981 -2.12399999999994
0.159099999999963 2.60639999999978 -2.70579999999995
10.5748999999999 5.18879999999988 0.210100000000068
8.95399999999996 4.9672999999999 -0.00679999999994152
14.3219 5.71929999999996 -0.331899999999931
16.0408 5.33289999999998 -0.870099999999931
17.3078 7.15989999999995 9.19130000000009
24.0603000000001 6.30870000000005 -0.421199999999927
25.0094000000001 7.56610000000005 -1.81349999999993
24.7324999999999 16.1275 -0.277299999999931
29.8641999999999 11.121 -4.14589999999995
31.8727999999999 14.4355 -4.04049999999993
35.8754999999997 12.6686 -4.71539999999993
39.6956999999998 12.6251 -3.79579999999992
39.8528999999997 13.8584 -3.75079999999992
40.5679999999997 12.4251 -5.07559999999991
-0.0301000000000664 -0.212400000000177 -0.0246999999999128
-0.186300000000129 1.46689999999976 -0.486099999999936
9.96659999999994 3.23349999999986 5.64410000000006
12.5086999999999 6.05399999999992 2.61210000000006
10.6927999999999 7.54799999999992 0.46980000000008
9.91969999999997 1.71089999999986 6.10460000000005
28.1099999999999 5.06459999999996 11.1542000000001
37.7720999999998 7.07369999999995 8.6105000000001
37.8123999999997 16.9042 0.152400000000097
43.5331999999997 8.6225999999999 4.44260000000008
40.7761999999997 13.2376 -2.52219999999992
-0.0301000000000664 -0.212400000000177 -0.0246999999999128
0.356499999999986 -2.02360000000024 -1.10669999999995

10.9385000000001 -0.626500000000127 5.50760000000006
12.9927000000001 -0.90840000000009 2.66190000000007
12.6169000000001 -2.82240000000008 0.786600000000043
41.9844999999999 0.0313999999999351 -0.0355999999999289
42.7513999999998 4.13889999999994 -2.70559999999993
ID=CYCdidMALBRAPV*BMNH261210

LM3=54
1.45349999999946 0.160399999999992 -0.989599999999854
1.76219999999949 -2.22100000000016 -2.1546999999999
17.4869999999995 0.318699999999969 1.50940000000005
15.7831703312398 -0.491529547838089 1.86274389301943
13.1271999999995 2.5147 0.0646000000000324
17.3612999999995 0.477499999999951 1.71370000000002
19.7736113140103 1.35602569906972 1.29016151570507
21.6275999999995 -0.683200000000042 10.4575
19.7187999999994 3.50919999999994 -0.0784999999999909
26.7679999999995 3.79759999999995 1.09479999999999
28.6361999999996 3.40419999999995 -1.06230000000002
31.5644999999997 -4.13630000000007 0.0216999999999583
35.4880999999998 1.30039999999993 -4.32880000000007
39.3206999999997 -0.371800000000073 -3.78200000000007
43.2393999999997 3.4902999999999 -4.28670000000009
45.5273999999997 4.7441999999999 -3.88420000000012
43.5519999999996 8.2907999999999 -5.51550000000009
48.2926999999996 9.4567999999999 -1.45320000000012
47.4764999999998 3.39929999999989 -3.67260000000012
46.7866999999997 5.7440999999999 -5.27730000000011
1.41719999999932 1.17889999999993 -0.85419999999991
1.04559999999932 2.29969999999994 -2.13439999999991
11.5610999999994 5.17439999999997 1.54400000000005
12.1415999999994 6.68139999999996 2.06290000000005
16.2533999999994 5.60959999999996 1.75440000000003
18.6572999999994 4.93049999999995 1.12460000000003
19.5629999999993 7.84929999999995 10.1021
26.7487999999994 5.95249999999993 0.814299999999992
27.8071999999993 7.52959999999991 -0.894300000000017
27.1965999999992 15.3958999999999 -0.122199999999999
33.4000999999994 11.3441999999999 -4.14280000000003
35.8248999999992 13.6990999999999 -4.09560000000004
40.8356999999994 12.7136999999999 -4.63370000000007
44.7622999999995 12.7289999999999 -3.78440000000009
45.0181999999994 13.9341999999999 -3.8407000000001
45.7863999999995 12.2082999999999 -5.2068000000001
-0.120600000000699 0.0283999999994 -0.275999999999916
-0.774029190268652 1.43189778805618 -0.99787564772785
11.6113999999994 4.18109999999997 7.51300000000004
13.3803999999994 6.36469999999997 3.99730000000004
13.7966999999994 8.46809999999997 2.17120000000005
11.0434999999994 1.58109999999997 8.02360000000004
31.1946999999994 4.86669999999991 12.0594
40.9909999999995 6.61529999999989 9.59939999999993
40.9630999999993 15.3558999999999 0.153099999999906
48.7088999999996 7.79309999999988 4.56309999999988

45.7694999999994 12.0301999999999 -3.02420000000011
-0.120600000000699 0.0283999999994 -0.275999999999916
-0.24370000000067 -2.65870000000006 -0.523899999999907
12.4541999999995 -0.761300000000034 7.72610000000004
14.6387999999996 -2.09690000000001 4.44150000000003
14.7562999999996 -3.34240000000005 3.07240000000003
45.2496999999997 -0.609900000000098 0.270199999999892
47.5115999999998 3.04459999999989 -2.55920000000012
ID=CYCdidFEMBRAPV*BMNH261212

LM3=54
2.6090000000011 0.271200000000153 -0.678400000000039
0.458899999999469 -1.61730000000019 -1.12549999999995
12.2358000000013 -1.2774999999998 2.16669999999994
12.2903000000003 -1.07069999999996 2.34729999999999
11.2841000000002 2.22790000000001 0.85699999999995
16.4472000000002 1.09019999999998 1.79709999999997
18.0681941067896 1.84932626136038 1.32074748605657
20.1692000000009 -0.52109999999985 9.83369999999997
17.3558000000005 3.22060000000012 0.146899999999959
25.1919000000002 3.9741 1.56350000000001
26.8757000000005 2.75160000000005 -0.557499999999992
29.3261000000004 -4.14639999999994 0.734699999999996
32.5159000000002 1.70480000000001 -3.491
35.9038000000001 -0.229900000000025 -3.6108
39.5643000000002 3.0064 -3.5144
41.6808000000002 4.35399999999998 -3.0467
40.2039000000002 7.78829999999997 -4.49099999999999
44.7224000000003 8.6667 -0.161999999999987
43.5775000000002 2.76229999999999 -2.4681
43.8342000000002 4.86939999999997 -4.1104
1.00760000000101 0.829600000000136 -0.191700000000036
-0.72679999999935 2.45720000000006 -1.44820000000001
10.7055000000006 5.19060000000005 1.61479999999996
11.0804000000005 6.03850000000005 2.30489999999997
14.9647000000003 5.48150000000004 2.33779999999996
16.5899617993288 5.36178693489558 1.58076869670114
18.2456000000007 7.60790000000009 10.5128
24.5766000000003 6.64050000000006 1.29869999999998
25.4959000000002 7.47140000000004 -0.587600000000018
25.0724000000003 15.4669 0.117899999999986
30.6808000000002 11.076 -3.37650000000001
32.9344000000003 13.5168 -3.74290000000001
37.8463000000002 12.8476 -3.0824
40.2046000000003 11.6896 -2.65419999999998
40.8335000000003 13.4329 -2.8978
41.8578000000002 12.3708999999999 -3.87339999999998
0.0063000000044662 -0.357199999999959 -0.00829999999999076
-0.250599999999485 1.27200000000005 -0.734800000000008
10.6224000000009 4.29150000000011 6.80779999999996
12.9531000000005 5.85860000000006 3.54199999999996
10.9673000000005 7.45620000000006 2.73769999999995
10.485100000001 1.89820000000014 7.44829999999994
28.9757000000007 5.19460000000009 11.9344

37.5054000000006 7.28730000000007 9.17440000000001
38.5335000000003 16.7032 -0.0248000000000126
45.1644000000005 8.92810000000001 3.99680000000001
42.0389000000003 13.515 -2.42009999999999
0.00630000000044662 -0.357199999999959 -0.00829999999999076
0.0756999999998666 -1.79300000000007 -0.941499999999976
11.3855000000011 -0.0400999999998363 6.64239999999996
14.9873000000007 -0.24039999999988 3.17419999999998
13.438000000001 -2.71969999999983 2.268
41.2829000000004 -0.0717999999999856 -1.272
44.1790000000003 4.39889999999998 -2.61979999999998
ID=CYCdidMALBRAPV*BMNH26129*

LM3=54
-0.1106 -0.1589 0.1628
-1.0399 -2.4682 -0.3804
9.106 -1.4834 2.3182
8.3726 -1.2966 2.1433
10.119 2.3398 1.1464
14.3354 0.9801 1.991
15.7902 1.6919 1.5243
20.7155 0.3508 9.7493
16.7984 3.9572 0.459
21.9527 3.8249 1.3009
23.7628 3.1438 0.4356
30.0879 -3.9386 -1.9629
30.6352 1.6482 -3.6037
33.3461 -0.6373 -4.2086
39.0041 2.5544 -5.9452
40.8578 5.0372 -4.3983
38.8931 8.2388 -5.8049
43.664 8.9473 -1.4415
42.8106 3.9752 -4.1131
42.0732 5.3107 -5.8251
-0.1923 0.5553 0.4061
-2.0717 2.1867 -0.2573
8.0517 5.4492 2.2827
7.5992 5.5574 2.5828
14.02 5.5523 2.5739
15.242 4.5111 1.4829
19.4756 8.6802 10.0663
21.4793 5.4575 1.4573
22.6503 6.6919 0.4933
24.3707 15.0625 -1.3997
28.6509 10.5459 -3.0588
30.0234 13.2022 -3.9387
36.8297 13.7074 -5.76
39.9393 12.024 -4.0898
40.178 13.425 -3.8499
41.0633 12.3261 -5.8143
-0.1511 -0.2526 0.129
-1.1875 1.2304 -0.74
8.9764 5.2474 5.7871
10.621 7.556 2.8598

8.6371 8.7407 2.7844
9.2227 1.953 6.8866
28.0366 5.4891 11.8094
38.4575 7.2363 9.0712
40.172 17.0779 0.1453
45.9668 9.0307 4.1149
43.4152 13.0629 -2.6277
-0.1511 -0.2526 0.129
-0.2471 -1.8316 -0.7017
10.6854 -1.1575 5.7529
12.0121 -2.2328 3.0713
11.014 -3.6461 2.1766
44.2997 0.1553 0.1226
44.9707 4.7682 -2.506
ID=CYCdidMALBOLRO*BMNH261522

LM3=54
2.40900000000035 0.369100000000133 -1.15610000000005
1.69589999999996 -1.69100000000024 -2.39639999999998
12.3815000000011 -1.18599999999975 2.38849999999992
14.1440000000001 -1.70089999999997 2.09199999999997
12.7351999999999 2.26210000000004 1.25680000000001
16.717 0.315900000000069 1.92489999999998
19.5364000000001 1.81150000000014 1.25059999999997
21.7645000000004 -1.07939999999988 11.8087
19.0514999999993 3.23909999999994 0.519900000000045
25.4382999999997 3.39820000000001 1.56570000000001
26.6658 2.56490000000003 -0.515100000000004
30.9030000000005 -5.3120999999999 -0.113100000000005
33.5674000000002 1.58230000000009 -4.403
37.5090000000002 -0.623699999999914 -3.81339999999999
42.3152 2.54900000000004 -4.55069999999999
45.7198 4.15600000000008 -3.8687
42.9618999999999 7.98400000000001 -5.81989999999999
47.6288 8.69050000000005 -1.66360000000001
46.4650000000002 3.40750000000006 -4.38560000000001
46.639 4.53350000000005 -5.3698
2.18680000000026 0.898500000000083 -1.33930000000006
0.852600000000163 2.64440000000003 -2.23110000000006
11.107 5.14840000000006 2.14529999999999
12.3379999999998 6.53540000000003 2.70209999999999
15.2040999999996 5.36590000000001 2.40180000000004
18.3864873361405 4.7411984760534 1.95628851689123
19.8375999999999 8.44500000000006 11.9888
24.5060999999996 6.11989999999999 1.82850000000003
25.8077999999996 7.76839999999999 -0.497499999999973
26.7778999999995 15.6084 -0.246899999999969
32.5339999999996 10.6747 -4.11669999999997
34.4283999999997 13.8463 -3.81949999999997
40.5034999999997 13.0721 -4.70519999999998
43.9786999999998 12.4597 -4.32899999999999
43.8903999999999 13.9823 -3.94389999999999
44.8886999999998 12.442 -5.24279999999997
0.070000000002167 -0.0125999999999972 0.15549999999996

-0.970299999999845 1.52090000000001 -1.07240000000004
9.70120000000022 4.00060000000009 7.84159999999998
12.9660999999998 6.70220000000002 3.9005
13.3101999999997 8.50640000000002 2.9058
9.42280000000042 1.49420000000012 8.29949999999993
31.6999000000001 5.61250000000008 12.9351
39.2202000000001 6.94130000000008 10.0273
42.2680999999998 16.4277 0.176700000000004
47.2574000000001 8.67930000000005 4.2928
45.4853999999999 13.7649 -2.57189999999999
0.0700000000002167 -0.0125999999999972 0.155499999999996
-0.563099999999824 -1.65270000000003 -1.59160000000005
10.5001000000006 -0.946899999999845 7.51539999999993
14.2345000000002 -2.0766999999999 3.74309999999997
13.8612000000003 -4.53369999999988 3.43950000000001
46.1758000000003 -0.113299999999928 -0.0511000000000118
47.3828000000002 4.02920000000005 -2.5371
ID=CYCdidFEMPERUA*BMNH721102

LM3=54
2.28499999999891 0.09849999999986 -2.21220000000006
0.616299999997589 -2.09040000000032 -2.89849999999987
11.8661999999985 -0.667400000000126 -0.265699999999879
13.2915999999984 -1.40160000000023 -0.367999999999981
12.4067999999992 2.75370000000005 -1.27739999999997
16.0216999999991 0.125999999999926 -0.469500000000019
18.7604999999989 1.78599999999992 -1.02599999999997
20.112199999999 0.269799999999892 9.56310000000004
19.2215999999993 4.00839999999992 -1.36629999999999
24.9504999999994 3.50439999999993 -0.299699999999942
27.4294999999995 2.90569999999999 -2.62369999999997
28.7792999999994 -5.44980000000002 -0.948599999999991
32.2995999999993 1.11019999999996 -5.35549999999995
36.1965999999993 -0.95650000000004 -5.13009999999993
40.4347999999992 2.78929999999994 -4.41879999999991
42.7199999999992 4.75769999999994 -3.7187999999999
41.1116999999992 8.18759999999997 -5.56229999999992
45.6252999999992 9.14959999999994 -1.4254999999999
44.8029999999992 4.25199999999993 -3.42849999999992
44.6926999999992 5.19129999999995 -4.5949999999999
2.21339999999887 0.932199999999839 -2.18660000000004
-0.0556000000013602 2.3965999999998 -2.88150000000001
10.8159999999992 5.43369999999997 -0.485100000000017
11.4329999999993 6.1754 -0.392200000000019
15.2257999999993 5.7314 -0.43800000000002
18.6173999999993 5.28969999999998 -1.17490000000001
18.3136999999992 9.11739999999994 9.40560000000001
24.6918999999995 5.82239999999996 -0.3199
26.5641999999994 7.42549999999997 -2.90149999999999
23.9404999999992 15.8106 -1.22899999999998
30.5354999999993 10.9383 -5.57959999999996
32.6931999999992 14.1785 -5.36669999999994
38.0505999999992 12.0595 -4.00399999999992
41.1561999999991 12.8856999999999 -3.45099999999991

41.9946999999991 14.2745999999999 -3.16849999999991
42.8425999999992 12.5323 -4.2892999999999
0.246799999998289 0.199999999999745 0.418700000000028
-1.28020000000161 1.99199999999976 -1.04419999999998
9.30579999999904 5.22319999999993 5.3099
12.3804999999993 7.65909999999999 1.25569999999998
12.1129999999993 8.21049999999999 0.324199999999974
9.23969999999886 2.76759999999988 6.25050000000002
28.9085999999991 6.05889999999991 11.8956
36.5465999999992 7.55519999999992 10.0747000000001
39.0232999999991 17.6014 0.15140000000008
44.4936999999991 9.97769999999993 5.25140000000011
42.5998999999991 14.1335999999999 -2.41759999999991
0.246799999998289 0.199999999999745 0.418700000000028
-0.577400000002193 -1.88240000000031 -0.884799999999918
10.0931999999987 0.172599999999853 5.10510000000006
14.2194999999988 -1.22210000000013 1.82740000000005
13.474799999999 -3.13890000000011 0.262500000000044
43.3589999999992 0.827399999999926 0.222300000000087
44.9142999999993 5.02809999999993 -2.31579999999991
ID=CYCdidFEMPERUA*BMNH721103

LM3=54
0.443400000001298 0.108400000000458 -0.840499999999927
1.23400000000151 -1.34169999999939 -1.60019999999977
12.6508000000011 -1.22059999999962 1.59120000000004
11.9054000000009 -0.946899999999965 1.76330000000002
10.9535000000009 2.56900000000029 0.910600000000039
15.9346000000008 0.403600000000305 1.83040000000001
17.3914000000007 1.73660000000028 1.37739999999999
20.2471000000008 -0.435299999999661 11.5916
19.9314000000007 3.81170000000026 0.129299999999987
24.4125000000006 2.85340000000022 1.86289999999996
26.0598000000006 2.60580000000022 -0.0279000000000326
28.0978000000008 -4.74999999999971 1.56459999999998
32.4100000000005 1.28700000000019 -3.32490000000002
34.2205000000005 -0.413999999999796 -3.28460000000001
38.6670000000004 3.01990000000017 -3.12529999999997
41.3915000000005 4.06390000000019 -2.34249999999996
39.3545000000003 7.42860000000014 -4.24999999999995
43.8934000000004 8.55710000000019 -0.669999999999954
43.1163000000006 3.53720000000021 -3.15709999999997
42.6992000000004 4.31180000000018 -4.39689999999994
0.0103000000013314 0.644800000000512 -0.856799999999928
0.532800000001346 2.14200000000051 -1.28249999999993
11.105600000001 5.94160000000036 1.94680000000006
10.3283000000009 6.27060000000034 2.03130000000005
14.0082000000008 5.0190000000003 2.27020000000002
16.0926000000007 4.23260000000028 1.546
18.5063000000008 8.52800000000031 11.3644
23.3868000000006 5.71330000000021 1.87749999999997
24.7893000000006 7.14910000000019 -0.258100000000025
24.0704000000006 14.8550000000002 1.28340000000002
30.5456000000004 10.4155000000001 -2.96939999999999

31.9758000000003 13.1220000000001 -3.30779999999998
36.7905000000002 11.6590000000001 -3.11729999999995
39.8763000000002 11.9062000000001 -1.93309999999995
40.6693000000003 12.8984000000001 -2.96009999999994
41.3391000000003 11.5652000000001 -4.32869999999993
-0.0280999999985408 -0.181599999999453 0.0637000000000805
-0.698599999998519 1.5415000000055 -0.773999999999916
9.0862000000011 4.80020000000041 6.65010000000009
12.0883000000009 6.62370000000033 2.94940000000005
11.110400000001 7.82330000000035 1.81460000000005
10.0192000000012 2.29170000000043 8.01160000000008
28.4580000000007 5.03650000000028 13.601
38.2553000000005 7.21360000000022 10.7959
40.5708000000003 16.2671000000001 -0.193699999999947
45.1792000000005 8.96310000000022 5.01680000000006
42.0749924050057 12.7013359978377 -1.87246325335601
-0.0280999999985408 -0.181599999999453 0.0637000000000805
0.0434000000014552 -1.76489999999945 -0.628899999999905
10.0542000000012 -0.669199999999577 6.66050000000007
13.619000000001 -1.17939999999963 3.22110000000003
13.797300000001 -3.39709999999961 1.82440000000004
43.1479000000007 0.0867000000002416 0.209500000000026
43.6586000000005 4.4857000000002 -1.20949999999996
ID=CYCdidFEMPANCHIBMNH312610

LM3=54
1.74669999999945 0.25920000000006 -1.98130000000003
1.11099999999969 -2.08329999999974 -2.38340000000002
12.7594999999992 -1.03690000000002 0.8019
12.8171999999987 -1.18450000000017 0.999300000000056
12.1159999999994 2.58440000000005 -0.320899999999979
15.7018999999994 0.593399999999973 0.631499999999982
17.6778556222486 1.56258209274818 0.612035373905608
17.8074999999996 -0.0766999999999596 9.97969999999999
19.5801999999994 4.451 -0.518000000000013
24.2656000000002 2.79620000000011 1.28689999999994
25.6451999999993 2.42389999999997 -0.704499999999984
28.7365000000001 -5.03349999999985 0.0586999999999646
33.2526999999996 1.77180000000003 -3.9191
35.7900999999998 0.096500000000055 -4.18320000000002
40.2983 2.94780000000005 -3.60350000000004
43.2139999999999 4.49400000000006 -2.34560000000003
40.4181999999999 8.57940000000002 -4.51740000000003
45.6574 8.99330000000002 -0.773000000000052
43.6764000000001 3.58680000000007 -3.21250000000005
44.3031 5.28610000000006 -4.50500000000004
1.71339999999944 0.905200000000027 -1.75279999999998
0.927099999999483 2.93830000000006 -2.09769999999998
10.9901999999993 5.71509999999999 0.900100000000013
11.0344999999993 6.33059999999999 1.21150000000002
14.1118999999994 5.31059999999999 1.0673
16.6807124844303 5.31451066001202 0.841794216742793
18.9664999999995 8.9035 10.1938
23.4002999999996 6.41349999999999 1.27499999999999

23.8276999999994 7.46079999999998 -0.388699999999995
24.6451999999994 15.9518 0.849099999999996
31.3169999999995 11.7448 -3.3441
33.0470999999995 14.191 -3.83480000000002
37.8463999999998 12.5357 -3.49910000000003
41.3425999999999 14.0419 -2.17040000000003
41.5002999999998 14.3295 -2.87060000000003
42.2302 12.748 -4.17530000000004
0.0761999999995653 0.0270000000001058 0.129000000000027
-0.396700000000478 1.9174000000001 -0.211199999999976
9.39639999999934 5.37839999999999 6.05360000000001
12.6679999999994 6.56159999999998 2.23030000000001
11.3182999999994 8.2554 1.33080000000001
10.0258999999994 2.21470000000003 7.40290000000001
28.0286999999996 5.70990000000003 12.3394
38.5801999999998 7.64440000000003 9.62439999999997
41.1795999999998 17.2263 0.0881999999999637
45.7832 9.45900000000004 4.00749999999996
43.3450999999999 13.4379 -2.77510000000003
0.0761999999995653 0.0270000000001058 0.129000000000027
-0.197400000000295 -1.8529999999998 -0.733399999999973
10.4862999999995 -1.01329999999994 6.17370000000001
13.5980999999995 -1.51989999999996 2.37860000000001
14.0753 -3.3309999999998 0.810499999999975
43.6928000000001 -0.145799999999892 0.118199999999953
45.3204000000001 5.03220000000007 -2.49370000000004
ID=CYCdidFEMPANCHIBMNH333100

LM3=54
2.64850000000073 0.120400000000091 -0.778099999999835
1.30210000000151 -2.18510000000028 -1.78900000000007
12.6777 -1.52630000000016 1.85050000000024
11.8832000000004 -0.619600000000104 1.76160000000012
7.56599999999985 1.37999999999984 0.222100000000144
16.8428 1.36519999999987 1.76550000000013
19.1489000000002 2.26059999999991 1.1094000000001
20.8811999999999 -0.436500000000133 10.2808000000001
20.6857000000004 4.00960000000002 0.112100000000038
24.5387999999999 3.67529999999993 1.85800000000009
26.7256999999995 3.34089999999987 -0.094299999999903
30.2282999999998 -4.58000000000013 -0.89299999999988
32.8612999999998 1.83799999999987 -3.61969999999995
35.6669 0.204099999999884 -4.12279999999996
40.1494000000002 3.57949999999992 -4.75480000000001
42.9379000000003 4.59039999999995 -3.96110000000001
41.6281000000003 8.26299999999996 -6.02390000000003
45.8954000000003 8.79239999999992 -1.69360000000002
44.6688000000002 3.43429999999992 -3.9179
44.6947000000003 4.49609999999993 -5.25810000000002
2.19040000000077 0.8988 -0.417699999999855
0.361000000000929 2.48599999999999 -1.49709999999987
11.5367000000001 5.63189999999993 1.70660000000013
9.79770000000008 5.73129999999994 2.06470000000011
15.2850000000001 4.95039999999995 1.66120000000008

18.1526000000002 4.95349999999997 1.08610000000007
19.4550000000001 7.82099999999992 10.9539000000001
24.3024999999999 6.16189999999992 1.66100000000007
25.5128999999998 7.33089999999991 -0.615699999999929
26.1567000000001 15.6924999999999 -0.260099999999953
31.2154 10.2893999999999 -3.45969999999997
33.6455000000001 13.3494999999999 -3.78459999999999
38.3073000000002 11.9029999999999 -4.28430000000002
42.1696000000002 12.7711999999999 -3.20980000000001
41.8731000000002 13.7608999999999 -3.30309999999999
43.0449000000003 12.2832999999999 -4.86330000000001
-0.139899999998918 -0.119200000000003 0.157400000000133
-0.505599999998952 1.53090000000002 -0.77179999999986
10.3976000000002 4.82339999999995 6.68460000000016
12.9886000000001 6.49709999999994 4.15690000000011
10.6153000000002 7.50709999999996 2.33340000000011
9.29200000000021 1.66419999999992 7.40380000000016
30.7373 5.92809999999988 12.6992000000001
39.7151 7.41399999999989 9.04530000000006
41.0999000000002 16.2380999999999 -0.176899999999987
46.9653415524819 8.6501441815812 2.94165519782493
43.6186000000002 13.1754999999999 -2.891
-0.139899999998918 -0.119200000000003 0.157400000000133
0.0621000000013177 -1.5759 -0.627899999999877
11.1115000000002 -0.507400000000101 6.32420000000018
15.2432000000001 -0.900300000000119 3.79140000000015
13.2769 -2.50320000000009 2.61710000000013
43.4202000000002 -0.535300000000085 -0.111399999999976
45.5866000000002 4.09359999999992 -2.9304
ID=CYCdidUNKTRITRIBMNH55618*

LM3=54
2.13500000000113 0.449600000000419 -1.60830000000005
1.11970000000109 -2.3608999999995 -3.1928000000002
13.091800000001 -1.14549999999962 0.726899999999964
13.6610000000009 -1.21919999999963 0.250899999999948
13.3218000000009 3.16220000000043 -0.555700000000077
17.7027000000008 0.506300000000348 0.475199999999945
20.1374000000007 2.54100000000031 -0.182200000000062
21.4426000000008 -0.811999999999634 9.63729999999994
19.4108000000006 4.01850000000029 -0.753100000000048
25.9341000000005 3.85510000000026 0.807699999999949
28.4289000000004 4.94840000000025 -2.04580000000005
30.3533000000004 -4.30169999999973 0.0454999999999615
34.5436000000001 3.48530000000017 -4.93930000000005
37.0555999999999 1.42390000000016 -4.72090000000005
41.2664999999997 3.65690000000012 -4.03720000000006
43.2579999999996 5.5076000000001 -3.55480000000004
41.4917999999995 8.73870000000006 -5.37500000000004
46.4078999999996 9.34040000000008 -1.23310000000004
45.0629999999996 3.84140000000011 -3.00060000000004
45.1552999999996 5.7264000000001 -4.98830000000004
1.55330000000116 2.09880000000049 -2.21680000000009
-1.10879999999878 3.98120000000051 -2.95890000000011

10.590100000001 7.6657000000004 0.281099999999933
10.7213000000009 8.1931000000004 -0.0303000000000877
15.1804000000008 7.32110000000035 0.0217999999999243
17.9545000000007 6.51530000000031 -0.436900000000075
19.9173000000007 9.01340000000035 9.94699999999993
24.7354000000004 7.16200000000026 0.725999999999948
27.6808000000003 8.21230000000024 -2.31100000000004
26.6529000000003 17.2765000000002 -0.584300000000078
33.0417999999999 11.6892000000002 -5.00940000000006
34.7074999999999 14.5007000000001 -4.50140000000006
39.5727999999995 12.6532000000001 -5.43300000000004
42.5949999999995 12.6877000000001 -3.57180000000004
43.2614999999995 14.1490000000001 -3.65930000000004
44.2459999999995 12.8379000000001 -5.01550000000005
-0.122599999998729 -0.321699999999474 -0.0601000000000708
-0.953399999998726 1.64930000000052 -1.26200000000009
10.0960385671934 4.7823190957042 5.84344196653466
14.1405000000008 8.19890000000038 1.86319999999993
11.8376000000009 11.0773000000004 0.304899999999934
11.0518191444829 1.9412352543379 7.63365466677642
32.3323000000004 5.3685000000003 11.9893
39.7904 6.74480000000023 10.036
42.8972999999996 15.7796000000001 0.0492999999999521
46.6070999999997 7.94980000000016 6.18579999999994
45.3865999999995 12.7177000000001 -2.39080000000004
-0.122599999998729 -0.321699999999474 -0.0601000000000708
-0.511699999998727 -2.16519999999945 -1.46320000000012
12.493800000001 -0.852399999999601 6.34759999999998
14.5595000000009 -2.14229999999961 1.88009999999994
15.442700000001 -4.38119999999961 1.08049999999995
45.7857999999998 0.0527000000001669 -0.179400000000026
46.9389999999996 4.17590000000011 -1.91980000000004
ID=CYCdidMALBRAPARBMNH811184

LM3=54
2.35349999999809 0.125799999999578 -1.58969999999981
1.09609999999784 -1.79160000000057 -2.49489999999986
12.3510999999985 -1.49040000000029 0.899200000000166
12.2809999999985 -0.842600000000296 0.645500000000148
12.3646999999986 2.65179999999972 -0.783599999999848
16.5730999999987 1.35539999999973 0.69690000000013
18.0133999999987 2.24859999999973 0.382700000000111
18.3560999999984 0.336499999999662 9.45820000000015
17.8495999999987 3.94339999999973 -0.547899999999903
23.4406999999989 3.93409999999972 1.41040000000006
25.1912999999988 2.9401999999997 -0.978999999999953
27.7535999999987 -2.93940000000037 0.0431000000000306
31.1403999999989 3.77249999999966 -3.49140000000002
34.3851999999989 1.12759999999964 -4.20270000000005
42.0575999999988 5.4991999999996 -3.14620000000008
43.0143999999988 4.32379999999957 -2.84110000000009
39.8811999999987 9.15299999999959 -5.5020000000001
44.7290999999985 10.7450999999995 -0.552500000000071
42.5629999999987 3.88019999999953 -3.2029000000001

42.8059999999987 5.90189999999955 -4.63280000000009
2.323599999998 1.56939999999968 -1.60149999999982
0.398999999999789 2.87409999999962 -2.46769999999982
10.6665999999984 7.00739999999973 0.81990000000018
11.1836999999985 7.17669999999976 0.798500000000171
15.5191999999986 6.72979999999975 0.558300000000138
17.7637999999987 6.17949999999974 0.28410000000012
17.2721999999985 8.33289999999971 9.26160000000019
23.3900999999988 7.57429999999973 0.282400000000077
24.7271999999988 8.68079999999971 -1.46029999999995
23.8149999999986 16.7667999999997 -0.884999999999944
29.5639999999988 11.4219999999997 -4.27320000000002
31.5879999999987 14.8290999999997 -4.59220000000003
37.3214999999987 14.7443999999996 -3.62790000000008
40.3863999999986 13.3668999999995 -3.57240000000008
40.0998999999985 15.1797999999995 -3.14780000000006
41.2977999999986 14.0615999999995 -4.81030000000008
-0.0722000000021384 0.11019999999965 0.0736000000001888
-0.961500000002207 1.83499999999963 -1.30599999999982
9.61339999999836 5.33629999999973 5.94690000000021
12.0775999999985 6.74489999999975 2.41040000000018
10.4719999999985 9.23999999999973 1.26270000000017
9.22339999999835 2.3916999999997 6.11270000000021
28.9894999999984 6.09689999999961 11.4690000000001
35.5277999999984 7.56899999999954 9.93540000000007
37.6142999999984 16.9227999999995 -0.142700000000042
43.0044999999984 8.80919999999948 5.38969999999997
40.9043999999984 13.9736999999995 -2.78350000000008
-0.0722000000021384 0.11019999999965 0.0736000000001888
-0.317200000002196 -1.53940000000042 -1.05479999999982
10.9117999999984 -0.840600000000284 5.64910000000019
13.1617999999985 -0.817000000000295 2.73910000000016
12.8748999999986 -3.35590000000028 1.79630000000014
40.5565999999986 -0.0198000000004528 0.106499999999959
42.5668999999987 3.63809999999956 -2.74800000000007
ID=CYCdidFEMBRAPARBMNH811187

LM3=54
1.7388 0.1891 -1.7633
0.6208 -1.962 -2.7993
12.1942 -1.5815 0.7719
12.6688 -0.9947 0.2995
13.3246 3.1148 -0.641
16.3679 1.3628 0.5245
18.5653 2.3353 0.2183
21.2461 -0.7418 9.5244
19.3519 4.2387 -0.8766
24.5789 3.9332 0.8426
26.1475 2.5889 -1.1914
28.4767 -5.0251 0.0351
32.6775 1.5783 -3.9202
35.5434 0.0129 -3.4133
39.8352 3.0012 -3.8295
43.0725 4.7346 -3.332

40.4727 8.2914 -4.805
45.4317 9.0918 -0.1196
44.4557 3.2008 -2.4859
44.2202 5.1623 -3.9909
1.7542 0.6677 -1.7938
-0.2163 2.2547 -2.6397
10.7973 6.1978 0.7589
10.2624 6.3287 0.8733
15.2479 5.1821 1.0424
17.0866 4.6332 0.5287
18.9759 8.0774 9.7864
23.8053 5.4918 0.9712
25.2177 7.6102 -0.7212
24.4636 16.0611 0.2937
30.8342 11.3735 -3.9485
32.8221 13.6279 -3.71
38.1283 12.5556 -3.718
42.0084 12.7104 -2.5485
42.2507 14.4623 -2.4792
43.6956 12.9404 -3.9824
0.418 -0.3862 0.1766
-1.0328 1.0545 -0.9465
10.0555 4.382 5.3861
12.9909 6.8575 1.9948
11.7843 9.1157 1.7576
8.8505 1.5029 5.8696
29.9604 5.359 12.1518
36.5406 6.7617 10.3813
40.0411 16.6377 0.0208
44.7142 8.2518 5.9794
43.6083 14.1275 -2.5639
0.418 -0.3862 0.1766
-0.0889 -1.9026 -0.6496
10.9119 -0.5531 5.1131
14.6025 -1.0191 2.2788
13.1622 -3.9895 0.7907
44.0556 -0.0933 -0.0139
45.0255 3.4296 -2.5371
ID=CYCdidFEMBGUDEMBMNH112910

LM3=54
-0.0398999999995945 -0.262799999999907 -0.315500000000261
-0.724399999999718 -2.40959999999988 -0.499200000000218
9.73240000000036 -0.821299999999844 2.20549999999984
9.32570000000037 -0.808099999999847 2.28369999999985
8.90990000000037 2.42300000000015 1.20959999999989
13.7929913955592 0.818387901821492 2.58959708201306
15.7041905902032 1.82745818677427 2.06412028666492
17.9449000000003 -0.421599999999872 11.1049999999999
16.8635000000003 3.7620000000001 0.780599999999871
21.5303000000002 3.54140000000008 1.88529999999989
22.4660000000002 2.38350000000009 0.505599999999882
25.7301000000003 -4.73539999999991 0.682999999999854
28.8228000000002 2.35570000000005 -3.11670000000013

31.7037000000001 -0.0443999999999652 -3.42620000000013
35.3028 2.71449999999998 -4.73490000000012
39.2199999999999 4.20189999999996 -3.44360000000012
36.3382999999999 7.68609999999995 -5.80760000000013
41.9029999999999 9.00179999999995 -1.85550000000012
39.7208 3.87919999999999 -3.54870000000013
39.8563999999999 4.20839999999996 -5.05140000000012
-0.0288999999995406 -0.145099999999821 0.256399999999762
-1.83649999999952 1.71030000000016 -0.594100000000219
7.05905216265243 4.58482639618909 2.01721310472983
8.86000000000034 5.12210000000014 1.85349999999986
12.7580126738992 5.49384128068257 2.08892593380464
14.743419329877 5.10050989756294 1.77698861083481
15.5916000000002 9.06580000000011 10.4888999999999
21.0932000000003 6.6380000000001 1.4961999999999
21.9343000000002 7.07120000000008 -0.233300000000104
21.3995000000002 15.077 -0.674200000000117
27.1836000000001 10.1866 -3.60820000000011
29.0773000000001 12.6836 -4.22770000000012
33.5619 11.8253999999999 -4.27930000000012
37.4759999999999 12.0238999999999 -3.7110000000001
37.5871999999999 13.0322999999999 -3.86980000000011
38.1493999999999 12.0316999999999 -5.22090000000012
-1.94738467232199 -0.19588704585514 1.63784482176597
-2.64529999999954 1.28890000000018 0.880099999999744
7.60940000000038 5.40270000000017 7.42139999999984
10.4788000000003 6.53930000000014 3.60229999999986
7.20040000000034 4.99460000000013 2.05209999999986
7.9127000000004 2.07860000000017 9.01769999999983
26.1436000000002 5.93260000000008 13.5206999999999
35.1977000000001 7.50930000000003 10.3624999999999
36.811 16.1499999999999 -0.0396000000001266
41.7758 8.71479999999997 4.11789999999987
39.7247999999999 11.6725999999999 -3.42270000000012
-1.94738467232199 -0.19588704585514 1.63784482176597
-1.47289999999952 -1.8057999999998 1.04529999999971
9.08060000000038 -1.03409999999985 7.63029999999986
12.5955000000004 -0.916999999999842 4.09089999999988
11.0969000000003 -2.79499999999987 2.64579999999985
39.7725 -0.24179999999999 -0.0699000000001298
40.3659 3.96889999999996 -2.96390000000012
ID=CYCtriFEMCOLCWABMNH971738

LM3=54
2.29829999999906 0.529800000000071 -1.78339999999999
1.41449999999895 -1.86240000000008 -2.65069999999985
12.4002999999993 0.153500000000045 1.48870000000007
13.1552999999993 0.508000000000008 0.962400000000067
12.3426999999993 4.64770000000002 -0.53989999999994
17.0792999999995 2.57140000000003 1.14220000000006
19.2537999999995 4.78900000000001 0.833100000000056
20.4871999999992 2.40749999999999 11.0059000000001
19.1211999999995 7.34170000000002 -0.482599999999957
24.0113999999995 7.3003 1.55860000000005

25.4732999999996 6.94900000000002 -0.787499999999977
30.3054999999994 -0.294800000000009 -0.0584999999999622
30.7722999999996 7.0375 -3.86289999999999
35.1037999999995 5.31499999999996 -4.13499999999998
39.0621999999993 10.7178999999999 -6.08679999999997
41.3616999999992 11.9441999999999 -5.45029999999997
39.3548999999992 15.6845999999999 -6.78129999999996
44.5284999999991 17.3674999999998 -1.97169999999995
43.3981999999992 11.1153999999998 -5.33859999999996
42.6665999999992 13.3421999999998 -6.39049999999996
2.40269999999899 1.02850000000004 -1.87989999999992
-0.0310000000010463 2.25520000000005 -2.94759999999993
9.22569999999918 7.07920000000001 0.675600000000078
9.95479999999926 8.38220000000002 0.86580000000007
14.7835999999994 8.71490000000002 1.04990000000006
16.8196999999995 8.69610000000003 0.519100000000065
17.1074999999992 11.9201 11.0836000000001
22.3219999999995 11.1015 1.61580000000005
23.3657999999995 12.536 -0.737399999999966
21.3732999999993 20.5784 -0.177999999999954
27.3992999999994 15.4278 -3.90749999999998
28.2371999999993 19.8683 -4.02989999999997
35.5118999999992 19.0019999999999 -5.86019999999996
37.7653999999992 19.5036999999999 -5.50039999999996
38.8353999999991 21.8130999999998 -4.83189999999996
39.9353999999991 19.3606999999999 -6.42969999999996
0.121899999998929 0.0120000000000361 0.297800000000072
-1.15960000000113 1.20720000000004 -0.948599999999928
8.66969999999916 6.70580000000004 6.90860000000008
10.8886999999993 9.51570000000001 3.43860000000007
10.2172999999992 10.5276 1.40130000000007
9.38469999999917 3.36340000000003 8.03530000000006
29.5328999999992 11.4217999999999 14.4909000000001
37.6976999999991 14.6903999999999 11.1836000000001
36.586099999999 24.6459999999999 -1.42079999999994
45.441099999999 18.1568999999998 2.47790000000007
40.216199999999 21.5190999999998 -3.26859999999994
0.121899999998929 0.0120000000000361 0.297800000000072
-0.0756000000011303 -2.15889999999998 -1.03429999999992
11.4035999999992 0.335900000000037 6.68400000000008
14.8687999999994 0.502600000000043 4.17130000000006
14.9127999999993 -1.27759999999998 2.19740000000005
42.8228999999992 7.65569999999986 -1.42669999999996
43.6983999999992 12.3958999999998 -3.36689999999996
ID=CYCdidFEMAMNH130107

LM3=54
2.78420000000056 0.31930000000038 -2.03240000000023
1.6581000000005 -1.87609999999954 -2.08740000000018
13.1929000000003 1.05880000000032 1.43769999999984
13.9285000000003 1.31560000000028 1.48729999999985
13.2414277738155 4.67293545032561 -0.221675962189297
18.7420000000003 3.56320000000026 1.44519999999987
21.4293000000003 5.70820000000029 0.727099999999864

22.2159000000003 2.72890000000025 10.4861999999998
19.8887000000002 7.34900000000025 -0.306700000000126
24.8095000000003 7.09590000000019 1.9377999999999
27.7202000000003 7.43380000000013 -1.0371000000001
31.7163000000002 0.182400000000099 -0.365500000000126
33.9666000000001 7.61630000000003 -3.91000000000011
37.6931000000002 5.54229999999997 -3.67920000000011
42.0263000000001 11.1276999999999 -4.2366000000001
44.2173000000001 12.8776999999998 -4.5061000000001
41.7487 16.1770999999998 -5.24320000000009
46.4324000000001 17.9118999999998 -0.78860000000012
46.4923000000001 11.5461999999998 -3.26730000000011
45.3607000000001 14.2067999999998 -5.42920000000011
2.76330000000049 1.1699000000004 -1.78370000000021
0.140300000000458 2.35580000000041 -2.61940000000022
9.94740000000033 6.92980000000032 0.731499999999829
11.2986000000003 7.47780000000032 1.24859999999985
16.1699000000003 9.2194000000003 1.06949999999987
19.5462000000003 9.7041000000003 0.487299999999882
17.7888000000003 13.1216000000003 10.2665999999999
22.6689000000003 11.2848000000002 1.6535999999999
24.9316000000003 12.3604000000002 -0.987600000000101
23.3188000000002 21.5534000000002 -0.17230000000011
29.7225000000001 16.627 -3.86520000000009
30.9132000000001 20.5722 -4.2945000000001
38.367 20.0764999999998 -4.22420000000008
40.7320999999999 19.8565999999998 -4.09820000000008
41.7075999999999 22.5477999999998 -3.97210000000008
42.5804999999999 19.7247999999998 -5.88180000000008
-0.0463999999995244 -0.0526999999996264 -0.0442000000002548
-1.34239999999951 1.48210000000041 -1.3625000000002
9.70120000000036 6.98380000000035 6.18989999999982
13.1609000000003 10.3741000000003 2.02659999999985
12.1009000000003 10.9122000000003 1.37549999999986
9.74030000000033 3.23950000000036 7.8420999999998
30.2418000000003 11.9990000000002 14.1924999999999
38.4826000000003 15.2213 11.7901999999999
38.854 24.5212999999998 -0.889400000000102
46.8694000000001 18.0199999999999 4.15049999999989
43.4160999999999 21.9886999999998 -2.11460000000009
-0.0463999999995244 -0.0526999999996264 -0.0442000000002548
-0.243999999999535 -2.15589999999965 -1.20190000000026
11.8757000000003 0.630200000000342 6.37089999999981
16.5032000000003 0.620400000000304 2.70129999999984
16.3184000000003 -0.78409999999969 1.60659999999983
45.5156000000002 7.69539999999991 0.561099999999868
46.8400000000001 12.2926999999998 -2.27340000000011
ID=CYCdidMALCOLAMNH133484

LM3=54
2.73020000000028 0.820199999999883 -1.14879999999999
2.52549999999979 -1.54540000000026 -2.42709999999997
13.5751999999999 1.06199999999999 1.64220000000002
16.5135999999996 1.46329999999983 1.33440000000012

13.2362000000008 4.87160000000022 0.00839999999998287
19.1572000000002 4.13340000000002 1.74130000000005
21.1660571691473 5.37731726667061 0.9381770360793
22.9889 2.89739999999999 9.88120000000003
21.1169000000002 7.53960000000013 -0.38859999999999
25.4573000000004 8.21060000000014 1.58699999999997
28.3475999999998 7.86239999999995 -0.684499999999946
32.3169999999997 0.330699999999923 -0.231199999999931
34.4130999999997 7.98379999999991 -4.49649999999997
37.4018999999996 5.60689999999986 -4.96149999999997
42.4370999999996 10.8004999999998 -6.43339999999998
44.9285999999995 11.9128999999998 -5.65739999999999
43.4466999999996 15.9553999999998 -6.8668
47.7370999999995 16.9168999999998 -3.4051
47.1478999999996 10.9458999999998 -5.37929999999999
45.7051999999996 13.6290999999998 -6.684
2.68360000000041 1.43879999999994 -1.389
1.1366000000003 3.04759999999992 -2.68469999999998
10.9503000000007 8.54530000000015 1.86699999999998
13.0169000000006 9.59680000000017 1.73199999999998
16.6338000000005 9.61050000000016 1.79169999999999
18.9397000000004 9.25130000000014 1.5446
19.9095000000002 13.3675000000001 10.4156
24.0555000000002 11.0136000000001 1.74129999999999
26.0466000000002 12.2688000000001 -0.601000000000017
24.5985000000002 21.1244 0.186299999999979
30.961 16.1917 -4.22840000000001
31.6921 19.873 -4.70780000000002
39.6169999999998 19.1423999999999 -5.56460000000003
41.9330999999998 19.4193999999999 -5.61000000000003
43.4432999999997 21.6651999999998 -4.85390000000002
43.7536999999997 19.2270999999998 -6.67780000000001
-0.0258999999999056 0.374599999999887 0.284400000000054
-0.63189999999982 2.0096999999999 -1.10939999999996
10.7416000000004 6.46730000000008 7.90519999999999
14.3304000000005 10.4652000000001 4.10149999999999
11.6043000000006 12.3298000000001 2.80299999999998
9.98990000000021 4.34800000000004 8.14890000000001
30.8910999999998 11.6141999999999 13.059
40.7398999999997 14.4911999999999 9.77150000000001
40.2479999999997 24.3752999999999 -1.70070000000003
49.1932999999995 17.2567999999998 2.11060000000001
45.4411999999996 21.3314999999998 -4.19300000000001
-0.0258999999999056 0.374599999999887 0.284400000000054
0.821699999999813 -1.71410000000016 -1.15229999999991
12.2392 2.1793 7.56080000000001
17.0069 1.22440000000001 4.18040000000006
17.1818000000003 -1.86919999999991 2.64350000000003
45.0676999999995 5.83459999999982 -1.30829999999996
48.2189999999995 11.9603999999998 -4.31039999999999
ID=CYCdidUNKBRAAMNH133505

LM3=54
2.5489 1.0799 -1.78659999999998

2.34299999999997 -1.17690000000003 -2.87199999999971
12.5328999999998 0.773999999999932 1.12150000000003
15.5987999999998 0.844099999999898 1.11060000000004
13.5204999999998 5.29229999999993 -0.415499999999949
19.0153999999998 2.88419999999991 1.29450000000004
20.7976188237729 4.81977566859112 0.997871181372038
21.2757999999998 1.68389999999988 10.4230000000001
20.3540999999999 7.29529999999993 0.0658000000000573
25.7278999999999 7.00339999999988 2.33810000000004
27.7627999999999 7.2842999999999 0.0409000000000559
31.7307999999999 -0.0298000000001412 -0.194599999999927
34.7250999999999 7.73769999999985 -3.12119999999993
38.0530999999999 5.51279999999985 -3.3034999999999
43.1999000000001 10.2359999999999 -3.05989999999989
44.7545000000003 11.9369999999999 -2.45689999999988
43.0899000000002 15.6453999999999 -3.8407999999999
48.0161000000003 16.7084999999999 -0.254099999999871
47.6118000000002 10.7430999999999 -1.57089999999988
46.4994000000003 13.1763999999999 -3.39249999999989
2.39519999999995 1.03700000000001 -1.57129999999997
1.01169999999999 2.87989999999999 -1.93779999999995
10.0292999999999 7.44909999999997 1.78960000000006
12.3898999999999 8.94279999999995 1.60570000000005
16.2683999999998 9.58289999999991 1.89950000000004
18.8708174213824 9.20496385487933 1.02466368175227
17.5010999999999 11.7432999999999 10.4595000000001
24.0701999999998 10.7590999999999 2.20480000000005
25.5364999999999 12.2338999999999 0.268800000000046
24.692 20.2632999999999 0.0850000000000714
31.6155 16.2602999999999 -3.02979999999994
31.8585 19.9238999999999 -2.52919999999993
40.0011000000002 19.6622999999999 -2.70119999999991
42.2341000000002 19.2785999999999 -2.68819999999989
43.2111000000002 21.9450999999999 -1.68619999999989
44.4787000000002 19.4245999999999 -3.43259999999987
0.0681000000000044 -0.146099999999986 0.152600000000034
-1.35370000000007 1.42080000000001 -1.63949999999996
9.45549999999987 6.90729999999995 6.93650000000006
13.2231999999998 10.3854 2.55290000000005
11.4331999999999 11.0889 3.45080000000005
10.5101999999999 3.59219999999995 8.54330000000006
29.1115999999999 10.3193999999999 14.3441000000001
39.4315000000001 14.0579999999999 12.0645000000001
40.1534000000002 24.6852999999999 1.5782000000001
47.1855000000003 16.7897999999999 6.57470000000013
43.9995000000002 21.8999999999999 0.119500000000129
0.0681000000000044 -0.146099999999986 0.152600000000034
-0.214100000000041 -2.48829999999994 -2.41819999999998
11.7583999999998 0.00789999999991998 5.87510000000004
16.5062999999998 0.73759999999991 3.01120000000005
16.2479999999998 -1.26830000000009 2.64400000000003
46.1937000000002 6.39559999999987 1.64360000000013
47.8592000000002 11.5955999999999 0.382600000000111
ID=CYCdidUNKCOLAMNH139228

LM3=54
2.05819999999985 0.735200000000018 -1.6225
2.0037999999995 -1.20769999999988 -2.34559999999995
12.0702999999998 0.856500000000013 0.945799999999994
13.8274999999998 1.24079999999998 0.814400000000002
12.0961999999998 4.79989999999998 -0.735699999999993
17.4685999999999 3.56400000000001 0.744999999999993
19.0750999999999 4.59829999999999 0.469199999999992
19.0545999999997 2.42159999999997 9.87939999999999
20.1102999999999 7.57369999999999 -1.24650000000003
23.5813999999999 6.40079999999998 1.15569999999998
25.7400999999999 6.6237 -0.888200000000038
29.1709999999998 -0.0121000000000161 0.301499999999957
31.6966999999998 7.27439999999997 -3.69480000000006
35.2522999999997 4.98089999999994 -3.72910000000006
40.1158999999995 9.23499999999986 -3.26150000000006
42.0604999999994 10.2655999999999 -2.91650000000007
40.1942999999995 14.3611999999999 -4.40500000000008
44.4099999999994 15.7954999999998 -0.390500000000062
43.9925999999993 10.0619999999998 -1.92500000000007
43.2881999999994 11.9774999999998 -4.06880000000007
1.86839999999974 0.904199999999985 -1.387
0.657999999999758 2.53599999999999 -2.30540000000001
9.70599999999977 7.23919999999996 0.77520000000001
11.6782999999998 8.34939999999997 0.792000000000007
15.3385999999999 8.72099999999998 0.766700000000002
16.9019999999999 8.74489999999999 0.517799999999992
15.9589999999998 11.0762999999999 9.4662
22.0003999999999 10.2308 0.665999999999964
23.8477999999999 11.8302 -0.976600000000026
22.5351999999998 18.7703999999999 -0.124600000000047
29.2758999999998 14.8589999999999 -3.66500000000006
29.8869999999998 18.2707999999999 -3.86440000000007
36.5602999999996 17.9301999999998 -3.39720000000007
39.2757999999996 17.9455999999998 -3.14140000000007
39.9533999999995 19.7674999999998 -2.60520000000007
41.0193999999995 17.9684999999998 -4.06500000000007
0.0245999999997167 -0.224600000000036 -0.0142000000000177
-1.39480000000028 0.924999999999971 -1.15790000000002
8.3469999999998 6.61329999999999 5.6226
11.7088999999998 9.48939999999997 2.19720000000001
11.3169999999998 10.4514999999999 0.350400000000003
9.63949999999978 3.71369999999999 7.4682
27.7120999999997 9.97379999999993 12.8768
36.8709999999995 13.5977999999999 10.6443999999999
37.8634999999996 23.0105999999998 0.716099999999938
45.1925999999994 16.3104999999998 3.76939999999995
39.9932999999994 20.3643999999998 -2.14670000000007
0.0245999999997167 -0.224600000000036 -0.0142000000000177
-0.273200000000328 -2.18220000000002 -1.28410000000002
10.6740999999998 0.717900000000004 5.57649999999999
14.6275999999998 0.835300000000012 2.63369999999999
15.0664999999998 -0.447199999999999 0.941399999999986

43.4954999999994 6.86419999999987 0.813499999999944
43.7108999999993 10.7131999999998 -1.94760000000006
ID=CYCdidFEMCOSAMNH139460

LM3=54
2.45539999999938 0.90419999999969 -1.84319999999996
1.9106000000007 -1.52340000000003 -2.69579999999999
13.2997999999999 1.26269999999991 -0.0830999999999562
15.2611000000002 1.78570000000001 -0.649899999999976
12.2767 5.15830000000002 -1.79480000000001
17.7906999999998 3.38239999999983 -0.663299999999961
19.2779940153145 5.14106253390148 -0.881615976983217
19.5667999999998 2.17429999999987 9.46230000000003
20.2084999999999 8.29049999999993 -1.90419999999999
24.2515999999995 8.01239999999982 0.910200000000039
27.3483999999999 7.99689999999992 -1.73849999999999
31.0215999999997 0.156399999999861 -0.106700000000001
33.0385000000001 8.9398 -3.99330000000002
36.9793999999997 6.18449999999989 -3.6614
41.4339999999997 12.3509999999998 -3.77390000000002
43.3114999999998 14.0999999999998 -3.37930000000002
41.6210999999998 17.8154999999999 -4.83990000000003
45.6217999999999 19.2929999999999 -0.629300000000046
45.8141999999999 13.2178999999999 -2.08320000000003
45.0832999999999 15.3025999999999 -4.08210000000003
2.10569999999939 1.13809999999967 -1.8075
0.342599999999688 3.11129999999979 -2.76740000000001
10.2044999999998 8.29409999999986 -0.00540000000000629
12.2281999999999 9.62179999999989 -0.456699999999999
15.4166999999999 10.0121999999999 -0.665899999999988
19.4452999999999 10.4979999999999 -0.984399999999992
15.3462999999998 11.5962999999999 9.4347
22.3402999999997 11.5412999999999 0.240000000000013
24.5045999999997 13.3943999999999 -1.84669999999999
21.4365999999998 21.7785999999999 -0.562600000000013
28.7945999999999 17.2860999999999 -4.07050000000002
29.5076999999999 21.7678999999999 -4.13610000000002
37.4629999999999 21.0343999999999 -3.68290000000003
39.8529999999998 21.6902999999999 -3.66740000000003
40.8357999999999 23.8297999999999 -1.68990000000004
41.9544999999999 21.5203999999999 -4.32430000000004
0.133099999999895 0.178299999999841 0.312299999999992
-1.21490000000019 1.84189999999981 -0.89540000000001
9.71349999999974 6.74719999999983 6.04150000000002
12.2164999999999 10.7533999999999 1.61820000000001
10.6365999999998 12.2198999999999 0.4791
9.87019999999969 3.85229999999981 6.82930000000002
28.2852999999998 11.2436999999999 13.2793
37.2912999999998 15.0140999999998 11.1335
36.9819999999999 25.9022999999999 1.04429999999996
45.6307999999999 19.0867999999999 4.99799999999997
41.6185999999999 23.3568999999999 -0.623000000000034
0.133099999999895 0.178299999999841 0.312299999999992
0.109800000000343 -2.1179 -0.770100000000028

11.6842999999996 1.68419999999981 5.97080000000003
15.9058999999995 0.503999999999758 1.56960000000005
16.3695999999991 -0.9047000000004 0.156700000000075
44.8124999999998 7.98839999999983 0.991699999999979
46.0267999999998 13.3920999999998 -0.567300000000031
ID=CYCdidMALPERAMNH167845

LM3=54
2.3318 0.7554 -0.9652
1.451 -1.3695 -1.8923
12.128 0.8473 1.3821
13.2836 1.0982 0.9995
11.8902 4.7178 -0.4712
16.2016 2.6018 1.0851
17.9605 4.2144 0.5319
17.5416 2.0271 9.7452
18.4219 7.3116 -0.7489
22.113 6.5416 1.3999
23.0474 6.1501 -0.3025
26.9972 0.1108 -0.054
28.7487 6.4048 -3.0128
32.8205 4.9135 -2.987
37.8759 9.5225 -3.9354
39.4495 11.2119 -3.6203
38.133 14.9212 -4.4318
42.0374 16.3318 -0.7955
41.4727 9.8171 -2.9171
40.7188 12.701 -4.5313
2.2908 1.0539 -0.9664
0.1816 1.6861 -2.0048
9.0196 7.8935 1.2811
10.0685 8.2714 1.2923
13.9525 8.665 0.8224
15.4707 9.1657 0.304
14.9842 11.5419 9.6023
20.3406 10.4628 1.3009
21.1907 11.5259 -0.553
19.6834 18.4793 -0.0478
25.0184 15.0109 -3.0906
27.4933 18.4707 -3.2517
34.4366 18.895 -3.9801
36.6273 18.4379 -3.5609
36.8557 20.9443 -2.8581
38.4607 18.2594 -4.5093
0.0879 -0.1916 -0.0797
-0.8147 1.3114 -0.5445
7.5256 5.9867 5.8004
11.1166 9.8939 2.4679
9.4283 10.1062 2.4089
7.9907 3.3166 6.9881
27.4597 10.82 12.5649
34.935 13.6131 10.9206
34.9086 23.5767 0.2433
42.4497 16.6464 3.9621

38.8154 20.2815 -1.3349
0.0879 -0.1916 -0.0797
0.5365 -2.0867 -0.5598
9.7193 0.556 6.0462
14.4278 0.3239 2.6716
13.7316 -1.1619 2.4108
40.6288 6.19 0.1917
42.0083 10.9002 -1.3853
ID=CYCdidMALVENAMNH16956*

LM3=54
3.05009999999956 0.602199999999679 -1.45709999999996
1.88030000000011 -2.20280000000033 -2.26640000000011
13.202100000001 0.718300000000113 0.974099999999938
14.2618999999998 0.712699999999786 0.867900000000043
13.4753999999999 4.9041999999997 -0.798599999999921
18.7200999999998 3.23139999999975 0.838000000000016
20.4085000000001 4.78149999999991 0.338000000000027
20.8071000000002 2.23649999999991 10.3321
21.3794000000001 8.02889999999988 -0.967399999999951
26.1152 7.78479999999992 1.13380000000001
26.9754999999998 6.72099999999989 -0.793999999999937
31.7707000000003 0.231500000000055 -0.308999999999973
33.5602000000002 7.88260000000006 -3.36109999999998
37.9834000000005 5.38890000000014 -4.23499999999998
42.4493000000004 11.1022000000002 -4.367
44.0523000000003 12.2825000000002 -4.36689999999999
43.1076000000003 16.3179000000002 -6.52209999999999
46.2129000000003 17.9660000000002 -2.0191
46.1624000000003 12.0852000000002 -4.28159999999999
45.7435902538415 13.8736429835496 -5.91543084772417
2.92909999999962 0.989899999999654 -1.40519999999994
0.703399999999621 2.16249999999965 -2.81239999999995
10.3854999999999 7.95569999999973 0.667100000000028
11.1884999999999 8.83389999999975 0.511400000000038
15.7182999999999 9.6050999999998 0.717800000000032
18.3176 9.59589999999984 0.362700000000035
17.2202000000001 12.0088999999999 10.463
24.5560000000001 11.2375 1.15620000000003
24.7106000000002 12.797 -0.669899999999976
23.1847 21.0066999999999 -0.0584999999999735
30.0577000000001 16.1277 -3.27999999999998
31.1161000000001 20.9136000000001 -3.79219999999999
38.2723000000002 19.9856000000001 -4.60829999999999
40.7376000000002 20.4670000000002 -3.96479999999999
41.9256377416121 22.4546194912443 -3.39809293332929
43.131689105959 20.5802262224351 -5.51563139159796
0.301299999999738 -0.195000000000333 0.567400000000054
-0.876900000000325 1.33959999999965 -1.02169999999995
9.56440000000007 7.01059999999979 6.20380000000002
12.6875999999999 9.94459999999977 2.77390000000003
11.1643999999999 11.1929999999997 0.519800000000033
9.11770000000025 3.25169999999984 7.3551
30.7697000000003 11.3774 14.1718

39.3935000000004 15.0540000000001 11.3038
38.2248000000002 25.0621000000001 0.556800000000014
46.9729000000004 18.1546000000002 3.73840000000002
43.8546000000003 22.1073000000002 -2.43429999999999
0.301299999999738 -0.195000000000333 0.567400000000054
0.274799999999809 -2.40460000000033 -0.573099999999944
12.0643000000005 0.871699999999914 6.40059999999999
16.2452000000002 0.251499999999852 3.10450000000001
15.9634000000004 -1.59510000000014 1.36120000000005
45.5528000000004 7.73420000000019 1.03110000000001
46.8630000000004 13.1717000000002 -2.21189999999999
ID=CYCdidFEMTRIAMNH174172

LM3=54
1.74339999999826 0.439599999999918 -2.30549999999982
1.15499999999812 -2.08030000000012 -2.8726999999998
11.4599999999986 0.509999999999916 0.695100000000114
13.7279999999986 0.719799999999892 1.1993000000001
13.3854999999986 5.42509999999991 -0.281899999999915
19.0031999999989 3.59939999999994 1.38920000000009
20.319699999999 4.85389999999993 1.11600000000008
20.1665999999987 2.46719999999989 10.1856000000001
20.9329999999991 8.55229999999995 -0.229299999999936
25.1771999999992 7.88099999999994 1.00780000000006
26.7805999999993 7.22509999999996 -0.562699999999971
31.5530999999992 -0.192600000000079 -0.0163999999999425
33.4915999999995 8.05659999999992 -3.61899999999997
36.8410999999995 5.44849999999988 -4.09629999999996
41.6011999999994 11.2011999999998 -5.12219999999996
44.3093999999995 12.7619999999998 -3.87339999999995
42.4482999999994 16.7732999999998 -5.54929999999995
47.1961999999994 18.3433999999998 -2.60199999999993
46.2931999999994 11.8074999999998 -3.27669999999995
45.4309999999994 14.2421999999998 -5.54109999999996
1.46069999999815 1.00669999999984 -2.24779999999985
-0.290800000001875 2.41219999999985 -2.93829999999985
8.98569999999847 7.76639999999987 0.923700000000131
10.5016999999986 8.3134999999999 1.30120000000011
16.2085999999988 10.3714999999999 1.5594000000001
18.005399999999 10.6159 1.08390000000009
17.7356999999986 12.7021999999999 10.4364000000001
23.2992999999992 11.6443 1.02950000000006
24.6596999999993 13.2765 -0.246999999999942
22.533099999999 21.4096999999999 -0.156299999999928
29.8150999999993 17.0414999999999 -3.68639999999996
30.3773999999993 21.1892999999999 -4.45989999999995
38.9462999999993 21.2647999999998 -4.82789999999995
41.2384999999993 21.1927999999998 -4.11319999999995
42.0157999999992 22.9155999999998 -3.94599999999994
43.2574999999993 20.6481999999998 -5.38629999999994
-0.0436000000019469 -0.0556000000001642 0.133400000000157
-1.864100000002 1.10669999999984 -1.18389999999984
8.95969999999841 7.30389999999988 6.27620000000013
11.8157999999986 10.5422999999999 3.12150000000011

11.2444999999985 11.7938999999999 1.08590000000012
10.3590999999984 3.87039999999988 7.95430000000012
30.4253999999989 12.4240999999999 13.8774000000001
39.495699999999 15.8317999999998 10.7662000000001
38.9758999999991 26.3918999999998 -0.130799999999911
47.4262999999992 19.2260999999998 3.16200000000008
43.2039999999992 22.8580999999998 -2.42109999999992
-0.0436000000019469 -0.0556000000001642 0.133400000000157
-0.655700000002006 -2.18300000000018 -1.37519999999984
11.6223999999985 0.661799999999895 6.13300000000011
15.8516999999987 -0.104100000000074 2.73350000000009
16.4278999999987 -1.22420000000009 0.806100000000074
46.7175999999993 7.80689999999979 0.217600000000067
47.1567999999994 13.6112999999998 -2.23079999999995
ID=CYCdidMALBOLAMNH262656

LM3=54
2.24330000000217 0.802200000000662 -1.89080000000024
2.45230000000217 -1.1384999999995 -2.68660000000026
12.0469000000017 1.07850000000054 0.974999999999864
13.5506000000017 1.62140000000055 1.35309999999985
11.5768000000016 4.86800000000055 0.0951999999998665
16.1363000000015 3.25630000000048 1.80239999999986
17.7826938576006 4.95178571351303 1.56270641468016
18.2578000000014 1.94270000000045 10.8200999999998
18.6782000000012 7.89240000000044 0.45089999999987
22.314300000001 6.87350000000037 2.37009999999988
24.2365000000009 6.92110000000033 0.314799999999875
28.8715000000007 -0.0209999999997097 0.314399999999843
30.1664000000003 8.46840000000021 -2.42900000000014
33.7956000000001 5.58140000000014 -2.99340000000016
38.0589999999997 11.0588 -2.74850000000015
39.8177999999996 11.9338 -1.94020000000016
35.7114999999997 14.5198 -2.76470000000016
42.2337999999997 17.3342 0.846199999999844
42.4596999999996 11.4883 -1.19940000000017
41.8949999999996 13.6586 -2.46180000000016
1.96880000000218 1.34010000000064 -1.92390000000021
0.974500000002209 2.87550000000066 -2.54100000000022
8.65670000000182 7.43840000000055 0.888699999999852
10.7529000000017 8.54800000000053 1.27329999999985
13.4532000000015 9.0718000000005 1.76039999999985
15.7434000000013 9.56970000000046 1.37499999999986
14.1695000000014 11.2887000000004 11.2013999999999
20.123700000001 10.9575000000004 2.14149999999987
21.9708000000009 12.6219000000003 0.172099999999863
20.5714000000009 20.0211000000003 0.616499999999839
26.7775000000005 15.4021000000003 -2.65600000000016
27.8591000000004 19.8797000000002 -2.93080000000016
34.6992 19.0065000000001 -2.44600000000016
36.9858999999999 19.7792000000001 -1.54230000000016
38.1023999999999 21.8800000000001 -1.07570000000016
38.5117999999999 20.0870000000001 -2.49010000000017
0.213700000002258 -0.535099999999346 -0.242800000000213

-1.15159999999772 1.14590000000066 -1.13510000000022
7.1208000000019 6.70260000000057 6.03469999999985
10.0384000000017 9.10140000000052 3.40889999999986
9.81700000000167 10.9394000000005 1.45829999999985
8.63710000000188 3.32110000000056 7.84449999999985
26.9124000000009 11.0879000000003 14.5407999999998
36.1510000000004 15.3595000000002 11.8708999999998
35.0783000000002 24.9362000000001 1.23349999999983
42.5745999999999 18.2869000000001 5.93299999999984
39.1646999999999 21.5387000000001 -0.0942000000001757
0.213700000002258 -0.535099999999346 -0.242800000000213
0.478800000002279 -2.04129999999934 -1.29830000000021
10.4682000000019 -0.209799999999435 5.63709999999985
14.1287000000017 0.71480000000052 3.37859999999986
15.0493000000016 -0.920599999999494 1.32499999999984
42.4746999999998 7.79310000000005 1.02189999999983
42.8522999999996 13.6499 0.175699999999839
ID=CYCdidFEMNICAMNH30755*

LM3=54
2.6418 0.5593 -2.0609
1.8884 -1.9799 -2.8661
12.0236 0.9062 0.2669
13.1774 0.9353 0.4314
12.6019 4.7647 -0.7727
17.5002 3.9301 0.8124
19.1509 5.0778 0.3548
19.3856 2.6098 9.7275
19.4211 7.2859 -1.025
23.7318 7.6218 1.3634
25.522 7.436 -0.6828
29.4651 -0.2538 -0.1478
31.3751 7.3381 -3.6566
34.8063 4.9118 -3.9599
39.7257 9.7771 -3.5781
41.7182 11.3541 -3.4791
40.1717 15.0048 -4.938
43.6995 16.3478 -0.5587
43.092 9.9604 -2.9217
43.249 12.4315 -4.4105
2.1889 1.3342 -1.8536
0.4215 2.637 -3.0619
8.9888 7.1439 0.4315
10.8774 8.2353 0.1909
15.1408 8.8135 0.8501
17.4745 9.0743 0.3845
16.7952 11.3739 9.7538
22.5669 10.6735 1.524
23.5145 11.8217 -0.7834
21.9317 19.9274 -0.2122
27.8389 15.1494 -2.8851
28.8172 19.4187 -4.1148
36.2589 19.3088 -3.8001
38.8565 19.2331 -3.4877

39.2081 21.5126 -2.9865
40.7134 19.3433 -4.3438
-0.0038 -0.1996 -0.1742
-1.2173 1.0647 -1.1833
9.0031 6.8537 5.1599
11.9455 9.5016 2.5975
10.5202 10.8396 1.2223
9.4958 3.2188 6.8297
28.4096 10.7956 12.4693
35.3455 13.5413 10.5653
36.298 24.2971 0.3528
43.8994 16.917 4.9789
40.096 21.2519 -1.9092
-0.0038 -0.1996 -0.1742
-0.0639 -1.9717 -1.1221
11.7346 0.3933 5.1998
15.5005 1.0141 2.6579
15.4994 -0.693 0.7621
42.5492 5.7857 0.7543
43.7507 10.9446 -1.5478
ID=CYCdidFEMBRAAMNH37473*

LM3=54
2.94030000000038 0.434000000000371 -1.33609999999995
2.50040000000018 -1.46189999999965 -2.06139999999995
11.9779000000004 0.709700000000234 0.870300000000066
14.8324000000003 1.6200000000002 0.939200000000074
11.9548000000004 4.2901000000002 -0.258999999999961
16.8868000000002 2.85810000000013 1.39220000000007
19.2718000000003 4.9588000000001 0.596000000000052
20.2327000000001 1.5134000000001 9.30850000000011
18.7597000000002 6.98950000000008 -0.691699999999971
22.9736000000001 6.1766 1.77780000000004
24.1958 6.23779999999998 -0.458199999999988
28.8749999999998 0.0285999999999595 0.198500000000043
29.7417999999998 6.91419999999994 -3.45450000000001
33.3353999999996 5.48569999999989 -4.17039999999999
37.8088999999993 10.4811999999998 -4.23870000000001
40.0760999999992 11.6269999999998 -4.4649
38.2233999999994 14.9640999999998 -5.40860000000003
42.4064999999992 16.4073999999998 -1.98889999999998
41.8289999999992 10.7024999999998 -4.03859999999998
40.9229999999992 12.8235999999998 -5.3396
2.20520000000038 0.960100000000379 -1.11879999999997
0.996000000000377 2.63100000000039 -1.77889999999997
8.99550000000042 6.99020000000024 1.09650000000006
12.0375000000004 8.11680000000022 1.12300000000005
15.3764000000004 8.21830000000014 1.26400000000006
16.8369000000003 8.48600000000011 0.893400000000044
17.1977000000002 11.5901000000001 9.96660000000012
21.2620000000002 10.2237 0.817800000000037
22.1585000000002 11.2283 -0.780399999999991
20.7408886121583 19.7725195162594 -0.150954160184832
26.4142999999999 15.2199 -3.67680000000002

28.2502999999998 18.3989999999999 -4.20050000000002
35.0653999999996 17.8727999999999 -4.29510000000003
37.5222999999995 18.3462999999998 -4.31090000000002
37.9694999999995 20.3580999999998 -3.69990000000002
38.9559999999994 17.6931999999998 -5.36430000000003
0.0662000000003669 -0.229299999999596 0.0132000000000482
-0.335299999999597 1.11550000000042 -0.538999999999965
9.0965000000004 6.28620000000026 6.1268000000001
12.7954000000004 9.06660000000019 3.30090000000007
10.9985000000003 10.3220000000002 1.61680000000006
10.1771000000003 3.26950000000024 7.64840000000011
27.2462999999999 9.65909999999997 12.6363000000001
35.7657999999995 13.1140999999999 10.2754000000001
36.2913999999996 23.3273999999999 0.766000000000016
43.6268999999992 16.3063999999998 3.53040000000004
39.9671999999994 20.2156999999998 -2.1592
0.0662000000003669 -0.229299999999596 0.0132000000000482
0.781100000000402 -2.04439999999958 -0.752399999999971
11.2668000000003 0.223400000000235 6.0379000000001
15.7719000000003 0.555800000000174 3.06690000000008
15.4027000000003 -0.721999999999807 1.11930000000007
43.1107999999992 6.6503999999998 0.0992000000000449
43.3955999999992 11.3651999999998 -2.42369999999998
ID=CYCdidFEMCOLAMNH37786*

LM3=54
1.073 0.2363 -2.2948
0.5355 -1.8935 -3
10.4668 0.2509 0.1897
12.4791 0.7489 0.0726
10.5362 4.571 -1.0591
15.3685 3.0398 0.4192
17.0896 4.2891 0.1239
18.0398 1.8661 9.4743
16.6494 7.0276 -0.957
21.6691 7.2995 1.4804
23.8628 6.7994 -0.8779
27.2361 0.1526 -0.1388
28.8398 7.9072 -2.6353
33.0728 5.6584 -2.6979
37.3441 11.0567 -1.9609
39.5793 12.3196 -1.412
37.4826 15.9672 -2.3527
41.1163 17.4998 2.1239
41.319 11.199 -0.9039
40.6134 13.5755 -1.9455
0.9766 0.6627 -2.5113
-0.8258 2.1469 -3.129
7.0562 6.7698 -0.1249
9.5473 8.5638 0.0713
12.8015 8.9711 0.3881
14.8176 8.9104 0.2622
13.6485 11.9519 9.5944
19.9564 10.2094 0.9503

21.601 12.2675 -1.3022
18.3766 19.4041 -0.2626
25.1019 14.9512 -2.7582
26.456 19.6221 -2.6751
33.5985 19.1575 -2.1826
36.1068 19.9781 -1.4967
36.5535 21.9591 -0.6902
37.9253 19.9274 -2.0023
-0.0768 -0.0671 -0.1289
-1.6696 1.1083 -1.28
6.8203 7.1997 4.6703
9.633 9.8864 1.7986
9.0809 10.3063 0.259
7.8838 3.1436 5.8806
24.2508 10.7351 13.736
33.9847 14.7287 12.009
33.0094 24.791 1.9586
40.6336 17.2161 7.6855
37.5134 22.0791 1.0432
-0.0768 -0.0671 -0.1289
-0.3322 -2.1416 -1.4517
9.8433 -0.2156 4.2427
13.0062 0.4141 1.8931
13.7487 -1.0455 0.4667
39.8732 7.3548 1.8057
41.5053 12.5209 1.0354
ID=CYCdidFEMBRAAMNH95506*

LM3=54
3.7715 0.7141 -1.1291
2.2424 -1.815 -2.1776
13.0986 0.6417 1.8721
15.4297 1.5977 1.7045
12.9748 4.9188 -0.283
20.0427 4.4932 1.0345
21.6781 5.5038 0.7096
20.0082 2.3209 9.8972
21.2164 8.1268 -0.8073
24.8828 7.5923 1.3707
26.8289 7.4707 -0.3978
30.9531 0.1325 0.1049
34.3051 7.7522 -4.0651
36.2369 5.0427 -4.5104
41.8278 10.7882 -3.9695
43.8226 11.617 -3.4421
42.1039 15.6992 -4.7747
46.2446 16.8932 -0.7126
46.002 10.9243 -2.8416
45.2277 13.2505 -4.5942
3.3131 1.5714 -0.9478
0.8754 2.7257 -2.317
10.0049 7.3701 1.5311
12.7335 9.1419 1.5872
18.2674 9.8125 1.0773

20.0546 9.9487 0.7251
17.2458 11.2444 9.961
23.7842 10.434 1.1391
25.1699 12.3461 -0.764
23.4578 20.0305 -0.1671
31.4013 16.1777 -4.0504
31.3222 20.0102 -4.497
38.7414 19.3874 -3.7423
40.8776 19.3437 -3.2896
42.2237 21.997 -2.5944
42.8681 19.2961 -4.4734
-0.0389 0.0074 0.0449
-1.1169 1.422 -1.0809
10.1171 7.1701 6.6317
13.622 10.0758 4.443
12.0773 10.7668 1.8876
10.8696 4.3477 8.2876
30.9313 11.3252 12.7812
39.3377 14.4573 10.4757
40.214 25.1209 0.653
47.2255 17.3885 4.1308
43.6399 21.002 -2.2804
-0.0389 0.0074 0.0449
0.2079 -1.8979 -1.1658
12.2934 1.241 6.9388
16.5204 0.8553 4.1443
16.1316 -0.7127 1.9314
46.0142 6.7098 -0.1854
46.6258 12.0171 -2.3204
ID=CYCdidFEMBRAAMNH96446*

LM3=54
3.5798 0.8592 -1.1366
2.5536 -1.4574 -2.4396
13.2937 0.8287 0.1928
16.6172 1.4959 0.2228
13.4019 4.9345 -1.452
19.6902 3.8637 -0.002
20.7146 4.9537 -0.189
20.856 1.9275 9.5538
20.2146 7.2266 -1.2086
25.647 7.2759 0.77
28.173 7.5169 -0.8785
32.7471 0.2617 -0.1582
34.8386 7.6607 -3.5141
38.334 5.1882 -3.6424
43.8951 10.0713 -2.7829
46.0392 11.7017 -1.8708
44.2229 15.6779 -3.2402
47.7222 16.6331 1.2729
48.377 10.6332 -1.059
47.7303 13.1924 -3.1392
3.3608 1.6272 -1.3038
0.9261 2.7464 -2.5403

10.6379 7.7637 0.5151
13.1937 9.0539 0.4194
14.6565 7.5109 -0.5759
18.9891 9.2695 -0.0429
18.035 11.5525 10.4307
24.2666 10.7189 0.9393
26.4994 12.5081 -1.0309
25.2943 20.7925 -0.2059
32.1462 16.1799 -3.1488
33.5067 20.2923 -3.4356
40.6866 19.5456 -2.3968
43.0668 19.6241 -2.0665
43.8271 21.7098 -0.9141
44.7999 18.7416 -2.9366
-0.0102 -0.2421 0.0724
-0.8145 1.493 -0.7341
10.3135 7.0052 6.165
13.706 9.9254 4.1255
12.2387 11.0577 1.1053
11.3904 4.0235 7.3278
29.9619 10.4332 13.2037
38.1645 13.3622 12.3647
40.4762 24.1861 3.611
47.0189 16.7639 8.0731
44.4527 22.0385 0.4197
-0.0102 -0.2421 0.0724
0.2852 -1.7533 -1.0257
12.8797 0.2975 5.8816
16.4084 0.564 3.3565
17.0659 -0.7893 1.1923
45.9334 6.1802 2.6312
48.1267 10.9125 -0.4792
ID=CYCdidMALBRAAMNH96449*

LM3=54
3.71449999999988 1.12549999999983 -1.24229999999996
1.83910000000174 -1.68879999999945 -2.41880000000024
13.9805999999991 1.14969999999957 0.447200000000141
16.7013999999999 1.09349999999984 0.458800000000055
14.0804000000009 5.22150000000024 -1.17240000000009
20.5452000000003 4.21810000000001 0.158299999999979
21.6295000000003 5.96050000000001 0.165699999999979
21.9133000000002 2.61209999999996 9.58820000000003
21.2059000000001 8.00879999999991 -1.51189999999994
26.0966000000006 8.06260000000004 0.417099999999971
27.3175000000004 8.1430000000001 -1.44380000000001
32.0167000000003 0.0327000000000668 0.0569999999999915
34.6023 8.3649 -5.02309999999998
37.3340999999999 5.88429999999998 -5.29889999999998
42.4947 11.1628 -6.0973
44.6509 12.4445000000001 -6.23810000000001
43.0650000000001 16.3981000000001 -7.30690000000003
47.4844999999999 17.8816 -3.81090000000002
47.2128 11.4557000000001 -5.25700000000002

46.1573000000001 13.9899000000001 -7.39030000000003
2.8838999999999 1.83289999999985 -1.34049999999994
-0.113999999999681 2.92269999999996 -2.51219999999999
11.0932000000003 8.99670000000002 0.970099999999991
12.9336000000004 9.80470000000004 0.594899999999983
16.6080184688282 9.56612866336084 0.626905749170388
18.9325902688883 9.72725288794043 0.0251498475311051
18.3975000000001 12.0544999999999 10.1705
24.5611000000002 11.2612 0.874500000000019
25.5510000000002 12.2661 -1.48169999999999
23.7821000000002 21.3052 0.107300000000006
30.7988000000001 17.0994 -4.91879999999999
31.4299000000001 20.6739000000001 -5.05119999999999
39.1229 20.6243000000001 -5.9101
41.4224999999999 20.7261000000001 -5.9575
42.6483 23.1084000000001 -5.43680000000001
43.5615 19.9971000000001 -7.36890000000002
0.0375000000005837 0.0126000000000027 0.0346999999999984
-1.15679999999947 1.49960000000002 -1.00110000000001
11.7788999999999 7.24169999999987 6.77490000000006
13.9298000000002 10.435 3.7051
12.8904000000003 12.6713 0.742899999999994
12.0797999999998 4.3832999999998 7.69650000000007
32.8920000000002 12.2748 12.5086
40.8034000000001 15.4003 9.55660000000001
40.0265 25.7592 -1.3472
48.4712999999999 18.3078 2.05579999999999
44.1486 22.5045000000001 -4.72570000000001
0.0375000000005837 0.0126000000000027 0.0346999999999984
0.0463000000012563 -1.9788999999998 -0.822200000000107
13.7434999999997 1.78199999999975 6.7174000000001
17.2114999999999 0.94379999999983 3.61150000000007
18.2088000000001 -0.992800000000148 0.576400000000076
46.2199000000001 7.69310000000008 -2.15870000000002
47.6971000000001 12.1956000000001 -4.70700000000002
ID=CYCdidMALBRAAMNH96452*

LM3=54
3.0677 0.3064 -1.7249
2.1824 -1.8371 -2.4607
13.3564 0.8467 1.1201
15.4121 1.656 0.7103
12.9079 5.1291 -0.908
18.1628 3.5928 0.8371
19.886 5.1366 0.6152
21.6373 2.6238 9.9812
20.3763 7.8558 -0.7262
25.9211 8.1147 0.4301
27.5349 7.5795 -1.4479
31.1206 -0.138 0.296
33.3723 8.4173 -4.8314
37.1032 5.3538 -4.2913
42.3416 10.9906 -4.9527
44.2405 12.3855 -4.5677

41.97 16.1322 -5.9334
46.3877 18.281 -1.2367
46.0434 11.4085 -3.5037
45.2852 14.0544 -5.2327
2.6607 1.3963 -1.171
0.0907 2.76 -2.3505
9.9397 7.9837 1.0138
12.294 9.2417 0.8927
15.3859 9.7246 1.0868
17.5889 9.7707 0.6383
18.4682 13.6907 10.8613
23.9945 11.4778 0.4226
24.4352 12.4545 -1.2616
22.7968 21.4611 0.0867
30.244 16.4164 -4.5107
30.2 21.0395 -4.0713
38.3817 20.5734 -4.8826
40.6912 20.2358 -4.4317
41.1353 22.8127 -3.6589
42.421 20.3843 -5.5878
-0.0394 0.1831 -0.0473
-0.9403 1.6792 -0.8997
9.8715 7.5574 6.6439
12.398 11.3845 2.4106
11.2085 11.9581 1.4793
10.807 4.4129 7.6743
30.4651 11.8101 13.7024
37.473 14.6091 12.0352
38.7286 26.0834 -0.7051
46.3139 17.8403 4.9342
42.3462 22.8221 -2.4712
-0.0394 0.1831 -0.0473
0.2445 -1.9997 -0.6138
12.4564 1.7547 6.4945
16.0341 1.2628 2.1867
16.4502 -0.6349 0.8219
45.9349 8.1546 -0.548
46.2148 13.6103 -2.2638
ID=CYCdidMALPERAMNH98523*

LM3=54
2.5949000000005 0.883300000000183 -1.74539999999998
2.26510000000038 -1.16230000000002 -2.59879999999997
11.7227000000004 1.28090000000013 0.579500000000027
12.7559000000003 1.52960000000012 0.598600000000019
10.8785000000004 4.62250000000016 -0.632399999999989
15.9410000000003 4.37700000000011 1.10880000000001
17.1175000000005 5.09270000000016 0.820500000000015
18.5737000000003 2.7223000000001 10.775
18.3448000000005 8.14800000000016 -0.646799999999989
22.0207000000003 6.89910000000007 1.28140000000003
23.4868000000004 6.96550000000009 -0.509199999999981
28.2491000000003 0.464400000000036 -0.0291999999999653
29.3728000000002 7.2318 -3.92589999999999

33.6678000000001 6.15189999999994 -4.18399999999997
38.1786999999999 10.9561999999998 -3.75709999999995
39.6787999999998 12.3336999999998 -3.17739999999993
38.0764999999998 16.1593999999998 -4.82879999999994
41.9276999999998 17.8847999999998 0.148500000000097
42.2352999999997 11.7711999999998 -2.46229999999992
41.5317999999997 13.7181999999998 -4.44449999999993
2.72750000000051 1.71820000000017 -1.87309999999998
1.20130000000051 2.75600000000018 -2.57969999999997
8.93440000000048 8.17280000000019 0.393800000000022
9.9137000000005 8.5433000000002 0.733100000000029
13.8084000000004 9.11750000000014 0.750000000000022
16.3629000000004 9.43420000000014 0.457100000000018
15.1386000000004 11.0500000000001 10.8407
20.4513000000004 11.3205000000001 0.652500000000016
21.2793000000003 12.6140000000001 -0.718799999999982
20.0943000000003 20.3533000000001 -0.515999999999966
25.5673000000002 15.9484 -4.03129999999998
27.4159000000001 19.8748 -4.07749999999998
34.4102999999999 19.9622999999998 -3.26589999999994
36.9308999999998 19.9094999999998 -3.66399999999992
37.9340999999998 21.9238999999998 -2.25699999999993
39.0896999999998 20.6137999999998 -4.38559999999992
-0.0951999999995316 -0.0278999999998479 -0.0216999999999598
-1.23299999999951 1.26140000000016 -1.07109999999996
6.79920000000045 6.00330000000017 5.29860000000004
11.3046000000005 9.21670000000018 2.43670000000003
9.42770000000044 11.2477000000002 1.03600000000004
9.19440000000043 3.73430000000017 7.63140000000005
26.8368000000002 10.8543 14.2352000000001
34.8278000000001 14.2249999999999 12.1725000000001
35.3543999999999 24.4152999999998 1.40140000000008
42.3630999999998 16.7702999999998 5.8395000000001
39.0996999999998 20.7975999999998 -1.04859999999991
-0.0951999999995316 -0.0278999999998479 -0.0216999999999598
0.255200000000463 -2.07379999999985 -0.53309999999996
9.6833000000004 0.0757000000001571 5.70020000000002
14.6711000000003 1.05030000000011 2.58180000000002
14.6166000000003 -1.02759999999989 1.73030000000004
41.3323999999998 6.80069999999984 0.65040000000008
42.3339999999997 11.8603999999998 -1.22749999999991
ID=CYCdidUNKCOSUSNM011377

LM3=54
2.05030000000073 0.577300000000546 -1.62560000000009
2.19040000000052 -1.39149999999951 -2.18919999999995
11.6673000000007 0.694200000000445 0.882399999999975
13.4284000000007 1.06750000000044 0.635599999999968
11.3320000000007 4.20580000000045 -0.50550000000006
16.4532000000005 2.86210000000037 0.802699999999974
17.7631000000005 4.07300000000034 0.316899999999978
19.1453000000005 2.06750000000038 9.12089999999997
18.6340000000004 6.83630000000031 -1.21040000000002
22.7562000000002 6.23420000000022 0.540499999999974

23.6035000000002 6.69370000000019 -0.915200000000019
27.9130000000001 0.142000000000202 -0.348100000000009
29.5103 6.93220000000011 -4.23860000000001
33.3963999999999 5.3083000000001 -4.36580000000001
37.9159999999999 10.2534 -4.23840000000001
39.7553999999999 11.2038 -3.62350000000001
38.1550999999999 14.7393 -5.06700000000003
41.9835 16.1189 -1.17280000000004
42.302 10.4938 -3.41990000000001
40.7255999999999 12.4448 -4.95700000000001
1.70750000000075 1.01360000000053 -1.99800000000006
0.668200000000767 2.34840000000055 -2.60570000000005
9.09400000000063 7.13910000000045 0.523199999999963
10.6390000000006 7.78090000000044 0.741299999999967
14.1083000000005 8.50760000000039 0.692499999999974
15.3109000000005 8.71840000000036 0.480999999999974
15.5895000000005 11.3137000000004 8.81799999999995
20.9191000000003 10.1526000000002 0.169899999999978
21.3282000000002 11.3783000000002 -0.883100000000025
20.4675000000003 18.1659000000003 -0.202400000000058
25.7991000000001 14.6693000000001 -3.90320000000004
27.4529000000001 18.2590000000001 -4.06690000000006
34.9193 18.1747 -3.61480000000005
37.1353 18.7583 -3.64920000000005
37.9458999999999 20.8428 -3.11930000000005
38.6383999999999 18.7537 -4.68770000000004
-0.130999999999182 -0.191499999999435 -0.180700000000069
-1.05179999999918 1.20930000000057 -0.737800000000072
7.88320000000067 5.98540000000048 4.64749999999995
11.3296000000006 8.77940000000043 2.75739999999996
10.3633000000006 10.3116000000005 0.873099999999967
9.46060000000071 3.29060000000049 6.43979999999994
26.2082000000003 10.1854000000003 12.8598
34.2725000000002 13.2556000000002 10.8414
34.6995 23.0790000000001 -0.432000000000051
42.6529 16.5708000000001 3.54659999999997
38.5639999999999 20.2389 -2.36720000000005
-0.130999999999182 -0.191499999999435 -0.180700000000069
0.534800000000818 -1.98409999999943 -0.460000000000064
9.9395000000007 0.60570000000048 4.84259999999995
14.4793000000006 0.624100000000413 2.36769999999997
14.7599000000006 -0.937499999999564 0.985699999999976
41.3494 6.50930000000007 -0.0206000000000054
42.2649 11.4972 -2.0665
ID=CYCdidUNKCOSUSNM012097

LM3=54
2.8732 0.2359 -1.5263
3.1816 -1.4185 -1.7772
13.5322 1.1777 0.494
14.6514 1.4618 0.6913
13.4864 5.2341 -0.4132
17.6165 3.5448 0.9817
19.2945 4.5414 0.629

21.3699 2.9256 10.851
21.133 8.3813 -0.639
24.6129 6.7562 0.6848
26.2611 7.1773 -0.7942
30.7066 -0.0017 -0.1186
32.1808 7.3105 -3.8096
35.8189 6.1088 -3.6615
40.7463 10.1972 -3.5055
42.1821 12.0611 -3.3128
39.9325 15.779 -4.8412
44.0233 17.9166 -0.5726
44.7162 11.7982 -2.5674
43.1799 13.8305 -4.4536
2.7274 0.9288 -1.7925
1.8103 2.3938 -2.3795
10.5731 8.1955 0.1482
11.6337 8.7653 0.3237
15.1278 9.7749 0.3845
17.3814 9.5335 0.3285
17.3644 11.8142 10.2997
22.8152 11.5235 0.1547
23.506 12.1159 -1.0453
21.968 20.2593 -0.518
28.0974 16.2312 -3.5486
28.9464 20.0037 -3.2977
36.1472 19.6952 -4.0856
38.2303 19.8591 -3.4478
39.8546 22.141 -2.8573
40.5014 19.7344 -4.6859
0.0347 -0.2688 0.097
-0.6387 1.3086 -1.0311
9.1816 6.6821 5.9913
12.9677 9.7198 1.4514
11.6058 11.7566 0.3842
10.91 3.8361 7.6863
26.8034 10.3846 13.6214
37.0048 14.7448 11.2921
35.8959 24.9262 0.6618
44.2998 17.7554 5.0642
40.856 22.0549 -1.4596
0.0347 -0.2688 0.097
0.3936 -2.1484 -0.5436
11.9807 0.6974 6.2178
16.2374 0.863 2.7053
16.7068 -0.885 1.1512
43.4798 6.9435 0.8201
44.7099 12.0907 -2.0281
ID=CYCdidFEMMEXUSNM078111

LM3=54
2.4494 0.7022 -1.1331
2.3574 -1.4758 -1.7539
13.0046 1.8832 0.7254
13.6782 2.2009 0.7547

13.2332 5.5815 -0.3758
17.5913 4.8106 1.4059
18.772 5.5281 0.9593
20.1747 3.3136 10.9555
20.9382 8.9589 -1.0301
23.1943 7.5801 1.6209
26.1077 7.389 -0.4564
30.1298 0.4133 -0.0752
30.8507 8.6103 -3.945
34.7826 6.964 -4.3475
39.0122 12.059 -4.3626
41.0261 13.5239 -3.7733
38.5624 17.0727 -5.2967
43.4466 18.7501 -1.0923
43.2776 13.0711 -3.0175
42.1786 14.7495 -5.1812
2.1486 1.4329 -1.1541
0.8045 2.8874 -1.6919
10.0459 8.499 0.8806
10.8962 8.8202 0.87
15.1968 9.7414 1.3891
17.0369 9.9241 0.9835
16.518 11.9431 11.0716
21.3854 12.0971 1.3032
23.0074 14.2398 -0.2656
21.0007 21.8346 -0.3432
27.1767 17.2072 -3.6632
27.972 21.1519 -3.9602
35.2836 20.8346 -3.5028
37.8484 20.9866 -4.1527
39.0872 23.2492 -3.0605
39.6454 21.1094 -4.895
0.2823 0.017 0.3597
-0.5538 1.203 -0.2134
8.6081 7.2143 6.354
12.2464 9.5631 2.5238
10.5999 11.4677 1.3108
9.9564 3.9204 7.8705
27.8229 11.564 13.5607
36.3076 15.1122 11.0445
36.2672 25.9519 1.1654
43.3618 18.0808 5.0021
39.6949 22.7102 -2.2563
0.2823 0.017 0.3597
0.597 -2.0476 -0.901
11.1704 0.2628 5.5289
15.7744 2.1105 2.5494
15.6316 -0.0182 1.4428
42.8255 7.7137 0.0551
43.8131 14.5161 -1.9597
ID=CYCdidMALMEXUSNM100040

LM3=54
2.4917 0.8299 -2.1201

2.5274 -1.4226 -2.6888
13.3275 1.2837 0.3446
14.8269 2.2191 0.2037
13.1314 5.1477 -0.9328
17.8016 4.2095 1.0576
19.0794 5.3104 0.7437
21.2652 3.0358 11.1588
21.1796 9.0098 -0.8235
24.1457 7.5944 1.3505
26.3746 6.9991 -0.3753
30.0559 0.349 0.1484
32.4827 7.764 -3.2174
35.92 5.482 -3.3323
40.3222 11.2223 -3.0879
42.7043 12.6359 -2.9701
40.9285 16.3049 -4.878
44.81 18.0239 -0.2518
45.145 12.5611 -1.4585
44.0681 14.2227 -3.9959
2.2505 1.434 -2.0306
0.966 2.7492 -2.9023
10.0824 8.4059 0.1284
11.1836 8.8094 0.1306
14.7465 9.6776 0.7725
17.3202 9.7088 0.4413
17.0786 12.024 11.0211
22.3232 11.4526 1.398
23.9186 13.1838 -0.3343
21.7256 21.0482 -0.1757
28.5492 16.5788 -3.0683
29.4191 20.765 -3.341
36.6633 19.8393 -3.3489
39.453 20.6467 -3.4624
40.437 22.5452 -1.8533
41.3113 20.2613 -4.1497
0.0894 0.0585 -0.0387
-0.9867 1.7526 -1.1567
8.2197 7.3607 5.5786
12.6456 10.1191 3.0862
11.6333 11.6497 0.121
9.2434 3.853 7.2475
27.8199 10.512 14.1005
37.8463 14.8816 11.494
37.3893 25.5182 0.9005
44.4659 17.8738 6.394
41.6144 22.2358 -0.8
0.0894 0.0585 -0.0387
0.4307 -2.3633 -0.9726
12.2003 -0.102 5.7173
15.3027 0.3446 2.213
16.4897 -0.0759 0.1569
43.8518 7.3356 0.1753
45.3787 14.0105 -0.3641
ID=CYCdidMALMEXUSNM100172

LM3=54
2.13660000000056 0.840800000000453 -1.1519999999999
2.0984000000005 -0.865699999999572 -2.05699999999997
12.8749000000004 0.613400000000314 1.59700000000006
14.4523000000004 1.53430000000032 1.74540000000006
12.5553000000004 4.9854000000003 0.415100000000052
17.0641000000003 3.92450000000023 1.93230000000005
18.1168000000003 4.52610000000023 1.58520000000005
20.3769000000004 2.29670000000026 11.0114000000001
20.1995000000002 7.84310000000019 -0.0335999999999438
23.3838000000003 6.43360000000017 1.67480000000005
25.3526000000003 6.44230000000017 -0.28909999999994
28.8826000000004 0.260200000000256 -0.147199999999877
29.6725000000003 7.48240000000018 -3.17669999999992
33.8415000000003 5.47780000000021 -3.7165999999999
38.9353000000003 10.9413000000002 -3.9414999999999
40.8337000000003 12.2707000000002 -3.71519999999987
38.4672000000003 15.6624000000002 -5.58129999999991
42.6920000000003 17.4362000000002 -1.04119999999988
43.1683000000003 11.8434000000002 -3.48689999999986
41.5611000000003 13.8953000000002 -4.89619999999986
1.44330000000052 1.45870000000042 -0.953899999999865
0.579600000000548 2.79370000000044 -1.30599999999988
9.76470000000037 7.96660000000029 1.57890000000004
11.0507000000003 8.48920000000028 1.35460000000004
15.1066000000003 8.99020000000023 1.67150000000004
16.3803000000003 8.64210000000021 1.43450000000005
16.9465000000002 11.5213000000002 11.4927
21.2243000000002 11.0939000000001 1.76890000000006
22.2731000000002 12.5796000000001 -0.29379999999994
20.6302000000003 20.0066000000002 0.169800000000059
26.2684000000002 15.2539000000001 -3.32469999999992
27.7340000000003 19.6588000000001 -3.69389999999991
35.1263000000003 19.3403000000001 -3.9481999999999
37.4115000000003 19.6329000000001 -3.9643999999999
38.9476000000003 21.3620000000002 -2.81999999999989
39.6892000000004 19.8451000000002 -4.75139999999988
0.0969000000005744 0.200200000000448 0.632000000000124
-0.990299999999438 1.87040000000043 0.0769000000001012
7.82890000000036 6.32670000000029 6.98020000000004
11.8680000000003 8.96120000000024 3.52100000000003
10.7080000000003 10.9557000000002 1.23530000000004
9.27200000000038 3.2404000000003 9.36540000000004
26.8826000000003 10.3798000000002 13.8742000000001
36.3440000000003 14.5759000000002 10.7468000000001
36.0462000000004 24.1600000000001 0.0429000000001048
43.3488000000004 16.9915000000002 4.10020000000012
39.3076000000003 20.9858000000002 -2.47149999999989
0.0969000000005744 0.200200000000448 0.632000000000124
0.175000000000669 -1.78349999999951 -0.161599999999893
10.8081417454544 0.183478015848933 7.48613488340843
14.6733000000004 0.98310000000031 3.01320000000005
15.7379000000004 -0.752199999999703 1.28610000000008

42.9450000000003 7.34880000000025 0.288600000000144
43.4040000000003 12.1321000000002 -1.95909999999986
ID=CYCdidMALMEXUSNM100173

LM3=54
2.3858 0.7871 -1.0837
2.4937 -0.7165 -1.9231
11.7134 1.0016 1.2153
12.2896 1.6676 1.1913
11.8577 4.6888 0.0336
15.661 3.2732 1.8386
17.0247 4.1644 1.6115
18.2554 2.3383 10.6883
19.255 7.1615 0.0933
22.1277 6.195 1.906
24.0555 5.9774 0.379
27.981 -0.0342 0.2877
29.4409 6.1845 -2.591
33.4932 4.8235 -2.7779
38.4334 9.2824 -2.518
40.7147 10.7487 -2.1829
38.4928 14.0079 -3.75
42.856 15.846 0.43
42.8934 10.5819 -0.9367
41.2926 11.9255 -3.2212
2.3094 1.0786 -1.1313
1.7384 2.5634 -1.8679
9.2096 6.6184 1.3449
10.5951 7.3944 1.5092
13.7664 7.9814 2.0808
15.5236 8.128 1.79
14.939 10.6945 10.4943
20.5926 10.0335 1.9937
21.8441 11.0005 0.5681
20.9589 18.3878 0.2325
26.4683 13.8218 -2.4492
28.1703 17.7638 -2.9151
35.2107 17.6958 -2.6345
38.1546 17.9107 -2.1903
39.2856 19.5474 -1.0851
39.2902 17.7627 -3.1232
-0.0531 0.0872 -0.1694
-0.6522 1.7482 -0.5942
7.0971 6.4743 5.7515
11.5297 8.8104 3.3018
10.5371 9.6949 2.1233
8.6084 3.2797 7.5628
26.3246 9.7224 14.0241
35.7089 13.615 11.1731
35.8262 22.4854 0.7064
42.6065 16.0925 5.4347
39.369 19.8376 -1.0323
-0.0531 0.0872 -0.1694
0.4319 -1.724 -0.9452

9.4727 -0.181 5.4835
14.1141 0.9422 3.1274
14.2607 -0.3581 1.7034
41.5243 6.8891 0.3761
42.8892 11.7956 -0.1735
ID=CYCdidFEMHONUSNM148761

LM3=54
2.10300000000086 0.534500000000319 -1.21160000000006
2.46639999999956 -1.26840000000009 -2.00719999999989
12.2544999999996 0.618099999999938 0.692000000000094
14.3880000000002 1.02380000000008 0.351599999999924
11.2428 4.4308000000001 -0.52860000000003
16.3771000000002 3.1980000000001 0.602499999999946
19.0507 5.22740000000004 -0.351700000000047
20.4776 2.89280000000003 10.0414
19.4119999999998 7.4214999999999 -1.16889999999999
23.0076999999995 6.58949999999984 0.676500000000058
24.2852999999997 5.50459999999988 -1.08929999999996
28.4519999999997 -0.320100000000083 -0.121099999999943
29.5606999999997 6.47109999999989 -4.64239999999996
34.0228999999997 5.3931999999999 -5.42299999999995
37.5749999999998 10.3857999999999 -5.81579999999996
39.3477999999998 11.2574999999999 -5.26039999999996
37.1891999999999 14.4877999999999 -6.81679999999997
41.9324999999999 16.4906999999999 -3.36359999999997
40.9012999999999 11.3179999999999 -5.34039999999996
40.2151999999999 12.7434999999999 -7.11539999999996
2.12580000000072 0.876000000000291 -0.999700000000022
0.994800000000514 2.05140000000022 -1.8024
9.56290000000018 7.39760000000011 0.747299999999978
10.9430000000001 8.33280000000011 0.779199999999974
13.5447000000001 8.73630000000006 1.00009999999997
16.9496999999999 9.2717 -0.43270000000002
16.9014000000001 11.9420000000001 9.90399999999997
20.3235999999998 10.0368 0.7093
20.6807999999998 11.0950999999999 -0.904199999999994
19.3438999999999 18.7465 0.463099999999994
25.0469999999998 14.7068999999999 -4.30929999999999
26.8385999999999 18.5136999999999 -5.07219999999999
33.4050999999999 18.1251999999999 -5.58689999999999
35.3555999999999 18.1712999999999 -5.51759999999999
36.7784999999999 20.0987999999999 -5.75469999999998
37.1879999999999 17.7724999999999 -6.91399999999998
0.115300000000261 0.388600000000151 0.165400000000033
-1.00179999999962 1.80310000000017 -0.947699999999979
7.9002000000002 6.31370000000014 5.79209999999999
11.9690000000001 9.15160000000012 2.71929999999997
10.9527000000002 11.0790000000001 0.556999999999969
9.82500000000007 4.15280000000009 7.60040000000001
28.3872999999999 11.443 11.7762
36.5029999999999 14.5199999999999 8.26770000000003
35.9928 23.2530999999999 -1.80389999999999
43.4651999999999 17.0151999999999 1.25160000000003

39.8361999999999 19.4949999999999 -5.03879999999998
0.115300000000261 0.388600000000151 0.165400000000033
0.668999999999833 -1.61829999999998 -0.750699999999919
10.6502999999999 0.699400000000023 6.25810000000004
14.6574 0.927600000000053 3.0879
15.8288999999998 -0.81180000000002 0.459700000000064
42.1320999999998 8.04399999999987 -2.10859999999995
42.2018999999999 12.7509999999999 -4.70139999999996
ID=CYCdidFEMPANUSNM248343

LM3=54
1.94839999999881 0.451099999999755 -1.77299999999993
2.00099999999854 -1.73810000000033 -2.10259999999983
11.4471999999991 0.905499999999789 0.722500000000074
14.0379999999991 1.87109999999978 0.736300000000065
11.849799999999 4.97069999999979 -0.471399999999932
17.3430999999993 3.41979999999982 1.15270000000005
19.0559999999993 5.0119999999998 0.465600000000043
20.0885999999991 2.18769999999977 9.36540000000007
18.9183999999994 7.68299999999983 -0.848499999999967
23.2871999999994 6.72359999999983 0.844200000000035
24.2761999999995 7.10729999999985 -1.02669999999999
28.6378999999994 -0.0678000000001561 -0.114399999999968
29.2054999999996 7.08249999999985 -3.52559999999999
32.9426999999995 5.69189999999984 -3.93249999999998
37.5699999999994 10.5006999999998 -4.02519999999997
39.7755999999994 11.4639999999998 -3.66779999999996
37.7329999999994 15.3693999999997 -5.17659999999996
41.8836999999993 17.3204999999997 -1.32079999999993
42.2125999999993 11.2550999999998 -2.82119999999995
41.8486999999993 13.0704999999997 -4.51939999999995
1.69059999999874 0.60699999999973 -1.61169999999993
0.39269999999873 2.36109999999973 -2.32579999999994
8.73249999999894 7.34239999999974 0.994800000000079
11.025099999999 8.69619999999976 0.748500000000074
14.2977999999992 9.75539999999979 1.08540000000006
16.8508999999993 9.76899999999982 0.440700000000054
16.815599999999 12.9618999999997 9.54730000000009
21.4826999999994 11.2294999999998 0.837100000000034
21.8167999999994 11.9950999999998 -0.820499999999971
19.7741999999992 19.7588999999997 -0.123799999999961
25.2581999999994 15.3362999999998 -3.34729999999998
27.3548999999993 19.3111999999998 -3.87469999999997
34.0013999999993 19.2842999999997 -4.30059999999997
36.2771999999993 19.7156999999997 -3.68379999999996
37.5268999999992 21.9758999999996 -3.48699999999995
38.4437999999992 20.0001999999997 -4.62039999999995
0.109699999998685 -0.0876000000002852 -0.0568999999999297
-1.04930000000135 1.31209999999971 -1.23309999999993
8.67089999999893 6.75299999999976 5.95390000000008
11.8868999999991 10.0022999999998 2.38200000000007
10.658099999999 10.8745999999997 1.15990000000007
9.64819999999893 4.08989999999976 7.06990000000007
26.4338999999991 10.4189999999997 13.4385000000001

35.1492999999991 14.1791999999997 11.3048000000001
34.5418999999991 24.8833999999996 -0.37319999999993
42.6679999999992 17.4241999999997 4.00360000000009
38.9476999999991 21.4993999999996 -2.06239999999993
0.109699999998685 -0.0876000000002852 -0.0568999999999297
0.522699999998643 -2.35540000000029 -0.960099999999912
11.204299999999 0.68699999999979 5.95160000000007
15.5671999999991 0.77449999999982 2.73360000000005
15.2981999999991 -0.359600000000208 1.55580000000005
41.8736999999993 6.62439999999977 -1.00179999999994
42.5730999999993 11.9661999999998 -1.81599999999995
ID=CYCdidFEMPANUSNM283876

LM3=54
2.1626 0.4617 -1.5134
2.4469 -1.2936 -2.7413
12.7693 0.6791 0.9788
14.9363 1.5521 0.5817
12.8641 5.0786 -0.3426
18.3563 3.768 0.755
20.0575 5.6558 0.7441
20.4983 2.738 10.2637
21.1675 8.3262 -1.1684
24.8202 7.4228 1.6051
25.8873 7.3312 -0.8151
29.7438 0.0243 -0.014
31.0052 7.6587 -3.6626
34.7681 6.0403 -4.0488
40.0979 10.717 -4.7402
42.1492 11.4807 -4.3431
40.8168 15.6965 -6.0543
44.6531 17.2863 -2.3419
44.7329 10.8281 -4.2877
43.9248 12.9342 -5.6618
2.509 0.9984 -1.7318
1.4659 2.6526 -2.7351
10.0172 7.6103 0.7351
12.2528 9.0063 0.7003
17.0191 9.4465 0.8653
18.5591 9.4165 0.4887
16.5781 11.6228 10.3304
23.0654 11.0408 1.4831
23.923 11.9933 -0.7545
22.4951 20.2753 -0.3281
28.7326 15.2336 -3.8981
30.2007 18.9071 -4.3432
36.9655 19.3145 -4.8315
39.4728 20.1856 -4.4215
39.985 22.2413 -4.0887
41.3356 20.4989 -5.5212
-0.0406 -0.024 0.0551
-1.1149 1.3527 -0.6796
8.8132 6.6072 6.3576
12.3763 9.7632 2.7353

12.0163 11.3195 0.4807
10.0068 3.5983 8.0045
29.6739 11.6545 13.5271
37.0075 14.7677 10.8747
36.5797 24.8321 -1.0286
44.7412 17.4047 1.5628
39.7032 22.2369 -3.6695
-0.0406 -0.024 0.0551
0.0136 -2.1777 -0.6555
10.9033 0.8029 6.7236
15.6207 0.7464 2.422
16.3257 -0.7264 0.6688
42.7385 6.8141 -1.2637
44.0712 11.6964 -3.7366
ID=CYCdidUNKPANUSNM292252

LM3=54
2.18299999999953 1.08009999999974 -1.49039999999989
2.07970000000068 -1.21829999999988 -2.22520000000004
12.6631 1.43199999999995 0.973200000000031
13.9206000000003 2.19680000000003 0.802999999999997
11.9121000000002 5.11810000000008 -0.360499999999982
16.8821 4.1492999999999 1.37640000000004
18.5168755199744 5.15870292004095 0.948710594811487
19.8741999999999 2.23889999999991 9.98800000000004
18.8780000000001 7.79459999999998 -0.432799999999997
23.1804999999997 7.33169999999988 1.15660000000003
24.7004000000001 7.31629999999995 -0.831400000000034
28.1910999999999 0.260899999999885 0.21219999999997
29.7041000000002 8.0724 -3.60410000000007
32.7027999999999 6.10199999999989 -4.34960000000005
36.7991999999998 10.7760999999998 -5.10630000000008
39.1023999999998 11.6166999999998 -5.00210000000008
36.8803999999999 15.0111999999999 -6.16520000000008
41.6525999999998 16.8780999999998 -3.43840000000008
41.1209999999998 11.0385999999999 -4.67290000000009
40.3705999999999 12.8151999999998 -6.42520000000009
2.00309999999957 1.23299999999974 -1.26339999999991
0.312199999999833 2.31919999999983 -2.06089999999994
9.76549999999995 8.25689999999993 1.00970000000003
11.5029 8.51789999999996 0.94220000000003
14.6994000000001 9.18599999999999 1.61830000000003
16.4467000000001 9.16339999999998 1.07650000000001
15.8805999999999 11.0673999999999 10.3527
21.6427999999999 11.2102999999999 0.896100000000014
22.8633999999999 12.3371999999999 -0.818300000000007
20.1294999999999 19.6092999999999 -0.184900000000013
27.3398 14.5720999999999 -3.81030000000005
27.3892 18.1885999999999 -4.70840000000005
33.6146999999999 17.6521999999999 -4.86020000000007
36.6187999999999 18.2488999999998 -4.01260000000007
37.7787999999999 20.6457999999999 -3.95280000000008
38.4896999999999 18.8762999999999 -5.70330000000009
0.17270000000001 0.0039999999998907 0.0635000000000453

-1.03510000000005 1.40909999999987 -0.731099999999952
8.05419999999987 7.0562999999999 5.84130000000007
13.2622 9.00819999999996 2.24950000000004
10.6906 10.8431999999999 0.844100000000032
9.48629999999984 3.46339999999988 7.72280000000007
26.8129999999999 10.5513999999999 13.2639
36.5152999999998 14.3136999999999 9.32459999999997
34.6550999999999 24.1741999999999 -1.72180000000006
43.0638999999998 16.5953999999998 1.58159999999993
38.7011999999999 20.3166999999999 -3.10170000000007
0.17270000000001 0.0039999999998907 0.0635000000000453
0.34890000000043 -1.71419999999998 -0.3597
10.8689999999998 0.0193999999998654 5.78830000000007
15.4765999999998 1.64699999999983 2.55210000000006
15.4776999999993 0.0128999999996732 0.959700000000114
41.8211999999999 7.33429999999984 -1.43380000000007
41.3983999999998 11.4715999999998 -4.06190000000008
ID=CYCdidFEMPANUSNM294075

LM3=54
2.034 0.5272 -1.08
1.5176 -1.6909 -1.6251
12.4414 0.8371 1.5377
14.4036 1.4998 1.4438
12.2589 4.6441 0.4903
17.7467 3.3701 1.8429
19.1383 4.6563 1.2576
20.1821 1.0731 10.7642
20.3244 7.682 -0.4714
24.48 7.0804 1.3379
26.0143 6.282 -0.0504
29.917 0.0411 -0.2093
31.8747 7.7153 -3.9029
35.0992 5.4146 -4.2113
40.107 10.966 -4.0769
42.8252 12.5206 -4.2477
40.0598 15.8403 -5.5568
45.1214 18.0093 -1.694
44.8876 12.0442 -3.7924
43.5562 14.0471 -5.3529
1.8226 0.9223 -1.0945
0.3905 2.3787 -1.7976
9.617 7.62 1.5657
11.5216 8.0919 1.1574
16.0177 8.8371 1.2399
17.5278 8.7198 0.7374
16.1325 12.1708 10.6246
22.6953 11.1369 1.414
23.1183 12.3097 -0.1755
21.4932 20.2354 -0.3158
28.4784 15.6084 -3.9066
28.9058 19.5051 -4.6651
36.1884 19.9169 -3.9876
39.0313 19.9899 -4.0627

39.7542 22.2079 -4.2937
40.778 19.9985 -5.5063
-0.0171 -8e-04 -0.0215
-0.6631 1.8191 -0.7234
8.77 6.843 6.1791
12.3052 9.6775 2.8046
11.5499 10.418 0.9153
10.0536 3.5706 8.0366
28.5555 10.6832 14.4469
38.3893 14.944 10.8138
37.9565 23.7542 -2.4671
46.2282 17.3059 2.6234
41.7589 21.4072 -2.6767
-0.0171 -8e-04 -0.0215
0.9568 -2.2203 -1.4511
11.7354 -0.2371 6.369
15.8787 0.1931 3.4533
16.1025 -0.5223 1.5966
44.7956 6.9838 -0.3739
45.7707 11.688 -2.5918
ID=CYCdidFEMPANUSNM297892

LM3=54
1.8365 0.7142 -1.6552
1.7586 -1.5527 -2.6097
11.5053 0.1901 2.1985
13.4698 0.9621 1.6372
12.217 4.74 -0.3023
16.5948 3.3395 1.4671
19.195 4.9266 1.0033
19.3202 1.9748 10.4117
19.6446 7.3828 -0.6764
23.3816 6.9098 1.9885
25.7169 6.6785 -0.4497
29.7062 -0.1108 0.3901
30.9663 7.0445 -3.6948
34.6258 5.0629 -4.1702
38.5733 10.2387 -3.5227
41.3655 12.0869 -3.0013
39.6486 15.5286 -4.7704
43.2498 17.6843 -0.5982
43.4747 11.2649 -1.9092
42.8974 12.6897 -3.9376
1.6746 1.061 -1.6562
0.1067 2.3251 -2.6137
8.1747 6.6248 1.1185
10.0453 7.829 1.6151
13.9838 8.8293 1.3147
18.3254 9.1536 0.4915
15.705 11.4845 10.3538
21.6943 10.7889 1.8132
23 12.3461 -0.3922
21.4454 19.8207 0.04
27.1481 15.6966 -3.7262

27.7299 19.3553 -4.0066
35.1255 19.3196 -3.3263
37.882 19.3504 -3.3988
38.692 21.8033 -2.6232
39.9216 20.3089 -4.1494
-0.0132 -0.0848 0.0054
-1.4663 1.3644 -1.2233
8.3855 6.627 6.3392
12.2401 9.1106 2.1878
9.9155 10.3187 1.7053
9.1698 3.3821 7.941
26.7463 10.5598 14.3824
35.2511 14.1056 12.1984
35.7895 24.4883 1.6071
43.6216 17.3368 6.0208
39.8734 21.6144 -1.2281
-0.0132 -0.0848 0.0054
-0.285 -1.9549 -1.2437
11.0606 0.0813 6.2797
15.4288 1.0727 2.4551
14.4961 -1.2265 2.2572
43.202 6.1731 2.1628
44.035 12.2864 -0.6019
ID=CYCdidUNKPANUSNM305592

LM3=54
2.4518 0.6627 -2.0964
2.2133 -1.2741 -2.3655
12.6681 0.7362 1.076
14.9402 1.6838 0.7929
12.1654 4.7232 -0.548
18.1028 3.4841 1.1257
19.3332 4.8665 1.0561
17.2926 1.282 11.0438
20.2179 7.3537 -0.2126
24.3435 7.0946 2.0284
26.1026 6.3872 -0.0096
30.4085 0.0837 -0.1354
32.2245 7.4876 -2.0939
36.0396 5.2382 -2.7975
40.4015 9.5938 -2.4622
42.4942 10.9358 -1.8805
40.9918 14.7312 -3.4924
44.9276 16.0926 0.6369
44.8154 10.5061 -1.505
44.1235 12.1485 -2.9533
1.8369 1.2996 -2.0857
0.4556 2.1452 -2.3394
10.0826 7.3618 1.1133
12.1868 8.477 0.9364
15.8745 9.1202 1.2226
15.0498 8.2357 1.0237
16.0452 11.0976 10.7213
22.9932 10.2598 1.6716

24.5033 11.7176 0.0089
23.3456 19.1838 -0.4455
29.6595 14.8518 -2.149
30.0905 18.6374 -3.0418
37.3463 18.269 -2.6106
39.6954 18.4241 -2.1064
40.5579 20.4143 -0.9916
41.6712 18.5932 -2.75
0.046 0.0172 0.3493
-0.8275 1.098 -0.5168
9.5303 6.8332 6.0931
12.6188 8.9946 2.9069
11.5601 10.3332 0.8045
10.2803 3.6919 8.2495
27.6712 10.0198 14.2269
36.8144 13.4121 12.2373
37.8267 23.3719 2.2996
45.3631 16.542 5.1196
41.9545 20.129 0.102
0.046 0.0172 0.3493
0.267 -1.5811 -0.2486
11.6153 0.9813 6.5957
12.1287 0.1753 3.3928
15.6349 -0.1274 0.9539
40.776 5.2048 2.7888
41.6944 9.707 0.3083
ID=CYCdidFEMPANUSNM314573

LM3=54
2.11459999999892 0.499900000000164 -1.22959999999999
2.72889999999868 -1.5160999999998 -2.17229999999997
12.9651999999991 0.92860000000012 1.13480000000004
14.3438999999991 1.53730000000008 1.13910000000003
12.1830999999991 5.08270000000009 -0.188699999999952
17.2222999999993 3.7025000000001 1.27710000000003
18.9597999999993 5.26560000000007 0.988500000000035
19.3381999999992 2.32230000000005 10.8002
20.1702999999994 8.03080000000007 -0.829099999999985
23.5614999999995 7.13020000000005 1.38590000000003
25.3371999999996 6.95020000000007 -0.689599999999983
29.2663999999995 0.111600000000072 -0.10829999999998
31.0584999999997 7.57550000000003 -3.88449999999999
34.9181999999996 5.9479 -3.76149999999999
38.7743999999995 10.3748999999999 -3.49949999999999
40.6524999999995 11.6329999999999 -3.24929999999997
39.2148999999995 15.4162999999998 -4.82079999999998
43.7440999999994 17.5126999999998 -0.279799999999955
43.5870999999995 10.6691999999999 -2.31469999999997
42.6998999999995 12.7372999999998 -4.24749999999997
1.79329999999883 0.748800000000113 -1.29989999999995
0.669799999998822 2.63550000000012 -2.34989999999996
9.98709999999903 7.62570000000005 0.883900000000048
11.4053999999991 8.22940000000006 1.09460000000004
14.6602999999993 9.10060000000006 1.39030000000004

18.1684999999994 9.59860000000007 0.68040000000003
15.2501999999992 11.4479 10.4848000000001
21.6501999999995 11.0360000000001 1.30760000000002
22.8862999999995 12.6411 -0.746199999999972
21.3736999999993 20.5146 -0.133399999999973
27.6790999999995 15.7435 -3.55259999999999
28.8738999999995 19.5684999999999 -3.88389999999999
35.1154999999994 19.1114999999998 -3.46589999999997
37.4624999999994 19.9625999999998 -3.04319999999997
38.7471999999993 22.1811999999997 -2.45679999999996
39.7967999999994 20.0437999999998 -4.21509999999997
0.17429999999877 0.032900000000111 0.389700000000038
-0.477200000001248 1.63330000000011 -0.410799999999955
8.53899999999903 7.48260000000008 5.87690000000006
11.9142999999992 9.25920000000005 2.85830000000005
10.7316999999991 10.5925 0.200500000000047
11.137099999999 4.64530000000009 8.54970000000005
26.4165999999993 10.239 14.5888000000001
35.2204999999993 14.0767999999999 12.3337000000001
35.1831999999993 24.8436999999998 0.132600000000056
43.8448999999993 17.9390999999998 5.18720000000006
39.6674999999993 22.3338999999997 -1.54349999999996
0.17429999999877 0.032900000000111 0.389700000000038
0.352199999998732 -1.82619999999989 -0.629999999999952
11.2427999999991 0.61470000000011 6.20690000000005
15.5553999999992 1.36780000000011 2.93410000000003
15.3608999999991 -0.227699999999902 0.852600000000036
42.8201999999994 6.51929999999991 0.46460000000003
43.8553999999995 11.2909999999999 -1.88569999999997
ID=CYCdidFEMPANUSNM314576

LM3=54
2.9816000000014 0.409600000000169 -1.5369000000001
2.30600000000133 -1.49979999999992 -2.25969999999994
12.4420000000012 0.392000000000195 0.913599999999948
13.8990000000011 1.15870000000016 0.920799999999937
12.6373000000011 4.35890000000015 -0.355300000000061
16.762300000001 3.32000000000016 1.23389999999995
18.0026000000009 3.88700000000013 1.00899999999996
17.953200000001 1.47150000000018 10.474
20.0066000000009 7.19320000000014 -0.317600000000046
22.4365000000007 6.01870000000012 2.42209999999996
24.8576000000008 5.59820000000014 0.875099999999955
29.2262000000008 0.136000000000158 -0.133500000000048
30.4893000000006 6.50710000000012 -2.16600000000004
33.8337000000006 4.06050000000013 -2.45870000000005
38.8589000000002 9.25850000000004 -1.36450000000004
40.0329000000001 10.9998 -0.810700000000041
38.7957999999999 14.0691999999999 -2.69350000000003
42.7905999999999 15.7325999999999 1.39889999999997
42.8178000000001 10.3967 -0.0932000000000375
41.2849 12.1707 -2.08410000000004
2.25260000000139 1.32200000000022 -1.53890000000007
0.73610000000147 2.67670000000018 -2.39360000000007

9.9197000000011 7.22500000000013 0.915499999999971
11.6789000000011 8.00730000000015 1.11009999999997
14.965100000001 7.95840000000015 1.60179999999997
16.0461000000009 7.87620000000014 1.31189999999996
15.413800000001 10.4208000000002 10.6453
20.8067000000007 9.61440000000012 2.15179999999996
22.3989000000007 10.9609000000001 0.473599999999965
21.5285000000006 18.3754 -0.300100000000028
27.4837000000004 14.3308 -2.56310000000003
28.1374000000003 17.9841 -2.87860000000003
35.2768 17.8710999999999 -1.34640000000003
37.5107999999999 17.9848999999999 -1.20120000000003
38.7719999999999 19.6374999999999 -1.10710000000003
38.9921999999999 17.7870999999999 -2.39510000000003
0.0596000000015011 -0.0128999999997881 0.287999999999927
-0.782499999998445 1.52090000000019 -0.720300000000071
7.68610000000121 5.64420000000017 5.40869999999997
11.1936000000011 8.70220000000015 2.73399999999997
10.872000000001 10.4288000000001 0.930999999999971
8.9024000000012 3.41620000000019 7.39989999999996
26.7028000000006 9.94180000000011 15.121
35.2643000000003 13.1097 12.9834999999999
35.4466000000001 22.7834999999999 2.11179999999997
43.1093 16.2326 5.80559999999995
39.7132999999999 19.2692999999999 0.572699999999969
0.0596000000015011 -0.0128999999997881 0.287999999999927
0.0469000000014902 -1.79879999999983 -0.582800000000072
9.40540000000117 0.0845000000001788 6.05139999999994
14.1209000000011 0.207400000000183 2.85059999999994
15.1805000000011 -1.37749999999986 1.20089999999995
41.7351000000003 5.48830000000007 2.32109999999995
42.9614000000001 11.5801 1.23559999999996
ID=CYCdidMALNICUSNM337712

LM3=54
3.33250000000034 0.865599999999649 -1.23780000000013
2.14580000000108 -2.03079999999999 -2.65360000000028
13.3812000000002 0.555799999999635 1.13799999999992
14.8139000000003 1.06519999999974 0.91359999999997
13.6399999999999 5.00049999999966 -0.786499999999994
19.3739000000001 3.66489999999971 1.21919999999999
20.5386999999999 4.45489999999966 0.751700000000023
23.9360000000002 2.96199999999975 10.15
22.2926000000002 8.55449999999987 -1.27330000000004
25.9752999999997 7.89129999999969 0.839500000000019
28.6432000000001 7.34459999999987 -1.33190000000002
31.5725000000002 0.046899999999845 -0.42260000000001
33.6678000000002 7.88729999999993 -4.4214
37.5147000000003 5.52499999999995 -5.18170000000002
43.0411000000003 11.343 -5.90860000000002
44.3420000000003 12.6913 -5.60260000000002
43.1305000000003 16.4548 -6.62640000000001
47.5528000000004 18.192 -3.46920000000004
46.9050000000003 11.6193 -5.37120000000003

46.0754000000002 14.0499 -6.76170000000002
2.93890000000031 1.34129999999971 -1.19350000000012
0.493800000000449 2.19029999999976 -2.47080000000015
9.81960000000001 7.69979999999968 1.03289999999993
11.6996 8.81309999999972 1.19099999999995
16.7771 9.90929999999974 1.64809999999997
18.3825000000001 9.97529999999979 1.19539999999997
20.0519000000001 13.2923999999997 10.2235
24.4486999999999 11.1408999999998 0.870100000000002
26.2386999999999 13.5929999999998 -1.076
23.6168 20.6029999999998 -0.170800000000016
30.3145999999999 16.7456999999999 -4.46419999999999
31.6328 20.6411999999999 -5.2407
39.7270000000001 20.6044 -5.5902
41.7616000000002 19.5892 -5.55440000000001
43.0808000000002 22.178 -4.99120000000001
43.6416000000003 20.3151 -6.65120000000002
0.141800000000652 -0.207500000000188 0.453799999999795
-1.06359999999941 1.31919999999979 -0.647100000000191
10.1859000000001 7.60909999999967 6.54219999999991
13.6044 10.9209999999997 3.08299999999995
11.8251 11.8492999999997 1.88019999999994
10.8672000000002 4.04629999999968 7.83609999999991
31.8988000000001 12.0499999999998 13.5111
41.7084000000001 15.5756999999998 10.5132
40.6526000000002 25.7266 -0.115000000000021
49.1205000000004 18.1363 2.99999999999995
44.3276000000002 21.8885 -3.86760000000003
0.141800000000652 -0.207500000000188 0.453799999999795
0.163200000000909 -2.01980000000009 -0.884400000000278
12.7748000000003 0.780199999999705 6.34369999999992
16.9758000000002 0.421899999999731 3.71849999999997
16.9662000000001 -1.15160000000027 1.37319999999997
47.7529000000004 7.32999999999996 1.40939999999997
47.6519000000004 12.335 -3.67170000000003
ID=CYCdidFEMPERUSNM364503

LM3=54
2.1586 0.5063 -1.2056
2.3376 -1.6082 -1.8587
12.8081 0.7736 1.2961
14.4293 1.7699 1.2754
12.1367 4.601 -0.3031
17.8357 3.5733 0.9952
18.8793 4.8977 1.0577
19.6539 2.5711 9.902
19.5589 7.9068 -0.777
23.3096 6.8307 1.2128
24.0066 6.5898 -1.1778
28.7306 -0.0323 -0.2958
29.7708 6.7596 -4.2239
33.5101 5.0097 -4.5058
38.6699 9.7997 -5.2306
40.5491 11.2343 -4.7557

38.7765 15.1896 -6.5479
42.6815 16.878 -2.9796
42.5109 10.3063 -4.7866
42.1879 12.2318 -6.0376
2.0875 1.1127 -1.4904
1.1159 2.2159 -2.0952
9.7072 7.8238 1.1155
11.9402 8.754 0.673
15.0542 9.4725 1.0209
16.6131 9.3803 1.1282
16.4363 12.6084 9.8322
21.5413 11.0332 0.8846
21.7036 11.5375 -0.8842
20.803 19.6637 -0.4096
26.3883 15.265 -4.1625
27.9472 19.1125 -4.7172
34.8255 19.0799 -5.1024
37.0127 19.0618 -4.9595
38.1144 21.3636 -4.7741
39.0092 19.5083 -6.3014
-0.0162 -0.0267 -0.3459
-0.6974 1.4075 -1.0539
8.6777 6.9567 6.4112
12.3667 9.7481 2.929
11.4402 10.6476 1.1851
10.6654 4.271 8.271
26.2854 10.8989 13.8193
36.7887 14.8984 10.5
36.2856 24.8436 0.0017
43.6889 17.1458 2.1403
39.5074 21.151 -2.7256
-0.0162 -0.0267 -0.3459
0.2753 -1.76 -1.0656
11.0826 1.1385 6.5761
15.4911 1.4404 3.0738
15.8111 -0.0443 1.5923
43.3164 6.2983 0.0467
43.3697 12.2161 -2.1965
ID=CYCdidFEMPANUSNM460158

LM3=54
2.5067 0.929 -1.1679
1.9746 -0.9009 -2.3792
12.0507 0.9319 0.3564
14.9956 1.2992 0.5323
12.7353 4.9937 -0.6957
17.6204 3.4553 0.7512
19.8235 5.6067 -0.1468
19.6345 1.7183 9.1917
18.7534 6.9991 -0.9109
23.6297 7.7566 0.9239
24.9534 7.1099 -0.9908
28.8457 0.4564 -0.1537
30.9772 7.3122 -3.8944

34.4913 5.5189 -4.5481
38.7559 10.1126 -4.0752
40.547 11.3499 -3.6888
38.9597 14.853 -4.8848
43.1895 16.301 0.0504
43.1294 10.1113 -2.6421
42.3787 12.5559 -4.5232
2.2471 1.6763 -0.9651
0.6226 2.582 -2.0561
9.2016 7.75 0.4404
11.7796 9.0679 0.7519
14.7965 9.3829 0.9406
17.0496 9.5326 0.6955
16.2762 10.5589 9.7658
22.266 10.6724 1.1716
23.2425 11.7407 -0.3424
21.9885 19.4209 0.0836
27.8979 15.6919 -3.6719
29.1712 18.953 -4.1062
35.6989 18.208 -3.9407
37.8165 18.2412 -3.6017
38.6076 21.1308 -3.0689
39.8461 18.8007 -4.5276
-0.0773 0.0134 -0.1272
-0.9736 1.2529 -0.5244
9.4233 6.7795 5.6771
12.0328 9.6619 2.9017
11.3962 11.1532 1.4141
9.3203 3.1642 6.0156
28.6508 10.0263 13.4472
36.6718 13.2423 10.3079
36.1135 23.695 1.0816
43.9574 16.0665 4.4951
40.4447 20.3604 -1.4432
-0.0773 0.0134 -0.1272
0.1145 -1.8223 -0.5445
11.7517 0.3113 5.3798
15.4161 0.1775 2.7959
15.9202 -0.4229 0.919
42.9956 5.4765 1.6018
43.5586 10.6996 -1.4998
ID=CYCdidFEMBRAUSNM545910

LM3=54
2.32250000000034 0.187399999999904 -2.38169999999991
2.01140000000032 -2.06369999999974 -2.96220000000006
11.6314536017431 0.286205815499593 0.377825754703574
12.9917289101216 0.934292264376382 0.288807281908939
11.6177000000003 4.19860000000002 -0.881700000000057
16.0765999999998 3.48539999999986 0.660999999999966
16.5441000000003 3.73980000000001 0.623399999999989
19.5638000000003 1.31640000000001 10.2051
19.839 7.45319999999986 -0.575100000000014
22.454500000001 6.05500000000034 1.65309999999997

24.9851000000005 6.98680000000003 -0.606599999999998
28.7605000000006 0.0721000000001069 -0.388000000000016
29.9075000000004 7.0109 -3.37469999999999
33.6477000000004 5.85350000000005 -3.6377
38.1983000000002 10.7954 -2.75179999999999
39.8547000000003 12.1123 -2.085
38.4374000000004 15.6656 -3.49089999999999
42.2191000000004 17.0385 0.444900000000011
41.9989000000004 11.4797000000001 -1.49819999999999
41.3636000000004 13.5466000000001 -2.8082
2.10890000000031 0.811199999999927 -2.28799999999998
0.348200000000308 2.29559999999994 -3.23979999999999
8.85700000000032 6.83739999999996 0.127799999999994
9.98940000000033 7.41669999999997 0.376699999999989
13.5555000000002 8.15629999999994 0.777799999999977
15.1593000000002 8.49819999999992 0.541999999999982
15.1141000000003 10.8766 9.98479999999997
20.1537000000005 10.7743 1.27099999999999
21.9825000000005 12.1599 -0.781300000000007
20.3903000000005 19.256 -0.519899999999999
26.0362000000005 15.6008 -3.1892
27.5815000000005 19.3337 -3.4151
34.7838000000005 19.0036 -2.75129999999999
36.9485000000004 19.2465 -2.3357
37.5223000000003 21.3482 -1.4508
38.8740000000004 19.1916 -2.92359999999999
0.0675000000002583 -0.103000000000017 0.185999999999972
-1.02269999999971 1.03199999999996 -1.22550000000001
7.36360000000029 5.77289999999997 5.32089999999998
10.7644000000003 8.45489999999994 1.93879999999999
10.1959000000003 9.98949999999993 0.70469999999999
8.43010000000028 2.75839999999997 7.62309999999998
26.3804000000005 10.0941000000001 13.4348
34.8701000000005 13.7006000000001 11.4327
35.9071000000005 23.6513 1.73460000000001
42.3508000000004 16.7559 5.1736
39.1267000000004 20.8278 0.0784000000000061
0.0675000000002583 -0.103000000000017 0.185999999999972
0.0337000000002217 -2.00309999999998 -1.19670000000005
10.0385000000002 -0.606800000000012 5.54489999999996
14.2781000000001 -0.125800000000016 2.34559999999995
14.5734000000003 -0.591599999999958 0.885199999999975
41.9643000000004 7.09780000000007 2.15639999999998
42.1538000000004 11.5539000000001 0.0784000000000036
ID=CYCdidFEMCOLUSNM554227

LM3=54
1.72350000000031 0.601399999999765 -1.73240000000013
1.91310000000055 -0.988500000000095 -2.37360000000017
12.1147000000003 0.839399999999868 1.0972999999999
13.3305000000003 1.48909999999986 1.17239999999989
11.7820000000003 4.66879999999989 -0.425000000000095
16.2985000000002 3.54579999999988 1.11779999999989
17.0849000000001 4.40149999999988 0.841799999999879

19.7052 2.27789999999981 10.6366999999999
19.8115000000001 7.77639999999993 -0.496800000000115
22.55 7.0257999999999 1.56659999999986
24.1021999999999 6.33549999999989 -0.0683000000001588
28.5979999999998 -0.0620000000002088 -0.0589000000001701
29.4544999999996 7.02959999999981 -3.27590000000018
33.8464999999994 5.67789999999978 -3.72900000000019
38.1303999999992 9.99779999999974 -3.60880000000022
40.3227999999992 11.5881999999997 -3.04100000000023
38.2141999999993 15.1553999999998 -4.92950000000023
42.7973999999991 16.8020999999997 -0.854600000000252
42.5509999999991 11.1273999999997 -3.06820000000024
41.4129999999992 12.8647999999997 -4.48230000000024
1.52230000000029 1.26289999999977 -1.69650000000014
0.747200000000302 2.61299999999979 -2.40020000000014
9.2341000000003 7.35299999999989 0.680399999999912
10.6570000000003 8.07179999999991 0.777499999999906
13.9609000000002 8.76579999999993 1.5088999999999
15.6070000000001 8.73389999999991 1.17879999999989
15.9282 11.0631999999998 10.9360999999999
21.0991 10.7018999999999 1.48899999999986
21.6355999999999 11.9572999999999 0.227899999999853
20.5091999999998 19.4620999999999 0.0679999999998372
25.7525999999996 14.9627999999998 -3.2756000000002
28.2471999999995 19.1725999999998 -3.48460000000021
35.0080999999994 18.7806999999998 -3.78830000000023
36.8149999999993 19.2535999999998 -3.29820000000024
37.9247999999993 21.1466999999998 -3.55040000000026
38.7639999999993 18.8310999999998 -4.74670000000025
0.0748000000002758 0.0111999999997677 0.191799999999858
-0.944599999999719 1.38329999999972 -0.770800000000161
8.28400000000027 7.03489999999986 5.88629999999992
11.7805000000002 8.81919999999991 2.24089999999992
10.7198000000002 10.5405999999999 0.599899999999905
9.27820000000027 3.48989999999985 7.69329999999992
27.4191999999997 10.6488999999998 13.8587999999998
36.1159999999994 13.9058999999997 11.6714999999998
35.5125999999993 23.8461999999997 0.22189999999975
43.1603999999991 16.8298999999997 5.04919999999975
39.9051999999992 20.5420999999998 -1.51740000000026
0.0748000000002758 0.0111999999997677 0.191799999999858
-0.0207999999997192 -1.82810000000024 -0.668700000000136
11.1093000000003 -0.405800000000173 5.70049999999991
14.7568000000003 0.66199999999987 3.5162999999999
15.2094000000002 -0.376400000000142 0.944699999999894
42.3686999999992 7.14049999999968 0.897599999999773
42.8557999999991 12.3684999999997 -1.10680000000025
ID=CYCdidFEMBELUSNM583067

LM3=54
2.51929999999964 0.597800000000466 -1.87210000000004
1.2912999999987 -1.57529999999981 -2.88370000000001
12.5943999999997 0.86740000000026 0.809199999999998
13.9702999999999 1.22330000000034 0.383099999999951

12.4304 4.73920000000034 -0.482000000000058
18.4817000000001 3.87980000000037 0.229299999999933
20.9664999999998 5.74690000000021 -0.134900000000065
20.7004999999998 2.39490000000029 9.62589999999997
21.6396999999996 8.12700000000012 -0.901499999999965
25.6849 8.2082000000002 1.01169999999999
27.2947999999997 7.41790000000011 -0.907999999999983
30.136 -0.0745999999998406 0.132299999999993
31.9979999999998 7.20880000000006 -3.08899999999998
35.6014999999998 5.1838000000001 -3.90669999999998
40.5013999999999 10.8299000000001 -3.23889999999995
42.5316 10.3571000000002 -3.27399999999996
40.5592 15.6069000000001 -4.12799999999997
44.8725725892806 17.3751604590423 0.354052233405267
44.6943999999999 11.1940000000002 -2.47579999999997
45.0307 13.2558000000002 -3.74339999999997
2.61042999559441 1.0577303739487 -1.5788076441971
2.21869999999955 1.38370000000038 -1.54690000000004
11.0083999999999 8.79030000000037 0.650799999999961
10.8986999999999 8.70630000000034 0.463899999999959
15.7952999999999 9.35450000000026 0.519199999999978
19.3603999999998 9.1595000000002 -0.229800000000005
17.9821999999999 11.5644000000003 10.0724
24.4245999999999 10.6163000000002 1.02210000000001
24.896 12.8457000000002 -1.11419999999999
22.1324 19.7012000000002 -0.267799999999995
28.4190000000001 15.7024000000002 -3.31359999999999
29.6003 19.7255000000001 -3.65499999999997
36.9792000000001 18.6565000000002 -3.45609999999997
38.3182 20.0773000000002 -3.57719999999996
39.8196 21.4304000000002 -2.53229999999996
42.021 20.1486000000001 -4.07219999999994
-0.00100000000061617 -0.0853999999996617 -0.322900000000037
-1.49140000000058 1.19530000000035 -1.31770000000003
9.1208999999998 5.75160000000037 5.80619999999997
12.9306999999999 9.09260000000031 2.25939999999997
11.1960999999999 10.7953000000003 1.20279999999997
9.68689999999973 2.46970000000034 7.09099999999996
29.2929 10.3918000000003 12.9758
35.8344000000001 13.3498000000003 11.8177
38.5473 24.6941000000002 0.77700000000003
44.7582000000001 16.9472000000003 5.90860000000002
41.1609 21.3881000000002 -1.04579999999995
-0.00100000000061617 -0.0853999999996617 -0.322900000000037
0.170299999999151 -2.49649999999977 -1.98640000000002
11.0753999999997 0.642500000000304 5.73269999999997
15.8069999999999 0.858800000000328 2.38719999999996
15.9798999999998 -0.456399999999681 0.786899999999964
45.5520999999999 7.5288000000002 0.362900000000026
45.0378 12.2266000000002 -0.889299999999969
ID=CYCdidFEMBRAMVZB121210

LM3=54
2.1697 0.7219 -2.0746

1.5564 -1.3788 -2.5953
12.2721 0.4388 1.3976
14.1199 1.0167 0.922
12.0847 4.2869 -0.0613
16.9896 2.6691 1.0138
19.1247 4.3829 0.5226
20.6091 2.7779 10.1766
20.3731 7.3514 -0.5102
23.9198 6.6994 1.8523
25.6495 6.778 -0.1639
29.4193 0.0485 0.1212
31.0519 7.1528 -3.0882
34.4405 4.6322 -4.0107
40.1172 9.9355 -4.3299
41.6809 11.1051 -4.0362
39.6145 15.0731 -5.4722
44.6588 17.0498 -2.2933
43.7496 10.8902 -3.2265
43.8803 12.9715 -5.295
2.3406 0.8624 -1.8467
0.0149 1.8817 -2.7662
9.5606 7.3343 1.3444
11.1844 8.2111 1.0901
14.5489 8.6744 1.0923
16.8799 8.6195 0.6789
16.6825 10.8783 10.0229
22.4918 10.035 1.6269
23.6533 11.6061 -0.8022
21.9648 19.1927 0.2864
27.9049 14.6961 -2.8799
27.9899 18.9737 -3.7438
36.5158 18.608 -4.6356
38.6772 19.0974 -3.9493
39.4461 20.9458 -3.5879
41.2274 19.469 -5.0228
0.0569 -0.0233 -0.038
-1.1429 1.189 -1.6892
8.5747 6.5373 6.094
11.6696 9.1453 3.2795
11.0503 9.9108 1.4405
8.3391 2.9861 7.0624
28.4815 10.4178 12.5748
37.5984 14.0314 9.6632
37.0351 23.5912 -0.6601
45.769 16.6688 2.0433
41.6099 20.0759 -2.8671
0.0569 -0.0233 -0.038
-0.105 -1.8253 -1.2705
10.3259 0.4007 6.2946
14.656 -0.0143 3.5962
15.2987 -0.5991 1.5237
43.5181 6.5379 -0.2315
44.1267 11.6085 -2.6924
ID=CYCdidFEMPERMVZB157801

LM3=54
2.38589999999916 0.522499999999668 -2.12249999999987
1.13309999999899 -1.96440000000046 -2.90519999999993
11.3415999999992 0.673099999999703 1.18300000000009
11.8155999999992 1.4389999999997 0.743500000000086
11.6036999999993 5.35159999999974 -0.74239999999991
16.7460999999993 3.59879999999979 1.12010000000008
19.1737999999994 5.4741999999998 0.513300000000069
20.0530999999993 3.34599999999973 9.9751000000001
18.8304999999994 8.06359999999984 -0.54379999999995
23.1723999999995 8.21909999999984 1.42010000000005
24.9914999999995 6.98099999999984 0.00940000000003571
29.6475999999993 0.387399999999972 -0.0135999999999346
31.2168999999995 8.25559999999978 -2.85859999999996
35.3169999999994 5.94769999999973 -3.38149999999995
40.4984999999996 10.9973999999997 -2.95149999999997
41.7316999999997 11.0851999999997 -2.93159999999997
40.9706999999997 15.9562999999998 -3.81849999999999
44.6044999999998 17.8610999999997 0.78130000000002
44.4873999999997 11.7219999999997 -1.15129999999996
44.2230999999997 14.0892999999997 -3.10619999999998
1.49969999999903 1.11519999999959 -1.97729999999989
-0.998200000000945 1.96809999999959 -2.61249999999989
8.07889999999927 8.09639999999975 1.04570000000007
8.80239999999929 7.92399999999977 0.74200000000008
13.9430999999994 9.86779999999984 1.13310000000006
16.5431999999994 10.1589999999999 0.527500000000057
16.6831999999994 12.3700999999998 9.62960000000008
22.3114999999996 10.7054999999999 1.48780000000004
22.8511999999997 12.8349999999999 -0.210599999999968
21.9816999999997 20.8664999999999 0.155800000000034
28.3711999999997 16.0126999999999 -3.09719999999998
29.6823999999998 20.5518999999999 -3.67179999999999
36.7059999999998 19.9645999999998 -3.49819999999999
38.1560999999999 21.0953999999998 -3.3073
40.1712999999999 22.3268999999998 -2.0847
42.0752999999999 20.2733999999998 -2.8225
0.0866999999989315 -0.0766000000004445 -0.267799999999872
-1.93050000000103 0.604599999999567 -1.95739999999988
8.00669999999918 6.9588999999997 5.65260000000009
11.1601999999993 10.4224999999998 2.48180000000007
9.59459999999932 11.5491999999998 1.39470000000007
10.0067999999991 4.36809999999968 7.35200000000011
26.9649999999994 11.2167999999997 13.9376000000001
36.6850999999996 15.3167999999997 11.5303
38.6720999999998 25.3431999999998 2.11050000000003
45.3864999999996 17.9148999999997 5.64840000000003
42.1875999999999 21.6299999999998 -0.536399999999992
0.0866999999989315 -0.0766000000004445 -0.267799999999872
-0.891400000001033 -1.85940000000047 -2.10279999999988
10.8492999999992 1.18499999999967 5.75920000000012
14.9290999999992 0.675299999999723 2.8734000000001
14.6549999999991 -0.762200000000291 1.59890000000009

45.6388999999995 8.93389999999968 1.98140000000006
45.0900999999996 12.9916999999997 -0.0155999999999714
ID=CYCdidFEMBRAMVZB190355

LM3=54
2.4923 1.1498 -1.0124
2.2028 -0.8483 -1.9552
10.5858 0.5955 0.7212
12.1784 1.2126 0.8588
11.4985 4.6012 -0.0742
15.6504 3.0807 1.4207
17.7562 4.3496 1.1994
15.0141 1.0741 8.6691
17.884 6.5955 0.054
21.3838 6.5426 2.0535
24.0656 6.425 0.1209
27.8316 0.1071 -0.2767
29.2378 6.3764 -2.344
32.4806 4.0426 -2.5928
37.7339 8.9741 -3.0462
40.0744 9.9218 -3.1079
38.2826 14.4276 -4.4483
41.8779 15.5986 0.8323
41.4483 8.9885 -2.5078
41.9702 11.5208 -3.8225
2.6206 1.4798 -0.7861
1.0591 2.2539 -2.178
8.0484 6.6054 0.372
9.2466 7.2276 0.5557
13.7351 8.5034 1.2261
16.7571 8.3085 1.2524
12.4905 9.7793 8.5567
19.9979 9.7069 1.906
22.1206 11.2911 -0.2338
20.6307 17.7882 -0.0293
26.3454 13.8604 -2.5352
27.2575 18.0279 -2.8031
34.362 17.5875 -2.7712
36.1735 18.018 -3.1573
37.2415 20.1039 -2.5109
38.6859 17.9306 -4.4065
-0.035 0.1496 -0.0743
-0.8902 1.6286 -0.9999
6.9899 5.2681 5.6503
10.0134 8.427 2.8711
9.4384 9.0448 1.5946
6.7914 2.6798 6.1472
24.8713 9.8725 13.5526
33.5524 12.8572 12.1245
34.1608 23.3779 1.2242
42.1844 16.0798 5.6132
38.1862 20.3695 -1.5625
-0.035 0.1496 -0.0743
0.2011 -1.2567 -0.7228

9.046 0.7426 5.9708
12.99 0.7031 2.6067
13.3383 -0.7832 1.794
40.6597 4.8851 1.179
42.2223 10.3048 -1.531
ID=CYCdidFEMUNKUNKMfNB3317**

LM3=54
2.74220000000046 0.700400000000597 -1.35929999999998
1.42620000000047 -1.71699999999947 -2.53090000000012
13.1520000000002 -0.0520999999999603 1.76639999999999
15.2173000000002 0.996700000000363 1.23299999999997
13.3257000000002 4.82020000000039 0.336999999999966
18.6620000000002 3.49290000000034 1.70589999999995
20.5153000000002 4.85760000000032 1.40369999999994
20.2433000000002 1.5474000000004 9.74839999999996
20.9855 7.99590000000025 -0.0524000000000681
24.8580000000002 7.34390000000028 2.13139999999993
26.5849000000001 6.88110000000024 -0.189800000000072
29.9908000000002 0.0990000000003063 0.220099999999927
32.7444000000001 7.51100000000023 -3.53220000000008
36.4757000000001 5.91160000000025 -3.64360000000008
42.0875000000004 11.3186000000003 -4.2122000000001
43.8067000000004 12.9710000000003 -3.98060000000011
42.0175000000004 16.2827000000003 -4.48690000000011
45.7923000000005 17.8479000000003 -1.37110000000012
46.1880000000005 11.5368000000003 -3.16130000000011
46.6913000000005 14.0665000000003 -4.18070000000011
2.64100000000036 0.941000000000566 -1.35230000000001
0.00250000000040074 2.02270000000057 -2.29700000000003
9.8823000000002 7.4175000000004 1.95409999999998
12.3354000000002 8.78090000000037 1.52779999999998
16.3592000000001 8.99060000000032 1.70259999999996
18.5585107713496 9.04637546115286 1.24495967218519
16.9463000000002 11.1612000000004 10.3136999999999
23.631 10.0977000000002 1.71519999999993
24.7049 11.9661000000002 -0.576500000000076
22.7966000000001 18.9213000000003 -0.429500000000077
28.7091 15.5318000000002 -3.40980000000007
31.6527000000001 19.6522000000002 -4.1341000000001
38.8025000000004 19.9358000000003 -3.49380000000012
41.0293000000004 19.7739000000003 -3.52340000000012
42.2051000000004 22.3095000000003 -2.8233000000001
43.5341000000005 20.5354000000003 -3.9666000000001
0.00350000000039918 0.179700000000634 -0.108500000000005
-1.13159999999963 1.50870000000061 -0.851200000000015
9.46360000000025 6.44060000000044 7.47099999999999
13.0909000000002 9.82830000000035 3.50009999999997
12.0306000000002 10.8556000000004 2.81829999999997
8.95070000000025 2.82710000000045 7.54979999999998
32.6690000000003 11.6919000000004 11.9007999999999
39.1266000000004 14.5729000000004 9.4226999999999
39.1932000000005 24.3524000000004 -0.815100000000107
46.1089000000006 17.3279000000004 4.2985999999999

42.9053000000004 21.9474000000003 -1.9655000000001
0.00350000000039918 0.179700000000634 -0.108500000000005
-0.0497999999995893 -1.72129999999938 -1.22540000000001
11.6989000000002 0.306800000000439 6.91309999999999
16.3176000000002 -0.138299999999628 2.80619999999997
16.4660000000003 -0.933399999999581 2.44899999999997
45.9667000000004 8.92350000000036 -0.842700000000099
46.4485000000004 13.0079000000003 -1.86130000000011
ID=CYCdidFEMBRAAMAMfNB35827*

LM3=54
2.96609999999935 1.15699999999969 -1.27399999999987
1.73439999999888 -1.5835000000004 -1.86139999999982
12.7203999999995 0.634799999999785 1.39380000000007
15.5776999999996 1.00299999999978 1.46490000000006
12.7809999999996 4.96079999999983 -0.371799999999964
19.7104999999997 3.86179999999984 1.04200000000005
21.8942999999997 5.72039999999984 0.302100000000031
19.2638999999995 2.16399999999977 9.48670000000006
20.3297999999997 7.78399999999987 -0.644599999999977
25.1999999999997 7.17709999999984 1.20680000000001
26.0274999999998 6.49579999999985 -0.562500000000013
30.8731999999996 0.040999999999763 0.0505000000000002
31.7142999999997 6.63739999999979 -3.47890000000003
36.0293999999996 4.46089999999972 -3.53300000000002
40.3761999999994 9.72859999999963 -3.34870000000002
42.3413999999993 10.9527999999996 -3.53010000000004
41.4903999999993 14.8978999999996 -4.49860000000003
45.6955999999991 16.5440999999996 -0.519500000000023
44.9434999999992 9.5081999999996 -2.42600000000003
45.9465999999992 11.9043999999996 -3.19700000000003
3.43099999999932 1.11179999999971 -0.918699999999895
0.803499999999306 1.9146999999997 -1.8034999999999
9.99669999999943 7.32839999999978 1.50020000000008
12.3570999999995 8.94109999999981 1.37370000000008
16.4034999999996 9.39239999999985 1.25380000000006
19.3035999999997 9.58099999999987 0.537800000000051
16.7628999999994 11.0311999999998 9.83890000000007
23.7327999999997 10.9545999999999 1.38900000000001
24.0738999999997 12.0159999999999 -0.0483999999999931
23.6787999999995 19.6907999999998 -0.27309999999999
28.2587999999995 14.8759999999998 -3.79180000000002
30.4946999999995 19.0481999999998 -4.18770000000002
37.6538999999993 18.2989999999997 -3.76270000000005
39.8970999999992 18.0303999999996 -3.88900000000004
41.2686999999991 20.6892999999996 -2.80480000000005
43.0112999999992 19.5954999999996 -3.24340000000003
0.600099999999272 0.0127999999996798 0.492000000000091
-0.832000000000755 1.65309999999968 -0.772799999999896
10.2805999999994 6.5052999999998 6.90170000000009
12.6533999999995 10.0767999999998 2.64010000000007
11.7739999999994 10.8234999999998 2.73520000000007
11.1819999999994 3.79239999999978 7.59330000000007
28.1356999999994 10.6193999999997 13.5282000000001

37.2058999999992 13.7121999999997 11.3134
37.1779999999992 23.8976999999996 1.16139999999999
45.5855999999991 16.8194999999996 4.61799999999998
41.9053999999991 21.1810999999996 -0.969300000000025
0.600099999999272 0.0127999999996798 0.492000000000091
0.513199999999222 -2.41640000000033 -0.724699999999884
12.4638999999995 1.12559999999979 6.89630000000008
16.3888999999996 0.270899999999824 2.61460000000004
16.2421999999996 -0.879000000000209 2.67920000000004
42.8177999999993 5.86369999999959 -0.0387000000000177
45.4103999999992 11.3500999999996 -1.29140000000003
ID=CYCdidFEMBRAAMAMfNB38397*

LM3=54
2.0175999999994 0.332399999999992 -1.37730000000002
1.93749999999969 -1.91660000000002 -2.33970000000006
12.6296999999997 0.706799999999993 1.74949999999996
14.5846000000004 1.18230000000015 1.50739999999996
12.6079999999997 4.43439999999993 -0.260099999999947
17.6041000000005 2.45070000000016 1.8956
19.0333000000003 3.63850000000011 1.44469999999998
18.8937000000004 2.2161000000002 10.4666
20.2863999999999 7.11390000000001 0.359100000000013
25.0650000000003 6.55900000000014 1.89959999999997
26.1164000000001 6.44700000000002 0.431899999999987
30.5819000000007 0.193100000000228 -0.143000000000027
31.9858000000006 6.75790000000016 -2.47690000000003
35.0744000000005 3.74050000000013 -2.37060000000003
41.3850000000003 9.3007000000001 -2.61610000000003
42.6282000000004 10.1801000000002 -2.63140000000005
41.1727000000002 13.7235000000001 -3.84300000000004
45.8125000000002 15.6224000000001 0.105099999999959
44.7601000000003 9.07340000000015 -1.60740000000005
45.6039000000003 11.7412000000001 -2.97320000000005
1.72679999999937 0.795400000000014 -1.5295
0.775899999999393 2.3232 -2.81039999999999
9.83709999999952 7.18029999999996 1.22590000000004
11.9125999999996 8.13109999999998 1.26690000000005
15.2551999999999 8.9722 1.56960000000003
17.5043999999999 8.94760000000001 1.30190000000002
15.2530000000001 10.8807000000001 10.0499
23.5911000000001 10.5449000000001 2.25009999999999
24.7378059458302 10.5075614443487 0.616770783374019
24.1056999999998 19.0454 -0.0339999999999907
29.2248000000001 13.877 -2.64840000000001
30.0528 18.2266000000001 -3.14260000000001
38.2572000000001 17.4152 -2.87770000000003
39.9432000000001 17.508 -3.08050000000004
41.6468000000001 19.8061 -2.13000000000004
43.1596000000001 17.8402000000001 -2.99900000000003
0.248499999999505 0.0968000000000264 0.484200000000015
-1.39390000000056 0.875500000000016 -1.18719999999999
8.55819999999969 6.46460000000003 6.47580000000001
11.5770999999997 9.9869 3.07320000000003

11.3417999999996 11.1261 1.65460000000003
9.84719999999983 3.91520000000007 7.98899999999998
27.3511000000002 10.5610000000002 14.1132
36.5452000000002 13.3372000000001 12.7396999999999
38.3470000000001 22.4398000000001 -0.425900000000037
45.6297000000002 16.4758000000001 6.70669999999996
42.2631000000001 19.5052000000001 -0.52830000000004
0.248499999999505 0.0968000000000264 0.484200000000015
-0.207500000000391 -2.02219999999998 -1.02249999999999
10.3835 1.0757000000001 7.05349999999997
15.7153000000004 -0.0354999999998037 3.39009999999996
15.2924000000005 -0.967899999999753 2.65889999999995
44.0587000000004 5.56500000000018 0.941399999999954
45.2672000000002 10.4251000000001 -0.22320000000005
ID=CYCdidFEMBRAAMAMfNB38399*

LM3=54
2.47971725988349 0.754453075592382 -1.68696536281549
1.62929999999941 -1.75650000000008 -2.52209999999964
13.3118999999997 0.887199999999737 1.29480000000025
13.8052999999982 1.54149999999938 1.53040000000025
12.5831 4.78049999999995 -0.291799999999929
19.0845999999996 4.14419999999985 0.707500000000153
20.5271999999997 5.25669999999992 0.45340000000009
19.9294999999996 2.17539999999985 9.48210000000021
21.7107999999993 8.30509999999969 -0.968799999999859
25.9697999999987 7.73069999999957 0.889200000000198
28.2278999999994 7.68919999999983 -1.27439999999987
31.1454000000001 0.171300000000092 -0.0655999999998949
33.9271999999994 8.15429999999988 -4.60009999999987
37.3041999999994 6.25549999999993 -5.51839999999987
43.7422999999994 11.9901 -5.33929999999989
45.4946999999995 12.5808 -6.2077999999999
43.7011999999993 16.4797999999999 -7.0285999999999
48.3569999999993 17.9396 -3.25529999999989
46.9141999999994 11.477 -5.4476999999999
47.7497999999994 14.2273 -6.5104999999999
2.37889999999951 1.4980999999999 -1.63629999999976
-0.192500000000498 2.53769999999992 -2.64739999999975
10.7270999999999 8.05909999999989 1.43900000000014
11.4600999999999 8.37049999999988 1.44250000000013
17.0183999999996 9.46659999999981 1.28940000000012
18.786279115841 9.33364661705062 0.739733006413787
18.2226999999994 12.0275999999998 9.40850000000019
24.8455999999993 11.5207999999997 1.01170000000014
26.6112999999995 12.6461999999998 -0.843899999999884
24.1486999999994 20.3803999999998 -0.345499999999874
31.2069999999994 15.6989999999999 -4.1647999999999
32.3368999999994 19.8221999999998 -5.43009999999989
40.4622999999992 19.8382999999999 -5.46289999999991
42.4362999999992 20.0920999999999 -5.8576999999999
43.0760999999992 22.6560999999999 -5.10699999999991
44.9520999999992 20.5004999999999 -6.79459999999989
0.0659999999994508 -0.0423000000000893 -0.0303999999997367

-0.772600000000543 1.50549999999991 -0.60029999999974
9.73179999999969 7.31199999999985 6.55150000000017
13.5041999999997 10.4248999999998 3.23720000000014
12.1261999999998 11.5764999999999 1.32570000000014
10.8477999999997 4.08709999999985 8.3035000000002
30.4558999999994 11.7835999999999 11.9385000000002
39.1188999999994 14.7881 10.2170000000002
40.7402999999992 24.8746999999999 -1.21399999999988
48.2579999999993 18.1772 3.55340000000013
45.0351999999993 22.1979 -4.04859999999989
0.0659999999994508 -0.0423000000000893 -0.0303999999997367
0.415199999999445 -2.25210000000009 -1.09729999999973
12.0570999999997 0.444099999999849 6.26100000000022
17.0639999999994 0.712199999999763 3.80680000000021
16.5114999999999 -1.27700000000005 1.45870000000018
46.8971999999996 7.81240000000004 -1.26609999999987
48.4846999999995 13.1529 -3.41719999999989
ID=CYCdidFEMUNKUNLMfNB3913**

LM3=54
1.98960000000045 0.559100000000152 -1.14330000000012
2.25609999999842 -1.21450000000042 -1.83619999999994
12.6519000000002 1.08130000000008 1.18859999999993
14.3128999999996 1.35579999999984 1.19639999999996
11.9234999999994 5.03499999999978 0.210400000000123
16.4753999999999 3.3421999999999 1.242
17.2082000000003 4.50930000000005 1.04790000000001
18.8705000000002 2.80260000000007 10.5156
20.2574 8.34259999999993 -0.400499999999977
22.5657999999998 7.2275999999999 1.32950000000005
25.2172 6.70790000000003 -1.04629999999996
28.5577000000003 -0.0837999999998682 -0.025399999999989
29.7807000000004 7.25630000000018 -3.7922
33.2901000000004 5.22950000000021 -3.9697
37.8647 10.9700000000001 -5.25649999999994
40.1548000000001 12.4877000000001 -5.26659999999994
38.1157673688763 16.2063961933555 -5.92314300458643
42.3610000000001 18.1430000000001 -1.66599999999993
42.0981000000002 11.6598000000001 -4.51099999999994
42.0078000000001 14.2769000000001 -5.89509999999994
1.68680000000028 0.929300000000178 -1.03340000000004
0.477099999999873 2.77890000000002 -1.58930000000001
9.09939999999987 7.71499999999992 1.40320000000004
10.6444999999998 8.45609999999988 1.22570000000005
13.7625999999999 9.41309999999988 1.58460000000005
15.2225 9.10739999999992 1.41270000000004
14.3615000000002 10.4678 9.29250000000003
20.4024 11.3151 1.85510000000003
21.8967999999999 13.1070999999999 0.0212000000000395
19.8355999999999 20.2561 -0.0394999999999472
25.8090999999999 16.2194 -3.33469999999993
26.3294 20.3511000000001 -3.41419999999994
33.8811 19.4773000000001 -4.28639999999993
36.1503 20.0161000000001 -4.45629999999993

37.7474000000001 22.1561000000001 -3.52639999999994
39.4561000000001 20.5552000000001 -5.36259999999992
0.107699999999586 0.00269999999990495 0.128399999999999
-0.642000000000325 1.45169999999994 -0.351900000000009
8.18640000000012 8.19300000000004 5.7499
11.3310999999999 9.95519999999992 2.04770000000004
9.69609999999987 11.6701999999999 1.28480000000004
9.41010000000015 3.72250000000006 7.33499999999998
28.5167000000002 12.1517000000001 13.2589
36.0784000000002 15.0659000000001 10.3088
32.9863000000001 25.0465000000001 -0.23939999999993
42.2447000000002 18.0554000000001 4.88420000000006
38.7036000000001 22.5823000000001 -2.47659999999993
0.107699999999586 0.00269999999990495 0.128399999999999
0.900499999998919 -1.59040000000037 -0.596599999999971
10.7575000000002 0.310400000000072 5.74239999999998
14.8255 0.985899999999994 2.76569999999999
15.9387000000001 -1.32159999999991 1.68240000000001
41.5745000000002 7.98210000000013 -0.888599999999954
42.8820000000002 13.7402000000001 -2.49889999999995
ID=CYCdidUNKMEXTABMfNB81457*

LM3=54
7.9422 0.9152 0.6509
10.4533 -1.7282 1.2463
39.8517 -1.289 -6.3693
43.4051 1.035 -9.8625
36.5761 9.0369 -8.6905
46.9791 4.3683 -12.3779
46.6793 5.8049 -13.4233
60.3822 0.6244 2.5681
58.3229 15.0983 -20.2503
63.241 11.6206 -12.0783
68.0744 10.9541 -15.9491
59.2803 -0.3122 -14.8823
72.2836 10.9475 -16.8742
76.4688 6.6349 -16.6005
83.0144 13.6365 -17.3784
89.1768 13.9948 -15.302
89.851 22.7391 -16.3447
95.1856 23.7959 -8.0949
94.5188 11.2878 -12.9612
93.1684 17.9236 -17.3377
6.9251 3.1661 0.6203
7.9735 5.802 0.6007
34.3351 19.942 -7.0068
38.8524 20.4306 -10.3317
43.9251 18.6948 -12.6404
44.1096 17.0144 -14.0181
54.2836 29.2372 2.666
61.947 19.4197 -12.1959
65.0296 21.8968 -15.598
52.8677 27.8905 -14.6509
68.9993 24.0179 -16.2411

71.0991 30.9123 -15.4847
79.7373 27.3409 -17.6717
85.5961 28.7839 -15.023
89.1078 34.0667 -13.286
91.0448 27.5697 -17.485
5.8228 2.1878 10.3797
7.829 7.0584 8.1738
29.6596 12.9757 8.6513
40.2845 22.645 -6.2623
42.8287 24.0264 -10.8259
44.1893 11.2543 10.3429
75.7339 18.07 10.4811
83.7349 20.7198 8.9722
83.1421 35.9259 -8.7027
92.0962 22.54 3.9395
91.0815 30.7036 -8.8146
5.774 0.8924 10.3847
9.8529 -3.4646 8.2664
32.4207 1.5126 8.7899
47.3517 -2.5781 -6.6282
50.7035 -2.11 -11.1493
91.4029 6.1746 -9.6854
94.6197 14.1458 -9.0509
ID=MANjavMALVIEBMNH104199

LM3=54
5.9505 -0.9795 0.7493
9.8132 -2.9858 0.8003
39.2141 -1.9403 -7.4266
43.1035 0.8469 -9.7387
35.5507 7.7696 -9.6184
46.033 4.4688 -12.2813
44.1685 5.0881 -12.9865
57.4845 0.6886 0.5503
56.6084 13.9294 -20.5107
62.0345 12.0195 -12.9704
66.783 10.0798 -15.7036
58.2989 -3.1287 -16.0968
73.2697 10.1852 -17.4231
75.4634 5.3143 -16.1754
82.7989 14.0156 -16.7545
88.7174 14.5773 -15.8845
90.5147 23.703 -17.7046
94.962 24.7348 -8.0491
94.6843 12.118 -13.1446
93.7149 20.0574 -18.7207
5.6546 2.1144 0.5021
7.7155 5.5589 0.9322
32.9963 19.3132 -7.0214
38.0352 20.2155 -9.8977
41.8885 19.3386 -11.8718
40.7766 17.8365 -12.3922
52.6676 28.3165 0.5916
60.9865 18.3314 -12.6693

63.949 21.5666 -15.6752
51.2537 29.3757 -16.0173
70.0303 25.0015 -16.7975
70.044 31.4516 -15.6971
78.8511 28.1116 -17.3365
85.3721 29.4971 -15.7316
88.4536 35.1492 -12.6901
91.3532 27.5634 -19.1111
6.7913 1.1231 11.651
8.3994 6.1209 9.3241
30.3659 12.6336 9.1384
42.4723 21.0946 -6.5081
48.096 26.743 -14.1379
45.4908 10.2499 9.832
72.7556 18.2965 9.9215
81.7255 19.7512 8.4554
82.5667 36.3137 -7.6439
92.7775 21.7926 2.8588
91.3394 31.1258 -10.0684
6.615 -0.0975 11.8702
11.0272 -4.3249 9.5935
33.3991 1.9442 8.5281
49.2244 -2.2625 -6.4271
55.1068 -4.9105 -14.2165
90.1891 5.3353 -6.9659
95.4235 13.2068 -10.0197
ID=MANjavMALMALBMNH32681*

LM3=54
6.5367 -0.3494 -0.1374
8.9052 -1.2672 -0.7282
34.9249 -0.0126 -7.7444
39.0763 2.495 -10.0648
30.8333 8.2806 -8.2398
41.9566 5.5719 -11.6077
41.6753 6.2211 -12.6129
53.8708 0.3678 0.6427
53.9342 14.1514 -19.6716
57.7241 11.472 -11.4892
60.6635 10.091 -15.1809
53.3992 -0.369 -16.6792
65.2783 10.7989 -17.3057
70.1707 5.7817 -15.9117
76.7195 12.1089 -17.0966
82.3836 12.3365 -15.6065
83.5493 21.1168 -18.6913
88.708 22.1746 -10.4605
89.4542 10.269 -14.9368
86.7948 16.4016 -19.2431
6.4113 3.4209 0.2396
7.9638 5.5315 -0.3257
30.7469 17.4106 -7.8392
34.7461 17.6736 -9.6017
39.2656 16.437 -11.7051

39.1554 14.7976 -12.6631
49.4979 26.8578 1.8647
56.4409 17.4269 -11.5879
59.1033 18.9058 -15.1064
47.1188 25.8322 -15.7829
63.0983 19.862 -16.9182
64.5514 28.9631 -15.6563
73.8863 25.1022 -16.3527
78.8698 27.7757 -15.9886
84.2333 33.2241 -15.1415
84.3632 26.2358 -19.3697
5.9038 2.5787 9.4067
5.5225 7.0646 5.796
25.1081 12.1048 7.9019
36.4984 22.0798 -6.0612
40.6554 23.6739 -12.641
42.0168 11.8066 10.3097
66.3657 18.5216 10.3809
76.1229 20.442 8.5994
74.9901 34.5511 -9.261
84.187 22.3579 3.1483
84.1109 30.5214 -10.0239
6.489 1.4593 9.2596
8.3397 -1.8744 6.0473
27.2214 3.3277 8.1221
42.6335 -1.571 -5.9334
47.2112 -0.8444 -12.1917
82.5093 5.5499 -8.0614
89.0129 14.8146 -10.001
ID=MANjavUNKUNKBMNH73158*

LM3=54
0.0356999999985625 -1.30799999999935 -7.86430000000018
3.70990000000057 -2.01449999999892 -6.94360000000061
28.170199999999 -0.0754000000001681 -10.0703999999998
30.2466999999982 1.6772999999994 -12.1700999999996
25.9274999999986 8.37899999999967 -11.2568999999997
32.9533999999994 4.49749999999983 -13.9130999999998
32.1522999999985 5.36949999999955 -14.2896999999997
40.0848999999986 0.451199999999623 -0.601999999999764
42.0507999999982 14.0272999999997 -16.4396999999997
46.7522999999983 13.0720999999996 -10.1060999999997
50.2978999999984 10.6118999999996 -12.3473999999997
46.192599999999 0.217599999999809 -14.6031999999998
55.0927999999983 12.4281999999996 -12.4588999999997
57.5811999999983 7.59339999999959 -11.3862999999997
63.7111999999981 14.4323999999996 -10.4953999999996
68.9168999999981 15.4850999999995 -9.35619999999964
69.4507999999979 22.9195999999995 -10.7066999999996
72.6336999999979 23.9833999999995 -2.3011999999996
73.070199999998 13.5602999999995 -6.26219999999963
73.2849999999979 19.1463999999995 -10.9010999999996
-0.549300000001694 0.947700000000365 -7.73989999999991
1.54849999999823 2.78170000000025 -6.56759999999985

22.5342999999984 16.8014999999998 -9.94669999999965
25.4157999999984 16.7606999999997 -11.8415999999996
30.0487999999985 16.3096999999997 -13.6474999999997
30.7172999999984 15.8705999999997 -14.4187999999997
33.3864999999984 24.2787999999998 -0.656999999999644
45.9986999999983 17.8175999999996 -9.95119999999966
47.4434999999983 20.3784999999997 -12.3932999999997
37.6937999999981 26.8802999999997 -14.6505999999996
52.3363999999982 21.9661999999996 -12.5502999999997
51.6843999999981 28.7803999999996 -11.5067999999996
59.720399999998 26.0954999999996 -10.2630999999996
64.6143999999979 28.4461999999995 -8.54979999999961
67.3844999999977 33.0510999999995 -5.77609999999958
69.3039999999978 26.4088999999995 -11.4691999999996
-0.502700000001227 0.242800000000515 -0.100600000000072
-1.10390000000164 3.59200000000032 -1.58239999999992
15.9506999999984 10.0578999999999 3.13870000000025
27.2846999999985 19.4870999999998 -8.11889999999965
30.3454999999983 22.3534999999997 -12.0956999999996
28.4963999999987 9.26839999999983 6.47860000000024
48.8436999999982 15.9716999999996 11.1186000000003
57.7711999999981 18.8823999999996 11.5355000000003
60.6663999999978 33.4628999999996 -2.55469999999961
68.6667999999979 22.6804999999996 6.96610000000038
68.0375999999978 30.0457999999995 -2.9114999999996
-0.249100000001119 -0.75999999999943 -0.245500000000092
1.79309999999944 -3.82699999999928 -1.70200000000025
19.2697999999989 1.23740000000002 2.91560000000011
34.9176999999994 -0.0703000000001082 -8.34079999999985
39.2292999999992 -0.847700000000186 -12.5543999999998
69.2788999999981 9.53689999999954 -3.00979999999967
73.0313999999979 16.7208999999995 -3.05989999999961
ID=MANjavFEMTHABMNH810548

LM3=54
6.79490000000178 0.746999999999175 0.748599999999748
10.2039000000025 -0.765499999999822 1.30729999999967
37.9671000000008 -0.665800000000595 -7.2314
41.1590000000016 0.731499999999646 -9.07250000000022
34.3689000000013 8.85509999999953 -8.00040000000009
44.8006000000012 4.58239999999979 -12.0329000000001
42.7276000000011 5.3198999999997 -12.2037000000001
57.5015000000009 0.79129999999985 0.42299999999999
56.6595000000013 14.3246999999999 -19.4077999999999
60.9846000000014 12.0835 -11.8241000000001
64.7546000000015 10.296 -15.1485
57.7064000000011 -0.956600000000112 -15.9158
70.3369000000012 10.8645 -16.9435
73.6565000000009 6.17439999999996 -15.9773999999999
80.4032000000011 13.931 -17.17
87.2424000000012 14.217 -16.0807
88.4759000000014 21.4027 -18.9622
93.5383000000015 22.4062 -10.2593
93.3641000000011 10.3082 -14.0797

90.3735000000013 18.3664 -20.2313999999999
6.41920000000181 3.5795999999993 0.951899999999835
9.27070000000159 6.16119999999941 1.5550999999999
34.1669000000015 18.0198999999996 -6.69160000000003
38.4588000000012 18.9154999999995 -8.00339999999997
41.4019000000015 18.0005999999997 -11.5062000000001
40.3425000000014 15.4211999999997 -11.9622
52.3382000000013 26.2311999999997 0.236200000000036
60.3592000000011 18.1593999999999 -11.8475
62.7723000000011 21.1116999999999 -14.6876
48.993442529714 26.1360441535135 -13.8206315577904
68.527200000001 22.8560999999999 -16.8814999999999
70.2381000000012 29.5000999999999 -15.3346999999999
78.3277000000012 25.7163999999999 -16.9116999999999
84.5773000000013 27.7061999999999 -15.7122
88.6419000000015 33.8708 -13.1916999999999
89.1834000000015 24.5343 -20.1339999999999
5.63280000000214 1.68159999999968 9.45849999999985
8.54710000000187 7.13429999999954 7.9898999999999
28.5560000000011 12.2547999999996 8.55700000000001
40.9163000000014 21.5200999999996 -6.8842
41.7840000000015 22.7556999999997 -9.6387
42.1749000000012 9.41749999999969 10.6942
71.5062000000012 17.8610999999999 9.29130000000001
78.2953000000012 19.7573999999999 8.04210000000002
80.3162000000014 34.4979999999999 -7.11149999999993
88.7808000000014 22.0394999999999 2.58390000000003
88.5899000000015 31.0469999999999 -9.66949999999994
5.32030000000218 0.7835999999997 8.98789999999982
10.9830000000022 -3.61270000000015 7.9086999999998
31.6730000000011 1.57009999999964 8.03039999999999
45.7803000000011 -2.27810000000024 -6.41690000000011
48.1823000000011 -2.04910000000015 -10.8081000000001
88.153500000001 6.11669999999997 -7.70629999999997
93.3110000000012 13.2458 -9.69799999999996
ID=MANjavUNKTHABMNH98836*

LM3=54
1.60360000000173 -1.17339999999939 -7.6074999999993
4.2198999999993 -2.70759999999944 -7.67759999999915
33.9283000000015 -0.840499999999709 -11.4651999999998
34.4575000000014 0.54660000000009 -12.1296999999998
31.3794000000016 8.52350000000038 -13.1357999999998
38.7259000000016 3.80440000000026 -14.7336999999999
36.6941000000016 4.96010000000018 -15.4052999999998
50.7344000000017 0.965800000000424 -2.46179999999988
50.2137000000014 12.5958000000002 -21.3009999999999
55.7606000000013 11.9128000000003 -12.3884999999998
61.2849000000012 10.7202000000002 -15.4843999999998
55.1258000000011 -0.831399999999803 -17.7466999999998
65.5694000000012 10.2468000000002 -16.4613999999998
66.3538000000012 5.08960000000025 -15.7563999999998
75.8573000000011 10.6904000000003 -14.9458999999997
82.0092000000011 13.7788000000004 -11.5058999999997

82.5229000000014 20.8700000000004 -15.2196999999997
85.4500000000012 21.6970000000004 -5.18029999999962
83.764500000001 10.7915000000004 -8.26699999999962
87.5972000000014 16.6882000000004 -14.6282999999996
0.0651000000016341 3.73660000000048 -6.54359999999944
1.18610000000139 5.94320000000048 -6.29679999999945
27.7846000000017 17.9813000000004 -11.7780999999998
28.5448000000017 17.4419000000003 -13.2913999999999
35.3438000000017 16.2161000000003 -15.8546999999999
33.9341000000016 14.8616000000003 -16.5340999999999
44.8391000000017 24.2819000000003 -2.16589999999986
54.4486000000015 16.6584000000003 -12.9906999999999
58.1158000000013 20.3404000000002 -14.9021999999998
48.1291000000017 27.6489000000002 -17.5206999999998
63.0559000000013 22.4573000000002 -16.3888999999998
61.3794000000015 27.7450000000003 -15.5970999999998
72.3114000000013 27.6242000000003 -14.8158999999997
79.2654000000013 27.9816000000003 -11.0179999999997
80.0677000000015 29.8836000000004 -8.58099999999964
84.6432000000014 27.0307000000004 -14.1974999999996
-0.270199999998904 -0.495999999999478 -0.134699999999378
0.523389037866649 4.88482816327424 -0.634662055955723
21.3326000000013 9.86220000000045 2.72290000000032
33.6408000000018 20.5169000000003 -10.2400999999999
36.2646000000017 23.8721000000003 -15.6536999999999
37.1279000000016 10.1772000000004 5.65850000000021
59.2302000000015 15.8307000000004 8.93260000000024
71.2236000000014 18.0805000000004 8.63570000000031
74.0366000000015 33.4637000000003 -4.98589999999966
78.2624000000013 19.4999000000004 6.03650000000037
81.1018000000013 28.5398000000004 -6.02029999999963
0.807100000001014 -1.47209999999946 0.349400000000663
4.27440000000043 -4.69959999999948 0.0128000000007075
23.895600000001 1.68950000000046 3.28900000000037
38.6655000000012 -0.88099999999978 -9.86369999999983
42.9607000000013 -1.71949999999978 -13.9732999999998
80.0687000000012 6.47520000000038 -4.77479999999967
84.7457000000011 13.7881000000004 -6.09519999999962
ID=MANjavUNKVIEMNHN622119

LM3=54
5.3707999999992 -0.167399999999416 -0.429099999999668
8.36990000000266 -1.10319999999918 -0.450000000000466
34.4186000000001 -0.0596999999998405 -7.55010000000008
36.2910999999997 2.0520000000001 -8.55319999999998
30.1644999999997 8.21209999999993 -9.44869999999996
39.1637999999998 5.7972000000002 -11.3512
38.0224999999994 6.02910000000003 -11.8562
48.872 1.43270000000005 -1.38259999999997
50.4959000000002 13.7186000000002 -17.8462
53.1530000000001 11.8771000000002 -10.1053
56.6187000000003 11.4145000000002 -12.7518
50.4956000000003 -0.0801999999997928 -14.6063
61.5994999999996 10.4643 -14.5275999999999

64.3452999999998 5.68170000000002 -13.4218999999999
72.4249 12.3839 -13.3013999999999
77.8986000000001 11.8866999999999 -12.4057999999998
79.6776999999999 20.4649999999998 -14.6612999999998
84.5161 21.0503999999999 -5.62369999999978
81.15 10.9702999999999 -10.9474999999998
83.6962000000002 16.1908999999999 -15.2151999999998
4.75589999999987 2.35620000000035 -0.138999999999767
6.42690000000017 4.73010000000021 -0.230499999999821
29.9675000000002 16.8290000000002 -6.83170000000002
32.4876 16.1352000000002 -8.0066
36.3463999999999 14.6783000000001 -10.4739
35.7885999999999 13.6363000000001 -11.7214
44.2909999999999 23.9974000000001 -0.753799999999927
51.4219 16.7241000000001 -9.94469999999995
54.4732 19.1526000000001 -12.2252999999999
44.4555 25.3660000000001 -14.2967999999999
59.4500999999999 21.6854 -13.9664999999999
59.2562999999998 26.7284 -12.7994999999999
69.5962 24.3794 -13.5373999999999
74.9597999999999 26.9160999999999 -11.3713999999998
76.9074999999999 29.9895999999999 -9.16649999999977
81.5808 26.1835999999999 -14.7896999999998
7.16980000000101 2.36250000000027 8.43160000000003
5.53740000000065 6.12240000000018 6.66730000000011
26.1048000000005 11.7759000000001 7.73369999999997
35.8036999999999 20.1578000000001 -6.91859999999997
38.8354 22.2265000000001 -11.5077
39.4288000000003 10.6531000000001 9.52370000000001
59.0063 15.3139 10.2010000000001
69.8304 17.8767999999999 9.72610000000016
72.5893999999999 32.7604999999999 -5.30859999999979
78.1526000000001 19.6375999999999 6.25340000000019
80.6357 28.8212999999999 -7.58459999999977
7.49670000000112 1.7033000000003 8.51
7.51830000000165 -2.42029999999954 6.88289999999986
27.5790000000008 2.70840000000021 7.69499999999988
40.4212000000002 0.139100000000208 -7.01190000000004
44.3178000000002 -0.296299999999784 -11.8356
78.9514000000001 5.39309999999994 -5.82559999999987
83.3908000000002 11.8760999999999 -7.50119999999982
ID=MANjavUNKINDMfNB60539*

LM3=54
4.91299999999644 -0.111800000000023 -0.124800000000041
7.94019999999846 -1.11260000000015 -0.486400000000253
36.1483999999988 -1.63229999999962 -7.13590000000004
39.1221999999993 -0.197999999999389 -8.52980000000011
33.0718999999995 7.83600000000069 -9.7572000000002
42.0583999999987 3.32320000000038 -11.5516
41.9081999999991 4.8901000000003 -13.367
54.3384999999995 1.14190000000036 0.0261999999998912
52.9694999999994 12.6247000000005 -18.5212000000002
58.0448999999991 10.9933000000003 -11.5307000000001

61.8093999999997 8.72670000000039 -14.4417000000002
57.2925999999994 -2.20009999999967 -15.2001000000001
65.8161999999997 9.89300000000041 -16.9120000000002
69.2543999999995 5.18690000000032 -15.3156000000002
77.0670999999997 11.8301000000004 -15.9332000000002
82.9456999999997 12.8520000000004 -13.7790000000003
83.0858 20.6624000000004 -16.4270000000003
87.8076999999997 21.4242000000004 -7.41480000000035
86.7050999999997 10.5202000000004 -10.6975000000003
88.6342999999998 16.6879000000004 -15.7441000000003
4.77639999999733 2.14980000000005 -0.120200000000133
7.13189999999812 5.19680000000023 -0.738500000000243
31.2202999999992 17.2519000000006 -7.51360000000019
34.9633999999993 17.8172000000006 -9.54910000000018
39.1277999999993 15.3906000000006 -11.7390000000002
39.3045999999994 14.1538000000006 -13.1657000000002
48.2820999999992 24.3615000000005 0.0872999999998381
56.0311999999992 16.9387000000004 -11.8787000000001
58.8785999999995 20.2535000000004 -14.8637000000002
49.7929999999994 27.8905000000006 -15.1994000000002
62.8835999999997 21.4386000000005 -16.5656000000002
63.1704999999995 28.4028000000005 -14.4755000000002
73.7044999999996 25.7929000000004 -16.0470000000002
79.5194999999999 27.2998000000005 -13.5480000000003
81.3642999999999 31.0291000000004 -10.5452000000003
85.3910999999999 26.2276000000004 -16.3123000000003
10.1265799578253 3.68937123290491 8.98222431543352
10.1387999999983 8.50020000000024 7.29129999999972
28.0952999999993 12.5473000000005 7.76479999999973
38.5370999999991 22.3949000000006 -6.77160000000017
40.6911999999992 24.1816000000006 -11.3586000000002
41.4055999999992 10.1330000000005 8.64229999999984
64.3054999999994 15.9875000000004 9.6304999999998
74.3030999999995 18.3240000000004 8.28269999999974
76.7514999999998 33.2589000000005 -5.54830000000031
84.8524999999998 20.8084000000004 3.39929999999971
83.9164 29.3783000000004 -8.86600000000034
10.4196841730744 2.7518113700809 9.05956219977381
13.6543999999983 -1.26429999999996 7.55639999999973
30.5876999999993 2.31050000000042 7.84339999999976
44.0361999999993 -1.88209999999957 -6.31000000000009
46.8673999999993 -2.23219999999963 -10.7947000000001
83.0391999999997 6.19370000000036 -5.63820000000026
87.3684999999998 13.0460000000004 -8.30470000000031
ID=MANjavFEMINDMfNB2720**

LM3=54
4.64849999999761 0.184900000000223 -0.240199999999475
6.91890000000086 -1.13459999999941 -0.174200000000072
33.2735999999996 -0.698299999999913 -5.26040000000006
35.8596999999992 1.0013 -7.38809999999993
30.1289999999991 7.34859999999988 -7.7187999999999
38.6701999999994 4.43910000000015 -9.21799999999996
38.436799999999 5.13519999999995 -10.3897999999999

50.0716999999993 0.435899999999978 -0.0531999999999373
47.9034999999995 12.8870000000002 -15.3203999999999
50.9087999999995 9.50220000000017 -8.53069999999994
55.2383999999994 9.43850000000016 -11.0587999999999
48.5260999999995 -0.839299999999845 -11.7908
58.7536999999986 10.0303 -12.5100999999998
63.7710999999986 5.81569999999997 -10.5894999999998
70.2594999999983 12.4693999999999 -11.7142999999997
75.155199999998 12.3521999999999 -11.2899999999997
75.9550999999977 20.4110999999998 -13.5512999999996
80.2117999999975 21.5593999999998 -5.88289999999959
78.1852999999977 10.9924999999998 -9.38649999999964
80.1595999999978 16.8136999999998 -14.4459999999996
4.39609999999816 2.07410000000026 -0.0429999999995822
5.71199999999851 4.34770000000013 0.486700000000356
27.1471999999994 16.0815000000002 -4.61619999999989
31.6375999999994 16.2008000000002 -6.86179999999988
35.3851999999993 14.3337000000001 -9.09059999999989
35.2115999999993 13.5779000000001 -9.9382999999999
41.958099999999 21.9756000000001 0.505400000000188
48.6171999999992 16.3957000000001 -8.70499999999988
51.8673999999991 18.6293000000001 -11.1500999999998
41.399899999999 23.8565000000001 -11.1612999999998
55.8872999999988 20.3139 -12.2067999999998
56.4621999999985 25.9417 -10.7440999999997
66.6462999999983 24.1674999999999 -11.1563999999997
71.2257999999979 26.3993999999998 -11.2424999999996
73.7169999999977 29.5983999999998 -8.66169999999957
77.1823999999977 25.4411999999998 -14.0839999999996
4.70809999999917 0.385600000000194 7.95380000000026
3.78329999999889 3.80960000000011 6.31190000000031
22.2571999999995 9.78310000000006 8.8424000000001
33.0591999999992 19.6603000000002 -4.13639999999985
36.7622999999992 22.5392000000002 -9.15549999999984
35.9416999999994 9.20220000000005 10.5425000000001
56.1991999999985 14.7255999999999 11.4395000000002
65.8151999999981 17.3118999999999 9.95340000000032
69.7039999999979 31.1020999999998 -3.38379999999961
74.7527999999978 20.4626999999998 5.43020000000037
75.3542999999977 28.5486999999998 -7.41029999999958
4.96299999999925 -0.13229999999982 7.33670000000023
6.09049999999986 -3.65719999999967 5.23200000000013
24.7085999999998 0.571000000000115 8.45170000000002
38.8325999999999 -0.585599999999855 -5.58360000000003
44.1556999999996 -1.04789999999983 -10.2443
76.5975999999981 8.53859999999987 -5.1149999999997
79.9167999999977 14.5554999999998 -8.37219999999964
ID=MANjavUNKINDMfNB34906*

LM3=54
4.8239 -0.5192 0.3405
7.2812 -1.7672 0.8038
37.9093 -2.4093 -3.7169
38.2118 0.3429 -4.7562

32.9439 6.8804 -5.2861
43.9023 3.3678 -8.2032
44.6842 4.9707 -9.5492
57.4302 0.2252 2.5812
55.6551 12.0564 -15.4892
60.6291 10.0498 -9.2141
64.6575 8.2275 -12.874
56.3265 -2.9949 -11.5992
68.6511 8.6728 -14.5405
72.799 3.4498 -12.8912
81.1427 10.6949 -13.4615
87.4818 11.3793 -12.8016
86.8053 19.018 -15.4532
92.266 20.6318 -7.3553
89.6782 9.0138 -10.9989
92.7463 15.3624 -16.4639
4.493 2.4336 0.396
6.2229 4.7025 0.4411
33.1641 17.1042 -4.4706
33.9235 15.1778 -4.2038
41.5267 14.639 -8.4147
42.0769 13.4098 -9.6973
51.8732 23.1415 0.4171
59.4979 16.0556 -9.2477
61.3806 18.544 -12.5792
49.8341 25.6476 -12.3949
65.802 20.2312 -14.4275
66.5286 26.9439 -13.0307
77.209 24.7697 -13.6986
82.4879 26.2309 -12.9653
83.1551 29.9758 -10.924
87.9822 24.7653 -16.9426
5.5383 1.2092 9.1765
5.9173 4.8963 8.4842
29.4714 11.1212 10.6112
39.1669 20.8281 -2.9261
43.3618 23.1487 -9.1749
45.4411 10.4944 11.726
69.8917 15.9787 11.483
82.2478 18.6642 8.409
82.2956 31.2275 -3.9431
89.0227 20.8234 3.7952
86.4146 29.1696 -9.389
6.4397 -0.065 9.452
7.8491 -2.8885 9.015
31.1028 2.3983 10.9802
44.6974 -2.192 -2.4411
49.3877 -2.531 -8.2825
89.3589 8.4299 -3.8507
92.5199 12.3861 -9.1267
ID=MANjavUNKINDMfNB36071*

LM3=54
6.9976 0.0634 0.7861

9.6867 -2.153 0.8188
45.1012 -2.5656 -8.453
49.6997 0.217 -10.8685
39.7092 8.15 -9.8864
55.0524 3.2465 -14.0887
52.9862 3.8991 -14.6295
71.9062 -0.029 -2.1836
67.1209 15.5941 -24.7718
76.1516 10.9554 -15.1074
79.2456 10.5732 -18.9999
69.1436 -3.2386 -18.7157
84.211 12.4474 -20.7297
86.8789 5.0026 -18.9087
95.7994 13.991 -18.7336
103.8067 15.5754 -18.5161
105.9953 23.9576 -21.6259
111.3284 24.7596 -10.409
108.6524 11.98 -15.3195
111.6036 19.7192 -22.6389
6.6919 2.492 0.8954
7.4386 5.3671 0.9055
40.2215 19.5261 -7.4422
45.4003 19.2876 -10.5439
51.2012 18.7306 -13.7695
47.7743 17.4298 -14.0283
63.1648 26.9458 0.6089
73.531 19.7261 -14.4906
76.0034 23.088 -17.4331
60.5457 30.3892 -17.241
81.0466 23.6321 -19.9758
80.4736 32.0436 -17.899
92.9302 28.6114 -18.8971
100.7006 31.1115 -17.5882
102.8396 35.9394 -12.488
108.6773 30.3731 -22.6577
11.0105 2.7913 14.0102
10.0336 7.6352 11.6366
38.2879 12.3889 11.2889
52.002 24.7167 -7.4649
57.6179 28.9004 -14.8396
57.2173 10.9028 11.6148
85.3626 17.6414 9.8086
94.4906 20.2018 8.7114
93.3067 38.8138 -8.6801
105.0699 22.5735 3.3775
106.1323 33.57 -11.5559
9.5658 1.4689 14.3714
12.3226 -3.9392 11.4703
39.807 1.7914 11.3211
58.1261 -2.784 -8.5026
63.6167 -3.5871 -15.497
101.2834 4.449 -10.0864
109.2361 14.6463 -11.9953
ID=MANjavUNKMALMfNB37805*

LM3=54
5.40909999999912 -0.0208000000005202 0.880099999999964
8.74299999999905 -1.43770000000051 0.864600000000007
37.7812999999985 -0.474200000000539 -5.42160000000001
40.4597999999986 1.07969999999951 -7.25730000000003
32.7780999999989 8.62499999999959 -6.62509999999998
43.2906999999984 4.42569999999945 -9.75820000000001
40.7220999999984 3.91679999999944 -9.29129999999999
55.0880999999978 1.96329999999919 0.495899999999988
52.9043999999979 13.6133999999992 -16.8229
58.4925999999979 11.6533999999992 -9.30780000000001
61.5210999999976 10.5494999999991 -11.7195
56.5502999999979 -1.33960000000081 -13.5939000000001
66.0872999999974 11.178999999999 -13.8405
68.6369999999976 6.478099999999 -13.6962000000001
77.1456999999976 13.1527999999989 -12.5621000000001
83.0219921274863 12.8636068538501 -12.2192886561971
83.8056999999971 20.0478999999987 -14.155
88.8817481900567 21.3682916121873 -6.25850165096391
87.5535341888343 10.0881016126114 -10.3935081007195
88.2897130822396 16.3618821181941 -15.0724732748351
4.75379999999912 2.65079999999946 0.837300000000005
7.26099999999895 5.82319999999945 0.680200000000027
32.171099999999 18.6172999999996 -5.44259999999992
35.091799999999 18.8722999999996 -6.95829999999992
39.2148999999986 17.0490999999995 -8.83319999999992
37.1208999999987 15.5908999999996 -8.69969999999996
49.4526999999982 25.6022999999993 -0.191599999999904
56.1942999999978 18.9080999999992 -9.40569999999994
59.6120999999976 20.6037999999991 -11.9798999999999
49.2947999999979 28.7742999999993 -13.7300999999999
63.6767999999976 21.9803999999991 -13.6892
64.0394999999977 27.7104999999991 -12.8649
74.0900999999973 25.1766999999988 -12.3018
79.7022999999971 26.8925999999988 -12.1428
82.3296250948837 31.3033430038299 -10.2712515716266
85.8496875626951 25.4212390350519 -15.1483317638887
6.65999999999903 1.5797999999994 8.28910000000001
6.61009999999906 6.25759999999943 7.58240000000002
27.2348999999989 12.7429999999995 8.51920000000008
37.8636999999989 23.5351999999996 -4.3546999999999
39.4687999999983 24.0006999999994 -8.4763999999999
41.2306999999984 10.3448999999994 11.0499000000001
64.0847999999977 16.464399999999 11.8662
73.5375999999973 18.7482999999989 10.0505
76.7732999999974 33.4778999999988 -6.56070000000001
84.9213999999969 20.9365999999986 4.53409999999998
84.3409503258538 29.2752975672697 -7.86023677818047
7.03329999999899 1.2455999999994 8.50979999999998
8.64289999999894 -3.01520000000062 7.79880000000001
29.2429999999988 1.77459999999947 8.33680000000003
45.6555999999982 -1.64540000000062 -6.45380000000005
46.978299999998 -2.12180000000072 -8.93010000000004

83.4434999999972 5.76489999999874 -6.51360000000006
88.5159413267234 13.015067530159 -7.7653060806409
ID=MANjavUNKSINMfNB4621**

LM3=54
4.60249999999697 -0.51280000000028 1.07770000000057
7.79039999999812 -2.03720000000057 0.882000000000416
30.7643999999994 -1.51460000000032 -4.56369999999972
33.9927999999993 0.578699999999496 -6.40889999999974
28.8600999999993 6.9807999999995 -6.50089999999972
37.6481999999997 3.24489999999958 -8.76809999999967
36.6000999999997 4.96719999999968 -9.93259999999972
49.0351999999991 0.639899999999481 -0.0577999999996408
48.1794999999992 11.6525999999996 -16.1795999999996
52.0250999999989 9.06519999999949 -10.2649999999995
55.7759999999989 8.54579999999957 -13.3848999999996
49.9746999999991 -1.14870000000048 -13.3766999999996
55.9831999999992 8.43089999999961 -13.0435999999996
62.1832999999988 4.22629999999953 -14.0807999999995
70.0089999999987 11.1221999999995 -14.2684999999994
75.9341999999983 12.6218999999994 -14.2814999999993
77.0717999999982 18.2621999999994 -15.3921999999993
81.7356999999978 18.9444999999993 -8.24149999999928
78.8429999999981 9.02079999999936 -11.0059999999993
80.5265999999981 14.6541999999993 -15.7067999999993
4.14439999999788 1.89179999999964 1.28210000000042
6.13349999999816 4.97089999999962 1.12080000000036
26.7633999999995 15.7677999999998 -4.56459999999977
29.4686999999994 15.0062999999998 -6.08879999999975
35.1115999999992 13.7428999999997 -8.93519999999969
36.5804999999992 12.5886999999996 -10.0463999999997
43.5326999999989 22.1722999999996 0.16820000000038
50.173599999999 16.1093999999996 -10.3300999999996
53.060899999999 17.9040999999996 -13.1491999999996
43.194299999999 23.5753999999996 -13.2260999999996
56.3255999999988 19.6009999999995 -14.7344999999995
57.7725999999986 25.3059999999995 -13.5694999999995
67.1333999999984 23.0719999999995 -13.7790999999994
72.4687999999982 24.5089999999994 -12.2874999999994
74.861499999998 27.9871999999994 -10.4005999999993
78.2314999999981 22.9234999999994 -15.4303999999993
5.81279999999842 1.29929999999956 9.56710000000033
7.41999999999847 5.62549999999959 7.64930000000032
24.7365999999991 11.0159999999996 7.94160000000027
34.6062999999992 19.5724999999997 -5.0941999999997
36.1585999999991 20.9877999999997 -9.60139999999966
39.3768999999991 9.53049999999953 8.84910000000033
61.4515999999984 15.4682999999994 7.87270000000052
69.4127999999982 17.4063999999994 6.0069000000006
71.0944999999982 29.2022999999994 -5.96609999999936
76.3491999999979 19.2629999999993 2.53640000000066
77.6022999999979 26.8624999999993 -8.4934999999993
5.92379999999837 0.764299999999535 9.73000000000033
9.17509999999838 -2.21780000000052 7.68920000000034

26.4746999999991 2.18909999999955 7.90850000000028
38.6755999999996 -2.00530000000053 -4.43899999999968
42.6104999999994 -1.4081000000005 -10.1589999999996
75.8209999999983 6.24809999999939 -6.0769999999994
80.759099999998 11.9680999999993 -8.31359999999932
ID=MANjavUNKINDMfNB60540*

LM3=54
4.8937 -0.1846 0.7185
8.1732 -1.0614 1.4736
34.0963 0.0456 -4.3257
36.5667 2.0439 -5.8676
32.0204 8.0339 -6.717
40.7544 4.4922 -8.4986
40.3094 5.7245 -9.7378
50.9808 0.8384 1.3432
51.1856 12.907 -15.8647
54.8339 10.8026 -9.916
57.4351 9.5742 -12.8805
51.9991 -0.4907 -12.5177
62.1911 9.6852 -14.3664
64.7078 5.0815 -13.7588
72.8472 10.9617 -13.7488
77.9083 11.6631 -13.9664
78.6508 19.4063 -16.03
83.0708 20.5483 -7.165
81.8027 8.9384 -10.9025
82.8462 15.6888 -16.5544
4.4064 2.3835 0.7785
6.5058 4.9158 1.4283
29.8721 16.3626 -4.0029
33.0183 16.1208 -6.0767
37.9167 15.2351 -8.7663
38.0987 14.0062 -9.9362
46.5912 23.1631 -0.0466
53.4261 16.766 -9.7195
55.3917 18.4654 -12.5473
45.7438 24.9555 -12.9226
59.6687 19.8386 -14.1323
59.388 25.4726 -13.7759
69.4609 23.5957 -14.3111
73.885 25.0454 -13.3724
75.8099 29.7865 -11.0602
80.1825 24.7157 -16.5883
5.0108 1.2678 8.6213
5.4481 5.7039 7.3909
28.1394 12.7655 8.7684
36.3287 20.722 -5.032
40.4194 22.6441 -9.9037
41.0763 10.4133 10.0018
62.2659 15.9169 9.4639
72.6449 17.831 7.1153
72.7755 31.3885 -6.6442
80.4566 20.0587 2.697

78.975 28.1662 -9.2075
5.5082 0.7465 8.8207
7.4875 -2.2757 7.5122
30.1597 2.7873 8.7058
41.2317 -0.2982 -4.3661
46.3537 -0.1605 -9.9795
78.9912 6.0395 -6.156
83.3699 11.9656 -9.0564
ID=MANjavUNKINDMfNB75151*

LM3=54
6.4162 -0.0996 1.1027
10.2733 -1.1923 1.3679
37.5541 -1.155 -5.2825
39.5985 1.6542 -7.6567
33.4875 8.5791 -6.7867
43.6461 4.5653 -10.6865
43.1617 5.4116 -11.5219
59.3522 1.1333 -0.4012
55.9723 14.0473 -18.4076
60.674 12.094 -11.9748
64.4293 10.1397 -15.366
56.7529 -2.2524 -15.2351
68.6191 10.6783 -17.0039
71.2216 6.0371 -15.6891
79.6525 13.1446 -16.0519
85.3574 13.7471 -14.6099
86.3973 22.0377 -17.0782
92.3912 23.7074 -5.4206
89.3984 10.1441 -10.974
90.9385 18.1461 -16.9436
6.1068 3.5211 1.63
8.587 6.3808 2.0675
32.9393 18.5448 -5.0641
36.5108 17.7493 -7.6204
40.8018 16.3201 -10.6355
41.2056 15.8966 -11.6117
50.0427 27.1031 0.0177
59.0733 18.6242 -11.9222
61.0322 22.2367 -15.4664
49.4723 28.8279 -15.5533
65.4979 23.3853 -17.0555
65.9526 29.139 -15.8193
76.8551 26.596 -16.6131
82.2175 28.7037 -14.7553
83.8282 34.0247 -11.7091
87.779 26.7954 -18.1152
6.3428 1.6245 9.9276
6.0681 5.5863 8.8298
27.77 11.223 10.5146
40.126 23.2988 -6.0594
41.9135 25.2943 -10.5669
43.5939 10.9067 10.867
67.7787 18.1441 9.9427

78.0363 20.0181 8.9512
78.7212 36.1703 -7.9536
87.3477 23.0073 5.0556
86.5006 32.7494 -9.6735
5.6726 0.7982 9.628
8.2739 -2.7648 8.7255
29.2973 1.8768 9.9694
45.4679 -0.6578 -6.9139
47.7433 -1.7051 -10.6347
86.605 6.54 -8.18
91.5905 14.7557 -8.8588
ID=MANjavUNKINDMfNB75152*

LM3=54
0.257899999998406 -2.29649999999947 -10.2796999999997
2.72970000000186 -3.08669999999934 -9.27820000000049
31.9639 -2.1271999999998 -11.6254
36.0050999999995 0.446900000000092 -12.7774999999998
28.2443999999995 7.03459999999992 -14.1271999999999
39.3869999999997 3.65210000000024 -14.4331999999999
38.8582999999993 4.40850000000004 -15.0110999999999
52.0550999999996 0.929900000000075 -1.11009999999983
51.1262999999999 12.7380000000003 -19.9678999999999
55.0011999999998 10.6408000000002 -11.1113999999999
58.6772999999998 9.23370000000023 -13.7937999999999
50.9436 -2.17329999999976 -15.8323999999999
63.818599999999 10.4076000000001 -15.6022999999997
65.9527999999992 4.05790000000005 -13.6350999999997
74.559699999999 12.8057 -13.2598999999997
80.0008999999989 12.9113999999999 -12.8068999999996
81.7956999999987 21.2054999999998 -14.7891999999996
85.4309999999987 21.8399999999999 -6.22389999999956
83.2204999999988 10.7020999999999 -8.83579999999959
86.2788999999989 17.4448999999999 -14.6524999999996
-0.720500000000945 1.64360000000037 -10.0430999999997
1.04429999999941 4.04640000000022 -9.44119999999976
27.3656 17.1917000000003 -11.7399
32.2105999999999 17.3281000000002 -13.2091
35.7066999999997 15.4846000000002 -14.5349999999999
36.3798999999997 14.7059000000002 -15.6416999999999
44.8783999999995 23.3170000000001 -2.36799999999986
52.9819999999996 16.5893000000002 -10.9469999999998
55.8164999999995 19.7893000000002 -13.4963999999998
43.1499999999996 26.2938000000002 -16.0545999999998
60.5035999999993 21.6332000000001 -15.5422999999997
59.5416999999992 28.029 -13.9608999999997
71.0958999999992 24.992 -13.5360999999997
75.7600999999989 27.2016999999999 -12.8248999999996
78.5361999999988 31.0958999999999 -8.35249999999959
82.6748999999988 25.5870999999999 -14.9744999999996
0.0911000000001969 0.257500000000242 0.134100000000086
-0.371600000000136 4.14580000000018 -2.31839999999982
21.1061000000001 10.3386000000001 1.88819999999999
34.6891999999997 21.3417000000002 -9.43279999999996

37.3517999999997 23.2847000000002 -14.2472999999999
35.3813999999999 9.84080000000013 5.3876000000001
59.9274999999993 16.0318 9.71560000000028
71.848299999999 19.2105 9.60120000000034
74.168799999999 34.1554999999999 -8.47929999999965
79.1625999999989 21.0333999999999 5.68790000000038
80.8229999999988 29.4104999999999 -6.79219999999959
-0.0876999999997343 -0.560899999999742 0.0629000000000712
1.73990000000083 -4.14749999999962 -1.98650000000009
23.2388000000004 1.01410000000016 1.93339999999994
40.9746000000001 -2.25959999999976 -9.03879999999991
44.1586 -3.15509999999974 -13.8481999999999
81.3097999999989 5.30509999999995 -8.23089999999965
84.8596999999988 14.4457999999999 -6.37239999999961
ID=MANjavUNKBURMfNB75153*

LM3=54
5.7446 -0.5119 0.3098
9.3704 -1.8382 0.6433
35.9437 -1.4592 -6.242
38.1955 0.4317 -8.2568
30.0862 7.6731 -6.4287
42.9853 4.2141 -10.5565
41.6026 5.501 -11.943
55.4534 1.0071 1.7739
53.0847 13.5016 -17.4746
58.6959 12.1325 -10.3194
61.8976 10.7665 -13.4555
55.5939 -1.5557 -14.729
65.8832 11.2102 -16.0978
69.2375 5.6341 -15.4343
76.9292 13.5708 -16.1474
81.1801 14.8098 -17.1299
84.3059 22.6125 -19.1559
89.0546 23.6351 -9.8247
87.2049 12.6225 -14.2348
87.9704 19.2742 -19.7384
4.6184 2.8278 0.4343
6.6839 5.9908 0.6795
30.4345 17.7304 -6.1753
33.6991 17.7087 -8.09
39.013 15.8338 -10.1996
39.0859 14.4713 -12.0877
46.9491 24.0339 2.0516
56.6775 16.8373 -10.1758
59.3739 19.755 -13.5884
47.4501 26.979 -14.9501
62.6371 21.6517 -16.0723
62.6367 28.6055 -15.337
73.2056 26.1562 -16.7031
77.0868 27.5513 -17.1731
81.0054 32.4252 -14.3203
85.3219 27.3024 -20.1204
5.5302 1.2539 9.0999

6.1478 5.8679 7.0309
27.7561 11.7933 9.7667
38.2955 20.9351 -5.7752
41.4954 23.6171 -11.383
43.5256 11.161 11.4408
67.9191 17.9489 9.444
79.5414 21.0614 6.7077
77.7475 34.5079 -7.5899
86.5186 22.9426 2.1695
83.5946 30.6881 -11.7173
5.9469 0.4469 9.2536
8.3629 -3.1862 6.8657
30.2623 1.5087 9.5288
44.2325 -1.606 -5.0941
48.4224 -1.8125 -11.4063
84.639 8.0013 -7.3649
88.3633 14.7277 -11.4188
ID=MANjavMALINDAMNH102041

LM3=54
5.82890000000559 0.0971000000010979 0.0683999999996681
8.21890000000556 -0.369399999998859 0.28569999999966
34.190900000003 -0.506899999999294 -5.73030000000003
37.3634000000029 1.8524000000007 -8.21630000000004
33.1360000000037 8.39710000000077 -8.66560000000012
41.4990000000028 5.14070000000057 -10.8055
41.1245000000028 5.95830000000056 -11.1532
52.2186000000027 0.0686000000004637 1.23660000000006
52.4154000000031 13.0620000000003 -16.3225
55.0302000000027 9.53840000000027 -9.6890999999999
59.6944000000028 9.63110000000025 -12.5234999999999
53.0928000000028 -1.31059999999949 -13.6133999999999
63.0884000000031 10.1292000000003 -13.9938999999999
65.2785000000032 4.92110000000035 -13.1573999999999
74.9879000000035 11.9648000000003 -12.4900999999999
79.7884000000038 12.2727000000002 -11.4849999999999
81.091100000004 19.9972000000001 -14.2396999999999
84.6923000000043 21.2644000000002 -5.94029999999988
83.8764000000041 10.6005000000003 -8.58479999999989
85.4413000000042 15.8592000000002 -13.5823999999999
4.95530000000586 3.3217000000012 0.130399999999649
7.19060000000576 5.29000000000114 0.306999999999628
29.038600000004 16.0714000000007 -6.1540000000002
33.9670000000037 16.0083000000006 -8.92190000000016
38.8329000000034 14.9905000000005 -10.8480000000001
38.7639000000034 14.3596000000006 -11.3401000000001
46.1381000000035 23.9312000000003 0.698999999999911
52.649200000003 16.4345000000002 -9.76969999999994
56.8508000000033 18.7799000000002 -12.6895
46.7532000000038 25.6619000000003 -13.9512000000001
60.4590000000034 20.7039000000002 -13.9544
60.1334000000037 26.3113000000002 -13.272
71.0077000000037 24.4208000000001 -12.9158
76.619600000004 26.5207000000001 -11.2851999999999

78.7270000000043 29.8216000000001 -8.74079999999995
82.3499000000043 24.6605000000001 -14.6955999999999
6.63180000000585 2.39500000000114 8.56869999999957
5.94160000000597 6.27550000000114 6.75219999999958
24.3079000000043 10.9902000000008 8.54109999999977
36.4244000000037 20.0595000000005 -6.55200000000017
40.4057000000038 22.3603000000004 -11.3387000000001
36.2678000000034 9.22350000000057 10.4467999999999
60.0698000000033 15.5147000000002 10.6356
71.2785000000038 18.1518000000002 8.78
73.7070000000042 31.3399000000001 -4.66169999999998
79.5154000000042 20.2077000000002 5.21670000000006
79.7152000000043 28.9477000000001 -6.85299999999992
6.53890000000589 1.83870000000113 8.45549999999955
7.97810000000566 -2.34949999999885 6.8997999999996
26.3585000000039 1.92260000000079 8.64759999999983
41.5024000000022 -1.14309999999941 -5.93959999999993
46.6355000000025 -1.68519999999946 -10.4595999999999
79.4674000000038 6.87070000000035 -5.07689999999992
83.9299000000042 14.1259000000003 -6.46699999999989
ID=MANjavFEMINDAMNH107600

LM3=54
5.28939999999712 0.203099999999905 0.283600000000586
9.28290000000114 -1.35299999999984 0.567999999999771
39.6051999999996 -1.53020000000016 -8.80559999999997
42.8015999999991 0.190599999999715 -10.6630999999997
35.7059999999989 8.71659999999954 -12.1994999999998
46.6846999999994 4.34809999999993 -12.9479999999999
46.3762999999988 5.0056999999997 -14.4039999999998
57.6705999999992 0.755699999999748 1.04690000000017
57.9657999999992 14.1963 -22.3206999999999
62.5079999999991 11.0415 -12.1876999999999
67.3945999999991 9.41499999999996 -16.6575999999999
58.1009999999994 -2.90760000000008 -16.8215999999999
72.201199999998 11.1979999999998 -18.2288999999998
72.9567999999982 5.49969999999975 -18.5206999999998
83.7479999999977 12.5727999999997 -17.4706999999997
89.5086999999974 14.0733999999997 -17.9490999999997
91.364199999997 22.2891999999996 -20.9903999999996
96.3752999999968 23.7892999999996 -11.7862999999996
94.2257999999971 11.8130999999996 -15.3331999999997
96.0132999999971 18.7298999999996 -20.3887999999997
5.17399999999782 3.08959999999981 0.133800000000493
6.6772999999982 5.41679999999966 0.17000000000042
34.3986999999992 19.6937999999999 -8.75669999999987
38.3225999999992 18.6965999999999 -11.1197999999999
42.8356999999991 17.5028999999999 -12.7327999999999
42.5404999999991 16.1706999999999 -14.6775999999999
50.8856999999986 25.2897999999999 -0.639899999999795
60.0924999999988 19.4289 -12.1567999999999
63.9326999999986 22.6363999999999 -16.7662999999999
51.1958999999986 29.9893999999999 -17.2571999999998
69.1188999999982 23.5701999999998 -18.2262999999998

67.372399999998 29.2137999999998 -18.1528999999997
79.6929999999977 26.5524999999998 -19.2797999999997
85.0350999999972 28.7661999999997 -18.2086999999997
88.512899999997 32.8377999999996 -14.9532999999996
92.549299999997 27.1304999999996 -21.7300999999996
7.46919999999906 1.88529999999968 9.89530000000026
7.06409999999869 7.02549999999962 7.91650000000034
30.4022999999994 13.8057999999997 8.02760000000012
41.143299999999 22.7213999999999 -8.25159999999983
43.3836999999988 26.0837 -14.0159999999998
45.2248999999993 11.3552999999998 9.94990000000017
70.9858999999981 18.3446999999997 9.10260000000028
80.9180999999976 20.9168999999997 7.34290000000031
82.6256999999973 35.6435999999997 -9.07789999999966
92.4088999999972 23.8126999999996 1.34350000000037
89.794499999997 31.2012999999996 -12.7527999999996
8.63579999999915 1.73289999999969 9.96000000000024
9.54139999999992 -2.36000000000015 8.05400000000007
33.2358999999999 2.34319999999973 7.81910000000001
47.2686 -1.41690000000012 -8.10149999999992
50.4602999999997 -2.97220000000008 -13.5297999999999
89.2115999999976 6.96049999999963 -8.38459999999972
94.2906999999972 15.6217999999996 -11.8749999999997
ID=MANjavMALTHAAMNH167959

LM3=54
8.1658 -0.1163 0.0528
11.6473 -1.8461 -0.3282
39.707 -1.3921 -9.6704
39.9441 0.6188 -10.7145
35.3082 9.0288 -10.5979
44.7062 4.1832 -13.3557
42.9269 4.6397 -13.6355
58.1763 1.0455 -0.257
56.7598 14.9602 -21.2985
61.115 12.9091 -12.0307
64.5361 10.8946 -16.2378
56.419 -1.7096 -17.3679
69.7848 12.4287 -18.0478
71.45 6.0138 -17.5801
81.6997 15.4317 -17.6481
89.1768 15.8244 -16.1608
90.8353 24.733 -18.4346
94.9109 25.5385 -9.2839
94.6481 12.9431 -13.2592
94.851 20.6037 -19.8459
7.4576 3.4819 -0.2796
8.621 7.6322 -0.6295
33.3045 20.081 -9.444
35.2579 18.5919 -11.212
40.5758 18 -13.4608
39.5236 16.6403 -14.1717
50.9739 28.1214 -0.4003
58.8398 19.071 -12.8431

60.714 22.2928 -15.1709
47.985 28.6658 -17.5041
66.4614 24.2182 -18.25
64.8116 29.6272 -18.018
78.0269 27.9921 -17.5934
84.486 31.6895 -16.0805
87.6139 36.389 -12.1196
92.2848 30.4545 -19.3839
6.9342 1.9498 9.9773
6.9878 6.4648 8.0528
28.5295 12.0932 9.0745
40.434 22.9527 -9.3554
44.4658 26.9232 -15.4493
42.5528 10.5277 10.6138
70.3854 18.3534 9.5335
81.2456 21.7166 8.0444
80.6051 38.6595 -8.8957
89.7442 24.3642 3.0023
89.2932 33.7044 -9.5163
7.284 1.3698 9.8259
9.5012 -2.4351 8.1406
30.9962 3.3638 8.7351
46.8813 -1.0494 -9.3534
52.073 -1.9901 -14.9464
88.6566 7.3701 -8.2034
94.8522 16.6976 -10.2517
ID=MANjavMALLAOAMNH87624*

LM3=54
6.30049999999933 0.583500000000271 1.4854000000001
9.2410000000011 -1.16479999999953 1.81559999999971
39.6500000000004 -0.0222999999995636 -5.56940000000009
41.7138999999985 1.73119999999977 -7.40589999999983
34.077400000001 8.69440000000071 -7.65750000000022
46.7495000000008 5.19660000000033 -10.8948000000002
46.503900000001 5.99620000000037 -11.7475000000002
58.2740999999991 0.747599999999995 0.43800000000014
55.1228999999988 13.8322000000001 -18.3566999999998
61.7419999999989 12.7763 -12.6919999999998
65.471699999999 11.0582 -15.1069999999998
59.2116999999994 -1.2102999999999 -15.3242999999999
69.2889999999985 10.9207 -16.1448999999997
72.0275999999987 5.7943 -15.4358999999997
78.6971999999977 12.1765999999998 -15.8431999999995
84.6696999999978 13.2266999999998 -14.9340999999995
86.0585999999976 21.5041999999998 -18.0243999999994
91.4858999999973 22.5658999999998 -9.2676999999994
88.9462999999977 11.1843999999998 -11.8366999999994
90.3823999999976 17.4732999999998 -18.0584999999994
5.77289999999934 3.06050000000019 1.38500000000006
7.57599999999997 6.09930000000031 1.77639999999997
35.1177999999999 18.3704000000002 -5.87170000000004
37.7587 17.5642000000003 -7.58760000000005
43.5765 16.7437000000004 -11.1480000000001

42.7395 16.2248000000003 -11.6432
51.5622999999991 26.4923000000001 0.156800000000143
60.4145999999989 16.7273 -12.9122999999998
62.7163999999985 20.0949 -15.2604999999997
51.8315999999987 28.6346 -15.6919999999998
66.0048999999984 22.7391 -16.3614999999997
65.279299999998 28.7764999999999 -15.2302999999996
75.6525999999977 25.9200999999999 -15.6322999999995
81.5375999999974 27.5361999999998 -15.3164999999995
83.7572999999973 31.2216999999998 -12.4003999999994
87.7403999999973 26.2425999999998 -17.8749999999994
9.15269999999958 2.97060000000023 11.2044
8.12929999999964 6.33420000000023 8.99999999999999
31.9956000000005 13.1137000000004 9.44339999999987
40.7968999999996 22.2458000000002 -5.92949999999997
43.1276999999992 24.3452000000002 -10.8095999999999
44.9349999999999 11.4038000000002 9.55669999999999
68.9422999999984 16.7356999999999 8.90240000000032
78.3941999999979 19.3791999999998 6.78250000000045
79.1739999999974 33.6892999999998 -7.13129999999949
86.6561999999976 21.5051999999998 2.15810000000056
85.9322999999973 29.6564999999998 -8.87099999999994
7.35679999999963 1.73530000000022 10.6639
10.6602000000002 -2.20829999999971 8.72699999999988
33.8757000000008 3.18200000000044 9.6915999999998
47.068 -1.55809999999977 -6.03280000000005
49.7390999999998 -1.82179999999983 -10.8274
85.8188999999979 7.08169999999984 -7.93499999999948
90.2614999999976 15.2241999999998 -9.03869999999941
ID=MANjavFEMTHAUSNM083250

LM3=54
7.0103 -0.1083 -0.0301
10.6746 -1.6813 0.3934
45.726 -1.5449 -7.4245
48.5649 0.0676 -9.2516
43.9416 10.0469 -10.4806
53.1289 4.0669 -11.9483
50.1514 4.2353 -12.7548
66.1101 1.3118 1.1301
64.9563 14.2749 -20.0471
71.8286 13.0879 -10.8201
75.9235 10.6481 -13.6397
70.3922 -2.0761 -16.2727
82.1048 11.4482 -14.9936
83.9639 5.8689 -13.6761
93.3072 13.1152 -12.9351
100.5701 13.4401 -10.8654
102.2658 21.927 -13.396
105.7945 22.5907 -3.3559
104.2592 10.552 -6.9819
107.1095 16.8551 -13.5038
5.9657 3.4062 0.0719
9.0514 6.372 0.4635

41.3937 21.9939 -7.663
43.9565 20.7692 -9.501
49.3221 18.7495 -11.5393
49.4448 17.8174 -13.3521
61.7739 27.1979 1.3428
70.6005 19.2401 -10.959
73.1087 22.3204 -13.7823
63.368 31.5598 -16.1347
79.8693 23.9399 -14.9459
78.8737 30.2191 -13.5627
90.7983 26.4874 -13.53
96.8436 28.9758 -11.5549
98.8013 33.5801 -6.7312
104.3328 28.8253 -13.5898
7.0149 2.7327 10.081
7.9656 7.4612 8.956
33.8965 13.9403 10.6013
47.61 24.9182 -5.8289
52.9883 27.8286 -12.4429
51.2342 10.6002 12.7922
77.3752 17.2292 12.8694
88.937 19.159 12.6756
92.0675 36.1707 -2.1882
100.6327 21.0743 6.628
101.3636 31.4664 -4.2891
6.9521 1.7661 10.1898
10.2798 -2.6761 9.1681
36.6281 1.6982 10.4074
53.1373 -2.1165 -5.9947
59.134 -2.1322 -13.3077
97.9315 5.8695 -2.4802
105.3746 14.7314 -4.1323
ID=MANjavMALTHAUSNM083513

LM3=54
7.367 0.2981 0.0071
9.9014 -2.0082 0.1438
39.2443 -1.1083 -7.66
42.9066 1.2672 -10.0659
37.6241 8.9204 -10.4681
49.2555 4.8251 -12.6883
47.1479 5.5922 -13.5236
60.3157 1.2557 -0.3905
57.3621 13.9573 -21.2278
64.0388 11.7302 -11.9372
67.5142 10.8559 -15.6749
58.7586 -3.1971 -15.0949
72.8692 10.9952 -17.8968
75.7584 4.8269 -17.0648
83.968 12.6151 -17.5099
92.0806 13.9429 -15.83
92.982 21.8974 -19.608
98.0946 23.1851 -10.4818
95.7702 11.1507 -12.9572

97.8874 18.2684 -20.3871
6.5765 3.2891 0.2267
7.3529 5.6445 -0.3135
33.8916 18.9595 -8.0904
38.2521 18.9598 -9.9073
45.6078 18.1993 -12.799
43.8425 16.8978 -14.0403
54.1964 27.226 -0.618
62.1029 19.3318 -11.9114
65.4032 22.5805 -15.5987
50.4195 30.2539 -15.7077
69.6605 23.3598 -17.8502
69.9833 30.8335 -16.6701
80.7667 27.8151 -16.4083
88.0853 29.9482 -16.1971
90.3793 33.2834 -13.7923
95.3893 28.6632 -20.7554
5.624 1.725 10.2376
6.0632 7.0451 8.506
30.947 13.5228 8.8331
43.5449 24.4908 -6.7308
50.8278 30.4662 -15.3687
45.5573 11.0731 10.9103
74.1266 18.5961 8.9912
84.1327 20.3525 7.0684
85.1508 36.6382 -10.1621
94.8297 22.7691 1.0402
92.7814 31.8936 -10.7128
5.6413 1.1283 10.3342
8.6574 -3.3942 8.7335
32.9709 2.3573 8.9339
49.8442 -1.1721 -6.6716
58.8162 -2.9711 -15.2172
91.2547 6.0735 -9.6338
97.1326 15.6178 -9.7516
ID=MANjavMALINDUSNM142460

LM3=54
6.8962 0.4059 0.8458
10.8717 -1.8127 1.3835
45.774 -1.0167 -7.8509
49.5932 1.345 -10.7782
41.654 9.8504 -12.0658
54.3002 5.1744 -13.7103
51.6528 5.5737 -14.4909
70.9188 1.336 -1.5122
67.731 14.9613 -23.4781
72.5112 13.0694 -13.5804
76.3437 10.6276 -16.5786
66.898 -2.3115 -17.2487
80.978 11.1177 -18.4625
83.2222 5.524 -16.5179
92.2133 14.0102 -15.7337
100.11 13.9093 -14.4083

102.4051 22.647 -17.7285
106.4651 22.7556 -7.3016
103.7408 11.5684 -10.7886
108.3885 16.8215 -17.6741
6.0976 2.7361 0.8334
8.627 6.2792 0.9105
40.743 20.7063 -7.9066
44.7022 20.3721 -10.14
50.8127 18.6765 -13.3842
47.839 16.1643 -14.6796
64.7037 28.5772 -3.0124
71.4016 18.8971 -13.5306
74.2493 22.5772 -17.0143
59.996 31.4028 -17.0102
78.3082 24.2648 -18.5564
78.6751 30.9088 -16.3543
90.1228 27.384 -16.3188
96.9991 30.0502 -14.2525
99.3398 33.3938 -10.9767
105.064 30.451 -17.3602
8.581 2.5246 11.1594
7.9433 7.6073 9.4022
36.1047 13.3606 9.9915
51.0968 25.9469 -7.9649
54.3882 28.9743 -14.0264
51.7824 11.4028 10.6486
82.776 18.4429 8.9051
91.4222 20.076 7.9782
93.2844 36.0272 -6.4671
99.1861 21.6095 5.2432
100.7771 32.5064 -8.5869
10.3292 1.4584 11.4699
10.7581 -3.3802 9.5392
38.1671 3.2542 10.0174
56.2931 -1.9425 -8.0467
60.919 -2.5901 -15.168
99.0581 7.0157 -6.486
104.7549 13.445 -9.0767
ID=MANjavMALINDUSNM198852

LM3=54
8.22949999999655 0.501400000000051 -0.173699999999729
11.716299999998 -1.79430000000021 0.0619000000001924
41.3417999999989 -1.23700000000013 -6.78349999999979
43.5182999999988 0.208999999999666 -8.65489999999989
37.0028999999991 8.33249999999968 -8.99589999999991
47.6352999999992 3.66669999999973 -11.5901999999998
46.2607999999994 4.76759999999986 -12.3174999999999
59.9717999999986 0.121999999999648 0.780800000000089
58.533199999999 13.1832999999998 -19.0092999999999
62.2530999999985 10.6034999999996 -10.9183999999999
66.2215999999986 9.72859999999974 -13.8457
60.1352999999987 -2.45780000000029 -15.2016999999998
72.3955999999991 9.19729999999978 -14.8973

74.2891999999986 5.15089999999973 -14.018
81.5395999999986 11.6414999999997 -14.8086
88.2434999999983 12.5892999999996 -12.2296
89.0962999999983 20.0863999999996 -15.7105
93.9738999999979 21.4431999999995 -6.88830000000002
92.7803999999981 9.42299999999957 -9.59750000000002
94.4270999999982 15.2847999999996 -15.662
7.55109999999772 3.04580000000007 -0.0710999999998534
10.3289999999981 6.39550000000003 -0.210099999999938
36.9942999999996 18.5921 -6.78010000000005
40.0153999999996 18.7647 -8.70170000000001
44.7120999999992 16.7569999999998 -11.8092999999999
44.1183999999992 15.6788999999998 -12.7405999999999
53.891399999999 24.9599999999998 0.264700000000001
60.8265999999988 17.0558999999997 -11.2241999999999
63.8126999999989 19.6577999999998 -14.3213
53.4231999999991 27.7954999999998 -15.1086
69.4475999999987 22.7867999999997 -14.7773
69.7176999999987 27.3079999999997 -13.9305
78.9831999999984 24.5404999999996 -14.1821
85.3418999999984 26.7720999999996 -12.145
88.0010999999982 31.2830999999995 -9.84860000000003
91.8463999999982 26.1129999999996 -16.1371000000001
7.68759999999843 1.7146 9.47060000000006
7.40829999999849 5.78240000000003 7.76980000000004
32.4152999999991 12.2291999999999 9.06010000000001
42.7255999999994 22.9821999999999 -5.67810000000001
46.1082999999993 24.7260999999999 -11.3281
46.9105999999989 10.7590999999997 10.7999000000001
70.6669999999984 16.7329999999996 9.27180000000005
79.8324999999982 18.5717999999996 8.19400000000003
83.4154999999984 33.0753999999996 -5.86610000000007
91.167899999998 20.9704999999995 2.45690000000001
89.5670999999982 29.3008999999995 -6.93560000000002
7.18919999999837 0.940599999999975 9.02900000000006
9.20029999999828 -3.0540000000001 7.67420000000007
34.7203999999989 1.23529999999983 8.90110000000008
48.4035999999989 -2.4415000000004 -5.70069999999977
51.8409999999989 -3.13450000000033 -11.6433999999998
88.9405999999983 5.53359999999961 -5.18500000000002
93.420599999998 12.5335999999995 -6.85440000000001
ID=MANjavMALTHAUSNM257682

LM3=54
7.12900000000122 0.456000000001482 -0.0146000000006873
9.66510000000147 -0.809899999998512 0.0669999999993991
33.6544000000014 -0.495699999999108 -5.8416000000003
36.4665000000016 1.36770000000086 -7.59900000000031
31.3282000000017 8.33660000000094 -8.14370000000041
38.8502000000015 4.52540000000071 -9.72370000000025
38.9520000000013 5.75270000000072 -10.5886000000003
51.639000000001 0.651800000000646 0.641599999999895
48.1924000000011 12.3767000000006 -17.1162000000001
53.589800000001 10.5172000000005 -11.173

56.7756000000009 9.90660000000046 -13.5921
51.8300000000013 -0.938999999999294 -13.1666000000001
60.591900000001 10.0043000000005 -15.3955
63.3553000000011 4.94190000000058 -13.6202
70.8787000000008 11.4280000000005 -17.1884999999999
76.3360000000007 12.6878000000005 -16.7443999999999
77.2708000000004 20.0242000000005 -19.5045999999998
83.6992000000004 21.5371000000005 -11.6920999999998
82.0943000000008 10.7875000000006 -14.0997999999999
82.9156000000006 16.0503000000006 -20.2061999999998
6.09390000000144 2.8348000000015 0.285499999999345
6.96070000000144 5.00100000000144 -0.0625000000006614
29.0819000000013 17.0352000000008 -6.14610000000041
32.5648000000012 16.9365000000007 -8.06120000000036
36.3200000000013 15.4452000000006 -10.1412000000003
36.1909000000013 14.1625000000007 -11.1474000000003
45.3916000000008 24.1656000000005 0.754199999999803
51.5935000000009 16.8266000000004 -10.8188
54.0080000000009 19.0158000000005 -13.7717000000001
43.700700000001 25.2226000000006 -13.7059000000002
57.5797000000008 20.0482000000004 -16.0216
57.5742000000007 25.7302000000005 -15.4139
67.8125000000005 23.9082000000004 -16.8443999999999
72.5097000000004 26.4528000000004 -17.2169999999999
76.7266000000003 30.4174000000004 -14.1676999999999
79.4550000000004 26.2553000000005 -20.4786999999998
6.22600000000128 1.2241000000015 8.51359999999927
5.48680000000144 5.23280000000149 7.36959999999925
24.7412000000012 11.213200000001 8.83619999999954
35.0167000000011 21.4489000000006 -5.05120000000031
37.8380000000012 23.4710000000006 -10.1613000000003
40.922000000001 10.4578000000007 10.5937999999997
62.2884000000004 15.2328000000005 8.21869999999996
73.5239000000004 17.4922000000005 4.6453
70.7892000000003 32.9421000000004 -12.4185999999999
81.4339000000005 20.1461000000005 -1.83179999999992
78.3267000000003 29.2291000000004 -13.0113999999998
7.35930000000134 0.829300000001511 8.96719999999924
7.97890000000131 -2.1997999999985 7.32069999999929
27.5877000000012 2.04040000000101 9.04529999999957
40.3134000000013 -1.37979999999926 -4.08160000000021
43.7418000000013 -1.52789999999929 -9.90270000000015
78.2758000000009 6.43150000000064 -10.3535999999999
83.0511000000008 13.0778000000006 -13.0339999999998
ID=MANjavFEMZOOUSNM395132

LM3=54
6.87550000000062 -0.0159999999998264 1.14240000000016
10.1340000000023 -1.4649999999996 1.35299999999965
38.8603000000014 -1.40409999999953 -7.7143000000001
40.4122999999993 0.845799999999755 -8.97309999999976
32.3506000000017 7.96960000000077 -8.14810000000018
43.3724000000015 4.66820000000039 -10.5086000000001
42.9226000000017 5.21330000000038 -11.9766000000001

57.5717999999997 0.990399999999935 -0.04969999999986
55.3910999999992 14.5111 -19.0497999999998
59.2957999999992 12.4627999999999 -11.8599999999998
63.0256999999993 11.2574999999998 -14.6886999999998
54.8646999999999 -2.11689999999998 -14.2705999999999
68.0265999999988 11.8408999999998 -16.4497999999997
70.8832999999989 5.92149999999983 -16.0850999999997
79.2356999999978 13.4940999999996 -14.9024999999996
86.0599999999979 14.4928999999996 -14.6587999999996
86.7800999999977 22.8775999999996 -17.5980999999996
92.1664999999974 24.6599999999994 -8.39999999999958
90.4283999999977 11.5190999999995 -12.1695999999996
91.3505999999977 17.7934999999995 -19.0930999999996
6.1383000000005 2.74380000000011 1.28140000000008
8.52820000000107 5.56850000000024 1.54729999999998
32.9378000000007 19.1766000000003 -6.96609999999998
35.7416000000007 18.3556000000004 -8.57820000000001
40.2761000000007 16.8006000000005 -10.0733
39.4198000000006 16.3763000000004 -11.3031
50.1591999999996 26.6397000000001 0.531200000000154
58.4108999999992 17.0536 -11.8848999999998
60.7392999999988 20.4245999999999 -14.5641999999997
44.8852999999992 27.8949 -13.3057999999998
65.2703999999986 22.7904999999998 -16.3656999999997
64.3600999999983 29.0336999999998 -15.6488999999997
75.470599999998 27.5312999999996 -14.9707999999996
81.9487999999976 29.4943999999995 -14.6677999999996
84.6517999999976 34.5527999999995 -11.9928999999996
88.3807999999974 29.1693999999994 -18.6027999999996
7.02660000000075 2.41050000000012 10.8465
7.16690000000078 5.87920000000015 9.1046
30.1399000000014 13.0918000000004 9.22889999999989
39.0802000000003 23.5507000000004 -7.32929999999993
42.1995999999998 26.2739000000003 -12.2658999999999
43.8466000000006 11.7848000000002 10.0131
69.0678999999989 18.4353999999998 9.52930000000024
77.9204999999982 20.6782999999996 8.64610000000031
77.9724999999977 36.5612999999995 -9.52319999999961
89.4766999999978 23.8997999999995 2.16790000000039
86.1184999999975 33.2816999999995 -8.9953999999996
7.5661000000008 1.29340000000015 11.2937
8.80350000000134 -2.34599999999979 8.5580999999999
32.7235000000017 2.9615000000004 9.21939999999983
45.3557000000009 -1.22169999999979 -6.72739999999999
49.7863000000005 -2.34529999999986 -12.5103999999999
85.487399999998 6.7667999999996 -8.09939999999964
90.6411999999977 13.6574999999995 -8.85799999999963
ID=MANjavMALZOOUSNM395146

LM3=54
5.61529999999785 -0.173899999999151 -0.103800000000652
8.60180000000172 -1.39729999999912 -0.280000000001084
39.8722999999996 -1.81089999999974 -8.30670000000051
42.9131999999991 0.0335000000001754 -10.2957000000002

32.9430999999989 8.5763 -8.34720000000038
45.9950999999993 3.4654000000003 -12.2230000000004
45.7779999999989 4.79020000000015 -13.5228000000003
59.2891999999995 0.649500000000081 -0.483800000000412
58.4418999999995 14.4279000000003 -22.1058000000005
61.2062999999996 11.1662000000003 -13.0718000000005
64.8734999999997 9.85370000000023 -16.3781000000006
56.7673999999998 -1.89219999999974 -16.9045000000005
70.9101999999988 10.3287 -18.4590000000005
73.542599999999 4.7978 -17.3156000000006
82.1412999999989 12.2000999999999 -16.7095000000007
88.1963999999989 12.1793999999998 -16.3685000000007
91.1494999999986 20.9777999999998 -18.7791000000008
95.2362999999986 22.8425999999997 -9.11500000000081
93.2708999999987 11.0958999999997 -11.9328000000008
96.1563999999988 16.8399999999998 -18.5456000000008
5.26859999999855 2.7363000000005 0.0169999999995378
7.32109999999889 5.32170000000031 0.370199999999472
35.1824999999993 19.8587000000004 -7.97010000000049
38.2229999999992 19.0683000000004 -9.67160000000044
42.1891999999991 17.9149000000003 -12.2184000000004
43.1227999999991 17.0609000000003 -13.5730000000004
54.197199999999 26.2110000000002 -1.62190000000053
59.6843999999993 17.8015000000002 -13.0899000000005
62.6004999999992 20.6832000000002 -16.0093000000006
49.634199999999 27.2477000000003 -15.7567000000005
68.400599999999 22.8545000000001 -18.1442000000005
68.1296999999988 29.5434 -16.7213000000006
79.1378999999989 25.9231999999999 -17.0465000000007
85.3456999999987 27.6934999999998 -15.9158000000007
87.8205999999986 31.4263999999998 -11.7352000000008
93.4902999999987 27.0623999999998 -18.7467000000008
7.80129999999984 1.76940000000034 10.6384999999993
7.32869999999942 7.00040000000026 8.95859999999939
29.3188999999996 12.0612000000002 9.24339999999936
41.7547999999989 23.9888000000003 -5.79190000000046
47.791699999999 27.5090000000003 -14.3820000000005
45.2485999999995 11.4589000000002 10.9637999999995
72.543999999999 18.1037 8.3788999999994
80.7202999999989 19.0035999999999 6.81219999999928
81.8263999999987 35.0022999999998 -9.6257000000008
86.7926999999988 20.3667999999998 3.45129999999922
90.2617999999987 29.8568999999997 -9.83530000000076
8.78929999999994 0.957700000000361 10.9728999999993
9.46330000000062 -3.30629999999948 8.86729999999922
32.0627000000001 2.77800000000024 8.97309999999936
47.4978 -2.61449999999971 -6.32230000000031
52.8282999999998 -2.1572999999997 -14.1749000000004
87.961499999999 4.39699999999984 -9.57170000000072
94.3297999999988 13.8830999999997 -9.23880000000078
ID=MANjavMALINDUSNMA49936

LM3=54
7.37179999999627 1.76319999999952 -0.742299999999666

8.5959999999963 -0.23150000000062 -1.06789999999974
27.6820999999973 -0.519900000000445 -8.66379999999977
31.6238999999976 1.80119999999959 -10.8287999999998
28.4625999999976 9.13939999999962 -9.03979999999975
36.4919999999978 5.55859999999962 -11.5025999999998
34.2308999999978 6.06259999999962 -12.9223999999998
44.6678999999979 0.549799999999653 0.623500000000158
42.7820999999983 14.6364999999997 -18.2390999999998
47.4904999999985 12.0543999999998 -12.1869999999998
49.3906999999985 10.3400999999998 -15.3794999999998
45.385299999998 -0.257100000000327 -15.0477999999998
55.6477999999987 11.1431999999998 -15.3964999999998
59.9948999999986 5.24989999999977 -14.1560999999998
64.8895999999992 15.0151999999999 -14.6782999999999
71.6200999999991 15.0192999999999 -14.0743999999999
68.1124999999991 23.9387999999999 -15.9769999999999
76.9762999999987 26.6571999999998 -7.38649999999987
78.040999999999 12.6200999999998 -11.1500999999999
75.1165999999989 19.9078999999998 -15.5780999999999
6.58849999999635 3.69089999999942 -0.78469999999973
6.97519999999639 5.22349999999942 -1.15379999999971
22.2922999999973 17.3457999999996 -8.70489999999973
26.5517999999976 18.6281999999996 -10.6818999999997
32.7486999999978 17.7188999999996 -11.8734999999997
31.1259999999978 16.0984999999996 -13.0655999999997
36.3926999999977 27.8004999999996 1.25780000000023
45.6404999999985 18.5882999999998 -12.5804999999998
46.5665999999985 20.7611999999998 -14.2481999999998
36.6562999999981 27.6077999999997 -14.8834999999998
51.2670999999987 24.1779999999998 -14.7844999999998
50.8723999999985 31.8503999999997 -13.7507999999998
60.3935999999989 28.6263999999998 -14.8928999999999
65.8388999999989 32.4138999999998 -13.8616999999999
69.4496999999987 38.5793999999997 -11.6298999999998
71.4068999999989 30.5893999999998 -16.1654999999999
5.13129999999608 2.94929999999937 7.8497000000003
5.16389999999617 7.24999999999942 6.68360000000029
19.6202999999969 14.2122999999995 8.49970000000026
29.1733999999977 23.2207999999996 -7.67369999999974
31.5323999999979 24.1073999999997 -12.6086999999997
30.2507999999972 10.7403999999996 11.1834000000002
55.563099999998 18.1728999999997 12.4527000000002
65.0891999999983 22.2988999999997 10.6891000000002
61.2127999999985 38.5273999999997 -6.03369999999983
74.8084999999984 25.2429999999997 5.51730000000018
72.1744999999986 34.9152999999997 -8.49699999999983
5.50889999999604 2.03999999999936 8.1650000000003
8.03179999999621 -1.56960000000057 7.11820000000028
23.6938999999969 1.09999999999952 8.81050000000022
38.2744999999979 -2.82420000000033 -6.66729999999984
40.7911999999979 -2.36260000000035 -12.0633999999998
73.7334999999987 7.10069999999974 -4.84899999999988
78.848599999999 15.2988999999998 -7.4362999999999
ID=MANpenMALCHIBMNH389757

LM3=54
4.05619999999677 -0.811300000001157 -9.89879999999984
5.88439999999662 -2.56380000000141 -10.0198999999997
29.6437999999973 -1.43660000000074 -12.7508999999997
35.5253999999975 2.04639999999927 -14.9921999999997
31.9001999999976 9.21109999999931 -16.2385999999998
42.9416999999977 6.93589999999935 -15.1397999999997
39.0649999999978 7.13279999999936 -16.6220999999997
49.1986999999979 0.404799999999332 -0.844899999999658
49.8463999999982 16.3904999999994 -20.7701999999997
55.5170999999985 14.0272999999995 -12.6031999999997
59.3594999999984 10.3273999999995 -14.7217999999997
52.701599999998 -0.868100000000667 -17.6972999999996
63.4944999999988 12.6146999999995 -15.0806999999997
69.0720999999989 7.17559999999952 -13.6531999999997
75.9129999999997 16.3758999999996 -13.2951999999997
80.5299999999998 17.5048999999995 -10.9450999999997
81.9570999999999 25.8284999999995 -12.8729999999998
84.4419999999999 26.9668999999994 -0.783599999999653
86.7307 13.1691999999995 -7.27269999999966
87.0319999999999 21.3988999999995 -12.4978999999997
3.48439999999663 3.5925999999988 -10.0283999999998
3.63769999999651 5.74949999999881 -10.0340999999998
23.1366999999975 17.8196999999992 -12.0490999999999
29.5602999999976 20.3370999999993 -14.6238999999999
38.6792999999979 20.6789999999994 -15.6027999999999
35.1714999999977 18.6270999999993 -16.5474999999999
40.861099999998 27.5980999999993 -0.885099999999883
53.3125999999985 19.6680999999995 -12.7094999999998
55.5510999999987 23.6108999999995 -15.0459999999998
44.1455999999981 29.7402999999994 -17.5132999999999
60.590099999999 24.0087999999995 -15.2153999999998
61.6042999999987 32.2739999999994 -14.1291999999998
71.4233999999995 29.0581999999995 -11.7749999999998
76.9071999999995 30.7969999999995 -10.8258999999998
78.7008999999995 38.2439999999994 -7.18469999999976
83.7841999999998 30.5054999999995 -12.6866999999998
-0.15360000000368 0.0384999999987076 0.528000000000227
2.99789999999637 5.99339999999874 -1.23479999999978
22.3475999999971 13.8164999999991 4.73970000000018
35.3372999999977 25.1076999999993 -11.9088999999999
39.9326999999979 26.9212999999993 -16.9584999999999
35.9972999999976 10.8886999999992 9.49440000000019
60.5313999999987 18.5443999999994 12.7457000000003
71.9423999999992 21.6627999999994 12.0295000000003
72.2039999999992 37.6437999999994 -4.07959999999978
79.1488999999997 23.9654999999994 10.3968000000003
80.9204999999996 34.5413999999994 -4.02659999999977
-0.361600000003659 -0.789700000001277 -0.0609999999997737
3.75139999999639 -4.47940000000126 -2.34189999999975
26.044099999997 0.741399999998996 4.62990000000025
43.1607999999975 -3.85070000000073 -12.7673999999996
49.7946999999977 -2.61100000000072 -16.8771999999996

81.3661999999997 7.79989999999946 -3.90039999999963
86.9716 16.2257999999995 -3.64829999999965
ID=MANpenFEMINDBMNH218227

LM3=54
3.53689999999778 -0.446900000000098 -7.25909999999955
4.16059999999819 -2.7312000000002 -7.03849999999984
24.2809999999991 -0.478900000000169 -11.4749999999999
27.0552999999991 1.84029999999984 -12.9523999999999
24.276599999999 8.20229999999972 -12.2544
32.4244999999994 6.01339999999989 -13.2358999999999
30.3196999999992 6.75789999999984 -14.3836999999999
40.2516999999992 0.0531999999998171 -0.143699999999763
37.8950999999991 14.2636999999999 -18.2932999999999
44.1145999999992 12.8421999999998 -11.9765999999998
46.0403999999993 11.2335999999999 -14.4908999999998
41.0976999999993 -0.0157000000001163 -16.1687999999998
51.3337999999992 11.6404999999999 -14.5542999999998
54.9792999999991 5.17639999999985 -13.7878999999997
58.9952999999988 14.7584999999999 -13.4734999999997
63.0244999999986 15.5470999999998 -12.8909999999997
63.8783999999986 23.1367999999998 -14.0215999999997
68.8358999999983 26.4350999999998 -3.82479999999974
70.8858999999984 12.6391999999998 -8.69659999999964
68.1416999999984 18.7431999999998 -13.2342999999997
2.50839999999825 0.821099999999711 -7.55019999999994
2.76279999999824 3.42989999999969 -7.92749999999992
19.0090999999987 15.6471999999997 -11.7184
22.3343999999988 17.0364999999997 -12.8193
28.428399999999 16.2396999999998 -13.4982
27.3534999999989 15.2359999999998 -14.3732
30.1732999999987 25.9068999999997 -0.509399999999991
41.5816999999992 18.7795999999998 -12.2776999999999
42.4808999999991 20.3374999999998 -14.4630999999999
31.7235999999988 25.3732999999997 -16.0208
47.276899999999 23.3300999999998 -14.2809999999999
45.5141999999988 31.2017999999998 -13.3276
54.4247999999988 27.4712999999999 -14.0315999999999
57.8922999999987 29.9271999999999 -13.0556999999999
57.9556999999985 29.6944999999998 -12.9798999999999
61.9448999999985 37.2874999999998 -8.0451999999998
-0.208700000001903 0.0115999999996604 -0.181499999999926
-0.469180166055349 3.69721091630888 -1.62302047109203
15.4306999999984 10.3099999999997 4.01290000000007
23.4885999999987 21.1279999999997 -10.5231
27.3146999999987 22.0856999999997 -14.4826
24.1769999999987 9.1242999999997 6.68100000000008
49.8377999999987 18.7229999999997 11.4600000000002
57.4910999999985 21.0701999999997 10.2399000000002
55.7448999999985 36.7418999999998 -3.09049999999993
67.1368999999982 24.5612999999997 5.95320000000023
63.5739999999985 33.9030999999998 -5.84589999999987
0.13264609168495 -0.940630986680272 -0.0644859838677869
1.97706739682538 -4.23916538177253 -1.08328548483932

19.0524999999987 1.37909999999974 3.77160000000009
32.8933999999992 -1.53720000000016 -10.9453999999998
36.3285999999993 -0.335200000000109 -14.7990999999998
67.9609999999986 8.51929999999976 -3.40189999999965
71.1378999999984 16.1084999999998 -6.60589999999968
ID=MANpenMALVIEBMNH341506

LM3=54
2.30659999999952 0.0896000000001319 -7.70779999999988
4.27769999999845 -2.12460000000004 -7.83839999999983
24.8402999999986 -0.329300000000259 -10.2196999999998
28.0130000000003 1.98210000000041 -12.5272
27.3180999999991 9.36619999999998 -12.7106999999999
32.8427999999989 6.07950000000007 -12.6248999999998
29.8208999999994 6.11800000000011 -13.8909999999999
39.823499999999 0.0504000000000976 0.727900000000159
40.1905999999991 14.6696000000001 -17.1032999999999
44.0615999999988 12.5617999999999 -10.4721999999998
45.5382999999992 11.5923000000001 -12.4773999999999
40.726999999999 0.879400000000106 -14.2234999999999
50.8625999999987 12.4847 -12.6106999999998
54.2790999999991 5.87600000000014 -12.0230999999999
58.7243999999986 15.5476 -11.9060999999998
63.8818999999985 15.1499 -10.6958999999998
64.7164999999984 24.5110999999999 -12.2824999999998
69.3799999999982 26.599 -2.29279999999978
71.2770999999983 12.5716 -6.71109999999978
67.4606999999983 20.3858 -12.8808999999998
2.02959999999937 1.57210000000015 -7.7726
1.81879999999915 3.60910000000023 -7.92219999999999
19.0084999999989 16.3011000000001 -10.8560999999999
22.3077999999989 17.0180000000001 -12.4328999999999
29.187599999999 17.2859000000001 -12.3925999999999
26.6680999999991 15.4492000000001 -13.8898999999999
31.4391999999986 26.4171000000001 0.0511000000001237
41.3730999999989 18.5723 -10.2059999999999
42.4353999999988 19.9733 -12.9234999999999
32.0185999999988 24.8517000000001 -14.3708999999999
46.5953999999988 22.4561 -12.9507999999999
45.5883999999986 30.346 -11.7808999999999
55.1706999999986 27.0232 -11.8121999999998
58.5688999999985 30.1431999999999 -10.6276999999998
62.2509999999982 37.8324999999999 -5.94239999999978
65.0598999999983 28.3895 -12.4105999999998
-0.693700000001208 0.166900000000082 -0.0536999999999278
-0.611700000001091 4.38010000000017 -0.521499999999951
14.5916999999986 10.4731000000001 4.55170000000012
23.5250999999988 21.422 -9.43829999999988
27.3861999999988 22.5284000000001 -13.6738999999999
21.5654999999985 7.97120000000003 6.50360000000014
46.8396999999983 17.9517 13.6001000000002
58.7691999999981 22.3986 12.3574000000002
53.4726999999982 37.3337999999999 -2.17519999999979
64.461399999998 25.1588 10.1083000000002

61.8393999999982 35.6058 -3.91379999999979
-0.0836000000012549 -0.846299999999928 0.2662000000001
2.12039999999861 -3.87619999999997 -1.17839999999986
18.8621999999984 1.19129999999997 4.42710000000016
33.144299999999 -2.20409999999987 -9.07249999999985
36.753199999999 0.0264000000001368 -13.5131999999999
65.7165999999983 7.24899999999998 -2.33169999999979
70.9151999999982 15.4372 -3.90499999999977
ID=MANpenFEMUNKBMNH341508

LM3=54
7.93109999999867 1.5316999999993 -1.19609999999998
9.73849999999856 -0.690900000000788 -1.52020000000005
30.7934999999988 -1.82900000000059 -8.74959999999997
33.087999999999 0.154499999999469 -11.2104999999999
29.713999999999 8.87629999999949 -11.1852999999999
38.1454999999989 5.02619999999952 -12.3620999999999
34.566599999999 4.5167999999995 -13.8316999999999
47.6462999999991 0.471099999999593 1.01360000000003
43.1548999999993 13.4902999999997 -19.4617999999999
49.3927999999994 10.9079999999997 -13.4462999999999
51.5363999999993 9.17739999999972 -15.4365999999999
46.1672999999989 -1.69350000000041 -14.6674999999999
56.4634999999995 11.7641999999998 -15.7701999999999
61.1974999999994 4.49869999999976 -16.0723999999999
66.6224 14.8742 -17.1725999999999
70.5907999999999 15.4887999999999 -16.8650999999999
69.9495 23.5982 -19.0667999999999
78.4387999999996 26.2755999999999 -10.8963999999999
77.1242999999997 13.0717999999999 -14.6870999999999
74.7246999999997 18.7452999999999 -19.3473999999999
7.26829999999865 3.23759999999927 -1.44930000000004
7.97779999999866 5.64879999999927 -1.63550000000001
24.7333999999991 19.1439999999994 -9.53009999999993
27.8624999999992 18.9819999999995 -11.3010999999999
33.8768999999992 17.8658999999995 -12.7956999999999
31.5144999999991 16.5007999999995 -14.0267999999999
38.9005999999992 28.0569999999996 1.08190000000008
47.1111999999995 18.4764999999998 -12.6740999999999
47.4268999999995 20.4523999999998 -15.4569999999999
37.0024999999995 26.6452999999997 -15.9447999999999
52.9564999999996 21.5720999999998 -15.9566999999999
52.1251999999996 31.2900999999998 -16.5595999999999
61.9743999999998 26.9876 -18.0070999999999
65.5487999999998 29.0759 -17.2245999999999
69.4777999999997 35.2002999999999 -15.5500999999999
71.1162999999998 27.922 -19.7011999999999
4.85639999999851 2.31169999999923 8.25659999999998
4.91899999999859 6.76719999999925 6.64179999999996
21.9906999999989 13.5738999999994 8.18040000000002
29.7903999999993 24.8568999999995 -9.13969999999992
33.7244999999994 24.9667999999996 -13.9982999999999
32.8014999999989 10.6519999999995 9.8292
60.0145999999993 19.7487999999997 10.9488

69.6845999999994 23.2410999999998 6.96220000000009
64.8106999999997 37.5896999999999 -9.93359999999992
77.1606999999995 25.8194999999998 1.68930000000014
72.8550999999997 33.3381999999999 -11.2346999999999
5.55049999999849 1.68479999999921 8.16909999999997
7.64319999999859 -2.54630000000075 6.81509999999997
26.5832999999989 1.51249999999941 8.31739999999998
41.2753999999991 -2.98630000000044 -7.483
43.4802999999989 -2.05330000000045 -13.4409999999999
75.4416999999995 7.77929999999979 -9.38689999999989
79.6096999999997 16.7460999999999 -11.1573999999999
ID=MANpenMALCHIBMNH389758

LM3=54
1.75190000000078 -1.50889999999989 -7.93570000000059
2.3899000000004 -2.96670000000009 -7.33490000000056
28.0033000000004 -2.94579999999999 -12.3128000000003
32.8867000000004 -0.615999999999961 -14.7299000000002
27.3731000000006 8.93220000000011 -14.5370000000003
36.8655000000006 4.21220000000013 -16.2457000000002
34.0021000000005 4.63470000000007 -16.9588000000002
48.1181000000001 -0.379200000000054 -1.7039000000001
43.8371000000003 14.9831000000001 -22.0039
50.4238000000002 11.1904 -14.2281
52.9521000000002 11.315 -16.7440999999999
48.1397000000001 -1.00509999999999 -19.5109
58.6 12.8284 -17.5060999999999
64.3307999999999 5.82619999999995 -16.7483999999999
69.8082999999997 15.4576999999999 -19.3080999999998
77.2910999999996 15.8004999999999 -16.9386999999997
78.2552999999997 26.6618999999999 -19.6871999999998
81.2198999999997 26.6941999999999 -6.91579999999979
82.1297999999998 12.3298999999999 -15.5232999999998
82.1706999999998 20.9013 -20.0727999999998
0.431700000000633 2.32749999999999 -7.66480000000065
0.397600000000672 3.49370000000002 -7.51370000000066
20.7296000000005 18.7547 -11.6898000000004
25.7775000000005 20.5566 -14.4853000000003
32.9492000000005 19.5283000000001 -16.8416000000002
29.8552000000006 16.6683000000001 -17.2126000000002
38.3073000000002 29.7818999999999 -0.636100000000218
47.0537000000003 21.0428000000001 -14.5964
49.0373000000001 21.9705 -16.576
38.1339000000001 30.1683 -18.7912000000001
54.3246 23.9363 -18.6565
54.9345999999999 33.5172 -16.571
65.0377999999998 30.0167999999999 -19.2339999999999
72.1308999999997 32.6936999999999 -16.8286999999999
73.9828999999996 40.0980999999999 -14.2462999999999
77.7938999999997 31.5377999999999 -19.7557999999999
-0.525799999999423 -0.19890000000002 -0.30380000000066
-1.05290013939997 4.37363353617434 -1.20312958741411
16.6347000000005 11.9363 3.89769999999951
27.5547000000003 24.3043 -12.5728000000002

29.0259000000002 25.0621 -16.3149000000002
23.2538000000004 7.3999 7.17859999999966
58.1557999999999 19.5148999999999 10.6689
69.3388999999998 23.5243999999998 9.28090000000005
65.6015999999996 41.0304999999999 -7.89939999999991
77.7442999999997 26.6549999999999 5.72440000000013
74.3287999999996 37.3180999999999 -9.69559999999984
-0.189999999999405 -1.12110000000002 -0.0601000000006615
2.24580445460609 -4.85796771151317 -0.965058972894279
21.7312000000006 -0.0810999999999934 3.8037999999995
38.7529000000003 -4.39079999999999 -12.3610000000001
40.5238000000002 -4.09649999999998 -16.3152
79.4810999999998 8.27999999999989 -6.44819999999985
83.1792999999997 17.1944999999999 -9.94339999999979
ID=MANpenUNKINDBMNH47326*

LM3=54
6.92589999999875 1.69269999999972 -1.92529999999974
8.34820000000097 -1.14600000000034 -1.85770000000033
30.5341000000005 -2.90369999999993 -9.07870000000014
34.8997000000012 -0.839899999999674 -11.0361000000004
30.7258000000008 9.24360000000016 -13.0978000000002
41.2534000000007 5.09760000000014 -13.9222000000002
36.7578000000009 5.14870000000019 -14.9979000000002
51.5944000000008 0.454500000000226 -0.255100000000246
45.4040000000002 13.4873 -22.3668000000001
52.6496000000006 11.2904000000002 -13.0180000000002
55.3109000000007 8.73130000000029 -16.8190000000002
49.2013000000009 -2.83259999999981 -17.9015000000002
62.2133000000008 10.5116000000002 -17.4669000000002
65.912100000001 4.10300000000023 -17.0024000000002
74.1268000000008 14.3802000000002 -18.6123000000001
81.9015000000009 14.6493000000002 -17.7270000000002
81.1415000000008 24.4365000000002 -21.4863000000002
88.0502000000011 27.1694000000002 -10.3831000000002
86.9982000000013 10.8484000000003 -14.8945000000002
83.824500000001 20.8004000000002 -21.4677000000002
6.22009999999943 3.94029999999988 -1.89700000000001
5.62650000000014 6.92820000000009 -1.91450000000016
24.2574000000006 20.6205000000001 -8.91450000000018
28.8407000000005 21.0290000000001 -11.6711000000001
37.6093000000006 19.3457000000001 -14.3516000000002
34.2267000000006 16.8410000000001 -15.4401000000001
43.5576000000004 30.0551000000001 1.02539999999985
50.7850000000004 19.0865000000002 -12.8986000000001
51.5842000000002 22.5005000000001 -16.7983000000001
38.4839000000003 27.2853000000001 -16.8517000000001
58.1869000000004 23.1297000000002 -18.0450000000001
58.0055000000003 32.3327000000002 -16.9913000000001
68.9350000000005 28.1043000000002 -19.1300000000001
76.1776000000006 31.3774000000002 -17.9240000000001
78.8369000000006 38.5915000000001 -15.8376000000001
81.0993000000007 27.5451000000002 -21.3755000000001
5.08320000000062 3.15790000000003 8.67649999999977

4.73380000000048 8.99050000000006 6.89779999999979
21.5442000000006 15.0219000000001 9.66679999999978
33.2870000000004 26.9496000000001 -9.43300000000013
37.1089000000004 28.3599000000001 -16.0987000000001
34.5467000000007 11.8075000000001 12.5602999999998
64.5992000000009 20.9858000000002 11.0999999999998
76.703100000001 24.6337000000002 8.63539999999979
70.4005000000006 40.3826000000002 -11.1425000000001
84.6847000000011 26.9470000000002 4.6128999999998
81.3170000000007 36.7431000000002 -11.9184000000001
5.5529000000006 2.01069999999999 8.98359999999975
8.93600000000073 -2.9927000000001 7.43859999999976
25.3139000000008 2.11730000000003 9.91809999999977
42.631700000001 -4.77069999999984 -8.93040000000029
45.4342000000009 -3.27569999999985 -13.5114000000003
81.9395000000012 6.14650000000024 -9.82070000000023
87.3064000000012 15.9654000000002 -11.9940000000002
ID=MANpenUNKINDBMNH728c**

LM3=54
4.89620000000126 0.485500000000167 -0.241700000000137
7.53680000000105 -0.706799999999665 -0.674500000000172
29.7359000000006 -1.13469999999979 -6.86230000000003
31.6031000000004 0.757000000000176 -8.57150000000003
29.3052000000005 9.13380000000022 -9.53940000000003
35.5343000000004 4.98930000000032 -9.84700000000003
33.1847000000011 5.44330000000039 -10.7198000000001
46.400300000001 0.529800000000261 1.15309999999993
41.3197000000009 13.4717000000004 -15.2599000000001
49.1049000000006 12.2327000000003 -10.5761
50.3045000000002 10.2370000000002 -13.2784
45.9235000000004 0.266200000000321 -13.1625
53.9651000000005 11.0254000000003 -13.6083
56.2101000000004 4.89570000000028 -13.1001
64.2338 13.5075000000002 -14.5257999999999
67.8423 13.8431000000002 -14.8486999999999
68.9243999999998 21.4624000000001 -15.7018999999999
76.0275999999997 23.5457000000001 -6.90979999999986
73.6375999999998 9.70270000000018 -10.8092999999998
75.0922999999997 16.7071000000001 -15.3949999999998
4.41380000000073 2.58200000000019 -0.34580000000006
5.84300000000049 4.77320000000022 -0.690600000000067
23.9282000000003 17.9474000000001 -6.70790000000001
26.5596000000004 17.4032000000002 -8.00840000000004
31.8828000000003 16.6521000000001 -9.91129999999999
30.7061000000004 14.8619000000002 -10.9182
38.9045000000004 25.7693000000001 2.16559999999998
46.9416000000005 18.2023000000002 -10.5145
46.6027000000005 21.0661000000003 -12.6365
37.8445000000004 26.2157000000002 -12.8078
50.4227000000002 22.4852000000002 -13.3912
48.9072000000002 28.6007000000002 -12.7556
60.2507 26.2549000000002 -14.0871999999999
63.5435999999998 27.4344000000001 -14.5221999999999

65.9465999999997 34.2205000000001 -10.9546999999999
71.3509999999996 28.9165000000001 -15.3820999999998
5.81419999999999 2.06110000000004 8.45900000000004
4.9193999999999 5.94980000000002 7.30340000000006
20.4498000000005 12.1472000000002 9.73519999999995
30.3861000000004 22.9952000000001 -5.90010000000002
32.4185000000004 23.0405000000002 -9.98950000000002
32.5814000000007 9.37660000000022 11.0943
56.7328000000003 17.1875000000001 11.4789
66.5493 19.9672000000001 8.41780000000008
62.2785999999997 36.8397000000001 -5.66599999999988
74.2173999999998 22.7756000000001 4.06830000000014
70.6404999999997 31.7318000000001 -9.37489999999987
6.34830000000004 1.34720000000006 8.69280000000005
7.55800000000024 -2.13359999999988 7.5639
24.1325000000008 0.865700000000259 9.37609999999992
37.7208000000008 -1.84309999999961 -6.02240000000008
39.4198000000007 -0.665699999999597 -10.1010000000001
72.9659999999999 5.6619000000002 -6.27059999999989
76.1800999999998 14.3733000000002 -9.16079999999986
ID=MANpenUNKCHIMfNB17520*

LM3=54
2.22780000000098 -0.193700000000038 -8.82760000000013
5.30859999999969 -2.5067000000003 -9.40140000000008
25.1447999999992 -1.02840000000029 -11.9903999999998
27.218700000001 1.32050000000029 -13.1155000000001
24.7181999999998 9.28719999999987 -14.3602999999998
33.3782999999994 6.128 -12.9798999999999
31.5302 6.15060000000003 -14.6223999999999
41.3447999999995 1.25830000000013 -0.190999999999963
40.4490999999996 15.2032000000001 -18.0728999999998
43.3836999999991 12.8461 -11.2456999999998
45.1644999999995 11.2872000000001 -13.2856999999999
41.2341999999994 -0.0332999999998764 -14.5723
50.754499999999 12.0675000000001 -13.0298999999999
53.4183999999993 5.4132000000002 -12.2309999999999
58.9263999999989 15.1566000000001 -12.3657999999999
63.5820999999988 15.7287000000001 -12.1058999999999
64.7425999999988 23.9221000000001 -12.8767999999998
70.0944999999987 26.0205000000002 -3.61259999999983
69.4142999999986 12.2022000000002 -6.74979999999987
70.7561999999987 20.4500000000002 -12.6163999999999
1.74440000000072 1.63210000000007 -8.95610000000004
1.9179000000005 5.09710000000006 -9.05010000000003
18.3905999999999 17.3316999999999 -11.6001999999998
20.7530999999998 16.9299999999999 -12.4204999999998
28.7506999999997 17.2496999999999 -12.6564999999998
27.5053999999998 15.7936999999999 -14.7167999999998
30.8537999999995 24.3398999999999 -1.9955999999998
41.2325999999994 18.521 -11.3260999999998
41.5717999999994 20.9008 -12.7456999999998
32.1210999999997 27.8857999999999 -13.3676999999997
45.9236999999993 23.1527 -13.3712999999998

44.4665999999994 30.7108 -12.0314999999998
54.9400999999991 27.6961000000001 -11.9058999999998
58.529999999999 29.6255000000001 -11.9208999999998
59.7767999999989 35.8335000000001 -7.62419999999977
66.8687999999989 30.5376000000001 -12.2223999999998
-0.77069999999981 0.541499999999939 0.136399999999981
-1.35979999999965 4.5347 -1.70179999999999
14.0814999999997 10.9815999999999 4.8144000000001
24.6181999999997 24.2349999999998 -9.32029999999975
28.3318999999998 25.6507999999999 -14.1630999999997
24.2207999999995 9.4008999999999 7.89970000000011
45.8451999999991 17.015 12.4304000000002
57.9193999999988 21.2222000000001 11.8100000000002
55.970599999999 38.2162 -2.29619999999978
64.7310999999987 24.1287000000001 9.05630000000016
63.9312999999989 34.3881000000001 -4.94939999999981
-0.522299999999839 -0.603300000000067 -0.289500000000022
2.04769999999998 -3.83300000000015 -2.05860000000001
17.1048999999994 0.905499999999829 4.36570000000006
33.7988999999996 -1.95829999999996 -10.0129
36.6945999999994 -0.171599999999916 -14.8159
67.3774999999986 7.59420000000016 -4.2382999999999
71.9766999999986 16.9499000000002 -6.73879999999988
ID=MANpenUNKFORMfNB18639*

LM3=54
4.7399 1.194 -1.0731
6.664 -1.4718 -1.4492
25.5218 -1.2137 -6.5529
29.2435 1.3735 -8.4299
25.4031 8.9321 -9.7839
34.6185 5.6416 -9.8734
31.941 5.6275 -11.4376
44.7791 1.283 2.1196
39.7709 14.0553 -17.1238
44.3217 12.0092 -9.8432
46.2257 10.3799 -12.5097
42.3346 -0.9502 -12.5941
51.8865 11.2451 -13.3729
54.5521 5.148 -12.8089
62.0639 14.164 -14.4292
66.2037 15.1821 -14.807
66.2982 23.4521 -16.4842
73.7349 26.0272 -8.8693
72.0672 11.4021 -11.4677
72.7056 19.7017 -17.1547
4.4229 2.2842 -1.2723
4.8505 4.6043 -1.2638
19.7366 17.5597 -6.9871
23.3416 17.4961 -8.4919
29.972 17.1829 -10.1517
28.5528 15.6357 -11.4498
34.1501 26.2455 0.5918
42.2177 18.7938 -10.1078

42.5488 20.6696 -12.4884
32.3341 27.033 -12.8969
47.4857 23.4805 -13.3901
45.9284 30.3484 -13.0664
57.2379 27.5238 -14.7522
60.766 29.4909 -14.9898
63.2646 35.2335 -11.6363
68.0196 29.8585 -17.685
3.7372 1.6534 8.3137
2.1958 5.4707 6.3902
18.4931 12.4437 10.4178
28.714 23.1186 -6.9277
32.4433 27.7216 -12.3795
29.0293 10.2218 12.6133
52.2059 18.6695 12.4067
64.2692 22.0886 10.2744
58.8554 38.8033 -7.9918
70.9724 24.4472 6.192
67.5985 35.0222 -10.1396
4.2432 0.6473 8.4204
5.8309 -2.6456 6.6052
22.4173 2.2532 10.3723
36.7214 -0.1238 -6.3828
42.4554 -1.26 -12.3219
70.3401 6.2453 -8.5317
74.918 15.5048 -9.8203
ID=MANpenUNKFORMfNB18640*

LM3=54
5.96779999999919 0.832499999999695 -0.324999999999908
8.14199999999963 -0.985400000000011 -0.513199999999996
30.6065999999999 -1.4307000000002 -6.10179999999998
32.9812999999994 0.579699999999695 -8.00819999999988
30.3457999999998 9.20639999999991 -8.84259999999994
38.4859999999994 5.04899999999972 -9.7032999999999
36.5042999999996 5.6190999999998 -10.8932999999999
47.7865999999995 1.03159999999975 2.5400000000001
45.5061999999997 13.7870999999999 -15.1166
50.4850999999995 12.3691999999998 -9.26519999999992
52.4514999999995 10.5002999999998 -12.1804999999999
47.7691999999995 -0.770400000000177 -12.7705999999999
57.9258999999996 11.6693999999998 -13.8746999999999
59.3499999999993 4.59179999999971 -13.4057999999999
67.3878999999994 14.3194999999998 -14.3440999999999
72.5912999999995 14.5976999999998 -14.8795999999999
73.3088999999994 22.5929999999998 -16.9583999999999
80.3779416469152 24.7443657628766 -9.13340264745534
78.8907999999995 12.4683999999998 -10.8467999999999
79.3008999999994 19.0417999999998 -17.3011999999999
5.39979999999938 2.28079999999981 -0.352399999999933
6.43659999999967 4.84579999999986 -0.208000000000029
25.0606999999998 18.4685999999999 -6.1969
27.5824999999998 17.7874999999999 -7.83589999999999
34.6603999999998 17.3736999999999 -10.0026999999999

33.5040999999997 15.2798999999999 -11.3307999999999
38.1343999999996 25.0161999999999 2.12880000000006
48.7082999999996 18.4322999999999 -9.47489999999992
48.9710999999996 20.8804999999999 -12.2965999999999
39.0748999999997 27.4217999999999 -11.9702999999999
54.6201999999995 23.1664999999999 -13.4325999999999
51.3900999999995 29.6450999999999 -13.3309999999999
63.1802999999995 26.6210999999998 -14.6888999999999
68.2520999999993 27.7408999999998 -15.0115999999998
70.6369999999993 34.2388999999998 -11.3564999999998
75.9579999999994 29.3046999999998 -16.9589999999998
5.66429999999972 1.86129999999999 5.27919999999997
4.80659999999975 6.24439999999999 6.90329999999997
20.6788999999998 11.1046999999999 10.6557
30.8722999999998 24.0276999999999 -5.23309999999996
33.8863999999997 24.5187999999999 -10.0432999999999
33.0745999999997 10.0482999999998 12.7751
58.6189999999995 18.5318999999998 11.7567000000001
70.2577999999994 21.3882999999998 7.35100000000015
67.7061999999994 37.9702999999998 -8.15339999999987
78.4037999999994 23.8882999999998 2.55100000000016
74.5825999999993 32.0530999999998 -9.97129999999983
5.8445999999997 1.38870000000001 5.55549999999999
6.73809999999967 -2.30769999999998 6.52559999999999
23.5896999999997 2.02849999999987 10.6598
38.0080999999996 -3.38400000000019 -4.83909999999993
41.7435999999995 -1.76590000000018 -9.63869999999992
77.0840999999995 6.88859999999978 -9.20129999999986
80.2891999999995 15.8988999999998 -9.49069999999987
ID=MANpenMALCHIMfNB43340*

LM3=54
5.3107 0.5164 -0.6585
6.7457 -1.1369 -0.8832
28.8633 -1.6859 -7.5718
31.6686 -0.0564 -9.8477
27.9673 7.9062 -10.3711
37.0204 4.2286 -11.5553
34.0363 4.4735 -12.7627
46.0188 0.7457 0.3181
42.0105 12.7673 -18.3548
47.0315 11.0588 -11.0555
48.3605 8.9344 -14.3393
43.3265 -1.1088 -13.7088
52.6034 9.5533 -14.8511
55.4178 3.5853 -13.6674
63.1996 12.5198 -14.771
67.471 12.9436 -14.5945
68.5617 21.0201 -16.9966
73.8651 22.7117 -8.3691
73.0154 9.1422 -11.1083
75.4052 16.6863 -14.7832
5.1383 1.9121 -0.8192
5.4979 4.5218 -0.7461

23.2033 16.5585 -7.8895
26.5515 16.6238 -10.4828
33.3463 16.052 -11.4167
31.1411 14.1205 -13.0296
36.8201 23.7426 0.3106
45.2052 16.3589 -10.9638
44.9191 19.2251 -13.3066
35.3825 23.8785 -13.8034
48.9629 20.4882 -14.6507
47.378 27.4251 -13.9021
59.3271 24.3307 -15.1836
59.9448 30.3803 -15.5117
64.7504 32.5785 -10.9605
70.4123 27.8601 -15.3591
4.9572 2.5908 8.269
4.3438 6.2987 6.7764
21.6455 12.5552 9.3634
29.4401 20.9659 -7.7579
31.7503 23.1737 -12.1806
30.6605 8.7279 10.5006
54.4149 16.435 10.1881
64.3801 19.6759 6.6798
61.1964 35.2893 -8.6957
71.4868 22.0565 3.0879
67.5291 31.5206 -9.5068
5.187 1.1855 8.1994
6.9752 -2.4188 6.6701
24.8187 1.6902 8.6761
36.6683 -1.0664 -7.8535
39.1368 -1.7552 -11.9966
71.1556 5.2047 -9.6103
75.0749 13.1548 -9.788
ID=MANpenUNKCHIMfNB75095*

LM3=54
7.89379999999799 1.0470000000001 -1.31569999999986
9.45350000000009 -1.44309999999998 -1.32640000000012
28.8773999999998 -1.54579999999966 -7.85180000000011
31.3779000000006 0.549200000000598 -10.3211000000002
27.9458000000002 9.37130000000043 -10.3576000000001
37.6241000000002 6.69690000000035 -11.5398000000002
34.8951000000003 5.88410000000043 -12.8412000000002
47.0146000000003 0.216600000000402 2.06269999999981
44.7060999999998 14.8442000000002 -18.9025000000001
49.5144000000003 12.1049000000003 -11.0114000000002
51.3712000000003 11.1498000000003 -13.8851000000002
46.4294000000004 -1.71959999999968 -14.4329000000002
57.9563000000005 11.0768000000002 -14.1544000000002
57.0977000000006 3.85220000000026 -14.0416000000002
67.6507000000004 13.5274000000002 -15.4387000000002
70.9839000000005 14.3482000000001 -15.7209000000002
73.3108000000004 22.7896000000001 -17.4862000000002
79.4711000000006 24.9809000000002 -9.01070000000027
78.0837000000007 9.71900000000017 -11.6048000000003

79.7014000000004 17.9903000000001 -17.0616000000002
7.05289999999868 2.99660000000028 -0.50419999999996
7.1576999999994 5.4327000000005 -1.28150000000011
22.6857 18.2128000000004 -7.22270000000017
26.2335 17.8463000000004 -9.72610000000015
34.1281000000001 17.2876000000004 -11.1073000000002
31.9456000000001 16.1148000000004 -11.8573000000002
38.7842 28.0092000000003 1.99549999999984
46.9023000000002 19.8659000000003 -11.2515000000001
47.6581999999999 22.0101000000002 -13.7779000000001
36.7613 28.9913000000003 -14.0081000000001
53.3671000000001 25.5294000000002 -14.3878000000001
49.6802000000001 30.3340000000002 -14.0786000000002
63.2557000000003 28.3778000000001 -15.4492000000002
66.2783000000003 29.4961000000002 -15.7379000000002
69.6149000000003 36.7459000000002 -12.0698000000002
75.5652000000003 30.4144000000001 -17.2344000000002
5.54859999999974 2.30530000000044 10.0623999999999
3.8231999999997 5.71700000000052 7.24069999999988
21.8893 13.6392000000005 10.7800999999998
31.2029000000001 25.7024000000004 -7.57450000000015
31.9390000000001 27.1358000000004 -11.8523000000001
34.1226000000001 11.6550000000004 12.2549999999998
57.9828000000005 19.0369000000003 12.1992999999998
69.9326000000004 22.4260000000003 9.03089999999975
65.2889000000003 39.1449000000002 -7.69400000000022
77.5468000000006 24.5937000000002 3.86679999999974
74.1415000000003 33.6890000000002 -9.76710000000022
5.89649999999978 0.589400000000426 9.98769999999999
6.85989999999987 -3.01159999999969 6.2532999999999
25.9067000000001 2.25020000000036 10.0089999999998
40.0388000000003 -1.58749999999965 -7.88180000000018
41.7150000000004 -2.14009999999966 -13.9882000000002
75.8302000000006 5.80610000000024 -8.09740000000026
79.8410000000006 15.9733000000001 -11.1616000000002
ID=MANpenMALFORAMNH173637

LM3=54
6.62209999999892 1.15449999999926 -0.867500000000146
8.81059999999877 -1.36100000000083 -1.39300000000015
30.0180999999991 -1.71340000000056 -6.60830000000009
33.1184999999992 0.683799999999503 -8.98960000000009
28.6202999999991 8.99429999999949 -10.6690000000001
39.0803999999991 5.84619999999956 -10.5975
36.4157999999992 5.77569999999954 -12.142
48.0671999999993 0.942999999999617 1.75219999999986
46.2613999999992 14.9358999999997 -18.7168
49.9793999999993 12.3102999999998 -10.0659
52.1506999999993 10.7805999999998 -12.4595
47.5016999999992 -1.16500000000035 -12.9240000000001
57.1672999999993 11.4042999999998 -13.5616000000001
58.9353999999993 4.81109999999981 -13.5461000000001
68.6580999999996 14.8901 -13.9350000000001
72.1665999999995 15.3907 -14.3996000000001

74.8015999999994 24.5137999999999 -15.5502000000001
79.8803999999992 26.2299999999998 -7.01230000000011
79.3706999999994 11.6605999999999 -9.24110000000017
80.0824999999993 20.2168999999999 -14.9668000000001
6.18029999999883 2.73259999999918 -0.781400000000196
6.88359999999885 5.75129999999919 -1.30400000000016
23.064599999999 18.6182999999994 -6.81620000000004
26.9663999999991 18.9679999999995 -9.14280000000003
35.2256999999991 17.9410999999995 -11.3406
32.4650999999991 16.4220999999995 -12.4389
38.3950999999991 27.0230999999995 2.67929999999996
47.6300999999993 19.1733999999998 -10.3222
47.8833999999993 21.7030999999998 -12.7173
37.4206999999992 28.6009999999997 -12.6755
53.1722999999993 23.5710999999998 -14.066
49.8503999999993 30.3795999999998 -13.3644
64.0533999999994 28.3150999999999 -13.8539
67.8902999999993 29.4810999999999 -14.0801000000001
70.4148999999992 37.6279999999998 -9.49150000000006
76.3420999999993 31.1110999999999 -15.1787000000001
5.45729999999873 2.37249999999913 10.1870999999998
4.7323999999988 7.10009999999917 8.21109999999982
21.109499999999 12.6482999999994 11.5827999999999
33.1970999999991 25.1192999999995 -5.77139999999999
36.2898999999991 28.2171999999996 -11.7199
34.757899999999 11.2263999999994 13.6221999999998
59.1511999999992 18.6707999999997 13.1102999999999
68.9417999999991 21.8317999999997 10.7021999999999
64.4623999999991 39.0396999999998 -5.96130000000006
76.0172999999991 24.0021999999998 7.00119999999989
74.4497999999991 34.9220999999998 -7.26480000000007
5.54169999999873 1.42799999999914 10.0838999999998
7.66049999999883 -2.78000000000081 8.28259999999979
24.5702999999991 1.66999999999937 11.5677999999998
41.6266999999994 -2.28820000000041 -5.33260000000015
45.9232999999992 -1.5798000000004 -12.1962000000001
75.6486999999993 5.81299999999984 -6.64330000000015
80.8099999999994 16.7435999999999 -8.24340000000016
ID=MANpenUNKFORAMNH183148

LM3=54
6.944 0.0793 0.0356
8.3762 -1.6553 -0.4794
29.9745 -2.4054 -7.2174
32.8611 -0.0236 -9.8341
29.1866 8.8151 -10.3315
36.9669 4.7821 -11.1866
35.0628 4.702 -12.3604
47.7494 -0.507 1.8111
43.672 13.493 -17.9286
49.0643 11.0319 -11.2807
51.1816 10.1092 -13.7039
47.8541 -2.4461 -13.1482
56.8493 11.4306 -15.0184

59.317 3.6319 -14.0555
67.8836 13.4826 -16.1765
71.9642 13.9803 -16.8531
71.8158 22.1309 -18.7036
80.3039 24.7563 -10.8748
78.998 10.7561 -12.4127
78.2614 18.407 -19.5371
5.8079 2.7173 0.2428
6.5927 4.7573 -0.8056
23.8204 18.0748 -6.7837
27.1328 17.9352 -9.0682
33.6788 16.2884 -11.2418
31.9435 15.6981 -12.209
38.4487 26.3212 1.8254
46.7612 18.0965 -11.016
47.9019 20.1958 -13.4936
38.8253 28.107 -12.9846
53.0875 22.6812 -14.957
50.6025 29.6899 -14.2663
63.4107 26.2905 -17.068
67.3245 29.5407 -16.314
71.7754 35.2936 -12.2155
74.3884 27.9543 -19.2476
6.0287 2.549 9.0801
5.1263 6.0527 7.5453
21.9173 11.718 10.9491
32.7314 23.345 -7.0329
34.5116 25.8419 -11.5896
34.4675 10.6878 11.9542
57.8615 17.9025 11.2087
71.0115 22.5125 7.2552
67.0475 38.1393 -9.1855
80.2928 25.1924 1.4587
74.6533 33.023 -11.131
6.6315 1.3229 9.185
8.3334 -2.4662 8.1051
24.7293 2.2495 10.7414
40.0678 -1.3303 -6.3946
48.0328 -2.4646 -12.1308
76.5925 6.5413 -8.7725
80.599 15.3358 -11.3464
ID=MANpenMALCHIAMNH47851*

LM3=54
5.66310000000027 0.430699999999843 -0.343299999999514
7.98979999999996 -1.5591000000002 0.0121000000003526
30.6520000000006 -2.50270000000006 -6.89229999999987
32.3563000000008 -0.248400000000003 -8.70189999999993
29.5057000000004 8.60419999999997 -8.79939999999977
38.4070000000008 5.05880000000004 -9.61559999999991
35.5017000000009 5.27110000000007 -11.0996999999999
48.9509000000005 1.04399999999996 3.5945000000001
47.4648000000007 13.8034000000002 -17.6010999999998
52.0049000000007 12.3159000000002 -8.8492999999999

54.1076000000008 10.7351000000002 -12.0265999999999
46.5188000000009 -0.946899999999928 -12.2977
59.9815000000007 11.1155000000002 -13.0273999999999
59.8573000000006 4.80670000000009 -13.1491999999999
69.5991000000002 14.5536000000002 -13.7554999999998
75.2709999999999 15.3316000000001 -13.7130999999997
76.3608 22.3958000000002 -16.7689999999997
82.1946999999999 24.8250000000001 -7.73069999999961
80.0301999999997 12.0613 -9.67719999999968
79.3921999999998 19.2906000000001 -16.6108999999996
5.39270000000009 2.2674999999998 -0.473199999999615
5.72950000000005 5.74859999999982 -0.348499999999601
23.5334000000002 17.8594 -7.79039999999966
26.4153000000004 17.3866 -9.91479999999969
35.3276000000005 16.3251000000001 -10.3630999999997
32.3903000000006 14.4296000000001 -11.5917999999997
39.68 26.4757 2.97720000000038
50.6449000000005 17.6792000000002 -9.85599999999979
50.5872000000005 20.1253000000002 -12.3464999999998
37.8300000000003 27.0412000000002 -13.9689999999996
55.8750000000004 22.6348000000002 -13.2553999999997
52.7298000000004 28.1716000000003 -14.6200999999997
66.4529000000002 26.1145000000002 -13.8425999999997
70.9642 28.5236000000002 -13.1483999999996
72.9945999999998 34.5363000000001 -10.5668999999995
77.9158999999998 27.6074000000001 -16.3678999999996
4.02419999999992 2.08819999999977 9.48670000000043
3.71249999999991 6.83519999999976 7.71620000000043
21.973 14.4414999999998 10.6935000000004
34.7607000000002 21.5979000000001 -7.01729999999964
37.9359000000003 27.9074000000001 -12.4280999999996
32.1851000000001 10.0045999999999 13.8803000000003
59.9765999999998 19.3031999999999 11.9498000000004
68.0550999999999 21.5259 10.1021000000004
67.3884999999998 36.8782000000001 -7.40949999999954
78.7769999999998 24.9751 3.96830000000043
75.7575999999998 32.5037000000001 -9.33749999999953
4.33449999999987 1.28359999999975 9.53730000000042
7.07120000000008 -3.10910000000023 7.99850000000038
25.4162000000002 0.839099999999875 10.9297000000002
41.4649000000008 0.52669999999998 -7.08219999999996
47.6886000000009 -3.00549999999999 -11.6803
76.9156999999998 7.73749999999998 -5.51019999999972
81.2468999999998 16.8583 -9.28709999999965
ID=MANpenMALCHIAMNH60004*

LM3=54
5.9091 0.7072 -0.8807
7.5468 -0.8308 -1.1395
32.3293 -1.1731 -9.7878
33.5422 0.813 -12.1572
32.1692 10.7672 -12.85
40.2365 5.6212 -14.252
35.7705 5.822 -15.0497

50.4881 0.5022 -0.1917
47.4835 15.6704 -21.0421
52.3268 12.5448 -13.0085
54.6465 10.8301 -16.1286
48.9241 -0.2963 -18.1037
58.7536 11.6789 -17.3941
60.9876 5.2218 -17.572
70.6973 15.6287 -18.6698
75.0831 16.906 -19.1921
77.5033 25.2551 -21.1231
82.9522 26.1234 -11.2234
81.8055 13.005 -13.961
83.0375 19.8282 -20.7043
4.9048 2.8468 -0.5284
5.1674 4.834 -0.6612
25.6914 19.7813 -9.2392
27.6363 19.2182 -11.7373
35.7604 18.7289 -13.5488
32.7243 16.2302 -14.8427
40.6383 28.0455 0.296
49.7248 19.7705 -12.9167
50.3726 22.6797 -16.103
39.4763 29.6522 -17.1838
54.6397 24.5817 -16.9141
51.9621 30.958 -16.876
66.4645 28.555 -18.3621
70.2009 30.837 -18.736
73.7517 36.5356 -14.8038
78.5211 30.8016 -20.374
6.3574 2.938 10.1219
5.3291 7.4024 8.0219
22.5262 14.7578 10.013
32.2236 26.4789 -9.2051
36.8145 28.1511 -15.3197
35.4473 11.6924 10.8841
61.1486 19.3786 10.4845
71.5601 22.4811 6.7434
68.8789 39.8947 -10.7195
79.8821 25.8654 0.4135
75.7781 34.4308 -11.3017
6.7255 1.5596 10.0479
8.9192 -2.5654 8.3176
26.1297 0.8662 9.7001
41.4529 -3.2556 -10.0336
45.5612 -1.7573 -15.4688
78.1477 6.7109 -10.563
82.6186 15.8789 -11.4861
ID=MANpenMALCHIAMNH60020*

LM3=54
2.66519999999987 -0.816700000000379 -7.95689999999987
5.15980000000149 -2.51099999999984 -7.69560000000007
28.5354000000004 -1.93120000000006 -10.8395
30.8841000000011 -0.0623999999999827 -12.5230000000001

28.5915000000011 9.04169999999999 -15.1254
36.3124000000003 4.29170000000004 -12.7861
32.6063000000012 4.35600000000012 -15.2217000000001
45.9578000000009 0.073700000000076 0.371299999999927
43.9072000000007 13.3282 -19.4999
49.6812000000007 11.9384000000001 -10.6588000000001
51.7253000000006 10.3274000000001 -13.1404
45.2472000000008 -1.84639999999994 -15.6581000000001
57.4563000000007 11.1746000000001 -14.3587
59.0495000000006 4.26670000000008 -13.1654
67.5591000000007 13.4177000000002 -12.6943000000001
71.5371000000007 14.0365000000002 -12.1912000000001
74.4078000000006 22.4301000000001 -14.0408000000001
78.2679000000006 24.6814000000001 -2.62650000000007
77.6631000000006 11.9633000000002 -5.94380000000009
80.2678000000005 18.1153000000002 -12.6071000000001
2.00550000000077 1.5178999999999 -7.95679999999999
2.18430000000151 4.02900000000015 -7.83440000000008
22.2225000000016 17.0618000000002 -10.9905000000001
25.6913000000015 16.9030000000002 -12.8581000000001
32.6505000000011 17.1694 -12.9557
29.2018000000012 14.9795000000001 -14.8075
37.5864000000012 25.4168000000001 0.947899999999914
47.1022000000007 18.2615000000001 -10.4744
48.2597000000009 20.4570000000001 -13.3074000000001
36.7029000000011 27.1639000000001 -15.946
53.8589000000009 22.9637000000001 -14.0181000000001
50.897900000001 29.4240000000002 -13.5579000000001
62.6809000000007 26.5312000000001 -13.0968000000001
67.3105000000008 28.7059000000001 -12.2259000000001
69.1904000000007 34.2501000000001 -6.97670000000008
75.9254000000006 29.9493000000001 -12.4966000000001
0.0890000000010259 0.220699999999987 0.0459999999999741
0.19160000000121 3.56340000000006 -1.12280000000004
17.6904000000015 10.7124000000002 5.98499999999989
30.5230000000013 24.4596000000001 -8.95330000000007
37.4127000000012 28.7895000000001 -12.9792000000001
29.5435000000012 8.71410000000014 8.99129999999993
57.576300000001 18.4306000000002 13.6416999999999
65.3469000000009 19.9636000000002 12.4580999999999
64.5081000000008 36.4017000000001 -2.47310000000008
72.3773000000007 22.4583000000002 9.78809999999992
72.7983000000007 32.3193000000001 -2.58630000000008
0.117100000001035 -0.9301 0.133199999999963
2.61810000000102 -4.2575 -1.27860000000002
20.2454000000012 0.249600000000159 5.39789999999993
39.0200000000008 -3.56779999999995 -9.00820000000006
45.7482000000009 -1.32059999999992 -15.9620000000001
73.8141000000006 6.55570000000017 -2.59290000000007
79.2848000000005 14.9855000000002 -3.13120000000009
ID=MANpenFEMCHIUSNM240168

LM3=54
6.33669999999842 1.0170999999996 -0.85800000000025

8.11649999999832 -1.1968000000005 -0.72400000000022
28.7596999999989 -0.933300000000396 -6.34570000000015
31.122999999999 0.161299999999647 -7.83770000000016
27.8025999999989 9.01589999999968 -9.58310000000016
37.076899999999 5.80479999999966 -9.4343000000001
33.971799999999 5.87099999999967 -10.8397000000001
45.346699999999 1.51789999999966 1.15099999999986
42.986099999999 14.4939999999997 -16.9273000000001
47.9978999999992 12.3382999999997 -10.2637000000001
49.8531999999991 11.5957999999997 -12.7937000000001
45.972999999999 -0.400000000000326 -13.7241000000001
55.3177999999992 12.2656999999998 -12.9582000000001
57.5385999999991 4.87859999999975 -12.5272000000001
65.7190999999994 13.6473999999999 -13.0664000000001
69.7525999999992 15.5586999999998 -13.0798000000001
71.8806999999991 23.2678999999998 -15.2950000000001
76.7461999999988 24.9137999999997 -6.01430000000009
76.398299999999 12.3757999999997 -8.81290000000013
78.0011999999989 19.4198999999997 -14.0471000000001
5.63939999999839 2.41229999999953 -0.806100000000262
5.72789999999839 5.19809999999952 -0.53950000000025
22.6039999999988 17.6792999999997 -6.11070000000017
25.0708999999989 17.6381999999997 -7.97420000000017
33.3296999999989 17.5470999999997 -9.43170000000012
30.4952999999989 15.4082999999997 -10.9177000000001
37.3914999999988 25.3951999999997 2.11329999999984
46.1050999999991 18.5446999999998 -10.0951000000001
47.337799999999 21.1044999999998 -12.3152000000001
36.866699999999 28.0343999999998 -12.2736000000001
52.0484999999991 23.6205999999998 -12.7141000000001
49.725499999999 30.4766999999998 -11.8181000000001
61.2755999999991 27.8398999999998 -12.6139000000001
65.0109999999991 29.2613999999998 -12.7854000000001
68.2306999999988 34.7029999999997 -9.1437000000001
74.2306999999989 30.5642999999998 -14.4202000000001
4.62479999999821 1.74619999999946 8.83939999999976
4.6812999999983 6.0858999999995 7.25819999999974
20.2283999999987 11.9801999999996 10.9567999999998
31.2376999999989 22.8849999999997 -5.49140000000015
32.210499999999 25.7652999999997 -10.3784000000002
32.5024999999987 10.3712999999996 12.3793999999998
54.9254999999988 16.8575999999997 12.1696999999998
65.1379999999988 20.8254999999997 9.63909999999986
63.0366999999989 36.9381999999997 -4.91120000000011
74.2311999999987 23.6562999999997 4.32049999999988
71.7428999999988 31.4944999999997 -5.71540000000009
5.34829999999821 1.35429999999947 9.18039999999975
7.23909999999834 -1.94530000000049 7.31849999999974
23.6751999999987 2.01729999999961 11.0837999999998
38.8422999999992 -0.0344000000003069 -5.97430000000016
41.3491999999991 -1.49330000000034 -10.8451000000001
72.8244999999989 6.43609999999971 -6.64570000000012
76.614199999999 15.9403999999998 -6.77070000000014
ID=MANpenFEMFORUSNM294178

LM3=54
8.40950000000118 1.34749999999951 -0.31390000000006
9.64379999999978 -1.27460000000054 -0.904399999999813
32.1050000000006 -1.50420000000007 -7.38650000000003
34.1103000000006 0.40979999999985 -9.63419999999997
30.3609000000002 8.7734999999999 -11.7885
40.2779 5.85969999999984 -12.0035
36.6360000000002 5.9852999999999 -13.0726999999999
50.8770000000008 1.52419999999991 0.613199999999937
46.8110000000001 13.8322999999998 -19.989
51.9422999999998 12.5675999999998 -11.5093999999999
53.2756000000003 10.6751999999999 -13.5749
49.0129000000001 -2.38970000000024 -14.1711999999999
58.5119999999998 11.4273999999997 -15.0712999999999
59.5445 3.64719999999974 -14.6598999999999
69.7671999999998 12.6538999999998 -17.1548999999999
73.3745999999998 13.0042999999997 -18.4492999999999
74.7988999999997 21.8063999999997 -20.1266999999999
83.3468999999997 24.4741999999996 -13.2550999999999
80.4415999999999 10.2413999999996 -14.3752999999999
82.0291999999996 17.5597999999996 -21.2056999999998
7.59260000000102 3.51199999999964 -0.641700000000049
6.96760000000091 6.43809999999979 -1.39810000000013
25.4585000000004 18.5565 -7.29130000000009
28.0627000000003 18.6074 -9.51380000000005
36.6562000000002 17.2757 -11.9444
33.7169000000003 14.8965 -13.1916
41.9904000000001 26.4675999999999 0.32449999999996
50.0101 18.2501999999999 -11.4574
50.5242999999998 20.6669999999998 -13.869
39.513 28.7831999999999 -14.6442
55.1314999999999 22.3750999999999 -15.3177
51.9487999999999 29.4322999999998 -15.1616
65.6785999999998 26.7876999999998 -17.8068
69.2236999999997 27.3562999999997 -18.2991999999999
72.9416999999998 34.4823999999997 -15.7810999999999
78.1661999999998 29.1502999999996 -21.3138999999999
5.83890000000047 2.39779999999957 10.323
4.98960000000062 6.32389999999966 8.03719999999999
24.2600000000005 13.6630999999999 11.4393999999999
34.3559000000001 25.2205 -6.84260000000005
35.8635000000001 28.0473999999999 -11.7285
35.3954000000005 10.7138999999999 12.7498
63.1870000000003 18.5429999999998 11.2205
74.9941000000001 21.7015999999997 6.78430000000002
68.6638999999998 38.9179999999997 -11.3673999999999
82.6573 24.3933999999996 0.810000000000063
77.0176999999998 33.4515999999996 -11.2535999999999
6.32640000000044 1.26229999999957 10.7013000000001
7.52850000000025 -2.56120000000051 8.46880000000011
28.1357000000005 2.0193999999998 11.4953
42.7488 -1.3392000000003 -7.49669999999988
45.3507999999999 -2.79450000000032 -12.7019999999998

78.2402 5.31019999999966 -10.3795999999999
82.2283999999999 14.0088999999996 -12.0772999999999
ID=MANpenMALZOOUSNM307385

LM3=54
8.28949999999942 2.25780000000064 -0.590100000000315
10.0616999999993 -0.119199999999454 -1.50770000000037
30.9826999999995 -0.704499999999696 -7.13360000000025
32.8627999999995 1.35980000000026 -8.85370000000019
30.3505999999992 9.25410000000022 -11.5522000000001
38.9945999999993 5.89620000000016 -10.6132000000001
36.2053999999993 5.81150000000015 -11.8822000000001
46.8491999999992 0.84750000000009 1.64369999999995
45.4259999999987 13.8644999999999 -18.7641999999998
49.3944999999988 11.7269999999999 -9.71539999999982
51.1121999999987 10.0627999999998 -12.5453999999998
46.4300999999991 -2.18279999999999 -12.7532
55.9438999999985 10.8071999999998 -13.7933999999997
58.7792999999985 4.57389999999977 -13.2136999999998
67.1884999999977 12.8947999999995 -14.0467999999996
70.0766999999974 13.2006999999995 -15.5685999999995
72.039899999997 21.7174999999993 -16.5801999999994
78.7691999999966 24.0553999999993 -8.89559999999935
77.0210999999971 10.1147999999994 -10.2599999999995
78.182399999997 17.7672999999994 -16.9342999999994
8.29809999999929 3.92590000000056 -0.813800000000406
7.94619999999923 6.69560000000054 -1.59800000000037
25.4867999999991 18.2247000000003 -7.42980000000006
27.8916999999991 17.6332000000002 -9.4702
35.6329999999991 17.3457000000001 -10.7163999999999
33.2620999999991 16.0477000000002 -12.2915
39.0327999999987 25.5716 1.99500000000021
47.1083999999986 18.1071999999998 -9.80859999999973
47.9897999999983 19.9293999999998 -12.7642999999996
36.9044999999983 27.2982999999999 -12.7176999999997
53.131599999998 22.6703999999996 -13.2287999999996
51.2127999999977 29.2571999999996 -12.8976999999995
62.6981999999972 26.3811999999994 -15.3273999999994
65.4543999999968 28.7864999999993 -15.8335999999993
69.1885999999964 34.9898999999993 -10.8145999999993
74.1541999999966 28.6694999999993 -17.0724999999993
5.89969999999916 2.29270000000053 9.08819999999956
5.08109999999913 6.30440000000054 7.3204999999996
22.9402999999993 12.7029000000004 10.9574999999998
34.0528999999989 22.5229000000002 -6.57089999999986
36.8076999999986 26.9501 -13.0673999999998
32.8418999999993 10.0257000000003 12.8018999999999
58.2563999999983 17.0749999999999 12.4263000000003
69.5684999999974 20.5043999999996 9.40670000000047
65.4155999999966 36.0987999999994 -6.5181999999993
77.7474999999968 23.3724999999994 3.44200000000058
73.2918999999965 32.7410999999993 -8.12529999999929
6.22659999999917 1.29440000000054 9.32909999999958
7.63319999999928 -2.53469999999944 7.87519999999955

26.1194999999993 2.33080000000039 11.2412999999997
40.8329999999995 -0.318699999999824 -5.80330000000018
46.1600999999993 -2.43309999999989 -12.4020000000001
75.1169999999975 6.99039999999956 -5.71089999999962
78.581699999997 13.1722999999994 -7.41989999999947
ID=MANpenFEMZOOUSNM308733

LM3=54
6.1702 1.3618 -0.546
7.7349 -0.9345 -1.0034
27.742 -0.8185 -6.7626
28.829 0.9605 -8.0923
25.9264 8.6302 -9.3367
34.0707 5.4989 -10.0374
32.0586 5.7769 -10.8568
43.0442 0.0517 1.471
39.9042 13.3766 -16.3722
45.6633 11.4244 -10.6915
46.9997 11.1372 -13.1273
43.4365 -1.1967 -14.0281
52.2287 11.1623 -13.7999
55.062 3.1965 -12.3388
62.1226 13.8454 -14.7511
67.0861 14.3614 -14.9762
67.4516 22.5674 -17.3067
74.063 24.8633 -7.5731
74.0651 11.9141 -10.8112
73.0215 19.4886 -17.6616
5.4391 2.38 -0.3637
6.0963 5.2246 -1.0123
21.9522 16.5229 -6.6872
23.9014 15.5847 -7.8369
30.5357 15.6754 -9.7593
28.8797 14.3672 -10.7975
33.2756 24.0788 1.5018
43.8326 17.4959 -10.6631
43.8881 20.0703 -12.6855
34.0406 26.4252 -13.6886
47.9847 22.2204 -13.7101
46.2267 29.6348 -12.7253
56.9884 26.4281 -14.4027
61.2565 29.9049 -14.8862
65.7816 35.3826 -10.3744
69.7433 29.5633 -17.4439
4.6463 2.304 7.9523
4.1591 5.454 7.0015
20.6703 11.2065 10.1848
29.074 20.8293 -6.3729
30.0725 23.8745 -11.0639
29.9292 9.0581 10.9089
52.9668 16.4122 11.7884
64.8211 20.1507 8.9995
60.2439 37.2281 -6.2582
72.6187 23.6983 4.0211

69.1667 32.3496 -8.1085
4.6871 1.1128 8.0981
6.8913 -2.2321 6.5931
23.2327 2.1915 10.0185
36.0592 -0.2702 -7.0575
38.4474 -1.5111 -11.9456
71.1588 7.2063 -6.6495
74.387 16.1214 -8.1664
ID=MANpenFEMFORUSNM308864

LM3=54
7.78680000000126 1.46519999999996 -0.642600000000126
9.78289999999978 -1.48220000000017 -1.56459999999994
31.0983000000004 -1.99599999999982 -7.80040000000011
33.1593000000004 -0.137199999999921 -9.56090000000004
29.2267000000002 9.03630000000011 -10.384
40.4968999999998 5.94730000000005 -11.9034
37.7836 5.99650000000008 -13.0105999999999
49.2110000000006 0.546800000000189 0.401
46.9940000000003 15.0917 -19.2255999999999
51.9452999999999 13.0773 -11.6841999999998
53.3890000000004 11.6030000000001 -14.2446999999999
48.6058999999998 -1.89089999999999 -14.4431999999999
59.6652 11.5525 -15.1682999999998
61.0301 5.51970000000001 -15.3609999999998
70.2291000000002 14.3632000000001 -17.1434999999997
73.8545000000002 15.9721 -16.9538999999997
75.8003000000003 24.0637 -18.6642999999997
83.1750000000003 26.4217 -9.77419999999962
81.6279000000003 11.5062000000001 -11.9552999999997
82.6755000000002 19.8823 -18.5524999999996
7.4695000000011 2.87210000000007 -0.720200000000119
7.81860000000103 6.2162000000002 -1.60290000000017
24.8011000000005 19.4452000000002 -7.47280000000011
27.7373000000005 18.9038000000002 -9.42070000000008
36.5233000000003 18.2273000000001 -11.4581
34.5999000000003 15.9858000000001 -13.4801
39.5006000000005 26.5753000000001 1.57040000000003
49.8554000000002 19.1563000000001 -11.1891999999999
49.7769000000002 21.4344 -14.0454999999999
38.7562000000004 29.0273000000001 -13.8348999999999
56.0095000000004 24.6557 -14.6550999999998
52.4725000000005 31.0124 -14.4159999999998
65.3166000000004 28.6519 -16.4527999999997
69.0083000000005 29.7057 -16.8167999999997
73.6789000000006 37.376 -12.0869999999997
78.0134000000005 31.212 -18.6564999999996
6.34670000000056 2.2428 10.3318
4.46820000000077 5.90560000000009 7.89059999999994
23.7732000000006 14.0792000000002 10.8124999999999
34.0027000000004 24.2517000000001 -7.87880000000004
36.6568000000004 28.7847000000001 -13.0872
35.4335000000005 10.9123000000002 12.1120999999999
61.1981000000006 19.0376000000001 12.4182000000001

73.3312000000005 22.7125000000001 8.60450000000022
67.3821000000005 40.2148 -8.0896999999997
81.3264000000005 25.4571 3.19640000000033
76.1263000000006 34.6507 -9.22729999999967
6.69610000000053 0.63800000000001 10.3616
7.49090000000026 -3.12360000000006 8.06350000000004
27.1264000000004 0.854500000000147 10.6564999999999
41.3447999999997 -0.773200000000052 -8.08749999999994
46.1194999999996 -2.59120000000008 -13.5094999999999
78.8291000000003 6.26750000000007 -9.24169999999969
82.4581000000003 17.0825 -8.85649999999964
ID=MANpenFEMFORUSNM308865

LM3=54
6.66799999999775 1.113099999999 -1.14669999999954
8.55199999999761 -1.29910000000101 -2.41799999999959
28.0123999999981 -1.46840000000083 -7.33469999999971
29.5850999999981 0.44749999999922 -9.33159999999972
27.3780999999979 8.86739999999921 -10.7923999999996
35.2353999999983 5.33089999999932 -10.8758999999997
31.4562999999982 5.12559999999928 -11.9616999999997
45.2565999999982 0.298599999999277 1.26810000000023
40.9949999999981 13.0642999999993 -18.3565999999997
46.6683999999983 11.3550999999994 -11.0051999999998
48.4273999999984 10.1551999999994 -13.0357999999998
44.4925999999983 -1.26650000000069 -14.7383999999998
53.6850999999983 10.9626999999994 -14.2246999999998
54.7518999999982 3.49559999999928 -13.4173999999998
62.7516999999983 13.3534999999993 -14.8788999999998
67.5464999999981 13.8985999999993 -16.6176999999998
69.4406999999978 22.1198999999992 -19.2545999999997
75.5877999999975 24.2143999999991 -9.95609999999966
75.0163999999976 11.1266999999991 -12.9620999999997
75.9994999999976 18.3821999999991 -19.0986999999997
6.66399999999757 2.82499999999899 -0.936599999999551
6.77809999999753 5.56619999999899 -2.02259999999954
22.3225999999977 16.8478999999992 -7.17149999999956
24.8340999999977 16.8346999999992 -8.79989999999958
31.9352999999979 16.2234999999993 -10.7478999999996
28.6334999999979 13.9179999999993 -11.5016999999996
36.9466999999978 26.2186999999993 0.85640000000041
44.446899999998 18.1823999999993 -10.4789999999997
44.8797999999979 20.5731999999993 -13.1801999999997
35.6068999999977 27.4122999999993 -14.6192999999996
49.6045999999979 22.5210999999993 -14.2227999999996
46.4029999999975 29.0160999999992 -13.5569999999995
58.4347999999978 25.8883999999992 -15.4068999999996
62.7295999999976 28.3459999999992 -16.8529999999996
66.6339999999973 34.4442999999991 -12.8183999999996
71.5018999999974 29.4075999999991 -19.2991999999996
5.58569999999748 2.64169999999893 8.74420000000048
4.20639999999733 6.10829999999892 6.55350000000052
20.6822999999975 12.7303999999991 10.6630000000004
29.3273999999977 23.3454999999993 -6.56199999999959

30.5566999999977 24.5551999999993 -11.5554999999996
31.1082999999979 10.6255999999992 11.6419000000004
54.3494999999978 17.6680999999992 11.4761000000004
66.5705999999977 21.2889999999992 8.35530000000035
62.3865999999973 37.9705999999991 -6.52879999999952
73.3316999999975 23.9660999999991 3.12310000000037
69.2585999999973 33.2335999999991 -8.58759999999956
6.07269999999753 1.48929999999894 8.88280000000047
6.54739999999753 -2.30540000000106 6.90110000000045
23.9601999999978 2.26069999999911 10.2788000000004
36.8187999999983 -1.61170000000075 -6.49209999999977
38.6444999999984 -1.90000000000069 -11.5057999999998
72.5716999999976 5.94969999999991 -7.36669999999973
75.7647999999975 15.4810999999991 -8.47759999999997
ID=MANpenFEMZOOUSNM314135

LM3=54
6.4038000000028 1.09180000000048 -0.342800000000472
8.32400000000268 -0.903999999999562 -0.939100000000448
27.5865000000014 -0.296499999999779 -6.74590000000009
29.3811000000011 1.10820000000016 -8.91530000000001
26.7849000000012 8.74080000000019 -10.2175000000001
34.5854000000007 6.38780000000007 -10.3838999999999
31.4826000000007 6.35260000000006 -11.2716999999999
43.5806000000009 0.398900000000002 1.54480000000015
40.4461 13.1688999999999 -17.6675999999998
46.0861999999998 12.0568999999998 -10.5286999999997
47.3704999999998 10.8401999999997 -12.7270999999997
45.1155000000003 -0.942100000000093 -13.1945999999997
53.0499999999996 11.4028999999997 -13.2044999999996
55.3042999999998 4.75189999999971 -13.3000999999996
61.7326999999991 13.9326999999995 -14.7000999999994
66.7752999999989 14.5926999999995 -14.7362999999994
67.1211999999987 22.2905999999994 -16.1415999999993
73.1639999999989 24.3235999999994 -8.0858999999993
74.056399999999 11.6953999999995 -10.0343999999994
73.9012999999988 18.6229999999994 -16.5360999999993
6.30210000000275 2.72200000000045 -0.628100000000464
6.40950000000256 4.96920000000042 -1.46440000000043
21.6743000000014 16.3149000000002 -7.20800000000018
24.1126000000012 15.9069000000002 -8.88550000000011
31.6374000000008 15.8542000000001 -10.7485
28.6630000000009 13.8142000000001 -11.0536
34.1311000000006 24.584 1.53840000000007
44.2381999999998 17.5023999999998 -10.4772999999997
44.4942999999997 19.5137999999997 -12.9731999999997
35.6115000000002 26.3140999999999 -14.0498999999998
49.4155999999993 21.8321999999996 -13.7891999999996
46.9431999999994 28.8508999999996 -13.1101999999996
57.7404999999988 25.5363999999994 -15.0932999999993
61.5480999999986 28.6172999999994 -14.4765999999993
66.1003999999987 34.9306999999994 -10.4832999999993
70.3787999999986 29.7714999999994 -16.2690999999993
4.31080000000284 2.10330000000044 8.67409999999951

3.55900000000276 5.14390000000045 6.30019999999951
19.939900000002 11.8743000000003 9.78539999999968
29.5276000000008 22.2536000000001 -7.03620000000003
30.1560000000005 24.0379 -11.7371999999999
28.9611000000016 9.32600000000018 11.0591999999999
51.8993000000002 17.2233999999998 11.7474000000003
64.2561999999996 21.1036999999996 8.7145000000005
59.9374999999988 36.7794999999994 -7.51319999999937
71.1694999999992 23.6641999999995 3.96590000000063
67.7980999999988 32.3406999999994 -8.62779999999929
4.93250000000292 1.11660000000047 8.59199999999949
6.28270000000293 -2.34289999999952 6.88369999999952
23.0121000000022 2.32430000000035 9.73659999999973
36.9311000000009 -0.311799999999914 -7.1960999999999
38.8864000000006 -1.51520000000001 -11.2327999999998
70.3134999999994 6.62969999999953 -6.93229999999942
74.2280999999989 15.1990999999994 -8.42019999999932
ID=MANpenMALFORUSNM332898

LM3=54
3.69830000000048 -0.672299999999707 -7.6248000000005
5.57700000000041 -1.84719999999956 -7.95330000000062
27.2287000000004 -0.31969999999974 -9.7500000000004
29.7861000000004 1.17860000000024 -11.0528000000004
26.3398000000002 8.69850000000025 -11.1327000000004
36.1393000000001 6.76400000000015 -10.4900000000003
33.3555000000002 6.62470000000018 -12.3390000000003
42.0707 0.637800000000171 2.19799999999977
42.7227999999999 14.3584000000001 -16.0375000000001
47.0878999999998 13.4184000000001 -8.69260000000011
49.6762999999997 12.2153 -10.8626000000001
45.3695 0.0411000000000798 -12.6930000000002
54.4016999999996 12.283 -10.6333
56.7165999999996 5.38220000000003 -10.2481
64.1391999999995 15.0256 -8.93929999999998
67.9523999999993 16.5631 -8.41169999999995
69.4670999999992 24.0853999999999 -9.47819999999994
73.2458999999991 26.0437999999999 0.0568000000000397
73.9934999999994 13.3254 -3.01689999999997
74.1884999999992 20.6742999999999 -8.78909999999996
2.96870000000054 2.51660000000041 -7.76800000000061
3.66960000000048 4.91740000000041 -7.9152000000006
21.9947000000002 16.8254000000002 -9.78600000000038
24.8735000000001 16.8367000000001 -10.9635000000004
32.2985000000001 16.8166000000001 -10.5821000000003
30.1182 14.8321000000001 -12.4761000000003
33.5461999999999 25.3954000000002 1.66319999999972
45.3789999999998 17.8438000000001 -8.45910000000012
46.1599999999997 20.8981000000001 -10.7987000000001
35.3486999999997 27.4351000000001 -12.8321000000002
50.0474999999996 23.9642 -10.4209
47.3193999999995 30.5484 -10.2704000000001
59.1533999999993 27.8637999999999 -9.28079999999999
62.5763999999992 29.52 -8.51279999999998

64.3431999999992 35.6828 -3.87590000000001
70.3940999999992 29.7804 -8.61969999999996
-0.361999999999546 0.145200000000398 0.0483999999999333
-0.55269999999957 4.0693000000004 -1.81300000000061
15.6644000000002 10.6455000000003 5.71089999999951
28.3135 23.1341000000002 -6.5067000000003
31.4951570984267 25.3114744319747 -12.4077834162932
26.7557000000002 8.92940000000027 9.52719999999958
49.6897999999997 17.1052000000001 14.5403999999998
61.3428999999995 20.9515000000001 14.2172999999999
59.4309999999993 37.7504 -0.196800000000042
70.6121999999993 24.9991 9.53699999999997
67.1246999999993 33.9344 -1.30449999999999
-0.208399999999509 -0.736199999999574 0.00599999999936229
2.3923000000005 -3.97399999999958 -1.61660000000063
19.2721000000004 0.616100000000342 5.87809999999949
36.8641000000002 -1.13989999999981 -7.08030000000031
41.2771000000001 -1.11549999999986 -12.4455000000003
70.2296999999994 8.07500000000004 -1.05950000000002
74.0267999999993 17.3809999999999 -0.867799999999971
ID=MANpenFEMFORUSNM332899

LM3=54
6.20379999999971 0.813499999999698 -0.499700000000466
7.96929999999973 -2.21200000000026 -1.24410000000042
34.3008999999999 -2.48230000000016 -8.75940000000018
35.5825999999997 -0.341200000000148 -11.4218000000001
31.7709999999997 8.90189999999984 -11.2120000000002
41.0782 5.47439999999997 -11.6742000000001
37.8314999999998 5.66709999999992 -13.0507000000002
52.8304999999999 0.468199999999886 0.822199999999998
46.7631999999999 13.5815 -20.5655000000001
55.458 11.7747 -12.1152
56.8737000000001 10.6323 -15.019
50.0758000000001 -3.28070000000001 -16.615
62.3003000000001 11.4617000000001 -16.2365
63.8286000000002 3.63570000000006 -15.3682999999999
72.2352000000008 12.3297000000002 -17.9396
78.1183000000009 14.7833000000002 -16.7032000000001
77.7407000000009 22.5515000000003 -19.5797000000001
85.898400000001 25.1787000000003 -11.6825000000002
84.3292000000009 10.8279000000002 -11.9718000000001
85.898600000001 17.4484000000003 -18.8852000000001
5.64799999999971 2.78609999999973 -0.164100000000472
5.83339999999969 5.86589999999973 -1.21120000000047
26.8815999999995 20.2383999999998 -9.11050000000034
29.4966999999995 19.4289999999998 -11.5280000000003
37.2435999999997 17.5726999999999 -11.9239000000002
33.8880999999996 16.0689999999999 -13.1700000000002
44.6776999999997 27.9211999999998 -0.237100000000271
52.9105 18.983 -12.1822000000001
53.5748 21.3998 -14.6708000000001
42.2008999999999 29.2409999999999 -18.1469000000003
58.3236000000001 23.9198000000001 -16.0027000000001

56.3870000000002 31.6961 -15.4742000000002
67.0554000000008 28.7029000000002 -17.5587000000002
73.488000000001 30.0549000000003 -16.8528000000002
75.4869000000012 36.1565000000003 -12.7700000000003
81.0078000000011 31.2063000000003 -19.2551000000002
5.91719999999971 2.6711999999997 10.0744999999995
5.47329999999959 5.82099999999969 7.66329999999951
25.5032999999995 13.9567999999997 10.5427999999996
36.5744999999996 26.4669999999998 -9.29420000000029
38.4166296120645 27.5781527142115 -15.7203746207105
40.2243999999996 11.9492999999997 11.5147999999997
65.9152000000002 19.6455 10.7280999999998
74.9750000000007 21.5684000000001 7.80479999999981
73.1756000000011 38.2287000000003 -7.23010000000031
84.7638000000009 24.1898000000002 0.931799999999805
80.0908000000013 34.5422000000004 -10.6990000000003
6.48409999999972 1.22359999999971 10.7614999999995
8.41709999999967 -2.80830000000029 7.83509999999957
29.0149999999998 1.51979999999978 10.3870999999997
44.712 -2.31520000000009 -8.81700000000007
50.2083 -1.7959 -17.8536
81.4529000000008 6.9870000000002 -7.44500000000001
86.3101000000009 15.7269000000003 -10.9029000000001
ID=MANpenMALUNKUSNM582702

LM3=54
5.2315 -8.2931 -13.4549
11.3207 -4.9914 -11.207
165.0179 -3.2425 2.1716
202.9667 -3.0269 -3.2807
167.664 12.3927 4.0487
229.0313 6.6834 -2.2162
243.802 10.4557 -5.6863
256.9429 -2.8133 18.227
274.4148 23.0078 -17.5866
265.9613 14.384 -2.0189
275.4232 12.9327 -12.5796
280.4564 -7.1267 -6.1344
286.8889 11.4496 -14.7805
309.8299 -0.5071 -11.1871
318.4928 12.0417 -12.8589
325.8945 15.3363 -12.4727
328.6797 25.5923 -17.646
337.697 26.4929 -5.1867
330.1093 7.731 -7.8509
335.1913 17.1079 -21.685
3.9817 9.3612 -13.4485
10.0004 6.9653 -11.0325
167.0471 28.9275 3.1817
199.6563 33.4006 -1.1361
226.7368 27.7692 -0.8882
242.4911 27.976 -4.9682
252.074 41.0715 18.853
264.898 28.5699 -1.7919

274.5652 32.7608 -11.3885
276.3785 52.2934 -5.8109
286.7248 36.2111 -14.8804
307.3969 50.2828 -10.219
317.0766 38.781 -11.7278
325.2353 36.5052 -11.4521
329.7183 44.5364 -7.7408
333.3698 35.0456 -21.5992
-0.1538 1.3783 -0.7819
0.8934 6.987 -3.0188
154.683 18.0766 25.4923
228.3476 37.0669 -1.049
198.5134 36.4448 -7.6791
122.0023 7.3013 25.1002
286.0079 21.9112 31.215
305.5712 23.1532 28.3056
317.9477 48.7772 3.1824
333.9974 25.6926 20.8924
331.588 38.039 -4.6043
-1.4404 0.1009 -0.3473
0.8506 -5.1067 -4.0469
153.6345 2.335 24.8672
229.7739 -2.3313 -2.0276
201.7964 -5.6288 -9.4285
320.2879 0.0684 1.1315
332.6052 11.4295 -4.9628
ID=MYRtriUNKUNKAMNH100068

LM3=54
6.2486999999889 -8.23759999999882 -11.591999999999
13.2119999999957 -3.67580000000097 -8.2696000000011
197.737100000007 -3.37109999999866 4.2620999999996
212.799099999998 -0.130499999999713 2.91340000000014
192.595100000007 16.6586000000006 9.58529999999972
246.276599999993 10.6203999999996 2.35980000000028
261.367699999999 14.0254999999999 -1.38229999999979
270.610899999999 -1.52600000000006 23.5805000000001
294.0025 25.0196000000001 -14.0601
283.998799999996 14.8144999999998 1.23130000000014
293.0334 14.1426999999999 -7.90669999999993
304.101499999997 -7.39000000000013 -6.95509999999983
304.989799999998 10.7813999999999 -12.8221999999999
327.246299999994 -0.712400000000258 -12.1262999999999
335.253399999994 10.1292999999997 -12.7933999999998
344.159499999993 17.0001999999997 -16.4958999999998
344.870099999993 26.5718999999996 -21.7218999999998
357.737399999993 28.7878999999996 -6.90439999999978
348.143899999992 8.85069999999962 -10.1481999999997
349.624099999992 18.9317999999995 -25.3776999999997
3.737799999997 10.7796999999996 -11.5859000000012
11.7511999999976 7.48919999999934 -8.68800000000123
189.012400000007 34.8425000000006 8.15999999999967
212.116800000006 36.9109000000004 4.83079999999975
244.443600000001 31.2414 2.88619999999995

259.771699999999 30.9400999999999 -1.00859999999995
266.016099999999 45.4210999999999 25.7053
281.915699999999 33.2580999999999 1.89770000000001
289.714099999999 34.7462 -7.49930000000002
300.178499999999 56.7157999999999 -4.60499999999998
303.736099999998 39.5979999999999 -11.7629999999999
324.256099999996 53.2849999999996 -11.4666999999999
332.781899999995 42.1090999999996 -12.5841999999999
342.432399999993 37.9327999999996 -16.6179999999998
346.165599999994 45.8255999999996 -8.96429999999988
348.358799999993 36.3782999999996 -25.7532999999999
-0.698800000007041 1.2094999999994 -0.161900000000553
0.780599999995417 8.5821999999941 -2.1028000000085
171.950000000006 21.9521000000006 34.7469999999998
246.918900000001 42.2412000000001 4.87869999999998
212.134800000005 41.4075000000003 0.235799999999728
154.734700000005 11.2394000000005 35.4035999999997
299.552799999997 24.1116999999999 35.4717000000001
327.700699999995 25.8279999999997 30.3985000000001
334.275499999995 52.9523999999996 0.923200000000059
352.459899999993 29.0752999999997 19.7240000000002
346.908099999993 41.3257999999996 -7.16999999999984
0.0764999999920961 -0.429000000000784 -0.484500000000463
2.26469999998937 -7.28420000000086 -1.57830000000011
173.953300000006 5.79580000000074 32.4953999999998
248.308699999996 0.709099999999818 2.35030000000017
214.572300000007 -4.32689999999951 -2.09810000000043
338.373899999993 1.09989999999969 -0.351199999999756
348.893799999993 15.3491999999996 -7.20309999999975
ID=MYRtriUNKUNKAMNH100139

LM3=54
3.01039999996533 -10.0053000000011 -12.6270999999981
8.96969999996581 -6.47330000000063 -10.8581999999981
179.754499999984 -2.94590000000087 1.17080000000114
202.936799999988 -1.69290000000069 -2.7447999999989
163.340399999985 13.8151999999993 0.934100000001117
236.275399999993 7.03969999999948 -6.24089999999908
251.257399999995 11.0427999999995 -10.4559999999992
263.031299999993 0.256799999999428 16.5581000000011
279.870899999998 23.6435999999996 -19.2119999999992
271.435599999996 15.4812999999995 -5.47789999999914
276.484199999997 13.0940999999995 -13.5944999999992
287.296499999995 -11.5469000000005 -12.757799999999
295.401699999998 11.1618999999996 -16.1875999999992
320.112399999997 1.29909999999945 -13.056099999999
328.328099999997 12.8455999999994 -13.177299999999
337.644899999996 17.4174999999994 -14.8329999999989
337.743399999997 27.7840999999995 -19.537999999999
347.202899999996 28.1724999999993 -5.62739999999885
342.853099999995 9.15999999999939 -9.76939999999888
347.325799999996 19.0238999999994 -23.0238999999989
1.38389999996519 7.94829999999917 -15.163699999998
8.366599999966 5.8110999999993 -12.203899999998

177.860299999985 33.1103999999991 0.0203000000010547
200.81519999999 35.2741999999993 -3.78229999999903
236.572599999993 32.2785999999994 -6.7424999999991
251.271299999996 30.5539999999995 -10.9594999999992
257.444499999996 42.7750999999995 17.7291000000009
270.240399999997 30.6006999999996 -5.56689999999918
275.849299999998 34.1044999999996 -13.7416999999992
282.537699999998 59.0592999999996 -12.7305999999992
293.850999999998 37.9637999999995 -15.9527999999992
317.960799999998 51.5197999999995 -12.2322999999991
326.844399999998 41.2315999999995 -12.6811999999991
335.641299999998 38.4181999999994 -14.2589999999991
340.499299999998 46.5328999999995 -9.53459999999902
344.968699999997 36.9377999999994 -23.150899999999
-0.0815000000354063 0.590299999999362 -0.0496999999978676
-0.0150000000357043 6.97329999999914 -3.11529999999783
155.645999999983 20.2572999999991 23.4249000000012
237.261699999994 36.5675999999994 -7.23799999999911
206.972999999991 40.8438999999992 -11.8530999999991
136.438799999979 12.1948999999991 24.3563000000014
280.951399999995 23.3647999999994 28.8911000000011
312.624899999996 24.7766999999994 25.6105000000011
328.718899999998 51.6668999999995 3.59770000000094
340.051199999995 25.9460999999994 20.0723000000012
339.796799999998 40.6030999999995 -7.21359999999899
-0.18040000003537 -0.758200000000788 -0.210699999997863
1.3310831131684 -8.27256503108551 -2.715932511805
159.166499999982 5.19059999999918 23.5229000000013
239.047299999993 2.0687999999995 -8.93509999999909
209.867299999989 -6.01840000000065 -10.8090999999989
329.795499999996 1.08779999999946 2.51000000000106
342.554499999995 16.1042999999994 -8.18089999999888
ID=MYRtriUNKCOLAMNH136248

LM3=54
3.72660000000868 -7.78029999999823 -15.0067999999999
11.6572000000071 -3.31709999999853 -11.4945999999996
180.729600000002 -0.854799999999384 1.91370000000002
211.784100000002 -1.7879999999995 -4.28110000000004
189.969400000003 16.1732000000006 1.27589999999997
247.362500000001 9.97130000000026 -5.1701
262.110700000001 13.4270000000002 -9.58470000000002
277.233200000003 0.318800000000191 20.7567999999999
289.352900000002 25.5123999999999 -18.9279000000001
282.741700000002 14.8942000000001 -4.39600000000005
288.872700000003 13.4809 -10.6842000000001
296.167600000002 -10.1757 -13.8738
304.611700000002 14.1647999999999 -15.6764000000001
328.145000000003 1.29769999999977 -11.7839000000001
336.540700000003 11.1902999999997 -11.7112000000001
347.500500000003 19.1222999999996 -11.4209000000001
349.1118681265 29.9159869070328 -17.5226092111494
357.003164271561 29.9380458563279 -1.53002101828782
348.777689864634 8.76375530723883 -7.05983831127741

357.818919375305 20.4921707960189 -19.8127115025524
1.64910000000583 8.81970000000166 -11.5788999999995
10.1104000000069 7.19870000000167 -9.89519999999956
179.690800000003 32.8025000000008 2.29210000000002
208.288700000002 37.5640000000006 -3.42320000000002
244.101600000002 32.0336000000004 -5.1353
261.413300000002 31.6996000000003 -8.9136
270.542900000003 46.5404000000003 19.9167
281.701500000003 33.5710000000001 -3.2041
287.902900000002 35.3168999999999 -9.6942
290.669400000002 60.7658999999999 -12.5455
303.905600000002 37.1422999999998 -15.4736000000001
324.492200000002 54.0722999999996 -11.1253
334.023900000003 44.9409999999997 -11.0370000000001
346.088000000004 40.3193999999997 -13.0181000000001
347.472800000004 49.4003999999997 -5.94830000000006
355.947049475545 40.6563197653392 -20.2850066387694
0.0571000000069981 0.0863000000016481 0.168700000000434
-0.199899999993182 7.02570000000175 -3.1518999999996
162.875700000003 22.2262000000007 22.9044
245.995600000002 41.7600000000004 -5.16309999999999
213.327700000002 43.5236000000006 -9.8751
135.474800000004 11.5794000000009 24.2372
297.814500000004 25.1081000000001 30.7773
325.541700000004 27.0476 27.0619
331.394700000003 55.7439999999997 2.45949999999996
346.052800000004 28.5026999999998 21.8962999999999
349.949800000004 43.4601999999997 -4.69260000000008
0.0647000000068036 -0.919999999998348 -0.09949999999958
2.32970000000663 -7.90759999999844 -2.88419999999956
166.934200000003 5.89430000000074 23.3134
248.075700000002 -0.475199999999733 -4.76790000000002
218.386700000002 -5.28149999999957 -11.8272
337.542700000004 0.0749999999998607 2.3183999999999
350.641802854971 15.3059783077707 -3.70018221350648
ID=MYRtriUNKPERAMNH147492

LM3=54
4.8093 -8.2585 -11.4284
12.13 -3.9458 -8.7842
200.6692 -3.3579 3.6566
225.0398 -1.6251 0.7
181.6706 13.1986 8.4151
255.5758 7.7984 -0.4487
268.0024 10.2025 -4.998
287.2116 -2.9415 21.5551
295.4456 22.5359 -17.4818
287.4741 13.9532 -0.393
299.5823 12.2209 -7.5683
303.4561 -8.1737 -6.1725
307.7629 11.0451 -12.6665
334.2752 0.2756 -10.6129
339.2867 11.5898 -12.897
348.4256 16.4337 -14.3802

350.9051 25.8016 -20.2648
360.1376 26.8207 -7.6647
353.3353 8.6903 -10.9422
354.2362 17.3346 -24.425
1.8733 8.7144 -9.8303
11.0349 5.5421 -8.161
197.3074 32.5544 5.6693
222.7296 33.4996 2.9769
253.5355 29.352 0.2002
264.8348 28.5264 -4.2134
285.2553 43.1905 23.8503
286.2773 29.125 0.7849
294.6418 32.729 -7.3905
299.2053 53.068 -4.6829
306.7677 36.3089 -12.2287
330.1997 49.474 -10.1634
337.8903 39.4718 -12.3526
347.2415 36.2621 -15.3628
351.0393 43.9002 -10.4501
353.6786 36.098 -24.7975
-1.614 0.4985 -1.3876
-1.8834 6.5698 -3.3234
170.1103 16.4944 30.4591
256.3975 36.1698 1.6955
223.407 37.6482 -3.389
134.8593 8.5037 27.6105
303.422 21.2762 34.9194
325.4327 24.1532 32.0077
339.9463 51.0123 0.0329
357.851 26.3671 17.0059
351.0913 38.9073 -7.5415
-1.4084 -0.8854 -1.325
-0.0658 -6.8328 -3.209
169.6406 4.7191 28.8518
258.8864 2.0999 -0.2307
228.0784 -5.0251 -5.7777
343.9174 0.8753 -2.4096
352.8886 15.4739 -7.1771
ID=MYRtriUNKVENAMNH16137*

LM3=54
1.60620000002206 6.80330000000144 15.1004000000007
10.7665000000222 3.65200000000236 11.2253000000013
169.502600000009 27.692800000001 -9.4921999999995
204.618200000007 33.1687000000008 -3.75499999999957
175.070500000011 12.8385000000011 -7.81799999999951
238.761100000004 28.4024000000006 -1.82769999999955
258.531700000002 27.4049000000005 3.68120000000036
271.716100000002 41.6567000000006 -23.5884999999996
290.029900000001 21.2069000000005 18.9852000000004
279.796300000001 27.8872000000005 -0.853199999999595
292.3683 30.9779000000005 8.67220000000037
290.655299999999 53.1801000000005 5.00900000000023
299.567699999999 33.9663000000004 13.8081000000003

321.715099999997 49.6004000000003 11.4496000000001
332.588199999997 41.5856000000003 11.8694000000001
343.580299999996 37.1984000000003 14.8246
344.804899999997 26.5718000000003 21.2047000000001
356.225199999997 28.2015000000004 7.3348000000001
346.499299999996 47.1870000000003 8.30770000000009
352.806899999996 36.5155000000002 24.4537000000001
1.63410000002232 -10.240299999998 12.0829000000009
10.9187000000221 -5.91519999999798 9.31730000000091
172.977100000011 -3.39769999999887 -11.6057999999995
207.408900000009 -4.36849999999907 -4.38779999999946
241.226200000006 6.61100000000069 -1.29999999999952
262.348700000004 10.9370000000005 3.48540000000046
276.441300000004 -1.51869999999939 -23.0316999999995
281.861400000001 13.1383000000005 -0.863799999999608
294.041600000001 11.2348000000005 8.15980000000042
297.192500000004 -9.07239999999943 6.39300000000049
302.3785 10.0375000000003 12.9495000000003
328.7687 -0.873199999999644 10.6463000000003
336.096899999999 9.40660000000032 12.8191000000002
345.374499999998 16.4745000000003 16.1121000000001
350.533699999999 6.2449000000004 8.49590000000021
352.597799999998 17.9977000000004 25.2286000000002
-0.750499999977665 -0.764799999997947 0.652000000000834
0.889800000022653 -7.368699999998 1.35900000000084
172.941200000012 6.88360000000123 -33.3331999999994
249.259600000005 0.317200000000653 -2.40759999999953
213.247900000008 -8.08509999999913 1.55690000000052
137.248600000014 11.0856000000014 -29.8553999999994
292.764500000001 21.8034000000005 -33.3768999999996
326.620099999999 25.5729000000004 -26.8531999999997
343.2079 0.97240000000044 -1.45449999999969
351.896699999999 28.1848000000005 -17.9372999999998
350.900899999998 13.5622000000004 6.95780000000015
-0.514999999977806 0.936100000002117 0.643200000000782
-0.134399999978359 6.82100000000202 4.00180000000084
170.800000000012 20.9212000000013 -32.0607999999994
243.980000000004 36.8174000000006 -1.56809999999956
207.972200000006 37.4193000000009 2.91500000000046
336.332899999997 51.0985000000004 -2.6727999999999
348.040999999996 39.7025000000003 6.19630000000006
ID=MYRtriMALVENAMNH21302*

LM3=54
5.2201 -10.2169 -12.4726
12.2113 -6.9717 -10.6415
198.8684 -2.7974 0.5686
231.4003 -2.7014 -7.0843
187.5992 13.5942 0.1119
260.2119 7.3186 -5.9221
276.7589 10.7624 -11.0608
286.7984 -0.8868 17.8396
301.1463 20.9723 -22.3525
297.494 14.0044 -4.3876

307.0575 11.1567 -12.6008
312.6346 -11.2222 -9.3173
317.7972 9.5012 -17.3356
343.5066 0.0956 -11.215
350.6848 12.1915 -9.8402
360.3043 15.9071 -12.2472
362.2677 26.7297 -17.4332
371.8359 28.0073 -3.7595
364.0209 7.8191 -7.4763
368.76 18.6023 -19.6345
4.3825 8.2684 -13.1171
11.6389 6.0884 -10.9666
195.9934 31.5383 -0.7475
226.8778 35.5905 -6.2269
258.6001 30.8022 -6.4693
273.4804 28.6168 -9.868
281.1009 43.6884 19.9275
296.2744 29.9032 -4.5641
308.3378 32.5823 -12.023
306.7019 54.7334 -10.7592
315.6819 34.8664 -17.3774
338.5981 49.3983 -12.1152
347.9723 38.683 -11.2082
357.8819 37.2834 -12.6369
360.1076 44.6972 -7.2137
366.6153 36.1072 -20.0193
-0.2561 0.3663 0.4395
3.0732 8.726 -1.0544
182.2605 17.3747 23.4563
262.9668 37.7813 -6.6065
231.7009 41.2506 -16.4311
157.6197 10.868 23.7481
306.7834 22.463 31.7724
334.2859 24.6661 28.7805
350.3202 51.4529 -1.0851
364.5309 25.8308 22.0803
361.5363 38.9582 -4.5201
-0.2256 -0.8588 -0.3572
4.0517 -8.6526 -0.6215
185.2509 7.8093 23.7227
266.1453 0.8072 -5.7395
240.1509 -7.7506 -15.9447
353.6177 0.4735 -1.1829
363.9827 15.5734 -5.36
ID=MYRtriUNKBRAAMNH75348*

LM3=54
3.6537 -9.7852 -13.4192
8.9041 -6.3047 -11.6998
186.9628 -3.9972 0.3171
212.6665 -3.4847 -4.2755
168.6841 12.5904 1.4515
242.0602 6.7883 -5.0752
255.676 9.5625 -7.2453

261.6332 -1.9378 19.0084
281.8003 21.2885 -16.6417
275.3864 12.8622 -3.8188
283.3777 11.6684 -10.6192
295.1422 -8.8228 -8.6525
297.9961 10.3817 -15.6487
321.4413 0.5399 -11.051
329.659 8.8168 -12.7049
339.0412 14.3579 -13.1566
339.9698 25.4972 -20.0433
349.4626 26.9948 -3.135
344.3864 6.2557 -10.5975
346.3854 16.1816 -24.3656
1.9866 6.7099 -15.5564
8.2424 4.1358 -12.859
183.4515 33.1196 -2.0008
211.0291 36.2108 -6.1996
240.6926 31.3566 -5.3681
255.403 29.3995 -7.1028
257.0614 42.2796 20.0919
274.1703 29.0073 -3.8006
281.4349 31.1821 -10.212
290.2274 53.2974 -10.6735
295.9494 34.5743 -14.8951
317.6799 48.5433 -10.9734
326.7912 40.2105 -13.1998
337.5056 37.3439 -14.0493
340.6798 45.5611 -11.3279
343.3197 34.9457 -24.7613
-1.5517 0.4957 -0.2514
-0.1721 6.8638 -3.4998
156.1625 20.326 25.1236
240.9892 38.2532 -3.1273
212.078 40.0475 -10.4515
121.3992 10.0193 23.5259
279.1733 19.718 31.4069
314.9053 22.9608 27.3941
328.3066 51.1154 1.6343
344.4248 26.4674 16.9296
340.8883 40.1981 -7.5335
-1.5894 -0.8204 -0.198
0.5536 -7.1742 -1.9466
157.4866 5.1943 26.5912
242.2343 -0.6414 -3.4336
216.5344 -6.6917 -9.0014
331.0282 -0.2655 0.7643
342.9214 13.5127 -8.8338
ID=MYRtriFEMPERAMNH75983*

LM3=54
7.50120000003423 -10.4639999999988 -11.6104999999999
16.3405000000318 -5.41629999999956 -8.75030000000123
194.448400000016 -3.37739999999952 1.88909999999981
217.529500000014 -2.3242999999996 -1.79880000000017

184.281500000018 15.1677000000005 2.86019999999962
245.04910000001 9.23010000000006 -4.52479999999989
259.63240000001 11.2248 -8.08099999999976
268.429800000008 -2.61680000000033 21.7087000000003
284.661300000006 22.2173999999995 -16.5880999999996
281.360000000007 13.3074999999997 -3.40469999999959
291.927900000006 12.0889999999995 -10.4882999999996
301.536871631871 -10.0400618677586 -7.59056430534215
308.973300000004 10.2243999999993 -14.3249999999995
329.918900000004 -0.950800000000697 -12.2161999999993
338.143300000004 9.04869999999913 -12.3245999999993
346.994400000003 14.9289999999991 -13.9772999999992
349.675200000004 26.3082999999991 -21.4121999999992
356.389100000004 27.404299999999 -2.08239999999921
351.594000000002 6.21919999999896 -7.75169999999923
358.591613141129 16.843310120204 -22.8201755898803
5.54810000003407 7.20340000000033 -13.3634000000008
14.8746000000327 4.15360000000023 -9.46990000000084
193.920700000017 36.5660000000005 -2.03480000000033
213.259400000016 37.2573000000006 -4.61390000000024
243.480500000012 30.0275000000002 -5.54760000000002
257.63610000001 29.5845999999999 -8.6299999999998
268.262600000011 46.6326999999999 20.9250000000002
279.465500000007 30.7605999999996 -2.83809999999962
289.963300000007 31.5787999999995 -11.9021999999996
296.119000000009 57.2947999999996 -14.6027999999996
305.858300000006 34.8680999999994 -15.8552999999995
325.739100000007 50.7922999999993 -13.8014999999994
334.916000000006 41.1583999999992 -13.7826999999994
343.672500000005 37.6872999999991 -15.4292999999993
348.319700000005 47.138899999999 -9.29499999999941
355.94781069089 36.8887334031352 -24.0431200838579
-1.52349999996667 0.942600000000118 -0.411400000000767
-1.17629999996633 7.78420000000033 -3.10940000000081
165.883800000019 23.6946000000006 25.6155999999997
244.845500000012 40.3380000000002 -4.18779999999995
214.268500000016 40.9529000000006 -11.9703000000001
138.917100000022 13.5966000000005 26.8612999999995
297.200900000007 24.3900999999994 35.5138000000004
323.178700000007 25.4410999999994 30.4291000000006
336.470800000007 53.264599999999 1.65020000000048
349.859300000005 28.393499999999 20.8198000000006
348.893800000005 40.603499999999 -7.18269999999935
-0.813699999966975 -1.14729999999987 0.109799999999204
0.633000000033916 -8.13629999999966 -1.76390000000062
165.858000000018 6.96700000000027 25.7543999999995
247.41780000001 -0.221199999999929 -2.68259999999989
219.119300000014 -6.66449999999962 -8.3083000000001
340.586700000005 0.115699999999267 3.24530000000081
352.724100000003 16.074299999999 -6.3178999999992
ID=MYRtriFEMVENAMNH77561*

LM3=54
5.12339999999309 -7.62840000000105 -9.83959999999787

14.7661999999987 -2.61610000000159 -7.78030000000126
170.854300000006 -3.80159999999938 3.6795999999999
203.143699999998 -2.50400000000016 -3.17309999999965
166.135400000007 13.6471999999999 5.03540000000001
230.224999999992 6.24509999999923 -1.85289999999938
244.552799999998 11.6544999999994 -5.56509999999945
256.369499999999 -2.21420000000041 19.5133000000005
272.191900000001 21.8923999999998 -15.7171999999996
264.562299999996 13.7680999999995 -1.85309999999951
274.1026 12.2884999999996 -8.17829999999956
281.822699999999 -8.14780000000043 -7.02109999999943
285.8687 11.2704999999997 -12.5605999999995
308.271099999998 0.10949999999503 -11.9401999999994
318.866599999998 9.91299999999953 -13.6188999999993
328.187499999998 16.4864999999994 -13.6455999999993
330.685899999997 26.8263999999994 -18.9533999999993
338.424899999997 27.9273999999994 -7.10939999999925
333.508199999997 8.07839999999945 -10.4656999999993
344.321999999996 17.6555999999993 -17.0905999999992
4.3105000000003 8.92429999999879 -9.26280000000014
13.6054000000006 5.97429999999863 -6.91510000000046
173.011000000006 31.6537 3.25779999999998
198.469600000004 35.6937999999999 -1.82069999999996
229.7908 32.8277999999995 -2.77379999999972
242.985499999998 29.1951999999995 -5.12599999999962
249.826199999999 43.7920999999995 19.3596000000003
263.120099999999 29.4974999999996 -2.18259999999959
268.870699999999 32.0445999999997 -8.23969999999959
276.320999999999 53.6667999999995 -6.39809999999957
282.644599999999 34.2224999999995 -13.1170999999995
304.529799999998 49.9554999999994 -11.8164999999994
316.209499999997 42.3604999999994 -12.8948999999994
325.876999999997 37.4339999999994 -14.7617999999993
329.241899999997 46.1034999999994 -9.68769999999934
341.859899999997 38.9385999999994 -16.2905999999993
-0.305700000003361 1.2212999999868 0.0832000000004625
0.988499999998772 6.64709999999864 -1.44949999999994
165.780900000006 20.5492999999999 27.2237
234.4181 38.3451999999996 -0.0984999999996773
205.120000000004 39.7190999999998 -8.60579999999989
135.140700000005 10.0177999999998 27.091
277.870899999998 24.0710999999996 33.1469000000005
305.862299999997 25.2824999999995 28.3211000000006
316.658599999997 50.9980999999995 0.66660000000066
331.655499999997 26.3518999999995 20.6957000000007
330.609199999997 41.6150999999995 -6.56339999999934
-0.304500000004109 -0.35880000000141 -0.00819999999961052
1.79719999999327 -5.47410000000146 -1.65369999999907
165.652300000006 5.11320000000008 25.98
236.821599999996 0.402999999999422 0.864700000000506
208.893900000006 -5.66749999999998 -9.7718999999999
319.329899999998 0.141699999999474 -0.75029999999926
332.864199999997 12.9949999999995 -5.20489999999924
ID=MYRtriUNKUNKMfNB102639

LM3=54
7.0301 -8.2372 -12.9768
12.5447 -4.8561 -11.3385
195.8766 -1.2024 6.5244
224.9166 -2.1997 -0.108
189.8289 14.658 5.7966
261.6639 7.7939 -3.0638
272.9792 12.2066 -6.0558
282.6539 -0.0129 19.9762
306.3486 23.8824 -20.19
294.1842 14.5677 -2.4771
300.0797 13.2918 -11.1635
316.4062 -7.4998 -8.14
317.8957 12.936 -14.9324
341.3829 -0.1775 -12.6888
349.4345 10.6085 -13.7756
358.8061 17.922 -14.1866
360.1307 29.2622 -20.8708
368.8223 30.1117 0.8646
362.3659 9.6163 -9.8028
367.2134 20.7017 -23.9009
4.6541 9.6001 -9.8433
10.6683 6.2777 -9.2452
191.5472 31.4552 8.6977
219.4792 37.162 1.5732
258.5621 33.7807 -2.3095
269.9685 31.9211 -5.2377
278.9446 43.9092 20.8156
291.241 32.7255 -2.5168
298.1621 35.5973 -10.4088
310.144 57.9868 -6.9148
315.3995 38.261 -14.2554
336.2138 54.7881 -11.712
346.5912 44.7198 -13.0396
356.1608 39.8097 -14.0451
359.7115 48.7588 -10.1283
365.7704 38.9028 -23.4575
-0.2098 0.3423 -0.5906
1.6329 6.8037 -1.1829
173.8551 18.9706 29.7803
261.2452 38.998 1.0523
232.9201 42.081 -3.4621
142.3183 10.1831 28.3527
316.6961 25.908 34.239
338.9225 27.129 31.3759
351.6643 53.6609 -0.6877
363.6186 28.2651 23.2719
362.8456 42.8665 -5.5495
-0.4448 -1.8662 -0.1734
2.1825 -7.685 -3.5507
175.9274 6.7593 29.2155
263.1736 2.2251 -0.2502
232.5329 -5.7334 -5.4002

355.4138 2.9969 -0.352
364.2597 15.0912 -5.4279
ID=MYRtriUNKCOLMfNB10622*

LM3=54
5.47910000002215 -8.29280000000297 -13.897899999998
9.75160000002773 -4.84000000000219 -11.9789000000008
210.877900000019 -2.19689999999939 1.42559999999924
235.750800000007 0.913899999999991 -2.37480000000001
193.962100000021 15.2732000000001 2.88119999999948
268.092799999998 8.29339999999918 -5.00939999999987
279.492900000003 12.1662999999995 -7.05369999999995
289.489900000004 -0.265600000000318 19.0148000000001
298.306300000005 24.6403 -17.4838000000001
300.795299999999 16.2497999999996 -4.27469999999992
307.538200000004 13.7149999999998 -11.9084
318.521500000002 -8.41470000000026 -7.95119999999984
323.888300000003 12.4493999999998 -14.6142999999999
347.4698 1.51959999999969 -11.9617999999997
356.8878 11.8772999999997 -13.1694999999998
368.5009 17.1960999999996 -12.8465999999997
369.7296 30.7898999999996 -17.8767999999998
383.1957 31.2442999999997 -1.80969999999973
371.622499999999 8.82119999999964 -8.14869999999967
378.411299999999 20.3247999999995 -23.2065999999997
4.24510000002966 9.49629999999828 -14.6053000000005
9.97340000002942 6.76269999999812 -12.6319000000006
208.73650000002 36.1180999999999 1.24009999999954
231.173000000016 37.5310999999999 -1.60700000000035
266.490700000007 36.0640999999996 -4.91370000000013
277.862000000004 33.8793999999996 -7.24860000000006
282.368500000005 47.0253999999996 18.3864999999999
298.981600000003 33.9339999999998 -4.41650000000002
302.420400000004 36.5878999999998 -14.0166
312.372500000004 60.7675999999998 -9.83440000000003
321.091200000003 40.2901999999998 -15.8437999999999
343.721300000002 56.4848999999997 -11.3982999999999
354.077200000001 47.1106999999997 -12.5312999999998
365.2681 42.3948999999997 -14.1656999999998
366.959600000002 52.2001999999997 -7.00159999999978
375.774800000001 42.3073999999996 -23.5992999999998
-0.749099999974352 0.239099999998112 -0.42379999999986
0.419700000028123 7.46929999999825 -3.99160000000027
177.229400000022 18.8945999999999 26.6854999999995
266.789800000007 42.2954999999997 -2.29180000000013
236.402900000014 41.3694999999998 -7.63650000000034
155.678700000024 11.2497999999997 27.5990999999996
311.398100000003 26.6798999999997 35.5958
340.969400000001 28.6134999999997 32.0495000000001
356.713300000002 57.7850999999997 -0.128699999999878
370.922000000001 30.6802999999997 24.7436000000002
368.854200000001 48.7540999999997 -5.00599999999978
-0.555999999975036 -0.744700000001846 -1.50129999999983
1.5145000000002147 -6.4469000000021 -4.65959999999938

176.870000000021 7.49549999999998 26.6812999999996
268.654800000001 1.73189999999946 -2.5321999999999
240.658400000016 -2.72979999999993 -7.87350000000047
360.7744 1.55009999999968 1.6027000000003
372.943299999999 13.7441999999996 -4.02039999999969
ID=MYRtriUNKBRAMfNB1921**

LM3=54
4.29699999999023 -7.75639999999982 -13.8448999999991
11.3559000000015 -4.79890000000017 -10.4993999999994
185.569100000003 -3.04110000000001 -0.257399999999737
216.303900000005 -2.76489999999982 -8.10930000000016
180.453700000007 16.0312000000002 0.912499999999807
249.174699999991 7.04049999999926 -8.08829999999923
257.4274 11.0146999999998 -10.5115999999997
270.4877 0.40849999999961 16.9816000000002
283.238899999998 23.7929999999995 -22.0422999999999
279.852799999997 15.5995999999995 -7.02279999999985
283.963599999997 12.4202999999996 -13.9638999999999
296.924300000001 -9.91590000000033 -9.5840999999999
297.7109 12.0335999999996 -19.8084999999999
327.255000000002 -0.123400000000416 -12.8204
332.310500000002 10.8550999999995 -16.0984999999999
342.447800000002 16.2850999999995 -17.1591999999999
343.086100000002 27.8674999999996 -22.9022
354.531500000003 28.8942999999996 -6.35049999999992
346.699500000003 8.7471999999995 -11.5079999999999
355.511000000003 19.9391999999996 -25.8964
2.45789999999755 6.55359999999937 -13.7941999999992
10.4610999999991 5.52519999999951 -10.1210999999992
182.611400000004 34.7949000000002 -2.12999999999994
213.6405 38.6774999999999 -9.58769999999986
249.205299999996 36.0915999999995 -8.05679999999969
257.438099999996 33.2795999999995 -10.0529999999997
268.897499999998 46.6642999999996 15.4891000000002
277.713799999997 32.9544999999996 -7.00379999999985
286.308099999998 35.9727999999996 -13.9716999999999
292.686299999998 58.4628999999996 -9.47529999999989
296.023599999999 37.6784999999996 -20.0613999999999
322.5921 53.3939999999996 -14.4743
331.116700000001 43.4519999999996 -16.5009
339.763400000002 37.9458999999996 -19.3821
344.345200000002 47.6133999999996 -13.3916
354.307200000002 37.4485999999995 -26.9850999999999
-0.711100000004696 0.81589999999938 -0.232699999998971
0.955699999996784 8.34569999999943 -1.48359999999912
167.493900000005 24.1364000000002 25.6449000000003
247.291099999997 43.7564999999996 -5.64879999999976
214.513299999999 41.6042999999998 -14.4976999999998
138.998700000002 13.6491999999999 26.6415000000008
291.0546 25.8852999999996 33.7282000000002
323.298600000001 27.7238999999996 30.3354000000001
336.993300000001 54.4555999999996 -0.77609999999993
346.943200000002 28.9671999999995 20.3814000000001

346.904400000002 44.4968999999995 -9.89549999999997
-0.244100000004919 -0.89540000000067 -0.867099999998894
2.50229999999346 -7.8992000000007 -1.97229999999884
170.798100000004 9.0442000000001 26.3948000000003
250.878599999996 0.113899999999404 -6.12019999999945
218.815000000001 -5.37220000000034 -12.7081
339.752700000002 2.43379999999951 0.441800000000069
349.127100000003 14.1388999999995 -8.3865999999999
ID=MYRtriUNKBRAMfNB1922**

LM3=54
6.08580000000569 -7.7040999999998 -14.889499999998
11.496800000007 -3.8682999999998 -12.3713999999988
195.462600000004 -2.76540000000004 0.133800000000821
230.423800000005 -4.41709999999996 -8.71439999999931
179.390800000004 15.9801999999999 2.82000000000083
260.474200000004 8.9533 -3.85559999999937
277.236400000004 13.9497000000001 -7.12939999999949
285.313500000004 -0.260099999999945 20.5966000000006
307.269200000002 26.4674000000002 -17.1311999999996
296.856500000003 16.0600000000001 -2.0783999999995
309.042300000002 16.1599000000001 -9.78659999999953
321.572500000002 -10.6175999999998 -11.5817999999993
324.184800000001 14.1818000000003 -14.2203999999995
349.543800000001 -0.458499999999575 -12.8713999999993
357.557000000001 11.1217000000004 -14.5992999999994
368.527600000001 18.4798000000005 -15.8348999999994
368.763700000002 29.7256000000006 -20.5426999999995
382.048800000002 32.0648000000005 -3.55339999999944
373.438900000001 8.65430000000054 -10.3956999999993
381.032900000001 21.3540000000006 -25.2942999999994
2.88760000000686 12.3061999999998 -12.8993999999984
9.82670000000704 9.87809999999979 -10.9143999999985
195.072800000005 37.8182 0.465500000000706
226.431500000005 44.3027000000001 -7.2259999999994
262.463600000004 37.8925 -4.0779999999995
273.333600000004 34.2320000000001 -7.68129999999957
283.139100000003 48.0608000000001 20.8019000000005
294.121900000002 34.2542 -2.83439999999958
308.475900000002 36.3316000000002 -9.58979999999958
317.470500000003 63.0760000000003 -9.43939999999964
323.217900000001 40.4476000000003 -14.1387999999996
345.952300000002 58.0801000000004 -13.4861999999996
356.854000000001 46.9733000000005 -13.4137999999996
367.727900000002 41.5613000000006 -16.3383999999996
371.236700000002 52.3840000000006 -12.1557999999996
380.029800000003 40.4946000000007 -25.6578999999996
-0.547799999993959 0.561399999999573 -0.548699999998347
-1.11209999999372 9.52039999999955 -2.84699999999835
174.297500000005 22.8051 29.9651000000008
257.489300000003 46.5925 -0.986599999999516
231.510500000005 45.8627000000002 -13.7116999999995
148.899300000005 13.3630999999998 29.054900000001
309.291000000002 25.5187000000001 35.1904000000005

341.357000000001 28.2673000000002 31.8775000000005
359.721000000002 58.3571000000005 1.03990000000036
373.550200000002 31.5728000000005 25.1394000000006
372.820100000002 47.4589000000006 -7.61089999999958
0.220000000006219 -1.06870000000024 -0.118799999998356
2.17430000000686 -8.68580000000024 -3.7563999999984
176.817200000005 7.23819999999995 29.6244000000008
263.607600000004 0.0974999999999815 -2.56999999999938
237.460500000005 -5.21649999999995 -15.3399999999993
363.295300000001 1.54450000000044 1.79250000000067
375.135300000001 15.0726000000004 -5.94599999999934
ID=MYRtriUNKUNKMfNB23678*

LM3=54
0.791299999976981 -7.99299999999694 -16.4477999999989
6.89839999997824 -3.9184000000002 -14.4563999999984
146.964999999988 -0.970500000001098 -0.0128999999989925
183.58109999999 -1.7770000000009 -5.44649999999906
154.738699999989 13.1194999999991 0.504200000001087
208.137499999992 8.2976999999994 -2.97579999999929
221.684799999993 10.8220999999994 -5.56489999999933
236.147799999992 -2.06530000000043 17.3713000000007
251.654399999994 22.1932999999995 -14.8465999999994
245.651999999994 11.9038999999996 -2.63409999999941
251.876399999994 11.6462999999996 -10.4499999999994
257.340299999993 -11.8540000000006 -10.4491999999993
265.243399999994 10.3300999999995 -15.0641999999993
289.269899999993 -0.764300000000559 -12.9936999999994
296.501399999992 10.4128999999994 -13.5584999999994
304.014499999992 17.0627999999995 -13.3889999999994
305.809699999993 27.4987999999995 -18.6315999999994
315.344599999991 28.5035999999995 -4.6554999999994
308.607099999993 8.5955999999996 -8.20559999999932
315.155399999992 18.4881999999995 -21.9649999999993
-0.56420000002283 11.7416999999983 -12.713699999998
5.58719999997753 9.26989999999789 -11.6813999999981
145.962799999991 27.1105999999993 1.55890000000102
181.526699999992 34.6897999999993 -3.49579999999913
207.956299999993 28.9576999999995 -2.11029999999928
219.711099999994 27.9926999999995 -4.87079999999937
230.347099999993 42.2412999999995 16.5262000000007
243.794399999992 30.9039999999993 -3.3169999999994
250.579299999993 33.0646999999994 -10.7601999999994
250.383299999994 57.3144999999997 -10.7949999999994
263.172099999995 37.0443999999997 -16.2024999999995
283.508599999993 52.1812999999994 -12.5883999999994
293.419899999995 42.2847999999998 -13.2347999999994
301.365899999994 37.2070999999996 -13.0374999999994
303.867099999993 46.7936999999993 -7.55549999999931
312.864999999993 37.2090999999995 -21.6112999999993
-3.3893000000239 7.43989999999782 -2.05619999999807
-2.77187571246484 9.21710989052495 -2.84851782775545
147.181199999986 17.4515999999986 22.7640000000012
207.027699999994 34.1710999999995 -0.093499999999346

185.446299999992 39.9175999999995 -11.2757999999992
118.681599999986 9.3896999999986 20.3140000000013
260.02899999999 23.1826999999994 31.6701000000006
285.119799999992 25.6823999999994 27.6797000000006
296.813699999992 52.2743999999995 -0.0936999999994073
304.089799999991 26.9714999999995 22.1333000000007
304.375499999992 41.1779999999995 -5.01789999999939
-2.00600000002349 -1.5708000000022 -1.09299999999803
-2.06640000002402 -6.97480000000228 -4.24739999999804
146.842399999989 5.96719999999901 22.0551000000012
209.375699999992 1.79649999999949 -0.304299999999266
189.381799999993 -5.64520000000057 -13.3979999999991
302.087599999992 0.323199999999563 0.528900000000698
307.792199999992 12.4672999999995 -5.7397999999993
ID=MYRtriUNKBRAMfNB29667*

LM3=54
4.24940000000469 -7.8050999999983 -12.9547000000006
9.86200000000169 -4.73989999999979 -10.6248000000009
182.448300000003 -4.23219999999985 5.41469999999962
217.291200000002 -4.14050000000002 0.457399999999601
186.338500000003 12.6115000000001 6.70309999999966
246.120000000002 5.78089999999993 0.186899999999602
259.695500000003 9.50549999999999 -4.5956000000003
270.528200000003 -1.54149999999996 21.5591999999996
288.947200000003 22.2756000000001 -14.5382000000003
280.908100000002 14.3378 -2.50640000000052
287.674800000002 11.9538 -9.28080000000044
300.975371176473 -7.81516082978669 -8.48050175059211
303.369400000002 13.3855999999999 -15.2208000000004
327.786500000001 -0.0434999999999715 -10.9329000000003
336.472900000001 10.6852 -12.5031000000003
344.5113 16.2544999999999 -14.1800000000002
345.980000000001 28.2722999999999 -19.7724000000002
355.3454 29.6718999999999 -2.19350000000033
348.0691 7.04959999999994 -7.39790000000018
355.127581136295 18.9851659685182 -21.8911237802689
2.23660000000246 9.53910000000062 -11.501
9.39130000000247 7.10830000000058 -8.63229999999991
178.199100000002 30.157 5.80029999999962
214.849900000002 35.969 -0.946000000000459
242.928000000002 31.7787999999999 -1.03620000000041
257.821300000002 30.4526999999999 -4.99040000000036
264.158400000003 44.6334 19.9636999999996
279.106000000002 31.1250999999999 -2.91220000000046
286.014800000002 33.2336 -9.70180000000037
291.253000000002 57.8736999999999 -7.63240000000039
302.072500000002 36.1957999999999 -14.7728000000004
322.780100000001 53.2272999999998 -11.9133000000003
333.284100000001 43.7082999999998 -12.8990000000003
342.454200000001 38.3288999999998 -14.8959000000002
344.748500000001 49.4549999999999 -8.47560000000021
352.582182164993 38.9864496331877 -22.6315861477287
-0.503999999996807 0.628500000000732 -0.509399999999834

1.91800000000242 6.54130000000078 -0.629200000000027
171.630700000004 20.6312000000002 27.0567999999998
246.556800000002 38.8058999999999 -1.02870000000048
219.143900000001 41.3599999999999 -8.99530000000046
151.191100000003 11.4962000000002 28.3850999999997
290.490100000001 24.5734999999999 36.8375999999996
321.957100000001 26.5717999999999 33.0298999999997
336.3421 54.1788999999998 -0.285500000000413
351.5486 28.9095999999999 20.2030999999998
346.735500000001 42.9927999999998 -5.02570000000029
-0.596599999997382 -0.697499999999471 -0.323199999999777
2.78950000000261 -6.86259999999935 -1.94989999999984
175.500400000003 3.03810000000012 27.8403999999996
249.550000000002 -1.13529999999998 0.587899999999554
221.779700000002 -8.54889999999998 -8.42090000000057
342.071800000001 1.45439999999999 -0.183100000000273
350.6477 14.4722999999999 -4.57770000000028
ID=MYRtriUNKBRAMfNB47918*

LM3=54
4.4727 -8.7078 -11.8912
9.911 -4.7787 -9.9887
175.5858 1.0156 1.0694
204.9904 1.669 -5.0723
158.2535 12.7407 1.3867
226.621 9.1145 -6.2813
242.6044 13.0574 -10.4687
248.9552 0.1848 13.992
272.3607 22.9469 -20.5521
262.0179 15.7264 -5.7876
274.4165 14.1477 -12.9707
278.7727 -5.8183 -11.7075
284.0733 12.7939 -15.794
309.2049 1.7548 -11.8641
316.5684 10.0826 -12.5562
324.3602 16.3871 -13.4684
323.7128 27.4191 -17.0856
334.6312 28.7599 -6.0742
328.0758 8.8996 -8.1187
336.5037 21.0129 -20.4063
2.9142 7.836 -12.3923
9.1369 5.5177 -10.173
174.0294 29.1048 0.846
202.4559 33.4755 -4.7862
226.6957 28.7572 -6.7033
243.2691 28.1044 -9.9382
245.8604 41.9817 12.8329
260.8028 30.1431 -4.9078
271.6764 32.3574 -12.3916
274.1529 53.0727 -11.0543
283.4503 35.4449 -14.552
305.0954 49.8585 -11.0592
314.3747 43.2579 -11.6708
322.749 37.688 -13.1802

325.2903 46.5582 -7.8854
334.9425 37.0427 -19.8223
-1.5412 1.0237 -0.2269
0.615 7.1917 -2.0722
155.0879 20.0712 20.8942
233.1371 38.5242 -4.1614
209.0607 38.7316 -11.4111
144.0378 12.3318 25.5442
276.2767 22.7479 27.0229
298.796 24.4975 25.5923
315.2575 51.6703 1.7736
324.5562 27.5979 21.05
326.7285 41.585 -6.4806
-1.2698 -0.6506 -0.4545
1.8347 -6.2869 -1.6719
157.1402 6.4227 21.8552
235.5025 2.3283 -4.4066
211.6375 -1.6543 -11.3988
318.5648 2.5298 2.5015
329.7031 14.274 -6.4203
ID=MYRtriMALUNKMfNB77272*

LM3=54
4.480599999987 -9.13389999999842 -12.9179999999991
11.4227999999904 -4.79129999999871 -10.8623000000002
165.241700000003 -3.10539999999815 1.3302999999994
194.624899999995 -2.68579999999903 -5.37369999999989
156.742400000003 12.7772000000014 0.766399999999781
222.646399999991 6.70370000000005 -5.12109999999968
239.489399999997 11.7614000000002 -9.83489999999984
248.439199999997 -2.40829999999952 19.3528000000002
272.7714 22.5677000000005 -22.2715999999999
258.886099999995 13.3751000000002 -4.20369999999985
269.3621 12.7989000000004 -13.3432999999999
277.126599999998 -11.1057999999996 -13.8348999999997
285.461699999999 10.8391000000004 -17.5396999999998
307.764299999997 0.565600000000367 -13.2316999999996
316.632599999996 10.2728000000003 -14.5378999999996
325.187799999996 17.4914000000003 -14.7644999999996
327.378199999996 29.5362000000002 -20.8361999999996
336.020699999996 30.8831000000004 -4.77019999999958
328.517499999996 9.95940000000033 -8.89319999999958
340.830699999995 21.6693000000003 -21.0708999999995
1.78539999999172 9.39280000000136 -12.2951
9.50429999999235 7.38520000000119 -10.4558000000001
163.941200000003 29.6294000000012 0.811699999999778
192.626400000002 34.6357000000009 -5.44150000000015
220.272099999998 29.4877000000004 -6.17689999999996
237.471299999998 27.5366000000003 -10.4060999999999
243.533699999999 43.3624000000004 18.5453000000001
255.744499999998 30.2189000000003 -4.31969999999981
267.601099999999 32.1960000000004 -13.9390999999999
269.990799999999 56.8728000000004 -14.6708999999998
282.164699999999 36.4647000000003 -17.2592999999998

300.640199999998 52.7015000000003 -14.5210999999998
311.105099999997 45.6774000000003 -15.3615999999997
321.747499999996 41.1935000000002 -16.5403999999996
321.883499999997 51.0557000000003 -8.25689999999963
335.674399999996 41.4853000000003 -22.0967999999996
-0.82070000001215 1.43260000000124 -0.559799999999478
0.0400999999901939 7.7722000000013 -0.703499999999814
146.408100000002 20.6554000000014 25.2349999999998
221.675099999999 38.1999000000004 -4.23469999999999
197.140600000002 39.0665000000007 -14.7817000000001
124.4056 9.3882000000015 26.6563999999999
276.017899999997 23.6346000000004 33.9590000000003
297.552299999997 26.0236000000004 30.1183000000003
311.366799999997 54.8678000000004 -0.0416999999996843
322.627399999996 30.1842000000004 22.1293000000005
325.038899999997 44.2783000000003 -5.58019999999963
-0.30020000001278 -0.989299999998847 -0.492799999999351
2.88599999998439 -8.01699999999902 -2.51719999999892
149.156500000001 1.92100000000149 25.0739999999998
224.740299999994 -2.5739999999997 -1.97209999999973
200.335100000004 -6.31529999999893 -13.4049000000003
318.994399999996 1.60460000000038 0.839600000000417
328.621999999996 13.3851000000004 -5.18709999999957
ID=MYRtriUNKUNKMfNB******

LM3=54
8.29789999998158 -7.76370000000204 -12.0728999999999
15.6608999999662 -2.60270000000426 -9.59859999999952
168.541000000004 2.03610000000029 7.40629999999973
207.74829999999 3.66929999999903 3.08419999999986
168.169499999994 17.1635999999991 8.48480000000013
233.497910748619 13.1505962359658 3.69581232146281
245.675199999998 17.0245999999998 0.907500000000053
256.262899999995 4.39349999999971 24.8705
279.602299999994 31.4201000000001 -8.52649999999991
268.564999999996 21.7868000000001 5.32930000000006
278.860099999991 21.5712999999997 -1.00379999999994
285.321799999995 -0.0514000000002 0.403200000000089
289.836799999995 21.2893999999999 -4.90209999999993
313.591299999994 9.87629999999994 -3.0599999999998
320.392799999995 21.3388000000001 -2.43279999999975
328.694399999995 25.6511000000001 -0.597199999999718
330.177199999992 34.4592999999999 -6.27569999999973
339.725299999993 34.5825 6.62790000000028
332.614699999994 16.7958 3.14570000000028
342.437399999993 27.0505 -7.3267999999997
5.94589999997368 9.60019999999645 -11.1585999999996
14.6394999999713 6.86539999999606 -8.78139999999962
167.433399999994 33.1488999999991 9.64709999999995
204.818399999993 41.2724999999992 4.42539999999989
233.881999999994 35.6944999999995 3.39439999999995
243.330999999996 35.8015999999998 1.48430000000004
252.953299999994 50.5044999999998 25.6324
266.775399999995 37.7863000000001 6.12190000000006

278.759499999994 41.3485000000001 -0.373599999999915
281.907299999994 62.8258000000001 0.403200000000111
290.891599999994 45.0480000000001 -3.73899999999989
311.079899999993 58.8861000000001 -2.54089999999982
319.872699999994 48.3732000000001 -1.56529999999976
328.755799999993 43.9815 -0.934499999999753
332.113199999992 53.3497 2.97430000000024
340.873399999992 43.8993999999999 -8.60349999999971
-0.415800000025133 0.334499999996576 -0.684999999999627
2.0696999999738 7.46449999999642 -2.0067999999996
157.277999999994 20.8533999999989 31.5873999999999
235.463599999995 43.7446999999996 6.18119999999999
202.074599999994 45.7238999999993 -0.241900000000084
132.331799999994 12.7765999999985 29.6074999999999
277.695999999994 29.6539999999999 40.6744000000001
308.200399999993 33.5187 36.8933000000001
320.859599999993 57.7322000000001 10.7468000000002
335.047699999992 34.5157 30.1077000000002
333.038999999993 48.6759 5.57070000000027
-0.382900000024759 -0.73590000000339 -1.14589999999961
3.50479999997622 -6.40270000000324 -3.60949999999958
158.894499999996 10.5318999999991 30.3581999999998
238.162399999996 6.08119999999949 5.29880000000001
208.086999999995 -1.6214000000008 -2.38430000000009
320.211299999994 10.4052 11.8381000000002
334.426499999994 21.9694 7.28200000000029
ID=MYRtriFEMUNKUSNM258580

LM3=54
4.8634999999699 -8.63410000000072 -11.5409999999991
13.344299999977 -3.65960000000194 -8.18100000000144
175.879899999997 3.3862000000009 7.27679999999952
198.567399999991 4.38939999999989 1.75129999999991
161.171399999997 18.1428000000003 12.1011999999998
228.777099999987 15.4323999999992 4.25829999999995
241.083099999993 19.6106999999996 0.727200000000139
254.932299999994 7.14819999999978 29.4303000000002
269.465199999997 31.6088 -7.09779999999971
262.957099999992 22.0368999999996 8.01480000000013
270.716199999996 21.6721 2.12240000000027
274.404699999994 -0.058900000000194 0.454900000000319
283.288999999996 21.6481999999999 -3.0404999999997
305.382299999994 10.5984999999997 1.00070000000029
312.530599999994 20.8112999999998 3.10450000000034
321.474899999995 25.0894999999998 3.01290000000034
322.971699999994 36.6877999999997 -0.779999999999688
332.314599999994 37.9737999999997 12.1429000000004
327.047999999994 17.0879999999997 7.36110000000034
335.220499999993 28.4481999999996 -3.29169999999967
2.99359999997738 8.70679999999853 -11.8227000000009
12.7573999999783 5.5752999999984 -8.00900000000096
171.304399999997 36.5488000000002 6.94879999999976
192.111199999998 40.4247000000002 1.47679999999991
225.268999999995 35.9723999999998 3.42700000000002

238.624699999994 36.1916999999997 -0.0524999999999016
251.358599999995 51.5907999999998 30.1373000000001
261.562899999995 37.7979999999998 7.85080000000016
269.990399999996 40.2390999999999 1.93840000000023
267.318599999995 62.3427999999998 0.584300000000185
281.423399999996 42.8623999999998 -1.96209999999976
299.553899999994 60.0758999999996 2.25870000000027
308.733199999994 52.7779999999997 3.36860000000028
318.864699999994 49.4049999999996 2.6834000000003
321.800999999994 57.6926999999997 7.40090000000027
332.864399999993 47.9659999999997 -3.57599999999971
-0.650900000026762 0.809999999998265 -0.116400000000494
0.953999999975602 7.2013999999842 -2.14380000000081
159.208299999995 24.9796000000002 34.1600999999998
227.414799999995 43.8741999999998 4.23100000000008
198.252699999997 44.4945000000001 -8.27680000000005
140.668599999992 17.28 35.3733999999996
271.213899999994 31.9382999999998 44.3696000000002
297.555899999994 34.5024999999997 41.4533000000002
309.949199999994 60.0823999999997 13.6381000000002
327.912499999994 37.2892999999997 33.1833000000003
322.511699999994 52.1903999999997 12.0223000000003
-0.336500000027361 -0.369900000001787 -0.307100000000501
2.52549999996994 -6.43540000000203 -1.8576000000002
161.660999999995 12.9669000000003 34.1720999999997
230.982899999991 9.50499999999949 4.84440000000007
203.504899999999 0.394700000000343 -6.85280000000008
314.888499999994 11.6975999999998 13.8321000000003
326.516099999994 23.0580999999997 11.4526000000003
ID=MYRtriFEMUNKUSNM269398

LM3=54
6.3371999999695 -9.06270000000348 -10.5083999999981
14.396199999969 -3.70090000000244 -7.43889999999813
175.411999999984 5.70479999999908 15.7307
203.718099999987 6.50139999999947 11.3645999999999
158.726699999983 19.213999999999 13.1528000000001
227.12749999999 17.5096999999999 9.61179999999982
247.129699999992 20.2321000000001 5.61969999999978
258.379399999992 8.92340000000015 31.1955999999997
279.422099999996 33.0465000000007 -5.90710000000025
271.127999999994 23.1137000000004 11.3928999999997
276.825399999996 22.0436000000006 3.78289999999972
283.852799999995 0.173800000000466 0.0718999999997161
290.140999999998 21.5813000000007 -1.86350000000034
314.287799999999 11.2211000000009 -0.390100000000327
321.103599999999 23.717500000001 -1.80340000000029
330.275499999999 27.844300000001 -4.02060000000025
329.444299999998 37.453100000001 -8.1878000000002
340.974199999997 38.3847000000009 6.85729999999976
334.760899999998 19.4003000000008 2.06129999999975
338.612999999998 31.6371000000009 -11.4109000000003
4.88959999996731 8.75159999999654 -11.6014999999986
13.3520999999692 5.88089999999694 -8.23279999999854

172.988699999984 36.7813999999992 15.2796
198.010199999986 41.7087999999994 11.2626999999999
226.73759999999 36.8076999999999 9.67359999999978
244.700099999992 37.1630000000001 6.14729999999979
254.898799999992 50.7523000000002 32.0516999999999
269.188799999994 39.3078000000004 10.9974999999998
279.037099999995 40.9166000000006 3.14349999999981
278.119299999994 64.8157000000005 -0.0761000000001411
288.504899999996 44.3610000000007 -0.212800000000245
309.955699999997 60.0094000000009 -0.0224000000002036
318.123299999998 51.1031000000009 -0.246400000000253
328.143899999998 46.7459000000009 -3.54850000000021
330.530699999997 55.772900000001 2.11779999999983
336.751499999997 45.2072000000008 -11.6029000000001
-0.0697000000325232 0.354399999996653 0.164900000001391
1.6698999999678 6.89819999999672 -1.43049999999852
154.691799999982 24.7759999999989 37.5881000000002
234.910247777933 44.9051185908334 10.779307403243
208.966599999987 48.5073999999996 0.780299999999928
146.654599999979 17.8612999999983 37.9131000000003
282.643099999993 32.2928000000004 44.3221999999998
310.348199999996 34.5355000000008 38.9354999999998
322.063899999996 60.8094000000008 13.5015999999998
337.759899999996 38.1076000000008 28.7249999999998
332.349399999997 52.1907000000009 6.74449999999976
0.236599999967533 -0.75600000000343 -0.128399999998627
2.75869999996782 -6.94120000000343 -0.980599999998604
156.197199999983 13.3922999999988 37.7604000000001
236.928296006832 10.2560951396693 9.94930721379146
214.944599999986 1.60829999999948 2.22769999999984
325.355699999998 12.8820000000008 12.0807999999997
335.154299999998 26.1211000000009 4.87439999999975
ID=MYRtriMALBGUUSNM339666

LM3=54
5.40750000003128 -9.24629999999702 -13.1628000000002
11.8996000000297 -4.93869999999766 -11.6414999999999
181.946300000014 3.66950000000136 6.27270000000033
221.103600000012 4.13780000000107 1.20930000000033
186.984600000015 20.4593000000016 4.16690000000026
248.195300000009 14.6031000000009 1.99540000000025
260.561100000007 19.5642000000006 -0.197699999999805
268.106900000007 7.3400000000006 27.1206000000003
294.901900000005 32.7235000000004 -9.87349999999984
280.100800000005 22.9965000000005 4.99140000000026
294.056800000005 23.3830000000004 -1.09779999999975
299.714300000004 -0.198999999999709 -0.0292999999997194
304.922900000004 20.7261000000003 -4.87839999999975
330.114300000004 10.1973000000001 -0.118499999999777
338.008500000002 21.4838999999999 2.47710000000015
348.225700000002 27.2588999999999 0.834800000000167
350.049200000003 37.5698999999999 -4.12179999999988
354.803000000004 38.1173999999999 13.8634000000001
351.498700000003 17.0978 7.49880000000015

362.328800000004 29.2982 -4.15149999999989
4.81350000003125 9.9364000000029 -13.2937000000001
10.8261000000308 6.96630000000288 -11.6356000000002
180.549100000015 37.2871000000017 5.5377000000002
217.309600000013 43.8385000000015 0.594300000000159
244.701300000009 38.609800000001 1.14930000000018
258.425500000008 37.5550000000009 -0.489699999999817
258.955500000007 50.6468000000008 25.4204000000001
278.234500000005 38.7054000000006 4.40210000000013
289.785700000006 41.4637000000006 -0.914899999999863
293.560700000008 64.4721000000007 0.137300000000059
302.051100000005 45.7675000000004 -4.10029999999991
323.453500000006 62.0934000000003 1.50440000000008
335.559032813255 51.8556305045427 2.20304939163011
346.043900000004 48.4077 0.765800000000072
347.644500000004 58.0273 6.13770000000007
359.789200000005 48.8839 -4.54539999999991
-2.01579999996819 -0.0338999999970707 -1.36390000000004
-0.569499999968754 6.99560000000299 -3.82900000000003
160.491800000017 24.1751000000019 28.2892000000002
245.243000000009 44.756900000001 3.34330000000016
212.471100000013 47.3288000000014 -6.23709999999988
145.732900000019 17.246300000002 27.3735000000003
280.465100000006 31.4687000000005 42.7134000000001
313.453600000005 34.2499000000003 43.5924000000001
336.335800000005 63.0220000000002 14.3758000000001
349.030100000005 39.3442000000001 36.6374000000001
346.731200000004 52.9494 9.70800000000007
-2.22929999996869 -1.76589999999716 -1.06430000000005
0.865100000031529 -8.09279999999711 -3.23570000000006
161.964900000017 13.2899000000018 29.0396000000003
249.348100000009 9.01860000000074 4.59390000000023
221.137400000011 0.0584000000010015 -5.90049999999976
338.605500000003 11.1776 15.4463000000002
350.729500000003 24.473 11.3245000000001
ID=MYRtriFEMBGUUSNM361028

LM3=54
6.4401 -5.9625 -8.5546
11.6627 -2.4916 -5.9906
162.221 4.4286 7.5198
186.1368 4.9219 4.1033
150.6366 18.8486 5.1517
216.0709 14.5883 0.3074
232.6344 18.0903 -2.7751
247.7527 7.0894 24.6686
258.782 29.2197 -10.2566
251.2666 20.7052 3.3229
263.3544 20.2741 -3.7308
266.7598 0.5462 -0.1864
270.7998 18.3862 -7.1934
295.3354 7.0397 -5.5234
303.6876 18.2128 -5.6372
313.9319 24.0422 -6.6911

316.9425 35.0383 -11.686
320.8457 34.5558 10.0743
314.6416 15.6123 -1.7697
326.7821 26.3964 -13.3912
5.7684 6.6517 -10.1944
10.3244 4.8469 -7.6729
161.2263 36.4224 6.2209
180.5041 40.2997 3.997
214.9287 36.3872 0.5264
231.808 35.9303 -2.9098
241.1587 48.4961 23.742
249.7575 36.9037 2.8333
260.9839 40.1 -3.7221
261.6744 60.7021 -0.033
268.0439 42.7473 -7.1393
290.9413 58.891 -3.9778
300.5518 49.7573 -5.3632
311.5286 45.4735 -6.2113
311.9774 54.1406 -0.133
323.2869 46.3999 -13.851
-0.1184 -0.1275 -0.3483
1.2326 6.1581 -2.1414
144.7753 26.2038 26.4815
223.0133 42.7904 3.8332
187.6849 46.2367 -1.5485
118.5324 16.2358 26.2336
255.9171 28.4097 38.2755
286.7295 30.6593 35.3404
303.2455 59.5025 9.1028
312.7762 33.3483 28.0246
313.142 49.5179 1.7966
0.3443 -1.1059 0.0389
3.1723 -6.892 0.4317
146.9607 12.15 27.5751
222.8937 7.6819 3.4279
193.6631 1.1544 -0.0543
307.2255 6.5868 8.7265
317.7186 21.6224 1.2322
ID=MYRtriFEMVENUSNM372828

LM3=54
2.35239999998017 -6.32490000000071 -16.2116000000014
10.271899999982 -2.84010000000079 -12.538900000001
191.51749999999 1.70340000000015 1.99449999999963
214.455899999992 3.89600000000026 -1.69150000000022
172.124099999989 17.9478000000001 7.29139999999949
246.634599999993 14.1642000000003 0.327699999999936
257.937699999993 18.0884000000004 -1.20089999999995
265.350999999994 4.65330000000035 22.4241000000001
297.830099999995 33.2925000000005 -12.6701999999998
279.887399999995 22.4239000000004 4.3576000000002
285.832199999995 20.8018000000005 -3.49649999999978
296.917362495274 -0.521753485802417 -3.1687097924371
301.566199999997 19.9892000000006 -7.70369999999968

326.224599999998 9.93040000000074 0.283000000000503
332.492899999999 19.7245000000008 -1.00269999999949
341.020399999998 27.8002000000008 -2.81979999999946
344.114599999998 37.1351000000008 -6.66189999999948
351.549399999999 37.479700000001 7.3064000000007
345.219999999999 18.7417000000009 4.51000000000052
353.029689860431 29.2417594827952 -9.28783079536693
1.40579999998255 10.2792999999999 -13.3478000000012
8.8893999999821 7.42179999999985 -10.8853000000011
185.18039999999 37.2316000000002 8.14029999999958
210.033399999991 41.8142000000003 3.83129999999973
242.797499999991 38.5687000000002 4.12349999999987
254.778899999993 37.8154000000004 0.829300000000081
262.532699999993 50.0835000000004 27.3528000000001
277.979099999994 37.9426000000004 5.46230000000019
286.451499999994 42.3130000000005 -1.32609999999976
287.226699999996 66.1209000000007 0.291500000000384
300.482999999995 46.5873000000005 -5.3766999999997
322.528599999996 60.6093000000007 2.14510000000042
329.683599999997 52.1648000000008 1.03040000000054
340.138999999998 46.2942000000009 -1.15759999999945
340.765199999998 54.6112000000009 4.49940000000062
350.266199999998 45.492400000001 -8.19319999999954
-1.79770000001863 0.359399999999764 -1.07620000000126
0.862699999981703 7.00249999999984 -1.2969000000012
167.919999999988 21.6830000000002 29.8208999999995
245.507576326367 45.3587996519362 6.08268854616139
212.565399999991 46.7587000000003 -1.4990000000002
136.078799999987 11.7779000000001 27.2246999999993
291.663299999995 29.1683000000004 40.5852000000002
313.852799999998 32.4426000000008 38.5559000000006
331.655399999996 61.9845000000008 15.9978000000005
340.676899999999 36.3237000000009 31.8991000000005
340.838499999998 48.8838000000009 6.78820000000065
-1.21990000001848 -1.33880000000019 -1.01520000000133
2.85469999998223 -6.49920000000009 -3.62760000000116
169.353299999989 8.17570000000013 27.0467999999995
249.477728600956 7.89319701939421 1.14019494483902
218.135999999992 1.07580000000026 -7.74350000000021
334.774199999999 9.97200000000084 14.4408000000005
342.780899999999 26.4173000000009 5.65290000000061
ID=MYRtriFEMVENUSNM388302

LM3=54
3.4296 -5.6292 -12.1293
10.545 -2.6304 -9.3429
185.8928 1.9901 3.8224
205.5857 4.0598 0.2127
169.5177 17.734 5.8274
233.0497 12.8943 1.0214
246.9959 17.3457 -1.2323
255.0798 4.8371 23.3474
279.1871 28.1256 -10.8093
265.6941 19.4088 5.7052

272.2152 17.6247 -1.5515
286.4934 0.5112 -0.762
287.6157 17.9496 -5.6224
311.1387 8.1284 -1.972
319.1162 17.3469 -3.0594
328.4091 23.6384 -4.9197
330.2093 34.0412 -9.8188
338.6216 35.3395 7.6121
329.5548 13.5127 1.1308
343.3403 25.8554 -11.1289
2.2384 7.1937 -9.2874
9.582 6.2287 -9.1536
181.5496 37.4192 4.3771
203.2657 39.3224 0.7212
228.1582 35.0314 1.9548
243.8855 33.4633 -0.6159
250.8817 46.9533 23.5741
263.3753 34.1791 5.6543
269.3243 36.8754 -1.291
278.9083 58.0538 -0.2375
284.768 40.6913 -5.3524
305.4906 55.6742 -1.992
314.6236 48.3448 -1.8749
325.3596 44.6281 -4.9358
323.5737 54.4779 1.4598
338.3968 44.3702 -13.299
-0.5155 0.2843 0.0921
-0.9581 6.3736 -2.3943
156.2668 22.3439 29.7788
231.4817 40.259 1.9833
204.6407 43.6357 -4.9751
129.9182 12.7124 27.788
267.0787 26.8467 41.7804
303.0422 31.0134 38.9345
317.6103 57.8258 9.8182
333.6359 35.6472 28.5226
326.1493 49.2637 3.2869
-0.6427 -0.8382 -0.0895
0.6771 -6.777 -3.3566
156.856 9.6118 29.6874
234.9177 6.7014 2.0524
208.552 -0.7519 -5.1781
322.8561 8.2658 10.1607
330.8979 18.0781 3.9489
ID=MYRtriFEMUNKUSNM406490

LM3=54
4.6314 -7.8721 -13.2796
10.4919 -3.6344 -10.0293
180.0767 -2.9361 6.1515
199.6771 -2.731 1.9574
173.0123 14.7656 5.4433
230.052 7.5683 1.1238
249.5141 11.37 -3.6836

258.2511 -0.2952 21.8787
275.4495 22.691 -14.6045
268.5368 14.6427 -0.9794
275.6552 12.5125 -8.1248
285.5214 -7.6843 -7.0454
289.0317 11.609 -13.0103
312.2216 0.1117 -10.4063
321.6347 10.715 -11.0802
333.2753 17.6843 -14.6985
333.5672 27.5218 -18.8016
344.4644 27.5207 -2.8148
335.4361 9.0707 -8.0925
342.7701 18.6278 -22.0286
3.097 8.3556 -12.8381
8.5818 6.135 -9.998
176.8506 32.6244 6.782
197.154 35.8128 2.011
230.6958 32.0892 0.4403
247.8764 31.2208 -3.7959
257.7147 45.0047 21.1875
268.1625 29.7899 -0.6585
273.0331 32.8877 -6.9938
280.2218 55.0019 -5.274
272.5835 33.3447 -7.3149
310.4823 51.6191 -9.468
319.6715 42.2627 -11.3741
331.0763 37.8311 -13.8541
333.6111 46.1382 -7.3842
342.6078 38.2865 -21.3956
-0.7046 0.7664 -0.3836
-0.4122 7.8063 -2.5234
162.8454 20.4011 32.1703
238.901 38.8772 2.2881
212.2086 40.4623 -5.3999
137.3023 12.1447 30.4783
288.8324 23.2162 34.0513
311.585 25.8706 29.6032
320.2138 52.3874 4.9193
334.7027 27.5214 24.5363
337.6586 40.7527 -5.3863
-0.887 -1.278 -1.0424
0.7983 -7.5426 -1.7419
163.2084 9.4034 31.9219
241.0747 1.5875 3.2835
215.2959 -4.5177 -5.3814
324.3434 -0.3035 3.2435
340.1824 15.3169 -5.2986
ID=MYRtriFEMZOOMVZB185238

LM3=54
3.58429999996593 -9.17860000000311 -14.1267999999979
8.92869999998484 -5.35700000000189 -13.2989999999992
175.811999999991 -3.91270000000111 -4.92279999999869
204.661199999998 -6.09190000000008 -10.6109999999996

152.551900000003 11.6126000000002 0.543400000000393
237.462100000002 5.19219999999992 -11.4980000000001
251.7627 9.29179999999984 -14.7025999999998
263.923199999997 -0.67410000000049 14.0521000000003
286.831999999998 21.1117999999998 -22.3628999999998
274.002699999999 12.2753999999998 -7.20159999999977
284.764299999997 11.1049999999996 -14.5809999999996
288.706399999995 -13.0836000000008 -11.4513999999996
296.376599999996 9.15749999999965 -16.4891999999998
318.510699999994 -1.3433000000008 -11.4566999999995
325.083299999995 11.0578999999993 -12.5048999999996
334.742099999993 15.3847999999992 -13.6913999999995
335.548699999994 26.2507999999993 -18.2373999999996
343.814399999993 26.4686999999993 -1.04019999999949
337.576999999992 7.83399999999909 -8.95419999999947
344.568099999993 15.9186999999991 -20.3398999999994
1.3198999999837 8.84989999999822 -14.8419999999986
8.02839999998713 6.31679999999854 -13.1533999999989
176.142100000003 31.3464000000003 -4.65019999999966
199.675900000003 35.8604000000002 -9.20659999999982
237.174500000002 29.2268000000001 -10.3629
249.757900000002 27.7124000000001 -14.0149
258.429900000001 41.9087000000001 13.5517000000001
273.196 29.0268999999999 -7.65999999999984
283.6805 31.7788 -15.4917999999998
283.125699999999 56.4511000000001 -12.2204999999998
293.426911855143 34.6502029091572 -17.5246342923812
314.627299999997 49.9099999999998 -12.7685999999997
321.738099999995 40.4015999999996 -13.2327999999996
333.152299999994 37.9527999999995 -12.4886999999996
335.599999999994 44.9162999999995 -8.18239999999959
343.542399999993 37.3903999999993 -21.1336999999995
-0.897400000024493 0.42079999999734 0.0925000000017002
-2.32820000001915 7.17009999999788 -2.67359999999857
164.483700000001 18.2529000000002 20.9094000000006
240.350600000002 38.7438000000002 -7.82309999999996
215.146100000003 39.6504000000002 -16.9749999999999
145.699700000001 11.2788000000002 20.3599000000007
286.171099999997 22.9327999999996 29.8291000000003
312.019699999996 25.9916999999995 27.8155000000004
323.384299999996 50.6088999999997 2.02520000000038
337.293199999993 26.5415999999993 20.8951000000005
337.069899999994 42.0023999999994 -4.97689999999956
-0.809200000025879 -0.783700000002759 -0.283799999998299
-0.612000000031403 -7.92550000000324 -3.53909999999797
166.364499999999 6.79739999999986 20.4746000000008
242.176 -1.17720000000038 -8.8947999999999
217.900399999998 -7.1104000000008 -16.9068999999996
326.208699999993 0.855099999999116 3.4540000000005
337.866199999993 11.5635999999992 -3.9845999999995
ID=MYRtriUNKBGUBMNH111011

LM3=54
-0.1834 -10.315 -12.3006

8.1788 -8.1361 -9.8192
175.2944 -3.0513 1.8169
201.9185 -3.9363 -2.856
170.4587 14.7923 2.0846
233.0502 7.0005 -4.8711
246.3737 10.9518 -8.2772
257.4711 -1.3311 20.3723
276.7884 23.8771 -17.7864
271.0297 14.6544 -2.2735
274.9517 12.2166 -9.1912
290.6557 -7.4061 -7.9165
291.9078 12.6348 -13.8562
316.9721 1.0384 -11.1505
322.4496 14.772 -10.1267
332.9042 19.6169 -12.1778
333.5303 30.4328 -16.5465
341.0373 30.5227 0.1323
333.3935 10.3555 -5.352
344.835 19.3228 -17.0657
0.0148 8.7182 -11.968
6.632 6.059 -10.7584
170.0601 32.7839 1.3692
198.2392 37.3888 -3.8178
230.1489 32.1817 -5.4097
243.4638 29.8054 -8.4481
251.3865 44.3027 19.6833
269.5916 30.3273 -3.0641
272.3041 34.386 -10.1739
283.5129 56.3983 -8.7098
290.0017 37.8995 -13.1758
311.871 54.3304 -11.8019
319.6814 43.6303 -10.0258
330.3418 39.8635 -11.9372
329.8771 49.5698 -6.2765
343.7209 41.7642 -17.5916
-1.7648 0.2416 -0.8352
-3.3128 5.508 -4.6519
159.9164 21.6661 24.1349
236.4261 39.9716 -2.8508
214.2482 42.149 -8.6293
127.2292 11.1165 23.3897
278.4736 22.1561 32.9064
304.4877 26.0973 31.2092
320.7449 55.1549 3.6637
328.8478 29.0035 27.1124
332.0332 43.4388 -0.9498
-0.9195 -3.4315 -0.4143
-0.452 -6.7216 -1.702
161.7433 5.7485 24.8866
239.3733 -0.3732 -0.5772
216.4407 -4.6552 -7.567
325.9603 2.2546 2.321
334.5294 16.267 0.2057
ID=MYRtriFEMECUBMNH442588

LM3=54
1.20939999999929 -4.28509999999997 -11.9172
8.98546352848812 -1.45321101378909 -9.09411956528515
179.955199999992 -1.20600000000078 -5.91990000000011
201.782200000001 -0.183899999999884 -8.37239999999977
180.32359999999 15.739699999999 -1.81869999999927
230.131300000004 10.6850000000003 -5.87800000000025
240.947800000003 13.4146000000001 -9.04310000000002
252.216300000004 0.926400000000258 17.4334999999998
266.484100000004 23.7194000000003 -17.4304000000002
262.032100000004 16.3081000000002 -4.30120000000016
268.655000000004 13.7967000000003 -11.1095000000002
278.697800000005 -6.16779999999956 -9.24370000000032
279.825200000006 12.3649000000004 -14.6273000000003
302.029500000006 1.07640000000048 -11.8402000000003
309.456600000006 12.6471000000004 -14.0518000000003
318.511900000005 17.1945000000004 -12.3606000000003
317.583900000006 27.3891000000005 -16.8593000000003
328.521600000006 27.5216000000004 -0.507100000000278
319.598400000006 8.28830000000049 -7.1289000000003
326.522600000006 16.5144000000005 -19.2662000000003
1.09849999999697 11.0956999999998 -8.46769999999932
7.49110031475884 8.47595333504333 -7.60262662776411
178.528999999993 33.3810999999993 -0.535599999999488
199.416599999999 35.8463999999999 -4.06159999999974
228.511800000003 30.1744000000003 -4.48429999999999
239.893700000003 28.3118000000001 -7.12820000000001
253.636300000003 40.9017000000001 19.0893
262.411500000005 29.6197000000003 -2.66150000000014
268.235000000005 33.0294000000003 -10.0554000000002
274.967400000005 53.4219000000003 -7.31440000000011
278.523800000005 36.5682000000004 -13.1421000000002
299.907900000006 50.7245000000004 -9.98150000000022
307.713700000006 40.6054000000004 -13.8968000000003
317.659800000006 36.3424000000004 -11.0104000000003
318.022900000007 44.7002000000005 -6.42700000000025
326.370700000006 37.6980000000005 -19.2902000000003
-1.92660000000258 1.07319999999984 0.0524000000006339
-0.592100000003018 6.63509999999982 -0.145799999999399
158.85469999999 18.607199999999 21.4577000000006
229.478800000003 36.6480000000002 -2.20989999999996
204.1192 38.7407 -6.38599999999981
127.248399999989 8.29079999999879 17.9332000000005
272.627600000005 22.6923000000003 30.4506999999998
296.003300000005 23.8891000000003 28.4390999999998
311.606000000006 49.6700000000004 3.0440999999998
315.322900000006 25.8126000000005 23.8491999999997
321.614000000006 38.5109000000004 -4.67860000000027
-1.03590000000245 -1.63690000000014 -0.0677999999993832
1.85329999999775 -5.93200000000004 -2.23699999999938
158.22379999999 3.47679999999901 18.7796000000004
231.048400000003 3.27940000000026 -3.8419000000002
209.367400000002 -0.415599999999257 -10.7491000000005

313.434000000006 1.25590000000049 2.84059999999968
324.039400000006 11.6909000000005 -5.04050000000033
ID=MYRtriFEMBRABMNH377133

LM3=54
1.03690000003596 -7.7909999999992 -13.9002999999986
6.02850000003562 -5.45889999999516 -12.302600000001
220.085600000009 7.09139999999991 -4.81399999999923
201.434200000006 -2.70010000000017 -5.18029999999909
189.15280000001 -4.18739999999988 -3.56499999999948
238.809900000003 7.02299999999931 -8.47859999999895
247.333800000001 11.4106999999992 -9.90739999999889
255.060600000002 -0.804400000000744 16.1284000000013
278.4599 23.5545999999991 -15.5966999999987
268.357799999999 15.062299999999 -4.80059999999876
277.1197 13.6693999999993 -10.8431999999988
285.798200000002 -8.34720000000072 -12.8438999999985
290.973 11.5977999999993 -15.3043999999986
313.819600000003 1.05219999999914 -11.4861999999985
320.273800000002 11.5109999999991 -12.0974999999984
331.424300000003 15.8232999999991 -12.3958999999985
330.751600000003 27.2938999999989 -18.0425999999985
339.703900000005 27.3506999999991 -3.19639999999846
331.190500000003 8.9120999999991 -8.20399999999842
339.583100000004 18.359699999999 -21.9365999999985
-0.710399999964767 8.31640000000185 -13.0745
5.00920000003468 5.79160000000185 -11.8872
183.825500000009 34.9816 -2.90459999999954
198.048000000006 36.3815999999995 -3.63059999999939
231.111400000002 30.6379999999993 -5.30169999999915
245.373600000001 29.9228999999993 -8.55889999999907
252.357500000002 42.9887999999991 17.7266000000009
266.0821 30.2272999999989 -3.0902999999989
274.2987 32.0340999999991 -11.2956999999989
281.233000000001 56.407799999999 -11.199099999999
289.023 36.458699999999 -13.9870999999989
310.249300000002 50.833899999999 -10.8711999999989
316.160900000002 42.1555999999987 -11.1369999999987
329.079200000003 38.697399999999 -11.7476999999987
329.184200000004 43.9834999999987 -7.22819999999863
338.691100000005 38.0326999999991 -21.7286999999986
-3.91789999996435 6.42650000000217 -3.82499999999988
-3.82809999996418 0.0318000000019794 -1.28669999999993
157.056800000013 20.3323000000003 21.0700000000005
234.432800000002 36.7091999999992 -4.23339999999918
210.495600000005 40.2110999999995 -6.80539999999941
127.502500000017 9.41500000000067 21.4321000000004
275.397100000002 21.8035999999991 32.4275000000012
305.245000000001 23.7704999999988 28.7843000000014
318.9153661518 50.6673397357256 2.32429499764664
330.151600000004 26.371499999999 22.4362000000014
333.591400000004 38.0739999999989 -3.38929999999866
-3.56209999996415 -1.54029999999791 -1.36689999999994
-2.91819999996417 -8.08289999999807 -4.27909999999988

158.240600000012 5.76870000000006 19.7475000000006
238.654300000002 3.71129999999943 -4.81079999999895
216.543400000005 -4.14290000000041 -8.82989999999904
323.231000000004 2.55849999999923 0.417900000001533
333.434700000004 14.641699999999 -4.44429999999838
ID=MYRtriUNKUNKBMNH990517

LM3=54
2.4733 -8.3763 -14.4729
10.6671 -3.6623 -11.444
195.2727 -1.4452 -1.5056
221.7643 -2.5403 -5.5452
180.5103 14.2324 1.0109
248.2717 9.1242 -5.5941
264.9407 12.7696 -10.3371
271.1701 -1.3175 17.651
294.4622 23.9899 -19.0336
285.8475 14.3102 -5.0215
293.503 12.5966 -10.309
302.761 -9.2858 -13.3886
307.6572 12.7091 -15.5055
331.6221 0.8847 -12.8536
337.4784 12.2091 -13.4923
348.952 17.1652 -14.763
348.4359 27.854 -21.8668
359.5572 28.1677 -2.9796
348.636 7.7957 -9.2407
359.0063 18.4768 -22.7585
1.0792 9.4561 -12.1149
9.429 5.5809 -10.1507
193.5905 32.7045 -1.5818
219.2494 37.7646 -6.3454
245.9517 30.675 -6.2658
262.8771 29.5447 -9.8863
266.5346 44.5043 19.2594
284.2338 30.3322 -4.8739
292.7739 33.6492 -10.6728
297.7082 57.2534 -12.3932
306.5719 36.3289 -14.9269
327.6733 51.5278 -13.0921
335.7195 41.5729 -12.7225
347.6858 37.9571 -14.5802
348.1682 45.5477 -8.3335
355.7993 37.5866 -22.897
-3.2583 0.1063 -1.2758
-1.6803 6.1455 -1.8498
167.4186 21.1241 24.8925
250.513 37.1748 -5.0758
227.0907 41.4783 -11.4133
129.2228 10.9034 24.6555
322.6084 25.4454 30.754
322.584 25.5858 30.787
335.9687 53.2845 4.5705
348.7707 27.8226 23.3024

350.2643 40.8471 -3.8258
-2.1104 -1.2435 -2.0764
0.7384 -7.3881 -3.3769
169.668 6.6847 24.4538
252.3623 1.833 -4.2645
231.7333 -4.0686 -11.389
338.8266 0.4203 3.6161
352.3712 14.6491 -4.4877
ID=MYRtriUNKBOLBMNH282975

LM3=54
4.1927 -9.6196 -12.3221
11.5761 -5.3057 -10.7521
193.3849 -2.6553 6.9461
221.6883 -4.1527 0.3018
186.4746 13.4401 6.2208
251.9045 6.4983 0.278
266.1362 11.0802 -5.3063
278.1091 0.5501 21.8884
300.7603 21.5413 -18.9436
289.5542 12.6049 -2.0169
299.8112 12.6437 -10.1746
309.0855 -11.3278 -10.4583
312.4965 11.0168 -14.6982
337.7766 0.2825 -13.5577
344.9151 10.1176 -13.2151
355.9688 15.1705 -14.9817
354.7336 26.7233 -20.329
365.0103 27.9432 -5.9266
355.9576 7.7494 -10.686
366.0529 16.6423 -24.219
2.0398 8.4761 -12.125
10.3268 5.568 -10.7309
194.3199 30.6336 5.3484
223.3954 35.9814 -1.1159
250.4366 29.263 -0.8483
263.9372 27.3399 -4.6905
275.8638 42.4408 20.4253
288.9172 29.6004 -2.5539
297.6364 31.384 -11.0843
302.5775 56.9261 -11.6652
310.768 36.23 -14.9995
333.3071 50.3601 -14.2115
342.3832 42.3573 -13.9227
354.0737 37.4693 -14.2632
353.8243 45.7427 -11.0118
363.3186 37.478 -24.182
-0.8825 0.5029 0.1531
1.1761 6.8962 -1.1357
170.1493 19.388 28.597
256.7587 35.8762 -1.1547
232.89 39.1457 -6.3397
136.6713 11.0114 27.2241
289.9434 23.0676 32.9953

331.3642 25.5523 27.2587
343.7489 53.2696 -2.3055
356.7104 28.2734 22.3754
356.6633 42.7014 -7.7455
-0.8071 -1.536 -0.1716
2.5121 -6.8364 -1.9139
171.4021 7.772 29.6693
257.5866 1.6576 0.3558
233.6277 -5.8276 -4.8801
349.0304 0.6842 2.1271
359.449 12.7033 -6.904
ID=MYRtriFEMPERBMNH852220

LM3=54
-4.56219999999689 -8.33480000000083 -19.0685999999983
0.0154000000051435 -6.42320000000028 -16.0741999999979
185.563200000004 -3.41729999999979 6.35090000000057
219.162700000003 -3.69839999999995 -1.78309999999958
186.358200000004 16.1125000000002 6.22120000000061
254.248200000002 6.00419999999997 -2.83939999999973
261.6847 11.8649999999999 -5.39159999999976
281.3881 1.88839999999991 23.0178000000003
294.986199999999 24.5682999999998 -18.5079999999999
288.728499999999 14.4344999999998 -3.37669999999983
295.173899999999 13.8721999999997 -10.8956999999999
307.671799999999 -7.4920000000002 -7.35019999999978
312.215299999999 11.0756999999998 -16.1076999999999
335.618299999997 -0.100600000000281 -12.2455999999998
343.687299999998 11.3829999999997 -13.8856999999999
353.726599999997 18.1831999999996 -13.4469999999998
353.661799999997 27.9910999999996 -19.4460999999999
365.140799999996 29.1259999999995 -4.60669999999975
356.391699999997 9.88559999999958 -9.62499999999978
364.872199999996 18.9616999999996 -23.7502999999998
-5.80459440218301 7.81509028881589 -17.9272291456441
1.2735443092885 4.94297786769579 -15.5265278199387
180.673400000004 33.5299000000002 5.95870000000056
216.983000000004 39.8836000000003 -4.58579999999971
254.674400000001 32.8529 -4.07069999999979
263.2679 29.9569999999999 -7.51199999999983
277.5141 44.6032999999998 21.8027000000002
287.759 31.8984999999998 -3.28119999999989
294.026000000001 33.9496999999999 -9.74770000000001
303.097299999999 57.2312999999998 -7.58199999999989
309.897399999999 37.1048999999997 -16.5662
332.665099999998 53.3209999999997 -13.1015999999999
341.222699999999 42.2719999999998 -13.7391999999999
352.793899999998 37.3330999999996 -14.5397999999999
353.417799999996 46.0102999999996 -10.3429999999998
362.137999999998 39.0322999999997 -25.2543999999999
-9.49501083050465 -0.650775346241728 -4.94822263661421
-8.50089234784252 6.02398984197369 -7.08154565155834
164.591400000005 21.6234000000002 30.7906000000007
253.724700000001 39.7234 -2.72739999999985

224.094100000003 42.7720000000001 -7.79359999999973
142.803900000004 11.8442 32.6037000000009
300.245499999998 24.9191999999997 31.4536000000003
328.790399999999 27.4886999999998 28.6956000000003
344.408199999997 53.2131999999997 2.28890000000023
353.070699999996 27.5994999999995 23.1378000000003
357.653299999996 40.0674999999996 -4.87249999999977
-9.32552400433518 -2.41109060416838 -5.07582190192279
-6.39959999999476 -8.44580000000027 -6.91559999999811
168.367200000003 7.42270000000013 31.3172000000007
255.145900000002 1.68939999999997 -2.83219999999974
227.329900000004 -5.04239999999985 -5.78769999999959
348.230299999997 0.53399999999967 0.638100000000257
358.834799999996 16.6464999999996 -5.72829999999976
ID=MYRtriMALBRABMNH377131

LM3=54
3.8072000000332 -7.07019999999631 -12.9826000000001
10.4642000000332 -4.47239999999574 -10.2999999999999
188.418100000015 0.376500000001304 5.35810000000014
214.293500000014 -1.36919999999899 -2.18989999999984
178.922200000017 17.0248000000014 8.59500000000016
242.399600000013 8.54350000000083 -2.60649999999981
256.890300000012 12.9611000000008 -6.24009999999979
262.387900000011 1.21820000000082 17.4449000000003
288.741500000011 23.9551000000007 -15.1519999999996
277.783700000011 15.1246000000007 -3.1810999999997
286.15440000001 13.4048000000007 -9.8901999999996
293.00420000001 -7.91939999999915 -11.8049999999995
302.606100000009 12.9809000000006 -16.1153999999995
324.455600000008 0.684700000000655 -12.8963999999993
332.609500000006 11.7035000000006 -12.1436999999992
341.621200000006 17.5866000000006 -13.8664999999991
340.536000000006 28.1331000000004 -18.4949999999991
352.359600000006 27.9821000000005 -2.26809999999906
344.353500000005 8.84710000000062 -9.59109999999916
349.012500000006 19.1517000000006 -22.2775999999991
5.1237000000319 8.83740000000376 -9.97789999999976
8.39940000003128 7.13780000000361 -8.13529999999977
186.617500000016 35.0581000000013 4.15750000000011
211.969700000013 39.6880000000009 -3.68659999999986
240.367100000013 32.5082000000009 -3.80729999999982
255.032200000012 28.7316000000008 -6.73039999999974
260.664300000012 43.9451000000007 16.5880000000002
277.162200000011 30.0294000000007 -3.50979999999966
284.517300000011 32.9600000000006 -9.94369999999959
290.280497139623 55.2391376636201 -11.4058280575304
300.170300000009 36.1565000000005 -15.1945999999994
320.278000000008 51.1948000000004 -11.7359999999993
329.886700000006 41.6647000000003 -11.1588999999992
339.692700000006 39.6352000000004 -12.4770999999991
341.478400000006 46.9380000000004 -8.3227999999991
346.596400000006 38.0363000000005 -21.3781999999991
-1.21939999996778 -0.125999999996298 -1.36009999999976

0.0261000000322098 6.73850000000375 -1.37439999999972
170.873700000017 21.0712000000016 30.0384000000001
246.725100000013 38.6626000000008 -3.01929999999981
219.925200000014 41.136100000001 -6.75839999999985
147.20980000002 15.0296000000019 30.3674000000002
289.91420000001 23.3641000000007 31.3193000000004
318.833600000009 26.2080000000007 27.6593000000006
332.317500000007 51.7267000000004 -1.60449999999918
341.026600000007 27.7884000000006 23.6489000000008
344.888900000006 43.8897000000004 -6.79749999999909
-0.852599999967882 -1.73839999999642 -1.28069999999971
2.91450000003183 -6.62719999999615 -3.18149999999973
173.481500000018 12.5282000000016 31.1612000000001
248.252200000013 4.05810000000094 -1.04469999999982
223.206400000013 -2.75349999999893 -5.93649999999986
335.292800000007 2.46590000000067 -1.84639999999925
346.558400000006 14.2196000000006 -6.32409999999913
ID=MYRtriMALBRABMNH377176

LM3=54
3.3497000000255 -7.42969999999318 -11.5182999999993
9.45050000002577 -4.15449999999405 -10.9096999999994
187.312800000011 -3.83419999999709 -3.86419999999927
203.376200000009 -3.33819999999752 -5.90599999999914
164.889200000012 13.168200000003 -0.79709999999946
228.030100000007 7.84020000000198 -6.95059999999901
240.945800000006 12.5467000000019 -10.3670999999989
252.508600000006 -1.18329999999813 17.4261000000014
268.656200000004 23.2360000000014 -18.9677999999989
264.178900000006 13.8326000000016 -6.70569999999878
270.649900000005 11.7991000000017 -12.2724999999988
277.884500000005 -10.7432999999984 -14.9455999999986
283.324800000005 12.1877000000014 -16.7990999999987
307.474500000005 0.2570000000013 -12.9244999999984
314.802100000004 10.9752000000012 -13.9825999999984
324.684700000003 16.4523000000011 -12.0899999999985
325.088900000003 25.992400000001 -16.9842999999985
334.441800000002 26.6111000000012 -1.80209999999839
325.319600000004 8.39880000000133 -7.11239999999846
335.018300000003 17.5472000000014 -17.4003999999985
1.3578000000256 10.1783000000055 -9.33999999999986
8.94680000002437 7.44850000000528 -9.00909999999987
185.950200000012 34.3292000000028 -3.52839999999948
201.52870000001 35.9079000000024 -5.43299999999938
226.772600000007 28.6517000000019 -7.40699999999922
238.935600000006 26.6796000000017 -10.8680999999991
249.904100000007 41.0304000000018 17.677500000001
263.168200000006 29.5455000000018 -5.82099999999887
269.312000000005 32.0425000000016 -13.3650999999989
274.188500000005 56.0718000000015 -14.921499999999
282.825200000004 33.8370000000015 -17.6888999999988
305.150100000002 49.7663000000009 -12.6757999999987
312.873800000001 39.4055000000009 -12.9461999999986
324.565100000002 36.087400000001 -11.3242999999985

322.080300000002 44.4452000000009 -6.69019999999859
333.651700000003 35.324000000001 -18.1113999999985
-0.798399999974403 3.37750000000549 0.513700000000099
0.0587000000257589 6.9936000000055 -0.293599999999812
155.596000000013 16.0491000000032 22.8366000000005
229.069300000007 36.9087000000019 -4.63539999999917
211.245100000009 39.3264000000023 -9.20809999999935
132.620600000016 9.28600000000371 22.5641000000005
275.200728997679 21.0910596539934 29.7990633908475
298.892200000005 23.3662000000014 27.7336000000013
317.295400000002 50.347000000001 1.4782000000014
323.183400000002 26.6370000000014 22.1815000000014
327.451000000002 37.685600000001 -4.84879999999855
-0.294099999974538 -4.3849999999947 0.183000000000154
0.602000000025659 -7.10239999999461 -1.72259999999962
156.196100000013 6.33540000000311 21.7567000000005
225.372100000007 -7.27029999999788 -4.83359999999891
212.800900000009 -4.57739999999749 -10.244799999999
320.046700000005 0.804500000001604 0.996500000001601
327.274800000004 14.1291000000013 -3.23989999999846
ID=MYRtriUNKPERBMNH412417

LM3=54
5.04670000000117 0.664099999999589 1.38589999999975
7.07340000000131 -0.547900000000368 1.39199999999978
24.0929000000004 1.20799999999976 -2.62110000000011
27.1469000000003 3.1623999999998 -3.31600000000008
25.2624000000003 7.93159999999977 -2.3539000000001
29.2230000000002 5.3069999999998 -3.44360000000008
27.6124000000002 5.04859999999981 -3.85240000000009
33.4976000000005 0.536099999999783 6.70329999999991
39.4415000000001 12.4825999999999 -8.33400000000006
36.0265000000001 10.4107999999999 -1.97730000000004
43.4707 10.7573999999999 -4.94800000000005
41.1064000000001 1.12469999999978 -6.06010000000006
48.6753000000002 10.3729999999999 -6.19580000000006
52.4122000000003 5.5588999999998 -6.58310000000008
58.5805000000007 12.4203999999998 -6.52160000000013
62.6527000000007 13.0440999999998 -5.27480000000014
62.784800000001 20.2615999999998 -6.17340000000015
66.7274000000011 21.0059999999998 1.11419999999983
65.2238000000009 11.3956999999998 -2.98980000000015
65.1128000000009 17.4125999999997 -5.83750000000017
4.97150000000137 3.28929999999962 0.757499999999789
5.42130000000128 5.05329999999961 0.517899999999799
20.6024000000005 13.0216999999997 -2.93540000000012
23.9582000000004 13.8089999999998 -3.36100000000009
27.1723000000003 13.6795999999998 -2.95960000000009
25.6638000000003 12.5678999999998 -3.78930000000007
29.2546000000007 20.6745999999998 6.58829999999989
36.4754000000001 13.2029999999999 -1.24060000000005
39.1919000000002 16.2375999999999 -3.50360000000006
35.4694000000005 23.1424999999999 -6.2245000000001
46.7871000000004 19.9315999999999 -5.75840000000009

47.3380000000006 26.4857999999999 -5.67270000000012
54.992300000001 24.1545999999999 -6.22010000000015
58.5377000000011 24.8271999999999 -5.77620000000016
60.2889000000012 28.1427999999999 -2.91050000000017
63.6425000000011 24.3430999999998 -5.67210000000017
2.93730000000149 1.23789999999955 6.82299999999977
3.43390000000146 4.56399999999956 5.81169999999976
17.0244000000009 10.8651999999997 7.27359999999986
23.9689000000004 14.2928999999998 -1.0756000000001
25.0411000000004 16.3719999999998 -3.7704000000001
26.9185000000007 8.86119999999972 11.1562999999999
46.2220000000008 15.4808999999998 14.9923999999999
55.8108000000009 18.3093999999998 13.2035999999999
57.6811000000012 32.0661999999999 1.05549999999982
63.0143000000011 21.3292999999998 9.01039999999984
61.7613000000012 27.5229999999999 -0.266700000000177
2.0412000000015 0.274399999999566 6.11129999999979
5.14930000000148 -2.12150000000043 5.15129999999979
21.1684000000008 0.987599999999648 7.01699999999984
29.3563000000002 1.70909999999978 -2.17410000000009
30.7068000000002 0.511599999999778 -3.6275000000001
65.8916000000009 8.95129999999977 1.03769999999986
66.7684000000009 15.6059999999998 -0.0666000000001425
ID=PHAtetUNKEGUBMNH112135

LM3=54
6.08900000000253 0.318300000000554 0.953399999999764
6.85780000000237 -1.18219999999951 0.615699999999814
24.8832000000017 1.44290000000045 -3.87140000000011
28.2015000000015 3.42880000000042 -5.20830000000007
24.6560000000015 7.76880000000036 -3.13300000000009
30.1057000000015 4.96340000000042 -4.61460000000008
29.1200000000015 4.86920000000042 -5.57320000000008
36.3223000000015 1.21630000000048 5.78259999999989
39.8903000000008 13.0021000000002 -10.0808
36.7416000000009 10.4192000000003 -3.33390000000006
42.0129000000007 8.84720000000022 -5.10390000000003
41.781900000001 1.18260000000032 -7.43560000000008
49.6235000000004 10.8944000000001 -7.77660000000007
53.3723000000006 5.59610000000027 -7.40560000000008
61.3887483735526 13.6521914971797 -7.03766530950771
63.3244000000002 14.1991000000001 -5.83970000000007
63.4812000000001 21.1667 -6.80020000000008
68.0725 22.1728000000001 0.211099999999932
66.8827000000002 13.4559000000001 -3.0822000000001
66.7487000000002 18.0720000000001 -7.10020000000009
5.35950000000238 2.87820000000051 0.898199999999824
5.56500000000239 4.64860000000051 0.201499999999811
21.1322000000015 12.8052000000003 -3.92160000000009
25.5100000000014 13.8463000000003 -4.12870000000007
28.4912000000013 14.1183000000003 -3.58540000000007
26.4833000000013 13.6368000000003 -4.57650000000007
30.6943000000012 20.2157000000003 5.50809999999993
37.0636000000008 13.5600000000002 -3.03460000000003

40.4905000000006 16.7750000000001 -4.72860000000001
35.3714000000008 22.9364000000001 -8.16000000000001
46.4259000000002 20.5788 -7.4371
46.9976000000001 26.8802999999999 -7.62899999999998
56.062 26.5502 -7.10580000000004
60.5699000000001 27.656 -4.66640000000006
62.6812000000001 29.4473 -2.94330000000009
65.3444000000001 25.3521000000001 -6.21290000000005
2.21290000000256 0.387400000000533 6.80669999999979
1.62710000000257 3.47560000000055 6.04829999999978
16.3474000000019 11.0202000000004 7.13199999999986
25.0659000000014 14.4439000000003 -2.35710000000008
25.6789000000013 16.9519000000003 -6.21570000000008
24.9516000000016 8.69410000000044 10.4189999999999
46.9585000000007 15.5909000000002 13.6948999999999
56.5228000000004 18.9715000000001 12.6666999999999
58.4142000000002 33.1865 0.243899999999944
64.3635000000002 21.5289000000001 8.62889999999992
64.7402000000001 26.9762 0.66309999999993
2.37200000000256 0.124500000000541 6.78229999999979
3.61820000000256 -2.21459999999945 5.5733999999998
20.442700000002 1.04750000000051 6.73709999999982
29.9742000000016 2.71760000000046 -4.04400000000009
32.0954000000014 1.27680000000041 -7.44090000000011
67.3152000000002 8.98280000000018 -0.759800000000092
68.1349 17.4060000000001 0.288399999999922
ID=PHAtetFEMGHABMNH022156

LM3=54
3.96269999999758 -0.30930000000042 1.36439999999989
5.35940000000007 -1.82909999999971 1.44289999999991
25.0363000000002 0.738000000000234 -2.98550000000004
28.3188000000001 1.74360000000031 -3.7903
26.1082000000001 8.11830000000018 -2.79950000000002
30.1361999999998 4.11270000000008 -3.5081
29.8502999999996 4.06190000000016 -4.32549999999999
36.8569999999998 0.3967000000002 7.7528
40.8456999999999 12.4774000000001 -8.7468
40.0892999999998 10.1056 -2.84209999999999
44.1880000000003 9.59250000000015 -5.80819999999999
43.7611000000004 0.100600000000213 -5.79920000000005
51.5906000000005 8.86190000000021 -5.96070000000002
59.3161000000006 4.92570000000028 -5.69940000000005
64.7328000000002 11.9802000000002 -6.23120000000006
67.9625000000006 13.2709000000003 -5.04820000000007
65.4855000000003 19.3081000000002 -7.19270000000007
72.8232000000003 21.4326000000002 -0.527700000000045
71.9466000000005 11.6573000000003 -2.95220000000007
71.9273000000005 15.9099000000003 -5.18250000000006
3.40289999999875 2.69019999999997 1.17549999999993
4.22349999999913 3.91490000000008 1.00619999999992
21.6219999999999 13.7379000000001 -3.46720000000002
25.2552999999999 14.5084000000001 -4.24170000000001
27.6755999999998 13.7126 -3.92319999999998

28.0685999999998 13.4109000000001 -4.78719999999999
32.3613999999998 20.4747000000001 7.0746
39.9153999999999 14.669 -2.59149999999998
42.6625000000001 17.2339000000001 -6.0586
36.9151999999999 24.5224000000001 -5.7285
49.4660000000001 20.5061000000001 -6.99279999999998
51.9401000000001 27.8774000000001 -6.73699999999999
62.0844000000002 26.3595000000002 -5.42410000000001
64.8556000000002 26.4527000000002 -4.86730000000002
68.0384000000003 30.4158000000002 -1.68530000000004
68.6492000000004 25.4844000000003 -6.08390000000006
1.29209999999964 -0.0748999999997276 6.70299999999988
1.37169999999959 3.14200000000024 5.8637999999999
18.1988999999997 10.9483000000002 9.03799999999996
26.3920999999998 16.0247000000001 -3.2192
27.2406999999998 18.0949000000001 -4.71079999999999
27.2703999999998 8.47790000000015 11.7328
50.1999000000001 16.5025000000002 14.4959
60.8388000000002 18.7429000000002 12.67
63.6774000000002 33.0168000000002 0.240899999999976
68.0462000000003 21.2952000000003 8.89459999999996
68.7900000000002 27.6004000000002 1.25119999999996
2.52179999999968 -0.253299999999735 7.19669999999986
3.78629999999972 -2.57719999999974 5.95909999999985
21.8029 1.50300000000025 9.09039999999993
31.8128999999999 1.61730000000017 -3.15770000000001
33.6965999999999 0.330100000000156 -4.18770000000001
71.5782000000006 7.36780000000032 0.697899999999922
72.5172000000005 15.5744000000003 0.954799999999948
ID=PHAtetUNKUNKBMNH412110

LM3=54
4.21551536535096 0.162866290909222 1.91283264707029
5.74700000000126 -0.79759999999961 2.08329999999997
25.5462000000009 0.780900000000463 -2.4069000000001
26.8143000000008 1.84160000000045 -2.9081000000001
25.8291000000008 7.58230000000051 -2.20250000000009
28.8967000000009 4.62940000000049 -2.76130000000011
28.4637000000008 4.72590000000047 -3.88220000000009
34.6986000000012 1.25600000000045 6.84039999999989
39.1465000000011 11.7194000000005 -8.88810000000015
38.912600000001 10.3031000000005 -2.47240000000014
43.6730000000012 9.51990000000051 -5.41120000000016
42.4512000000013 0.121000000000492 -6.00270000000016
50.6251000000016 8.86560000000046 -6.74320000000017
53.4080154342727 3.97264827761573 -6.08665897077498
60.072900000002 10.0254000000003 -5.73070000000019
64.8943000000022 10.7828000000002 -4.29340000000021
64.4562000000023 17.6048000000003 -6.11680000000023
68.0651000000025 19.1201000000002 1.91129999999977
67.3244000000024 9.11000000000026 -1.40000000000022
69.1130000000024 14.0659000000003 -5.01340000000023
4.15433216063816 2.69982066522487 2.01311614422065
3.87980000000148 4.07360000000051 2.12699999999994

21.3415000000009 13.7492000000005 -2.67750000000009
23.3347000000008 13.4251000000006 -3.31550000000009
25.5749000000008 12.4625000000006 -2.73810000000011
25.3059000000008 11.6349000000006 -4.02260000000011
28.6983000000012 18.2808000000006 7.03689999999988
38.2278000000011 12.6066000000006 -2.61020000000014
42.4963000000013 15.1805000000006 -5.94120000000016
35.3395000000012 23.2065000000006 -6.00960000000015
47.6062000000015 19.5843000000005 -6.89820000000017
49.0115000000016 25.2535000000005 -6.50860000000017
58.199000000002 22.3888000000003 -6.47160000000021
60.1626000000022 24.6983000000003 -4.27780000000022
61.3898000000023 26.6418000000003 -1.31360000000023
64.7911000000024 23.3434000000002 -5.40490000000022
2.73260000000152 1.39400000000045 7.59119999999997
2.50990000000156 3.25500000000046 7.02709999999995
17.7512000000011 10.8704000000005 8.12119999999994
23.7544442401896 14.1659009404238 -1.69256607497081
25.8277000000009 16.3145000000006 -4.23030000000011
28.9650000000012 9.14380000000048 11.7116999999999
47.9324000000017 14.0452000000004 13.6848999999999
57.4908000000021 15.9412000000003 11.7620999999998
59.1803000000022 30.3974000000003 0.554799999999781
64.7855000000025 17.9533000000003 8.54499999999979
65.3885000000023 24.0974000000002 1.39779999999978
2.50940000000152 0.527100000000444 7.78849999999996
3.84830000000149 -1.88789999999958 7.09379999999996
20.7921000000012 1.37950000000045 8.26089999999993
27.9771704185421 1.76211127854279 -1.35476871171342
30.3896000000009 0.57290000000046 -4.56450000000012
66.2996000000024 4.66270000000029 0.724299999999774
68.9702000000025 12.5425000000003 0.751299999999781
ID=PHAtetFEMGABMNHN958194

LM3=54
5.58200000000235 -0.288499999999423 1.72669999999979
6.88080000000226 -0.872299999999391 1.0791999999998
25.1328000000016 0.748600000000513 -2.21810000000017
25.5392000000015 1.25470000000049 -2.65130000000016
22.8234425612787 6.64257241698852 -1.60898415136741
26.2443000000013 3.92070000000045 -2.15210000000016
26.8727000000014 4.38640000000049 -3.57290000000017
33.6942000000011 1.01880000000043 8.63859999999982
36.4404000000006 10.8927000000003 -7.54220000000014
36.7580000000008 9.67180000000037 -2.08000000000016
41.8120000000007 8.56490000000036 -4.67150000000014
40.1731000000011 -0.00059999999954985 -5.33370000000016
48.8943000000004 8.99970000000031 -5.68560000000015
52.4245000000005 3.96210000000037 -5.68860000000017
59.2459000000004 10.6738000000003 -5.68260000000017
63.9120000000004 11.9924000000003 -4.30720000000018
61.5842000000004 18.2708000000003 -6.64440000000018
66.2596000000003 19.8656000000003 0.0362999999997751
66.5340000000006 11.1513000000004 -1.66540000000021

66.4296000000005 14.5305000000004 -5.9294000000002
4.27080000000211 3.38060000000054 1.87789999999985
4.42390000000203 4.48540000000054 1.68189999999984
20.7847000000013 13.0329000000005 -2.24030000000017
21.9274000000011 12.9090000000004 -2.41450000000016
24.061100000001 11.2411000000004 -2.25350000000015
24.3636000000011 10.5410000000004 -3.27290000000015
28.8128000000009 18.6656000000004 7.72519999999981
36.0854000000006 12.4898000000003 -1.90600000000014
39.2905000000004 15.7092000000003 -5.06650000000013
32.9457000000006 22.7124000000003 -5.55190000000016
45.7033000000003 19.2310000000003 -5.62920000000013
45.5993000000003 25.4618000000002 -6.13470000000015
55.0008000000002 23.4719000000002 -5.95750000000016
58.6640000000002 25.0547000000003 -5.15020000000017
61.1065000000003 27.6279000000003 -2.60400000000021
63.5776000000004 24.5987000000003 -5.7798000000002
2.03480000000239 0.810800000000633 6.84109999999984
2.69880000000232 3.84860000000062 5.82659999999983
16.8281000000016 10.2988000000005 7.60369999999982
24.5984000000011 12.2918000000004 -1.54530000000016
25.799600000001 16.0706000000004 -4.35310000000016
28.0685000000014 8.35980000000047 11.8333999999998
47.1362000000005 13.9710000000003 13.5933999999998
57.1676000000005 16.6705000000003 11.4963999999998
59.1244000000004 31.5953000000003 0.569099999999795
65.2725000000004 19.0713000000003 7.56459999999977
63.6708000000003 26.0234000000003 -0.923900000000205
2.37160000000241 0.283800000000624 6.76709999999985
3.99310000000234 -1.57109999999938 5.16319999999983
19.1300000000018 1.08100000000053 7.81739999999983
26.7864000000015 2.98190000000051 -1.09940000000016
29.3497000000015 0.729600000000521 -4.00190000000017
66.1060000000006 4.99130000000041 1.66599999999978
67.6673000000005 12.1312000000004 -0.700200000000208
ID=PHAtetFEMCONKMMA14620*

LM3=54
4.7674 0.3446 0.401
6.0872 -0.7512 0.4782
23.9597 0.6391 -2.5052
26.4314 2.4311 -3.0391
22.3682 6.8668 -0.9262
27.4254 4.1625 -2.5106
27.5914 4.9133 -3.4534
33.3921 -0.0257 6.3948
36.4137 11.6734 -7.4247
36.2541 9.3499 -2.0875
40.5723 9.4433 -5.1906
40.159 1.189 -6.1938
47.3717 9.3659 -5.9148
50.5171 5.4372 -6.3088
57.9072 11.7916 -5.2589
60.6925 12.4508 -4.3901

61.6959 19.5759 -4.9877
66.1615 20.567 1.5642
64.7099 11.0527 -1.2615
66.9968 15.8329 -3.6492
6.7075 3.2812 0.6434
7.1039 4.6205 0.2509
22.3758 13.6611 -2.3978
25.4955 13.4974 -2.7141
27.3633 12.5341 -2.749
27.3626 12.4914 -3.7842
29.5158 18.8975 6.9308
37.6935 13.3941 -1.9745
41.1902 16.5713 -5.2414
35.5244 22.7366 -5.3562
46.4492 20.2764 -5.5055
46.9424 25.6342 -5.6576
56.6136 24.7325 -5.3119
59.3239 25.5571 -4.2598
62.3614 28.8291 -1.2219
66.3859 25.7235 -2.9951
2.0726 0.5614 6.0021
2.362 3.2447 5.7945
15.5046 9.102 7.9776
22.6458 13.586 -1.4782
24.0055 16.0419 -3.6444
24.3533 7.1756 11.8436
44.8163 14.552 14.6549
53.6356 16.967 13.2459
56.0928 30.9201 3.226
61.2322 19.8857 9.8304
60.5604 27.9701 1.6258
2.391 -0.2953 6.0864
4.0273 -2.3129 5.2131
18.4577 0.2681 7.9906
26.5388 1.8024 -1.6279
29.4646 1.0561 -3.9309
63.916 8.1693 2.4778
65.4424 13.7759 0.7603
ID=PHAtetUNKCAMMfNB37804*

LM3=54
3.47759828693697 0.236385439193221 0.736711264797592
5.21062894433202 -0.851973326742704 0.662528473240153
23.0891000000017 1.26850000000045 -2.89809999999994
25.6743000000007 3.05840000000023 -4.05069999999986
23.4030000000011 8.09910000000028 -2.86239999999996
27.8245000000003 5.19789999999987 -3.33119999999989
27.0149000000008 5.46600000000012 -4.40909999999993
34.2663999999998 0.717499999999841 6.61490000000016
37.8654000000004 12.5287000000001 -7.60929999999987
36.7400000000005 10.0098000000001 -2.14039999999984
41.2021000000003 9.82260000000003 -5.27419999999983
39.8980000000009 1.29330000000019 -5.87519999999984
47.0562000000006 9.68780000000005 -5.82429999999981

50.8438000000003 5.06249999999995 -5.58499999999979
57.8931000000006 11.8891 -5.32569999999975
61.3192000000009 13.4349000000001 -3.77679999999975
61.6074000000009 19.6155000000001 -4.05399999999973
65.4155000000006 20.4965 2.41180000000029
64.2272000000008 10.6395000000001 -0.724899999999731
65.6107000000008 16.1336000000001 -3.28309999999971
3.20840000000103 3.25540000000008 0.668999999999974
3.94780000000102 5.07080000000009 0.613499999999977
18.820900000001 13.7711000000002 -2.57659999999996
22.4776000000009 13.4895000000002 -3.49039999999993
25.8028000000008 12.9367000000002 -3.29029999999992
25.0404000000008 12.7922000000002 -4.38409999999992
27.5444000000004 20.4602 7.05820000000012
35.3329000000004 13.8259000000001 -2.26549999999986
39.3631000000005 16.4344000000001 -5.73009999999985
33.1913000000006 23.0206000000001 -6.04489999999987
43.9325000000006 19.3966000000001 -6.24199999999982
44.5996000000007 26.1307000000001 -5.6122999999998
54.2262000000009 24.0657000000001 -5.38309999999976
57.8642000000008 24.9287000000001 -3.74369999999974
59.1862000000008 28.8092000000001 -0.579399999999721
62.6959000000009 24.6960000000001 -3.57069999999972
1.3181000000009 1.21220000000001 6.67799999999997
0.8706000000009 3.82550000000001 5.67459999999998
15.3152000000008 11.0486000000001 7.02310000000003
25.5663000000008 13.6516000000002 -2.42719999999994
24.0288000000009 16.6934000000002 -4.17339999999994
23.2656000000004 7.79929999999998 11.1082000000001
45.8548259649458 15.515328310666 14.4414731299088
55.2434000000004 17.8428 12.7973000000002
56.8166000000008 32.0925 1.22640000000026
61.4100000000005 19.4883 9.72890000000027
60.6944000000007 27.7088 1.72290000000028
1.0518000000009 0.409700000000001 6.71229999999996
2.27260000000096 -2.01409999999996 5.33249999999998
18.9392000000009 0.580200000000107 6.78380000000003
26.7111000000009 2.72870000000016 -2.7867999999999
28.7341000000011 1.37090000000026 -4.6530999999999
63.9901000000007 6.50780000000003 1.52630000000027
65.2172000000007 11.3805 0.912800000000283
ID=PHAtetUNKEGUMfNB37968*

LM3=54
3.98239999999449 -0.242500000001675 0.239300000000202
4.69979999999476 -0.866600000001557 -0.122899999999713
24.2361999999964 1.20329999999896 -3.60249999999974
25.4270999999967 1.50259999999906 -3.43469999999977
23.1371999999968 7.23619999999908 -2.44759999999979
26.0433999999969 4.23509999999913 -2.96369999999978
25.9575999999969 3.83909999999914 -4.27489999999978
32.3246999999964 0.472999999998951 7.18090000000028
36.9662999999974 10.9254999999994 -7.97819999999975
35.5563999999973 9.95729999999932 -2.38089999999975

40.9711999999975 9.80159999999934 -4.92059999999973
39.8016999999969 0.441899999999135 -5.45889999999971
47.7974999999974 9.45379999999932 -6.6187999999997
51.362399999997 5.39299999999917 -6.07249999999965
57.951599999997 11.4912999999992 -5.09529999999962
61.8639999999968 12.7671999999991 -4.05759999999957
61.6595999999969 18.8792999999992 -6.41319999999958
65.6708999999964 19.967599999999 -0.69489999999952
65.0250999999965 11.875799999999 -2.23709999999954
65.1686999999964 15.456899999999 -6.47129999999953
2.29089999999482 2.79399999999844 1.09480000000029
2.23459999999492 3.61699999999849 0.448900000000288
20.0230999999967 12.8432999999991 -3.24489999999979
21.863899999997 13.5111999999992 -3.43299999999979
23.6805999999971 12.0003999999992 -3.46289999999979
23.5332999999971 11.5301999999992 -4.44089999999979
28.5614999999966 19.1027999999991 6.57790000000025
34.7382999999975 12.8585999999994 -2.09829999999975
38.1718999999976 14.7766999999994 -5.09579999999975
33.1110999999972 21.9051999999993 -6.31679999999974
44.8479999999976 18.5650999999994 -5.79289999999971
45.1608999999973 24.1983999999993 -6.68399999999968
54.6208999999972 23.2589999999993 -5.68329999999962
58.0150999999971 25.1420999999992 -4.2473999999996
59.5689999999967 26.4413999999991 -2.80399999999957
62.0237999999966 24.208699999999 -6.48069999999954
1.23869999999423 0.546199999998229 5.47120000000033
0.938899999994346 3.28079999999826 5.14170000000032
15.6041999999958 9.91869999999874 7.19580000000024
24.387999999997 12.7936999999992 -1.29189999999979
24.431399999997 15.1867999999992 -4.45619999999979
23.6360999999961 6.71689999999888 10.5926000000003
46.6700999999965 14.436199999999 11.9716000000003
56.7189999999965 16.352699999999 9.86190000000039
57.8807999999965 29.5579999999991 -1.26879999999958
63.3230999999963 17.765199999999 6.19320000000045
62.9832999999966 24.2699999999991 -1.91819999999957
1.2235999999942 0.508799999998198 5.64620000000032
2.5137999999943 -2.30420000000176 4.83280000000032
18.2680999999956 0.424399999998707 6.86470000000027
26.5802999999968 2.87819999999908 -0.664299999999766
28.2154999999967 0.36679999999904 -4.50199999999975
64.4869999999963 5.99199999999893 -0.705199999999539
66.5804999999964 13.176499999999 -1.16079999999953
ID=PHAtetFEMCARMNHN985101

LM3=54
4.6416 0.2123 1.0483
6.1097 -1.0267 0.8048
22.3757 1.3888 -2.0749
25.1586 3.1324 -2.9222
22.6332 7.7808 -2.2341
26.6062 5.2051 -2.5057
26.6677 5.5663 -3.722

33.4704 0.8187 6.8408
37.1878 12.7572 -8.6809
35.2067 10.232 -2.1451
39.5769 10.2697 -6.2232
37.6531 1.8413 -6.6483
46.2041 10.505 -7.1781
49.6065 5.5449 -7.1858
57.0375 12.7555 -7.7351
60.6078 14.276 -6.1373
60.2235 20.7272 -8.023
64.3315 22.0098 -1.4758
63.4764 12.686 -3.8969
64.5047 17.4435 -6.8089
3.8426 2.851 1.1587
3.9752 4.7852 0.884
18.2502 13.0529 -2.1768
22.09 13.0086 -2.9598
24.2223 12.5869 -2.6161
24.2668 12.1376 -3.7554
25.4239 19.6095 6.8153
34.3474 13.473 -2.6974
37.6779 15.8728 -6.3944
30.6367 22.3036 -6.5951
42.6752 19.6621 -7.4285
43.0721 25.8594 -7.2703
52.4256 25.0172 -6.984
55.9572 25.2141 -6.3595
57.7228 29.1069 -3.8861
61.4375 25.9248 -6.402
2.3803 0.8289 6.2122
1.8305 3.2837 5.7503
15.7344 10.2315 7.9186
23.3419 13.5806 -1.6354
23.9501 17.1089 -4.6603
25.9159 8.7173 10.8449
45.3836 15.8951 13.2747
53.1599 18.2962 10.9797
54.1451 32.63 -0.1267
61.8145 21.4926 6.3779
60.4771 28.1913 -1.9974
2.5933 0.1949 6.2312
3.9601 -1.8649 5.8584
18.6629 0.6995 7.7231
27.0341 3.633 -2.0896
29.1694 0.9186 -4.7795
63.2078 7.9896 -0.308
64.9581 15.6312 -2.4928
ID=PHAtetUNKUNKAMNH105409

LM3=54
5.04370000000049 0.241299999999646 0.489799999999999
7.23540000000066 -0.714400000000326 0.601999999999951
22.898500000001 1.47009999999992 -2.95040000000005
25.148800000007 2.96699999999989 -3.48790000000003

23.155600000001 7.88439999999997 -3.53310000000005
26.0820000000006 4.80699999999991 -3.16440000000002
26.8232000000006 5.57509999999992 -4.44640000000001
31.3907000000006 0.350099999999896 7.24210000000001
35.7161000000011 11.2079000000001 -7.76600000000005
35.6805000000006 9.10819999999999 -2.28990000000005
40.844900000001 9.49500000000007 -5.19060000000004
39.5566000000008 1.2262 -6.28490000000003
47.1358000000012 9.84400000000014 -5.58900000000006
51.092600000001 5.22100000000012 -5.58800000000007
58.1225000000013 10.9250000000002 -5.09210000000008
61.0183000000015 11.8076000000003 -4.37980000000009
61.7701000000016 18.6828000000003 -5.6380000000001
65.4358000000016 19.9371000000004 2.2220999999999
64.5513000000015 11.1424000000003 -1.7661000000001
64.8803000000017 15.8616000000003 -4.7738000000001
4.63570000000059 2.67239999999971 0.967600000000015
4.9957747741562 4.48014435537383 0.724624444116636
19.7674000000008 13.1003 -2.82870000000003
22.3226000000008 12.768 -3.47010000000003
24.5484000000008 12.2631 -3.39240000000003
23.9974000000007 11.9385 -4.12570000000003
24.9710000000009 18.1441 7.48919999999997
34.4523000000008 12.6886 -2.25340000000004
39.0425000000011 15.1688000000001 -5.05640000000006
33.4530000000012 22.1526000000001 -6.08390000000006
44.5456000000013 18.0692000000002 -5.59410000000008
45.2813000000014 24.4276000000002 -5.69730000000008
54.2636000000016 23.0870000000003 -4.3599000000001
57.1610000000017 23.8191000000003 -4.4729000000001
59.5035000000019 27.1027000000004 -1.5347000000001
62.6416000000019 23.1784000000004 -4.91080000000012
1.49690000000106 0.814999999999837 5.97409999999996
1.61710000000103 3.57089999999983 5.26299999999999
14.9059000000007 11.3130999999998 6.55869999999999
22.4500000000008 14.2579 -2.29230000000003
25.2163000000009 17.4126 -4.49210000000004
25.0463000000007 8.1526999999999 12.0826
44.5216000000011 14.1847000000001 13.9208
55.2024000000013 16.8208000000002 12.1942999999999
56.2728000000018 30.0899000000003 2.11849999999989
61.0574000000016 18.4955000000003 9.6118999999999
61.7224000000018 25.4135000000004 0.7330999999999
1.50410000000106 0.406599999999837 5.89859999999997
3.32130000000096 -1.71940000000021 5.19749999999997
19.0064000000007 -0.364300000000192 6.12529999999998
25.6014000000009 2.63609999999993 -1.90300000000005
30.1447000000009 0.668199999999935 -4.78850000000005
62.5312000000015 6.16240000000028 2.35579999999991
65.9296000000015 13.9469000000003 0.314099999999901
ID=PHAtetFEMDRCAMNH53866*

LM3=54
3.67249999999825 -0.294500000001216 -4.8269

4.01969999999798 -0.544600000001102 -4.75350000000006
22.4889999999985 1.08939999999909 -7.07079999999997
23.6168999999985 3.1445999999991 -7.20079999999995
21.1426999999984 6.98849999999912 -6.04939999999996
26.8161999999986 4.40409999999916 -6.17669999999996
24.9323999999985 4.77759999999913 -7.11919999999995
30.3038999999986 -0.550700000000866 4.69840000000004
36.1581999999991 11.7693999999994 -8.81289999999998
34.399299999999 10.1586999999993 -2.82259999999997
39.1473999999991 9.45459999999938 -5.91189999999998
38.1106999999988 1.09229999999923 -7.20039999999996
46.1140999999992 9.50249999999946 -5.22669999999998
50.7018999999991 5.30129999999938 -4.71269999999998
56.6478999999993 12.0777999999995 -2.64549999999998
60.3128999999994 12.9834999999996 -1.44259999999999
60.3874999999996 19.6484999999997 -2.74189999999999
63.2795999999996 20.3183999999997 4.9912
62.7177999999993 10.7712999999995 1.42650000000001
64.0811999999995 16.5170999999996 -1.14769999999999
3.34409999999789 1.95439999999888 -4.57520000000004
3.66529999999784 3.03399999999891 -4.76420000000002
18.3510999999984 12.9680999999991 -6.80869999999997
20.6492999999985 12.1733999999992 -6.62539999999996
23.8236999999986 12.2967999999992 -5.84979999999996
22.8411999999986 11.3764999999992 -6.79689999999996
23.7147999999987 18.5847999999993 5.08070000000001
33.229499999999 13.1405999999994 -3.45459999999997
37.5111999999992 16.3317999999995 -5.83779999999998
31.049999999999 22.2917999999994 -6.67099999999998
43.1416999999994 19.8447999999996 -5.00029999999999
43.6221999999995 25.1342999999997 -4.7739
52.6440999999997 23.7194999999998 -2.6008
56.1750999999997 25.2494999999998 -1.1295
57.3910999999997 28.4017999999998 1.51860000000001
61.7305999999997 24.5006999999998 -1.5064
0.0354999999976271 0.206299999998857 0.00439999999996243
0.0598999999977535 2.86709999999888 -0.676800000000043
12.6944999999981 9.38909999999904 3.80460000000001
21.9370999999985 13.8934999999992 -5.62369999999996
22.5976999999986 16.5644999999992 -7.09119999999996
21.0494999999984 6.98999999999913 8.35590000000002
39.269099999999 13.8117999999994 13.9266
51.0784999999992 17.0163999999995 13.8837
53.8506999999996 31.2190999999998 4.29880000000001
59.4221999999995 19.7090999999996 10.641
60.0382999999997 25.9308999999998 4.824
0.0637999999977686 -0.38860000000119 0.0581999999999676
1.74039999999771 -2.27490000000118 -0.981100000000036
15.7651999999982 -0.143300000000989 4.04120000000001
25.9005999999985 2.25479999999911 -5.30419999999996
28.1686999999986 0.816699999999125 -7.08779999999996
62.3366999999992 6.39019999999947 3.39810000000003
64.0706999999994 14.8555999999996 3.98760000000002
ID=PHAtetFEMDRCAMNH53869*

LM3=54
5.14289999999851 -0.216200000000566 0.535900000000227
7.35190000000082 -0.774999999999927 0.432100000000117
24.2902000000009 0.935500000000074 -2.79679999999998
26.6058000000007 2.58510000000013 -3.38969999999994
23.3481000000007 7.11299999999997 -2.55489999999993
27.7912000000004 4.69139999999992 -2.52029999999992
27.9357000000002 4.90009999999999 -3.90669999999992
33.7790000000004 0.136400000000112 6.74040000000013
37.4144000000004 12.1454 -7.80299999999992
36.7568000000002 9.81629999999993 -2.41619999999992
41.3781000000006 9.38890000000008 -5.18349999999993
39.6313000000008 1.18600000000015 -6.15219999999995
48.6728000000007 10.4046000000002 -5.36629999999994
51.5916000000008 5.11250000000029 -5.94559999999995
59.5240000000004 11.8525000000003 -5.7908999999999
62.7296000000008 12.5210000000005 -4.8567999999999
62.8674000000005 19.0995000000004 -6.31859999999988
67.2942000000005 20.5561000000004 0.459000000000131
66.7313000000007 11.6098000000004 -3.32189999999988
68.2129000000007 16.1198000000005 -5.42129999999988
4.34619999999971 3.21989999999976 0.436700000000184
6.18950000000006 5.89069999999986 0.116100000000165
19.9936000000005 13.8480999999999 -2.72279999999992
22.5422000000004 13.6126999999999 -3.29369999999992
24.2356000000004 12.8674999999999 -3.09829999999992
24.4318000000004 12.7519999999999 -4.23809999999992
27.0217000000004 19.5146999999999 6.75860000000012
34.5775000000003 13.6312 -2.20209999999991
38.3936000000005 16.5406000000001 -4.80209999999992
32.2180000000004 22.0913 -5.78609999999989
45.1250000000005 19.8036000000001 -5.5950999999999
44.1585000000004 24.5279000000002 -6.20229999999988
54.8140000000004 24.0140000000003 -5.92179999999988
58.3718000000004 25.3037000000004 -5.04129999999987
60.5621000000005 28.2054000000004 -2.64779999999986
64.3223000000006 24.6702000000005 -5.48979999999987
3.20570000000065 1.13920000000004 5.51950000000015
2.84470000000055 3.5983 5.06020000000016
16.0769000000005 10.7111 7.57190000000013
23.9662000000004 14.6553999999999 -2.00789999999991
25.1442000000004 17.1841999999999 -4.57229999999991
27.1319000000004 8.85839999999999 11.2697000000001
45.1255000000005 14.4030000000002 13.6508000000001
55.6239000000005 17.2641000000003 12.1035000000001
57.0986000000005 32.1058000000004 0.490200000000143
64.7821000000006 19.7514000000004 7.12710000000014
63.7461000000004 26.3172000000004 -0.493199999999861
3.26050000000066 0.693400000000041 5.65640000000015
4.62050000000066 -2.02249999999996 5.29650000000015
19.2955000000007 -0.0360999999999347 7.62230000000009
27.8779000000006 2.34770000000002 -1.43659999999994
30.5637000000006 0.761600000000018 -4.12199999999993

65.1438000000007 5.80440000000043 0.0482000000001202
67.5155000000006 14.6235000000005 -0.000699999999882683
ID=PHAtetMALDRCAMNH53870*

LM3=54
3.79809999999848 -0.196100000000285 -0.00189999999990254
4.65079999999909 -0.774000000000067 -0.11969999999994
23.4134999999994 1.29429999999993 -3.55909999999999
25.417699999999 2.9500999999999 -3.90719999999994
21.4010999999995 7.37299999999988 -2.84819999999997
26.8252273800159 5.14444705492399 -3.14049232948105
26.6602246621932 5.61093270545506 -4.14405751621208
33.3966999999997 -0.241699999999986 7.14460000000001
37.0360000000001 11.9102000000001 -7.27239999999998
35.7260999999998 9.7203 -2.52439999999996
40.1311 9.3678 -5.12749999999995
38.7301000000001 0.105300000000039 -5.37219999999996
48.1492000000004 9.44080000000029 -5.65029999999996
49.8195000000005 5.0305000000003 -6.21289999999997
58.6626000000004 11.8547000000003 -6.26419999999995
62.0086000000005 12.8373000000004 -5.22809999999996
60.4829000000006 19.1441000000004 -7.18689999999998
66.3325000000006 21.3455000000003 -0.503299999999974
65.6721000000005 12.5532000000003 -2.99249999999996
66.1496000000006 16.8211000000004 -6.15199999999997
3.24219999999932 2.05799999999993 0.186600000000006
3.41959999999959 3.44020000000001 -0.333700000000035
19.3568999999997 13.0964 -3.30580000000003
21.3936999999996 12.9146999999999 -3.9946
23.9222999999996 12.2695999999999 -2.95259999999999
23.5291999999996 11.5164999999999 -4.40199999999999
25.6221999999999 18.1178 6.08339999999998
34.2203 12.1177000000001 -2.87549999999998
37.7076000000003 14.8798000000001 -5.41119999999997
31.6272000000002 21.6771000000001 -5.67090000000001
43.8888000000004 18.5454000000002 -6.14059999999999
44.6034000000005 24.7692000000002 -6.81549999999999
53.8790000000005 24.1176000000003 -6.41869999999998
57.5055000000005 25.1053000000003 -5.68799999999999
59.8969000000007 27.5930000000004 -4.05199999999999
63.2834000000006 24.2125000000004 -6.55189999999998
-0.0495000000000101 0.468500000000137 5.04539999999996
0.514200000000064 3.48820000000016 4.28459999999994
13.8262999999997 9.86869999999999 7.08189999999996
22.9182999999996 14.3138999999999 -2.29249999999999
23.4368999999998 16.7444 -4.39170000000002
24.0241999999997 8.50429999999999 11.3124
44.5403000000003 15.6804000000002 14.6116
56.3896000000004 19.0302000000002 11.8666
57.1570000000006 32.4025000000003 -0.378699999999996
63.1927000000006 20.8640000000003 8.70750000000002
62.0301000000006 27.4108000000004 -1.79689999999998
-0.0239000000000622 0.0970000000001263 5.01699999999996
2.25619999999971 -1.92909999999992 4.7326

16.4596999999995 0.676899999999955 7.09929999999999
26.4735999999994 3.27239999999987 -2.15169999999995
28.3028999999996 1.70279999999992 -4.22749999999996
65.3521000000006 8.87210000000034 1.04390000000004
66.2172000000006 15.5953000000003 -1.02099999999997
ID=PHAtetMALDRCAMNH86840*

LM3=54
6.22449999999706 -0.400400000001135 1.21930000000012
7.66939999999976 -1.88540000000041 0.64900000000003
29.2968000000002 -0.0473000000002413 -3.97680000000004
30.8259000000001 2.35819999999988 -4.61230000000001
28.103 8.15899999999976 -4.00559999999999
32.8790999999998 5.08059999999967 -3.93099999999997
31.9698999999995 5.33609999999974 -5.20419999999999
39.4273999999997 0.173299999999747 6.85990000000005
44.1283999999999 13.1916999999997 -10.6834
42.6164999999997 10.7111999999997 -3.53329999999997
48.4063000000002 10.2304999999998 -6.97799999999997
46.6473000000002 -0.909200000000191 -6.50850000000001
55.7937000000002 10.9606999999999 -7.35329999999998
59.1910000000003 5.59919999999992 -6.95229999999999
67.7000999999997 12.3026999999998 -7.26269999999995
71.4480000000001 14.0219 -5.38939999999997
71.4489999999996 21.3322999999999 -7.66419999999994
74.9122999999997 21.9771999999999 0.428800000000073
74.9240999999999 11.7692999999999 -2.99589999999995
76.6825999999998 17.0142999999999 -5.72589999999995
5.06439999999844 2.87999999999931 1.17400000000007
4.87689999999885 5.17759999999944 0.756300000000045
24.6287999999998 16.4308999999997 -4.10309999999997
26.8218999999999 14.9283999999997 -4.33669999999997
30.5273999999998 14.0312999999997 -4.24379999999996
29.6302999999998 13.3529999999997 -5.10169999999997
32.5640999999996 20.4334999999997 6.89190000000004
41.6689999999998 14.9518999999997 -3.83359999999996
45.9538 17.7547999999998 -7.28699999999996
38.2879999999998 26.2113999999997 -6.72279999999995
52.3321999999999 21.3209999999998 -7.26529999999995
52.1046999999998 26.9149999999998 -7.64979999999992
63.1701999999997 26.4919999999999 -7.38519999999994
67.7940999999997 26.7131999999999 -5.56869999999993
69.0847999999996 30.5523999999999 -3.61159999999992
73.7018999999997 27.3131 -5.46319999999994
2.78779999999934 0.0593999999995454 7.92180000000002
2.60849999999926 3.77059999999953 6.75720000000004
19.2056999999996 11.0444999999996 9.07090000000003
28.1835999999998 17.5437999999997 -3.69819999999997
29.2782999999998 19.5457999999997 -6.18029999999996
31.6618999999996 9.43169999999967 13.0902
53.2193999999998 16.5193999999998 14.6973000000001
63.6510999999998 19.2746999999998 12.5810000000001
67.0298999999997 34.1582999999999 -2.38249999999993
72.0985999999997 21.6800999999999 8.76680000000007

71.6204999999997 28.5869999999999 0.00310000000007316
2.93379999999936 -0.286500000000458 7.75730000000002
4.7785999999994 -2.83500000000046 6.62270000000002
21.8684999999998 0.115899999999686 8.31739999999998
33.5414 1.09709999999973 -3.4418
36.0407999999999 -0.750500000000289 -6.02519999999999
73.7765999999999 7.9434999999999 -0.766699999999949
75.7491999999998 15.7888999999999 -0.135499999999939
ID=PHAtetUNKIVCAMNH89399*

LM3=54
5.36069999999749 -0.468500000000029 1.72379999999999
6.33469999999889 -2.27389999999946 2.56050000000002
26.7947999999993 -0.418699999999795 -1.92269999999999
29.4658999999999 1.34500000000051 -3.04700000000003
25.0791462721942 7.35117932297388 -1.50517069843004
30.6824257863028 3.91814908433453 -2.40013970130998
30.3314000064841 4.29703053566457 -3.4983553007187
38.6104999999999 0.164700000000401 7.56900000000004
39.3087093886155 12.4099719361243 -7.79983547534425
39.6554999999995 10.0340000000001 -2.36849999999994
43.6392999999986 9.96609999999996 -5.14389999999991
42.2587999999991 -0.160799999999887 -5.77329999999991
51.413699999999 9.95440000000007 -6.76279999999989
56.448999999999 5.85000000000012 -6.7865999999999
62.2574999999993 11.8342000000002 -6.61309999999988
64.941199999999 13.6056000000001 -6.77579999999984
66.0010999999991 20.4099000000002 -7.18179999999986
68.9969999999989 21.4057000000001 0.452600000000149
69.833899999999 12.3245000000001 -3.14749999999983
70.3971857929793 17.1290075184096 -5.95075424809414
5.78891318785704 2.78295828812014 1.89029132816341
6.36459061321204 4.70571083112365 1.9442157982637
21.887799999999 14.7121000000001 -1.86539999999997
24.0738999999992 14.4917000000002 -2.41329999999996
27.1236465662642 13.4952681745318 -2.66221063077117
26.8285282797767 13.2859906567445 -3.78004671410502
31.5170999999991 20.1125000000002 7.50950000000005
38.3902999999993 13.2880000000001 -2.26449999999994
41.8392999999992 16.1768000000001 -5.14689999999994
35.388099999999 23.4041000000001 -5.56229999999993
48.313999999999 20.3897 -6.32789999999991
50.0539999999989 26.6687 -6.0608999999999
58.106599999999 24.9826000000001 -6.22329999999987
61.272599999999 25.7922000000001 -6.30549999999987
64.3298999999988 29.2167000000001 -2.16889999999986
67.895361958873 25.8898409677034 -5.49596624196865
1.59979999999807 -0.247599999999829 8.96270000000002
2.40269999999799 2.82770000000012 8.56640000000002
18.8212999999988 10.9483000000002 10.0662
25.9389999999991 14.7849000000002 -1.47429999999996
26.661399999999 18.5037000000001 -3.64059999999996
27.4303999999993 7.40710000000032 13.2273
50.5678999999993 15.5237000000002 15.0207000000001

60.9782999999991 18.4341000000002 12.3338000000001
60.5591999999988 32.4455000000001 0.0175000000001349
67.609299999999 20.2456000000002 8.15370000000015
65.3884999999989 27.9460000000001 -0.364199999999846
2.09279999999812 -0.786299999999819 8.83690000000002
3.41069999999834 -2.90909999999972 7.71710000000001
21.4716999999992 -0.382599999999634 9.41380000000002
30.3681999999996 2.22620000000039 -1.05749999999998
32.1214999999994 -0.6509999999997 -3.70789999999996
68.6375999999991 6.5775000000002 -0.757699999999855
70.305499999999 14.0989000000001 -0.754399999999839
ID=PHAtetMALGABUSNM220402

LM3=54
5.25946306497819 0.143659086031928 1.58651373165782
7.18879999999895 -1.12450000000018 1.63640000000007
25.3158999999994 0.434400000000027 -3.16939999999998
28.8785999999996 2.49529999999998 -4.10169999999998
25.8515999999996 7.90020000000001 -3.38269999999992
32.4099999999994 4.94170000000008 -3.99259999999992
30.8628999999994 4.95079999999998 -5.01119999999994
37.3618999999996 0.733800000000001 6.95120000000006
39.7374000000003 12.2787000000002 -9.44689999999991
40.5494999999995 11.0772 -3.7405999999999
46.1651000000003 10.2252000000002 -6.8930999999999
42.9584999999998 -0.788999999999949 -6.55729999999992
52.2394000000001 9.57000000000009 -7.22399999999994
56.6987999999997 4.91340000000001 -7.18869999999993
63.5996000000002 10.9521000000002 -5.51469999999991
67.4332000000001 13.1676000000002 -4.5703999999999
67.3698000000004 19.4636000000002 -7.36479999999991
70.4363000000001 20.5635000000002 0.5325000000001
70.8334000000001 10.9059000000002 -1.93539999999989
72.0453000000001 15.8318000000002 -5.63659999999989
5.19681465900995 3.34152907122702 1.45246990747297
5.26579999999929 5.27980000000012 1.28900000000007
22.0211999999996 14.6813000000001 -3.68029999999993
25.1804999999995 14.1781000000001 -4.24599999999992
29.3030999999995 14.1036 -3.92899999999992
28.4345999999995 13.8776 -5.15209999999992
30.7341999999996 20.6833000000001 6.42420000000008
39.5301999999998 14.0619000000001 -4.13619999999991
44.1442000000002 17.1782000000002 -7.0060999999999
35.9335 24.9314000000001 -7.05949999999988
48.8802000000002 20.5986000000001 -7.31299999999989
50.4054000000002 27.2696000000001 -7.49949999999988
59.7256000000003 24.8056000000002 -7.1165999999999
63.4507000000004 25.6782000000002 -5.02959999999988
65.2495000000004 29.0242000000002 -2.50179999999989
69.0124000000004 24.8511000000002 -5.8973999999999
3.55779999999942 1.57560000000017 8.58470000000005
3.00759999999947 3.78680000000017 7.34070000000003
19.1835999999994 11.6841000000001 9.12410000000005
26.6182999999996 15.8350000000001 -2.61669999999991

27.6560999999996 19.1981000000001 -5.18139999999992
30.4593999999994 9.73000000000004 13.0006000000001
50.2854999999999 16.3551000000001 13.9934000000001
60.5484 18.0301000000001 12.2843000000001
62.2342000000003 32.6347000000002 -0.957099999999881
67.1804000000001 20.0014000000002 8.87320000000011
66.8735000000003 27.1225000000002 0.530100000000118
3.46379999999945 0.92230000000014 8.52480000000005
4.83369999999929 -1.35009999999992 7.83980000000004
22.5812999999992 1.57740000000002 9.55640000000005
30.6204999999996 1.88230000000005 -1.96189999999994
33.4702999999994 -0.701200000000021 -4.55639999999994
68.4674000000001 7.40680000000016 2.65760000000011
71.1724000000001 13.8872000000002 0.5966000000001
ID=PHAtetMALLIBUSNM481805

LM3=54
5.71639999999661 -0.221900000000823 1.51220000000006
6.51369999999916 -1.64240000000016 1.42839999999998
26.4433999999998 0.643099999999867 -3.22420000000004
28.3333999999998 2.29189999999998 -4.17150000000001
27.1675999999996 8.27729999999987 -4.23520000000001
31.3618999999994 4.95839999999979 -3.84569999999995
31.0903999999992 5.17649999999987 -5.08879999999998
37.1040999999993 0.452099999999883 6.74980000000007
41.1422999999994 12.5527999999999 -9.72229999999993
40.3620999999993 10.3392999999998 -3.55129999999993
45.4272999999998 10.1652 -6.34539999999993
44.0048999999999 0.289699999999972 -7.03929999999995
52.9066999999999 10.2881 -6.59639999999993
57.6581999999999 6.07590000000007 -6.96259999999993
64.2567999999995 12.5179 -6.16679999999991
68.1911999999998 14.2682000000002 -4.67359999999991
68.8825999999994 20.5852000000001 -6.0950999999999
72.3340999999994 22.5142000000001 1.13920000000011
72.2019999999996 12.9872000000001 -0.196899999999903
73.1252999999995 17.9571000000001 -4.6183999999999
4.55169999999785 3.0111999999995 1.17359999999996
5.11189999999825 4.68959999999962 0.924199999999932
22.1525999999992 13.6722999999998 -3.6015
25.2669999999993 13.3125999999998 -4.25829999999999
28.2285999999993 12.8834999999998 -3.63649999999996
27.1769999999993 12.8315999999998 -5.25959999999997
31.7217999999991 20.1502999999998 5.89730000000003
39.4157999999994 13.6795999999998 -4.18749999999993
43.3287999999996 16.8669999999999 -6.58479999999993
36.1078999999993 23.9750999999999 -7.75359999999994
49.4635999999995 20.9961 -6.6192999999999
51.3851999999994 27.6165 -7.35989999999989
59.7155999999994 25.5547000000001 -6.39059999999989
63.9998999999994 26.4979000000001 -4.5297999999999
65.8374999999994 30.0726000000001 -1.4389999999999
69.5320999999994 25.1697000000001 -5.5169999999999
3.03099999999864 0.974399999999734 6.7116999999999

2.84419999999857 3.73999999999973 5.94889999999991
18.227499999999 11.1498999999998 7.39969999999998
26.9858999999993 14.7603999999998 -3.40749999999999
27.3640999999992 17.5693999999998 -6.06039999999998
28.5292999999992 8.41939999999982 11.637
48.8944999999993 15.8338 13.0901000000001
59.6781999999993 18.6208 12.0400000000001
62.5260999999993 33.4303000000001 -0.00219999999989917
67.3932999999993 21.2868000000001 8.7244000000001
67.5795999999993 28.2724000000001 0.60280000000011
3.2077999999987 0.587499999999755 7.06359999999989
5.24759999999875 -1.57980000000024 6.87559999999991
20.8755999999993 0.696399999999854 7.35829999999996
30.6431999999997 3.33579999999985 -2.50749999999999
32.3902999999996 0.740499999999847 -5.49199999999997
70.7301999999995 7.8774000000001 0.398700000000091
71.6294999999995 15.0944000000001 1.6269000000001
ID=PHAtetMALLIBUSNM481806

LM3=54
2.70414932857828 -1.85901251206195 -7.95506882003904
4.22622036482108 -2.71572498090902 -8.42962071321555
26.5617999999997 -1.06119999999999 -10.7662000000001
29.3509000000007 1.75640000000005 -11.2717999999998
29.1205000000001 8.54190000000003 -10.5411
33.4768999999991 5.16899999999983 -9.36169999999998
31.7387999999995 4.70360000000008 -11.0027
41.3314000000008 -1.24759999999978 3.62560000000011
43.1345 12.3073999999999 -12.9645999999999
45.5161999999996 11.3985999999999 -6.36289999999987
49.9589000000006 9.67550000000011 -8.43859999999985
46.6267 -2.44660000000003 -12.2326999999999
56.5163000000001 10.2339 -8.43269999999978
59.8597000000002 5.80860000000004 -7.36379999999978
67.4259000000002 11.9109 -6.35189999999974
71.2418000000006 13.0286 -5.4002999999997
72.3326000000008 21.1215 -7.47899999999969
76.9742000000009 22.4043 1.71580000000034
76.2243000000006 10.9401 -0.798999999999669
76.6766000000007 17.1992 -6.13109999999967
1.16348020644626 2.65959810990578 -7.98311855849592
1.81833739485435 4.46705021291285 -8.53091834423915
22.3982000000004 15.3175 -11.2209999999999
26.3075000000004 15.6852 -11.5751999999999
31.7853000000002 14.0777 -9.13469999999995
29.9464000000002 13.4202 -10.964
33.5184000000006 24.1431 3.33740000000016
44.7537 15.2115 -5.87719999999986
47.9015000000004 18.0688 -8.71899999999985
38.2512000000006 26.5779 -12.1415999999999
54.2082000000004 20.9078999999999 -8.2407999999998
53.7329000000006 27.5703 -7.77969999999979
63.2614000000006 26.4311 -6.55399999999974
66.7683000000008 27.3086 -5.2893999999997

71.041000000001 32.2035 -1.33379999999967
73.7374000000009 26.5276 -6.66689999999965
-0.707499999999405 0.686100000000011 -0.133499999999833
-0.19029999999934 4.6456 -1.18409999999983
20.4140000000006 12.0896 5.07720000000011
30.5112000000004 17.5669 -9.12959999999994
30.6833000000005 21.2962 -12.4030999999999
29.7117000000005 8.71240000000006 8.4926000000001
51.965500000001 16.1011000000001 13.7409000000002
61.948900000001 19.1220000000001 14.3664000000003
63.204400000001 35.1071 2.06690000000031
71.7591000000011 21.1815000000001 11.6598000000003
72.755400000001 30.0378 1.66210000000033
-0.318199999999418 -0.751899999999994 -0.0605999999998498
3.48770000000048 -4.24130000000001 -0.818399999999849
24.8673000000003 1.62650000000001 5.72660000000009
36.1062999999991 0.564099999999861 -8.47859999999998
37.1387999999992 -2.93290000000009 -11.6224
73.2245000000006 5.24860000000002 1.9398000000003
77.9798000000008 14.9135000000001 1.39530000000034
ID=PHAtriMALNIGBMNH102858

LM3=54
5.53450000000011 0.683799999999846 1.39149999999988
6.86460000000008 -0.317400000000116 1.37779999999995
26.0085999999996 0.0936999999996797 -3.55090000000005
27.1232999999998 1.79769999999969 -4.70930000000006
28.9336999999999 8.38839999999968 -5.79850000000006
32.1777999999998 5.02809999999968 -5.66570000000006
30.0428999999997 5.46869999999966 -6.61890000000005
41.6210999999994 0.378399999999555 5.36169999999998
38.8017999999997 10.7490999999996 -11.3730000000001
43.9526999999994 10.5957999999995 -6.10470000000003
46.6085999999993 9.6325999999995 -8.50850000000004
44.9362999999993 -0.790300000000515 -8.59610000000003
52.596199999999 9.76199999999942 -10.2141
55.3631999999989 4.86639999999937 -9.8306
63.1706999999986 10.2699999999993 -10.9474
67.8015999999985 11.4142999999993 -9.25719999999998
67.4443999999988 19.0816999999993 -11.6495
72.5646999999984 20.0901999999992 -4.5505
71.3070999999985 9.78609999999925 -8.08919999999998
70.0627999999985 15.8517999999993 -12.1227
4.23060000000028 3.50749999999993 0.566799999999965
5.58620000000019 4.29549999999992 0.662099999999955
22.6905 13.7556999999998 -3.87400000000006
24.0658 13.3647999999998 -4.28890000000007
28.8885927992998 12.3235951648601 -4.96777521551698
27.7528361991166 11.2075541372203 -6.0837917659058
38.5086999999997 23.1806999999997 5.16579999999996
43.4920999999994 13.7136999999995 -6.22600000000004
46.2131999999994 15.8584999999995 -7.77490000000004
38.7053999999995 23.5316999999996 -8.80310000000006
50.8864999999992 18.7574999999995 -10.0804

51.6206999999993 25.1698999999995 -9.52900000000004
59.438999999999 24.3917999999994 -10.9631000000001
63.7838999999989 25.7647999999994 -8.93620000000003
66.8245999999987 29.4044999999993 -7.63600000000002
67.5409999999987 22.3118999999993 -12.1779
5.32270000000009 2.07899999999992 7.1778
6.12360000000019 4.34869999999992 7.31519999999998
21.4161999999999 11.1045999999998 7.59639999999998
27.5686999999998 16.8155999999997 -3.33550000000006
26.209775641958 17.1618622755561 -5.35989612748657
32.5268999999997 8.89269999999969 10.5696
54.7120999999991 14.8206999999995 11.7802
61.2029999999986 16.7170999999993 10.3769
61.3287999999988 32.4163999999994 -2.73360000000003
70.4444999999985 19.7883999999993 5.88330000000002
68.5830999999986 27.7215999999993 -4.82730000000001
4.8607000000002 1.49019999999993 7.47569999999999
7.13310000000016 -0.680000000000094 7.53669999999999
24.5727999999998 2.15869999999974 8.49739999999999
33.2592999999996 -0.504900000000362 -3.12740000000003
32.3116999999995 -0.954800000000398 -4.52040000000003
70.2678999999986 3.8657999999993 -3.46639999999998
73.9482999999985 12.8307999999993 -5.57089999999999
ID=PHAtriUNKCONBMNH121231

LM3=54
5.58279999999811 -0.00240000000017903 0.503100000000065
6.02949999999943 -0.704399999999646 0.410599999999913
24.3585999999989 0.0739999999999484 -3.95380000000001
26.516599999999 2.11719999999984 -4.53990000000004
25.3073999999996 7.62409999999997 -4.16390000000004
31.0540999999998 5.32530000000013 -4.56530000000013
28.9087000000005 5.45900000000026 -5.91220000000015
38.7808999999994 0.297200000000097 4.79159999999991
35.4899999999998 10.8653000000001 -9.04770000000007
40.2968999999996 9.45630000000005 -4.57450000000008
43.2602999999995 7.84770000000002 -7.18050000000009
41.3221999999995 -1.56759999999989 -7.54400000000008
48.3166999999999 8.81310000000021 -8.55150000000016
52.9257000000001 4.1580000000003 -8.06290000000018
59.5290000000002 10.2260000000004 -8.4009000000002
63.7051000000005 10.8739000000004 -6.95670000000026
63.3106000000005 17.8664000000004 -9.29950000000023
68.4453000000007 19.6207000000005 -2.90160000000026
67.4490000000007 9.87250000000054 -6.08790000000028
65.7964000000005 14.4244000000005 -9.79250000000027
4.74359999999899 3.11560000000003 0.662399999999991
5.23789999999907 4.38690000000003 0.397499999999957
21.4400999999996 13.6477 -4.14070000000005
22.8010999999996 13.2275999999999 -5.09330000000005
29.1548999999996 13.5073999999999 -5.18220000000005
27.3761999999996 12.4341999999999 -5.79510000000005
32.5605999999998 21.5075 5.59869999999989
40.0114999999996 13.9689 -4.1608000000001

42.0647999999997 15.8053000000001 -7.66980000000008
35.0906 23.7269000000001 -7.8718000000001
46.8927 18.3442000000002 -9.03110000000013
47.8670000000001 24.8402000000002 -8.63740000000015
56.6251000000003 23.8300000000003 -8.52690000000021
60.0656000000006 24.0975000000004 -7.23080000000025
64.4130000000007 28.0037000000005 -5.64610000000027
64.4146000000007 22.0354000000005 -10.0867000000003
3.30499999999902 1.83710000000005 6.82519999999991
3.84829999999906 5.39330000000003 6.03979999999992
19.1689999999992 11.7010999999999 7.94139999999995
25.6218999999997 15.9872999999999 -1.60560000000007
24.7698999999997 17.4316999999999 -5.56680000000008
29.5445999999994 9.68939999999999 10.6761999999999
48.9831999999999 15.6504000000002 12.9353999999998
56.2981000000001 17.3668000000003 11.4728999999998
55.1799000000005 30.9159000000004 -2.94850000000027
64.0542000000005 20.0802000000005 6.78279999999972
63.5630000000007 27.5126000000005 -4.12190000000027
5.06459999999903 1.64910000000007 8.12169999999993
6.290199999999 -1.02279999999989 7.06579999999994
23.0623999999991 3.31609999999999 9.17509999999996
31.1301999999995 0.658000000000042 -1.20140000000005
30.3808999999994 -1.36999999999996 -4.85590000000004
64.4110000000004 4.54580000000049 -2.45330000000027
68.4338000000007 11.8888000000005 -4.01650000000027
ID=PHAtriUNKCONBMNH121233

LM3=54
6.5772 0.0333 -0.9833
7.7554 -0.7821 -0.9273
26.1306 0.562 -6.3006
29.346 2.5534 -6.6759
27.6552 8.4599 -5.7258
33.2364 5.7565 -5.669
31.0164 5.4559 -7.3395
43.3439 0.4867 4.5686
39.9689 11.5196 -11.3992
44.3039 10.7734 -6.4963
47.4744 8.855 -9.1965
44.5055 -2.12 -9.1569
52.617 8.9613 -10.4167
57.0432 4.9413 -10.0103
63.7316 12.031 -11.32
69.2856 12.4491 -9.5712
67.9465 20.639 -12.1414
74.705 22.3992 -4.8064
74.452 10.7037 -8.2343
72.3263 16.091 -11.7537
5.7161 3.7924 -0.5339
6.1121 5.0615 -0.4162
22.2594 14.0508 -5.7201
25.0397 14.5923 -6.1894
30.4394 13.7852 -5.4423

28.817 13.276 -6.7368
34.803 23.0848 5.9832
43.1997 15.393 -6.265
44.6365 17.4749 -8.0643
37.2383 25.0575 -10.1497
49.9042 19.7571 -10.003
52.1634 26.5839 -9.9665
60.3623 24.5044 -11.0358
64.8881 27.1648 -9.7109
68.3246 31.8683 -8.2765
69.5871 25.7695 -11.6573
6.2289 2.3841 6.3396
5.3476 5.4708 4.5852
22.7784 14.8974 5.5314
29.2718 15.6846 -4.9154
27.3093 17.8868 -7.4936
36.105 10.5764 11.1325
54.6746 16.0891 11.8419
63.3495 18.8884 9.7689
61.4336 34.0281 -1.939
73.1749 21.6189 5.1524
69.9528 30.2346 -5.4849
6.4184 1.5183 6.1925
7.7527 -1.663 4.9939
27.843 -1.0314 4.3248
34.0234 1.1716 -5.458
33.7965 -1.2189 -7.4362
70.7806 4.9361 -2.6978
75.8398 13.0963 -5.0304
ID=PHAtriUNKNIGBMNH999115

LM3=54
4.9315 -0.773 0.6847
5.2255 -0.9831 0.547
21.2807 0.0471 -3.7997
23.4851 1.4516 -4.7042
24.4253 6.6388 -4.8285
28.2864 5.1139 -5.0773
27.2295 5.186 -5.9609
36.6469 0.2425 4.6277
35.1289 10.4955 -10.3428
38.8923 10.56 -6.3907
41.4971 8.7525 -9.0859
38.5553 -1.1779 -9.4433
46.6362 9.5142 -10.0295
51.3127 5.7534 -10.241
56.6865 11.2176 -10.9497
60.3992 12.9012 -10.7346
60.0453 19.5383 -12.7967
65.1356 21.4211 -4.5123
65.3591 11.2293 -9.6463
63.6311 16.2098 -12.3036
4.0687 2.3353 0.8684
4.275 4.0023 0.6084

18.1152 11.5725 -3.4182
20.3923 12.2109 -4.4175
26.5564 11.7722 -4.3791
25.2291 11.6601 -5.6329
31.2152 21.212 4.6699
38.3195 13.5933 -6.5952
38.9431 16.2554 -8.7008
30.9325 22.4439 -9.2527
44.1064 18.1694 -9.9687
43.8764 24.6258 -10.0645
52.5959 24.4644 -10.6924
56.2237 25.4459 -9.9957
60.3314 29.8806 -8.5407
60.7638 24.1859 -12.3265
5.2821 1.1796 7.0636
6.2495 4.396 6.7882
20.4894 8.7916 8.589
24.8038 12.9573 -2.1869
22.1819 14.917 -5.1328
29.3955 8.2136 9.8367
46.9752 14.3217 11.5824
54.1156 17.2327 9.3857
53.6712 31.794 -4.0964
64.2479 20.943 4.7345
62.7936 26.975 -5.8956
4.8984 0.9764 6.931
7.2939 -1.6836 6.2473
22.9715 2.8892 8.4177
29.1032 1.3272 -2.4793
27.7026 -1.1245 -5.7701
63.3264 5.122 -4.0344
67.0882 13.5232 -6.2382
ID=PHAtriFEMCONBMNH411189

LM3=54
6.58550000000291 1.16480000000038 1.71449999999978
6.81070000000292 -0.620399999999461 2.13959999999981
26.2178000000017 -0.122999999999462 -2.46150000000014
28.7870000000017 3.16360000000057 -3.48430000000016
29.0110000000014 8.41470000000056 -3.56510000000008
32.2124000000014 6.03120000000053 -3.70860000000009
31.9328000000014 6.50290000000053 -4.57370000000007
43.0273000000011 0.489400000000224 6.02919999999997
38.925400000001 11.9326000000004 -7.76489999999998
44.2207000000007 11.0511000000002 -4.84629999999993
45.9409000000006 10.0756000000002 -7.31009999999989
43.1651000000011 -2.04189999999977 -8.11179999999998
51.3162000000006 9.7176 -8.97739999999986
56.6665000000007 4.53439999999995 -9.08589999999984
62.8434000000006 10.8249999999997 -10.2592999999998
66.4651000000007 12.3457999999997 -9.81239999999977
66.0143000000005 19.2684999999996 -11.0807999999997
72.5092000000006 20.4808999999995 -2.48709999999979
71.4200000000009 9.6220999999996 -7.44769999999979

70.1123000000008 14.4547999999996 -12.0039999999997
5.69970000000304 3.21500000000054 1.63259999999979
6.006000000003 5.11460000000057 1.81149999999978
23.3538000000019 14.2677000000006 -2.27350000000012
25.6546000000019 13.7222000000007 -3.87330000000012
30.8041000000014 13.4217000000006 -3.39440000000009
30.3845000000015 12.4229000000006 -5.09490000000008
37.2107000000013 24.0880000000003 6.71049999999997
43.3125000000006 15.0093000000002 -4.87589999999992
44.2795000000007 16.5314000000002 -7.54339999999989
36.1316000000013 24.2741000000003 -8.09649999999998
49.9296000000006 19.4555 -8.83239999999985
50.1668000000007 25.9408 -9.01019999999984
59.2793000000006 24.0099999999997 -10.2227999999998
62.9519000000006 24.6151999999996 -9.74359999999977
65.7204000000007 29.6718999999996 -7.58239999999978
67.3882000000007 23.2170999999996 -11.9222999999998
5.92230000000293 1.81350000000043 7.80149999999981
4.799400000003 4.33950000000052 6.95739999999977
22.6114000000021 11.8610000000005 9.49359999999982
28.1176000000015 16.5639000000006 -2.77850000000007
26.2316000000017 17.6835000000006 -4.21770000000009
32.1953000000015 9.56400000000033 11.8229999999999
53.4904000000009 16.6345999999999 13.1709000000001
61.1693000000008 18.6195999999998 11.8043000000001
60.0105000000007 33.3402999999997 -2.05069999999978
68.9909000000006 21.1278999999995 7.63150000000021
67.9271000000006 28.8733999999996 -4.78399999999976
5.41210000000302 1.31850000000045 7.34359999999977
6.91680000000289 -1.19679999999951 6.73939999999977
26.0998000000017 2.60190000000039 9.38319999999987
33.0550000000014 1.08990000000049 -3.8667000000001
32.1568000000015 -0.802299999999502 -5.3756000000001
69.3678000000009 4.79459999999966 -3.20339999999982
73.2726000000008 14.4317999999995 -5.03649999999977
ID=PHAtriMALUGABMNH346292

LM3=54
6.69410000000089 0.849200000000363 0.213799999999746
7.83060000000108 -0.816299999999512 -0.116100000000267
25.9345000000008 -0.142399999999807 -5.8154000000001
27.9455000000007 2.08220000000017 -6.47340000000008
27.5203000000007 7.88710000000015 -7.52660000000006
32.5711000000007 6.03420000000014 -7.05300000000006
31.2226000000006 5.32950000000011 -7.65440000000002
40.3271000000005 1.08230000000017 3.30709999999994
39.3624000000003 12.071 -12.7746
42.6646000000003 11.0551000000001 -7.1029
45.2366000000002 9.34070000000008 -9.85069999999997
41.9487000000004 -1.33089999999989 -10.8797
50.7568000000003 9.01040000000015 -11.7549
53.7928000000003 3.93100000000017 -11.6476
61.4152000000001 10.5513000000002 -13.3863
64.6939000000001 11.9034000000003 -13.2423

63.5857000000001 19.0718000000003 -14.977
71.3300000000003 20.9747000000004 -9.25760000000001
69.9569000000002 10.3587000000003 -11.8246
67.2626000000004 16.0675000000004 -15.7363
5.54670000000112 3.56610000000047 -0.0732000000002722
6.57390000000102 5.09990000000045 -1.08940000000025
21.7243000000008 14.8143000000002 -5.62420000000012
24.3813000000008 14.9493000000002 -6.81090000000012
30.5231000000007 13.8223000000001 -6.54250000000007
29.0415000000007 13.7190000000001 -7.78370000000006
35.8793000000007 22.9015000000002 3.14739999999988
42.4099000000003 14.3391000000001 -7.16339999999999
43.4430000000003 17.3396000000001 -9.89799999999999
34.9156870776861 24.9905998912667 -10.7799887150954
48.0364000000002 19.5138000000001 -11.8107
48.3052000000004 25.9763000000001 -12.03
57.9327000000004 25.0969000000003 -12.8905
60.7773000000002 24.6506000000003 -13.0987
64.7946000000004 29.8387000000003 -11.8771
65.4123000000003 23.3305000000003 -15.751
5.45880000000124 2.28670000000056 7.32599999999969
4.86630000000123 5.58350000000056 5.92139999999969
21.4548000000011 13.6211000000004 7.10099999999978
28.1884000000007 16.5310000000001 -4.10050000000009
24.8144000000007 17.6374000000001 -7.33640000000009
35.2153000000008 11.3025000000003 10.3294999999998
50.4822000000006 15.4943000000003 11.1345999999999
58.0655000000006 17.7718000000004 8.69209999999991
59.2032000000006 32.3404000000004 -4.70830000000006
69.8044000000005 21.1129000000004 0.845599999999937
66.0967000000004 27.7206000000004 -8.96400000000003
5.68910000000125 1.74600000000054 7.22559999999967
7.17910000000119 -1.46159999999946 6.32819999999969
25.3009000000009 2.6260000000003 7.55649999999981
32.8684000000007 1.09430000000015 -4.02550000000005
31.1684000000006 -1.54499999999986 -7.13950000000005
67.3051000000002 5.0072000000003 -4.08860000000001
70.8095000000003 12.6196000000004 -9.00350000000002
ID=PHAtriFEMGHABMNH022157

LM3=54
5.24109999999821 -0.539100000000032 -0.0385999999999205
6.24709999999941 -1.69409999999951 -0.15780000000003
24.257599999999 -0.0968999999998706 -5.5517
27.0989999999992 1.79340000000002 -6.53900000000003
26.3020999999998 8.6134000000002 -5.31680000000006
30.2836 5.46900000000031 -5.75870000000016
29.7123000000006 5.29520000000046 -6.83240000000019
38.7270999999994 -0.0129999999998374 4.68799999999991
36.3329999999999 11.9010000000002 -9.8704000000001
40.7153999999997 10.7970000000001 -5.03520000000012
43.7158999999996 9.52970000000005 -8.4026000000001
41.9149999999995 -0.508199999999857 -10.5532000000001
48.5787999999999 9.76300000000018 -9.45060000000015

52.2361000000001 5.34760000000023 -9.92060000000017
58.7395000000001 11.0322000000002 -9.88350000000019
63.0555000000004 12.1245000000002 -8.83850000000022
61.8074000000003 19.8748000000002 -10.6700000000002
68.1536000000004 21.5555000000003 -3.55110000000023
67.0984000000005 10.8231000000003 -6.98280000000024
65.2926000000004 16.6938000000003 -10.6156000000002
3.40239999999908 2.97000000000026 0.485700000000029
5.12099999999915 4.95520000000026 0.199700000000012
20.2570999999998 14.3979000000003 -4.66700000000006
23.0986999999998 14.8939000000002 -5.84190000000007
27.9022999999998 13.3618000000002 -5.37830000000009
27.4393999999998 13.0714000000002 -6.16750000000008
33.5119999999998 22.2432000000002 5.51359999999985
39.8596999999997 14.9905000000001 -4.98610000000013
41.7063999999998 17.1257000000001 -7.81360000000013
34.4878000000001 23.4362000000002 -9.39470000000015
45.8146 19.1300000000002 -9.26410000000017
46.2617000000001 25.6617000000002 -8.92350000000018
54.6869000000003 24.9928000000002 -9.52580000000021
58.1618000000004 25.8003000000002 -8.64410000000024
61.5618000000005 30.2001000000003 -6.33490000000026
62.1172000000005 23.4624000000003 -10.5878000000003
4.04149999999906 1.63180000000028 6.8564
2.89049999999912 4.68320000000026 5.4559
20.0575999999994 12.5864000000002 7.46299999999994
26.8252999999999 15.9922000000002 -2.8753000000001
24.9600999999999 18.3673000000002 -6.3597000000001
31.5403999999995 9.93750000000015 10.1035999999999
49.6392999999999 15.7891000000002 11.8700999999998
56.6303 17.7877000000002 9.44399999999978
55.6583000000004 33.1367000000003 -2.50390000000024
66.0433000000003 20.4901000000003 4.78009999999975
62.6755000000004 28.0936000000003 -4.38450000000025
4.39489999999906 1.2938000000003 7.0603
5.15789999999904 -1.72329999999968 5.42710000000001
24.0228999999992 1.68570000000016 7.42109999999997
32.3789999999997 2.1530000000002 -3.31150000000007
31.8067999999995 -0.939799999999817 -6.28170000000005
65.4248000000003 5.50620000000028 -1.69120000000022
67.7755000000005 13.1888000000003 -4.77530000000024
ID=PHAtriFEMGHABMNH46500*

LM3=54
6.0989 0.7268 0.7263
7.4317 -0.3438 0.3935
27.4372 -0.4337 -6.6586
29.1601 1.7909 -7.4414
29.6236 8.1353 -7.5597
33.6182 5.0954 -7.7553
32.2225 5.0123 -9.2701
44.7909 0.6621 2.8521
40.1833 11.168 -13.7495
42.7264 10.1122 -8.3014

46.6142 9.7627 -8.6636
44.8474 -1.6142 -12.3895
53.2162 9.5971 -12.7985
55.0306 3.6714 -12.4822
63.8518 12.0102 -13.8998
68.5118 11.3455 -13.3231
70.1515 19.2445 -15.3492
76.7969 20.8061 -7.212
74.8811 10.4014 -10.7827
74.3621 13.8748 -15.0565
5.5372 3.6604 0.6727
6.4134 5.4703 0.472
22.5929 14.8156 -5.8444
25.465 14.5083 -7.6367
31.7187 14.4837 -7.8569
30.4372 13.5908 -9.2164
38.706 23.7403 2.8581
42.4281 14.1833 -7.9205
47.3237 15.5455 -8.7476
37.699 25.1257 -11.7795
51.1459 20.2222 -12.7608
48.9084 25.5997 -13.01
61.6821 24.1905 -14.3339
65.1889 26.0365 -13.7897
70.0357 30.6318 -11.2893
71.9459 25.8612 -15.6098
6.5156 2.6991 8.7153
4.9745 6.0129 7.4901
23.7418 13.353 8.6065
29.4157 17.4085 -4.6057
26.9586 17.114 -7.3023
33.3401 10.0909 9.602
55.2834 16.2893 10.8135
63.6917 18.8223 9.2606
62.7968 33.5631 -3.9888
72.5722 20.4926 4.9774
74.0406 26.4396 -8.1275
6.6085 1.4029 8.6968
7.1417 -2.2464 6.9002
26.5753 2.6378 7.6398
34.5112 -0.0076 -5.2739
32.4295 -1.4731 -7.6783
70.9266 3.671 -4.4793
77.2981 14.9707 -7.7047
ID=PHAtriMALCAMBMNH481320

LM3=54
5.82189999999915 -0.202000000000229 0.272300000000024
7.19630000000059 -1.25589999999958 -0.129799999999993
27.1681999999997 -0.468699999999974 -4.28379999999997
29.4199999999999 1.53559999999993 -5.05200000000001
28.7890000000005 8.14760000000007 -5.18229999999999
34.5075000000007 5.67260000000018 -4.86470000000011
32.3462000000014 4.80300000000034 -6.21020000000008

42.844 0.0386000000000927 6.01489999999994
39.4498000000005 10.6651000000001 -9.99560000000004
44.8302000000001 10.5143 -4.42490000000008
47.8528999999999 8.59060000000002 -7.55300000000005
44.7773 -1.4359999999999 -8.13710000000006
54.0649000000001 8.88050000000017 -8.38720000000011
56.8352000000003 4.52340000000025 -7.83020000000012
64.4476000000001 10.4269000000003 -8.25670000000017
68.5390000000003 11.2651000000004 -7.06490000000018
68.7774000000003 19.4822000000004 -9.03990000000018
73.3826000000004 21.0522000000004 0.00389999999979596
73.4413000000003 9.71040000000044 -4.21230000000021
72.2231000000003 15.0776000000004 -8.61380000000021
4.8801000000002 3.34130000000009 0.277400000000045
5.60730000000027 4.2982000000001 -0.261399999999969
23.4408000000007 14.4521000000001 -4.8425
25.4707000000006 14.5223000000001 -5.3541
32.3640000000005 13.654 -5.02600000000002
30.6577000000006 13.5357 -6.39920000000001
35.6630000000005 22.3910000000001 7.70719999999992
44.6173000000002 14.6009000000001 -4.73290000000008
46.0152000000002 16.7373000000001 -7.39640000000008
37.9831000000006 23.6104000000001 -7.99360000000007
51.3706000000003 20.0368000000001 -8.38050000000011
51.0414000000004 25.8361000000002 -8.13040000000012
60.5089000000003 24.8411000000003 -8.03190000000017
64.3040000000004 26.0485000000003 -7.13980000000019
68.1145000000005 31.0097000000004 -4.0288000000002
70.1958000000005 24.9575000000004 -8.36830000000022
6.47700000000024 2.73180000000014 7.4994
5.17890000000028 5.6382000000001 5.98140000000001
22.2443000000003 12.5784 8.54189999999999
28.9965000000007 16.3619 -1.20080000000003
26.9423000000007 17.1193000000001 -5.72800000000002
32.4184000000002 9.80360000000005 11.6216999999999
52.5561000000002 15.3410000000002 14.9804999999999
59.7675000000002 17.7052000000003 14.0380999999998
60.3727000000005 33.3415000000004 -0.129500000000182
70.7600000000004 20.5334000000004 9.09049999999978
70.2271000000004 27.6408000000004 -0.954100000000205
7.24260000000021 1.89800000000013 7.7261
7.82490000000019 -1.77039999999983 6.13180000000003
25.9715 2.56070000000005 8.7654
35.0294000000004 2.05840000000011 -1.63670000000003
33.2398000000002 -0.731399999999904 -5.12720000000001
70.0225000000002 4.14370000000039 0.201299999999818
74.6857000000004 14.3622000000005 -0.688000000000199
ID=PHAtriFEMCAMBMNH481325

LM3=54
-1.07959999999964 -0.973299999999933 -6.16380000000019
0.0777999999990667 -1.79650000000032 -6.27300000000004
17.5100000000001 -0.344600000000027 -8.33090000000001
18.7507000000004 2.02360000000003 -8.04250000000018

19.7070000000001 7.05919999999998 -7.89739999999998
21.9568 4.26240000000004 -7.64890000000002
21.5785000000003 4.38720000000005 -8.8192
29.9383999999995 -1.03670000000009 4.08709999999999
29.4316999999996 10.5454999999999 -10.4458
32.6515000000002 9.76190000000012 -5.74010000000011
35.0314999999998 8.11410000000004 -8.14810000000007
33.4092999999998 -0.678999999999969 -9.61070000000007
40.4666999999999 8.95710000000015 -8.64500000000011
44.0823 4.8137000000002 -7.56940000000013
50.8205999999996 10.6921000000002 -7.22670000000014
55.0609999999997 10.7063000000002 -5.67770000000017
53.6362999999994 19.2972000000002 -6.98770000000017
58.3201999999994 20.9445000000002 2.5194999999998
59.1870999999995 9.17600000000022 -3.71800000000019
57.7043999999995 15.3215000000002 -6.31430000000019
-1.15849999999965 0.605200000000112 -6.95320000000018
-0.646099999999762 1.73090000000008 -7.06800000000016
13.9899 11.2294999999999 -8.3886
15.7003000000001 11.215 -8.65800000000001
19.9625 12.0674 -7.57329999999999
19.8102000000001 10.9034 -8.93059999999999
21.8851 20.8340000000001 4.08419999999994
31.9688 13.6188 -5.66820000000007
33.4667999999997 15.0908 -8.05820000000005
25.8153999999998 20.2692 -9.07780000000004
37.6696999999997 17.6375000000001 -8.13470000000009
38.3473999999996 23.7594000000001 -8.11780000000009
46.4123999999995 23.7082000000001 -7.89670000000013
50.0630999999995 25.6441000000002 -6.09520000000016
52.5463999999994 30.2796000000002 -3.41100000000018
54.7664999999994 23.1842000000002 -6.58790000000019
-2.15554825412902 -0.587398188375658 -0.743367755460821
-2.10395653827794 1.93363955176037 -1.48208203857243
9.9608999999999 9.93359999999998 2.88139999999996
16.6512 13.9186999999999 -4.8723
15.8277 14.2478 -7.54230000000002
16.6314999999998 6.6602 6.80729999999997
40.5420999999997 14.9897000000001 13.5994999999999
43.1461999999996 16.4960000000002 13.1020999999998
44.2152999999995 31.6750000000002 -0.107200000000171
55.1181999999995 20.8889000000002 9.45649999999981
54.6504999999995 29.1433000000002 -0.42930000000019
-2.07615708857573 -1.13892832138069 -0.813145683665198
-0.587076749162304 -3.24289902815339 -1.34696129993647
14.2469999999997 -0.812500000000081 2.81879999999997
22.1823000000001 -0.292999999999984 -4.98450000000003
22.0421000000001 -1.5597 -7.29330000000003
54.9409999999996 4.85650000000019 0.312299999999814
60.5710999999995 13.8679000000002 0.0862999999998069
ID=PHAtriFEMCAMBMNH48133*

LM3=54
5.68920000000053 -0.0351999999998273 0.50220000000003

6.56720000000031 -1.48209999999991 0.406899999999931
23.0559000000003 -0.502799999999909 -4.90900000000002
24.7920000000002 1.20750000000008 -5.41020000000002
25.7556000000003 6.64040000000013 -6.23760000000005
29.3029000000001 4.32220000000008 -5.29980000000001
28.9396000000002 4.59570000000012 -6.64080000000002
37.9867999999996 -1.06910000000004 4.73940000000005
37.8892999999998 11.1095 -10.5237
39.7045999999997 9.35970000000002 -6.09259999999991
43.7886999999995 9.40649999999998 -7.57789999999989
41.3224999999995 -3.35259999999998 -8.31279999999988
48.0795999999991 8.26869999999991 -9.74169999999981
51.9715999999988 4.46539999999988 -9.40339999999978
59.2278999999982 10.4397999999997 -10.2604999999997
63.013999999998 11.7339999999997 -9.39489999999968
61.5856088389883 18.0978267762455 -11.5259317178647
67.2693999999978 20.4984999999996 -3.40179999999966
67.1093999999977 9.62909999999965 -6.50489999999964
65.6483999999977 15.6099999999996 -10.2655999999996
4.33810000000047 3.32350000000008 0.32299999999995
4.41660000000038 4.5307000000001 -0.0255000000000661
19.1309000000002 12.0670000000001 -5.12770000000004
21.6991000000002 12.8682000000001 -5.54000000000005
27.0681000000002 12.2800000000001 -5.43180000000003
26.8964000000002 12.1224000000001 -6.52960000000003
32.4654999999996 22.4583999999999 5.97660000000003
38.7174999999996 12.6453 -5.59789999999992
41.5360999999995 15.1526 -8.78669999999988
32.6461999999995 23.2384999999999 -9.90309999999991
44.6568999999991 17.4264999999999 -10.0104999999998
46.413199999999 24.3972999999999 -9.4122999999998
54.9148999999985 24.1534999999998 -10.2475999999997
58.5128999999983 24.7992999999997 -9.4635999999997
61.7959999999981 29.2662999999996 -6.78309999999967
63.9465999999979 22.0276999999996 -10.8322999999997
5.70850000000022 1.45670000000005 7.59099999999995
4.79020000000026 5.26250000000005 6.13109999999995
20.4817 12.489 8.13599999999994
26.5587000000001 14.2386000000001 -2.65480000000004
24.9222000000001 16.9414000000001 -6.77160000000003
30.5379999999997 8.83479999999992 10.6683
48.777599999999 14.6512999999998 11.5741000000001
56.8573999999985 16.8332999999997 9.3430000000002
55.3998999999982 32.0270999999996 -3.55559999999972
64.9562999999979 18.7956999999996 4.7491000000003
63.449799999998 28.1545999999996 -4.79139999999966
6.17830000000023 1.22700000000004 7.68889999999995
7.06990000000022 -2.06339999999996 6.14289999999995
23.7249000000001 0.928800000000017 8.12769999999995
31.3557000000001 0.834100000000065 -2.88150000000001
31.1424000000001 -2.11569999999994 -6.2859
65.304099999998 3.78119999999966 -3.4145999999997
69.4157999999976 12.7410999999996 -3.95869999999964
ID=PHAtriMALSIEBMNH5661**

LM3=54
6.03550000000184 0.317800000000463 0.560599999999843
7.62150000000068 -0.606699999999619 0.609399999999925
25.1665999999999 1.4599000000001 -3.81930000000005
27.2899 3.41180000000003 -4.63550000000004
28.9866000000003 8.55630000000009 -4.95580000000006
31.6946000000005 7.16530000000023 -4.55370000000007
31.5340000000006 7.79780000000004 -6.01350000000009
41.0497000000005 0.423200000000136 4.84829999999985
39.1355000000002 12.2050000000001 -9.3129000000001
44.5487000000006 11.8462000000003 -6.16430000000016
46.8648000000002 9.35750000000013 -8.70720000000014
42.5035000000002 0.318500000000173 -9.76380000000014
52.6037000000005 10.3181000000003 -9.70760000000021
55.0362000000002 4.84730000000019 -10.1946000000002
62.1573000000002 10.2030000000002 -10.4658000000002
65.9490000000003 12.1765000000002 -9.65720000000027
66.4288000000001 19.3569000000002 -11.1564000000003
71.8434000000001 20.1022000000002 -2.60260000000028
70.3848000000001 9.72220000000019 -7.85730000000027
70.1108000000002 15.2499000000002 -10.9211000000003
5.15270000000074 2.50950000000019 0.799899999999911
5.57570000000063 3.33750000000019 0.581399999999913
21.1152000000004 12.3458000000001 -3.92130000000005
24.2004000000004 13.5971000000001 -4.59990000000005
29.6235000000004 12.8985000000001 -4.56070000000006
30.5512000000003 12.0652000000001 -6.15020000000005
34.6085000000006 22.9337000000002 4.94849999999987
44.2914000000005 13.6567000000002 -5.63880000000015
45.0403000000004 17.6175000000002 -9.41600000000015
37.0758000000003 24.2154000000001 -10.0508000000001
50.1736000000004 19.7970000000002 -10.0373000000002
50.8145000000002 26.6379000000001 -10.1416000000002
58.5072000000003 25.5990000000002 -10.7236000000002
62.6973000000002 25.3554000000002 -9.28370000000025
65.9890000000001 29.3456000000002 -7.20670000000027
67.1568000000001 23.3732000000002 -10.9339000000003
4.8317000000007 2.07670000000019 6.81709999999994
6.4448000000007 5.39370000000019 6.38299999999993
20.7604000000008 11.9859000000002 7.2790999999999
26.1037000000005 15.2754000000001 -2.91400000000007
24.6705000000004 16.1739000000001 -4.69560000000006
29.3805000000007 9.21650000000021 8.96649999999988
52.3354000000005 14.8590000000002 12.1352999999998
59.5924000000004 17.2171000000002 10.0287999999998
60.0947000000002 33.3452000000002 -3.60390000000024
70.1494000000001 20.0309000000002 4.80569999999973
68.8257000000001 26.9769000000002 -5.25390000000027
5.32360000000072 1.84170000000019 6.72749999999992
7.08500000000081 -0.498499999999748 6.24449999999991
22.9345000000007 3.00130000000023 7.68109999999989
30.7385000000005 1.98080000000021 -2.73840000000009
29.8186000000003 -0.121899999999833 -4.85990000000008

68.4322000000002 3.70200000000017 -3.90350000000028
72.5179000000002 13.5801000000002 -5.70010000000029
ID=PHAtriUNKUGABMNH63852*

LM3=54
6.62269999999853 -0.795800000000671 1.62000000000016
6.98019999999977 -2.56190000000012 0.969600000000022
24.6583999999992 -0.695600000000272 -4.31019999999994
28.0268999999994 1.08499999999964 -5.61749999999997
26.2621999999999 6.92569999999985 -4.49679999999998
32.2005000000001 4.91709999999995 -4.7494000000001
30.1860000000008 4.84980000000012 -6.16000000000013
39.8748999999998 -0.892900000000254 5.31579999999998
36.1763000000002 10.9254999999999 -9.52290000000004
40.5229000000001 10.0541999999998 -4.42010000000004
44.4274000000001 8.73269999999977 -7.17010000000001
42.0813999999999 -2.21830000000021 -8.58470000000001
49.7580000000005 9.27209999999988 -8.69200000000008
51.0333000000007 3.2749999999999 -8.65700000000009
59.3492000000008 10.3737999999999 -9.5048000000001
62.4339000000011 11.8703999999999 -9.28240000000014
60.5617000000011 17.9268999999999 -10.8082000000001
67.7740000000014 20.3901999999999 -4.44660000000017
67.1158000000013 10.3883999999999 -7.27440000000016
65.4643000000012 14.6362999999999 -10.9908000000002
4.22159999999941 2.17139999999963 0.25140000000007
5.20849999999945 4.02259999999965 -0.443299999999958
21.0546999999998 12.4861999999999 -5.61340000000002
24.0460999999998 12.3481999999999 -5.75780000000001
29.5739999999998 11.9645999999999 -5.13040000000003
27.7586999999999 11.5171999999999 -6.53620000000002
32.8694000000001 21.8041999999998 4.92299999999994
41.4582000000001 13.0087999999998 -4.14960000000005
42.6676000000002 15.9232999999998 -7.66240000000006
34.0571000000003 23.0602999999999 -8.25390000000007
46.4540000000005 18.6526999999999 -9.10340000000009
44.9521000000006 25.2590999999999 -7.9574000000001
55.0146000000009 23.1855999999999 -9.49330000000012
58.7732000000011 23.7995999999999 -9.24500000000015
61.3659000000013 28.5325999999999 -7.09870000000017
63.3632000000014 23.1086999999999 -11.2290000000002
5.03339999999943 1.87539999999964 8.15290000000003
4.66679999999948 5.34509999999963 6.80070000000002
20.7288999999996 11.9225999999997 7.87300000000001
26.3143999999999 15.9088999999999 -4.30190000000003
24.6170999999999 18.3456999999999 -6.40370000000003
31.4438999999997 9.81199999999973 10.6989
49.6756000000005 15.8968999999998 12.4419999999999
57.0506000000007 17.9119999999998 10.3978999999999
56.2289000000011 32.7776999999999 -2.27020000000013
66.6308000000012 20.8348999999999 4.28579999999985
64.4751000000013 27.0635999999999 -4.99320000000016
4.82269999999942 1.28719999999962 7.17590000000001
6.03189999999941 -1.40950000000035 6.07590000000003

24.1018999999994 1.9463999999997 7.38840000000003
32.2728999999999 0.873299999999834 -5.58510000000001
32.3224999999997 -1.73750000000019 -6.91819999999999
66.1696000000011 5.40499999999987 -2.25250000000012
68.5177000000013 15.1633999999999 -4.69640000000016
ID=PHAtriFEMGHABMNH707161

LM3=54
4.46120000000105 -1.13440000000009 -0.79320000000006
5.51269999999994 -1.24950000000025 -1.58559999999995
24.5138999999996 -0.0268000000001316 -6.40329999999999
23.3753999999997 0.911299999999855 -6.26259999999997
21.7360999999998 6.33519999999987 -4.59220000000001
27.8523999999998 5.28019999999989 -5.79610000000002
25.5289000000003 4.50320000000001 -6.60140000000002
34.7186 -0.096600000000055 5.33409999999997
34.4349758898141 10.7194212892244 -9.77487120865516
38.6288 10.7611999999999 -4.96250000000009
41.9185999999998 8.64299999999989 -6.92310000000007
38.7002999999997 -1.57620000000009 -9.01310000000005
47.2164999999998 9.31319999999985 -7.94660000000009
48.1914 4.23489999999993 -7.95200000000009
56.2849000000003 10.319 -7.84980000000017
59.8370000000005 12.2474 -6.78140000000019
56.6523000000004 17.3819 -8.32690000000018
64.9812000000006 20.313 -1.19530000000019
64.1935000000004 10.8687 -5.06670000000019
66.3017000000003 14.3388 -6.5408000000002
4.1524000000011 2.78879999999998 -0.159300000000083
4.811800000001 4.21599999999994 -0.870100000000078
19.9424000000004 13.455 -6.45590000000007
19.4545000000003 11.8836999999999 -6.45460000000005
25.3070000000001 11.4389999999999 -5.15790000000006
22.9688 10.6716999999999 -6.17900000000003
29.6847000000006 21.2283 4.97079999999987
38.1391000000002 12.911 -5.1449000000001
39.2051000000003 16.1555 -6.74230000000011
31.3723000000005 23.6606 -9.00990000000012
44.4496000000004 18.7551 -7.70840000000014
42.4262000000005 24.4114 -7.60580000000014
51.3463000000005 23.2457 -8.34960000000017
55.4829000000005 24.5758 -6.92530000000019
59.2271000000007 27.5538000000001 -4.80860000000022
63.0088000000006 25.7744 -6.8695000000002
4.60780000000107 1.15119999999997 4.84409999999991
4.70330000000113 3.96559999999999 4.35329999999991
17.7916000000005 10.7292 6.48639999999992
23.0646000000003 13.9363 -3.06350000000008
22.6578000000004 16.3462 -7.30780000000008
27.1596000000003 8.92589999999997 9.41469999999992
44.6499000000006 14.0771 12.4643999999998
53.5292000000006 16.534 10.2642999999998
54.9296000000008 31.5389000000001 -1.6704000000002
60.4607000000006 18.85 6.98079999999977

60.7995000000007 26.6736000000001 -2.85790000000021
5.39020000000103 1.28059999999997 5.62349999999992
6.02290000000074 -1.45580000000009 4.62289999999995
20.9982000000002 2.1503999999999 6.82799999999997
26.4796999999995 2.00859999999983 -4.2089
27.0034999999996 -1.09570000000016 -7.26719999999998
62.8176000000003 4.86849999999996 -1.56700000000018
64.9433000000005 12.7369 -3.73480000000021
ID=PHAtriUNKIVCMNHN622118

LM3=54
4.63780000000066 -0.477499999999828 2.32499999999984
5.67609999999951 -0.550300000000035 2.21419999999996
24.6843999999995 0.360800000000052 -3.58640000000011
24.5412999999996 0.946200000000009 -3.63880000000011
27.1147999999997 7.45390000000001 -2.58840000000014
29.1674999999997 4.52660000000002 -2.82320000000015
28.2142000000003 4.77260000000015 -4.15900000000016
37.5162999999999 0.287400000000113 6.4907999999998
37.286621177452 10.6173244036615 -8.67440333300348
41.6525999999998 10.4157 -4.96110000000021
43.9270999999996 9.39100000000001 -6.85830000000019
41.8566999999996 -1.54399999999992 -6.9142000000002
49.8597999999995 9.35999999999997 -8.54050000000022
52.2290999999998 4.12880000000008 -8.90970000000025
60.0954999999999 9.80160000000015 -9.04060000000034
64.8401000000001 10.9670000000002 -8.89720000000037
62.0556999999998 17.1686000000001 -11.1974000000004
70.3523999999999 19.1127000000002 -5.36240000000039
69.4198999999999 9.35950000000018 -7.62400000000039
69.5587999999998 12.5708000000002 -11.3281000000004
4.35120000000072 3.05230000000017 2.08969999999981
4.95400000000063 5.06510000000012 1.75189999999982
21.1032000000002 12.4004000000001 -3.39620000000019
21.3483 12.6271000000001 -3.70670000000018
26.7220999999999 11.9714000000001 -3.39940000000018
26.1378999999998 10.4979 -3.89470000000016
32.4756000000003 20.0521000000002 6.91489999999974
40.2553 13.1615000000001 -4.73630000000023
42.5535 15.4652000000001 -6.55360000000023
35.2248000000002 23.2395000000001 -7.03630000000025
47.5080000000001 18.1120000000001 -8.36190000000028
46.5399000000001 23.2170000000001 -8.8225000000003
55.5219 23.2446000000001 -9.54180000000034
61.1899999999999 25.2564000000001 -9.09720000000036
62.868 27.6707000000002 -7.2393000000004
65.4542999999998 25.0516000000001 -11.3358000000004
2.48320000000068 1.31850000000017 6.93089999999981
2.61630000000075 4.70380000000018 6.42369999999981
18.0516000000003 10.4694000000001 9.19999999999979
26.0246000000001 14.7238000000001 -2.7692000000002
24.5374000000001 15.2335000000001 -4.49690000000019
29.0634000000001 8.16270000000011 11.6092999999998
52.1332000000002 14.0887000000002 12.4548999999997

60.8641000000002 16.3812000000002 10.0328999999996
61.7023000000001 30.7525000000002 -2.02790000000037
67.1975 17.8874000000002 5.8940999999996
65.7105999999999 27.7711000000001 -6.18070000000039
2.54100000000064 0.657700000000173 6.95789999999981
3.86510000000035 -2.16089999999988 6.08369999999983
20.9009 0.791100000000074 9.03979999999984
29.3573999999995 1.28339999999998 -2.23550000000013
28.1804999999995 -0.394199999999995 -5.00720000000013
67.1790999999998 4.43650000000017 -2.34400000000035
69.7588999999999 9.8095000000002 -6.05030000000039
ID=PHAtriUNKCARMNHN967981

LM3=54
4.52349999999897 -0.774499999999962 1.23470000000004
6.44790000000004 -0.621799999999693 0.761899999999984
24.5013999999999 0.138800000000133 -3.23130000000003
24.1665000000001 0.461800000000177 -3.46180000000003
24.1886999999998 6.53040000000011 -2.64300000000004
27.7854 4.6602000000001 -2.77230000000005
27.2878000000002 5.38580000000017 -3.90200000000004
37.1875999999999 0.287200000000141 6.71409999999992
35.3238999999998 10.0406000000001 -7.60660000000007
39.4612999999998 9.83660000000003 -3.14340000000009
41.3345999999997 8.58640000000007 -5.33060000000008
39.5406999999998 -0.069899999999908 -6.68500000000006
46.8128999999996 7.95700000000003 -7.06540000000009
47.8524999999995 4.12230000000002 -6.75940000000008
56.3151999999995 9.32270000000002 -6.55100000000013
60.5702999999993 10.1355 -5.05160000000011
59.9606657555521 16.4426076090305 -7.09055995377169
65.3335483713228 18.0806059893221 -0.939580951908354
64.8350841601355 8.55242955306018 -4.58595230486235
65.2998174816959 12.8172855291059 -6.6307596912144
4.66519999999914 3.27320000000006 1.0089
5.73079999999922 4.25700000000007 0.703700000000002
22.0910999999997 12.5153000000001 -2.90980000000005
22.1351999999997 11.6731000000001 -2.93330000000005
26.3839999999998 10.6100000000001 -2.41670000000007
25.8383999999998 9.3029000000001 -3.84130000000006
31.5981999999997 17.7659000000001 6.91129999999992
39.4952999999998 11.3425 -2.54930000000009
40.0693999999996 14.0103 -4.82840000000009
34.0382999999995 21.2362 -6.11050000000009
44.6083999999995 16.7984 -6.7785000000001
42.9456999999993 21.6921999999999 -6.3999000000001
52.5722999999993 21.2125 -6.30400000000011
57.1089999999992 22.3425 -5.31830000000011
60.1041255193431 25.6490233966194 -4.55306130285418
62.6666893355265 22.1998287275599 -6.79685864285654
3.7408999999992 1.30110000000007 6.97280000000002
4.06609999999916 3.69840000000005 6.7151
21.0939999999997 8.50700000000011 10.4054999999999
24.4865999999997 12.9839000000001 -0.0738000000000705

22.8216999999996 13.4414 -3.48010000000006
27.7083999999998 6.85930000000011 11.5400999999999
46.4186999999995 12.4848 12.7467999999999
54.4581999999994 14.5881 10.7373999999999
54.1995999999992 28.9788 -2.54050000000013
63.0243486451703 17.1625168948155 5.72448169347589
61.6772676018133 24.2503275875847 -2.80556941392632
3.55789999999922 0.178800000000074 6.61210000000001
5.67289999999941 -2.01229999999987 6.4093
23.0827999999999 2.60270000000016 9.89639999999995
26.9131 0.470000000000116 -1.46390000000003
25.8599 -0.419399999999888 -3.87700000000003
61.1429999999994 3.42160000000005 -3.01720000000012
65.5991981473596 10.7725536821746 -2.67475231727634
ID=PHAtriUNKCONKMMA19624*

LM3=54
-1.86100000000014 -3.93160000000053 -7.63779999999991
-1.33369999999979 -4.00470000000044 -7.87449999999996
17.9911999999999 -2.03570000000035 -9.10459999999985
17.6108 -1.42700000000027 -9.16049999999982
18.9041 5.52879999999976 -8.67109999999979
21.6474 3.05839999999976 -7.85229999999981
20.8654 2.93179999999976 -9.17899999999979
30.1041999999999 -1.0806000000003 2.99940000000019
29.9694000000001 10.4551999999998 -10.7875999999999
34.0204000000002 10.6279999999999 -6.08619999999988
37.4285000000002 8.79599999999984 -8.22989999999988
35.4780000000001 -1.97800000000023 -9.7854999999998
42.4282000000003 10.2073999999998 -7.64549999999988
43.3252000000002 4.5459999999998 -8.30109999999985
51.2887000000006 10.3025999999999 -6.77339999999989
56.3078000000006 12.2631999999999 -4.94669999999989
54.6326000000006 17.9841999999998 -6.93609999999988
58.7829000000006 20.0574999999998 1.14450000000012
58.6740000000005 9.3719999999998 -2.32319999999986
60.2727000000006 14.3286999999998 -6.08789999999986
-3.71169999999972 0.550099999999576 -7.86679999999981
-3.52789999999972 1.03649999999957 -7.94329999999979
13.0201000000001 11.0060999999997 -9.18819999999982
13.3334 10.7922999999997 -9.20089999999987
18.5929 9.69749999999975 -7.99909999999985
17.8574 8.74619999999976 -9.06399999999983
24.0026000000001 18.6160999999997 2.53540000000013
33.6214000000001 11.8916999999998 -6.29439999999988
35.0527000000002 14.6770999999999 -8.0131999999999
26.4157000000002 22.5364999999998 -9.23539999999987
39.3846000000003 17.5440999999999 -8.16469999999991
36.9263000000004 21.9122999999999 -8.42839999999991
46.0792000000006 22.5725999999999 -7.64569999999992
51.6159000000007 25.6373999999999 -4.8954999999999
52.6735000000006 27.7798999999998 -2.61139999999988
55.8857000000007 24.5626999999998 -6.28619999999986
-2.60881631738245 -0.791419068300396 -1.17845309732382

-2.66834677695337 1.83944565779147 -2.16018060181014
11.0665000000001 7.77909999999964 2.26650000000017
18.418 12.1534999999997 -5.54739999999986
15.8601000000001 14.3855999999998 -9.22999999999986
20.2298 6.06199999999968 6.16320000000018
38.4799000000002 12.7108999999997 12.5143000000001
45.0864000000003 14.1402999999997 12.3511000000001
47.4391000000005 29.8738999999998 0.824600000000098
55.0435000000005 17.4208999999997 10.0736000000002
54.6148000000006 27.0320999999998 -0.61419999999986
-2.64286257092909 -1.41448664830553 -1.22418902344999
-1.86759999999973 -3.93890000000046 -1.88619999999978
14.1696000000001 -0.646700000000348 2.78680000000021
21.4461999999999 0.449399999999728 -5.64269999999979
20.7762 -2.75170000000028 -9.44989999999978
56.2172000000005 4.83359999999978 0.758200000000172
60.2745000000005 12.3110999999998 -0.528999999999866
ID=PHAtriUNKLIBKMMA38506*

LM3=54
5.20278262101984 -0.487260224707059 1.48661215676785
6.42670000000063 -1.02770000000022 0.722499999999955
26.5252000000011 -1.02309999999994 -4.72300000000009
25.2879000000012 -0.278799999999739 -5.14080000000014
27.2701000000007 6.97579999999996 -5.38870000000005
30.029900000001 3.91870000000011 -5.13770000000009
29.5790000000012 4.42720000000014 -6.61220000000008
38.9908000000011 -0.468699999999838 6.81759999999991
38.2108000000011 9.81000000000018 -10.0351000000001
42.9054000000011 9.75030000000024 -4.7354000000001
46.3303000000007 8.07180000000018 -7.19820000000007
43.4111000000011 -2.48889999999983 -8.7316000000001
51.8205000000007 9.03100000000022 -7.63960000000006
52.7416000000009 3.69810000000024 -8.04440000000006
62.3297000000004 9.00360000000021 -8.45770000000007
66.4422000000007 10.4270000000003 -7.55860000000009
65.1677000000006 16.6109000000003 -9.58780000000009
71.0499000000005 18.0446000000003 -2.70900000000008
70.4949000000006 8.43880000000027 -5.73490000000009
71.9172000000007 12.0985000000003 -8.61010000000009
4.27400000000048 2.26759999999967 2.14130000000003
4.62630000000039 4.06079999999965 1.48310000000005
22.3871000000005 13.7903999999999 -5.0337
22.0625000000005 12.0011999999999 -5.3249
27.2730000000008 10.3836 -4.85070000000005
26.8323000000008 9.80470000000001 -6.18220000000004
34.4423000000006 18.2425000000001 6.29469999999997
41.877100000001 11.4133000000002 -4.83450000000008
44.4016000000008 14.6764000000002 -7.22880000000007
36.8271000000006 23.4648000000001 -7.88640000000004
50.3809000000007 16.9834000000002 -8.10290000000007
49.5158000000005 21.8593000000002 -7.62330000000007
58.3027000000005 22.0385000000003 -9.10050000000008
62.5278000000005 23.9355000000004 -7.47090000000009

66.7309000000004 25.7566000000003 -5.95180000000007
69.0919000000005 23.8914000000003 -9.30630000000008
4.35410000000049 1.47249999999968 8.7525
4.75620000000039 4.05469999999966 8.07580000000003
19.8306000000004 9.09699999999983 8.55340000000001
28.0213000000006 13.2336 -3.76280000000002
24.6450000000006 15.2975 -5.73250000000002
30.2306000000007 7.13069999999999 11.2091
53.1136000000006 12.8170000000002 11.6408999999999
61.6671000000006 14.2557000000002 10.3393999999999
63.2268000000003 28.4616000000003 -5.16140000000007
67.3896000000004 15.9627000000003 7.71279999999992
68.7871000000004 23.8604000000003 -3.99880000000007
4.21570000000052 0.710599999999699 8.71150000000001
6.0608000000006 -0.96320000000028 8.0239
22.6161000000007 1.0451999999999 8.69609999999997
29.9288000000014 0.558800000000179 -3.63620000000012
28.7374000000013 -2.24059999999983 -5.28780000000012
68.4889000000009 3.24800000000027 -5.25910000000009
71.7067000000006 10.2422000000003 -3.63640000000009
ID=PHAtriUNKCONKMMA42M131

LM3=54
6.9787 0.5317 1.1119
7.886 0.0993 0.8728
25.7434 -0.2379 -4.2733
25.0842 0.4074 -5.0008
28.5985 7.7199 -5.5006
31.2831 4.3323 -5.6464
29.7318 5.183 -6.2666
40.7599 -0.21 4.9253
36.5433 10.4288 -10.5145
44.1039 10.9425 -6.7328
46.4254 8.6421 -9.7039
43.3376 -1.9584 -10.1981
52.3025 8.9059 -11.9827
54.2894 3.4882 -11.1946
61.0122 9.1594 -12.9271
64.9711 10.8665 -12.9206
65.2596 17.8793 -14.7993
71.3771 19.2266 -8.6254
70.1659 9.3982 -11.3314
70.0317 13.4874 -15.1777
6.1321 3.457 1.533
6.8455 3.8391 1.4871
22.1191 13.5954 -3.7934
22.4354 13.3987 -4.7526
28.5611 12.2111 -5.67
27.6008 11.0866 -6.289
35.7756 20.8539 5.2628
43.6375 12.5802 -6.5182
44.1527 15.8045 -9.4358
36.3147 23.7181 -9.8065
49.3183 18.868 -10.9285

48.1903 24.7014 -10.2112
56.4767 23.1942 -12.9745
61.7565 24.6257 -12.6824
64.9206 27.0852 -10.5552
67.4636 24.2427 -14.6988
5.7306 1.9341 8.4818
5.2471 4.7198 7.2483
21.5258 11.0561 8.6886
27.3086 16.042 -4.0477
25.468 17.4211 -5.3325
31.6648 8.3229 11.1488
53.5427 13.2205 10.5338
62.277 16.3192 7.1639
60.4358 30.4576 -5.6987
69.2591 18.0547 1.1299
67.0622 25.9761 -8.9619
5.9202 0.9664 7.8201
7.574 -2.2624 6.8118
23.9405 0.7199 8.871
31.4409 -0.2098 -3.2659
30.1046 -2.4928 -5.8978
66.7419 3.2471 -6.0882
70.6282 10.6531 -9.3594
ID=PHAtriUNKCAMKMMA732M78

LM3=54
4.5827 -0.5118 1.1978
5.819 -1.1559 0.563
25.0257 -0.634 -4.6094
23.7741 0.1035 -4.8509
27.0514 7.6815 -5.2933
30.2849 5.0558 -5.4308
29.1485 5.5283 -6.5376
37.0909 0.1898 6.408
35.2837 10.2247 -9.0274
41.0012 10.6203 -5.8313
43.3288 9.2472 -8.3421
41.1087 -1.662 -8.366
49.7622 9.4164 -9.3509
51.4165 4.2683 -8.836
57.3662 9.0043 -10.4103
61.6772 11.1041 -9.1238
59.3015 17.387 -11.4597
65.8021 19.8157 -3.3092
64.9338 10.4456 -7.7166
65.2361 14.4701 -10.8502
3.1515 3.0581 1.1253
3.8749 4.5622 0.4401
20.4624 13.7983 -4.7231
20.1863 12.0953 -5.0288
27.3179 11.6474 -5.3324
26.1627 10.4826 -6.8284
31.1786 19.388 5.9374
40.452 12.8706 -5.7331

41.4101 15.3037 -8.2131
33.4825 23.6407 -8.0374
46.8934 18.8578 -9.4637
44.5684 23.3905 -9.1189
53.2697 22.5635 -10.1823
57.1685 24.6569 -9.0889
59.8563 25.821 -8.0368
62.6149 23.4102 -11.3532
4.2598 0.5146 7.3996
3.8818 3.8494 6.3849
20.0446 10.3057 8.4971
25.0565 14.333 -3.6071
23.0069 15.3428 -5.0016
30.7912 8.2057 10.8704
50.189 14.6011 11.3467
58.7488 16.1807 7.9938
56.7851 29.6523 -3.9777
64.561 17.8646 3.895
62.4412 25.1184 -5.5449
4.3689 -0.1473 6.9816
5.9175 -2.7011 6.2118
23.3737 1.1593 8.1658
28.4031 0.6387 -3.7233
27.4422 -1.4574 -4.9614
63.6145 4.716 -4.3836
66.5561 12.5446 -5.1175
ID=PHAtriUNKCONKMMA06M588

LM3=54
6.2137 -0.101 1.7081
7.2757 -0.4669 0.9296
26.3098 -0.2556 -3.4434
26.2613 6.5883 -2.6797
25.54 0.9961 -3.6056
29.612 4.2994 -3.5369
28.6627 4.6787 -4.4353
37.2302 0.0793 6.0328
39.2709 9.8503 -8.3355
42.333 10.1105 -3.4003
44.7882 8.0799 -6.2953
42.2474 -1.2254 -7.0721
49.7465 7.9636 -6.9933
50.3255 3.8896 -6.6944
57.9275 7.8495 -7.2151
62.1083 9.1724 -5.2967
62.4799 15.0518 -7.3225
67.4018 18.0662 1.3507
66.0362 6.6657 -3.0766
67.7802 11.0382 -5.2046
5.5952 3.6201 2.0904
6.3464 5.0021 1.3363
23.1268 12.639 -3.2797
23.4813 11.9825 -3.564
27.6603 10.6461 -3.3951

27.0531 9.6885 -4.107
33.4465 17.9076 5.5208
41.9226 10.9724 -3.2929
43.227 13.5472 -6.6874
36.9548 20.8616 -6.1324
48.2817 15.6652 -7.0917
46.5307 20.1135 -6.946
55.1637 19.7325 -7.2504
58.6652 21.8747 -5.1918
62.5782 24.1594 -3.1577
65.3036 22.5003 -5.323
4.8747 1.8932 7.2628
4.631 4.3992 6.2363
21.0375 10.5219 8.4203
26.3728 13.8382 -2.7821
27.1091 14.7454 -4.6509
32.5703 7.9446 11.1924
50.2978 12.5548 12.8295
58.1152 13.9853 11.2541
58.8239 26.9904 -2.1217
65.0304 15.2298 8.2435
64.4133 22.3845 0.2228
5.2638 1.2604 7.3235
5.9569 -1.1595 6.6005
23.5266 0.4841 8.2936
30.0331 0.1439 -2.3404
29.6484 -1.1879 -4.4082
63.8609 2.8478 -2.3235
67.1461 8.292 0.3359
ID=PHAtriUNKCONKMMA42M129

LM3=54
5.40829999999779 -0.494600000000105 1.08750000000013
6.37939999999912 -0.870599999999513 0.843399999999961
27.1479999999988 -0.474499999999902 -5.66039999999983
26.1298999999989 1.08989999999997 -6.34699999999988
28.1398999999996 8.43140000000008 -7.20279999999991
31.2052999999998 5.49710000000023 -6.9085
30.3988000000004 5.86570000000038 -8.45000000000006
39.2390999999991 0.462800000000182 4.18500000000003
37.2881999999998 11.0574000000002 -11.8904999999999
42.0513999999996 10.9654000000001 -7.69809999999995
44.1337999999995 9.15170000000012 -10.0978999999999
42.0868999999994 -1.43709999999981 -9.87749999999994
49.2960999999999 9.33020000000026 -11.1048
50.3858 3.94860000000033 -10.4547000000001
58.5949000000001 9.71550000000038 -11.7404000000001
62.8523000000004 11.8905000000004 -11.2274000000002
61.9491000000003 17.2435000000004 -12.7186000000002
68.2571000000004 19.8794000000005 -6.96100000000021
66.6778000000004 9.93490000000051 -9.02120000000022
68.0755000000003 14.1960000000005 -12.1430000000002
4.78509999999861 3.41740000000013 0.963200000000025
4.99959999999869 5.06540000000014 0.495400000000015

22.6004999999995 15.3452000000001 -5.64509999999994
22.5763999999995 13.4951000000001 -6.48189999999993
28.5566999999996 12.6208000000001 -6.69339999999992
28.5752999999996 11.0221000000001 -8.30449999999992
33.6133999999995 20.9357000000001 4.48599999999999
41.1530999999996 13.7882000000001 -7.44439999999995
42.3940999999997 15.4688000000002 -9.70619999999996
34.8738999999999 23.8742000000002 -9.2145
46.8751999999999 18.2829000000003 -10.7911
44.6268 22.9543000000003 -10.4329000000001
54.8174000000002 22.4081000000004 -12.0277000000001
59.9636000000003 23.5728000000005 -10.8113000000002
62.6578000000004 26.4975000000005 -8.43050000000019
65.2973000000005 24.2445000000005 -12.4643000000002
5.94269999999859 1.98790000000017 7.89019999999995
6.20139999999862 4.91080000000014 6.82059999999996
22.014399999999 11.9196000000001 7.81270000000002
26.5516999999996 16.1167000000001 -5.50689999999994
26.1244999999996 17.1913000000001 -6.91909999999994
31.5270999999991 9.25410000000009 9.7542
51.2829999999996 14.0690000000003 10.4801999999999
58.2049999999998 16.4345000000003 8.59229999999986
57.6632000000002 30.2589000000005 -5.66970000000016
65.6203000000002 18.7788000000005 3.34609999999979
65.0324000000003 25.0590000000005 -7.48430000000019
5.72039999999856 1.16980000000018 7.80319999999994
7.67499999999857 -1.12869999999979 6.97389999999998
24.6497999999988 2.1591000000001 8.00670000000005
29.9609999999994 -0.154599999999846 -4.57989999999992
30.2064999999993 -0.939799999999838 -6.83809999999991
65.2265000000002 4.11400000000046 -5.04650000000018
69.2537000000004 12.3807000000005 -7.08510000000023
ID=PHAtriUNKCONKMMA42M136

LM3=54
4.60659999999852 -0.0253000000003971 2.13170000000018
5.82799999999976 -0.496499999999885 2.3884
23.7743999999991 -0.0846000000001354 -4.18259999999988
22.8487999999992 0.626999999999771 -4.05669999999995
25.4414999999997 7.78399999999992 -5.02029999999998
28.6136999999999 4.92930000000004 -4.53320000000006
27.1433000000006 4.84790000000018 -5.41100000000012
37.1785999999997 0.464499999999958 5.99210000000001
35.9777 10.562 -10.4027
38.6393999999999 8.90749999999995 -4.72590000000001
40.5670999999998 8.49209999999992 -8.0711
38.6686999999997 -1.90260000000006 -8.898
47.2706000000003 7.54520000000004 -9.88950000000006
47.7796000000004 3.84480000000009 -9.34350000000009
54.6504000000006 8.54550000000013 -10.5872000000001
58.495500000001 9.89720000000017 -9.40710000000018
57.1848000000009 15.9912000000002 -12.8392000000002
64.3956000000011 18.4634000000002 -6.60180000000021
62.2231000000011 8.17020000000021 -8.2338000000002

62.561600000001 11.7811000000002 -11.7670000000002
4.16869999999933 2.50839999999981 2.05520000000003
4.98239999999939 3.57779999999983 2.54170000000001
20.5052999999998 13.2526 -4.22490000000002
19.5129999999998 11.7571999999999 -4.25860000000002
26.1511999999998 10.9706999999999 -4.56810000000001
24.5647999999998 9.98699999999994 -5.79520000000001
32.3361 19.5519999999999 5.42549999999993
38.1979999999999 11.9634999999999 -5.47480000000003
39.1746 14.2907 -7.98420000000003
32.4039000000002 22.2389 -9.25710000000007
43.8923000000003 16.8474000000001 -10.0709000000001
42.7539000000005 21.0318000000001 -9.59030000000011
51.0239000000008 21.6495000000001 -10.1324000000002
56.247700000001 22.5945000000002 -9.23540000000019
58.8454000000011 25.6289000000002 -7.9467000000002
59.5548000000012 22.9579000000003 -11.8101000000002
4.82009999999939 1.39539999999983 9.20689999999995
3.87749999999941 4.36589999999981 8.36999999999998
19.8909999999995 9.92389999999985 9.6135
24.9975999999998 13.3240999999999 -2.96640000000002
21.9767999999999 14.7808 -5.38990000000003
27.7793999999996 7.51419999999989 10.5165
48.1378000000002 12.6478 10.5417999999999
56.9560000000005 14.6321000000001 7.61599999999986
55.0965000000009 28.4417000000002 -4.2952000000002
63.7614000000009 16.2072000000002 1.63959999999979
60.1139000000011 23.8747000000002 -7.36340000000021
5.10929999999938 0.962399999999837 9.42559999999997
5.65029999999935 -2.29610000000014 7.84280000000001
21.8182999999993 1.28329999999986 8.92610000000004
27.6424999999996 1.31809999999994 -3.36859999999998
25.0831999999996 -1.68990000000007 -5.28669999999996
60.0968000000008 2.55830000000017 -5.77760000000017
63.6188000000011 10.0527000000002 -7.6227000000002
ID=PHAtriUNKCONKMMA42M146

LM3=54
3.29250000000179 -0.807199999999444 0.714299999999942
5.98990000000171 -1.2482999999995 -0.617299999999991
23.3045000000012 -0.606899999999572 -5.25119999999998
23.8151000000013 0.712500000000458 -6.30499999999996
25.0417788950521 7.79620044060873 -6.33564322464752
28.653300000001 5.52760000000038 -5.87809999999991
28.0235000000011 5.6742000000004 -7.24869999999992
35.978400000001 1.10080000000041 6.25960000000006
35.1795715820053 11.2529218993576 -10.405043397024
39.9000000000008 11.4097000000003 -5.3447999999999
42.7327000000008 9.55530000000034 -8.29959999999989
39.8004000000012 -1.50499999999954 -8.76559999999991
47.8649000000008 9.57210000000034 -9.09589999999985
49.701700000001 4.53910000000043 -8.96379999999986
58.0705000000009 11.3182000000004 -9.87429999999982
62.1072000000011 12.9622000000005 -9.07009999999979

61.3218000000009 19.1317000000004 -11.1847999999998
67.4398000000011 21.7748000000005 -3.81449999999977
66.9496000000011 11.1028000000005 -7.10149999999978
67.8483000000011 15.8878000000005 -9.5852999999998
2.13000000000184 2.70330000000054 0.512999999999971
3.66010000000178 4.68470000000052 -0.392499999999991
19.2168000000012 14.3300000000004 -5.08139999999994
19.5672000000011 12.8801000000004 -5.91629999999994
25.7579000000009 12.1324000000004 -5.52579999999996
24.9758000000009 11.1544000000004 -7.15879999999994
32.1672000000009 21.6043000000003 5.05560000000008
39.4580000000008 13.6182000000003 -5.85459999999989
40.5644000000008 16.3774000000003 -8.17389999999989
31.7773000000008 24.2948000000003 -9.33619999999986
45.4301000000007 18.8740000000003 -9.48869999999985
43.1600000000008 24.0898000000003 -9.34069999999985
53.2972000000007 23.8179000000003 -10.1709999999998
57.9184000000008 25.6637000000004 -9.09929999999976
60.6296000000009 29.1454000000004 -7.50989999999975
63.8669000000009 26.5880000000004 -10.0086999999998
2.51720000000181 0.957600000000535 7.36170000000001
3.30350000000179 4.56160000000054 6.46959999999999
18.5602000000012 10.5572000000004 8.0715
24.6284000000009 15.4527000000003 -4.78099999999992
22.262100000001 16.9455000000004 -6.09339999999994
28.9771000000011 9.40080000000041 10.5355000000001
49.3971000000009 15.2644000000004 11.9963000000001
57.8444000000009 17.6136000000004 9.90830000000013
55.9582000000009 33.1301000000004 -4.54279999999977
66.189600000001 20.1503000000005 5.38370000000022
63.1169000000009 28.1117000000004 -5.91359999999976
2.93660000000181 0.279100000000545 7.44629999999995
5.33290000000179 -1.93079999999947 6.42169999999996
21.0729000000013 1.96190000000044 7.91280000000002
28.3159000000012 0.595600000000428 -4.82179999999994
27.4622000000012 -1.95519999999954 -6.02799999999994
64.887200000001 4.75250000000052 -4.3224999999998
68.2017000000011 13.5603000000005 -4.90729999999977
ID=PHAtriUNKCONKMMA42M157

LM3=54
4.6876 -0.7137 2.749
6.2976 -1.3851 1.2592
24.5334 -0.262 -2.9033
25.0172 1.9001 -3.7398
26.973 7.3903 -3.5751
29.4564 5.0317 -3.8656
28.4888 5.468 -5.3216
38.5511 0.3954 5.7678
34.9376 10.1056 -8.154
40.7872 10.0708 -3.8977
43.6698 8.6368 -7.1823
41.1859 -0.5843 -7.9974
48.1841 9.1856 -7.7288

50.6189 4.0657 -7.9261
57.879 9.9251 -9.8497
63.3425 11.3908 -8.6118
62.0214 17.5398 -10.8608
67.5983 19.6265 -4.7661
66.7658 10.2627 -7.1063
68.9242 14.6672 -9.3557
3.83 1.208 3.1115
3.9879 3.6542 2.0109
20.4228 12.0165 -2.9893
21.5775 11.6808 -3.5873
27.0028 10.6809 -3.4741
25.9243 9.8849 -4.6756
32.5546 19.1089 6.5351
39.8699 11.9462 -3.8015
41.4055 15.0008 -6.9583
34.363 21.3364 -7.842
46.0591 17.4936 -7.8676
45.4011 22.7508 -7.9272
53.8002 22.3187 -9.659
59.0344 24.5182 -8.5982
61.9881 26.1786 -7.424
64.9767 24.7216 -10.1459
4.344 1.1207 7.9531
5.0372 3.8308 7.6375
20.055 9.6514 8.4032
27.2117 13.7908 -2.7705
24.9081 15.1359 -4.4597
27.7296 7.3226 10.8565
50.5972 13.7573 12.4081
58.7797 15.2838 9.4786
58.7982 29.449 -3.5683
67.3409 17.7138 4.6132
65.3696 24.6491 -5.6977
4.6566 0.2284 7.836
6.6057 -1.2138 7.3095
22.5346 1.5271 8.4162
29.6613 1.4473 -3.4456
28.56 -0.4535 -5.0653
66.2046 4.7018 -3.9973
69.3291 12.4461 -5.3434
ID=PHAtriUNKCONKMMA42M162

LM3=54
4.11370000000054 0.101000000000325 0.841299999999945
6.51170000000156 -1.35459999999981 0.589999999999816
24.388400000001 0.313200000000179 -3.91479999999997
23.0037000000016 1.09960000000038 -3.4823000000001
25.5944000000014 7.49540000000031 -3.30440000000001
28.8295000000009 5.70320000000024 -3.17120000000002
27.3551000000012 5.77410000000026 -4.55049999999995
36.8013000000014 -0.0786999999997094 6.65230000000002
35.6078000000007 10.4120000000001 -8.18899999999994
39.9300000000011 9.90370000000029 -3.9676

42.5272000000011 9.20750000000024 -6.69139999999999
40.9621000000009 -0.877599999999876 -7.27259999999998
48.1929000000008 9.34740000000011 -7.32329999999995
48.9410000000007 4.4990000000001 -7.89239999999994
57.1822000000004 9.14850000000006 -8.03259999999995
62.0655000000008 11.8143000000002 -7.05169999999995
60.5266000000004 16.9384 -8.97499999999994
66.0253000000005 18.6792000000001 -2.15439999999994
66.4856000000007 10.4932000000001 -5.25159999999994
66.7485000000006 13.6497000000001 -7.74059999999994
3.48050000000053 2.34810000000028 1.083
3.88650000000058 4.25880000000026 0.776599999999993
20.587400000001 12.8794000000002 -3.54389999999998
20.1631000000011 11.7834000000003 -3.54429999999999
26.1688000000011 11.1852000000002 -3.21769999999998
24.6952000000011 9.89650000000025 -4.88099999999998
30.6929000000009 19.8148000000003 6.49280000000005
38.750900000001 12.8995000000002 -4.00659999999998
40.7947000000008 14.9999000000002 -6.93649999999997
34.4479000000008 22.5180000000002 -7.44329999999995
46.0986000000007 17.5696000000001 -7.73149999999995
44.7427000000006 22.7540000000001 -7.54799999999994
53.8088000000004 22.6222 -8.05529999999993
58.4716000000004 23.8504 -6.99079999999992
60.6527000000003 26.7892 -4.95999999999991
64.5228000000004 24.6425 -7.55269999999993
2.34680000000065 0.765500000000288 7.32900000000002
3.27880000000057 3.79860000000029 6.46410000000002
19.195600000001 9.93970000000028 8.7512
25.008100000001 13.2074000000003 -2.16279999999998
22.8299000000009 14.8664000000002 -3.98169999999997
26.8874000000011 8.0672000000003 10.8671
48.5317000000009 14.2416000000002 13.4214
57.2100000000008 16.3371000000002 11.1251000000001
57.2206000000004 29.9792000000001 -2.30229999999992
65.3147000000006 18.0595000000001 7.68460000000006
63.4800000000003 24.7456 -3.43219999999993
2.69590000000068 0.441800000000301 7.48340000000002
6.12440000000089 -0.619199999999719 7.28009999999999
21.3559000000012 1.97700000000028 8.50939999999999
28.3608000000009 2.8584000000002 -1.76709999999999
27.2607000000009 -0.0173999999998505 -4.32009999999997
63.9009000000008 4.65800000000015 -2.67229999999995
66.3776000000006 11.8371000000001 -2.47379999999994
ID=PHAtriUNKCONKMMA06M580

LM3=54
4.9789 0.6961 1.4614
5.775 0.304 0.4495
24.0209 0.9983 -4.7235
23.2884 1.5235 -4.6833
26.2668 8.4243 -4.7835
28.1419 5.521 -4.57
27.6402 5.7656 -5.1882

37.9061 1.0267 4.6492
36.2407 11.5819 -9.5501
41.8345 10.8574 -5.078
43.6436 9.4627 -7.537
41.0999 -0.2437 -8.4788
49.5107 9.4898 -8.2445
50.182 4.6321 -8.387
58.2476 10.0721 -8.5497
61.8369 11.6001 -7.5059
61.5774 17.9854 -9.7452
67.5255 19.4987 -1.9063
66.041 10.208 -5.3968
67.4353 14.6267 -8.1955
3.3856 3.7296 2.7968
3.7715 5.1404 1.7546
20.4223 14.0513 -4.1964
20.2324 12.7507 -4.297
25.6221 11.8885 -4.3957
25.4545 11.3373 -5.1934
32.6071 19.7324 5.4226
40.9099 12.9788 -5.2478
42.2556 15.5671 -7.6262
34.9975 23.948 -8.1494
47.6167 18.7629 -7.7333
45.8557 23.4587 -7.7507
54.8305 22.4199 -8.2693
58.5704 24.1019 -6.8377
62.3345 26.5901 -4.5358
65.2024 24.2439 -8.1694
5.6034 1.5032 7.5279
4.2528 3.9719 6.5186
22.185 10.2211 8.7699
24.7482 15.3845 -3.9075
23.1729 15.1254 -6.1866
31.2442 8.6842 10.3562
48.0841 13.7633 12.216
55.9366 15.7413 11.1117
56.6374 30.1808 -1.6299
65.2472 18.4062 6.6421
62.9002 26.8572 -2.8357
5.9663 1.0018 7.049
6.3244 -1.5274 5.4034
24.2426 1.9854 8.0148
28.6939 0.8394 -4.294
27.7315 -0.2842 -5.5211
62.9351 4.6513 -1.2673
67.4529 11.5784 -2.3264
ID=PHAtriUNKCONKMMA42M145

LM3=54
1.66889999999987 -2.89309999999961 -7.76390000000029
3.89049999999885 -3.24110000000027 -8.37600000000018
24.0475000000008 -1.32569999999944 -9.53210000000019
26.3794000000004 0.934400000000235 -9.4405000000002

25.2583000000002 7.61290000000018 -8.74730000000014
30.1112999999999 4.39710000000024 -7.96660000000009
28.4319999999999 4.30790000000005 -9.24930000000014
38.1757000000001 -0.906899999999771 4.4454999999998
37.1039000000007 11.1532000000004 -11.4048000000001
42.9321999999998 11.1018000000002 -6.08140000000012
45.1289000000003 9.26030000000029 -7.93770000000016
42.1749000000002 -2.39589999999967 -9.91410000000018
52.1091999999999 10.1001000000002 -8.10250000000015
56.6393000000003 4.59240000000032 -6.60730000000018
64.5958000000001 12.1558000000003 -6.73680000000023
67.8383000000004 12.4430000000005 -4.87100000000027
68.3168000000001 20.9631000000003 -5.42630000000027
71.2379000000004 22.0614000000004 4.13239999999968
71.2940000000005 10.1866000000005 -1.3065000000003
73.8612000000005 16.6649000000005 -4.09880000000031
0.276200000000014 1.28990000000028 -7.52550000000026
1.69169999999994 3.27720000000024 -8.14620000000027
18.4012000000003 13.7383000000002 -9.35030000000017
22.0441000000003 13.5634000000002 -9.30290000000016
26.7162000000003 12.8252000000002 -7.95250000000013
25.2154000000003 11.7338000000002 -9.19970000000014
29.9241000000003 22.3501000000002 4.48219999999981
41.3530000000001 15.4559000000002 -6.34530000000013
42.2389000000002 18.1425000000002 -8.39870000000014
32.8520000000004 26.0278000000002 -9.92970000000018
47.9236000000002 21.2604000000003 -8.61860000000017
49.4646000000003 28.0538000000003 -6.75630000000021
59.6656000000002 26.8904000000003 -6.90970000000024
63.3277000000002 27.9688000000004 -5.28390000000027
64.3388000000003 32.3722000000004 -1.6372000000003
69.9193000000003 27.3200000000004 -4.52560000000031
-0.10380000000007 0.0743000000002884 -0.107300000000263
-0.348999999999983 3.2051000000003 -0.390400000000274
15.0843000000001 9.21310000000022 4.9984999999998
25.1053000000004 16.5865000000002 -6.64450000000015
23.8808000000004 19.0813000000002 -9.47460000000016
23.5032000000001 7.19080000000023 8.0816999999998
47.0884000000002 15.6716000000003 14.9751999999998
55.8379000000003 17.6807000000003 14.7848999999997
59.1441000000003 35.2126000000004 2.65019999999971
64.9622000000004 21.1594000000004 13.1099999999997
67.1919000000003 31.0399000000004 1.51289999999969
0.696899999999907 -2.22569999999972 -0.152900000000269
2.15759999999974 -4.6267999999998 -0.99390000000024
18.1666000000001 0.37440000000023 4.6370999999998
30.348 0.967000000000263 -6.65040000000012
30.7921000000002 -1.96679999999968 -9.45430000000014
68.1542000000003 6.0474000000004 3.72219999999972
72.6855000000004 13.4905000000005 1.65069999999969
ID=PHAtriMALCAMMfNB19138*

LM3=54
5.38050000000003 0.921100000000077 0.333100000000055

7.68399999999989 0.000600000000065548 -0.345200000000001
23.9406999999997 0.628899999999964 -4.60679999999999
24.8334999999997 2.31339999999996 -5.09680000000001
25.7783999999997 8.58649999999997 -5.46439999999999
27.8436454131436 5.61891796640496 -4.63512999223634
26.7732021635926 5.80803803326035 -5.86906386958832
36.7698999999997 -0.0058000000011061 6.13880000000001
33.1825125238516 10.3885075749068 -9.66799098846609
38.9477999999996 10.1298999999998 -5.04859999999999
41.0552999999997 8.61099999999984 -8.39039999999998
38.2045999999995 -0.599000000000154 -7.27349999999997
45.5772999999997 9.49259999999981 -9.2555
47.4938999999996 2.8088999999998 -8.96699999999999
56.6327999999998 10.3747999999998 -10.1031
59.9568999999999 11.1796999999998 -10.9745000000001
58.7189 18.7133999999998 -11.9143000000001
65.993 21.2762999999998 -5.35530000000008
65.6378999999998 9.79339999999976 -8.26230000000005
65.8016 15.1413999999998 -12.4963000000001
4.0252999999999 3.37790000000005 1.14260000000001
4.83919999999982 4.99740000000005 0.396800000000005
18.8378999999999 13.804 -5.152
20.4417999999999 12.1213 -5.52430000000001
24.5545157391364 11.5152018629932 -4.9845035659364
23.6480447657254 10.9215097080148 -6.21927348515415
29.2463999999998 20.5860999999999 5.66280000000001
36.6124934982005 12.7254131755754 -5.42090948242159
37.8215999999997 16.1126999999999 -8.27899999999999
30.3952999999997 22.0499999999999 -8.03329999999999
42.2903999999997 18.5010999999998 -9.00080000000001
40.8395999999997 24.5424999999998 -9.0179
52.1746999999999 24.0149999999998 -10.7786
54.9663 25.6102999999998 -11.3325000000001
58.8108 30.0832999999998 -8.47270000000008
62.1857000000001 25.6604999999998 -12.4966000000001
4.60919999999981 2.58410000000005 7.86320000000003
3.71899999999986 4.41690000000001 6.80880000000003
18.0505999999998 10.6169 10.1647
23.1930999999999 14.4145 -3.26030000000001
20.9796999999999 16.4158 -4.83450000000001
24.9921999999996 8.5411999999999 11.8991
45.5710999999997 15.2206999999998 12.6422
54.6549999999998 17.5326999999998 10.0072
54.6534999999999 33.6944999999997 -4.10860000000004
62.5753999999999 20.8317999999998 5.09919999999994
61.1532 29.2829999999998 -6.80790000000007
4.69939999999981 1.53490000000003 7.64590000000002
5.40259999999982 -0.729499999999954 6.69280000000001
20.7998999999997 2.35189999999998 10.2556
27.8349999999996 2.05949999999994 -2.6349
27.5122999999997 0.104399999999975 -4.55400000000001
64.5547999999997 5.68719999999976 -3.88290000000002
67.1962999999998 13.4875999999998 -6.91900000000006
ID=PHAtriUNKCAMMfNB37810*

LM3=54
2.08179348983269 -2.53854811711118 -8.07899855287685
3.58387203023713 -3.08410295377342 -8.56376628370061
25.6412121321131 -0.938484056421867 -10.0671112923971
26.1309973037632 0.547379105911361 -10.0355896318477
26.0134000000002 7.67440000000001 -10.0691
31.0811999999996 4.34849999999982 -8.79530000000006
29.9100000000003 4.48679999999985 -10.2557
41.9820000000002 -1.33640000000001 3.69749999999997
36.9342999999995 11.1120999999999 -12.4487
44.4289999999999 12.0622000000001 -6.31400000000006
46.4902 9.7341000000001 -8.35060000000003
44.1280999999998 -0.766499999999958 -10.5421
52.4718999999996 10.4979000000001 -7.82379999999998
55.5909999999998 5.48880000000014 -7.37720000000001
63.2239999999997 12.4292000000002 -6.12010000000001
66.7585999999998 12.7333000000003 -5.24000000000004
67.5062999999997 21.2099000000003 -6.24010000000003
71.7436999999996 22.2846000000003 2.71989999999997
70.3595999999997 10.7859000000003 -1.62450000000002
74.2378999999997 16.3218000000003 -4.25690000000002
1.61449999999961 0.832799999999849 -7.6589
2.52399999999959 3.25129999999983 -8.18380000000001
20.0799 14.7082999999999 -10.1475
22.0841000000001 13.4829999999999 -10.0896000000001
28.2380000000001 13.3748999999999 -9.17710000000005
27.3829000000001 12.1613999999999 -10.7093
31.7866000000001 22.6195 3.07409999999994
43.1810999999999 15.2492000000001 -5.87740000000004
43.6268999999998 18.5833000000001 -7.96030000000003
35.7243999999997 25.3806 -9.63530000000004
48.5510999999997 21.3411000000001 -8.07390000000003
48.2288999999998 27.4945000000002 -7.31870000000005
59.0107999999996 26.2873000000002 -6.18110000000003
61.8306999999997 27.9004000000003 -5.48750000000004
64.3258999999995 31.7010000000002 -1.72630000000003
69.8020999999996 28.2120000000003 -4.47980000000004
-0.214600000000384 0.172799999999842 0.14260000000001
-1.41650000000037 2.49229999999985 -1.31060000000001
17.0777 10.9020999999999 4.41529999999996
25.0521000000001 17.6008 -6.42230000000006
23.3012 17.8877999999999 -8.91060000000004
24.5414000000001 7.278 8.71529999999996
46.9645000000001 14.9070000000002 15.3865
58.0461999999999 18.3063000000002 14.7332
59.0819999999996 35.3070000000003 2.96369999999996
66.2213999999997 21.2820000000003 12.1817
66.7634999999995 30.6770000000002 0.736999999999964
-0.555600000000397 -0.781900000000149 -0.280200000000011
0.366399999999612 -3.56840000000016 -1.95000000000001
20.2246999999999 0.227899999999897 3.53789999999998
30.5778999999997 0.207599999999894 -6.59140000000002
30.9238545917923 -2.22876264063802 -10.2317685319749

68.3575999999997 5.03390000000021 1.55849999999999
72.3446999999997 13.6576000000003 0.833099999999978
ID=PHAtriUNKCAMMfNB37813*

LM3=54
5.76579999999943 -0.00400000000042888 1.43669999999992
7.95370000000034 -0.960000000000484 1.51169999999996
27.4238999999998 0.107199999999394 -3.22409999999996
29.0489000000006 2.06039999999986 -4.05830000000004
27.7784000000004 8.07779999999977 -3.14970000000004
32.4957000000001 5.38029999999969 -3.67890000000001
31.2774000000008 5.23249999999983 -4.66250000000004
42.8002000000007 0.277499999999751 5.59679999999997
39.8322000000001 11.6800999999997 -9.46620000000001
44.2554000000004 11.0360999999998 -5.01230000000002
47.2727000000005 9.48509999999983 -7.82570000000005
43.8187000000004 -0.975000000000311 -8.13550000000002
53.4076000000007 9.73179999999983 -9.39040000000005
55.3568000000007 4.64839999999979 -9.32580000000005
63.9525302113075 11.0140229032076 -10.267762365861
67.2261000000009 11.8748999999998 -9.79430000000007
67.2883021182031 18.9582000117277 -11.86227316005
73.9145000000008 21.6627999999998 -5.24830000000008
71.9393000000007 10.0124999999997 -6.86670000000008
74.8982000000006 15.2204999999997 -10.4078000000001
4.90659999999999 2.77849999999964 1.86619999999997
5.88120000000005 5.00509999999962 1.94719999999998
23.2690000000003 14.8888999999997 -2.94200000000004
25.1370000000003 13.5617999999998 -3.56650000000004
30.3399000000003 12.8248999999998 -3.36350000000004
29.1237000000003 12.0772999999998 -4.49010000000004
35.2249000000003 21.9183999999998 5.49819999999995
43.4634000000003 14.6883999999998 -4.94100000000002
44.7210000000002 17.2787999999998 -7.5718
36.3250000000003 24.6982999999998 -7.99100000000003
49.8573000000003 20.0263999999998 -9.58210000000002
49.9557000000004 25.8979999999998 -9.66150000000005
59.4295846451753 23.5364378409889 -10.2861940441949
62.8380000000005 25.5366999999997 -9.66860000000005
66.0088000000006 30.4189999999997 -7.10990000000007
70.2929000000007 26.6537999999997 -10.8733000000001
5.10070000000003 1.8136999999996 7.77109999999998
3.70460000000004 4.78199999999961 7.26949999999997
22.9719000000002 11.8261999999997 10.2712
29.6663000000003 16.9057999999998 -2.40140000000005
28.0324000000003 18.9124999999998 -4.89680000000004
31.2019000000004 9.50879999999971 11.7046
53.3115000000007 15.9752999999998 12.3053999999999
60.6769000000008 17.7377999999998 10.5253999999999
61.6791000000006 33.2199999999997 -2.23200000000004
71.9061000000008 21.1715999999998 4.96159999999991
69.2270000000007 29.3384999999997 -5.32880000000007
5.14160000000002 1.01859999999958 7.70829999999998
5.63410000000005 -2.00630000000045 7.06719999999998

25.6916000000002 2.69919999999957 10.1855
33.6763999999999 1.69199999999955 -2.19469999999998
33.0938000000001 -0.659700000000456 -4.53489999999998
70.1957000000008 5.62279999999974 -2.07110000000006
74.5768000000008 13.3890999999998 -5.07230000000007
ID=PHAtriMALCAMMfNB38099*

LM3=54
0.81063699569778 -2.45502451157659 -6.47055851842607
2.22523278841766 -3.05786054771993 -6.63961687351128
23.9469999999986 -0.941600000000552 -7.77859999999988
25.7964999999985 1.51719999999916 -7.90089999999986
24.9276999999995 7.4978999999998 -8.63129999999996
29.5852999999998 4.93139999999979 -6.7858999999999
28.3834999999988 4.55089999999936 -8.13669999999988
37.791599999999 0.616799999999501 3.42720000000002
37.5538000000002 11.7962 -10.8157999999999
41.9449999999998 11.0140999999998 -6.53099999999995
45.5562999999999 9.25829999999978 -8.65539999999993
42.6464999999997 -0.951300000000297 -9.47469999999992
51.4648999999997 9.95099999999976 -8.65269999999995
53.4407999999994 4.58439999999962 -7.71879999999996
61.1180999999989 11.6928999999995 -6.44739999999996
65.3714999999988 11.9746999999994 -4.40499999999996
64.6938999999987 20.3049999999994 -4.80649999999997
69.0982999999984 21.3158999999993 3.26420000000006
69.5477327884393 11.3630749376304 -0.834048526747245
70.6538482788276 16.0290954661072 -3.56128985735038
-0.477707650729051 1.02547599612342 -6.80529576981273
0.115399999999509 2.42179999999963 -7.25069999999986
19.0220999999996 14.1267999999998 -8.50179999999991
21.5317999999997 13.5492999999998 -8.19849999999994
26.8853999999999 13.0835999999999 -6.95159999999996
26.0173999999998 11.6624999999999 -8.68409999999995
31.2956999999994 21.9414999999997 3.00500000000006
40.8596999999999 15.1862999999999 -6.36449999999997
42.5975999999999 18.2182999999998 -8.62029999999996
34.3652999999998 25.7825999999998 -8.94669999999994
47.4911999999995 21.3538999999997 -8.77109999999997
45.9717999999994 26.9545999999997 -7.74919999999996
56.950199999999 25.3796999999995 -6.94219999999996
61.2786999999988 27.0598999999994 -4.57679999999997
62.9802999999987 31.2803999999993 -1.89079999999995
67.8137999999986 26.2677999999994 -3.76629999999994
-0.0758000000007051 0.464399999999557 -0.00779999999984682
-1.61180000000063 2.92729999999958 -2.03929999999985
16.8495999999994 11.6986999999997 3.1713000000001
25.2039999999998 16.2182999999999 -6.26759999999994
23.0007999999998 17.6559999999999 -9.16739999999993
27.5006999999992 8.81929999999962 7.92660000000007
45.8905999999988 14.6823999999995 12.9586000000001
54.4104999999987 17.4332999999994 12.84
56.1037999999987 32.9488999999994 3.65870000000006
63.9872999999984 19.6081999999993 11.3219000000001

65.0317999999986 30.1521999999993 2.08250000000004
0.0361999999993039 -0.512100000000432 -0.0879999999998439
0.646499999999256 -4.13460000000045 -1.12129999999985
20.9467999999991 0.142299999999575 3.8899000000001
29.7062999999997 1.66619999999974 -5.56239999999988
29.0007999999995 -1.17380000000034 -8.09309999999989
64.7299999999987 5.14139999999937 3.15900000000005
70.2106999999985 14.1868999999993 2.05140000000005
ID=PHAtriUNKTOGMfNB9075**

LM3=54
5.98879999999967 -0.568599999999963 1.8800999999997
6.71569999999981 -0.810299999999404 1.51289999999982
26.8358999999995 0.201000000000181 -4.94210000000015
27.1610999999986 1.08229999999982 -4.80360000000005
27.1893999999995 7.52260000000011 -4.29920000000012
31.8928999999995 5.14770000000007 -4.62930000000012
30.2286999999996 4.87410000000005 -5.57120000000015
43.5129999999993 0.559500000000038 5.91989999999978
40.0790999999991 10.6727 -9.77120000000017
44.539299999999 10.8696999999999 -5.30650000000014
47.8427999999987 9.26719999999987 -7.74250000000013
45.2734999999988 -2.15160000000004 -8.07420000000013
54.2883999999986 8.51389999999988 -9.30430000000016
55.4646999999987 3.4956999999999 -8.5484000000002
64.3820999999987 10.0271999999999 -10.0929000000002
68.6105999999985 9.72939999999988 -9.41750000000025
68.9467999999984 17.4252999999998 -11.2789000000003
73.2821999999983 19.2828999999998 -4.16610000000027
72.4580999999984 8.77989999999984 -6.96440000000025
72.2551999999984 13.2169999999998 -11.2339000000003
4.07829999999899 3.54320000000016 1.51119999999984
4.95429999999891 4.36810000000015 1.31149999999988
23.3793999999991 13.6690000000001 -4.93670000000013
23.9178999999992 13.1360000000001 -4.73150000000013
30.0791999999994 12.0971000000001 -4.32750000000014
28.3072999999993 11.041 -5.92740000000013
39.016299999999 23.1752 4.68239999999981
44.132999999999 12.9239999999999 -5.21480000000016
46.257199999999 15.7267999999999 -7.66950000000017
38.2547999999989 25.0042 -8.39950000000017
51.2002999999988 19.0645999999999 -9.40040000000019
50.6537999999987 24.6309999999999 -8.5503000000002
59.9686999999985 22.8027999999998 -10.5702000000002
64.6506999999984 25.1367999999998 -9.65190000000023
68.6689999999983 27.3726999999998 -7.51020000000026
69.7966999999983 23.4911999999998 -11.4222000000003
4.67699999999901 1.04460000000019 8.17649999999986
4.35409999999886 4.75830000000012 6.52999999999985
23.7537999999991 12.7282000000001 8.67439999999984
29.6606999999992 15.0367 -3.90270000000014
27.1746999999991 17.2265 -5.71080000000014
34.2980999999993 9.47400000000007 10.9224999999998
55.5587999999989 13.9856999999999 11.3559999999998

63.8953999999986 16.5458999999999 8.33529999999974
64.4565999999983 30.8683999999998 -2.06690000000025
71.7997999999984 18.9407999999998 4.03899999999972
71.4144999999982 26.0041999999998 -6.09660000000026
4.99719999999904 1.06760000000021 8.27789999999983
6.27539999999927 -1.92039999999969 6.43539999999982
26.1994999999994 0.673600000000172 8.02599999999981
31.8931999999991 2.4342 -4.6792000000001
30.949099999999 -0.909299999999992 -6.2472000000001
70.8491999999985 4.15479999999988 -1.95020000000025
74.5634999999984 12.3515999999998 -5.92240000000027
ID=PHAtriMALTOGMNHN956724

LM3=54
6.67310000000038 -0.354399999999768 0.939700000000038
7.23370000000008 -0.386299999999696 0.796499999999961
24.8628000000005 0.923400000000321 -3.68670000000001
25.6725000000012 0.72390000000039 -4.26200000000012
27.0958000000003 7.41570000000017 -4.30079999999998
30.9440000000005 5.24470000000027 -3.95600000000007
29.9186000000001 5.46210000000021 -4.99640000000001
40.3235 0.458700000000254 5.57180000000006
39.5952000000007 10.8919000000002 -9.69630000000004
43.0464 10.6553000000001 -4.28049999999998
45.9123000000001 9.04030000000018 -7.16849999999996
43.3117 -0.881599999999736 -7.782
52.0694000000001 9.06510000000022 -8.14660000000001
52.9107 3.36480000000026 -7.6581
61.6025999999999 10.1921000000003 -8.54999999999999
66.3129999999997 10.5321000000002 -7.85469999999998
65.5815999999997 17.7201000000002 -10.5572
70.8707999999995 18.9554000000002 -3.40769999999995
69.9270999999997 8.93890000000026 -5.96919999999997
69.3854999999996 13.2394000000002 -10.1281
5.2060000000002 3.09890000000019 1.03530000000001
4.55850000000018 3.86510000000017 0.851500000000005
21.9961000000002 12.2215000000001 -3.50169999999999
23.2359000000003 11.9510000000001 -4.31829999999999
29.4966000000003 10.7909000000002 -3.891
28.5836000000003 10.4035000000001 -5.3871
36.2533 19.9441000000001 5.69430000000003
42.9006000000001 12.4389000000001 -4.78949999999997
44.4438000000001 15.0388000000002 -7.14939999999998
37.4305000000003 22.5209000000001 -7.50459999999997
50.4749000000002 17.8455000000002 -8.59879999999999
48.4718 23.3448000000001 -7.53459999999996
58.8406999999998 20.7501000000001 -8.44189999999993
62.8543999999997 23.9985000000001 -7.61569999999994
64.3350999999997 26.5067000000002 -6.18769999999995
66.2658999999996 22.4710000000002 -9.61129999999996
4.91960000000015 1.94650000000021 7.89769999999999
4.50560000000013 4.85180000000016 6.56040000000002
19.8892 11.9141000000001 7.57810000000003
26.1726000000003 13.6479000000001 -3.01399999999999

23.4316000000003 15.4593000000001 -4.68539999999999
29.8562 8.31990000000017 10.6292
50.6691999999997 13.3662000000002 12.6463
59.3263999999996 14.8717000000002 9.85100000000005
60.1797999999997 29.5573000000001 -0.723899999999926
67.0343999999996 17.9028000000002 5.04970000000006
64.7605999999997 25.4953000000002 -4.81549999999995
4.46440000000016 1.45890000000022 7.5638
6.76450000000011 -1.59739999999974 6.46399999999999
24.2846 0.0918000000002028 7.54800000000002
29.1809000000005 1.73930000000028 -2.92210000000005
27.3890000000005 -1.1361999999997 -4.66970000000005
66.1981999999997 4.48230000000029 -0.180999999999975
68.7406999999996 10.8681000000002 -4.06379999999997
ID=PHAtriFEMTOGMNHN956725

LM3=54
5.51040000000118 -0.170099999999864 0.968599999999953
5.06950000000067 -0.798500000000272 0.506299999999956
26.3467000000001 -0.714000000000324 -4.21210000000007
25.9776999999995 0.0836999999995498 -4.2656
27.3536000000007 6.95519999999982 -4.02140000000013
31.2714137771754 4.83366265912968 -3.89478446868428
30.1775137179122 4.9812910761877 -4.95389063377248
40.6278000000005 -0.64890000000021 5.81779999999993
36.8185999999999 10.1280999999997 -8.61040000000008
42.5885000000004 10.1507999999998 -4.27740000000009
45.6171000000001 8.41969999999971 -6.95360000000011
44.0351000000005 -2.07800000000023 -7.23640000000016
51.2309000000005 8.2163999999998 -7.97000000000018
53.4825000000007 4.21429999999981 -7.9243000000002
61.2653000000006 10.3383999999998 -7.77680000000022
66.3313000000007 10.8775999999998 -7.44790000000023
65.5602000000005 17.7074999999999 -9.82190000000023
69.9962000000006 19.0801999999999 -2.48960000000026
69.8149000000007 9.67249999999983 -5.55100000000025
68.8449000000006 13.7330999999998 -9.15360000000027
5.18380000000075 2.32629999999985 0.73259999999993
4.96280000000055 3.89149999999979 0.606599999999932
22.4689000000002 12.5302999999997 -3.92770000000007
22.9293000000002 12.0347999999997 -4.17850000000008
28.9484000000005 11.2220999999998 -4.05050000000011
26.9093000000004 10.1332999999998 -4.9302000000001
34.3971000000005 19.8227999999998 6.0141999999999
42.8074000000003 11.1030999999998 -3.9574000000001
43.6233000000002 15.2451999999998 -6.90570000000011
37.1011000000002 23.0093999999998 -7.64650000000012
48.6565000000003 18.4395999999998 -8.00300000000015
50.4443000000002 23.3275999999998 -8.22320000000017
57.9080000000004 21.5878999999999 -7.91790000000022
62.7859000000004 24.2532999999999 -7.52760000000023
65.2243000000004 26.6028999999999 -5.88990000000024
66.1477000000004 22.6714999999999 -9.20070000000025
4.80530000000074 1.23769999999982 8.02199999999992

4.01710000000064 3.5534999999998 6.91629999999993
20.9653000000002 11.3491999999997 8.54689999999996
28.3651000000003 13.4329999999998 -2.49690000000009
25.9400000000002 15.2076999999998 -4.55260000000009
32.6581000000005 8.35999999999976 11.3222999999999
52.5056000000007 13.2068999999998 12.4577999999998
60.4035000000007 15.7881999999998 10.5371999999998
60.0527000000004 30.6921999999999 -2.49400000000022
67.2385000000006 17.6686999999999 7.16959999999975
66.7792000000004 25.2511999999999 -2.59670000000025
4.97650000000076 0.0887999999998449 8.03089999999989
5.63460000000075 -2.42220000000018 6.74699999999993
24.9244000000003 -0.677400000000317 8.36969999999996
29.6810000000005 0.664899999999781 -2.52040000000013
28.9215000000004 -2.27240000000026 -4.37380000000012
66.3008000000007 2.90349999999981 -1.58050000000023
70.0087000000007 10.8670999999998 -2.96460000000024
ID=PHAtriMALTOGMNHN956728

LM3=54
5.67939999999997 -0.793600000000227 1.67650000000009
6.21389999999995 -0.449900000000448 1.11960000000005
25.4424 -0.184800000000235 -3.61299999999994
25.5545 0.459099999999801 -4.04919999999995
26.8584000000001 7.29539999999986 -3.79919999999997
29.2391 4.62419999999985 -3.61779999999995
27.6449 4.50199999999984 -4.71579999999994
39.9565999999998 0.164199999999773 6.72600000000014
36.1660999999999 9.53029999999988 -8.94359999999989
42.4631999999998 10.0354999999998 -4.81959999999987
44.2180999999998 8.27609999999986 -7.26049999999985
41.0047999999997 -1.8405000000002 -6.78899999999984
49.5319999999996 8.14039999999981 -8.3228999999998
51.3878999999995 4.57329999999979 -7.16499999999977
59.9017999999994 9.33049999999976 -8.78059999999971
63.6453999999992 10.4028999999997 -8.90649999999967
62.400099999999 16.5357999999996 -10.7438999999996
68.4581999999989 18.1331999999996 -3.80119999999961
67.196699999999 8.04079999999966 -6.86599999999963
66.565199999999 12.8584999999996 -10.8391999999996
4.44879999999999 3.58059999999952 1.81100000000004
4.75750000000007 4.03119999999964 1.22260000000002
21.8632 12.3940999999998 -3.26499999999996
22.4250000000001 12.0921999999998 -3.33819999999996
27.9117000000001 10.3988999999999 -3.73379999999996
26.3503000000001 9.71429999999988 -5.15619999999996
35.0319999999998 19.3378999999997 6.57850000000012
41.9249999999998 11.0341999999998 -4.55699999999987
42.6824999999997 14.4177999999999 -7.50949999999985
34.6596999999998 22.0382999999998 -6.25169999999986
47.0196999999996 16.9876999999998 -8.61769999999981
47.5571999999995 22.2351999999998 -7.43189999999977
55.1966999999992 20.6627999999997 -9.39279999999971
59.8824999999991 23.0246999999997 -8.75609999999965

62.163499999999 25.7561999999996 -6.81119999999962
63.4519999999989 21.2773999999996 -10.5536999999996
5.64639999999999 1.42389999999952 8.40410000000003
3.57689999999997 3.82389999999957 6.61400000000004
21.7862 11.5860999999997 8.63390000000005
29.2998 13.1463999999999 -1.32419999999995
24.9646 15.0530999999998 -4.54829999999994
34.5603999999998 8.5599999999997 11.3598000000001
52.5527999999995 13.1697999999997 11.5649000000002
60.5750999999994 14.8658999999997 8.95100000000027
59.9547999999991 28.7559999999996 -2.27699999999966
66.9577999999992 16.9428999999996 4.03340000000035
65.437499999999 24.3394999999996 -4.74809999999961
5.59879999999998 0.873999999999536 8.20460000000005
6.13379999999999 -2.09390000000044 6.66430000000005
25.2476 -0.10830000000029 8.31710000000004
31.5528 2.7606999999998 -0.918399999999928
28.9243 -1.16180000000019 -4.36349999999992
66.1285999999992 4.33459999999968 -2.58189999999967
68.574399999999 10.8217999999996 -4.91039999999963
ID=PHAtriFEMTOGMNHN956729

LM3=54
6.5577 0.0448 1.3797
7.2344 -0.321 1.3584
28.7918 0.7371 -5.8745
28.2895 1.2053 -5.533
29.6032 8.2559 -4.8708
33.0548 5.227 -4.3253
31.6867 5.4331 -5.748
42.0406 0.3097 5.8147
39.3748 11.2292 -9.6376
45.3074 11.0738 -5.0511
49.2966 9.572 -8.2884
46.3959 -0.729 -10.1948
56.3645 9.058 -9.005
56.8183 4.0925 -8.9855
65.6889 10.5172 -8.6533
70.7297 11.7439 -8.4278
68.6706 19.1392 -10.9012
75.7607 20.611 -1.9492
75.2878 9.9055 -6.1538
73.8742 14.4012 -10.3552
5.2788 4.4126 1.47
6.7062 5.5745 0.9681
25.1617 14.5897 -5.5385
25.2856 15.2665 -5.4772
30.8251 12.5655 -4.6516
29.4578 12.1669 -5.944
38.1848 21.298 5.3439
44.5898 13.4499 -5.0687
47.2655 16.8851 -7.66
39.7452 24.7202 -9.036
53.645 20.0858 -8.2873

50.8503 24.8925 -8.4956
61.611 24.1836 -9.0681
66.6701 25.7965 -8.3805
69.4784 29.0891 -6.1753
71.6494 25.6695 -10.4129
5.973 2.2261 8.8821
8.0919 6.3401 7.6976
24.4456 11.0529 9.4202
29.2792 18.12 0.8327
28.4788 16.858 -6.167
31.0004 7.773 11.6823
55.5452 14.5503 13.0476
64.3147 16.775 11.5834
66.2732 32.0744 -1.4327
72.4517 18.7941 7.3749
71.2521 27.7536 -3.8681
5.6392 2.0964 8.6958
8.5485 -0.8225 7.6298
25.9574 2.6537 9.0612
33.0449 1.7418 -3.9876
31.8925 0.6706 -6.6499
72.2747 5.011 -0.9864
76.2502 14.3417 -4.0517
ID=PHAtriFEMGABMNHN199048

LM3=54
8.0485 0.4745 1.1024
9.2971 -1.0182 0.1586
30.7356 0.5039 -5.0884
32.7902 2.0062 -5.5971
32.5371 8.5214 -5.2064
36.8782 5.2851 -4.9768
35.7224 5.3949 -5.7164
45.5278 -0.9733 6.1412
43.4094 11.6117 -10.1762
49.8744 11.7998 -4.9608
52.1318 9.5106 -7.7466
48.485 -1.5921 -9.8267
59.2205 10.387 -8.8045
60.1572 3.8682 -9.2892
70.5047 11.7197 -8.6501
74.4545 11.9819 -8.1583
75.0763 20.2943 -10.496
80.9692 21.8682 -1.7593
80.1822 10.1422 -5.1739
81.7976 16.1524 -8.2403
7.1752 3.4519 1.3086
7.1415 5.4907 0.1726
26.8599 15.2506 -4.8204
29.5043 14.1549 -5.0269
34.4151 13.5097 -4.2093
33.9484 13.0425 -5.9093
38.2537 22.7013 6.7527
49.2218 14.8707 -4.89

49.626 17.995 -7.611
40.7534 25.4809 -9.6525
55.7935 20.7054 -8.992
53.7594 26.9164 -9.0244
65.6339 24.9993 -9.3867
69.9425 27.2189 -7.8431
72.8789 31.7423 -5.0623
78.3544 27.5953 -8.2648
6.1024 1.7861 9.0187
5.6262 5.4545 7.9361
26.9205 11.9454 10.4204
33.6404 17.5714 -2.4698
32.2076 19.221 -6.1167
32.3602 8.2848 12.22
58.4299 15.4835 14.0501
69.0936 18.3872 11.8451
67.5951 34.9741 -1.1163
77.5322 21.0466 7.7316
75.0252 31.0576 -3.2871
6.1175 0.7409 9.0357
7.4755 -2.8011 7.7286
29.6524 2.3181 10.1814
38.1943 0.5746 -3.2731
38.0185 -1.0556 -6.2651
76.6505 4.66 -1.4142
81.5507 13.75 -3.0965
ID=PHAtriMALCAMAMNH241130

LM3=54
1.05886844120078 -0.65852738945985 -5.72939827396029
2.22610000000311 -1.06189999999944 -6.02190000000003
19.7125000000021 0.0684000000004409 -7.97610000000012
21.2257000000019 1.58190000000034 -8.18730000000012
19.9924000000016 6.97940000000033 -7.23290000000011
25.4235220785894 5.53966513092853 -7.14633466289277
24.4988244493915 5.600371742264 -8.11587866636763
34.3977000000015 0.329900000000184 3.11859999999999
32.2794065800947 11.1979665758501 -10.4158159101884
35.3400000000009 11.0992000000001 -6.20319999999998
37.3819000000009 9.18640000000015 -8.15449999999996
35.5977000000015 0.0475000000002448 -9.26830000000002
42.6790000000009 9.29490000000008 -8.54119999999991
45.6293000000009 4.53769999999996 -8.61249999999984
53.0660000000007 10.5778 -7.50519999999981
57.5379000000007 11.6311999999999 -6.78079999999976
56.9773000000008 18.5936 -8.2683999999998
61.5487000000008 20.3953999999999 -1.19699999999975
62.0428000000009 10.1060999999999 -4.29649999999976
62.2211000000008 14.4280999999999 -7.88289999999973
0.459566389017028 2.48912103420878 -5.60347551362395
1.29303407010778 4.01474109730633 -5.89310634574895
15.8774000000018 11.8654000000003 -7.72030000000012
17.8732000000016 11.8992000000003 -8.2021000000001
23.2968761305124 11.4149897621666 -6.98377471993089

22.5756342521611 10.778606823824 -8.05949868513622
26.7656000000013 20.6331000000003 3.62939999999996
34.4017000000008 13.6498000000001 -6.06089999999998
35.6296000000009 15.8297000000002 -7.71939999999996
28.5458000000013 22.2283000000003 -8.61500000000001
40.0450000000008 18.6963000000001 -8.46189999999992
39.8927000000008 24.0913000000001 -8.56529999999989
49.0281000000008 23.4457000000001 -7.54229999999984
52.7544000000007 24.8324 -6.68399999999979
55.6189000000008 28.5752 -4.39019999999977
58.6754000000008 25.4003999999999 -7.45309999999975
-0.293699999996867 0.325300000000651 -0.400300000000224
-0.399099999997028 2.66160000000056 -0.439900000000183
18.752200000002 16.1711000000003 -1.21710000000011
21.0776000000015 13.4656000000003 -6.51790000000012
19.6158000000016 14.8313000000003 -8.84840000000012
21.5471000000016 7.65630000000022 7.55389999999995
42.8002000000011 14.5080000000001 12.9953000000001
49.6996000000009 15.9542 11.6284000000002
50.4386000000009 31.1113000000001 -0.0993999999998181
59.850700000001 19.2121 7.01270000000023
57.6392000000008 26.8677 -2.01719999999976
0.165100000003005 -0.821399999999477 -0.178600000000185
0.891900000003153 -2.7231999999994 -0.821300000000202
15.9132000000021 -0.288599999999654 3.8877999999999
24.8118000000017 1.65740000000027 -5.9892000000001
24.2678000000018 -0.69519999999972 -8.3859000000001
58.7442000000011 5.09910000000005 0.95290000000018
62.5161000000008 12.1667999999999 -1.77909999999973
ID=PHAtriMALNIGUSNM379617

LM3=54
1.63035115050691 -2.11872282018153 -7.54017442746612
3.24939999999727 -2.94430000000042 -8.08539999999961
25.3456999999985 -0.965800000000086 -8.69119999999973
26.8536999999984 0.0219999999998619 -9.01119999999966
25.1347999999987 7.4621 -8.12519999999973
31.0296715381676 5.0810039925992 -7.71605224811378
29.8239719694341 5.38274741091307 -9.00354082424222
39.6718999999985 -0.0836000000002742 5.28910000000033
40.1802264233047 11.5729759413567 -10.8726951712227
44.301599999999 10.9485999999999 -5.58919999999977
47.4251999999992 9.81599999999982 -7.88029999999978
43.9349999999987 -2.33340000000027 -8.3974999999997
53.4000999999992 9.76989999999974 -7.73519999999974
55.669399999999 4.27869999999962 -6.81749999999968
64.6013999999991 11.9996999999995 -5.19259999999967
69.082899999999 12.4623999999995 -3.66709999999964
69.417599999999 20.5429999999995 -4.96659999999963
72.1790999999986 22.0453999999994 2.76770000000045
73.5583999999987 10.8629999999994 -0.239699999999574
76.0594999999987 15.4405999999994 -3.28529999999958
0.195499999997495 1.89359999999974 -7.13259999999956
0.876499999997655 3.12009999999976 -7.4053999999996

20.6050999999985 13.5397000000001 -8.98439999999972
22.3959999999985 13.1366 -8.84469999999972
28.4271105667643 12.4105230508305 -7.79304215166591
27.4307838958359 11.8003593609268 -9.00292208708881
34.1370999999987 20.8046999999999 4.25630000000028
43.3343999999992 13.8237999999999 -5.38429999999977
45.5814999999992 17.2017999999999 -7.35809999999978
35.4529999999989 25.5038 -8.27919999999974
50.1126999999993 20.3519999999999 -7.25399999999976
49.3872999999992 25.3972999999999 -6.95029999999974
60.1858999999992 25.3354999999997 -6.3627999999997
64.4123999999991 27.7811999999996 -3.64579999999967
66.4365999999987 31.3501999999995 -0.825899999999609
70.9537999999988 28.5302999999994 -3.96549999999958
-0.0512000000026627 0.184499999999632 -0.0571999999995513
-1.2682000000026 2.66659999999971 -1.07699999999954
17.5489999999981 9.90329999999986 4.22470000000034
26.6974999999987 15.5992000000001 -7.36349999999975
25.6415999999986 17.6082000000001 -9.54169999999973
29.0849999999984 8.22459999999983 9.33090000000033
49.3184999999986 14.6772999999997 14.2905000000003
58.0389999999985 17.5920999999995 13.9689000000004
59.6119999999987 33.7336999999996 3.95900000000039
66.8639999999985 20.1238999999994 11.3879000000004
67.5474999999987 30.8338999999994 1.45840000000041
-0.145700000002735 -0.515500000000363 0.033400000000453
0.405199999997431 -3.92960000000036 -1.22539999999955
20.2307999999981 0.443999999999785 4.34580000000037
30.9786999999985 1.17489999999984 -7.32759999999969
31.1668999999985 -1.09030000000017 -10.1305999999997
69.1844999999986 4.79429999999939 3.58930000000041
73.7487999999986 12.4326999999993 2.00280000000043
ID=PHAtriFEMBENUSNM439127

LM3=54
5.3623 -0.6454 0.7262
6.752 -1.3138 0.8785
27.3734 0.1361 -5.9689
29.2504 1.4526 -5.904
28.2193 8.4013 -5.8119
32.5812 5.9002 -5.635
31.8765 5.2775 -6.5076
41.065 0.7989 5.2715
38.3372 11.6748 -10.438
42.2007 11.1932 -5.2912
45.2988 9.8238 -8.3927
43.1752 -1.3082 -8.5769
50.2538 10.4029 -9.328
53.3572 4.5438 -9.2839
61.8064 10.9469 -9.6044
65.6221 12.4895 -9.157
65.3478 18.9216 -10.8165
70.6824 21.1636 -2.6274
70.5146 10.1891 -6.0279

71.2192 14.6797 -9.9401
4.0012 3.3843 0.7473
4.6185 4.9236 0.364
22.7914 15.3552 -6.2337
25.1137 14.36 -6.3755
30.3131 13.2518 -5.702
29.3415 13.2734 -6.5484
34.7369 22.1889 4.7783
40.8047 14.5097 -5.3741
42.8957 17.0087 -8.1646
35.3043 24.404 -9.5632
47.4533 19.481 -9.7603
47.4938 25.3433 -9.3243
57.4578 24.1223 -9.4699
61.7946 25.8072 -9.1025
64.0049 29.2741 -7.1483
67.6393 24.6349 -10.1757
6.4603 2.1432 8.0952
5.0361 4.514 6.9291
22.6799 11.8344 8.0115
28.4261 15.9703 -4.0783
27.6413 18.8333 -7.7563
32.5067 9.3493 10.5332
49.8603 14.6645 12.4475
57.2819 16.6706 11.2753
60.0862 33.1268 -1.836
69.9997 20.5369 3.9387
66.9865 27.7216 -4.2322
6.1936 1.6221 7.9921
6.5408 -1.6957 6.7975
25.9225 2.4406 8.6834
32.8038 2.2191 -4.1804
33.4087 -0.5419 -8.2268
67.9763 4.0854 -2.2923
71.1767 12.8559 -4.1516
ID=PHAtriMALCIVUSNM450073

LM3=54
6.288 -0.246 0.6848
7.7026 -0.9997 -0.0645
25.9451 0.1864 -5.2804
25.6631 1.5026 -4.848
26.4218 7.9291 -5.9675
30.4926 5.1527 -4.706
29.0506 4.5172 -5.6103
39.1239 0.1343 5.4859
37.417 11.3675 -10.1942
41.1289 10.45 -4.509
44.2053 9.1423 -7.4877
40.7543 -0.6659 -8.7858
49.4821 9.3273 -8.6013
51.6289 3.9782 -7.9185
59.6865 10.4723 -8.4014
63.3433 11.7183 -7.9607

61.1179 18.0976 -9.554
69.3228 20.5302 -2.9927
68.1157 10.0492 -4.8602
69.2743 15.0674 -8.1194
5.594 3.809 0.7095
5.6911 5.4434 -0.0173
21.7296 14.3017 -5.5518
22.6536 13.6477 -5.2957
28.0308 12.4478 -4.511
26.7338 12.3517 -5.5001
32.1945 21.9578 5.0644
39.7274 14.1753 -4.5491
42.0336 16.4334 -7.4539
33.7769 22.5391 -9.2632
46.2035 19.0543 -9.1474
45.1875 24.9281 -8.5637
55.7327 23.556 -8.9474
59.2563 25.1543 -8.3738
62.8435 28.9646 -5.5802
65.3713 24.7501 -9.1424
6.1807 2.0516 7.6511
5.303 4.9078 6.3508
19.6644 11.1149 8.1962
26.0655 16.6508 -2.1322
25.452 17.8018 -6.295
29.8298 8.8137 11.3555
48.5178 14.9134 13.1308
55.6961 16.9217 11.3447
57.7099 32.3239 -0.6876
67.8874 20.5891 4.95
64.9824 27.2762 -2.882
5.7493 1.5343 7.2211
6.9954 -1.8712 6.361
22.1889 1.7469 8.4796
30.8934 0.3896 -2.6317
31.0374 -1.2329 -6.3757
65.8334 4.5244 -0.3963
69.0864 13.447 -2.5153
ID=PHAtriFEMCIVUSNM465920

LM3=54
4.5195 -0.3314 0.0726
5.7488 -0.992 0.4262
24.2023 -0.3367 -5.4988
24.4875 1.3661 -5.7249
24.7299 7.6368 -6.5053
29.6703 4.5776 -5.7221
28.2491 4.3927 -6.9348
37.5491 0.1187 4.4525
34.5205 10.8631 -10.4541
39.2373 10.7032 -6.5758
42.8749 9.2557 -8.8492
40.8466 -1.0347 -9.4482
47.1852 9.2953 -9.8323

50.1908 4.2216 -9.9051
59.1143 10.6769 -10.1436
62.0955 12.2011 -9.693
61.151 18.7442 -11.5712
67.4963 20.7677 -2.8051
67.0337 10.4738 -7.5002
66.8447 15.5739 -11.705
3.3701 2.8277 0.0061
4.16 3.9354 0.0054
19.761 13.6188 -5.5984
20.883 12.5672 -5.9531
26.9395 12.2174 -5.2783
26.0615 11.8033 -7.0817
31.5056 20.5202 3.8596
38.5853 13.158 -6.4678
41.4439 15.3581 -8.2954
33.2352 23.5472 -9.3071
44.1929 18.2255 -9.2908
43.9746 24.6513 -9.2029
54.597 24.5319 -9.8354
58.0307 25.1675 -9.4979
61.184 28.6187 -8.035
63.8688 24.3763 -11.3916
3.4201 1.4543 6.8608
3.0059 4.329 6.1631
19.2358 11.3401 7.9858
24.9369 15.6392 -3.2901
23.1486 16.5376 -6.3188
30.2559 9.1547 10.6544
47.549 14.4302 11.8467
55.6512 16.9682 10.4719
55.8275 32.8754 -5.414
67.1036 21.1497 4.0238
63.3078 27.6905 -5.9891
3.4759 1.158 7.0403
4.9392 -1.6759 6.2539
22.3758 1.1023 8.1719
29.6367 1.1825 -2.6085
28.3224 -0.6073 -6.5363
64.3758 4.4191 -6.0764
67.5659 12.8073 -5.7303
ID=PHAtriFEMCIVUSNM465923

LM3=54
5.6953 -0.508 0.7781
6.9698 -1.5406 0.125
24.4334 -0.6829 -4.4499
26.4555 0.7684 -5.4024
25.8106 7.431 -5.3653
31.1575 4.6052 -5.0143
29.9927 4.4865 -6.2956
37.858 0.1444 6.1233
36.5276 11.4209 -10.7869
41.0855 10.8483 -5.6512

44.014 8.8972 -8.2214
42.048 -2.1937 -9.477
49.1936 9.3347 -9.7277
51.8936 4.0107 -9.0918
59.0654 10.3468 -9.735
62.8363 11.5169 -9.5271
62.6604 18.8797 -11.4394
68.8689 20.8776 -4.8718
67.736 10.5819 -6.7592
68.7945 15.1802 -10.2568
4.4491 3.1243 0.4694
5.0656 4.6198 0.0886
20.4606 13.8676 -4.4263
22.0937 13.5731 -5.1588
27.9506 12.7973 -4.8971
27.1462 12.3809 -6.3197
32.2579 21.4465 5.0619
40.0803 13.8016 -5.6514
41.522 16.9651 -8.3499
33.7992 25.018 -9.8851
45.9534 18.9667 -9.4154
44.9223 25.1327 -9.064
54.7027 23.7585 -9.4199
59.4384 25.3835 -8.8172
61.9764 28.4539 -6.4888
65.2688 24.6585 -11.1694
5.1175 1.2044 7.7358
4.0397 4.3649 7.0213
19.7025 12.6111 8.9273
25.7304 15.4876 -2.6389
23.5734 17.5244 -5.6996
29.7751 8.7986 11.2975
50.0958 15.0536 12.5536
56.0847 16.7096 11.1124
59.0436 31.916 -2.637
67.7395 20.5671 2.6362
65.1593 26.9294 -4.0895
5.4079 0.791 7.6269
6.168 -1.9468 6.8304
23.4331 0.4915 8.6313
30.1943 1.615 -1.9113
29.8955 -1.6047 -5.7584
66.5612 5.6288 -3.0046
69.063 13.339 -4.3815
ID=PHAtriMALCIVUSNM465924

LM3=54
4.6353 -0.5622 1.1046
6.5916 -1.5775 0.5724
24.3772 -0.1695 -5.6508
26.5259 0.9002 -6.4552
24.435 7.2532 -5.3406
30.3363 4.9517 -5.7731
28.902 4.4894 -6.4474

38.4365 0.7307 4.9732
36.8534 10.642 -10.6443
40.7466 11.1888 -4.8823
43.3189 9.6343 -7.8225
39.9098 -1.1767 -8.8796
48.5477 9.4966 -9.5415
50.2296 4.3981 -8.9935
60.1961 10.1564 -10.294
63.267 11.682 -9.7897
62.7333 18.2196 -12.0525
68.4025 20.3121 -3.7283
67.3184 8.4932 -7.6341
68.996 14.3167 -11.0601
3.7871 2.6661 1.091
4.5272 4.4924 -0.0766
20.4862 13.4119 -5.5216
23.2121 13.1853 -6.2541
28.1969 12.1331 -5.4652
26.9827 11.646 -6.3546
33.1825 20.8643 4.1929
40.1165 13.031 -5.3759
41.2052 16.1857 -7.9859
33.0611 22.5377 -9.4664
46.11 18.5758 -9.4571
44.8397 23.7119 -9.3648
55.7969 23.7282 -9.9152
58.835 24.5822 -9.9261
61.4943 29.0501 -8.3978
64.9003 24.7359 -11.2776
5.2983 2.1692 6.9583
3.9806 4.6786 5.975
20.2234 11.7405 7.9735
26.084 15.3591 -3.4775
25.4847 17.5452 -8.2165
30.1951 8.6449 10.2263
48.9683 14.6746 11.9192
57.0857 16.4242 9.058
57.4905 31.7836 -3.0669
68.0281 19.9166 1.8194
63.6596 27.2822 -6.0779
5.5997 1.449 7.1757
5.703 -1.4427 6.2338
22.8229 1.7759 8.3765
29.7563 1.1057 -3.2371
30.7957 -1.1037 -7.6216
65.1081 4.2097 -2.7556
68.4291 11.9829 -5.5136
ID=PHAtriFEMCIVUSNM465926

LM3=54
6.147299999999 -0.232400000000446 0.189600000000197
7.40810000000031 -0.990199999999914 0.206699999999964
25.7511999999996 0.0808999999998709 -4.57559999999989
26.6565999999997 1.99449999999973 -5.35289999999993

25.3921000000001 7.80019999999994 -4.24219999999997
31.0717000000003 5.61220000000007 -5.07030000000007
29.337200000001 5.03100000000022 -5.70620000000012
40.0688999999998 1.08299999999998 5.53559999999998
36.2982 10.7641000000001 -9.79390000000005
40.9483999999998 10.3778 -5.48910000000005
43.7822999999996 8.48049999999995 -8.80290000000006
40.3735999999998 -1.3268 -8.91580000000003
48.9940999999999 8.63120000000012 -10.4096000000001
50.9636000000001 4.35250000000017 -10.7707000000002
58.3240000000001 10.8205000000002 -10.5814000000002
62.1168000000003 12.3495000000003 -10.0507000000003
60.9060000000002 19.4162000000002 -11.9335000000003
67.0713000000002 21.5993000000003 -4.94560000000033
66.5385000000004 10.0171000000003 -8.61990000000033
67.0784000000003 15.3416000000003 -11.8407000000003
4.8309999999998 3.4988999999998 0.142000000000037
5.65879999999985 4.60319999999981 0.00470000000000925
20.9150000000001 13.6767 -4.7228
23.0652000000001 12.8327999999999 -4.89019999999999
28.4281 11.9861999999999 -4.9605
26.8355000000001 11.6748999999999 -5.85780000000001
33.9814999999999 21.8513 5.07629999999992
40.0932999999997 14.397 -5.71060000000007
40.8919999999997 16.7642 -8.83870000000007
32.2554 23.4093000000001 -8.9286000000001
44.8038999999999 18.5398000000001 -9.66100000000014
44.5049 24.4795000000001 -10.0256000000002
53.9343000000001 23.5944000000002 -10.9104000000002
58.4509000000002 25.6462000000002 -9.03600000000028
59.8705000000002 29.9498000000003 -7.19460000000032
63.3925000000003 26.0707000000003 -11.2310000000004
5.68879999999981 1.57579999999982 7.11809999999998
4.93059999999982 4.59769999999981 6.4779
22.1412999999998 12.7174999999999 7.77650000000001
27.6029 15.2848999999999 -2.00190000000002
25.5485000000001 17.6493999999999 -6.06040000000003
32.0019999999998 9.61249999999991 10.9644
50.5341999999999 15.4383000000001 12.6941999999998
56.0677 17.1442000000001 10.9451999999998
56.3744000000001 32.5527000000002 -3.41050000000031
66.2716000000002 20.7270000000003 3.99209999999997
61.4908000000002 28.6191000000003 -6.1616000000003
6.3505999999998 1.45709999999983 7.6579
6.93039999999981 -1.29250000000015 6.88180000000001
25.4788999999997 1.83589999999987 8.01730000000005
30.7331000000001 1.79059999999998 -1.20829999999996
31.2786 -0.412100000000038 -5.96449999999995
65.3694000000003 5.66800000000025 -3.51040000000029
67.4792000000004 13.5045000000003 -6.41640000000031
ID=PHAtriFEMGHAUSNM465927

LM3=54
4.39949999999884 -0.466300000000239 1.65629999999984

6.35699999999882 -1.36600000000026 1.64059999999982
25.4295999999993 0.618099999999942 0.554699999999952
27.2631999999993 1.77719999999994 0.68719999999993
25.8289999999991 7.89569999999993 0.741499999999917
30.2186999999992 5.31309999999994 2.41199999999994
28.9759999999991 5.20049999999992 1.09709999999994
37.4860999999994 -0.0261000000001024 14.3493
37.3711999999993 11.8670999999999 -0.350400000000033
40.7529999999994 10.5257999999999 5.2529
44.3121999999994 9.18269999999988 3.25859999999998
41.4880999999994 -0.0875000000000858 0.152299999999983
48.4584999999994 10.3146999999998 3.61869999999997
49.2580999999993 4.18399999999978 3.12019999999997
59.3503999999993 10.9396999999997 5.06019999999995
62.4184999999992 12.6680999999996 6.4753999999999
62.7453999999991 18.7678999999995 5.15929999999988
66.554299999999 20.5129999999995 12.6255999999998
66.368199999999 11.5066999999995 10.0829999999999
67.6562999999988 15.8220999999994 7.35559999999982
3.76599999999889 2.69029999999974 1.47479999999982
4.8890999999989 4.02909999999973 1.07129999999982
20.6174999999991 13.1577999999999 0.476999999999897
23.8002999999991 12.8430999999999 0.728699999999916
28.3032999999992 12.8026999999999 2.04299999999994
26.5829999999992 11.9692999999999 0.90129999999992
30.2037999999992 20.5808999999999 13.7321999999999
39.1409999999994 14.4931999999999 4.9473999999999
41.5752999999994 17.9213999999999 2.9258999999999
34.1174999999993 23.4374999999999 -0.102300000000074
45.5862999999994 19.5369999999998 2.35239999999996
44.8620999999993 23.8335999999998 2.66779999999994
55.0984999999993 24.4208999999996 4.55739999999991
58.5558999999992 25.1302999999996 6.33659999999989
61.056599999999 27.7442999999994 9.56339999999984
64.613199999999 25.0303999999994 6.99249999999984
4.39509999999898 1.43759999999973 8.45249999999981
4.03449999999893 4.35639999999974 7.54429999999982
19.5934999999993 12.2749999999999 13.0828999999999
27.0893999999992 15.609 3.53479999999992
25.9265999999992 17.1917999999999 0.00819999999991394
28.0160999999994 8.77409999999992 17.6414
44.1100999999993 14.0771999999997 23.5438
51.4413999999992 15.8817999999996 22.9745999999999
55.968799999999 31.7693999999995 11.7928999999999
63.4268999999991 20.3653999999995 19.0226999999999
62.791399999999 27.5322999999994 9.95709999999985
4.46499999999897 1.12889999999974 8.40489999999982
5.61119999999895 -1.48280000000029 7.40959999999981
22.8364999999994 0.808399999999909 13.3052
30.9995999999993 2.08209999999995 3.54669999999997
31.4498999999992 0.229299999999908 -0.341400000000054
63.6724999999991 5.93689999999958 12.8178999999999
67.4959999999989 13.4923999999995 10.4856999999998
ID=PHAtriFEMGHAUSNM465928

LM3=54
5.8704 -0.2828 1.3398
6.7839 -0.8049 1.2234
22.662 0.5689 -4.2427
24.6965 1.6316 -4.6917
23.0655 8.0275 -4.4047
28.3927 5.2186 -4.079
26.8462 4.9549 -5.1135
36.7647 0.2284 5.3904
33.2144 10.9076 -9.2312
37.2922 10.6978 -5.318
40.2806 9.4563 -7.9229
38.0028 -0.941 -8.2388
45.3279 9.3828 -8.839
47.4365 3.9477 -9.503
54.8296 11.0297 -10.0021
58.6709 11.9694 -9.8933
57.9386 18.7769 -11.6014
63.0095 20.4023 -2.8627
63.7008 9.9123 -7.1407
63.9518 15.2163 -11.2534
4.4869 3.543 1.1449
4.7922 5.0528 1.0212
18.2432 13.2548 -4.1955
20.9253 13.5794 -4.9487
25.5338 12.8937 -4.3673
24.0481 12.3277 -5.0055
29.8352 21.849 5.4761
36.1391 13.6765 -4.8279
37.7775 16.4715 -7.7512
30.0406 23.6626 -8.0395
41.2367 19.1354 -9.1456
40.6877 25.0309 -9.13
50.5546 23.6171 -10.3873
54.5096 25.0663 -10.0055
57.0411 29.076 -7.9711
60.5796 25.3632 -11.3713
5.9661 2.4346 7.9323
5.8472 4.9873 7.0996
19.1235 12.3826 8.4125
23.7743 15.8043 -0.6633
22.909 17.184 -6.7487
28.7773 9.3393 10.8324
44.3662 14.5887 12.5411
52.113 16.8826 10.4178
53.3385 32.7161 -2.7246
61.6736 20.2351 4.2176
59.2114 27.5625 -5.1461
5.799 1.7623 7.7772
7.4131 -0.5886 7.1053
22.7988 1.4726 8.591
28.5116 1.4705 -1.2322
28.4385 -0.3836 -6.9896

62.5049 4.9067 -2.6645
64.3499 13.1008 -5.2049
ID=PHAtriFEMLIBUSNM481811

LM3=54
6.0556000000002 -0.122800000000789 1.89519999999992
7.82520000000156 -1.0860000000001 2.03599999999997
27.3941000000004 0.625199999999691 0.881399999999932
28.4951000000005 2.61829999999961 0.516699999999954
28.4723000000009 9.34459999999985 0.477200000000011
33.1290000000011 6.88389999999999 2.33059999999998
32.3118000000018 6.79680000000019 0.848300000000081
39.0285000000007 1.23519999999975 14.6981
40.4699000000008 13.5003999999999 -0.859
43.5374000000006 13.0491999999998 5.58089999999997
46.9695000000005 11.5926999999998 3.45339999999995
44.7368000000007 -0.242700000000203 -0.417600000000053
51.8590000000009 12.2975999999999 2.98619999999997
54.9415000000012 7.39019999999994 2.98789999999999
62.6125000000012 13.8218 4.59789999999996
65.7597000000016 15.2339 5.4842
66.0130000000015 22.192 3.84589999999998
70.5871000000018 23.6561 12.3236
70.1885000000018 12.9604 9.3637
71.9368000000017 19.1798 6.0191
4.3685000000011 3.72149999999956 2.11319999999998
5.17630000000112 5.35679999999958 2.22079999999996
22.2537000000009 15.8731999999999 0.503800000000017
23.7987000000008 14.3919999999999 0.906199999999999
30.0295000000008 14.3807999999999 2.16540000000001
29.4636000000008 14.0735999999999 0.837300000000007
33.2574000000009 22.8740999999998 13.6554
42.1624000000006 15.9954999999998 5.03419999999996
44.9410000000006 19.0030999999999 3.50499999999997
34.814000000001 27.1008999999999 0.243300000000002
48.9286000000009 21.5430999999999 2.59409999999998
48.5166000000011 27.4277999999999 2.74909999999998
58.1821000000013 25.9097999999999 4.34259999999998
61.5022000000016 27.9300999999999 5.619
63.9176000000018 31.9522999999999 8.6843
68.6413000000019 28.4044 6.31660000000001
3.7574000000012 1.15499999999955 9.10199999999994
3.90850000000118 5.08149999999955 8.47899999999996
19.6521000000008 12.5829999999997 13.6357
28.1884000000008 18.1356999999999 3.62750000000001
26.6192000000008 20.6749999999999 -0.767499999999986
30.1006000000008 10.7318999999997 17.8174999999999
48.2363000000011 17.1369999999998 23.6713999999999
56.2195000000013 19.8889999999998 22.8478999999999
57.4251000000017 35.9614999999999 11.5587
66.8433000000017 24.0115999999999 18.3709
64.5713000000017 31.8633999999999 9.4566
4.00300000000119 0.724999999999555 9.13359999999995
6.29740000000117 -1.83890000000041 8.25249999999995

22.5599000000007 2.23329999999967 13.3089999999999
33.118000000001 3.19399999999984 4.46549999999999
33.6912000000009 0.514299999999816 -0.311100000000014
66.7322000000016 8.57639999999993 11.5659
70.2453000000017 17.0424 9.814
ID=PHAtriMALLIBUSNM481812

LM3=54
5.4047 -0.091 -0.045
6.8384 -1.2111 -0.3238
26.6063 0.2699 -6.5852
27.2964 1.1098 -6.7885
27.3542 8.1602 -6.721
31.9242 5.5308 -6.4401
30.0148 5.5123 -7.6117
38.4832 -0.152 5.1672
36.7855 11.1245 -11.248
41.4681 10.7542 -5.4982
43.6823 9.1584 -8.1721
41.0391 -1.3109 -9.3847
48.5207 9.3179 -9.9856
50.5869 3.8256 -9.27
59.0245 9.999 -9.0573
62.2094 10.9387 -9.0432
61.1189 17.9639 -11.6339
67.3795 20.0611 -4.9119
67.1264 10.2717 -7.7387
66.3493 14.9885 -10.5466
4.0992 2.961 0.2059
5.12 4.8699 -0.9416
21.9423 14.2997 -7.0958
23.0313 13.499 -7.0807
29.6507 12.7616 -6.3835
28.0139 11.4512 -7.7791
31.6334 20.9758 4.7404
40.0491 14.0166 -5.7755
41.2592 16.154 -8.3139
33.1868 23.2787 -9.5338
45.4067 18.915 -9.8687
43.8875 24.473 -9.4636
54.5199 23.9966 -9.8924
58.0828 24.6362 -9.5168
61.4476 28.2371 -8.2333
63.9205 23.5192 -10.9258
4.8542 1.3453 7.0758
4.8356 4.509 6.8768
21.3352 11.5253 8.8521
27.1108 15.9921 -3.7438
25.4899 17.3488 -7.7785
30.1881 8.9785 10.5355
50.2976 15.0769 11.7611
58.1459 17.6584 9.3671
55.7511 32.381 -4.6146
67.0921 20.1643 2.7854

63.232 28.3303 -6.7488
5.6135 0.8623 7.6101
6.7671 -1.5899 6.7496
23.9136 1.7806 8.9202
31.8821 2.3432 -3.4035
31.2991 -0.1481 -7.5536
64.3368 3.8836 -4.8655
68.1036 11.2121 -6.6106
ID=PHAtriFEMDRCUSNM537784

LM3=54
6.03460000000181 -0.699199999999542 0.956099999999795
7.50919999999955 -1.38000000000023 -0.754399999999906
27.4605000000009 -0.0674999999996658 -5.49260000000009
28.4454 1.58959999999999 -6.13489999999998
27.9346000000007 7.77980000000021 -4.25950000000008
32.7137000000006 4.77200000000026 -5.92260000000009
31.5332000000006 5.30940000000015 -6.82930000000007
43.0563000000003 0.0402000000001084 4.32379999999996
39.9767000000001 10.8984000000001 -10.3766
44.8153000000001 10.9028000000002 -6.10850000000007
46.6135000000004 9.22380000000017 -8.65480000000008
44.9922000000005 -1.39439999999979 -9.58070000000006
53.6863999999999 10.2166000000001 -9.80700000000002
55.3891000000002 4.47020000000014 -10.1929000000001
65.7789000000002 11.5562000000001 -9.88350000000006
67.9194000000001 12.8568000000001 -10.1898000000001
69.1172000000003 20.4947000000002 -11.6373000000001
73.4474000000005 21.4521000000002 -2.15160000000013
73.1545000000003 10.9141000000001 -7.40530000000011
73.6990000000002 16.0227000000001 -10.6379000000001
5.02780000000158 3.21060000000033 0.60679999999984
5.51280000000144 4.3053000000003 -0.473300000000169
23.2942000000009 13.8446000000002 -5.0120000000001
25.5616000000008 13.0523000000002 -5.6394000000001
30.4330000000007 12.6051000000002 -5.41710000000009
29.4491000000006 11.5057000000002 -6.68440000000009
35.6755000000007 21.7400000000002 4.52749999999986
43.3808000000002 14.1753000000002 -6.38060000000007
44.1825000000003 16.9527000000002 -8.49850000000006
37.4295000000006 24.3833000000002 -9.8402000000001
50.3529000000003 20.1605000000002 -9.76290000000007
49.4711000000004 25.9968000000002 -9.78970000000007
60.7292000000003 26.0333000000002 -10.0807000000001
63.3497000000005 25.8659000000003 -9.88270000000013
66.5920000000005 30.3058000000002 -7.33200000000016
70.0246000000004 27.1245000000002 -10.8512000000001
5.6962000000015 1.78740000000031 7.50679999999983
4.37300000000162 4.16800000000034 6.9213999999998
23.0229000000011 10.8858000000003 8.73909999999985
29.6540000000008 15.9294000000002 -2.67940000000011
27.4101000000008 16.1991000000002 -6.6251000000001
31.8412000000007 8.59370000000022 10.6251999999999
53.3290000000005 15.2713000000002 11.2804999999999

60.0916000000005 17.3220000000002 9.66749999999988
61.7066000000006 33.6832000000003 -2.42960000000016
71.7836000000005 21.4285000000002 3.37179999999986
68.6822000000005 29.7264000000002 -5.34720000000016
5.88130000000145 0.852000000000295 7.78609999999985
6.23230000000109 -2.15099999999977 6.86119999999989
25.5448000000008 1.92760000000025 8.4265999999999
33.5124000000005 0.648300000000195 -4.69610000000006
32.0813000000005 -0.575899999999812 -6.86880000000005
70.7158000000003 5.50080000000013 -2.7640000000001
74.4171000000003 13.5964000000001 -5.17340000000011
ID=PHAtriMALDRCUSNM537785

LM3=54
6.92529999999928 -0.271200000000023 0.607000000000011
8.87820000000005 -0.959300000000207 0.617600000000007
26.8163999999998 0.00309999999999924 -4.47930000000006
27.6957000000001 1.19550000000001 -4.66980000000011
28.8311999999997 7.62089999999994 -5.29660000000004
33.7162999999999 5.1002 -4.65990000000005
31.4676999999994 5.36419999999978 -5.92299999999997
41.2076999999998 -0.110800000000053 4.38539999999994
37.6883000000003 10.0741000000001 -9.37040000000012
44.056876840778 10.3588394184086 -4.93707666836687
46.2529917412795 8.92401886091592 -7.48936948480299
43.6747999999994 -0.780400000000112 -8.68780000000006
52.4882783484835 9.29996644982355 -9.10334586936026
57.6594999999998 4.71659999999995 -9.55750000000012
62.8005 9.98680000000008 -10.5171000000002
66.334 11.9376000000001 -9.31950000000017
66.3442000000001 18.0489000000001 -11.0056000000002
72.1037000000001 19.4831000000001 -3.69860000000019
71.4714 10.4724000000001 -8.26920000000018
70.6462000000001 13.8231000000001 -10.9966000000002
6.72949999999993 3.24569999999999 0.747999999999964
7.26900000000009 4.7323 0.639799999999932
23.8596 13.6946 -4.13670000000008
25.4926 13.6878 -4.80330000000007
31.8525 12.2137 -5.07190000000006
30.3379999999998 11.0778 -6.02570000000005
35.9753000000001 20.4639 5.0945999999999
43.6314720384226 12.815318820678 -4.89855049522427
44.8488653223275 15.2436217568336 -7.23465896876518
37.7013000000002 22.1684000000001 -8.50630000000013
49.9159367643166 18.191485845761 -8.75396046336218
51.8806 24.7064000000001 -9.12150000000014
63.7354000000001 21.9402000000001 -10.4744000000002
67.7381000000001 22.9160000000001 -8.98850000000018
71.1053 26.1930000000001 -7.63580000000018
72.9661000000001 22.6944000000002 -10.5443000000002
5.55639999999999 1.9141 8.24119999999997
5.2530000000001 4.91870000000003 6.65779999999997
21.9687000000002 10.94 8.72109999999993
28.354 15.8381000000001 -3.01900000000006

26.8777 17.0364000000001 -5.45650000000008
31.5024 7.92650000000001 11.2440999999999
54.7783999999999 14.4653 11.4125999999999
61.3853 16.3892000000001 8.96399999999989
60.8559 31.2764000000001 -5.03580000000017
68.0231 18.3926000000001 5.06659999999985
67.9688000000001 25.6192000000001 -5.90910000000018
4.70509999999999 0.837799999999995 7.80089999999998
7.02539999999998 -1.72350000000006 6.43619999999999
25.0988 0.939299999999978 8.52179999999995
32.7761999999999 -0.294600000000012 -3.72970000000005
32.5552999999997 -1.14530000000006 -5.13400000000005
69.2547 4.27670000000005 -4.76210000000016
71.8624000000001 12.5155000000001 -5.49700000000017
ID=PHAtriFEMCONKMMA12331*

LM3=54
6.52840000000342 -0.0118999999992738 0.949799999999095
7.16080000000321 -0.27799999999929 -0.344000000000838
27.2700000000022 0.712200000000385 -5.25310000000048
29.3970000000021 1.62490000000034 -5.74110000000042
28.7526000000019 8.02480000000026 -6.02220000000037
33.7230000000017 5.76590000000023 -5.79690000000032
32.9859000000017 5.80510000000023 -7.07560000000033
42.8295000000019 0.970500000000319 5.26089999999971
41.1199000000014 10.1224000000002 -11.4169000000002
47.6006000000011 11.3097000000001 -6.97770000000009
49.7062000000011 9.21970000000016 -8.45590000000008
46.0534000000016 -1.16579999999973 -10.2287000000002
54.8758000000012 9.35500000000022 -9.62960000000002
59.6739000000013 4.69850000000028 -9.83280000000004
65.4688000000015 10.0035000000004 -10.176
69.5227000000019 11.3194000000005 -8.37690000000002
69.5304000000019 17.8190000000005 -10.865
76.0328000000025 19.0598000000007 -4.92450000000007
74.0631000000023 9.66020000000062 -8.19550000000004
75.3325000000023 13.3176000000007 -10.2872000000001
6.76890000000345 4.03900000000077 0.677199999999118
6.86090000000335 5.32140000000073 0.0674999999991566
25.5869000000023 14.3095000000004 -5.52580000000048
27.3983000000022 14.1054000000004 -6.00870000000046
32.8353769166557 12.397013554276 -6.07823859654586
32.1369546629617 11.2685068961325 -7.55832096410218
39.110200000002 21.7190000000003 4.1759999999997
47.1662000000012 14.0754000000001 -6.66520000000009
47.9089000000012 17.0542000000002 -9.26070000000008
39.6173000000019 23.7588000000003 -10.1283000000002
52.7251362381614 18.9213911401179 -10.1024628111082
54.2309000000017 24.9863000000003 -10.0617000000001
62.1077000000018 22.9866000000004 -10.3096
66.760600000002 24.7732000000005 -8.46450000000004
69.8787000000024 27.9674000000006 -8.40300000000009
71.9311000000024 24.0040000000006 -10.4321000000001
5.82640000000357 2.52260000000079 8.23989999999908

5.97250000000351 5.26310000000077 7.0767999999991
23.3865000000025 11.6540000000005 8.74669999999944
29.8069000000019 16.9492000000003 -3.63950000000037
28.2594000000021 18.1383000000003 -6.87770000000041
31.3211000000022 8.68870000000039 10.2284999999996
53.8616000000022 15.2002000000005 10.8413999999998
63.2631000000024 17.1144000000006 8.92469999999985
62.7380000000024 32.3855000000006 -4.62790000000011
72.8346000000026 19.7141000000007 3.3825999999999
70.5248000000025 26.9478000000006 -6.36350000000008
5.48050000000354 1.6358000000008 8.07909999999908
7.57330000000337 -1.76139999999927 7.01709999999914
25.7906000000024 2.46270000000047 8.67689999999945
34.1229000000019 1.82990000000028 -3.62140000000036
33.529800000002 -0.513699999999693 -6.57030000000038
70.2098000000021 3.77120000000057 -4.63920000000009
74.7832000000024 12.2109000000007 -5.87230000000006
ID=PHAtriMALCONKMMA12332*

LM3=54
6.18730000000144 0.449100000000803 1.07129999999947
7.46740000000143 -0.0713999999991577 0.581999999999559
23.4499000000011 -0.323299999999309 -4.02440000000034
24.8506000000009 1.20460000000063 -3.99440000000031
25.165400000001 7.84960000000055 -4.9689000000003
28.033100000001 4.7320000000006 -4.54180000000029
27.8583000000009 5.25970000000057 -6.36250000000029
36.0713000000016 0.264700000000848 5.99709999999962
34.2872000000014 10.1452000000007 -9.88950000000029
38.1518000000015 10.2949000000007 -6.6327000000003
40.9789000000017 9.5023000000008 -8.46390000000032
39.5302000000017 -0.117499999999081 -8.88610000000036
46.124800000002 9.30480000000092 -8.81710000000036
51.2873000000022 4.98620000000104 -8.72490000000039
56.3317000000024 11.5391000000011 -9.81590000000044
59.5243000000027 11.9094000000013 -9.66780000000049
59.2660000000027 18.9043000000012 -11.8058000000005
65.4670000000029 20.3369000000013 -4.83530000000055
64.8474000000028 10.5594000000014 -8.29740000000053
63.678000000003 14.6930000000014 -11.4720000000006
5.86930000000148 3.16780000000085 0.870899999999513
6.84510000000148 5.40830000000084 0.628199999999507
20.004900000001 13.7535000000005 -4.27640000000036
21.4008000000009 12.8608000000005 -4.67130000000034
26.0844000000009 12.1159000000005 -4.0691000000003
26.124000000001 11.2407000000005 -5.86120000000031
30.3600000000013 20.8282000000006 5.82769999999961
38.0271000000015 13.8359000000007 -6.2116000000003
39.5097000000016 15.8797000000007 -8.33440000000031
32.5401000000016 23.7654000000007 -8.44350000000037
43.7246000000019 18.4785000000008 -9.01360000000034
45.542800000002 25.3059000000008 -9.23690000000039
52.3878000000024 23.151500000001 -9.87230000000045
55.1754000000026 24.9386000000011 -9.83100000000051

59.5290000000029 29.0334000000012 -8.38860000000055
60.3594000000029 24.6160000000013 -11.3414000000006
4.82400000000154 1.46350000000088 7.08929999999949
4.29840000000151 4.97120000000087 6.54419999999949
20.2456000000011 11.6665000000006 9.54049999999958
24.190500000001 14.0687000000005 -3.26470000000033
20.6548000000011 15.1939000000005 -4.94370000000035
26.5409000000013 8.4426000000007 10.8411999999996
46.9316000000021 14.503100000001 11.0655999999995
54.5817000000024 16.7629000000011 8.71459999999952
53.2344000000027 31.8011000000011 -2.99120000000053
63.4070841642412 19.6195776509129 3.28233781040929
60.5928000000029 27.5033000000012 -7.31290000000056
4.91540000000155 1.4173000000009 7.09119999999949
6.21600000000152 -1.50929999999911 6.21539999999951
22.7259000000012 2.9004000000007 9.37959999999959
27.779100000001 2.18280000000065 -2.64210000000031
26.7167000000011 -0.280299999999282 -3.92670000000032
62.0477000000026 4.45370000000131 -2.25360000000049
65.3872000000029 12.9036000000014 -6.80850000000054
ID=PHAtriUNKCONKMMA12543*

LM3=54
5.76910000000038 0.823500000000196 1.24079999999984
7.29240000000055 -0.532599999999827 1.0019999999999
26.1464000000008 0.670500000000441 -4.64500000000009
27.4458000000008 1.97320000000047 -4.7320000000001
27.9487000000009 8.32740000000056 -3.44640000000011
32.076200000001 5.93650000000054 -4.91660000000013
31.012400000001 6.27590000000057 -6.15070000000013
41.2514000000008 0.173600000000327 4.44669999999991
38.8068000000009 11.4955000000005 -9.4449000000001
42.0622000000008 10.6574000000004 -6.31500000000011
46.417400000001 8.50760000000034 -7.4211000000001
45.115700000001 -0.232499999999646 -9.71600000000008
52.417700000001 9.23030000000024 -9.0203000000001
57.8996000000011 4.90210000000015 -9.14130000000008
62.6759000000011 9.62509999999997 -10.4735000000001
65.7853000000011 11.1548999999999 -9.58490000000006
65.309000000001 16.6208999999998 -11.4638000000001
71.5960000000008 18.5371999999997 -2.83350000000001
71.3891000000011 9.45289999999982 -8.60920000000007
69.337200000001 12.9017999999998 -11.2890000000001
7.10540000000039 2.5925000000002 1.21209999999991
7.76340000000034 4.40220000000021 0.741099999999923
24.5402000000005 13.4575000000005 -4.25580000000008
25.8290000000006 12.5731000000005 -4.66820000000008
31.7456000000008 11.5427000000005 -4.8559000000001
31.0522000000008 9.93840000000055 -6.3198000000001
35.8081000000003 20.0822000000002 3.90889999999993
42.8310000000008 11.9064000000004 -6.13730000000008
44.9538000000008 14.7201000000003 -7.7490000000001
38.6505000000007 22.0319000000004 -9.8172000000001
50.1349000000008 16.9390000000002 -9.4465000000001

52.6518000000007 23.4932000000001 -9.82900000000008
59.2354000000008 21.9796999999999 -11.0790000000001
61.9711000000007 23.5976999999998 -10.255
66.3592000000007 26.4339999999998 -8.63640000000003
66.8922000000008 21.9486999999998 -11.7839
6.01770000000022 1.6700000000001 7.44579999999994
6.21960000000023 4.99540000000014 6.76479999999992
21.8280000000004 9.66890000000027 8.79389999999992
26.6696000000006 15.3552000000005 -3.70990000000009
25.4204000000006 16.3738000000005 -5.60280000000009
29.1095000000005 8.17920000000027 9.87919999999991
51.8175000000006 14.7053 11.1630999999999
58.5447000000006 16.3796999999999 9.34309999999994
58.4414000000005 31.5334999999998 -3.39070000000005
66.5558000000006 19.0525999999997 4.31769999999997
65.7647000000007 26.1206999999998 -7.03210000000004
6.11960000000022 1.24740000000014 7.37469999999992
8.41840000000031 -1.26039999999987 7.04119999999992
23.6777000000005 3.93460000000028 8.77369999999992
30.5080000000009 1.73720000000049 -3.25480000000012
30.4833000000009 0.304500000000487 -6.19150000000011
67.191300000001 4.67409999999986 -3.60930000000007
69.814600000001 12.6925999999997 -7.45040000000005
ID=PHAtriUNKCONKMMA12544*

LM3=54
5.05940000000004 -0.279800000000091 0.799200000000115
6.36910000000008 -0.894300000000122 0.38750000000003
24.1639000000005 0.862400000000188 -5.24630000000009
24.5643000000006 1.90030000000021 -4.79000000000009
26.0992000000007 7.97530000000027 -4.97170000000012
27.7256000000007 3.99090000000028 -5.00700000000011
27.6394000000007 4.93600000000029 -5.84960000000011
37.2849000000005 0.538300000000223 5.12919999999991
34.6582000000006 10.2661000000003 -8.91870000000012
39.3422000000006 10.3324000000003 -6.63630000000011
40.9749000000007 8.85830000000033 -7.83630000000009
39.0419000000006 0.586700000000293 -9.92030000000008
46.5610000000007 9.2642000000003 -10.7366000000001
51.0620000000006 5.01540000000027 -10.5470000000001
56.4485000000005 10.0025000000002 -11.9112000000001
62.7097000000004 10.7390000000002 -10.2365000000001
59.6164000000003 17.2074000000002 -12.7112000000001
66.1283000000001 19.3916 -5.47630000000003
65.5609000000003 9.62640000000018 -10.2049000000001
64.9018000000003 13.3248000000001 -12.4241
3.98959999999996 2.74699999999984 0.464600000000001
6.52189999999996 4.95639999999988 0.690699999999997
21.6780000000004 12.6398000000001 -4.48320000000008
21.7084000000004 12.6001000000001 -4.85690000000008
26.5380000000006 11.8072000000002 -5.14290000000011
26.8934000000006 11.0547000000002 -5.76310000000012
32.0816000000002 19.8495000000001 4.60199999999992
38.1921000000006 12.8011000000003 -6.7830000000001

39.4717000000007 15.2191000000003 -8.2737000000001
33.3408000000005 21.2951000000002 -8.99630000000009
44.2461000000006 17.4080000000003 -10.0965000000001
46.0332000000004 23.2884000000002 -10.2881000000001
53.4873000000003 21.9544000000001 -11.6180000000001
58.0728000000002 24.2884000000001 -10.6531000000001
59.7816000000001 26.6869 -10.2141
62.3525000000002 23.5769000000001 -12.619
5.5947999999998 1.1848999999998 6.87210000000002
6.35849999999983 3.4788999999998 5.89740000000003
21.2052000000001 8.55059999999997 7.61019999999994
24.0898000000004 13.6034000000001 -2.72940000000011
23.1167000000004 14.3404000000001 -5.03310000000008
26.6649000000002 7.14010000000004 8.66429999999993
48.2640000000002 13.8792000000001 9.78329999999995
54.9630000000001 16.2468000000001 7.62619999999997
54.5650000000001 29.8719 -6.90350000000005
62.486 18.4621 1.3262
61.7584000000001 25.5834 -8.72980000000002
5.92279999999981 0.189499999999769 5.83250000000005
7.88349999999986 -1.21820000000018 5.44680000000002
22.8408000000001 2.72870000000001 7.49649999999994
28.2604000000007 0.678500000000262 -1.79440000000011
27.9181000000006 -0.161199999999743 -4.91000000000009
61.9803000000003 4.12810000000016 -5.95160000000005
65.9346000000002 12.2007000000001 -8.31220000000002
ID=PHAtriUNKCONKMMA15907*

LM3=54
6.1064 0.0465 1.0943
7.1403 -0.7931 0.7368
25.5898 0.358 -4.0138
25.862 1.0083 -4.4754
26.3953 6.6976 -4.9633
30.2956 4.0483 -4.6684
28.9937 4.3528 -5.5889
37.967 0.3989 5.2763
35.0233 9.3078 -8.8646
44.3996 9.6919 -5.5179
44.7975 8.4133 -7.9495
42.5122 -1.6682 -7.8652
50.0327 8.068 -9.0518
53.5834 3.3794 -8.0201
60.374 8.2183 -9.5415
64.1149 9.3497 -9.3003
64.167 15.5183 -12.4531
68.9487 17.368 -5.9589
69.148 7.5185 -7.6013
68.1167 11.4619 -11.7795
5.1205 3.7444 0.6134
5.4228 4.892 0.0799
22.4394 13.6225 -5.4902
22.7368 12.1602 -5.3332
28.687 11.8239 -5.2872

27.4198 10.8014 -6.4922
34.5017 20.984 3.5131
43.04 12.4728 -6.2412
43.6841 14.4367 -8.7981
37.4306 21.7941 -8.294
47.9318 16.4503 -10.2237
49.7942 22.7243 -9.7181
56.9013 20.5352 -10.8016
58.5772 22.7637 -10.2251
64.2578 25.5317 -9.2207
65.1096 20.6239 -12.584
6.1152 3.1126 7.0275
5.4681 4.8573 5.9118
21.3705 10.9613 7.8449
27.1744 14.53 -4.233
24.8269 15.1876 -7.1521
30.1823 9.5316 9.9072
49.9708 13.9198 11.7608
59.5168 16.0725 8.9031
59.0567 28.2708 -4.1987
65.6516 17.0711 5.325
66.2782 23.3618 -7.3544
5.6377 2.1062 6.812
8.1792 -0.2996 6.4656
23.9928 3.8125 8.6208
30.7486 1.7171 -1.9306
29.6205 -0.5512 -5.6035
64.9189 2.935 -1.1951
69.4927 9.5232 -5.5931
ID=PHAtriUNKCONKMMA28686*

LM3=54
1.80467778567267 -0.440463068066886 -5.26855938495011
2.84258794932192 -1.02428258374718 -5.67612809977667
19.9016999999999 0.241400000000069 -8.31170000000004
22.0583999999997 1.34379999999983 -8.40750000000004
20.5620999999997 7.61139999999993 -7.61109999999997
25.2920999999999 4.89610000000013 -7.08210000000001
24.1157999999987 4.63489999999969 -8.07539999999996
33.5940999999996 -0.0993000000000828 3.48559999999996
33.6671 12.0833000000001 -10.685
36.9667000000001 11.5341000000001 -6.30070000000004
39.9298999999997 10.4131 -8.22120000000003
36.8982999999996 -0.733700000000041 -10.3123
45.1366 10.2095 -8.56310000000003
49.8653999999999 6.51650000000001 -7.28800000000004
55.6616999999998 11.9435999999999 -7.30260000000006
59.5293999999999 12.6418 -5.89450000000005
59.0187999999999 19.9485 -7.51640000000004
62.282 20.8851 2.05739999999995
64.3778999999998 11.1159 -3.61630000000006
64.6146999999999 15.7033 -5.87050000000006
0.915603459685778 2.68644389891554 -4.96664625110443
1.36349999999969 4.0813999999999 -5.14749999999998

16.2582999999998 12.4355 -8.39249999999998
18.2515999999998 12.7273 -8.26490000000001
23.0374999999998 11.7221 -7.0875
22.0920999999997 11.2872999999999 -7.92319999999999
27.4105 22.2842 3.98879999999995
36.0102000000001 14.3412000000001 -6.42670000000003
38.2661000000002 16.9087000000001 -8.29480000000004
28.8798 24.3459 -9.65730000000001
41.8835000000001 19.7943 -8.48280000000004
43.4545 25.622 -7.37670000000003
51.3039 24.546 -7.55500000000005
54.5597000000001 26.4748 -5.72990000000006
57.9394 30.2352 -3.18820000000005
60.1571 25.665 -7.31010000000006
-0.0904000000003238 0.104599999999927 -0.0514000000000079
0.809299999999702 2.97229999999992 -0.248600000000006
15.4504999999998 9.82949999999996 5.02959999999997
21.3970999999999 15.5401 -4.9648
20.2274999999998 16.9629 -8.9417
24.6501999999998 8.68409999999999 8.67389999999997
42.2541999999999 14.2256 12.5264
47.3495999999999 15.5361 12.0967999999999
51.5075 32.1348 -1.22760000000004
58.2573 19.5746 9.24419999999993
59.1172 27.89 -1.11790000000006
0.162699999999678 -0.417200000000066 0.113099999999989
2.01349999999968 -1.70370000000006 0.136399999999995
17.4360999999998 2.30249999999999 5.08489999999997
25.4770999999999 0.821400000000086 -4.28470000000003
25.9307999999997 -0.796499999999996 -8.6578
61.0652999999998 5.43819999999995 -2.14750000000007
63.3132999999998 12.7846999999999 -0.882200000000063
ID=PHAtriUNKLIBKMMA38504*

LM3=54
2.58400000000173 -0.376599999999718 -6.72350000000028
5.12880000000155 -1.28489999999986 -6.5985000000002
22.941700000001 1.20689999999994 -7.70500000000015
24.1685000000009 1.77389999999993 -7.36670000000014
24.0379000000008 8.86629999999989 -6.83850000000012
27.5721000000007 6.31919999999988 -7.12620000000008
27.9261000000008 7.67399999999988 -8.32190000000011
33.4908000000008 0.0938999999999922 4.24959999999995
33.3822000000007 11.8146999999999 -9.62119999999999
38.8015000000005 11.9933999999999 -5.14319999999994
41.0936000000004 10.5327999999999 -7.00569999999991
38.9871000000007 -0.153600000000082 -9.1042
46.7741000000004 10.1113999999999 -7.19789999999988
51.6831000000004 5.78959999999994 -5.93889999999988
56.4179000000004 11.7501 -4.98829999999982
59.9024000000004 12.9044000000001 -4.12489999999982
58.8480000000006 19.0271000000001 -5.69249999999982
64.2104000000009 20.1591000000003 3.33320000000017
65.0398000000005 10.7454000000001 -1.14299999999985

64.9277000000005 14.5573000000001 -3.6807999999998
1.74810000000166 3.09880000000018 -6.50730000000023
3.64880000000168 5.21430000000015 -6.3081000000002
19.171800000001 14.4201 -7.78300000000015
20.783800000001 14.2105999999999 -7.65170000000013
25.1316000000007 13.2155999999999 -6.96830000000009
26.1390000000008 12.2979999999999 -8.22860000000009
26.7571000000009 20.4604 4.43609999999995
38.5705000000005 14.1513999999999 -4.99459999999996
39.4820000000006 17.1139999999999 -7.15129999999993
31.4794000000008 25.1351 -8.40659999999998
44.0062000000006 19.8255 -7.14429999999989
46.4660000000008 26.3480000000001 -5.70799999999989
53.1914000000006 24.1206000000001 -4.60529999999984
56.8857000000008 24.6464000000002 -3.85759999999983
59.9291000000009 28.8740000000002 -0.392799999999825
61.4658000000009 24.9526000000002 -3.20179999999981
-0.211199999998248 0.00440000000020424 0.0371999999997543
1.54710000000166 4.3602000000002 -0.319400000000235
14.5557000000012 10.9599000000001 3.69189999999983
23.194000000009 16.0630999999999 -4.59710000000011
21.3372000000009 18.2279999999999 -8.5290000000001
25.214900000001 9.56860000000002 9.01159999999988
43.6000000000008 14.3291000000001 14.1096000000001
50.9581000000008 16.7552000000002 13.8679000000001
52.685300000001 32.4565000000002 1.24520000000013
59.0662000000009 19.0940000000002 11.4110000000001
59.552000000001 27.4373000000003 0.836300000000157
0.140800000001764 0.0427000000002097 0.0516999999997627
3.74650000000175 -1.6779999999998 0.0670999999997732
19.1144000000011 1.54800000000004 4.41499999999982
26.9351000000008 3.14109999999992 -5.28460000000012
27.2646000000008 0.406999999999929 -8.2100000000001
59.6524000000005 4.57310000000007 1.30780000000014
63.5591000000006 11.6478000000001 0.87140000000017
ID=PHAtriUNKCONKMMA6993**

LM3=54
4.9082 -0.3089 1.9314
6.4581 -0.7558 1.2096
24.7397 -0.3774 -4.1355
24.6652 1.0364 -4.2909
26.7878 7.2549 -4.6993
28.3872 3.9128 -4.3898
27.1611 4.5409 -5.3097
37.7741 -0.3445 5.9013
35.2887 10.3338 -9.5457
39.9982 10.3085 -5.2864
43.0987 9.3952 -7.7582
40.3118 -1.1502 -7.5481
48.3495 9.7664 -9.0127
53.9688 5.6859 -8.5701
59.1425 10.5101 -9.8316
63.483 11.3805 -8.5992

62.7429 18.4863 -10.314
68.5799 20.0164 -2.8593
68.3874 10.2473 -7.4351
68.1911 14.7228 -10.064
4.3365 2.3475 2.4696
5.5105 4.0347 1.5552
20.5283 12.9861 -3.883
21.29 12.1411 -4.3751
26.345 11.6321 -4.2695
25.8618 10.7388 -5.381
31.8767 19.9094 5.7556
39.3108 12.6486 -5.5833
41.6175 15.1614 -8.0119
33.2815 22.4014 -7.2739
46.1434 17.7586 -9.1892
49.1503 24.5928 -8.8758
55.3454 22.8682 -10.0226
59.4361 24.7254 -8.6634
63.3219 28.6705 -7.1968
64.7035 24.2755 -10.1883
3.4509 0.6963 7.3433
3.6235 3.7039 6.7128
18.5585 9.6506 8.9192
23.0432 14.7163 -2.724
22.765 15.2657 -5.6919
28.55 7.78 11.048
48.4106 14.1033 12.146
56.6694 16.8845 10.0998
55.8296 31.7948 -3.2403
64.9893 19.3468 6.0187
63.1575 26.7363 -4.7567
3.7163 -0.3682 7.3906
5.4698 -2.0285 6.5837
21.0291 0.5312 8.4467
27.8064 -0.1671 -2.9161
28.0286 -0.9545 -5.2006
64.6396 4.796 -2.4688
68.5211 13.7099 -4.7827
ID=PHAtriUNKCONKMMA6M0576

LM3=54
3.9109 -0.4314 0.7865
4.8763 -0.9894 -0.3255
22.7009 0.471 -4.3982
22.9627 1.9927 -4.5314
24.9828 8.0668 -4.8482
28.0315 5.9389 -4.6997
27.1625 6.3541 -5.938
36.5804 1.009 5.9807
34.9836 11.948 -9.8021
39.4219 11.5619 -5.2973
43.3049 10.6078 -7.8956
40.4471 0.2104 -8.7374
47.9952 10.7137 -9.5284

53.3314 5.6828 -9.9518
56.9071 11.3972 -10.5168
60.62 12.5164 -9.934
60.5574 19.2854 -11.9372
65.6768 21.0903 -5.1325
65.9694 11.4035 -8.6031
65.3488 15.8804 -11.8322
3.1126 3.4272 0.8155
3.6058 5.0347 -0.2596
19.147 13.6784 -4.1812
20.5984 12.6765 -4.6249
26.9269 12.4244 -4.6384
26.2252 11.4026 -6.0185
31.2364 21.3081 6.1119
39.1471 13.2205 -5.537
41.2948 15.7963 -8.574
33.4778 23.1988 -9.2027
45.6061 18.6158 -9.7393
45.6154 26.1636 -9.7356
56.676 25.2772 -10.0907
56.8155 25.2853 -10.113
60.8204 29.1211 -8.5505
62.5439 24.8599 -12.0313
2.4974 1.5248 7.7608
2.0642 4.0032 6.5732
18.0262 10.6374 8.6915
24.1094 15.0082 -2.5798
21.9915 16.419 -5.1858
25.8032 8.5871 10.7524
49.0974 15.4663 10.481
54.35 17.4386 8.3999
54.0488 31.5538 -6.7783
63.0085 20.279 1.8833
60.5612 27.1138 -8.1511
2.4663 0.7063 7.5938
5.066 -1.4756 7.0377
20.6119 2.2597 8.62
28.4122 2.1482 -2.2684
27.838 0.0505 -5.2273
62.2406 5.8525 -6.5612
65.2609 13.2158 -7.7687
ID=PHAtriUNKCONKMMA6M0582

LM3=54
5.9075999999992 0.354600000000254 1.55869999999993
7.14840000000108 -0.470999999999621 0.651699999999743
25.2406999999998 -0.395400000000036 -4.1692
27.2767999999999 0.835699999999882 -4.95749999999999
28.4970999999996 7.69529999999986 -4.86669999999999
31.3937000000006 4.66780000000015 -4.98280000000006
30.1918000000002 5.51169999999998 -6.19900000000004
41.7806000000001 0.390900000000061 4.74309999999995
39.1397000000004 10.5604000000001 -10.7264000000001
43.7573581785552 10.6357313002889 -6.84391243482733

46.1384000000002 9.35350000000011 -9.55490000000003
43.9127000000003 -2.17779999999989 -10.3625
51.7856000000004 10.0286000000001 -10.416
57.2525000000002 4.64480000000009 -10.6075
61.9457000000002 11.3640000000001 -10.9904
66.1562 12.4079000000001 -10.0965
65.8959000000003 18.6383000000001 -11.7265000000001
71.4475 20.1328000000001 -5.09110000000003
72.0767000000001 10.4951000000001 -8.30890000000004
71.5578000000001 14.9469000000001 -11.0978
5.04669999999945 2.66840000000013 1.08399999999992
5.95109999999954 4.43890000000011 0.515699999999917
22.0517999999998 13.4423 -4.53550000000002
24.6438999999997 12.6722999999999 -5.15200000000001
29.8483999999999 11.806 -5.1216
28.6938999999998 10.6760999999999 -5.9427
35.0621999999998 21.0911 5.01319999999996
43.9851000000003 13.1075000000001 -7.29220000000003
45.7202000000002 16.6029000000001 -9.67030000000003
37.7743 24.3182 -10.0526
50.9908000000002 18.6877 -10.6538
52.8162000000002 25.702 -10.1612
59.4792000000003 23.2447000000001 -11.2697000000001
64.2464000000001 25.0173000000001 -9.92610000000003
67.6071000000002 29.4801000000001 -8.20250000000004
69.4464000000001 25.9758000000001 -11.1347
4.63769999999955 2.26820000000015 7.54949999999988
4.68069999999944 4.90430000000012 6.71959999999991
23.8543999999998 11.0354 7.76079999999993
27.0982999999998 16.0502 -3.8939
25.3706999999998 16.6967 -6.79880000000001
30.1647 8.39190000000006 9.27059999999994
51.8453000000001 15.2275000000001 9.79809999999997
59.3299 16.7426000000001 8.56729999999996
59.0112 32.1379000000001 -4.36310000000003
66.8762000000001 18.9242000000001 4.44249999999996
66.8030000000001 27.2495000000001 -6.14670000000004
4.58689999999958 1.20550000000017 7.42329999999987
6.59379999999989 -1.59419999999979 7.03129999999985
26.3229 3.16240000000009 7.9719999999999
31.5797000000004 0.0123000000000926 -4.09710000000005
30.6375000000002 -1.44849999999996 -6.01580000000003
67.3997000000001 4.05970000000013 -5.03230000000004
71.5149 12.4657000000001 -5.92660000000003
ID=PHAtriUNKCONKMMA6M0583

LM3=54
5.5191 -0.1387 1.5289
6.9012 -0.2413 0.6355
23.4709 0.6731 -3.1129
24.3239 1.4572 -3.083
25.2245 7.8599 -3.4248
28.8089 4.988 -3.5003
28.2877 5.6169 -4.3893

38.2601 0.473 5.7351
35.459 10.8494 -8.4935
39.6617 9.932 -4.9195
42.1278 9.1541 -7.4863
39.8149 -0.1074 -7.5838
47.7257 9.4726 -8.2922
52.0908 5.1726 -8.1017
56.8632 11.0601 -8.993
60.5024 12.4276 -7.8548
60.2092 18.0754 -9.6297
65.4831 20.4582 -1.4559
65.7719 10.6674 -5.6192
64.4116 14.5704 -8.8111
4.5234 3.3829 1.4913
5.3229 4.4699 1.0576
19.5056 13.0357 -3.3917
21.282 12.2628 -3.3823
26.6859 11.4883 -3.5218
26.3826 11.0014 -4.7172
30.8667 20.1964 5.5627
38.3773 13.7592 -5.3542
40.1968 16.2434 -7.6853
32.4299 22.4607 -7.4938
45.0929 18.5552 -8.5711
46.2796 24.9037 -8.234
53.144 23.2246 -9.5139
56.9117 23.56 -8.0154
60.0252 28.1805 -6.3144
61.3599 24.4439 -8.8581
5.0313 2.0879 8.6211
4.8932 5.0166 6.9124
20.1734 10.7401 8.7209
24.4249 14.2654 -1.4952
22.9485 16.076 -4.3901
29.5491 8.9584 10.3712
47.4598 14.4902 12.4131
53.6888 16.2111 11.2647
53.6737 31.3989 -3.2304
63.3591 19.7258 4.9173
60.595 26.7936 -4.4297
5.0416 1.7186 8.4782
7.2779 -1.2387 7.2045
22.6155 2.3724 8.5195
28.5089 1.8943 -1.185
28.3587 -0.1787 -4.076
62.6279 5.3569 -3.3958
65.3713 12.962 -4.2274
ID=PHAtriUNKCONKMMA2M0133

LM3=54
4.6239 -0.6967 1.2269
6.2047 -0.9107 0.2773
24.1773 0.6264 -4.4914
25.6292 1.9865 -4.8821

27.4578 8.9585 -4.7276
30.5979 5.8321 -4.9949
29.5311 6.415 -6.2852
38.0413 0.3912 6.4952
36.674 11.6528 -8.7301
42.3728 11.6247 -4.8458
44.377 10.1166 -6.5754
42.1968 -0.8449 -8.1029
50.107 10.4197 -8.2967
54.9685 5.9196 -7.8745
60.1084 11.4509 -8.899
64.898 12.3007 -7.0301
64.1745 19.4932 -9.3802
69.3199 20.7036 -1.0578
69.5258 11.129 -6.241
69.9915 15.2457 -8.5886
4.2631 4.9127 1.0258
5.0065 6.0815 0.2511
20.9603 14.1216 -3.982
23.2092 13.7504 -4.4798
29.0919 12.9102 -3.7211
28.6405 11.8203 -5.6607
32.6037 20.5636 7.3261
41.8705 14.3756 -4.4868
43.4618 16.8261 -7.1727
35.581 24.6171 -7.702
48.3707 19.3444 -7.7269
50.7985 26.7939 -7.7876
56.6101 24.1921 -9.0889
61.6046 26.0601 -6.502
64.7827 29.4612 -5.7811
67.2867 26.2036 -8.9348
5.4621 1.4879 8.9699
6.3295 4.6402 7.9928
20.8566 11.1139 8.7069
27.0986 14.5617 -1.6716
24.3444 15.6989 -5.9316
33.3443 8.972 11.9701
50.1413 14.443 12.1513
57.5643 16.5297 10.7014
58.5693 32.7003 -2.1122
65.5243 19.5589 6.4185
64.8333 28.126 -4.9786
5.5874 0.4004 8.9625
8.2686 -2.1007 7.8376
23.9172 1.2126 8.4883
30.7493 2.2801 -2.4303
29.401 -0.3174 -6.1768
67.0754 5.2952 -2.2676
69.9196 13.9954 -4.4414
ID=PHAtriUNKCONKMMA2M0135

LM3=54
5.0142 -0.8648 2.9389

5.7874 -1.1397 2.2032
24.5729 0.0057 -4.4905
25.0446 1.1351 -4.5403
27.065 8.1219 -6.3099
30.9649 5.2879 -5.9949
29.4691 5.7652 -7.2204
41.3618 0.9011 3.6205
36.2958 10.9245 -11.4026
43.0968 10.9832 -6.8365
44.843 9.1455 -9.437
42.6028 -0.8127 -9.7318
50.1023 9.9224 -10.6377
55.5922 5.954 -9.3363
61.3726 11.1994 -11.1217
65.281 12.1163 -9.4902
63.9787 18.6172 -11.4062
70.9309 20.4819 -4.6927
70.6978 11.2142 -8.0396
70.9695 14.3486 -11.3378
3.4727 3.5927 2.7474
3.9218 3.9872 2.4054
20.8183 13.4331 -4.4926
21.6708 12.312 -4.6132
29.0033 10.9748 -5.7422
27.6468 10.4974 -7.4047
33.6713 20.2348 3.971
42.1204 13.005 -6.4229
43.1669 16.3491 -8.8466
35.3706 23.2353 -9.7006
47.3238 18.0271 -10.3356
49.7221 24.6934 -9.6497
57.6119 23.9559 -10.704
61.3889 25.8807 -9.1545
65.0284 28.8971 -7.7846
67.6778 26.4009 -10.4638
4.6364 0.7908 8.142
4.9263 3.729 7.9972
22.887 9.5988 8.8679
26.3885 14.3012 -4.208
23.8029 15.3104 -5.9915
32.2015 8.6351 9.5802
52.1632 14.1456 10.8033
51.863 14.2413 10.7957
59.8848 32.0793 -5.0688
68.4578 19.4277 3.9767
67.3721 26.869 -5.9184
4.9247 -0.5075 8.2179
6.534 -2.1177 7.785
25.1909 3.2693 8.6529
31.1319 1.3027 -4.232
29.2635 -1.2337 -5.3183
68.3793 6.3057 -6.4966
71.2956 13.3398 -6.4131
ID=PHAtriUNKCONKMMA2M0148

LM3=54
4.7302 -0.3012 0.5749
5.6026 -1.6047 -0.3429
23.402 1.0036 -4.9992
25.2155 1.6683 -5.5343
26.1393 8.3095 -5.3395
29.4185 5.3537 -4.931
28.5516 6.1319 -6.2929
39.1908 0.42 4.773
34.6562 11.1907 -9.0517
40.5831 10.5058 -5.1538
43.1573 9.6871 -7.1805
40.1418 -0.2724 -8.8172
48.8866 9.6757 -7.9545
52.9476 4.6947 -8.107
56.933 10.9571 -9.0041
61.411 12.0976 -7.8708
61.1954 18.2205 -9.5057
66.8101 20.0433 -0.1547
66.1097 11.2094 -7.0901
66.5244 14.3655 -8.7592
3.4757 3.9966 0.3231
3.5146 4.8887 -0.5341
20.0971 13.3355 -5.4018
21.4778 13.047 -5.2773
27.3187 12.1804 -5.1293
27.6076 11.3681 -6.8517
32.6542 21.1965 4.4532
39.098 13.6782 -5.267
41.305 15.8297 -6.9954
33.3672 23.0725 -8.4436
45.6629 18.7696 -7.5467
47.1494 25.4778 -8.1528
54.2169 23.3933 -9.2795
58.2762 24.1873 -7.6241
61.7848 27.343 -6.4426
64.0293 24.7268 -8.321
3.463 1.1551 7.5929
3.272 4.3538 6.9882
19.0266 10.2161 7.4064
25.1863 15.8289 -3.2823
22.9446 16.2525 -5.9786
28.1946 8.8287 8.9492
47.1027 14.4051 11.6182
54.3924 16.5352 10.5017
54.8335 31.453 -1.7159
63.0231 19.8873 6.3215
62.4111 26.9037 -4.9936
3.4348 0.8811 7.0309
5.192 -1.5828 6.6319
21.5361 2.7461 7.3371
29.4827 1.174 -3.4631
28.1807 0.2318 -6.0284

62.9486 4.9812 -2.3081
66.4403 12.8913 -5.1264
ID=PHAtriUNKCONKMMA2M0152

LM3=54
4.63550000000347 -0.738999999999027 1.51389999999994
6.75240000000345 -1.30969999999914 0.849699999999918
26.1381000000022 -0.696899999999276 -4.36420000000008
28.3036000000023 0.765500000000803 -4.8882000000001
26.8149000000021 7.42030000000072 -4.65280000000008
31.873300000002 4.66680000000067 -4.64970000000006
30.3999000000021 4.35240000000072 -5.51180000000007
41.2736000000021 -0.492499999999419 5.94419999999998
37.6333000000018 10.5941000000005 -10.6658
44.7496000000016 10.0709000000004 -5.99199999999996
46.2182000000018 9.02840000000043 -7.75029999999995
43.0823000000022 -1.40149999999944 -9.68999999999999
52.0418000000018 9.36130000000033 -8.69039999999991
58.2565000000019 4.52280000000032 -8.65849999999991
63.0085000000017 10.4548000000001 -8.74399999999985
66.4890000000017 11.6750000000001 -8.27539999999978
66.3512000000016 19.0653 -10.5801999999997
72.2789000000016 20.5107 -4.38249999999973
72.1439000000018 10.4692 -6.89809999999976
70.9817000000018 14.2753 -10.0870999999998
4.07600000000349 2.21270000000091 0.678699999999858
5.85750000000354 4.20030000000093 -0.259700000000138
22.8029000000022 14.0162000000007 -4.36280000000007
24.8186000000021 13.4408000000007 -5.11220000000007
29.5268000000019 11.9803000000007 -4.24040000000006
28.277100000002 11.3510000000007 -5.59780000000006
35.3829000000016 21.3716000000004 5.77760000000001
43.2902000000016 14.0718000000004 -5.60929999999997
44.5170000000016 16.1016000000004 -7.47529999999996
36.5347000000019 23.5755000000005 -9.79649999999997
50.1366000000015 19.1468000000003 -8.97279999999991
52.5457000000015 25.6849000000002 -8.84599999999987
59.6793000000014 23.8523 -9.27619999999981
62.4603000000015 24.5401 -8.48649999999977
65.9062000000015 28.6133999999999 -7.14179999999973
67.4779000000017 24.7017 -10.1462999999997
3.66710000000352 1.2180000000009 8.07289999999989
3.3282000000035 4.47950000000089 6.60619999999987
22.8700000000023 12.2293000000007 9.0360999999999
29.1197000000018 16.7326000000007 -3.98970000000006
27.7782000000021 18.8333000000007 -6.73970000000005
32.969000000002 8.94280000000057 12.0315999999999
54.9669000000018 15.3283000000003 12.4072000000001
62.5000000000019 17.3299000000002 10.5995000000002
60.8554000000016 33.2433 -3.01099999999978
71.1603000000018 20.2943 4.28610000000025
67.3339000000015 27.4247999999999 -5.36519999999974
2.92050000000349 0.652500000000875 8.01909999999987
5.3189000000358 -3.2508999999991 6.88499999999986

25.9889000000024 0.795100000000698 8.9806999999999
33.9499000000022 -0.117399999999297 -3.60560000000005
33.6645000000023 -1.31829999999928 -5.91020000000007
70.2449000000022 4.5906000000002 -3.8482999999998
72.3833000000019 13.2923 -5.60599999999975
ID=PHAtriUNKCONKMMA0M0154

LM3=54
4.1587 -0.19 1.0276
6.1279 -0.9106 0.1681
23.1669 -0.1151 -4.275
23.0122 0.6996 -4.215
23.9809 7.1907 -4.4262
27.5908 4.2161 -4.8652
26.887 4.8012 -6.1833
37.5011 0.2573 4.5153
32.6514 10.122 -8.9176
37.6752 9.845 -5.8985
39.0204 8.6211 -8.1673
37.3854 -0.4841 -8.6462
44.7287 9.8768 -9.559
49.6079 5.3475 -10.0906
54.4569 10.6175 -12.086
57.4249 11.5238 -11.3127
57.1654 18.4565 -13.5875
62.6808 19.9758 -5.8212
63.6194 11.0228 -10.5197
61.2713 14.633 -13.4632
3.2445 2.2703 1.0461
4.2121 4.6669 0.4398
18.8103 12.131 -3.6784
19.9121 12.1826 -3.9565
25.1194 11.7021 -4.8306
24.783 10.4675 -5.8101
30.7196 19.9654 4.6425
37.4047 13.1244 -6.2963
37.3755 14.9228 -8.0226
30.8303 22.1277 -7.9429
42.7052 17.8941 -9.3255
44.2661 23.8181 -9.8469
51.2934 22.7999 -12.0849
53.8998 24.3293 -11.4311
58.869 28.2217 -9.8243
59.4874 24.2138 -13.0388
4.0796 0.955 7.6141
4.3386 4.0565 6.9913
17.9672 8.9332 8.0177
22.8558 13.8351 -2.3951
21.446 15.7441 -4.6633
27.3484 7.9161 9.7021
46.6414 14.5394 10.6999
51.9954 16.3791 8.9519
52.6577 31.0515 -4.6384
61.7222 19.5114 1.9161

58.78 26.867 -8.2338
3.9897 0.3864 7.3926
6.5646 -1.5104 7.0157
20.3069 1.3463 7.8251
26.8673 0.9538 -2.2154
26.5981 -0.7646 -4.9438
61.3916 5.9644 -4.5839
63.659 12.1394 -8.917
ID=PHAtriUNKCONKMMA2M0156

LM3=54
3.53870000000042 -1.40359999999998 0.369699999999664
4.11669999999983 -1.31579999999994 0.432299999999801
22.9588999999994 -0.458600000000062 -3.77760000000009
24.2980999999993 0.271299999999865 -3.98130000000008
23.9063999999996 6.12309999999994 -3.65040000000004
28.3970999999993 4.13049999999986 -3.62169999999997
27.690199999999 4.40949999999978 -4.72279999999998
36.3977999999992 -0.0189000000001193 6.39899999999998
34.1871999999999 9.29540000000002 -8.39350000000003
38.7550999999998 9.42429999999995 -3.5518
41.5028426117053 7.98515966088077 -6.40367333810124
38.8436999999995 -1.19000000000006 -7.69750000000002
46.4685000000001 8.15430000000005 -7.35400000000004
53.1360000000001 4.39640000000004 -8.09470000000002
57.3066000000001 8.83060000000002 -8.50590000000004
61.1761000000001 10.1279 -7.61590000000003
61.1543000000001 16.5819 -9.33590000000004
67.9446000000001 18.181 -2.54130000000007
66.2824999999999 8.4864 -6.34960000000004
66.9837000000001 12.184 -8.69110000000005
2.89760000000024 1.7527 0.595499999999739
3.85330000000016 2.6769 0.624099999999759
19.9144999999998 11.6218999999999 -3.78120000000005
21.9796999999997 11.9888999999999 -4.06890000000005
27.2782999999998 10.9536 -3.50870000000003
26.9165999999997 9.93509999999994 -4.60580000000003
30.3994999999999 18.7369 6.58089999999997
38.0888999999999 12.3855 -3.74779999999999
40.0026000000001 14.577 -6.59650000000001
32.9230000000001 21.2826 -7.21460000000004
43.8946000000001 17.5807 -7.12650000000002
47.2485000000002 23.5854 -7.40530000000002
54.0572000000002 21.7779 -8.58090000000003
57.8408000000002 23.2637 -7.11100000000005
60.6809000000001 26.4615 -5.84940000000005
63.7794000000002 23.6392 -8.32640000000006
3.19020000000012 1.84949999999998 7.32909999999971
3.76150000000016 4.40999999999997 6.50719999999974
17.7076999999997 9.15499999999992 8.42179999999991
24.3619999999999 14.8413 -3.90690000000002
23.1364999999999 15.2346 -5.77720000000004
25.8042999999996 7.76079999999991 10.1765999999999
49.3065999999997 13.2926 12.2592999999999

55.9325 14.8552 10.3192
56.1359000000002 29.9429 -1.85930000000004
65.1252 17.9025 4.75499999999994
62.9799000000001 25.5642 -4.55300000000005
2.95180000000011 1.00539999999999 7.33629999999972
5.42300000000002 -0.986100000000001 6.69619999999973
19.5135999999995 1.52719999999992 7.9409999999999
27.8821999999998 0.866999999999984 -3.77430000000007
27.5066999999996 -0.652600000000048 -4.99160000000008
62.9827999999998 2.94429999999999 -2.23740000000004
67.2163 11.3253 -4.95340000000005
ID=PHAtriUNKCONKMMA2M0159

LM3=54
3.63329999999885 0.964499999999777 -0.0703999999999367
5.26280000000065 -0.0408999999995362 0.531999999999868
21.0466000000002 1.02060000000025 -3.69280000000011
22.865900000001 2.10800000000059 -3.6691000000002
22.7053999999993 7.51179999999999 -4.05119999999998
26.9392000000001 5.42740000000015 -4.48450000000009
25.7804999999994 5.47740000000008 -5.25769999999999
33.3035999999991 0.440499999999904 6.64679999999998
33.5550999999993 10.7293999999999 -10.3918
37.4197480137526 11.0699774376843 -5.38735292687759
39.5850520017996 9.63354023370817 -7.89898022867803
36.7353999999992 -0.414500000000067 -8.04910000000008
44.8383999999989 9.77869999999982 -9.84690000000007
50.1995999999988 5.05019999999979 -10.0703000000001
53.9917999999981 10.5133999999995 -10.8845
57.764099999998 12.4625999999995 -10.5433
56.8579999999979 19.1278999999995 -13.2842
64.2023999999976 21.0852999999993 -8.22489999999999
61.8565999999978 10.3728999999994 -10.7756
61.4242999999977 14.7922999999994 -13.3578
3.55329999999896 2.1633999999998 1.6201000000001
4.07829999999912 4.21409999999985 0.590900000000079
18.1431999999993 12.6284999999999 -4.03159999999997
19.6175999999993 12.2211999999999 -4.13279999999997
25.4065999999993 11.2992999999999 -4.64069999999999
24.1689999999993 10.3317999999999 -5.89599999999997
27.9035999999988 20.9743999999997 5.93930000000002
36.4471514949168 13.2838276508612 -5.38855539634718
36.8149999999988 15.5592999999997 -7.8369
28.9389999999986 21.8553999999997 -8.30529999999995
41.3124999999985 18.1716999999996 -10.2088
42.7433999999983 25.0070999999996 -10.4303
49.203599999998 24.1282999999995 -11.3776
53.1721999999978 25.0429999999994 -10.7726
55.1736999999975 29.0467999999993 -10.9264
57.2680999999976 25.4747999999994 -13.3437
3.056599999999 1.42349999999983 7.4431000000001
3.6833999999989 4.09459999999977 6.84540000000012
18.0628999999994 9.89309999999995 9.46199999999998
22.5893999999993 15.4544999999999 -2.26829999999998

21.6834999999991 16.6704999999999 -4.76129999999995
25.6783999999993 8.46059999999993 10.654
45.9652999999984 15.0180999999997 11.375
54.724999999998 18.6678999999995 7.5539
51.2939999999976 33.0837999999993 -6.48529999999997
64.0056999999976 21.3376999999994 -0.465299999999988
57.8043999999975 28.4847999999993 -9.35059999999996
3.23779999999904 0.84389999999985 6.9709000000001
4.7762999999994 -0.987800000000014 6.87820000000003
20.7389999999998 2.64770000000012 9.50069999999993
26.8353000000001 0.832300000000184 -1.58010000000013
27.235 -0.428999999999806 -4.27650000000011
61.327899999998 4.72459999999953 -5.33910000000003
63.6449999999977 12.3257999999994 -9.2153
ID=PHAtriUNKCONKMMA2M0161

LM3=54
5.27800000000026 0.396900000000484 1.56909999999997
6.30310000000043 -0.678599999999398 1.18769999999998
23.9793000000006 0.535200000000408 -3.01830000000008
27.0115000000005 0.938900000000357 -3.68540000000005
27.3530000000006 7.76860000000031 -3.79310000000006
30.6226000000007 4.93470000000036 -4.24380000000006
29.5729000000006 5.94130000000035 -5.75450000000004
36.3124000000003 -0.205499999999958 6.07759999999998
35.8766000000006 10.7111000000004 -8.05050000000001
41.6621000000005 10.5200000000004 -4.0884
44.5129000000004 8.75370000000041 -7.17289999999997
41.7569000000004 0.140800000000451 -8.39339999999997
49.4197000000003 9.80800000000045 -7.88559999999992
55.1632000000003 4.96610000000053 -8.10339999999992
59.8411999999999 10.3381000000005 -9.75009999999985
64.4991999999998 11.1667000000005 -8.09489999999982
64.3271999999997 18.0198000000005 -11.2319999999998
70.0666999999997 19.3291000000006 -4.7763999999998
69.2997999999997 11.0384000000006 -8.01609999999981
68.4127999999997 14.5520000000006 -10.4617999999998
4.71836951617018 3.36689001957214 1.76474735030479
5.40440000000036 4.76690000000057 1.34879999999996
21.1303000000007 13.0420000000003 -3.13900000000007
23.7964000000006 13.3563000000003 -3.44400000000006
29.1566000000007 11.7786000000003 -4.17170000000006
28.1660000000007 10.8723000000003 -5.45570000000006
32.2273000000003 18.9960000000003 6.43319999999999
40.8180000000005 13.1688000000004 -4.01719999999999
42.0610081313977 15.5314664540681 -6.09562274866821
35.7410000000005 22.3273000000004 -8.24059999999998
47.1387000000002 18.1679000000004 -7.2804999999999
49.0940000000001 25.0189000000004 -7.81179999999988
56.2468999999998 22.6039000000004 -9.73479999999979
60.6545999999997 24.3392000000005 -7.95339999999979
64.2174999999997 27.2022000000006 -7.59799999999978
65.9936999999998 23.4763000000006 -10.4833999999998
4.4891000000003 0.877300000000622 8.29099999999996

5.96010000000025 3.7537000000006 7.37479999999997
20.1963000000004 10.0299000000004 8.53139999999992
27.4671000000006 14.6594000000002 -2.68030000000005
24.7399000000006 16.0621000000003 -4.46940000000005
27.6676000000004 7.41190000000038 10.8230999999999
50.6928 14.1733000000004 12.4084000000001
59.8259999999997 16.5534000000005 9.04410000000013
59.1636999999997 30.8502000000005 -3.70639999999979
67.6379999999996 19.0214000000006 4.41430000000019
66.0060999999997 26.0288000000006 -5.93349999999978
4.79010000000032 -0.235399999999397 7.89539999999995
7.48040000000037 -1.58219999999937 7.02629999999994
22.5826000000003 1.44000000000042 8.32079999999993
30.8019000000005 1.85830000000036 -3.00280000000005
29.7056000000005 -0.1238999999996 -5.10850000000004
66.7058999999997 5.15900000000059 -3.93339999999984
70.2876999999996 14.1816000000006 -6.1706999999998
ID=PHAtriUNKCONKMMA2M0162

LM3=54
15.444300000005 3.37150000000029 -1.71100000000015
19.924900000002 -5.33780000000034 -4.10419999999973
51.7411000000021 -7.30040000000024 -3.91459999999964
90.0789000000014 1.15380000000045 -16.7737
71.4750000000001 15.8765999999997 -16.0555999999997
96.5214999999994 7.09469999999969 -19.8578999999996
108.805899999999 10.9352999999995 -17.2810999999997
111.694199999999 -0.963700000000495 9.30110000000051
117.270200000001 26.5743999999999 -26.8667999999998
116.6325 14.1030999999997 -15.1752999999997
117.531899999999 10.5529999999995 -17.0547999999996
120.179999999999 -13.4661000000006 -17.2485999999995
134.3863 15.7187999999996 -21.7823999999996
146.2554 10.1218999999996 -22.4750999999996
154.2932 19.7912999999996 -25.0969999999995
158.4726 19.6428999999995 -30.7062999999995
153.1172 33.5601999999996 -34.0878999999996
169.330700000001 36.8137999999995 -21.2682999999995
165.1494 10.2985999999995 -27.6486999999994
169.6689 24.9498999999995 -36.1220999999994
15.444300000005 3.37150000000029 -1.71100000000015
15.8836000000042 14.0192000000002 -3.17779999999989
43.6971000000023 28.9519000000001 -1.79849999999983
82.4790000000008 37.2652 -15.7043999999998
89.3007000000006 35.6691 -18.1387999999998
100.677300000001 35.8168999999999 -16.8837999999998
102.285900000001 46.1558 10.2373000000003
116.397200000001 34.4576999999999 -12.8423999999998
111.774600000001 39.8833999999999 -15.7065999999998
104.222900000001 62.2722 -13.5660999999997
127.608600000001 42.3570999999999 -20.8441999999997
136.862700000001 52.3200999999997 -21.5221999999997
147.461100000001 46.7018999999997 -25.4027999999996
151.723600000001 48.6771999999997 -30.4586999999996

153.775100000001 59.9497999999995 -27.3603999999996
164.167200000001 48.1413999999996 -35.2425999999996
-12.3115633740698 -2.5696841143073 13.9989619082911
-5.87939999999644 8.55969999999961 12.0508000000004
53.3503000000022 23.177 16.4533000000003
90.4514000000009 43.2924 -9.8873999999998
86.260600000001 47.8304 -13.7173999999998
54.6662000000021 10.8951999999999 19.6644000000003
123.8394 26.2420999999997 21.1816000000004
157.098800000001 33.4954999999996 11.2298000000005
165.444400000001 35.0366999999995 7.89840000000051
153.454000000001 62.3279999999997 -5.88259999999958
156.117900000001 54.5800999999995 -20.1103999999996
-11.8758683044694 -3.82130424520378 13.8115673233138
-1.41909999999662 -11.1607000000007 10.7528000000005
58.0979000000019 0.0891999999998295 15.1406000000003
100.410899999999 -1.88150000000035 -10.8982999999997
97.9088999999999 -9.77770000000021 -15.5373999999997
166.0923 5.62239999999947 -5.55429999999944
165.1623 14.5278999999995 -22.1609999999994
ID=PRImaxUNKUNKMfNB37971*

LM3=54
16.4624 3.4617 -1.9402
20.8685 -5.7177 -3.4527
56.0368 -5.5945 -4.1225
88.6654 1.437 -15.6974
73.4549 15.1958 -14.3834
96.3678 6.3513 -17.897
107.4304 8.9802 -16.8009
110.6267 -0.5136 7.6852
112.9237 23.3086 -23.5187
120.6838 15.1004 -13.4188
119.6689 11.1819 -15.8348
118.2438 -13.3692 -16.1184
132.4434 13.5072 -20.5453
145.8177 7.9925 -22.737
154.466 17.304 -25.0267
159.4852 17.7677 -30.7364
155.1961 30.7875 -33.8244
170.068 33.983 -21.8965
165.6637 7.2129 -27.081
170.5871 21.8528 -36.1514
16.4624 3.4617 -1.9402
17.2041 13.5329 -3.2189
50.1295 27.4823 -5.3733
82.3021 34.7447 -15.8074
90.6722 32.8001 -16.5201
104.1531 34.1256 -16.2916
103.0411 44.8726 9.211
117.2748 33.7274 -12.9504
113.7194 37.2109 -15.7226
104.746 59.2457 -16.0606
128.5016 40.1606 -20.6936

138.4375 49.148 -22.5171
150.161 43.8092 -24.6654
154.3708 43.8952 -30.5698
157.5225 57.0049 -27.7436
167.3855 45.2428 -34.7217
-13.2316 -2.5601 14.2422
-7.4606 7.2844 11.4988
56.6348 23.1103 17.9575
92.8233 42.5407 -9.4995
87.5814 47.7232 -16.2099
55.7035 11.8825 19.7085
122.6358 25.9058 21.4808
157.7228 32.1032 11.9821
165.9416 33.1554 7.9659
153.9189 59.9135 -6.001
157.8204 54.258 -22.2133
-13.5269 -3.6874 13.4798
-4.7697 -10.2725 11.5283
59.1275 1.3787 17.221
99.8337 -0.7001 -10.7379
98.1584 -7.8875 -15.3268
163.5516 3.411 -6.8479
165.7594 9.9023 -22.8434
ID=PRImaxUNKBRA47528*

LM3=54
19.7258000000028 3.98619999999997 -2.08240000000049
22.0897000000029 -5.62630000000002 -4.1707000000005
56.122200000002 -6.51440000000004 -3.43090000000024
91.0876000000013 1.19289999999989 -15.3037
72.8809000000012 15.0783999999999 -13.9997000000001
95.661300000001 5.73319999999991 -16.3278
111.8777 10.4033999999998 -14.7881999999999
116.305600000001 1.18069999999998 9.16249999999995
113.193300000001 23.6653 -23.944
121.135500000001 16.6671999999999 -11.6822999999999
119.664500000001 11.3027999999999 -14.6251999999999
118.702000000002 -14.5530999999999 -14.4834
132.105100000001 13.5540000000001 -19.6566999999999
146.768900000001 8.55460000000008 -23.1405
154.321400000002 17.8650000000002 -26.3658
155.703800000001 17.0184000000002 -32.978
150.746700000001 31.1897000000003 -36.0062000000001
170.819800000002 35.6947000000003 -29.2032000000002
166.204600000002 8.51110000000019 -29.7392000000001
167.683000000002 22.8402000000003 -41.3814000000001
19.7258000000028 3.98619999999997 -2.08240000000049
18.6459000000023 13.7698999999999 -3.84270000000042
49.1661000000016 29.1983999999999 -3.71430000000034
83.9605000000006 35.1544999999999 -15.1915000000001
89.2621000000007 32.9594999999999 -15.1192000000001
106.5011 35.4613999999999 -14.9959000000001
105.569100000001 47.4609 9.57359999999976
116.7975 35.2032 -11.5707000000001

113.3007 38.3004999999998 -15.2377
99.8782000000006 60.8357 -15.7058000000002
126.8736 41.9978000000001 -19.8650000000001
137.753000000001 51.2176000000003 -23.5709000000003
147.428400000001 45.2962000000003 -26.6952000000002
150.680700000002 46.4807000000004 -33.0893000000003
156.663800000002 56.7873000000005 -28.7362000000004
162.481000000002 45.0467000000004 -41.1749000000003
-10.7964722799504 -1.41653548839967 14.5985626884094
-4.27489999999656 9.64030000000001 11.6916999999993
55.7721000000019 24.2387 19.1251999999997
93.2748828849356 43.0999732982238 -13.3210238860777
88.5746000000004 47.8120999999999 -15.4179000000001
57.5321000000018 12.5582999999999 22.6320999999997
128.251700000001 28.4124000000001 22.7942999999998
164.164400000002 34.3727000000003 9.57859999999967
171.863200000002 36.9837000000004 4.24209999999964
156.915000000001 62.9895000000002 -12.5555000000003
159.861500000002 52.5079000000004 -26.0398000000003
-10.3852515344239 -2.69551591783511 14.4973610012854
-0.403599999996681 -10.1597 12.1052999999994
60.6321000000018 1.1284999999999 18.7874999999998
103.640118391698 2.03316110879026 -12.1440133215578
101.076900000002 -8.37560000000008 -14.7608
169.144500000002 6.42530000000029 -10.8799000000001
166.901400000002 14.0147000000002 -26.6551000000001
ID=PRImaxUNKUNKMfNB******

LM3=54
31.2615 5.8882 -13.041
36.2552 -4.8736 -13.7193
71.9539 -6.7682 -7.278
104.9392 0.4803 -13.7978
92.4512 18.1691 -15.9277
110.6089 6.4803 -14.0345
126.0443 10.9337 -9.6269
125.2175 0.1756 17.7976
131.8963 27.0699 -18.9824
134.4092 16.5768 -3.6901
135.1343 11.5864 -7.0172
138.4447 -14.6443 -5.9924
150.1988 15.4763 -9.6009
164.3156 10.14 -7.8737
172.1529 20.3694 -9.7751
177.6868 21.0288 -15.2553
172.3558 35.0706 -16.9354
188.9841 39.0283 -5.1204
185.8999 11.8844 -12.0057
180.7682 28.5512 -22.3235
31.2615 5.8882 -13.041
31.474 16.9397 -14.9035
63.3045 33.0644 -9.1242
97.3157 40.0531 -14.7641
105.4312 37.2438 -14.6176

117.2657 38.6018 -10.5188
113.1214 49.2497 16.4653
131.5902 36.7844 -3.4951
128.078 42.0294 -6.5088
120.8345 67.6133 -7.3171
144.2451 45.349 -9.5129
153.9021 55.0304 -7.3911
165.652 50.3946 -9.723
171.1032 50.3016 -15.6424
173.8431 63.945 -12.4037
176.7821 46.6547 -21.9502
-0.4215 0.3705 -0.3796
5.0784 10.6347 -2.0411
66.2324 25.8937 15.1437
109.2372 44.8769 -9.0755
100.7394 55.9106 -10.9991
63.3014 13.4929 16.4961
132.9994 28.6965 31.6719
174.4162 36.8684 30.0287
184.8133 39.6629 26.1368
170.5101 68.2898 11.9627
176.2877 57.8112 -5.3482
0.5235 -0.1749 0.0172
10.1895 -7.2278 -1.8541
71.2935 1.8669 15.2451
118.7426 3.2161 -8.2039
115.3381 -10.85 -10.1507
182.8587 8.1875 12.1832
184.7568 20.9464 -4.605
ID=PRImaxUNKUNKAMNH130122

LM3=54
28.4413000000016 5.9783999999996 -12.6757999999999
31.265800000001 -3.59500000000059 -12.9042000000003
66.5059999999982 -5.64390000000088 -6.93189999999993
97.1485999999996 0.515699999999656 -12.0078999999999
81.4973999999994 17.3918999999997 -14.4805
104.109699999998 5.86319999999924 -11.8667999999999
116.361600000002 10.2091000000002 -7.49200000000012
119.1888 0.943099999999991 18.1196
123.616800000001 24.9883999999998 -13.9553000000001
126.091500000001 14.9028000000001 -3.0290000000001
127.030800000001 11.2537000000002 -5.56420000000013
128.254600000001 -13.9134 -5.7194000000001
140.086900000001 14.0953 -8.84510000000009
153.445200000001 9.04149999999995 -8.35430000000006
162.175700000001 17.7388 -10.3564000000001
166.199400000001 18.8312 -14.5947000000001
163.764500000001 32.3205999999999 -17.2842000000001
176.077100000001 34.7690999999999 -4.23900000000006
173.548100000001 10.4444 -10.1581000000001
172.642200000001 24.2054 -22.2613000000001
28.4413000000016 5.9783999999996 -12.6757999999999
27.7510000000015 15.6131999999993 -12.9599999999999

56.2566000000001 31.1939999999994 -6.49219999999994
88.7041000000001 37.3422999999997 -11.5665
97.6987000000003 35.3985999999998 -11.5728
110.3762 35.3540999999997 -7.563
106.7475 44.4778999999997 18.7166
121.587800000001 34.6526999999998 -2.84290000000004
120.644200000001 38.6373999999998 -5.27690000000005
111.9964 62.2899999999996 -5.2776
134.252100000001 40.7326999999999 -8.83970000000008
143.973800000001 49.8573999999999 -8.06200000000006
156.010000000001 44.9101999999999 -10.7266000000001
160.713800000001 46.9629999999999 -15.0022000000001
164.978300000001 56.7208999999999 -10.4569000000001
168.448900000001 43.0859999999999 -22.3250000000001
-0.522099999999982 0.4431999999992 0.157400000000227
6.48450000000162 11.9048999999994 -2.25139999999988
59.9661999999998 23.7725999999994 14.7648000000001
100.6461 40.8667999999997 -6.17719999999997
95.0027000000003 50.2483999999996 -8.23729999999997
57.9881999999996 11.3600999999994 17.0296000000001
123.974800000001 24.7474999999999 31.6447
162.166200000001 31.0507999999999 27.5784999999999
176.042400000001 35.5070999999999 22.9265
161.193500000001 60.9127999999999 8.69449999999998
167.987300000001 51.4871999999999 -5.16320000000005
-0.0112000000001165 -0.811400000000778 0.0390000000002154
9.59620000000094 -8.42560000000041 -2.99999999999981
64.7402999999993 0.1116999999994 13.8449000000001
105.7549 -1.44050000000028 -6.18740000000003
106.2966 -9.48220000000029 -9.73130000000003
171.616800000001 4.16899999999999 8.32089999999993
174.847800000001 16.3674 -5.53720000000009
ID=PRImaxUNKGUYAMNH64120*

LM3=54
28.7274000000004 4.99869999999992 -12.3546999999998
30.0686000000015 -4.42310000000032 -14.5898
62.9757000000017 -4.98889999999964 -10.6333000000004
92.4350999999999 0.217299999999898 -19.5720000000001
75.1193000000001 13.7883999999999 -20.4436000000001
98.1532999999989 6.29079999999985 -20.9531999999998
98.1540000000018 6.3812000000004 -20.9599000000002
114.8293 1.02749999999989 8.01310000000002
116.7295 23.3161000000002 -24.3195
119.114800000001 13.3859000000002 -12.9322
118.3426 9.98030000000022 -16.748
121.3364 -11.7746999999999 -16.4750000000001
133.105900000001 12.5720000000003 -18.5034000000001
143.7181 7.48620000000019 -18.9895000000001
153.1514 15.4858000000002 -21.3865000000002
157.396400000001 17.6733000000003 -27.8493000000002
153.549800000001 29.7298000000004 -29.9262000000002
166.819900000001 32.7216000000002 -16.1906000000001
165.8175 7.9262000000002 -24.5380000000002

162.928300000001 19.9126000000003 -33.1165000000002
28.7274000000004 4.99869999999992 -12.3546999999998
25.8051000000003 14.2494999999999 -14.2634
57.0429000000005 28.3771 -10.9985000000001
85.6924000000002 34.2651000000001 -19.5479
92.5684 30.7236 -21.0944
92.5875000000002 30.7100000000001 -21.1019
104.6129 42.6145000000001 6.55069999999998
116.0677 30.9164000000002 -13.2076
113.6229 33.9938000000002 -17.0686
108.7615 55.1326 -17.6366
127.377200000001 37.6840000000002 -18.6141
136.078100000001 45.9068000000002 -19.0427000000001
147.848100000001 41.4132000000003 -21.3584000000001
153.223300000001 43.1143000000003 -26.7502000000002
156.978900000001 53.5477000000002 -24.4179000000001
158.655200000001 41.3113000000003 -32.6578000000002
-0.125499999998146 0.184099999999867 -0.401300000000357
7.76570000000108 10.3988999999998 -1.32140000000018
59.1238000000006 24.0006 8.8775999999999
98.1767000000002 36.2200000000001 -17.222
90.5015000000003 43.7434000000001 -19.5717
59.5985000000008 11.4287000000001 12.5853999999999
121.8075 23.9803000000001 21.0567
155.173200000001 29.1645000000002 14.8608999999999
163.696000000001 30.7947000000002 11.7624999999998
151.111500000001 56.7826000000002 -2.37950000000014
159.608000000001 47.9061000000002 -19.6463000000001
0.0619000000019727 -0.810100000000154 -0.0382000000003685
10.4676000000017 -7.80610000000015 -0.777100000000191
63.814500000001 -0.766199999999881 9.14959999999983
104.2846 1.17140000000001 -16.3641
101.0067 -7.32870000000005 -19.3723000000001
162.672200000001 3.14410000000016 -2.24130000000017
166.910400000001 14.3683000000002 -20.3327000000002
ID=PRImaxUNKUNKMNHN885389

LM3=54
21.5424999999989 4.18650000000147 -17.6266000000008
23.4270999999989 -6.52609999999866 -19.6214000000007
59.2383999999996 -8.45939999999901 -13.5627000000005
94.9915999999999 -2.65139999999927 -20.8341000000001
87.2609999999997 15.7333000000008 -24.5768000000003
97.7781000000001 2.05350000000066 -20.4430000000002
97.821 2.00160000000052 -20.4177
117.012300000001 -1.43179999999932 7.5898999999999
121.2677 21.1711000000005 -27.3393
123.8602 10.3556000000005 -13.1165
125.169100000001 8.49530000000055 -17.2103
125.932300000001 -16.3679999999993 -17.7747
144.9318 13.3263000000004 -20.7002999999999
156.203000000001 7.68540000000052 -18.3964999999999
162.8591 15.6511000000004 -22.1511999999998
167.7557 16.3018000000005 -27.9940999999998

164.7363 29.4801000000005 -30.6896999999999
179.132 31.5849000000005 -18.6104999999999
174.584200000001 5.99320000000058 -24.1647999999999
170.7467 20.6192000000005 -34.9349999999999
21.5424999999989 4.18650000000147 -17.6266000000008
20.1692999999988 15.2934000000014 -18.7922000000008
53.9935999999992 29.024400000001 -13.6446000000006
88.5037999999989 36.2025000000006 -20.2681000000003
94.354999999999 32.0685000000005 -22.4426000000002
94.3377999999996 32.0763000000005 -22.4084000000002
108.606999999999 42.7144000000006 7.99469999999967
119.7392 33.3024000000004 -13.0951000000001
121.706499999999 35.0280000000004 -17.3195000000002
113.697399999999 59.9379000000006 -17.6890000000003
140.2573 36.8127000000004 -20.8932000000001
147.9228 47.9550000000004 -19.8112000000001
158.3556 42.3903000000004 -23.5373
163.1727 44.3711000000005 -28.1991
166.5406 54.7760000000005 -24.4941000000001
168.0783 40.8058000000006 -35.5183
-13.0659782795299 -1.6902787541141 -5.20669607846014
-5.93170000000111 9.0453000000017 -6.37700000000099
55.6640999999991 21.231600000001 8.49749999999947
97.905399999999 38.3757000000006 -16.2309000000003
95.1958999999991 46.5868000000007 -19.5169000000004
60.4152999999993 11.258500000001 11.6659999999995
129.092 22.2416000000006 20.7373999999998
162.8026 27.8640000000006 13.3829999999999
176.7025 30.5578000000006 9.05909999999993
164.2638 51.3751000000006 -14.6221000000002
171.0257 47.2406000000004 -19.6521
-12.6604397294773 -2.96957089100017 -5.31993559012869
-1.97650000000122 -10.6899999999983 -6.72550000000091
59.2510999999997 -0.368599999998973 7.47089999999956
105.3799 -2.86569999999943 -16.2312000000001
105.2045 -12.0397999999994 -19.7887
170.566300000001 1.90940000000065 -0.940799999999899
176.520600000001 12.8709000000005 -19.0129999999999
ID=PRImaxUNKUNKMNHN902637

LM3=54
30.8323090943714 5.62457639922157 -15.824431592575
36.6726999999907 -4.63470000000147 -17.7296999999979
72.1864999999931 -7.82320000000113 -14.8510999999983
107.367699999995 -1.7103000000009 -23.8326999999988
88.7789999999946 15.5622999999991 -24.7599999999989
113.688599999996 4.95249999999916 -23.6229999999991
113.715799999997 4.96689999999921 -23.5717999999993
133.650899999996 -1.14170000000099 5.12190000000092
134.795499999998 24.2660999999993 -30.6800999999995
140.158499999998 14.6010999999993 -17.8993999999995
139.325199999998 10.4523999999992 -20.3491999999993
139.805599999997 -18.751700000001 -22.671499999999
153.918899999998 10.8371999999993 -25.8221999999996

170.137399999998 6.09629999999918 -27.2299999999995
178.599299999998 15.8417999999992 -30.6286999999996
181.625699999998 16.3385999999992 -35.7524999999997
177.472099999998 31.2935999999993 -39.6771999999998
195.158899999997 34.225499999999 -28.5264999999996
190.028599999998 7.12769999999902 -34.6614999999995
185.043699999998 21.9973999999991 -45.9186999999996
30.8323090943714 5.62457639922157 -15.824431592575
31.3299999999906 16.9186999999986 -16.7032999999978
66.6496999999929 32.3404999999988 -14.6810999999985
102.793399999995 39.2988999999991 -24.1663999999991
110.929999999996 35.2015999999992 -25.1410999999993
110.956699999997 34.8007999999992 -24.8772999999994
122.665299999995 48.436299999999 3.16540000000076
137.850099999997 34.6391999999992 -17.4369999999996
133.304799999997 38.6017999999992 -21.7617999999995
125.077799999996 65.2864999999991 -22.8363999999995
148.696699999998 43.2925999999993 -26.6998999999997
161.854999999997 53.2197999999992 -27.4511999999997
174.161399999998 45.9923999999992 -30.2414999999997
176.619199999997 48.0530999999992 -37.2687999999997
180.928499999996 58.784599999999 -36.3842999999996
180.922199999997 43.9221999999992 -44.6094999999997
-0.406600000012271 0.428699999998066 0.227700000002804
8.56149999998831 12.2609999999982 -2.95479999999731
70.7266999999927 25.6614999999988 10.1198000000015
115.511799999996 40.3583999999992 -20.2115999999993
109.560499999995 50.9748999999991 -24.7267999999992
72.4149999999926 13.5355999999988 14.2943000000016
142.396599999996 24.9346999999989 19.5229000000007
184.701399999996 30.5592999999987 6.71120000000065
195.993399999996 30.4217999999988 2.60970000000063
182.875099999996 60.0459999999989 -11.8346999999996
186.637499999997 49.748399999999 -29.5433999999997
0.495599999987836 -1.87220000000191 -0.0574999999971654
12.8399999999884 -10.2794000000018 -2.72979999999727
75.5529999999929 -1.20210000000117 9.97830000000164
122.980299999997 -1.74570000000085 -19.571499999999
119.830499999996 -12.656300000001 -24.1032999999988
192.076399999997 -0.840200000001117 -11.8744999999993
193.015099999997 11.346599999999 -29.2652999999996
ID=PRImaxUNKGUYMNHN981682

LM3=54
27.5238107150758 5.45154874765322 -13.5729705618484
32.4638999999978 -3.67390000000056 -14.7377999999993
64.6040999999986 -6.26050000000038 -11.9647999999996
96.6461999999995 -0.983700000000192 -19.2933999999998
82.7923999999992 15.1373999999998 -19.6160999999995
103.8126 5.39819999999982 -18.4798999999998
103.8248 5.39989999999986 -18.5021999999998
120.3794 -1.82830000000027 7.77600000000021
126.2133 22.4047999999999 -25.9532999999997
127.615999999999 13.0526999999998 -13.3521999999997

126.0755 9.16399999999984 -17.0201999999998
126.9182 -16.7497000000003 -17.1662999999999
144.6701 11.5862999999998 -20.5916999999998
152.964399999999 5.84909999999965 -20.9774999999997
163.279599999999 15.2449999999997 -22.9073999999996
165.089999999999 17.1899999999997 -28.6486999999996
161.400499999999 29.7635999999996 -33.0074999999994
177.594499999998 32.5802999999995 -19.8582999999992
174.528899999999 8.15049999999949 -25.9662999999996
169.443199999999 20.9040999999995 -35.9528999999994
27.5238107150758 5.45154874765322 -13.5729705618484
28.5791999999982 16.0976999999995 -14.1771999999993
58.6296999999992 29.2765999999998 -11.0908999999993
89.7466999999996 35.8482999999998 -18.6860999999994
97.3652999999993 33.1499999999998 -19.6715999999994
97.3767999999997 33.1280999999999 -19.6728999999995
111.803099999999 45.0251999999997 7.57890000000078
124.1859 32.4445999999998 -13.0247999999995
121.2475 36.6427999999999 -15.1992999999994
113.870399999999 60.7577999999997 -16.5561999999991
138.272999999999 40.5466999999997 -20.5712999999993
144.750299999999 48.3089999999997 -20.9698999999992
156.995199999999 44.8007999999997 -22.6178999999993
159.774099999999 44.6862999999997 -28.3498999999992
164.938099999999 54.8247999999995 -25.078099999999
165.835699999999 41.1736999999996 -35.0481999999992
-0.039300000002457 0.176599999999282 -0.145499999999171
9.44469999999748 11.7955999999994 -2.08069999999912
67.0031999999987 25.4045999999997 9.77530000000071
102.549899999999 38.0239999999998 -15.9456999999993
98.4274999999994 46.9312999999998 -17.9099999999993
66.2769999999985 11.7132999999996 14.2685000000006
133.324899999999 23.6963999999996 21.4149000000006
169.314499999999 29.4235999999995 13.7001000000007
182.474899999999 32.7891999999995 4.42070000000076
163.944299999999 59.3143999999996 -6.96619999999901
170.076299999999 47.2328999999996 -21.4643999999991
0.304399999997539 -0.583300000000709 0.334200000000819
12.5338999999978 -8.01890000000062 -2.65409999999927
71.4299999999989 -0.912200000000338 9.54600000000046
109.636399999999 0.491399999999796 -16.2322999999998
107.903999999999 -9.50470000000031 -17.8222999999999
174.251799999999 0.768499999999511 -6.82909999999959
175.474999999999 11.8851999999996 -21.0163999999995
ID=PRImaxUNKUNKMNHNA3332*

LM3=54
32.1949000000038 6.44100000000031 -12.6396000000002
37.2801000000031 -2.86469999999978 -13.6317000000008
73.5044999999995 -4.09370000000033 -9.41410000000041
103.2854 0.444600000000152 -12.4413000000003
85.4214000000006 16.7043000000002 -14.8106000000003
109.509899999999 5.45469999999963 -12.1434000000003
126.004100000003 9.93410000000056 -6.71160000000047

122.843000000001 -0.73439999999961 20.8708999999996
136.622300000001 26.0959000000002 -15.7894000000005
134.981300000002 15.2956000000005 -1.3704000000004
137.026400000002 11.5969000000005 -3.78800000000047
135.502800000001 -15.5543999999996 -5.76340000000048
153.283600000001 14.8002000000003 -7.12150000000043
165.393400000001 8.71140000000029 -5.64950000000041
173.676800000001 18.9606000000003 -7.42580000000042
179.638900000002 19.0973000000003 -13.0400000000005
177.543500000001 33.8299000000002 -15.4458000000005
189.870500000002 35.7654000000002 -2.59820000000051
185.550700000001 8.43130000000039 -7.3568000000005
185.975200000002 24.5133000000003 -20.4931000000005
32.1949000000038 6.44100000000031 -12.6396000000002
34.3914000000037 16.7795000000001 -14.0924000000004
66.9093000000018 31.6947 -9.36780000000039
95.9130000000013 38.5011000000001 -11.9058000000004
106.062600000001 35.3568000000002 -11.9621000000004
121.775500000001 37.2287 -6.7710000000004
112.890000000002 47.3670000000001 19.2254999999996
131.333400000002 35.4440000000001 -1.57450000000044
131.416300000002 39.9260000000001 -4.65820000000043
120.364300000002 65.0378 -6.49080000000048
148.207900000002 43.4837000000001 -7.41430000000046
157.523700000002 53.1663000000002 -5.30220000000049
168.564500000002 46.5334000000001 -7.91220000000046
173.990200000002 48.0169000000002 -13.2213000000005
176.464900000003 60.4434000000002 -6.54280000000054
180.793400000002 45.9837000000002 -20.3944000000005
-0.261899999997299 0.469000000000116 -0.293000000000358
7.96600000000432 11.7870000000002 -1.95190000000043
66.4880000000015 27.1128 15.4961999999996
106.984700000002 42.2332000000001 -8.55590000000041
102.261700000002 52.6949 -10.4129000000004
68.1469000000012 13.4005 19.9989999999997
135.481000000002 26.4025000000003 35.2220999999996
174.518200000002 33.6949000000003 31.7736999999995
184.348600000002 35.0252000000004 28.6528999999995
170.987100000003 64.5580000000003 10.8076999999995
179.822800000002 53.5911000000002 -2.31990000000051
-0.364999999997429 -0.436399999999835 -0.186400000000363
9.35060000000348 -7.65889999999954 -2.11910000000037
71.0735000000007 0.0830999999999542 15.4361999999996
115.5934 1.31310000000013 -8.28330000000042
114.763700000001 -10.0295999999998 -10.3330000000004
182.122200000002 3.50980000000043 11.1449999999995
184.839600000001 14.5949000000004 -2.30740000000048
ID=PRImaxMALBOLUSNM261024

LM3=54
32.3377000000019 6.03899999999902 -12.1600000000001
35.4527000000022 -3.5286000000009 -13.1319000000001
68.7505000000013 -3.75060000000058 -7.93450000000001
101.2087 -0.30260000000044 -12.2853999999999

83.0277000000007 15.8963999999996 -15.6316
107.2004 5.49879999999968 -11.8532
119.7997 8.64399999999971 -8.79739999999997
120.9158 -0.843100000000323 19.4413000000001
131.5912 25.1431999999997 -17.7056000000001
133.5201 16.0264999999998 -2.99110000000001
134.4386 10.4020999999998 -5.6701
133.773799999999 -14.0422000000004 -8.94979999999998
151.8416 15.8197999999997 -7.96230000000004
160.4844 7.77569999999973 -8.87999999999995
168.520600000001 18.3127999999997 -9.09999999999994
172.885200000001 19.9150999999997 -15.2906
171.185300000001 32.5147999999997 -16.9370000000001
186.292100000002 35.4339999999997 -5.23130000000007
181.400100000001 9.45419999999968 -9.8295999999999
180.570500000001 24.2331999999996 -22.3293
32.3377000000019 6.03899999999902 -12.1600000000001
31.3357000000026 15.6019999999992 -12.8968000000001
62.2296000000017 28.6730999999994 -8.38790000000003
95.1033000000014 37.5157999999997 -11.5647000000002
102.750400000001 34.9977999999996 -12.2096000000001
114.311200000001 35.3411999999997 -8.47760000000012
112.491600000001 44.8971999999998 18.1463999999998
130.2641 34.3831999999998 -3.26500000000007
130.075100000001 38.9261999999998 -6.07830000000012
119.778000000001 62.2654999999996 -7.21970000000031
147.122300000001 41.1731999999998 -8.1780000000001
152.932600000002 50.7763999999998 -7.20570000000015
164.623600000002 45.3437999999998 -9.9809000000001
168.104200000002 45.7014999999997 -15.9183000000001
172.133400000003 58.6044999999998 -9.66420000000016
176.433600000002 43.9755999999997 -21.8118000000001
-1.22059999999704 0.563999999998836 -0.282400000000121
7.45590000000297 11.831099999999 -2.16740000000006
67.3561000000019 26.7695999999995 14.7990999999999
114.189100000001 38.6683999999997 -6.74080000000019
101.932000000001 51.2665999999996 -11.3868000000002
73.9730000000017 14.4743999999995 21.7394
134.203500000001 26.2887999999998 32.3385
173.186100000001 34.2056999999998 28.3547999999999
182.498900000001 35.9047999999997 23.9027999999999
168.395300000003 63.2758999999998 7.83289999999976
176.419100000002 51.8977999999998 -6.32120000000013
-0.820799999996958 -0.569200000001142 -0.234700000000125
10.6455000000025 -8.12230000000113 -1.98180000000016
71.6942000000013 0.0610999999994393 15.0429
111.6329 1.2529999999997 -9.59319999999996
112.7707 -9.27860000000043 -10.2738999999999
172.886000000001 1.45199999999974 6.53830000000013
181.126200000001 15.6199999999997 -5.40179999999991
ID=PRImaxMALBRAUSNM270373

LM3=54
30.1286 6.9296 -10.8813

35.1652 -2.9638 -11.7906
71.1099 -2.9903 -6.9119
102.3352 2.1831 -12.6264
85.7853 17.9678 -14.4883
108.4873 8.5603 -12.7602
120.8457 11.5255 -9.589
119.8003 0.9419 17.7211
132.7282 27.3175 -17.4003
131.4523 17.8819 -2.7177
132.4306 11.9162 -4.3589
133.8842 -11.1549 -6.9079
147.0589 14.7338 -7.912
160.0277 9.2486 -6.6145
168.1767 18.7601 -8.5861
173.0861 19.6867 -13.9588
170.176 33.2922 -16.2672
184.3689 35.8964 -3.1819
180.9948 10.0391 -8.9684
179.8551 24.8361 -20.6783
30.1286 6.9296 -10.8813
30.5071 18.617 -11.4639
62.8891 33.1231 -6.6926
97.6692 39.4972 -12.1383
103.5394 36.6508 -12.5573
116.2387 38.214 -9.6264
111.1625 46.4984 16.2028
128.4 35.7369 -2.686
127.0042 41.1061 -4.9277
119.1197 62.6452 -6.9923
144.1843 42.8165 -7.6949
150.6542 52.6645 -7.0265
163.1516 46.6568 -9.2916
167.7826 47.9512 -14.0511
170.5076 59.5094 -10.4758
173.6 44.1538 -20.8696
-0.7834 1.4928 -0.153
6.9899 11.3545 -0.3797
68.3294 28.7978 15.6819
109.3525 42.0137 -9.001
100.3813 52.278 -12.1402
71.3903 16.153 18.8968
133.3919 26.9217 31.6028
166.7108 33.5905 29.1422
176.2891 35.9026 27.0418
166.6062 61.5795 11.1431
172.1802 54.1501 -4.0731
-0.1709 -0.3284 -0.059
9.784 -6.6592 -0.7755
72.5584 2.8412 15.6013
115.5942 4.4723 -8.3593
110.4176 -7.9941 -10.9495
178.0289 6.9484 10.8984
178.9327 15.4073 -3.3723
ID=PRImaxMALZOOUSNM299630

LM3=54
33.16 6.5639 -11.2821
38.5381 -2.6617 -11.5136
70.0089 -3.2113 -6.9512
103.261 2.0245 -12.5062
85.2 17.5753 -14.2275
109.6546 7.4073 -13.298
121.9209 10.1715 -8.4568
120.2341 -0.7625 16.2284
130.5747 26.1181 -16.0435
131.2788 16.0344 -3.5592
132.1352 10.4375 -5.165
133.4715 -13.8412 -8.7627
147.3807 14.7447 -9.2349
160.9416 8.9972 -8.1549
167.9554 18.8656 -9.7713
173.7596 19.7664 -15.5331
170.1237 34.0669 -17.6898
184.821 37.0709 -3.6211
181.667 9.4045 -11.1671
180.7057 24.6225 -21.0631
33.16 6.5639 -11.2821
36.7323 17.4713 -11.7491
62.2189 30.7821 -7.7129
95.767 38.4078 -11.9324
102.4066 36.1116 -12.1835
116.1684 38.1942 -7.6628
110.4193 47.63 17.1497
127.6648 36.4892 -2.7849
125.0811 41.8814 -5.1204
117.9865 65.2361 -8.3
141.6084 44.988 -9.266
151.8241 53.5144 -8.0528
162.5339 47.2763 -9.8676
166.8879 48.4379 -16.0279
171.2547 60.7954 -11.1202
173.8163 45.6078 -21.8474
-0.2165 0.523 -0.0918
9.0483 11.8294 -0.733
66.9886 26.0935 15.565
100.5348 51.9614 -10.8629
107.1607 43.1374 -7.3934
68.5123 13.6247 19.2988
136.4278 26.1123 33.9447
169.9096 32.2023 29.2336
182.2351 36.1112 24.3874
166.4898 63.3489 8.1246
173.6822 54.9136 -6.4531
-0.0196 -0.8054 -0.1403
12.8978 -8.105 -1.2346
72.2487 1.5591 15.6987
112.9785 0.4782 -8.517
111.4619 -9.1886 -10.7333

178.1991 4.2626 7.7751
181.2121 14.9585 -4.914
ID=PRImaxFEMVENUSNM406694

LM3=54
32.1625999999998 6.14479999999984 -9.90049999999986
35.2226000000103 -4.33879999999773 -10.8164999999999
70.1405999999987 -5.20550000000006 -3.69990000000027
100.917899999996 0.583799999998857 -11.5785
85.0899999999988 16.2701999999997 -13.6706000000001
109.4404 7.64340000000025 -12.8015000000001
124.075899999999 10.6037999999998 -6.84420000000014
118.527 -1.98680000000008 17.4593999999999
131.284299999998 25.5210999999996 -15.1731000000002
131.687699999998 14.9813999999995 -2.69810000000013
132.3253 10.2989999999999 -5.30370000000016
135.993600000001 -14.3859999999999 -7.02010000000017
146.608899999998 14.8560999999996 -8.49910000000017
162.1806 9.00799999999984 -7.87820000000024
171.076699999999 19.6604999999997 -9.23670000000023
176.201399999999 19.4226999999998 -15.3285000000002
172.1968 34.6634 -18.2498000000003
186.1901 37.6674 -4.37220000000027
182.772 10.7427 -11.3456000000002
183.298166553633 25.9133352267904 -21.135748292252
32.1625999999998 6.14479999999984 -9.90049999999986
31.0424999999997 17.0817999999999 -11.2750999999999
60.264499999999 29.6751999999999 -4.19280000000007
92.3041999999986 37.4918999999997 -10.8887000000001
103.228199999999 35.5684999999998 -12.2909000000001
118.224399999998 37.2844999999997 -7.56880000000016
109.921599999998 46.5862999999996 17.5007999999999
128.784099999999 35.2909999999996 -2.73910000000018
126.519099999998 40.7088999999996 -5.66520000000015
119.632999999999 65.8852999999997 -6.48260000000017
141.327799999999 43.8238999999997 -8.25800000000021
152.612599999999 54.1499999999998 -7.18050000000022
164.634899999999 48.8462999999998 -9.14250000000023
168.859499999999 49.6392 -14.7355000000002
173.120499999999 61.3744 -9.94140000000025
177.537477444165 47.1649683428392 -20.0622791166441
-0.395499999996668 1.51290000000065 0.125200000000094
7.19500000000159 11.9229000000004 -1.8212999999999
66.684599999999 27.0680999999998 16.3372999999999
106.299599999999 42.0800999999997 -7.89550000000012
100.545599999999 51.4249999999998 -10.1752000000001
68.5766999999993 13.5626999999999 21.3442999999999
134.3679 26.6031999999998 32.7955999999999
172.6095 34.4515999999998 28.2326999999997
183.2508 37.3880999999999 21.2130999999997
166.361499999999 63.4374999999998 11.2129999999998
175.029999999999 55.4348999999999 -4.18990000000023
-0.432199999996498 -0.353699999999328 -0.155799999999894
10.8856000000033 -7.84659999999947 -1.95639999999986

71.3909999999995 0.671599999999949 17.4139999999999
114.665899999999 0.966199999999884 -7.32560000000008
113.428999999999 -9.26470000000028 -9.66590000000008
180.256100000001 5.27620000000002 9.40829999999973
183.0844 16.9737 -4.81950000000026
ID=PRImaxUNKVENUSNM406695

LM3=54
15.2339999999995 2.65329999999983 -2.6000000000009
13.6170999999992 -6.85800000000029 -2.87640000000056
47.8710000000004 -1.80159999999986 -11.8651000000006
58.0728000000005 -1.08879999999983 -18.4594000000006
44.9385000000004 15.9623000000003 -13.8513000000006
61.3704000000004 4.88180000000019 -20.2573000000005
60.5120000000006 6.81780000000028 -18.1395000000006
71.3768000000002 4.73540000000016 7.61409999999947
69.2919 24.9428000000001 -25.9045000000004
78.9841000000004 22.2940000000002 -21.2496000000004
85.8717 20.1338 -27.8020000000003
83.8796000000002 -11.4098 -16.8142000000005
92.3619999999998 19.4010999999999 -29.8285000000003
108.977399999999 15.5051999999998 -32.9030000000002
114.080099999998 30.3903999999995 -41.0873
116.088699999998 32.2793999999994 -41.6627
116.760599999998 39.8811999999994 -44.377
124.278999999998 42.9567999999994 -34.937
123.537499999998 29.1750999999994 -35.7852
125.350799999998 36.4186999999994 -46.1291000000001
12.8353999999991 9.23559999999977 -2.57170000000058
6.24909999999877 16.3061999999997 -2.86370000000054
35.6838999999998 33.0688000000001 -11.7599000000006
45.0079999999998 38.2530000000002 -19.3178000000005
51.3873 35.4854000000002 -20.7729000000005
51.6160000000001 33.7388000000003 -18.0333000000005
57.8962999999996 39.3166000000001 8.17069999999952
75.8253999999998 31.8662000000001 -21.0766000000003
80.0675999999993 37.6592999999999 -28.5833000000002
59.891099999999 61.8453999999999 -16.7786000000003
84.695399999999 43.2620999999998 -30.6258000000001
95.3185999999982 55.4652999999995 -33.162
108.653999999998 46.1913999999995 -42.54
110.191299999998 46.8296999999995 -43.1805
114.508899999998 53.1551999999994 -35.2115999999999
120.141699999998 48.2704999999994 -46.5487000000001
14.2622999999992 6.24529999999986 10.4879999999994
9.16479999999897 9.63529999999979 9.30689999999942
38.9462999999998 17.8806000000001 11.8481999999994
52.2123000000002 31.1761000000003 -10.3558000000006
45.8202999999994 48.295 -22.8471000000004
53.8726000000001 18.9574000000002 13.5915999999994
93.7682999999994 32.9744999999999 5.39539999999967
124.505199999998 43.3136999999995 -10.8163000000001
113.407399999998 57.5481999999995 -25.4256
131.055099999998 45.5532999999994 -17.1462000000001

116.465599999998 50.5473999999994 -34.4543
14.3989999999993 5.27609999999988 9.99119999999941
12.7322999999992 -1.22940000000019 9.12459999999939
40.0689999999999 11.0558000000001 11.4435999999994
59.8420000000006 9.00230000000025 -10.1255000000006
65.7025000000006 -8.42709999999982 -22.9998000000006
124.572299999999 25.5496999999995 -24.8743000000001
122.906799999998 32.7276999999994 -34.0888
ID=PROcriMALKENUSNM164503

LM3=54
12.4586000000003 1.30320000000021 -4.48330000000016
12.4124000000002 -7.58790000000014 -4.98670000000055
44.8276999999996 -3.90340000000023 -13.9250999999999
54.4133000000002 -1.22369999999969 -18.3662000000002
40.8915000000001 15.3345 -16.5584
58.5131999999994 4.90119999999991 -23.5493999999998
56.2948 6.66280000000004 -19.5765
65.6083999999993 4.9184999999999 7.20860000000019
66.8419000000002 25.2246000000003 -28.6566999999999
74.3487000000009 23.7919000000005 -21.6763000000002
81.9832000000009 21.9416000000005 -29.1810000000001
80.382883780523 -11.4841482169436 -16.841246064829
89.5198000000014 20.1488000000007 -30.7397000000003
106.466500000001 16.5513000000005 -32.7571000000001
107.218500000001 33.1646000000003 -42.0160999999999
110.332700000001 33.1454000000003 -43.4122999999999
111.536400000001 42.6711000000004 -45.421
118.1766 45.3322000000002 -35.7034999999998
116.391100000001 31.6989000000003 -36.2494999999998
120.624200000001 37.8784000000004 -46.9433999999999
9.84809999999937 7.4777999999999 -4.01240000000012
4.68349999999901 15.0288999999997 -4.95020000000001
32.2700999999996 32.8801999999999 -13.4200999999999
40.2902999999999 38.1264000000001 -18.1774999999999
46.3318999999999 33.3738000000001 -21.4193999999999
46.3764999999999 32.1176 -18.6242999999999
53.3275999999998 42.0623 7.56460000000012
69.7560000000004 32.9560000000003 -20.9868
75.4543000000004 37.9310000000003 -28.7751999999999
53.1490999999998 62.3641 -14.6970999999998
79.9847000000003 45.2192000000002 -30.2556999999999
90.1920000000005 58.6404000000003 -32.7443999999999
101.561400000001 46.9732000000002 -40.8882999999999
104.411500000001 48.2760000000002 -42.1571999999999
108.0392 54.1268000000002 -36.1421999999998
114.823200000001 51.6653000000003 -46.5787999999999
15.9523999999995 6.0457999999999 8.98599999999981
8.92509999999929 10.0240999999998 6.65629999999989
35.6352999999995 16.7227999999999 8.05330000000002
47.9278999999999 29.6573 -11.5150999999999
42.3222999999998 50.6452 -22.7991999999999
51.6052999999996 18.0596999999999 11.2945000000001
87.3265999999999 31.4735000000001 6.52440000000018

117.9946 43.0703000000002 -9.66939999999974
109.7173 57.0984000000002 -25.6136999999998
125.9786 46.4260000000003 -19.2009999999998
111.3101 50.6480000000002 -34.2013999999998
16.2908999999996 4.6014999999999 8.99549999999979
13.6328999999998 -2.84630000000006 6.43989999999972
38.5143999999996 9.37569999999989 7.99860000000001
55.9573 7.54070000000004 -11.8789
63.6081000000002 -11.8417999999998 -24.0651000000001
121.139200000001 28.0364000000003 -25.4022999999999
117.305900000001 34.2370000000003 -34.5122999999999
ID=PROcriFEMKENUSNM164837

LM3=54
12.2409 1.1483 -2.408
11.7803 -7.4398 -2.1177
41.6051 -4.4518 -11.3544
49.87 -5.7859 -18.0295
44.3648 13.5435 -17.0969
52.2918 2.0376 -19.8766
51.7994 3.3917 -17.5901
64.1955 3.2282 5.6651
59.9783 18.7636 -26.8369
70.4659 16.1192 -21.3155
75.5701 14.7271 -29.2318
73.9909 -12.72 -18.245
81.822 12.3779 -31.3455
96.8257 7.9782 -34.9063
99.2818 22.8308 -43.0075
102.1838 23.833 -43.4157
103.0364 31.8235 -46.8662
109.8363 33.689 -38.5624
108.3046 19.7541 -39.1896
110.41 26.3819 -50.4444
10.886 6.056 -2.311
5.3543 12.7761 -2.1684
31.3757 26.7378 -11.4647
37.6436 31.7841 -18.162
44.3756 27.6541 -19.5007
44.726 26.5101 -17.3303
53.3866 31.2043 6.1104
66.8636 25.8533 -21.2487
70.3675 30.1691 -28.9346
53.4316 51.3007 -17.4759
74.9112 34.3709 -30.8933
84.4075 48.5918 -34.3655
94.6022 36.9447 -42.4819
97.5019 37.5278 -43.0012
100.4416 44.5568 -38.4891
104.8916 40.6705 -50.4536
15.6116 4.6799 9.6073
10.7642 8.4495 8.4708
37.2067 14.2908 9.1671
44.4691 25.069 -10.4545

41.1781 42.1854 -23.2044
48.31 13.9934 10.2295
84.0289 25.8562 3.6452
111.8077 34.3618 -13.6918
103.5528 46.5363 -26.6465
118.6038 35.6765 -21.5017
104.118 42.4501 -37.9405
15.6339 3.7483 9.6896
14.0388 -2.412 8.1028
39.6351 8.4152 9.3598
51.1483 4.3619 -10.9679
57.045 -11.7217 -23.8617
111.6307 19.3234 -27.5559
109.8174 23.4105 -37.882
ID=PROcriMALKENUSNM181495

LM3=54
13.329400000005 2.73160000000203 -1.70250000000198
13.9354000000052 -7.44289999999792 -4.06770000000203
43.7701000000035 -1.96719999999861 -9.72530000000141
53.1641000000029 -1.24149999999884 -14.4651000000012
41.868300000003 14.5863000000012 -11.6424000000012
56.3898000000025 5.026500000001 -17.275600000001
54.0145000000026 7.71970000000106 -14.4445000000011
71.8109000000036 2.9696000000013 7.65699999999863
59.684200000002 21.3416000000008 -20.7621000000008
74.421100000002 21.5769000000007 -18.4187000000007
80.6085000000018 19.0417000000006 -27.0400000000006
76.9429000000031 -10.4493999999989 -11.8408000000012
86.1180000000018 17.0993000000006 -29.1627000000006
101.276307098934 13.8067264239593 -33.805030976351
103.960900000002 28.6866000000005 -43.6179000000004
106.724200000002 29.5978000000005 -44.4934000000004
106.146200000002 36.8705000000005 -49.2549000000004
116.060200000002 40.4803000000005 -39.4271000000006
113.130400000002 27.4949000000006 -42.3668000000005
116.566800000002 32.9435000000006 -50.7279000000005
11.9606000000048 7.31990000000194 -1.57260000000189
5.63700000000484 14.787300000002 -3.83190000000184
33.6407000000033 29.4119000000014 -10.6885000000012
41.4401000000028 34.2125000000012 -15.805500000001
46.9573000000025 29.8439000000011 -15.8438000000009
46.9076000000027 28.6030000000011 -14.638200000001
57.6110000000034 40.8139000000013 8.19059999999885
70.6812000000019 29.6274000000007 -18.8484000000007
74.9946000000017 35.7635000000007 -27.4374000000005
53.1965000000028 55.4733000000012 -12.9710000000009
78.3721000000014 41.1182000000005 -29.1319000000004
87.6245000000018 52.1829000000006 -33.9358000000005
98.4705000000017 43.4605000000005 -44.7869000000004
101.211000000002 44.0367000000005 -45.0015000000003
104.732700000002 48.3589000000006 -42.2077000000005
109.319700000002 44.1744000000006 -52.4145000000005
13.5943000000051 5.21610000000203 10.862599999998

7.30610000000541 9.53600000000214 8.83399999999796
36.543400000004 15.6469000000015 11.6723999999985
48.2060000000027 29.0320000000011 -7.58640000000103
43.2949000000027 45.2094000000011 -21.1821000000009
51.7157000000038 17.9264000000014 14.0887999999986
89.868800000003 32.115700000001 2.23129999999904
117.199100000003 42.6058000000009 -15.0092000000008
107.809600000002 54.8117000000007 -30.0890000000006
122.712000000003 44.6997000000009 -24.6552000000008
106.589200000002 47.9126000000006 -40.1604000000005
14.6380000000051 3.95490000000205 11.514699999998
12.0039000000056 -3.4739999999978 8.63189999999784
39.6521000000041 9.40470000000155 12.1944999999984
55.5078000000028 7.82070000000111 -7.78010000000115
62.1788000000031 -8.01229999999882 -22.0041000000012
117.852000000003 27.2038000000007 -30.0042000000007
112.869400000002 31.2641000000006 -40.1467000000005
ID=PROcriUNKSAFAMNH344893

LM3=54
15.0445000000008 1.8752000000014 -2.29560000000033
12.5655000000005 -8.00359999999867 -3.40350000000037
43.7631 -2.44709999999915 -11.2171000000003
51.4257000000002 -0.517399999999123 -15.5397000000003
39.5606999999994 14.4749000000005 -12.3986
57.9538999999998 6.66660000000062 -19.0763000000002
56.1100999999998 8.39440000000063 -16.2975000000002
67.6696000000002 6.42880000000092 9.56519999999974
59.8462999999995 21.7203000000004 -23.4802
77.1938000000001 23.5467000000006 -19.3676000000002
82.2068000000003 21.2169000000007 -26.4962000000003
80.5163000000005 -10.0985999999989 -12.8607000000005
89.0346000000007 20.0635000000009 -28.1414000000004
106.574400000001 16.2788000000012 -30.4623000000006
109.008700000001 33.0719000000012 -39.4683000000005
112.802500000002 33.1904000000013 -41.2620000000005
114.975800000002 42.9143000000013 -45.2034000000005
119.978900000002 44.6967000000014 -35.2236000000005
119.484100000002 30.9233000000014 -34.4427000000006
123.996700000002 38.6326000000015 -45.8448000000006
12.8497000000005 8.09460000000123 -2.41750000000026
4.2416000000037 14.6229000000012 -2.60380000000017
31.5614999999998 30.0584000000006 -10.9524999999999
41.8048999999998 36.0145000000005 -16.9312999999998
47.3861999999997 31.9832000000004 -18.4682999999998
47.7122999999998 29.9126000000005 -16.4772999999999
55.2937000000005 37.4730000000009 10.2492
73.2633 32.1450000000005 -19.3161000000001
77.8895999999999 37.5037000000005 -26.0874
54.2659 59.5383000000006 -12.2510999999998
80.1627000000001 43.6936000000005 -27.7529
91.1589000000008 56.8562000000008 -30.3099000000001
103.568100000001 47.0764000000011 -40.3206000000003
106.554500000001 48.7556000000011 -41.0371000000004

109.666700000001 55.1992000000011 -33.7861000000003
117.404000000002 52.2462000000013 -46.6595000000004
14.9420000000007 6.04980000000139 11.1458999999997
8.50650000000071 10.5602000000014 9.00149999999969
40.1831000000004 17.254900000001 11.9988999999999
47.8075999999998 29.9296000000005 -9.06509999999989
41.7178999999999 46.2275000000006 -20.6504999999998
53.2048000000005 18.980400000001 13.5734999999998
91.1495000000005 33.1211000000009 5.05329999999982
121.715100000001 44.1384000000012 -9.44990000000034
107.218500000001 57.6686000000011 -25.9960000000002
126.866500000002 45.9472000000014 -15.5941000000004
113.073000000002 51.8859000000012 -32.7173000000003
15.3305000000007 4.69920000000139 11.2454999999997
13.8540000000008 -2.98879999999853 8.67439999999957
42.0938000000003 11.435700000001 12.0327999999998
55.1305999999999 8.02260000000065 -9.39170000000018
63.4266000000002 -8.44949999999904 -20.6550000000004
118.428200000002 25.6235000000014 -26.8394000000006
119.742000000002 34.0252000000014 -32.2572000000006
ID=PROcriFEMBOTUSNM368496

LM3=54
12.9923000000025 1.77420000000098 -3.24230000000102
14.1305000000027 -7.30499999999893 -4.07890000000122
47.2615000000018 -3.36459999999934 -12.7456000000009
57.2616000000013 -2.14979999999949 -17.1171000000008
41.5732000000016 12.8054000000006 -13.7718000000007
59.2604000000012 4.28300000000047 -19.2939000000007
58.2896000000015 5.34190000000053 -17.3919000000008
72.4492000000008 6.22990000000035 7.71389999999932
62.5570000000007 19.7470000000003 -24.7336000000005
78.1867000000002 21.2143000000002 -21.3495000000004
85.4741999999996 17.2003000000001 -28.1793000000003
83.7652000000001 -13.4586999999997 -17.0103000000006
92.2207999999992 15.0190000000001 -30.1558000000002
107.782299999999 11.8911000000002 -33.8009000000002
112.735899999998 27.3577 -45.4076999999999
115.228499999998 28.3959 -45.0334999999999
115.082999999998 36.0228000000001 -50.2110999999999
122.107999999998 38.7871000000002 -41.3866999999999
119.841999999998 25.4523000000002 -39.1165000000001
123.977699999998 30.5942000000001 -51.7362999999999
11.7794000000029 5.94640000000108 -3.79290000000111
7.27550000000301 13.7310000000012 -5.71180000000114
37.9083000000024 29.3344000000008 -14.5938000000008
45.0486000000018 34.2800000000006 -18.9542000000006
50.1861000000014 31.1554000000005 -20.8304000000005
50.4222000000014 28.2540000000005 -17.9141000000005
61.3553000000014 38.7703000000005 6.37159999999945
75.3955000000004 28.8929000000002 -21.6946000000003
79.4229999999998 36.1299000000001 -28.8835000000001
59.182800000001 59.3327000000005 -20.3821000000004
83.7047999999997 41.3645000000001 -31.5018000000001

94.0068999999992 52.1517000000001 -35.5621
107.847799999998 42.0493000000001 -46.0484999999998
110.509499999998 42.8259 -45.8670999999998
111.657499999998 48.3383000000001 -40.5680999999998
117.766999999998 46.0108000000002 -53.5251999999999
17.4835000000029 6.16100000000102 9.89119999999892
9.97370000000292 10.2811000000011 7.7511999999989
39.2449000000021 16.1079000000007 9.83549999999915
51.8331000000015 27.6721000000005 -9.28050000000053
45.7208000000013 43.5315000000006 -23.1405000000004
55.0200000000017 18.6788000000006 10.8100999999992
95.9719000000002 32.5109000000003 2.18749999999957
124.838299999999 40.4206000000003 -13.7784000000002
115.539399999999 54.1590000000002 -29.9957999999999
129.218299999999 41.8957000000003 -20.6681000000002
116.477999999998 45.9900000000001 -37.8571999999999
18.5106000000031 5.19360000000108 9.74589999999879
13.9175000000029 -2.10919999999893 8.03269999999882
40.919700000002 10.8719000000007 9.75959999999913
58.0680000000014 8.83590000000047 -8.90780000000074
61.8247000000007 -9.30239999999956 -21.0603000000008
125.426399999998 24.7601000000002 -28.2282000000002
121.209899999998 30.9080000000002 -37.1016
ID=PROcriFEMBOTUSNM368497

LM3=54
13.3145999999997 2.41660000000036 -4.08140000000012
13.1361 -8.36099999999975 -3.81870000000018
44.2837999999996 -3.09349999999992 -11.1906000000001
54.0698999999996 -0.957499999999982 -17.9384
40.2425999999997 14.6985000000002 -14.518
57.2072999999997 5.63600000000002 -20.9890000000001
56.2867999999997 8.13630000000007 -19.1137000000001
68.3873 5.92519999999998 8.67939999999985
62.3099999999998 22.6832 -26.2596
74.8793999999998 21.5303999999999 -20.6528
81.7292000000001 19.2507999999999 -27.3682000000001
81.9535000000007 -9.97279999999991 -13.1071000000003
87.6876000000005 18.3130999999999 -30.0954000000001
103.590700000002 15.9175000000001 -32.2599000000004
109.861900000002 32.0927000000002 -42.9602000000004
111.955500000002 33.0995000000002 -42.6438000000003
113.566900000002 40.8976000000001 -45.6612000000003
118.094900000002 42.5325000000001 -37.5093000000004
115.831100000002 29.1407000000002 -35.3956000000004
120.685700000002 35.9989000000002 -47.1310000000004
11.6667999999998 6.97890000000019 -3.75620000000003
5.10739999999985 15.0053000000002 -4.13280000000004
32.0837999999998 31.9691000000003 -11.3739
41.5586999999999 36.0360000000003 -17.474
48.3117999999999 32.0059000000002 -20.5032
49.1229999999998 30.6047000000002 -18.8439
56.2118000000001 37.8892000000001 8.5497
71.3817999999999 31.0994999999999 -20.2329999999999

74.932 37.3686999999998 -26.5437999999999
56.9903 60.6637999999999 -11.9102999999999
79.0866000000004 42.7147999999999 -29.5253999999999
91.3005000000007 55.5068999999997 -32.5192999999999
105.131200000001 45.6934999999999 -42.4279000000002
107.134500000001 45.9976999999999 -42.4801000000001
107.874400000002 52.1670999999999 -35.2107000000001
115.194453985575 48.8921542057973 -47.4969151971504
16.4974999999996 6.84260000000011 9.58660000000001
9.03329999999978 9.7634000000002 7.83689999999998
37.5864999999998 18.4606000000002 10.1283999999999
48.1915999999997 30.2463000000002 -10.6414
41.1762999999999 45.9867000000001 -20.3388999999999
51.8453 18.0817000000002 12.2794999999999
86.4870000000004 30.6314999999999 4.32629999999991
121.970000000002 43.9294000000001 -11.5493000000003
113.518700000002 55.8000999999999 -24.3714000000001
126.973000000002 45.6834000000001 -18.7397000000004
112.148300000002 49.6448999999999 -34.5919000000002
16.5191999999997 5.73100000000015 9.00579999999997
12.7199999999999 -2.22719999999981 7.38419999999991
40.5420999999999 10.2247000000002 10.5037999999999
55.4209999999997 7.37640000000006 -11.3163000000001
60.0186999999999 -8.86739999999999 -22.0015000000001
121.947200000002 28.4106000000002 -24.9160000000004
117.587700000002 33.0796000000002 -35.3794000000004
ID=PROcriFEMBOTUSNM368499

LM3=54
14.0359000000015 1.58580000000009 -2.58280000000136
15.257800000001 -7.09780000000012 -4.05570000000122
47.9306 -1.74080000000064 -11.9043000000005
59.5789000000012 -0.816200000000051 -17.3726000000008
47.1065000000003 15.5452999999996 -14.9257000000007
61.9428000000012 5.69489999999999 -19.7014000000008
60.4240000000003 8.10299999999978 -17.4246000000005
74.367 6.79049999999962 9.30219999999962
68.6533000000003 22.5015999999998 -25.2748000000004
81.1576000000009 23.0846000000001 -20.3440000000005
89.2315000000011 21.0834000000002 -28.2218000000004
87.2675000000002 -8.80560000000026 -13.0958000000003
96.1057999999999 17.8354999999999 -29.5323000000001
112.5445 15.3326999999999 -30.5384999999998
118.3076 30.9147000000001 -39.7915999999998
120.449 31.5625 -41.3885999999996
120.0394 38.5735 -45.8137999999997
129.8847 42.1262000000001 -36.5155999999997
125.4557 27.7004000000001 -35.1239999999997
129.5528 34.6011 -49.5254999999996
12.3242000000007 7.57409999999969 -2.71180000000103
7.00620000000035 14.4201999999995 -3.77370000000096
37.7572000000002 30.6622999999995 -12.3166000000006
47.5240000000002 35.2939999999996 -17.6553000000005
53.7759000000002 31.5908999999997 -19.3999000000005

53.8586000000002 30.2483999999997 -17.7287000000005
63.157 38.2991999999997 9.98869999999958
78.5338000000003 30.6986999999999 -20.3908000000003
83.33 37.3442999999999 -28.2309000000001
64.5649000000001 59.6359999999998 -13.5755000000004
87.9911000000001 42.405 -29.7908000000001
99.7699999999999 54.1430999999999 -32.1933999999999
113.7019 44.2204 -41.3759999999997
116.2285 45.6489 -42.0664999999997
117.8443 51.309 -36.1258999999997
124.991 47.2079 -49.5018999999997
16.5036000000006 5.79339999999967 10.718299999999
9.85910000000059 10.1510999999996 8.43739999999896
41.8900000000002 16.5906999999996 11.1157999999993
54.6836000000001 29.5985999999996 -10.7376000000005
48.9955000000002 45.7438999999997 -21.0097000000005
55.4975 18.3375999999996 13.5368999999995
99.3568999999999 32.5513999999998 5.71639999999987
130.7576 43.0691 -8.60419999999983
122.262 56.7933 -25.2082999999998
136.7417 44.904 -15.2510999999998
122.0398 49.2725000000001 -36.0440999999997
16.8870000000006 4.50269999999966 10.959099999999
14.8846000000008 -3.43090000000029 8.42559999999891
44.2124000000002 10.8229999999995 11.2587999999993
61.2111000000002 8.07429999999967 -11.0730000000005
66.1758000000006 -7.66920000000018 -20.9058000000005
131.2018 26.7048 -25.5398999999997
126.9704 32.7169000000001 -34.6202999999997
ID=PROcriFEMBOTUSNM368501

LM3=54
12.972800000004 1.40870000000086 -3.10480000000138
11.9152000000037 -7.71429999999913 -2.87990000000142
43.7996000000023 -4.99629999999923 -10.308800000001
54.936100000002 -3.80349999999948 -17.3376000000008
41.8563000000026 12.7251000000006 -12.2796000000009
58.1465000000025 3.78490000000044 -19.5806000000009
56.3140000000019 5.46160000000036 -17.3805000000007
71.4828000000023 2.61910000000064 8.04719999999914
63.9150000000006 18.7719999999999 -24.5923000000002
75.8757000000024 19.5937000000005 -21.1725000000008
82.7919000000013 15.8559 -28.7544000000004
81.2635000000025 -10.5597999999994 -16.065900000001
89.1990000000024 13.9946000000004 -31.0484000000009
103.690000000001 10.7157000000001 -34.0757000000006
107.918800000001 25.4926999999998 -44.2055000000005
110.643800000001 25.0435999999999 -45.8294000000005
112.332900000001 33.8499999999998 -50.5074000000005
120.185600000001 36.7979999999999 -41.5585000000006
116.989800000001 24.2945999999998 -40.2011000000005
119.979900000001 29.4455999999999 -52.7882000000006
11.9793000000042 5.55680000000101 -3.17970000000137
5.75210000000395 13.011100000001 -3.22640000000133

33.5346000000031 27.7451000000007 -10.773900000001
43.9065000000026 32.4453000000006 -17.4625000000008
50.2387000000022 27.9418000000004 -19.0913000000007
49.8268000000022 25.6313000000004 -16.7345000000008
60.4117000000029 37.9045000000007 7.03889999999902
73.9135000000019 26.0712000000003 -21.1669000000006
77.1991000000019 33.4807000000002 -28.7362000000006
60.7442000000026 53.9930000000004 -16.9677000000009
81.934800000002 38.1042000000002 -31.2323000000007
91.8208000000018 49.8309000000001 -35.0969000000007
103.523900000002 38.8337 -44.0165000000006
105.468700000002 41.4455999999999 -45.9387000000006
110.073200000002 45.1468999999999 -39.9883000000006
116.832800000001 42.6760999999998 -52.5669000000006
15.4704000000036 5.21210000000092 9.42449999999874
10.0158000000038 9.15410000000094 8.15849999999871
39.7167000000032 14.0930000000009 9.37039999999888
49.1062000000025 26.2650000000006 -9.96000000000084
46.1725000000025 42.5829000000005 -21.2265000000008
50.4320000000028 14.8148000000008 11.247399999999
92.7316000000026 29.5136000000005 1.43619999999905
119.990300000002 37.3378000000002 -13.8735000000008
113.050800000002 50.6488 -31.0405000000007
127.340600000002 39.5751 -24.4337000000008
112.984400000002 43.1424999999999 -38.3192000000006
15.9338000000035 3.75660000000092 9.57559999999878
13.4435000000032 -2.42619999999915 8.43929999999881
41.1493000000031 9.47450000000085 9.63589999999891
55.0751000000021 5.09480000000055 -9.85220000000084
61.621900000002 -10.0005999999995 -21.0265000000009
122.681300000001 23.023 -30.8453000000007
117.922100000001 29.3907999999999 -39.5423000000005
ID=PROcriMALSAFUSNM382515

LM3=54
13.1762999999998 2.06420000000016 -3.35790000000048
12.7762999999994 -7.50650000000023 -3.7196000000002
45.7551999999992 -3.31470000000012 -12.3439000000001
54.6745109875297 -2.38935380744929 -17.8318063052916
41.513799999999 13.4789999999998 -14.9188
57.7965999999997 6.01309999999982 -19.3452000000002
57.124 5.52160000000026 -18.2957000000004
70.0013999999997 5.43530000000014 7.6661999999997
62.8012999999989 21.0281999999999 -24.8757
76.2951999999992 20.8858 -20.0235000000002
83.3888999999991 18.6161000000001 -27.2522000000001
82.0388999999992 -10.2271999999999 -15.3845000000002
89.490399999998 16.9686999999998 -29.6380999999998
105.907699999999 13.6978 -31.7229
110.223199999999 28.5646000000001 -41.8013
112.537399999999 29.1469000000001 -42.0601
114.751599999998 38.5915000000001 -44.4598999999999
121.316999999999 41.2718000000002 -34.8856000000001
118.630399999999 27.6147000000001 -35.0042

123.045999999999 34.2811000000002 -44.1454000000001
11.7914999999996 5.84540000000001 -3.45000000000017
6.34219999999948 14.3652 -4.34130000000009
34.2765999999991 29.6458999999999 -12.1888
42.7082999999992 34.1202999999999 -17.8886000000001
49.1031999999993 30.7481999999999 -19.4527000000001
48.4276999999992 28.8498999999999 -17.6367000000001
58.9790999999998 39.7755000000001 7.29359999999972
73.7690999999992 28.964 -19.8374000000001
77.3607999999992 35.4722000000001 -27.5328000000002
59.9772999999993 58.5555000000001 -15.4227000000001
81.593099999999 40.1949000000001 -29.4106000000001
91.5811999999988 53.0172000000001 -31.3417000000001
104.417299999999 43.9282000000002 -41.7182
107.732799999999 44.7541000000002 -41.5928
111.071299999999 50.2946000000003 -34.4329
117.936299999999 46.7944000000003 -45.4003
15.6286999999997 5.78900000000002 8.7660999999998
10.1334999999998 9.11060000000005 8.16449999999979
35.0103999999996 14.657 9.21629999999977
47.7779999999994 29.1866 -9.40590000000015
44.9689999999992 46.5904999999999 -21.0500000000001
53.1736999999998 17.7900000000001 11.4718999999997
90.5463999999996 29.9787000000002 4.49099999999972
121.120699999999 40.7317000000003 -10.3950000000002
112.989299999999 53.4880000000002 -24.2257000000001
126.484499999999 42.7494000000003 -15.8841000000002
113.341499999999 47.4064000000003 -32.7602000000001
16.4066999999996 4.59940000000003 8.75089999999981
13.2939999999996 -2.18840000000002 7.82309999999982
37.3803999999996 9.03250000000003 9.38319999999976
56.0691999999998 5.45900000000014 -9.45890000000029
62.185999999999 -9.24950000000005 -22.2664000000001
123.125699999999 26.8735000000002 -24.1145000000001
118.846999999999 32.9093000000002 -32.6847
ID=PROcriFEMBOTUSNM429175

LM3=54
13.0124 2.4702 -2.6255
13.6956 -7.091 -4.2229
43.7332 -1.6511 -11.1676
52.9513 0.3677 -16.0597
39.732 14.5602 -12.5283
56.2793 6.7516 -19.1554
54.8013 8.3943 -15.7993
66.3858 4.9665 9.3365
59.9309 21.7757 -23.182
72.8369 23.2699 -16.724
80.3807 21.4679 -23.9442
78.3716 -7.8849 -12.5381
85.7893 19.8638 -26.41
100.5686 15.9601 -28.4393
105.0501 31.6512 -37.5441
108.1608 31.4957 -38.0378

110.4344 40.812 -44.5549
116.1118 43.2559 -32.2783
113.4662 28.9089 -33.4
118.2772 35.8575 -44.3382
11.7674 7.0597 -2.328
5.9501 14.3445 -4.7409
33.4819 30.2168 -11.6182
39.9732 33.403 -15.536
46.5956 31.5365 -19.1445
46.6282 29.8965 -16.3499
53.9823 40.1352 8.4305
69.149 29.7114 -17.6896
73.6735 36.9617 -24.7624
53.6536 56.9651 -13.0414
76.7473 41.5763 -26.6169
85.628 53.6855 -29.1687
99.729 45.2505 -39.0109
101.9414 46.3466 -39.4122
104.7568 51.5335 -33.4764
112.5107 48.9271 -44.955
13.8 6.2347 9.8438
6.5519 9.3134 8.1266
44.1072 18.0601 12.261
47.1804 28.5712 -8.5674
43.4738 44.4699 -22.7362
47.3221 16.5128 12.926
90.123 33.1487 6.8959
116.918 43.5619 -7.2332
104.4386 55.9403 -24.2934
121.9021 44.9696 -14.0749
109.537 49.0496 -31.6235
14.6441 4.4195 9.9505
11.0823 -3.7241 7.9623
45.0787 13.9537 12.2134
54.2647 8.6624 -9.0488
61.051 -5.6905 -22.7246
116.0522 26.4632 -24.3424
114.2665 33.9603 -31.4095
ID=PROcriFEMSAFUSNM469885

LM3=54
12.0939 2.4167 -2.1513
12.4366 -6.8464 -3.9232
40.3961 -3.6711 -9.6713
49.1212 -1.8692 -15.7103
39.2979 13.1399 -11.8343
52.273 5.0846 -17.4412
50.8359 5.9095 -15.5888
65.5532 3.4527 6.1237
57.6752 19.0524 -22.6745
70.2113 18.7828 -18.3224
77.2408 16.3896 -25.8895
74.2448 -9.2366 -13.8591
83.3087 15.8779 -27.8372

97.3598 11.4382 -30.0026
100.8615 25.9205 -39.7068
103.6934 27.2136 -40.4424
104.8121 34.5088 -44.3105
112.4978 36.7911 -35.1071
109.5456 24.5492 -35.1252
112.459 30.0705 -45.3288
11.1981 6.1966 -2.1692
5.6739 13.7265 -3.2975
31.6903 27.7147 -9.3976
39.0101 31.1169 -15.5458
45.2136 27.5308 -16.4952
44.468 26.427 -14.8848
55.8889 38.2278 5.4246
67.6543 27.0931 -18.4951
71.7828 33.9248 -25.3523
54.8998 52.8756 -13.1398
76.2245 38.0789 -27.1082
85.9356 48.375 -28.7828
96.5177 39.806 -39.928
99.1686 41.4511 -39.2669
102.5876 45.5856 -34.4691
107.1212 42.7838 -45.8283
14.166 4.9633 9.0883
8.2995 8.5426 7.8453
34.6394 13.2536 9.3711
45.273 25.5869 -8.6839
41.9306 40.6521 -19.8331
48.0748 15.1611 10.865
87.1353 27.5057 3.5854
111.3395 36.0542 -10.5146
103.8091 49.0215 -24.4459
116.2966 37.5402 -16.7022
105.2413 43.5928 -32.8913
14.8147 3.6672 9.1339
11.3549 -2.5036 7.944
36.0263 8.125 9.4013
51.2705 6.5307 -8.7472
57.7095 -8.199 -20.0164
112.7184 22.9897 -23.8945
110.8469 28.0834 -33.1575
ID=PROcriFEMSAFUSNM469886

LM3=54
13.7235000000106 3.27920000000367 -2.64700000000329
12.8395000000112 -7.34859999999627 -4.67780000000331
44.4784000000073 0.131300000002586 -10.4538000000021
53.3481000000063 1.0176000000023 -15.7189000000018
40.5213000000062 16.7409000000022 -13.4123000000018
56.0209000000056 8.72860000000209 -17.9652000000016
55.5064000000055 10.1548000000021 -16.4377000000016
67.6470000000059 7.81120000000212 10.3931999999983
58.4543000000039 23.5991000000015 -23.1703000000011
75.5943000000031 25.0429000000011 -19.4378000000008

81.5196000000028 23.4637000000011 -27.2031000000006
81.2566000000055 -6.78839999999793 -11.5168000000014
88.269800000003 22.6712000000012 -29.7081000000006
108.076900000003 19.2065000000013 -33.3437000000006
108.452000000002 34.0033000000011 -44.4468000000005
111.169700000003 35.4868000000012 -45.6180000000005
111.920900000003 45.1939000000012 -51.5219000000006
119.439400000003 48.0743000000013 -39.8699000000007
118.204200000003 34.6671000000013 -40.0712000000006
121.189500000004 41.0433000000015 -53.0068000000008
12.2685000000109 7.41650000000367 -2.74650000000328
3.48430000001128 15.4340000000038 -5.07740000000342
30.5424000000069 30.9916000000024 -10.7603000000021
39.4823000000058 36.359800000002 -16.1472000000018
46.309400000005 32.5283000000017 -17.8081000000015
46.2728000000051 31.8924000000017 -16.9055000000015
53.2546000000056 39.7217000000018 10.5437999999982
70.9895000000032 34.8687000000011 -19.6694000000009
74.3531000000028 40.8845000000009 -26.4138000000007
52.6125000000049 61.4417000000016 -11.6054000000015
79.0419000000027 45.9340000000009 -29.4855000000007
89.6823000000029 60.7616000000009 -32.9294000000007
100.567300000002 51.084100000001 -44.6790000000005
103.151100000003 52.1856000000011 -45.3050000000005
107.881000000003 57.272800000001 -39.9299000000006
115.129300000003 54.7672000000013 -52.7956000000007
13.2208000000108 5.95370000000362 12.0480999999968
6.88050000001146 10.9120000000038 8.61079999999659
39.939600000008 18.6963000000027 13.3302999999975
46.9791000000054 30.9763000000018 -8.29740000000165
40.6037000000056 47.2622000000019 -20.4524000000017
52.9158000000072 21.2385000000025 15.1408999999978
91.9028000000041 38.6519000000014 4.92889999999879
124.096800000004 50.9774000000014 -11.870400000001
106.584900000003 61.5534000000011 -29.6043000000007
129.001500000004 53.1026000000014 -20.396400000001
111.914700000003 55.210100000001 -39.0077000000006
13.5479000000108 5.13040000000364 12.2296999999968
13.3675000000114 -3.26399999999618 7.74559999999663
41.4954000000081 14.3167000000028 13.5938999999975
55.3621000000057 10.7556000000021 -8.76610000000164
61.6007000000062 -6.17019999999762 -20.0585000000017
122.713664546534 33.7186823448441 -27.6025694132393
117.185200000003 37.6484000000012 -37.2645000000006
ID=PROcriFEMBOTUSNM470162

LM3=54
16.1389000000058 2.27980000000201 -1.59830000000132
14.7666000000063 -8.60509999999783 -4.49560000000189
46.7495000000044 -4.00559999999854 -11.8098000000017
56.3006000000041 -0.721099999998593 -14.7997000000017
44.533300000004 15.3063000000012 -12.4694000000014
60.8933000000034 5.87780000000119 -18.7851000000015
59.5666000000034 7.36620000000112 -16.4651000000015

72.5344000000036 4.08450000000143 9.56769999999837
65.2609000000025 22.057600000001 -23.4974000000012
79.4167000000019 23.1503000000009 -19.2832000000011
86.9626988314126 19.7119732562383 -26.0059514743416
83.1044000000035 -10.8370999999984 -14.5183000000019
92.9211000000016 18.4049000000012 -28.6713000000012
107.475800000001 14.2159000000014 -30.2555000000014
112.549300000001 30.5948000000014 -41.0380000000011
115.742200000001 30.5148000000014 -41.1678000000011
118.170600000001 39.1868000000015 -45.282400000001
123.919000000001 41.0669000000016 -35.5198000000011
121.111900000001 27.2303000000015 -34.7813000000012
125.678400000001 34.3539000000017 -46.1648000000012
14.3274000000058 7.72230000000194 -1.7971000000016
6.50390000000615 16.065200000002 -4.04890000000164
34.6377000000041 30.9842000000012 -11.1189000000012
45.6426000000036 35.2289000000011 -15.8201000000011
51.5126000000033 32.675000000001 -17.8738000000011
51.4392000000033 31.753400000001 -16.2086000000011
59.8636000000035 41.0338000000013 9.23019999999877
76.260300000002 31.487200000001 -19.461800000001
81.250600000002 38.6178000000011 -26.884000000001
59.4646000000031 60.1728000000013 -15.027900000001
84.0802000000018 43.8038000000012 -28.642600000001
94.4757000000015 54.8512000000013 -30.6124000000009
107.219900000001 43.6618000000015 -40.6277000000009
110.378700000001 46.0221000000015 -41.4143000000009
112.567900000001 51.2078000000015 -34.4266000000009
120.901400000001 48.9498000000017 -46.894200000001
15.7674000000058 6.18970000000197 11.0016999999984
10.5716000000063 9.69680000000216 9.61439999999831
42.2188000000043 19.3226000000014 12.4116999999986
52.1791000000032 30.8798000000009 -8.98070000000115
45.4167000000032 46.9762000000011 -18.929300000001
57.8163000000036 20.2374000000012 13.9245999999987
90.6565000000021 30.8850000000012 7.60429999999878
126.114100000001 42.2862000000016 -11.0962000000012
110.756800000001 55.1051000000014 -24.7742000000009
130.426000000001 43.3215000000017 -15.1806000000012
116.832600000001 49.9804000000015 -34.8559000000009
16.2796000000058 4.30080000000196 10.8718999999984
14.2514000000062 -1.46689999999785 9.29139999999823
44.5369000000042 9.98950000000138 12.2378999999986
58.888943976309 8.17965139586298 -8.98225139983402
64.6042000000039 -9.73739999999845 -19.4755000000018
120.756500000001 23.3752000000016 -23.6782000000013
123.608100000001 31.3389000000015 -35.0438000000012
ID=PROcriMALBOTUSNM470163

LM3=54
12.0837999999988 1.72749999999859 -1.91779999999856
13.5493000000003 -6.3148000000002 -3.32859999999884
43.787300000003 -1.2530999999992 -10.722
52.9973000000005 0.114499999999942 -14.8305999999994

41.7349999999999 15.5463999999997 -12.1382999999992
56.6627000000005 6.5669 -18.2993999999994
55.1016000000017 7.86040000000045 -15.3028999999998
69.8905000000008 5.66800000000002 7.70710000000055
62.1099000000016 23.0877000000004 -23.3953999999999
73.5489000000003 22.4138000000001 -19.0286999999996
80.4357000000012 20.3654000000003 -26.6997999999998
77.9480000000014 -9.05529999999979 -13.2996999999997
86.4101000000019 19.4803000000006 -27.9690000000001
101.365200000002 15.2946000000005 -30.3489999999999
104.488700000003 29.9574000000009 -39.1684000000003
107.485000000002 31.0856000000008 -40.2924000000002
109.487900000003 39.699200000001 -45.2795000000003
116.702200000003 42.288800000001 -33.6342000000003
113.182000000003 27.7646000000009 -33.0035000000002
118.188200000003 35.703900000001 -44.7761000000002
10.2514 6.71309999999944 -2.20749999999889
5.29630000000137 14.2199 -3.39779999999925
32.4181000000005 29.8911999999999 -10.5636999999993
41.7604000000008 35.0300000000001 -16.0635999999995
47.5063000000008 31.3293000000002 -17.6180999999996
47.1547000000007 28.9275000000001 -14.8487999999995
55.918500000001 40.2239000000001 8.24790000000039
70.9798000000008 30.9531000000002 -18.0463999999998
75.5506000000013 37.8680000000005 -25.8953
54.5617000000016 58.5805000000004 -13.3625999999998
78.8312000000014 42.1906000000005 -27.9425999999999
88.5024000000022 54.4940000000008 -30.4197000000001
100.839100000002 44.1073000000009 -39.4292000000002
102.629800000003 45.561100000001 -39.8660000000002
105.818100000003 51.6271000000009 -32.7739000000002
113.842100000003 48.3311000000011 -46.4666000000003
13.7508000000003 5.99949999999956 11.171200000001
8.024500000001 10.0842999999998 7.94690000000081
34.8278000000006 16.4082999999999 10.7220000000007
46.5158000000006 28.915 -8.57429999999949
41.3413000000013 45.0188000000003 -20.1268999999997
49.1454000000008 18.3666 11.8646000000006
85.9752000000015 31.9615000000003 5.51040000000021
118.436600000003 43.2043000000008 -9.58840000000004
107.600000000003 55.7358000000009 -24.6361000000001
122.877700000003 44.1526000000009 -14.6402000000001
110.530700000003 48.7782000000009 -32.5086000000002
14.1798000000002 4.99409999999955 10.875400000001
12.2906000000002 -2.6853000000005 7.61930000000112
36.7862000000008 10.1114999999999 10.6324000000007
52.6561000000014 7.88410000000028 -8.58389999999969
59.473800000001 -7.47489999999989 -19.7786999999995
116.987500000002 25.4063000000008 -25.0842000000001
115.066700000003 33.0976000000009 -32.3518000000002
ID=PROcriMALSAFUSNM597882

LM3=54
14.2989999999957 2.20299999999744 -2.0325999999993

13.5917999999955 -7.43570000000268 -2.70449999999934
45.4336999999955 -3.5351000000022 -11.8172999999994
55.5462999999961 -1.97250000000195 -16.1081999999995
42.1411999999961 15.5154999999981 -12.2099999999994
56.5450028523085 5.74163603468176 -18.423397709915
55.1022999999963 6.75089999999823 -16.0663999999995
69.0091999999964 5.25369999999813 8.70790000000052
63.2564999999972 22.7295999999986 -25.1588999999995
73.9003999999978 21.4219999999988 -19.5085999999997
81.5958999999983 19.2537999999989 -26.0545999999998
79.5359999999973 -10.7581000000017 -16.4193999999997
87.3361999999987 18.561799999999 -29.3945999999998
102.867699999999 13.4594999999991 -31.8776
104.171000000001 25.9251999999997 -43.2331000000004
107.012700000002 28.0912999999997 -43.2521000000004
107.555200000002 36.5016999999998 -47.5270000000004
114.975300000002 39.7561999999998 -37.9739000000005
114.305700000002 27.8745999999997 -37.0825000000005
115.715000000002 32.9042999999997 -48.5051000000006
11.8994999999956 7.01069999999743 -1.67899999999926
5.53389999999558 14.9254999999974 -2.21999999999923
32.5023999999965 31.6903999999982 -11.5595999999994
41.8338999999967 36.1988999999983 -16.0550999999994
39.5785999999967 34.6330999999984 -20.2693999999994
41.1561999999966 35.1126999999983 -20.0323999999994
55.795199999997 40.0551999999983 8.89490000000055
70.9293999999976 30.2900999999987 -18.7428999999995
75.0455999999985 36.551199999999 -25.9318999999997
53.4699999999983 58.4079999999988 -16.0459999999997
79.4038999999993 41.2073999999992 -29.0839999999999
87.7099000000009 53.4895999999996 -32.0603000000003
97.3759000000016 43.3231999999998 -44.1727000000004
101.413400000002 43.8392999999998 -43.2505000000004
106.092300000002 49.0588 -37.3788000000006
111.081400000002 45.2291999999998 -48.3064000000005
16.1741999999955 5.82199999999734 10.6337000000008
9.65789999999558 8.88889999999735 8.96960000000076
40.0691999999958 17.1430999999978 11.2676000000007
46.3919999999964 30.8808999999983 -8.52879999999936
41.3605999999975 46.3246999999986 -21.5888999999996
53.4830999999959 19.769799999998 13.0336000000007
91.8871999999986 33.9550999999988 5.44930000000024
116.199900000001 42.9899999999996 -11.6816000000003
107.196300000002 55.0682999999999 -28.4246000000006
123.833900000002 45.3823999999997 -22.9322000000005
107.084600000002 47.8946 -35.7104000000006
16.8044999999955 5.12719999999737 10.6427000000008
13.8805999999954 -1.28350000000278 8.71300000000078
40.8304999999958 12.1285999999978 10.9088000000007
55.1737999999961 6.57979999999815 -8.41249999999994
60.2163999999967 -8.73700000000182 -23.2246999999996
117.322900000001 26.0387999999996 -27.7703000000004
113.341000000002 32.5414999999998 -36.1613000000004
ID=PROcriUNKNAMUSNM165121

LM3=54
13.0270000000034 3.86770000000202 -0.586700000000258
13.7916000000035 -5.05079999999805 -1.54800000000024
44.0967000000022 3.42720000000124 -3.21140000000027
57.1203000000016 6.2160000000009 -7.92020000000028
40.0000000000022 18.4989000000014 -4.44280000000019
57.7447000000011 13.4343000000007 -8.26400000000024
56.4240000000013 13.6942000000008 -6.18460000000023
66.1432518943112 12.5609977453417 19.2281602793681
61.0540000000004 28.3160000000005 -9.96120000000015
75.9537999999996 30.8629 -3.33040000000014
83.0779999999987 29.3283999999995 -10.2985000000001
81.1418000000001 0.047599999999969 0.0707999999997828
89.8531999999984 28.9881999999992 -11.9864000000001
104.994999999997 27.7000999999986 -12.3455000000001
109.920399999996 41.9462999999983 -19.2888000000001
111.341499999996 44.2969999999983 -19.4177000000001
113.388599999996 51.8624999999982 -22.2895
116.616099999996 54.5345999999981 -12.2154
117.082799999996 40.8391999999981 -12.6787000000001
122.602199999996 48.2709999999981 -22.0197000000001
11.2507000000034 8.06200000000201 -0.222500000000173
4.87540000000352 14.0790000000022 -0.907200000000165
31.2433000000026 31.9098000000018 -2.75490000000015
40.5869000000018 37.4493000000014 -6.20730000000011
47.4094000000013 34.289500000001 -6.63770000000013
47.2230000000014 33.2881000000011 -5.13920000000014
53.3464000000012 44.5446000000009 19.0548999999999
72.3681999999997 37.8257000000001 -3.04640000000008
75.989599999999 43.8662999999998 -10.1721000000001
52.4536000000004 62.5967000000007 -0.271700000000029
79.8250999999986 50.3676999999995 -11.6748000000001
88.6414999999977 62.1932999999991 -13.1946
103.040799999997 57.0057999999985 -17.9504
105.413899999997 57.7317999999985 -19.8017
106.309899999996 63.3677999999984 -12.5025
115.742699999996 61.8791999999982 -23.1325
13.4802000000035 6.220500000002 12.7527999999999
6.08500000000352 9.49950000000206 9.41809999999986
34.8535000000025 18.2580000000015 18.0395999999999
47.1343000000016 32.0534000000011 1.97989999999988
38.6203000000011 46.0983000000011 -7.97450000000008
45.384600000002 21.6140000000012 21.3220999999999
87.2192999999988 40.8240999999996 20.9839999999999
115.503699999997 54.2758999999984 12.1338999999999
107.238399999997 65.9693999999985 -2.16270000000003
120.538099999996 56.5197999999981 7.96169999999991
108.508899999996 61.0147999999983 -10.5612
13.6614000000036 5.27850000000204 12.8193999999998
11.1682000000035 -2.043799999998 9.06899999999984
36.6882000000026 14.6699000000015 18.0287999999999
53.7430000000017 14.901000000001 2.22479999999978
60.1225000000012 -0.222199999999338 -8.48970000000031

119.628799999996 40.4567999999982 -1.57380000000011
115.928399999996 43.9463999999981 -9.87080000000009
ID=PROcriFEMBOTAMNH169089

LM3=54
15.4784999999976 2.95359999999899 -2.60229999999857
14.9177999999996 -8.27910000000053 -4.1769999999992
46.2389999999996 -3.94680000000055 -11.2945999999991
57.0359999999999 -2.38580000000041 -16.8393999999993
43.3993999999985 14.5454999999991 -14.6678999999989
60.8513999999998 4.33579999999955 -20.0362999999992
59.6841999999997 6.63379999999952 -18.3168999999993
76.1048999999987 3.03299999999912 6.91180000000134
64.6147999999996 22.2340999999997 -25.0837999999992
80.2968000000004 23.9306999999998 -23.0101999999993
88.7557000000002 20.1874999999998 -29.5145999999992
89.1793000000006 -11.2697000000002 -14.6620999999993
93.7187999999998 19.4397999999996 -31.2474999999991
110.4884 14.0329999999994 -34.4929999999989
112.4179 30.4088999999995 -42.9531999999991
116.3882 30.1184999999996 -44.1946999999991
118.7073 39.9410999999994 -49.609999999999
125.5821 42.4001999999994 -38.828899999999
123.7829 28.2236999999994 -39.606499999999
126.295800000001 35.4263999999995 -51.9261999999991
13.5777999999984 7.70199999999905 -2.75839999999896
6.30489999999878 15.933399999999 -4.21139999999907
34.9333999999987 32.4088999999993 -11.098899999999
45.0340999999989 35.5424999999993 -17.913899999999
50.9893999999989 31.3100999999993 -19.781499999999
51.1739999999988 29.4899999999993 -18.016599999999
62.1830999999988 42.9065999999992 7.1539000000012
78.2990999999997 30.0222999999997 -23.1898999999991
82.3678000000004 38.0850999999999 -29.6204999999993
60.4167675235374 59.7644469952436 -15.8596579872499
86.0554000000005 42.3499999999999 -31.3388999999993
95.9199000000004 56.1661999999996 -33.9573999999991
108.4015 44.9066999999996 -42.5298999999991
110.8795 46.6208999999996 -44.2669999999991
115.638700000001 53.2316999999996 -39.4132999999991
122.546 48.4983999999994 -50.700299999999
16.3596999999985 7.15689999999897 10.7940000000011
9.28109999999858 10.826899999999 9.31540000000107
50.1762999999985 18.9154999999991 13.9827000000012
50.6910999999987 30.3434999999993 -9.82259999999892
45.4436999999993 47.7681999999994 -21.401799999999
52.1399999999985 17.3112999999991 13.7998000000013
92.8486999999994 31.6986999999993 3.85440000000122
131.1812 43.8480999999993 -14.1951999999988
117.5242 58.3795999999995 -28.709299999999
134.2711 45.4943999999993 -20.5983999999988
117.533400000001 50.0570999999995 -36.0271999999991
18.0550999999985 4.63929999999897 10.8115000000011
13.9975999999987 -2.86750000000102 9.42290000000108

50.6041999999986 14.8936999999991 13.7956000000012
59.1141999999992 6.1012999999994 -9.91199999999907
64.9451000000002 -10.2358000000003 -22.0497999999993
127.8463 24.8969999999994 -30.4006999999989
123.4264 32.4071999999995 -36.627399999999
ID=PROcriMALBOTAMNH169091

LM3=54
17.3315000000028 2.78780000000096 -3.48160000000156
14.1218000000031 -8.51429999999922 -4.65260000000178
45.2752000000018 -1.10229999999951 -11.2621000000015
56.0310000000014 0.374000000000352 -15.3509000000014
42.4983000000017 15.4459000000005 -13.6922000000011
60.8069000000012 7.31440000000038 -19.6792000000012
58.8026000000015 9.22430000000045 -15.9135000000012
70.9382000000012 6.92040000000017 10.8589999999988
61.4949000000009 22.5310000000003 -23.8959000000008
77.8163000000006 22.7929000000002 -18.0673000000007
83.6976000000001 20.7013 -27.6223000000006
82.7148000000005 -9.48959999999998 -12.9318000000012
90.9043999999998 20.2482999999999 -29.9774000000006
109.0324 15.8402999999998 -33.5831000000005
110.119699999999 31.4786999999996 -43.7355000000001
112.847399999999 32.2282999999996 -44.5449000000001
113.4598 42.2226999999997 -48.5205000000001
121.917 45.2368999999997 -38.6720000000001
119.4526 32.3027999999997 -37.9258000000003
122.165099999999 37.3966999999996 -51.8123000000001
15.2034000000031 8.24880000000079 -3.18800000000158
5.29400000000338 15.1579000000009 -5.03460000000161
33.3630000000026 30.8471000000008 -12.2271000000011
43.3689000000021 35.1126000000007 -15.8027000000009
50.5609000000016 33.0514000000005 -18.8249000000008
50.8424000000016 31.1457000000005 -16.3825000000008
57.905000000002 40.7009000000004 10.9330999999991
73.6889000000007 32.9090000000002 -18.0007000000006
77.1781000000004 39.3563000000001 -26.8661000000004
55.4557000000018 60.7655000000005 -13.6133000000006
81.2117000000005 44.827 -30.0330000000004
92.2150000000004 58.5773999999999 -34.1659000000001
103.6772 47.2094999999997 -44.3083
106.2779 49.5628999999997 -44.639
110.9728 54.0714999999997 -38.6334
116.9438 51.1059999999998 -52.4388
16.7930000000032 7.1257000000008 10.6751999999984
10.1469000000033 10.5627000000008 8.97079999999843
38.5548000000024 17.9433000000006 12.8705999999987
51.0280000000017 30.9936000000005 -8.3762000000009
43.4702000000017 48.6699000000006 -21.1877000000006
55.3833000000021 20.7531000000004 15.8924999999988
94.2390000000011 36.3580000000001 5.62799999999925
120.890300000001 45.0836999999999 -8.29660000000042
112.7352 59.5955999999998 -27.8533000000001
127.167600000001 44.3749999999999 -15.4542000000004

111.04 47.1719999999996 -33.8609
13.0060000000036 3.29200000000085 12.7115999999982
9.81670000000333 -3.75559999999924 10.5409999999983
37.1196000000023 10.0128000000005 14.8889999999986
54.2482000000014 5.48540000000037 -6.18640000000122
59.131000000001 -11.7645999999997 -18.7028000000013
120.0814 24.7501999999998 -25.8415000000004
117.004899999999 32.7831999999997 -33.9155000000002
ID=PROcriFEMSAFAMNH169445

LM3=54
13.6228000000119 2.09420000000267 -2.24520000000316
15.056900000012 -7.38129999999742 -4.12000000000326
46.5610000000086 -2.2177999999982 -11.4629000000022
55.5521000000076 0.271800000001585 -15.3073000000019
44.4895000000075 17.1003000000016 -13.188900000002
60.6886000000063 5.7925000000013 -19.8071000000016
60.5213000000065 7.47030000000133 -17.8489000000016
72.5171000000067 3.86720000000137 7.54229999999824
69.8470000000046 26.4882000000009 -26.7961000000011
80.5039000000035 25.0112000000007 -19.2367000000008
86.0691000000029 22.1588000000005 -26.4561000000007
84.9171000000061 -10.0587999999988 -17.6977000000015
91.2591000000026 22.1941000000004 -29.4440000000006
108.315100000003 17.1940000000005 -34.4923000000007
108.751800000002 32.8219000000003 -43.3837000000006
111.379600000002 33.1573000000005 -44.2600000000007
112.139500000003 42.2548000000006 -47.8091000000009
120.575900000003 44.9401000000005 -39.6481000000009
119.781700000003 32.8711000000006 -36.9953000000008
119.900000000003 38.0803000000006 -49.650300000001
11.3574000000119 7.95350000000263 -1.8766000000033
6.20150000001197 15.9834000000026 -3.41330000000334
34.9506000000083 33.2869000000018 -10.9352000000023
43.6413000000073 38.2931000000016 -16.044800000002
49.7278000000064 35.8789000000014 -18.9627000000017
50.7603000000065 34.5588000000014 -16.5151000000018
57.9792000000071 43.5919000000015 8.41989999999795
76.9236000000037 34.9912000000007 -19.0733000000009
80.518300000003 40.4755000000005 -26.4604000000008
58.5535000000063 64.2696000000013 -17.1986000000019
83.325100000003 45.2980000000005 -28.8525000000008
92.9871000000037 58.5525000000008 -34.7413000000011
103.672200000003 46.8895000000005 -42.5759000000008
106.440500000003 48.2265000000006 -43.4101000000009
111.508900000003 53.6629000000007 -37.7161000000011
115.008300000004 49.7758000000008 -49.9711000000012
16.0891000000118 6.36780000000254 10.3650999999967
9.26760000001227 10.9197000000027 9.44109999999654
38.172300000009 16.8488000000019 11.9598999999975
50.2435000000067 33.6512000000014 -9.90640000000184
43.1087000000071 50.3152000000016 -21.130600000002
52.596000000008 19.1606000000017 13.5191999999978
94.8913000000048 35.566100000001 4.24869999999859

123.224300000004 46.6600000000007 -13.5003000000012
112.999100000003 59.2071000000007 -27.7999000000012
129.636300000004 49.4269000000007 -23.1292000000013
112.331200000003 51.8771000000006 -35.250200000001
16.3104000000118 5.38880000000258 10.7544999999967
13.5047000000122 -1.97149999999736 8.8627999999966
39.4294000000089 12.1874000000019 11.6439999999975
59.0754000000067 8.36190000000136 -10.2395000000017
62.6061000000071 -8.93339999999858 -22.0543000000017
124.106800000003 32.7449000000006 -27.4955000000009
118.450400000002 36.7850000000005 -34.6918000000007
ID=PROcriMALTANAMNH27768*

LM3=54
13.2574 1.8219 -2.0368
14.0784 -7.3975 -2.6406
46.7461 -2.175 -12.8652
53.4482 -1.3649 -17.0263
43.8693 16.5848 -14.9538
59.7938 6.3262 -20.6167
59.0838 7.9034 -18.1916
72.0711 4.6476 7.6808
67.9212 26.0346 -27.5624
77.4216 22.8701 -20.6241
86.2439 21.2801 -28.9961
81.9746 -10.4542 -18.8198
91.6831 20.4478 -31.1125
107.3116 16.5216 -36.7581
108.9079 32.5154 -42.8893
112.3159 35.0255 -44.5477
112.5641 43.0643 -48.1525
120.4532 45.8942 -40.0353
119.206 31.8028 -40.1878
120.0338 38.9917 -51.8736
10.8004 7.5252 -1.8648
5.6438 14.5947 -2.0187
33.1275 33.1976 -12.1542
40.2808 37.3243 -17.2555
48.6716 34.7702 -19.8686
48.9451 33.7465 -17.7754
57.6758 44.8978 7.7027
72.6389 34.6708 -20.5436
78.305 41.584 -28.3547
54.11 62.3495 -17.8003
82.3885 46.3005 -30.4366
90.9893 59.2176 -34.8946
102.5183 49.1958 -43.2569
106.4615 49.3168 -44.1823
109.7729 55.1647 -39.7239
116.8697 51.8418 -50.3232
16.1105 6.3194 10.9299
10.3233 10.476 9.396
39.143 19.3519 11.8203
51.4355 31.4164 -11.2059

41.6241 49.6394 -23.7637
55.0728 21.132 13.4555
94.2169 35.3065 5.0328
121.0919 45.543 -11.9414
112.9698 60.1673 -26.722
129.1036 48.3184 -19.6577
112.9177 51.3858 -36.0326
17.0312 5.6295 10.7228
15.3314 -0.6829 9.6247
41.7486 11.4776 11.7899
57.3279 9.5766 -10.8199
63.5373 -9.8353 -23.9341
122.4922 28.8981 -27.6585
118.3512 36.3237 -36.1418
ID=PROcriUNKKENAMNH34266*

LM3=54
11.3551000000029 1.77010000000068 -3.05390000000077
15.1172000000031 -8.06489999999935 -4.92780000000087
47.321800000002 -1.73859999999976 -13.0302000000008
55.3074000000016 -1.59749999999991 -19.0247000000007
43.8224000000018 16.9680000000002 -16.0559000000005
58.7884000000014 5.38060000000005 -20.5605000000007
57.3706000000017 6.79060000000014 -18.6499000000007
71.5197000000012 5.07909999999988 8.44249999999946
67.4969000000011 26.1267999999999 -29.3491000000004
78.0444000000007 24.7900999999998 -21.2178000000003
84.1842000000002 22.9380999999997 -28.6890000000002
83.6106000000007 -8.94780000000025 -14.3371000000006
91.9312 22.0302999999996 -30.9753000000002
108.8617 17.2988999999996 -33.9254000000001
108.548099999999 33.7657999999994 -43.2109999999997
111.110599999999 33.4380999999994 -43.5033999999997
112.076499999999 43.1917999999995 -47.4358999999996
121.801099999999 46.8834999999995 -37.3750999999996
118.2327 32.2647999999996 -38.9750999999998
118.426099999999 39.1464999999995 -51.1545999999997
9.9762000000033 6.62940000000071 -3.18160000000074
5.66240000000347 16.4047000000008 -5.11920000000073
33.1168000000026 33.6234000000005 -12.6554000000005
40.7255000000021 38.4979000000003 -19.9199000000003
47.2574000000017 34.6330000000001 -20.2085000000004
47.0180000000016 32.2382000000001 -17.7460000000003
56.1325000000019 43.7782000000001 9.32429999999973
74.4090000000008 34.7927999999998 -20.4228000000002
78.0015000000003 40.7239999999997 -28.0611
57.7027000000016 65.3392000000001 -15.1400000000001
82.4415000000003 45.4357999999997 -30.7057
91.9378999999999 60.1918999999996 -33.8528999999997
102.087799999999 49.3195999999995 -43.0799999999996
104.540099999999 49.4570999999994 -44.9058999999995
108.602899999999 55.4977999999995 -38.5063999999995
112.993799999999 50.2519999999995 -50.5779999999995
14.1351000000032 4.74440000000063 10.2939999999993

8.57250000000334 10.2292000000007 8.63439999999934
37.5081000000024 16.6202000000003 9.95699999999946
49.8556000000018 32.4033000000001 -10.5393000000004
42.2108000000016 50.7451000000001 -25.9391000000002
47.380600000002 17.5186000000002 11.8571999999995
93.351600000001 36.0871999999998 4.61079999999986
124.4685 48.2171999999997 -14.2549999999998
112.1036 60.5693999999995 -27.6464999999995
129.0632 49.8082999999997 -19.3108999999997
111.935599999999 52.3309999999994 -36.3866999999995
14.2534000000035 4.24420000000073 10.1621999999992
14.0172000000033 -2.79219999999933 8.70979999999919
40.0960000000023 11.5907000000003 9.81429999999938
57.9449000000015 7.52590000000006 -11.2069000000007
67.6753000000011 -10.136 -24.3910000000007
122.9386 30.6338999999996 -27.5448999999999
118.212199999999 36.0814999999996 -35.8030999999997
ID=PROcriMALETHAMNH81044*

LM3=54
13.2462 1.7743 -3.4367
13.7089 -8.3059 -4.702
45.9043 -2.7175 -11.7113
55.3367 -0.4098 -17.2738
40.9176 13.9849 -13.5809
57.5452 6.3782 -18.9904
56.3649 7.59 -17.5104
69.6324 4.4246 7.699
61.5364 20.9581 -24.0661
76.1378 21.437 -19.9877
85.1381 18.9605 -26.4961
80.4796 -10.4502 -15.5881
89.8637 18.5689 -29.0204
104.4141 13.5539 -31.638
109.1949 29.8404 -40.8162
113.0333 30.114 -41.6204
114.3612 39.4219 -44.4781
121.4709 41.5917 -34.927
120.1905 27.6747 -36.1888
121.8375 34.6113 -46.5142
11.8979 5.9737 -3.5963
5.6698 13.9102 -4.2729
34.2446 29.6875 -12.0205
42.2592 34.4187 -16.5279
48.5886 30.0965 -18.855
48.3112 27.9619 -16.758
56.6429 37.69 8.2106
73.1448 29.4081 -20.1158
77.5315 36.9111 -27.1196
55.3046 57.1053 -15.663
81.6276 40.4828 -28.8525
89.4225 53.1658 -31.2424
104.0871 43.731 -40.7889
107.1504 45.8535 -41.0925

110.4514 52.1596 -36.6911
116.8625 48.0169 -46.9299
15.0451 5.0325 9.4573
8.6903 9.7735 8.7288
37.8072 14.8057 10.1549
47.5427 28.7561 -9.2922
44.1238 45.856 -21.9349
51.8303 17.6248 11.6377
88.8148 29.9937 5.633
121.5788 42.1726 -11.4342
112.598 54.4516 -25.6218
127.4093 43.708 -18.3623
112.0528 49.928 -35.4487
15.2397 4.7827 9.4269
13.3009 -2.8444 8.4813
40.1163 10.8074 10.3147
55.2491 6.9395 -9.5015
61.5353 -7.7186 -23.09
121.8549 26.9575 -25.7041
118.8697 31.7191 -34.9367
ID=PROcriUNKANGAMNH88383*

LM3=54
13.0364999999967 4.08349999999833 0.0372000000002124
13.8012999999981 -5.47250000000018 -1.53289999999985
43.3771000000014 3.71860000000116 -3.76030000000013
53.2759999999992 8.88719999999996 -8.09709999999987
37.0845999999983 20.1680999999997 -4.41869999999976
52.5111999999992 16.0361 -6.22849999999973
56.6882000000003 17.0346000000006 -8.77199999999999
59.4882999999995 12.3760000000001 21.1974000000002
59.5052000000002 32.2027000000006 -12.0176999999999
70.4690999999991 32.5189000000001 -5.11469999999983
78.75 32.6742000000004 -10.8992999999999
79.0781000000001 0.261700000000269 0.132800000000079
86.2732000000006 32.0814000000007 -11.9643
102.4702 30.6789000000006 -10.8685999999999
104.548000000001 48.6055000000011 -18.9764000000001
107.471700000001 49.8963000000009 -17.7223
109.533500000001 58.7966000000012 -21.3217000000001
112.630500000001 60.0867000000011 -9.59090000000007
112.107600000001 47.7103000000009 -11.5514
114.363900000001 56.9253000000012 -22.3455
10.4882999999978 8.70079999999942 0.21760000000008
3.60179999999889 15.4482000000003 -1.74180000000006
26.2616999999985 34.8465000000001 -3.5582999999999
35.5290999999989 40.3622000000003 -7.66599999999991
42.2108999999991 35.8744000000004 -6.53639999999992
42.757899999999 37.8635000000003 -8.46059999999989
43.6518999999992 43.2089000000003 21.7974000000001
66.0952999999995 40.9556000000003 -4.60059999999991
70.0272999999999 47.7242000000006 -10.418
44.3485999999996 66.3289000000008 0.219399999999968
74.6161 53.5121000000006 -11.3289

82.3081000000005 69.0588000000011 -10.6087000000001
98.3870000000009 60.567100000001 -19.1035000000001
100.550100000001 62.2817000000011 -17.7166000000001
102.559400000001 66.9446000000011 -10.6667000000001
110.434700000001 63.9652000000014 -22.6570000000001
11.6690999999981 6.84739999999961 13.8501
4.93069999999861 9.2465 11.0762999999999
29.6140999999988 18.5376 17.7808000000001
39.387499999999 33.7192000000002 0.421000000000128
35.6425999999993 52.1303000000006 -9.53379999999999
40.3536999999992 21.2368000000001 22.4232000000001
79.5623999999999 42.7034000000005 22.5572000000001
108.716900000001 58.1965000000009 14.5977
97.9151000000009 70.3752000000011 -0.570800000000008
114.266700000001 60.7093000000011 9.24849999999996
104.134000000001 65.7524000000011 -9.69440000000008
12.255299999998 5.39429999999959 13.8378
10.7121999999979 -1.84370000000049 10.8157
32.040199999999 14.8049 18.1916000000001
50.091 14.3706000000004 0.617200000000066
62.6087999999997 1.03220000000014 -9.62569999999986
111.889000000001 42.3830000000009 -1.44419999999998
111.231800000001 49.510800000001 -9.19250000000006
ID=PROcriUNKSAFMVZB117841

LM3=54
14.7532000000002 1.15850000000001 -0.472800000000045
13.8132999999977 -4.99730000000015 -2.06179999999964
53.1591000000002 -1.97480000000007 -11.2812999999999
57.2045999999989 1.17319999999981 -13.9696999999997
60.5990000000009 13.7319000000001 -14.1886
70.1336000000036 6.38710000000035 -14.0323000000004
70.3394000000018 7.86770000000011 -15.7312000000001
93.7194999999996 1.47769999999988 3.20159999999996
90.1484000000012 20.5121000000001 -23.7277000000001
96.4119 17.8054 -12.9773000000001
101.3887 17.9781 -16.1456
96.7475000000003 -0.30260000000002 -22.0508000000001
113.2215 15.1942999999999 -20.9445000000001
118.180499999999 6.32489999999978 -19.1415000000001
131.987399999999 18.4934999999998 -21.88
136.352799999999 20.9086999999998 -21.0454
139.131999999999 33.6507999999998 -22.796
149.301499999999 37.2611999999997 -12.7844999999999
150.020099999999 17.2252999999997 -15.6466
147.911599999999 27.7589999999997 -24.9055
13.6641000000003 5.4197999999998 -0.883300000000065
10.2997000000005 10.0683999999998 -2.59670000000001
48.1151000000009 24.3805000000001 -11.0479999999999
51.5735000000009 23.0948000000002 -14.4331
65.1172000000014 24.4410000000002 -13.4191000000001
65.7310000000011 23.0434000000001 -16.3262
85.248200000001 40.8010000000001 3.21539999999995
94.0126000000005 26.1918000000001 -12.7805

99.4292000000006 27.1963000000001 -14.7648000000001
86.167800000001 42.6038000000001 -22.1665000000001
109.1326 35.5715 -20.4967000000001
107.5465 47.1186999999999 -18.0141
124.3605 41.8720999999999 -21.8288
130.426899999999 44.1429999999998 -21.6034
141.209999999999 54.2388999999998 -14.8268
143.349299999999 42.0062999999998 -24.4562000000001
13.0932000000002 3.94499999999988 14.8096999999999
18.4793000000007 14.4017999999999 11.3654999999999
57.9928000000014 23.9460000000002 11.8318
70.0496000000013 34.9250000000002 -7.70310000000006
72.4869000000012 37.9443000000001 -16.4175
74.455800000001 16.5533000000001 18.7319999999999
117.5215 27.5629999999998 15.5922
132.912399999999 31.7965999999997 11.9797
127.2546 56.7029999999998 -7.29140000000002
145.412699999999 35.5188999999997 3.52710000000004
141.356599999999 47.7961999999998 -11.6939
13.2204 2.26529999999985 14.3224
22.8513999999996 -4.82940000000008 11.2850000000001
63.542500000001 3.1843000000001 12.2906
80.6300000000023 -1.31019999999976 -6.69800000000019
83.8641000000017 -2.75629999999985 -16.1238000000001
141.805999999999 8.88589999999969 -7.0904
149.363399999999 23.1838999999997 -10.9389
ID=SMUgigUNKGHABMNH952938

LM3=54
6.54579999999988 1.32970000000011 -0.465500000000116
6.16490000000164 -1.21979999999956 -2.05900000000053
33.8341000000002 -0.224199999999543 -11.3778000000001
35.1118999999993 0.935200000000097 -13.6582999999999
39.7513999999996 11.2266000000001 -13.9182
42.9745999999998 5.27310000000009 -13.496
42.6286999999991 5.60780000000004 -13.9903999999998
61.9769 0.83890000000024 1.55849999999998
57.2745999999996 16.1309999999999 -20.1964
66.1598 16.7278000000001 -14.0603000000001
68.2668000000008 13.0623000000003 -17.5335000000002
67.2500000000006 0.486300000000344 -19.0224000000001
75.3476000000002 12.8761000000002 -18.5842000000001
78.5742000000001 5.67720000000022 -16.5051000000001
88.1006000000004 16.8115000000002 -16.7864000000002
94.4812000000001 15.5087000000002 -15.3870000000002
93.9443000000005 26.9101000000003 -19.1380000000002
101.889600000001 30.1792000000003 -6.46300000000025
101.269100000001 14.2889000000003 -12.0793000000003
101.391200000001 20.2287000000003 -19.1599000000003
7.25709999999995 3.5568000000002 -0.166500000000087
4.80020000000004 5.2529000000003 -1.15980000000013
28.6637999999998 16.8765000000002 -11.0674000000001
30.2397999999997 16.9780000000001 -13.5308
38.8569999999996 18.04 -13.26

39.1373999999997 17.4475000000001 -14.2855
52.7818999999999 31.5947000000001 0.725899999999954
64.4362999999999 20.4742000000001 -14.9598
64.5044 24.8334000000001 -18.1732000000001
56.9688999999998 34.1759 -19.6646
70.7655000000001 28.4738000000001 -19.0424000000001
69.0116000000001 35.1046000000001 -17.3525000000001
83.3822000000003 32.8348000000002 -16.8431000000002
88.9567000000005 37.0790000000003 -15.3131000000002
93.1041000000005 41.8844000000003 -11.5176000000002
97.6428000000006 37.6576000000003 -18.2892000000003
10.1957356298618 3.95213791088962 9.82130903819524
11.4155000000001 9.26080000000031 6.85119999999984
30.9746999999998 16.6441000000002 7.1973999999999
41.8493999999997 26.3564 -10.6973
44.2218999999998 27.9034 -14.1302
51.3253 13.9129000000002 11.9083999999999
77.1600000000002 22.1046000000003 14.6028999999999
87.5160000000003 24.7728000000003 13.4100999999998
86.4979000000004 44.6152000000003 -5.85210000000019
95.9588000000004 27.5184000000003 7.13509999999976
94.4929000000005 40.0958000000003 -8.51890000000025
10.6900026623273 2.2791916979232 9.67930991319217
13.4806000000007 -1.86899999999961 6.09429999999968
34.8023000000002 0.904800000000334 6.52949999999984
50.1865000000001 -2.33389999999978 -10.6156
52.6347000000002 -2.14339999999975 -13.5798
96.9021000000005 6.97260000000031 -4.92640000000024
102.805100000001 15.6930000000003 -8.96460000000029
ID=SMUgigUNKCONKMMA06M591

LM3=54
8.07080000000226 0.598400000000986 -0.513300000000533
8.82260000000268 -2.78739999999879 -0.62090000000052
41.9697000000018 -0.603199999999251 -12.7888000000004
44.560200000002 1.17450000000078 -14.6987000000005
49.7762000000018 12.7543000000006 -14.2323000000003
51.4406000000018 3.89170000000065 -12.4771000000004
53.5452000000018 5.61290000000064 -15.1290000000004
76.9226000000018 1.03090000000076 1.44169999999961
74.0640000000026 19.3136000000008 -22.6182000000004
82.8680000000023 17.7377000000008 -14.5239000000004
86.6264000000023 14.6214000000008 -18.5950000000004
80.177600000002 -0.293699999999187 -22.1299000000005
94.3925000000023 13.6920000000009 -20.1699000000004
98.2587000000023 4.908800000001 -18.2131000000005
110.510600000003 17.7558000000012 -19.2272000000005
116.844200000003 17.7239000000013 -19.2792000000006
119.850000000004 30.1469000000015 -21.7230000000007
128.583500000004 32.3292000000017 -10.5142000000007
128.931200000003 14.0500000000016 -14.7494000000007
128.071300000004 23.9327000000017 -21.9948000000007
6.82340000000238 3.40160000000109 -0.0727000000005262
5.069257236208 6.36063760918292 -0.660718960221522

36.9172000000021 20.6957000000007 -13.0795000000004
39.202600000002 19.3894000000006 -14.6264000000004
48.7087000000024 20.6648000000007 -12.8333000000004
49.1517000000023 20.0083000000006 -14.8770000000004
67.3830000000032 34.293700000001 0.0878999999995254
81.4753000000027 24.4390000000009 -14.8990000000004
82.688600000003 28.5346000000009 -19.0847000000005
70.0019000000035 39.010000000001 -22.1837000000005
89.3465000000035 33.1401000000011 -20.7420000000005
89.2982000000038 41.4499000000013 -19.3400000000006
105.847400000004 36.8652000000015 -19.7947000000007
110.555900000004 40.3865000000016 -19.3930000000007
119.990300000004 49.0801000000018 -14.5210000000007
123.461100000004 39.8432000000018 -21.5683000000007
12.7225000000026 3.88770000000116 11.0464999999995
14.1894723382293 9.72820588304966 8.14666770752656
42.2774000000022 21.6437000000008 6.21089999999957
52.7238000000029 31.5235000000008 -11.0584000000005
54.990800000003 32.8179000000008 -14.4001000000005
63.5291000000023 15.8828000000008 12.8171999999996
92.8979000000033 22.7632000000013 13.7639999999995
109.379800000004 27.1330000000015 11.5446999999994
107.229600000004 48.7372000000016 -6.37930000000069
122.518400000004 30.6684000000016 3.9728999999993
121.830900000004 42.7376000000017 -11.9932000000007
13.2429000000025 2.60060000000112 10.9358999999995
18.2704000000022 -2.44589999999894 8.06789999999956
47.0233000000017 0.694200000000748 5.91269999999964
60.7964000000016 -2.52979999999933 -10.6245000000004
64.2719000000018 -1.90529999999927 -14.4623000000005
118.138500000003 8.03000000000133 -6.28530000000056
127.254100000004 19.4912000000017 -13.0790000000007
ID=SMUgigUNKCONKMMA19113*

LM3=54
13.4884000000013 1.16620000000006 -0.915200000000084
9.48269999999933 -3.39999999999989 -1.22269999999963
51.3467000000006 -0.198299999999911 -14.3023
55.7227999999994 1.89229999999993 -15.7883999999998
59.3284000000011 14.6955000000003 -14.7294000000001
64.2074000000035 6.79860000000045 -14.9447000000003
64.5739000000018 7.62310000000022 -16.6579000000002
86.6115000000003 1.31739999999995 2.94539999999993
86.3308000000017 21.3935000000001 -23.0481000000002
92.6069000000007 17.6631 -14.9576000000001
96.446700000001 15.9275 -18.5950000000001
90.920600000001 0.295299999999999 -23.7025000000001
104.203600000001 15.0214999999998 -19.8358000000001
108.6377 6.28079999999974 -17.2040000000001
121.292600000001 19.5510999999997 -17.6659000000002
124.587900000001 18.9325999999997 -18.8500000000002
130.838400000002 32.7107999999997 -18.6276000000002
136.870800000002 33.8751999999996 -7.52520000000017
137.785800000001 16.9626999999996 -11.8861000000002

139.802100000002 26.6163999999996 -18.0371000000002
12.1332000000016 5.35190000000008 -0.398800000000045
6.87800000000191 7.80670000000004 0.0451999999999411
44.8305000000013 24.1746000000003 -13.2244000000001
48.7352000000013 23.6878000000004 -15.6723000000001
59.9947000000016 23.7497000000004 -13.9236000000001
60.6474000000013 23.0454000000003 -16.5100000000001
77.4711000000017 38.4258000000002 2.52509999999982
90.4529000000012 27.0920000000001 -14.7363000000002
92.8334000000013 30.8996 -17.8364000000002
80.0317000000017 42.8866000000001 -22.2459000000002
98.9543000000013 35.3842999999999 -19.7347000000002
99.0940000000015 45.1412999999999 -16.6466000000002
117.018600000002 39.7568999999998 -17.2852000000002
119.824700000002 41.1888999999997 -18.2690000000002
130.323000000002 50.6878999999997 -10.8566000000002
136.122900000002 41.5827999999997 -18.0084000000002
15.7043000000016 3.21390000000011 12.3978
15.949000000002 10.6096000000001 8.7986999999999
48.568700000002 21.9221000000003 8.27729999999988
62.9494000000017 36.1751000000004 -9.82920000000018
63.8585000000015 37.7879000000003 -15.1540000000002
72.6125000000016 16.9075000000002 12.4969999999998
104.509800000001 26.0815999999998 14.0221999999999
118.145900000001 29.4851999999997 11.8356999999998
118.757300000002 53.2052999999997 -4.71740000000021
129.655900000002 32.4979999999996 5.39739999999984
134.371100000002 45.4346999999997 -10.6199000000002
16.2582000000014 1.78070000000011 12.7161
20.5664000000009 -4.62789999999985 8.07250000000007
57.9749000000015 3.35870000000023 8.47719999999994
72.5566000000022 -3.8660999999997 -11.1765000000001
75.3612000000018 -3.82019999999978 -16.7469000000001
128.830900000001 10.8217999999996 -4.08250000000012
138.901100000001 22.9244999999996 -9.08770000000015
ID=SMUgigFEMCONKMMA25479*

LM3=54
9.4961 2.5329 -0.8166
7.5745 -1.3813 -1.5219
42.1397 1.1414 -14.3928
43.0244 2.4026 -15.3729
48.0401 13.4132 -13.5942
52.9284 5.9805 -13.7572
54.3621 6.8706 -15.2697
74.0022 1.7391 0.5457
71.0536 18.9343 -21.3004
79.7289 16.7355 -14.0435
83.9752 14.2737 -17.6699
80.0066 0.523 -21.0397
92.3079 13.7086 -18.6678
96.0113 6.3779 -16.39
105.6603 17.978 -15.857
111.0958 18.5929 -15.9858

115.243 30.9343 -18.073
121.5544 33.0874 -5.1339
121.8186 15.1133 -11.1743
123.7569 25.6357 -17.4283
9.486 4.8206 -1.6685
6.557 7.5609 -2.6533
37.8167 21.6207 -14.8833
39.1656 20.0134 -16.28
48.842 21.3047 -13.8563
49.7427 21.1508 -15.5882
65.246 36.4864 0.9067
77.7576 24.2968 -14.0176
80.6709 28.4552 -17.5974
70.1181 39.6963 -21.6186
87.4496 32.968 -18.6883
87.3287 42.2589 -16.4052
100.873 36.9384 -16.0102
105.5557 40.252 -16.2685
112.9587 48.3489 -11.2368
119.0959 40.1875 -17.8226
10.2455 5.0515 8.6816
11.7022 10.0081 5.1555
40.8315 22.2508 4.1325
51.3696 31.8849 -11.5146
54.5413 33.2219 -15.396
57.4286 15.835 11.1379
89.7028 24.7719 14.5484
100.7258 26.8252 13.3774
102.2444 49.788 -1.7977
115.1396 31.1742 6.7069
113.8433 43.4952 -8.3842
11.4414 2.9034 8.808
14.9475 -1.0158 6.347
45.7715 2.5013 4.3925
59.6404 -1.6396 -11.2298
63.3 -1.0967 -14.9109
112.5656 8.3684 -1.5069
120.7504 20.1161 -8.8612
ID=SMUgigMALCONKMMA27982*

LM3=54
12.0203 1.1727 -0.7603
10.5681 -3.8998 -0.8271
49.5 0.0464 -13.9837
52.7073 1.7421 -15.5357
52.3255 12.822 -14.1009
63.2311 6.3802 -15.3167
61.0365 7.0601 -17.0841
80.8768 1.0998 1.487
80.3498 20.3492 -23.8123
86.3198 17.467 -14.922
91.3226 14.8972 -18.5958
84.5223 0.2539 -23.1304
98.6992 14.2002 -19.6795

104.5777 6.8698 -17.5015
115.4909 18.4939 -18.4898
119.614 17.0409 -17.89
123.6259 32.2525 -20.0325
132.0466 33.993 -9.1867
132.737 14.5221 -13.5362
133.7789 25.723 -19.4887
10.9727 5.084 -0.6029
8.337 8.7161 -0.7142
40.7831 21.2965 -12.6706
44.678 21.3694 -15.3573
59.0898 23.2862 -14.8001
57.6608 21.4592 -16.4246
70.1869 36.0852 0.9602
83.4507 24.7711 -14.8781
86.7389 30.0689 -18.181
74.0114 39.9988 -23.0348
93.1612 34.7041 -19.6777
94.1321 44.4781 -17.5752
110.356 40.5428 -16.9382
113.5365 43.7984 -18.1009
124.2087 51.6201 -13.393
129.3613 43.0448 -19.4532
12.2483 3.3909 13.9658
13.2388 10.3502 9.2955
44.2782 22.7891 7.6316
58.1865 31.9433 -8.7437
59.3483 34.7065 -17.0491
61.4048 15.3925 14.9513
98.0834 25.37 15.8028
112.0234 27.1212 13.9218
113.2529 53.7341 -6.2936
126.9024 33.146 5.4285
125.4871 44.8477 -11.5258
12.7224 0.9721 13.8471
17.7655 -3.7585 9.2397
50.736 0.8625 7.4398
67.1558 -0.6801 -8.826
69.8401 -2.6058 -18.0363
124.5981 7.8581 -5.7507
131.1451 20.4053 -12.7057
ID=SMUgigMALCONKMMA2954**

LM3=54
11.6709 1.3283 -0.1538
10.5094 -2.3896 -1.2002
44.7658 -1.4591 -14.6956
44.9092 -0.3427 -15.4518
52.4377 13.4331 -15.1456
58.3079 5.3979 -14.4026
55.6379 5.2587 -15.9622
80.3624 0.5735 0.8698
75.7298 19.257 -23.35
86.1004 18.278 -16.3517

88.8461 14.3261 -19.4399
83.123 -0.3891 -20.8305
95.9005 13.3445 -21.5475
100.7845 6.7644 -19.0547
110.4382 18.4187 -20.4675
116.5479 17.8558 -19.6024
120.3207 29.9527 -21.8916
127.25 31.109 -10.544
127.543 15.117 -16.566
127.7336 23.8237 -23.5784
10.8972 4.4653 -0.7251
8.5125 7.4905 -1.3338
37.1338 21.2657 -13.8062
38.7174 20.9086 -14.905
53.8148 22.2108 -14.548
51.253 21.5207 -15.9632
72.3595 36.682 0.2987
84.7656 23.8872 -16.4133
85.1218 28.4281 -19.8518
73.1144 39.8227 -20.6185
90.5564 33.3826 -21.2457
92.4916 41.5506 -19.0802
106.1831 35.3679 -20.3976
111.7495 38.9641 -20.1394
121.1616 46.7381 -16.0775
124.9752 38.8496 -22.5699
13.7293 4.1412 11.1746
15.2136 10.9992 6.8004
46.7073 21.8982 6.2247
54.9436 32.3264 -12.1388
57.1064 34.029 -14.3442
62.8803 15.2296 11.5697
98.7793 23.6212 14.0493
110.5739 26.6977 11.55
110.3262 50.3821 -9.2234
123.1365 30.2846 2.0167
123.1318 41.5595 -14.2489
14.3686 2.2296 11.4238
19.0547 -3.1646 7.5372
51.7867 2.2598 6.1758
63.7688 -2.5255 -11.154
66.1567 -3.1654 -14.6842
121.028 6.874 -7.7845
127.4243 19.5114 -13.8478
ID=SMUgigFEMZOOKMMA35717*

LM3=54
10.9709999999964 1.38849999999826 -0.664399999998988
9.63749999999593 -2.44730000000166 -1.63649999999907
36.650099999998 0.259599999999159 -10.2842999999994
39.8150999999983 1.76719999999929 -12.5497999999994
44.2665999999988 12.3651999999995 -11.5612999999996
48.874499999999 6.17019999999949 -11.4644999999995
48.066899999999 6.81779999999951 -12.8088999999995

69.9126000000004 0.581999999999903 2.53500000000027
63.1860000000017 17.9557000000003 -18.0739
72.8268000000022 16.8649000000005 -13.0781000000001
77.9408000000026 15.4326000000006 -16.8461000000001
73.0183000000014 2.43650000000017 -18.4997999999998
84.6507000000035 14.5531000000008 -17.4946000000003
90.3422000000035 8.16490000000079 -15.7634000000002
100.031100000005 18.4405000000013 -16.4715000000006
106.045200000005 17.7625000000014 -15.8508000000006
106.087500000006 30.6835000000017 -18.3426000000008
115.765100000006 34.2139000000018 -5.31770000000079
117.970600000005 13.9462000000014 -12.3841000000006
115.024400000006 24.2986000000016 -18.3491000000007
10.8673999999957 4.99559999999833 -0.340699999999211
7.97069999999608 8.27819999999844 -0.772599999999202
31.5845999999982 19.1042999999993 -9.78939999999962
34.9426999999985 19.2188999999994 -12.6623999999996
45.0749999999995 19.8520999999997 -11.2705999999997
39.9104999999995 18.6667999999997 -12.0453999999998
59.9728000000013 36.2115000000003 2.33609999999988
71.1926000000023 23.3750000000006 -12.4948000000001
74.9412000000029 28.3569000000007 -17.0882000000002
64.1921000000021 37.2742000000005 -18.9134000000002
80.6531000000037 32.7570000000011 -17.5431000000004
82.3242000000041 42.3269000000012 -15.6165000000006
94.9774000000055 37.9432000000016 -15.5622000000007
100.891300000006 41.5775000000017 -15.2266000000008
107.065400000006 49.7859000000019 -11.9657000000009
109.920100000006 39.0564000000019 -18.7164000000008
11.8658999999959 4.46519999999838 10.3549000000008
13.770699999996 9.39599999999847 7.34030000000078
41.4794999999981 17.9424999999992 10.2809000000004
46.0159000000001 29.1833999999999 -6.89219999999989
47.5220000000004 30.8639 -11.7358
56.1454999999994 15.5612999999996 13.1055000000003
83.1883000000025 23.3711000000006 15.8860999999998
99.5116000000037 27.216200000001 13.2999999999995
94.9667000000056 49.8461000000017 -3.94080000000073
110.415000000005 31.0869000000015 7.8124999999993
110.080600000006 45.6584000000019 -8.82840000000086
12.7322999999959 1.33719999999838 10.6183000000008
16.9046999999962 -2.17190000000149 7.37100000000082
44.3622999999978 4.75989999999909 10.1060000000006
55.0083999999991 -1.45720000000047 -8.10439999999945
56.8906999999995 -1.14860000000038 -11.8999999999995
108.494700000004 8.8575000000011 -4.6596000000005
117.316400000005 18.1195000000015 -9.51550000000064
ID=SMUgigUNKLIBKMMA38194*

LM3=54
-4.79309999999615 -4.35939999999902 -14.2181000000008
-2.97690000000382 -8.0915000000002 -15.3311999999992
39.0793000000016 -2.69319999999988 -22.5849000000001
42.3736000000019 -0.147199999999332 -23.3484000000003

44.3764000000005 12.7308999999999 -21.8725999999999
48.080600000001 5.03419999999999 -20.1573000000003
50.1938000000006 6.04330000000003 -22.3680000000002
69.1775000000012 0.424900000000304 -2.2777000000002
71.7701000000009 21.3108 -24.8359000000001
75.4985000000008 18.6394 -15.1443000000001
81.2179000000002 16.6572 -18.3916000000001
74.8319999999999 -0.328500000000029 -24.9801
89.2031000000002 15.6439 -18.6902999999999
91.4249999999998 5.9102 -17.9707999999999
106.5738 20.371 -14.8882999999999
110.6653 19.8524000000001 -15.9086999999999
114.8174 34.8188 -14.2247999999998
121.4993 36.2475000000001 -3.69419999999986
121.9952 18.9470000000001 -9.34869999999982
124.6264 29.1604000000001 -14.6993999999998
-5.89849999999686 -0.610399999999242 -14.7607000000004
-6.94599999999752 2.5116000000006 -15.9579000000003
31.4374000000019 22.6614000000002 -22.0257000000002
34.1117000000016 21.0573000000001 -22.6636000000001
42.3193000000011 20.6975999999999 -19.8734000000001
44.360400000001 21.0590999999999 -22.0921
56.8319000000018 36.5205000000001 -2.61690000000014
72.7302000000009 26.5965 -15.1684
76.755300000001 30.1937 -18.3371
61.9364000000013 41.2249 -25.1241000000001
83.0703000000007 35.4573 -19.226
79.0229000000011 43.8253 -18.698
99.7388000000004 40.863 -15.3571999999999
103.705300000001 43.0530000000001 -15.3310999999999
110.4453 50.5262000000001 -7.70799999999987
119.6994 45.2656000000001 -14.9966999999998
-0.0101999999984629 -0.0598999999995248 -0.0804000000001017
-3.96519999999723 5.37580000000064 -4.70810000000035
30.1495000000023 21.3015000000003 -2.14280000000022
44.8205000000017 33.8081 -15.6455000000001
44.6416000000016 33.8036 -23.6802000000001
48.8883000000016 13.5840000000002 6.77189999999984
81.5947000000009 24.8929000000002 17.6662
98.1229000000007 29.7451000000002 17.2764
99.0159000000007 54.0446000000001 -1.51389999999995
114.3943 34.9394000000001 10.4526000000001
115.9074 46.8557000000001 -5.30209999999986
0.906800000001132 -2.01819999999959 0.867599999999936
0.944900000000243 -8.36799999999973 -4.27559999999993
34.8300000000012 -2.09999999999985 -1.78290000000009
58.0574000000002 -4.12760000000004 -16.3187000000001
57.7147999999998 -4.26940000000007 -24.5617000000001
112.8837 10.2197000000001 -0.142199999999872
122.7892 26.5614000000001 -5.5411999999998
ID=SMUgigFEMDRCAMNH53846*

LM3=54
12.0048 0.3239 -2.214

11.455 -3.632 -2.728
47.8323 -2.5492 -13.9576
50.6588 0.5843 -16.087
52.7149 11.9709 -13.9423
59.8961 5.2743 -14.7817
58.6678 5.2091 -16.1285
78.5908 -0.0532 0.8599
79.2264 18.7867 -23.3361
85.8503 16.3404 -15.1178
88.4126 13.9478 -18.5197
81.9087 -1.7387 -21.0542
96.6311 12.0993 -20.0827
97.7342 4.1741 -18.8907
113.2891 15.9401 -17.7342
118.6646 16.6419 -17.788
122.5473 29.4757 -19.8632
127.8932 31.5061 -6.9026
126.7849 12.8337 -12.0621
130.8292 21.9492 -19.3764
10.7748 3.463 -2.3844
7.4014 6.1272 -2.0208
41.3144 21.5644 -13.9359
45.2986 21.0018 -15.2213
54.7473 19.7896 -14.5124
54.3728 19.4265 -15.5796
70.2903 34.8683 -0.9449
83.0715 24.2032 -15.2774
84.5416 27.1967 -18.2621
71.1169 36.7738 -21.8329
90.5454 32.5806 -20.3595
87.7071 40.1352 -19.5086
107.0313 36.1106 -18.3062
111.7858 39.8834 -18.1142
117.5681 46.4958 -13.0232
125.6449 40.3098 -20.273
14.7173 3.8315 9.4023
15.0065 9.7059 6.2811
42.6297 20.4558 4.5802
55.2075 30.9161 -10.554
55.7608 32.8403 -15.5134
61.7742 15.2526 12.2675
94.9416 24.5431 14.8502
107.7342 27.441 14.3985
108.5074 50.8622 -6.1096
123.7408 30.9292 6.5644
123.2913 44.2611 -10.1911
15.3784 2.0439 9.2294
19.8057 -3.2637 5.5994
48.7568 0.7431 3.6935
64.0893 -3.2831 -10.3581
65.4035 -4.7043 -15.6203
120.5918 5.9552 -4.9915
130.6241 18.9088 -10.4917
ID=SMUgigFEMDRCAMNH53849*

LM3=54
-0.706300000003337 -3.13200000000114 -15.0690000000003
-1.93480000000219 -7.77890000000124 -15.6306999999994
37.9701999999984 -2.70290000000064 -22.5968000000002
42.0202999999967 -0.0994000000009897 -23.8855000000001
44.8034999999995 12.5613999999998 -22.0373000000002
53.220799999998 5.97899999999901 -21.5506000000002
52.9041999999982 6.04989999999909 -23.3002000000003
72.6342999999981 0.472699999998968 -2.05500000000011
73.0932999999995 20.7305999999996 -25.5083000000001
79.8955999999999 17.6671999999995 -15.4113000000002
85.8491999999999 14.9549999999994 -18.5484000000002
80.4054999999997 0.530499999999245 -23.3040000000002
95.1083000000003 15.3955999999994 -17.4451000000001
97.8649000000003 7.85129999999927 -15.1806000000001
109.966099999999 22.4821999999991 -10.447
116.6638 20.8720999999991 -9.6704
120.288899999999 35.358999999999 -9.30699999999996
124.237999999999 36.4420999999989 4.99810000000009
126.6204 17.7490999999989 -0.52949999999996
130.8939 28.1981999999989 -6.44569999999987
-2.31290000000381 1.40919999999876 -14.9364999999996
-6.41960000000373 3.77859999999873 -16.2419999999996
31.442199999998 21.9019999999994 -22.9391
35.5472999999982 21.5893999999995 -23.5457
48.694299999999 22.1309999999997 -21.2373000000001
48.737199999999 22.0389999999997 -24.0301000000001
61.4324999999983 38.6915999999994 -1.97490000000001
77.9206999999994 25.9853999999994 -15.6169000000001
81.2341999999994 31.7634999999995 -18.5530000000001
68.370599999999 41.5670999999995 -22.9725000000001
88.3649999999992 36.9386999999993 -17.2823
87.1636999999991 44.8248999999994 -15.1238
105.013399999999 41.9140999999992 -11.1641
109.301699999999 46.4862999999991 -9.67889999999995
115.857299999999 52.033399999999 -0.892599999999884
125.850799999999 46.6642999999989 -6.71039999999988
-0.890900000003445 1.10349999999874 -0.644599999999546
0.325099999996379 8.15999999999881 -5.01249999999962
32.9533999999977 20.5941999999993 -0.929299999999937
47.8819999999985 34.4668999999996 -18.3113
50.1322999999987 34.6679999999997 -23.9249000000001
54.1392999999979 15.7901999999992 9.5683
83.8109999999982 25.363499999999 18.4477
98.7921999999984 29.9600999999989 20.4935000000001
101.860199999999 56.6814999999991 2.66880000000008
112.617899999998 33.6963999999988 16.8252000000001
118.336299999999 51.1918999999989 1.98980000000012
-0.331900000003319 -0.312300000001287 -0.227699999999539
3.19189999999703 -5.91330000000122 -4.32049999999957
38.5643999999981 0.0177999999992029 0.284800000000031
57.9615999999996 -4.26270000000058 -17.7036000000002
62.5153999999998 -3.81270000000059 -22.8912000000002

116.503799999999 8.08589999999889 2.71520000000004
127.049799999999 21.8527999999989 1.72560000000004
ID=SMUgigMALDRCAMNH53850*

LM3=54
8.892 1.7509 -2.4642
8.2278 -1.4989 -2.3338
36.02 0.7395 -11.9141
39.2192 2.8047 -14.5589
42.6641 12.7964 -13.8816
47.7715 6.5626 -13.9241
47.3601 7.0019 -15.1683
64.0291 1.4891 0.7759
61.5705 18.1246 -21.5435
69.5742 17.6 -15.6238
71.6858 13.5961 -18.561
66.6889 0.2933 -18.5094
79.163 13.7397 -19.8516
82.3455 6.4726 -17.9896
93.5796 16.6972 -19.8665
98.5124 15.3789 -19.9627
100.682 27.6873 -22.4931
108.899 30.3676 -11.5745
108.4699 13.1667 -15.6226
108.539 21.5362 -22.576
7.4349 4.3746 -1.5797
5.541 6.7439 -2.0661
30.0452 19.2736 -12.0998
33.004 18.7082 -14.7028
43.2381 20.904 -13.9471
43.5074 20.5525 -15.6506
55.4525 34.8591 0.3109
67.4944 22.4581 -15.4729
68.1434 26.6973 -18.9818
57.28 35.9498 -19.0881
74.5606 30.1342 -20.2185
73.9525 38.072 -18.5926
89.1508 34.5443 -19.6764
92.7814 37.9754 -20.5993
99.5063 44.6023 -17.0832
103.8187 37.4579 -23.2924
12.3657 5.388 8.5825
11.8444 9.0548 6.0909
33.1747 20.554 5.1969
43.2588 28.4193 -10.7401
45.8321 30.2123 -15.3045
47.5265 15.1492 10.4256
79.8356 24.4855 13.9297
95.4359 27.9093 9.5949
93.1844 47.14 -6.906
105.7087 30.9889 1.4287
101.8269 43.9311 -14.7442
12.6463 3.6985 8.344
14.5636 0.2585 6.2504

37.7941 2.4911 5.0484
51.3279 1.0701 -10.071
54.0586 -0.0369 -13.9576
103.203 9.4794 -5.3248
109.7529 15.8084 -13.8082
ID=SMUgigFEMEGUUSNM598576

LM3=54
5.8864 0.1581 -0.7087
8.4485 -1.1341 -1.3038
24.5448 -0.2138 -9.8499
26.8065 1.624 -11.2385
26.4408 9.3399 -11.8871
30.3469 4.9121 -11.3417
30.5834 6.0444 -12.4918
42.4668 -0.3332 2.6365
39.6922 14.8913 -18.4976
42.9568 13.0229 -10.8689
46.4888 12.0158 -13.8825
44.5984 0.517 -17.0582
52.9275 12.0593 -16.5404
58.0316 6.2469 -14.9575
67.1189 15.2464 -16.3176
71.6296 16.5881 -15.39
73.8675 24.2123 -17.8473
77.2856 26.1126 -8.0875
77.9841 12.0379 -13.2237
76.688 19.7823 -17.4943
4.6373 2.5412 -1.3108
5.9817 4.936 -1.9498
18.4836 15.1018 -9.1195
21.9097 15.4402 -11.1942
27.3592 15.3931 -11.5098
27.5863 14.2332 -12.9636
35.4922 27.9732 0.994
41.4152 17.057 -10.457
44.3007 19.7534 -14.7458
36.1536 26.6525 -17.6377
49.4323 23.4679 -16.1772
50.3816 30.9483 -14.8935
62.8839 28.9283 -16.233
67.6179 30.3349 -15.2306
70.2569 36.5382 -13.746
74.5652 29.6969 -17.2078
6.709 2.6666 8.5596
5.4874 7.7273 6.2739
17.6474 13.4063 7.3965
24.8744 21.1686 -6.9004
26.2817 22.6626 -12.951
31.6992 11.211 9.7977
54.4329 19.279 10.4744
65.3115 22.2058 6.4015
62.4907 38.9978 -7.483
71.2882 24.1349 4.2127

72.4256 34.7905 -9.6737
6.9384 2.3227 8.3833
8.9013 -2.3029 6.2401
21.9623 0.9169 7.3561
33.5167 -0.512 -7.1949
34.9872 -1.9379 -12.8724
74.1647 6.9781 -7.5848
79.3922 16.8213 -9.2202
ID=SMUtemUNKZAMBMNH1534**

LM3=54
4.37580000000129 0.817800000001637 -1.35010000000053
6.41990000000111 0.163100000001543 -2.32590000000046
21.612300000001 -1.03339999999885 -8.01860000000032
26.106600000001 1.82370000000105 -9.66390000000031
23.6165000000009 9.469000000001 -10.2577000000002
29.9813000000009 5.65440000000096 -11.0420000000003
28.8711000000009 7.07070000000094 -12.5027000000002
41.6282000000009 0.384900000000975 2.7945999999997
37.7462000000005 14.7974000000007 -17.1364000000001
40.1505000000005 12.3881000000007 -10.6458000000001
43.1890000000006 10.2822000000007 -13.6900000000002
42.7806000000008 0.460600000000826 -16.1075000000002
50.4887000000006 12.0354000000007 -15.2606000000002
56.9570000000008 5.77750000000072 -14.4306000000002
63.1368000000006 15.5185000000006 -15.9595000000002
68.9085000000006 15.6865000000006 -15.3187000000002
68.843718793786 25.0327530260351 -18.0182710694853
75.3815503896339 27.0789858847871 -9.29048884057169
76.5151000000006 12.6634000000007 -12.2966000000002
74.505556076211 20.8242170888549 -18.0191433053456
3.32090000000116 3.59800000000155 -0.775200000000481
3.57210000000106 5.5151000000015 -0.899600000000427
15.2724000000008 16.1468000000011 -7.00450000000025
19.4084000000008 17.889200000001 -9.04840000000023
24.2972000000008 16.972100000001 -10.0297000000002
24.2280000000008 15.6402000000009 -11.3320000000002
32.0372000000006 29.2676000000009 2.64429999999983
39.1943000000005 18.0678000000007 -10.5883000000001
39.6559000000005 20.5450000000007 -13.4095000000001
33.6089000000005 27.3828000000008 -16.3727000000001
47.2038000000004 24.2005000000006 -15.4113000000001
47.4874000000005 33.3851000000007 -14.7650000000001
58.0360000000005 30.0132000000006 -15.3910000000001
63.6744000000006 32.8433000000006 -15.4492000000001
67.3236000000007 40.2466000000007 -13.0683000000001
70.6357962815328 31.3958379282634 -18.2804829612092
1.92500000000121 2.23100000000165 7.89999999999951
3.45590000000126 7.42530000000164 6.38279999999952
15.8474000000009 16.4220000000013 5.30319999999968
22.6865000000006 18.9930000000009 -6.16130000000019
21.3842000000006 21.2350000000009 -10.3216000000002
33.410400000001 12.9241000000011 10.7803999999997
53.5853000000005 20.3986000000008 11.5931999999998

64.3797000000006 23.6815000000008 7.43309999999982
62.3293000000007 39.7626000000008 -5.56310000000016
70.9720000000006 26.1789000000008 4.35149999999982
69.0781000000007 36.8755000000007 -10.7359000000002
2.4636000000012 0.716000000001662 7.89599999999949
6.91410000000118 -2.37809999999841 5.7978999999995
22.378500000001 -0.586199999998716 5.1652999999996
31.5785000000009 -0.393499999999047 -7.84470000000028
31.7085000000009 -2.58039999999902 -10.6092000000003
73.3836000000007 10.1894000000008 -4.01600000000025
77.3517000000006 16.2248000000007 -10.0019000000002
ID=SMUtemUNKTCHBMNH34741*

LM3=54
5.1218 0.0285 -0.2917
7.1165 -1.557 -0.4646
22.2275 0.0427 -7.0522
26.7756 2.4659 -9.1346
25.3318 9.6433 -9.9865
29.6486 5.5387 -10.0517
29.8658 6.262 -11.0639
41.0375 0.4659 3.0432
40.1969 14.257 -16.4735
42.1905 12.5259 -9.0643
45.165 10.2636 -11.7257
42.4089 0.4613 -15.0769
52.2309 11.0833 -14.5251
57.5274 6.0042 -14.4543
65.9554 15.1286 -17.0357
70.2845 15.127 -17.1065
70.4494 25.3675 -19.5485
77.0548 27.8311 -10.4056
77.9264 13.5458 -14.0549
73.6032 21.8874 -19.9128
3.8035 2.2496 0.2604
5.1313 4.9315 -1.1115
17.5549 15.208 -7.1459
21.623 15.8736 -9.0937
26.3589 15.5359 -9.9224
26.59 14.7676 -10.9782
31.9276 26.1112 2.9598
41.4009 17.6602 -9.1315
41.8024 19.6216 -12.1849
34.0294 26.424 -15.4214
47.874 24.8736 -14.5836
49.6452 32.0613 -15.0457
60.3483 28.9581 -17.1754
64.0632 31.8408 -17.849
68.4437 38.8616 -14.8102
70.6447 28.4838 -20.3563
4.4128 3.0848 8.6484
3.7481 7.1546 6.5649
13.7906 11.459 8.5814
24.6277 19.6527 -5.4186

24.6499 21.2456 -9.1878
33.1485 12.9347 11.5185
54.1388 19.1628 13.8103
67.239 24.4387 10.2905
61.1582 41.4025 -8.4667
73.2883 26.6462 6.3868
70.1721 36.8839 -12.0973
4.9698 1.792 8.7032
7.7003 -2.0178 6.7019
21.9278 0.0763 7.6344
32.2755 0.3449 -5.1102
33.3133 -0.8824 -8.8829
75.6198 6.9082 -7.1712
79.5246 17.3496 -10.9845
ID=SMUtemUNKUNKBMNH76137*

LM3=54
-0.00550000000333228 -1.5015000000015 -9.25329999999975
3.06069999999678 -1.8276000000013 -9.28469999999969
17.6246999999977 -0.466300000001005 -12.2925999999997
22.5926999999978 2.51409999999904 -13.8394999999997
22.7320999999978 9.40689999999991 -14.5873999999998
25.961199999998 5.10019999999913 -13.6537999999997
25.842299999998 5.95849999999912 -14.9319999999998
36.9385999999983 -0.983000000000831 0.294600000000283
36.8135999999985 15.3795999999993 -17.7820999999999
39.4189999999987 13.3889999999994 -10.5135999999999
42.1877999999988 12.5608999999994 -13.5541999999999
39.9059999999984 2.00149999999918 -18.0019999999997
49.7040999999989 12.5758999999995 -14.2138999999999
55.7142999999987 7.83909999999935 -13.3166999999998
61.7709999999989 16.9742999999995 -13.3047999999999
67.3530999999989 17.3867999999995 -11.9757999999999
66.9712999999988 27.2842999999995 -14.1382
72.6382999999987 28.6829999999995 -4.2615
73.8579999999988 14.8647999999994 -10.2037999999999
71.8588999999987 22.1814999999994 -14.3653999999999
-1.28430000000337 1.27509999999865 -8.77899999999972
0.602599999996858 3.44909999999874 -9.00239999999976
12.8648999999976 13.9587999999991 -12.5256999999999
17.6020999999977 15.4405999999991 -13.6019999999999
22.0238999999981 15.7802999999992 -13.1845999999999
22.036299999998 14.6153999999992 -14.1998999999999
25.6875999999982 27.2627999999993 0.651499999999954
37.3602999999987 18.0444999999994 -10.3474
39.2275999999987 20.4158999999995 -13.2044
30.3422999999983 25.6560999999993 -17.3958
44.8809999999989 24.7415999999996 -14.1310000000001
45.8810999999987 33.1353999999996 -13.1465000000001
57.0071999999988 31.4468999999996 -13.0105000000001
62.0480999999988 34.7807999999996 -10.9941000000001
64.5523999999987 40.6676999999995 -8.85940000000008
68.2512999999987 33.2180999999995 -13.6849
-0.404300000003416 0.201399999998595 -0.444899999999789

-0.28380000000325 5.22029999999871 -1.39709999999978
10.2937999999973 13.2195999999989 1.12240000000014
20.027399999998 19.1970999999992 -9.25069999999997
20.036099999998 20.6442999999992 -13.9124
23.7921999999979 10.5107999999991 7.0752000000001
44.6791999999984 17.5486999999993 12.3952
59.9693999999985 23.7092999999993 11.6564000000001
56.1328999999985 42.9804999999994 -3.20280000000013
66.0083999999985 26.2920999999993 8.68980000000009
66.6125999999987 37.6064999999995 -6.13360000000004
-0.071800000003258 -0.0110000000013097 0.342900000000284
3.31059999999661 -3.71300000000141 -1.1443999999997
16.8398999999974 -1.75060000000112 0.758300000000265
28.0682999999981 0.0112999999990798 -9.70989999999971
29.7562999999981 -1.04940000000092 -14.4722999999996
70.7003999999985 8.94399999999926 -3.63219999999979
74.3151999999986 19.5366999999994 -7.24249999999988
ID=SMUtemUNKMALBMNH937918

LM3=54
6.40030000000121 0.333400000000765 -0.909300000000273
9.34060000000167 -0.241499999999524 -2.44760000000048
26.932800000001 -0.211799999999798 -10.0671000000002
30.1736000000009 2.53690000000026 -11.3818000000002
28.1096000000008 9.23760000000023 -11.4125000000002
34.4024000000013 5.74800000000041 -11.8853000000002
34.327400000001 6.78160000000039 -13.4441000000002
49.0599000000012 -0.0970999999997784 -0.30510000000027
47.2860000000016 15.3616000000004 -20.5741000000004
48.8327000000012 12.7209000000003 -12.7164000000003
52.4647000000014 11.9451000000004 -16.2371000000003
46.9123000000016 2.30910000000039 -17.8466000000003
60.275200000001 11.7135000000002 -16.8866000000003
63.7235000000013 6.61170000000026 -17.0282000000004
72.9986000000012 14.1390000000002 -17.9222000000004
78.7527000000013 14.8701000000002 -16.1529000000005
78.4544000000013 24.2266000000002 -18.9713000000005
85.1183000000014 25.7722000000002 -8.22920000000058
83.2642000000013 11.7289000000002 -13.0900000000006
85.4313000000014 20.1932000000002 -18.5513000000006
5.31150000000077 3.30810000000032 -0.7641000000002
7.15820000000079 5.17110000000024 -2.23580000000022
21.5176000000007 15.6493000000002 -9.77650000000025
26.2494000000007 15.8862000000002 -11.2583000000002
31.1010000000008 15.8478000000002 -11.6369000000003
31.9783000000008 14.5569000000002 -13.6139000000002
38.8905000000009 26.5051000000002 -0.254400000000317
46.9255000000012 17.7610000000003 -12.3189000000003
49.6905000000012 20.7806000000003 -15.9822000000003
36.918900000001 24.5938000000002 -17.0645000000003
55.2277000000013 25.6484000000003 -17.1337000000004
55.9279000000012 31.1693000000002 -17.0320000000004
67.8421000000012 29.8647000000002 -17.5761000000005
72.9577000000013 31.7338000000002 -16.9267000000005

75.3792000000014 37.1749000000002 -12.7432000000006
80.8101000000015 31.0941000000002 -18.9058000000006
7.91990000000077 2.98120000000015 8.81659999999979
6.84650000000066 6.9588000000001 6.91819999999981
22.6863000000008 16.0616000000002 4.95799999999975
30.5623000000007 20.6457000000002 -7.60180000000027
30.5523000000007 22.6748000000002 -13.1846000000003
38.079800000001 12.6625000000002 8.90759999999972
60.0966000000011 19.1646000000002 8.60009999999961
71.8287000000012 22.1322000000002 6.7221999999995
71.6872000000014 39.2627000000002 -9.82590000000055
77.6116000000013 23.8775000000002 4.60929999999946
79.0163000000014 36.0307000000002 -11.5334000000006
7.48100000000085 2.18030000000017 8.65239999999976
9.41610000000109 -1.61629999999972 7.08019999999972
27.7929000000012 0.327700000000264 5.4724999999997
36.2279000000014 1.27200000000036 -7.88900000000023
37.6304000000015 -0.814299999999626 -12.8017000000002
81.0467000000014 7.36020000000019 -10.2561000000005
85.0351000000014 14.6157000000002 -11.0292000000006
ID=SMUtemUNKUNKMfNB105445

LM3=54
6.3977 0.5852 -0.9548
9.1653 -0.8555 -2.3935
27.4691 -0.7889 -8.9127
31.542 0.5495 -10.9593
31.748 9.7368 -12.8326
36.8697 4.5763 -12.5991
35.9324 5.6751 -13.9236
50.7174 -0.1995 -0.1064
51.2552 15.2574 -20.7705
53.2676 12.9995 -12.1359
56.5246 10.4818 -15.3966
51.1068 -0.3865 -18.4386
64.069 11.2276 -17.528
67.2086 6.0245 -17.2931
78.0103 15.6345 -18.0817
83.4714 15.0866 -17.6924
82.8518 25.0166 -19.8556
90.328 27.2794 -11.3338
88.5464 13.3266 -14.373
90.8998 21.5912 -19.8761
5.8031 2.7908 -1.0316
7.5128 5.7031 -2.444
22.3838 15.9867 -9.2965
26.5523 16.1129 -11.3031
33.6448 15.7085 -12.3636
33.5686 14.7261 -14.0313
42.794 27.7398 -1.1522
51.7921 18.8536 -12.3663
52.78 21.7579 -15.3644
40.9172 27.2371 -18.4441
59.2262 25.66 -17.4131

60.4857 33.045 -16.255
72.4035 30.5324 -18.6421
77.821 32.979 -17.1233
79.5163 38.6774 -13.9442
86.1561 33.4309 -20.4001
7.0679 3.0975 9.4122
5.4026 6.803 6.6241
22.9957 15.105 6.0616
33.3853 20.8483 -8.6938
31.9373 22.8715 -12.6319
39.1478 11.8331 8.9809
63.732 19.2753 11.7219
77.9456 23.6373 8.0209
74.5992 42.489 -7.0807
85.0792 25.8582 4.7886
83.1749 37.9172 -12.1781
8.0914 1.4291 9.3711
8.4306 -2.4243 7.266
27.8547 -0.2165 6.4152
39.4128 0.9025 -7.9609
39.4314 -1.1211 -12.0762
86.8456 6.704 -8.4897
90.8746 17.2443 -11.5839
ID=SMUtemUNKTANMfNB20335*

LM3=54
6.45470000000106 0.264099999999743 -0.426300000000101
9.33990000000078 -0.657300000000377 -1.36080000000006
26.8752000000006 -0.800800000000222 -6.81730000000003
31.5193000000005 2.06969999999977 -8.43949999999998
29.5662000000008 9.67249999999993 -9.55459999999999
33.4020000000006 5.78149999999988 -9.7292
33.4397000000006 6.48689999999987 -10.3933
48.0420000000005 0.719099999999613 2.71510000000004
47.8226000000008 16.6219999999998 -17.3816999999998
49.1425000000006 13.9044999999998 -10.6194999999999
51.0912000000007 11.3376999999997 -12.9348999999999
46.7935000000006 0.971499999999711 -14.8529999999999
58.6569000000007 12.4055999999996 -15.6488999999998
62.5085000000007 6.04039999999952 -15.2604999999998
72.046000000001 14.8482999999995 -16.0956999999997
75.8530000000011 15.3553999999994 -16.3932999999997
77.1222000000015 26.5079999999995 -18.7007999999996
83.3715000000015 28.3262999999995 -9.24879999999959
82.1000000000011 13.1972999999993 -13.2494999999996
83.5193000000016 20.9771999999995 -18.2249999999996
4.91440000000094 2.89689999999964 -0.227800000000088
7.16780000000099 5.40509999999971 -1.46130000000009
20.7057000000011 16.2108 -6.82290000000001
26.5406000000011 17.2072 -8.63169999999999
29.749400000001 15.2084 -9.16829999999997
30.561900000001 14.9849 -10.2906
38.3671000000012 28.3733999999999 3.59250000000009
46.8578000000007 19.6223999999998 -10.6222999999998

47.9802000000009 22.8467999999998 -13.9162999999998
37.4712000000013 28.7035 -14.4363999999999
54.4833000000011 26.9484999999998 -15.6169999999998
53.9791000000016 33.9024999999999 -15.8025999999998
66.3230000000016 31.5739999999997 -17.0737999999997
70.9644000000017 34.2722999999996 -16.0862999999996
74.5217000000019 39.6718999999996 -13.0793999999996
78.9523000000016 33.8328999999995 -18.3646999999996
6.07550000000081 1.71089999999954 8.9373999999999
5.9349000000009 6.66709999999959 6.6490999999999
20.9301000000007 15.1686999999997 7.62019999999998
30.8548000000011 20.6072 -5.45449999999994
28.6472000000012 22.3105000000001 -9.63949999999993
36.4258000000006 12.3189999999997 10.889
60.6658000000007 19.7785999999995 12.3622000000002
74.099200000001 25.1673999999994 8.58460000000028
70.0077000000019 43.1266999999997 -6.87859999999966
81.2176000000013 26.7406999999994 5.26490000000031
77.6171000000018 38.4716999999996 -11.0647999999996
6.66350000000084 1.25379999999956 8.95749999999988
8.22970000000078 -2.39450000000044 6.88809999999989
26.2584000000005 -0.508800000000385 7.48049999999993
36.4879000000003 1.99429999999972 -5.13669999999998
36.3543000000003 -0.349200000000297 -9.06279999999996
82.2712000000011 6.94989999999935 -7.0788999999997
84.9816000000013 17.0984999999994 -11.1200999999996
ID=SMUtemUNKTANMfNB20337*

LM3=54
1.2296999999992 0.282500000000065 -8.06339999999996
3.80340000000054 -1.08819999999919 -8.11180000000044
18.5593999999993 -0.0792000000001968 -11.7361999999998
21.2090999999988 2.10889999999985 -12.5852999999998
19.4420999999993 7.39390000000003 -12.4599
25.1600999999998 4.67090000000005 -11.9166000000001
24.3746999999994 6.13540000000002 -13.0871999999999
37.1028000000001 -0.603799999999776 0.502399999999952
36.4426691786745 14.4299664615818 -17.3739592913898
37.9429999999998 11.3294 -10.9674
40.7414999999999 10.5889000000001 -13.2462000000001
36.9020999999997 2.79060000000011 -16.9511
46.9577999999998 11.1573000000001 -14.1477000000001
50.6137999999997 5.43340000000004 -14.1768
58.8481 14.4745000000001 -14.2180000000001
63.6948999999998 14.6058 -12.9519
63.5404999999998 24.2227 -15.2838000000001
68.3141999999998 25.7416 -6.55720000000007
68.9817 12.5499000000001 -9.69990000000004
67.9050999999999 19.6551000000001 -15.4333000000001
0.513099999999308 1.5017000000008 -7.94079999999992
1.10349999999951 3.71710000000018 -8.16750000000002
13.5601999999995 13.4313 -11.4709
16.5349999999995 13.5621 -12.2065
21.4370999999996 13.9199 -12.2835

22.5219999999995 12.303 -13.459
26.1837999999999 25.0753000000001 0.555599999999933
35.4873999999998 17.8119000000001 -10.6361000000001
36.9445999999997 20.3122000000001 -13.1746
29.3392999999995 23.2932 -16.5273
42.3534999999996 23.9787 -14.1201
40.3988999999996 29.9945 -14.2211
52.9762999999997 28.7794 -14.8962
56.7287999999996 31.5517 -13.2239
59.7532999999997 36.9791000000001 -10.1038000000001
63.8497999999997 31.0445 -14.9812
-0.0441000000004012 0.271200000000213 -0.0524000000000194
-0.838700000000523 4.45200000000014 -1.59349999999999
11.9207999999998 13.6224000000002 1.06259999999993
20.9105999999997 17.5992000000001 -8.76190000000005
20.7957999999996 19.6131 -12.9543
23.5623000000002 9.02950000000024 6.54069999999991
46.703 17.4595000000001 11.4376999999999
57.4763 22.4717000000001 10.6063999999999
54.4559999999997 40.7169000000001 -7.53180000000007
63.4457 24.9776000000001 8.9420999999999
63.4563999999997 36.0077 -7.69470000000008
0.0814999999996699 -0.213799999999776 -0.0743000000000383
1.94339999999985 -3.55849999999965 -1.63270000000011
18.1030000000001 -2.1406999999997 0.951699999999911
27.6344999999997 0.892400000000113 -8.89549999999998
28.5216999999994 -0.816999999999934 -12.8900999999999
67.5267000000001 7.37850000000011 -7.60450000000007
71.1425 16.6939 -8.11150000000006
ID=SMUtemUNKTANMfNB29304*

LM3=54
6.12650000000167 1.00479999999977 -0.701600000000138
8.96830000000157 -0.0734000000002601 -1.69740000000007
28.2947000000012 -0.108700000000146 -9.06060000000001
32.3246000000013 2.01509999999994 -11.1803000000001
30.0965000000015 9.4905 -10.8066000000001
35.9475000000014 4.9593 -11.5185000000001
35.9885000000014 6.22540000000003 -12.6209000000001
51.013100000001 0.24949999999983 0.311199999999864
49.0752129845105 15.4036667095267 -19.5291321316442
51.1586000000013 12.7217 -11.4209000000002
54.6184000000011 11.844 -15.0280000000002
48.1503000000011 0.69079999999991 -18.4589000000001
62.3932000000011 11.1341 -17.4936000000002
65.4300000000009 4.57669999999988 -17.0697000000002
73.831800000001 14.4479999999999 -17.6209000000003
79.627600000001 15.7182999999999 -17.6173000000003
79.9400000000016 25.0285 -20.5767000000004
87.7022000000014 27.6002999999998 -11.7775000000004
85.3678000000008 12.0342999999998 -15.2054000000003
85.8760000000014 20.6160999999999 -21.2423000000004
5.2158000000019 3.76079999999981 -0.901600000000164
7.16400000000184 6.22849999999981 -1.75690000000015

23.5468000000016 17.57 -9.16330000000012
27.9415000000016 16.8504 -11.0271000000002
32.2355000000016 16.3046000000001 -11.0024000000002
33.4851000000016 15.0818000000001 -12.8868000000002
41.0483000000018 27.1355 0.596699999999771
48.8758000000015 18.0605000000001 -10.9402000000002
51.2982000000016 21.2341000000001 -15.2039000000002
40.5326000000019 27.1596000000001 -17.8338000000002
57.2164000000016 25.9050000000001 -17.7824000000003
55.7214000000018 32.6607 -16.7557000000003
69.6137000000016 30.6429999999999 -17.9790000000003
74.2113000000017 32.6363999999999 -17.4780000000004
76.3766000000019 38.6388999999999 -15.1465000000004
82.5956000000018 32.2447 -21.5335000000004
6.84850000000197 2.66689999999978 8.8673999999998
5.48130000000198 7.4475999999998 6.70289999999981
24.1678000000018 16.7293999999999 5.9478999999998
33.4530000000017 20.1891000000001 -6.80430000000018
32.2178000000017 23.2996 -12.4297000000002
39.3363000000015 12.5303999999999 10.2443999999998
65.3568000000013 19.8529999999998 9.80609999999971
76.3804000000012 23.0982999999998 6.70459999999965
72.0183000000019 42.7366999999999 -11.9523000000004
83.8184000000013 25.8102999999998 3.00649999999962
80.5488000000018 36.9986999999999 -13.1208000000004
9.00500000000191 2.28799999999977 9.38779999999982
8.31970000000186 -1.68620000000022 6.76359999999984
29.0383000000015 -0.0929000000001594 6.09679999999988
38.8954000000011 1.9460999999999 -6.91080000000005
39.0091000000011 -0.729300000000108 -13.0724
83.5100000000007 7.15989999999976 -13.3998000000003
87.2575000000009 16.4113999999998 -13.2274000000003
ID=SMUtemUNKTANMfNB37969*

LM3=54
5.8255 -0.0357 -0.6858
8.4715 -1.0359 -2.0012
23.7863 -1.1074 -8.1976
27.457 0.9272 -10.2764
26.9505 8.9276 -11.0933
31.5462 4.7378 -10.4754
31.0114 5.7551 -12.0246
45.6735 -0.3113 1.175
41.9519 14.1032 -17.549
45.3215 12.6231 -11.3005
48.445 10.6157 -15.1864
45.1124 -0.1286 -16.7433
55.3725 11.4108 -16.4892
59.1742 5.5746 -16.8199
68.7132 14.4866 -16.4143
72.8897 15.9361 -15.7759
71.7111 25.3616 -19.5531
78.095 27.1845 -10.2372
77.9403 14.0988 -13.846

78.0788 22.1223 -19.4085
4.7721 1.3842 -0.2047
6.6326 4.5111 -1.4212
17.9676 14.3154 -7.9076
22.2357 14.5733 -9.7128
27.8042 15.1199 -10.5473
27.8325 13.7415 -11.787
35.8446 28.0231 0.9312
42.8785 17.5876 -11.7671
43.8097 20.6255 -14.7297
34.4871 26.0398 -17.2684
49.3557 24.6359 -16.8481
48.2373 31.5637 -16.2436
60.5273 30.1573 -16.7011
65.4899 33.74 -16.0089
68.4882 37.6364 -13.8064
73.4278 31.9631 -19.2862
5.3147 1.5 7.4651
4.8232 5.676 6.1845
18.189 14.1904 5.3971
27.1599 19.3649 -7.1482
24.2465 20.1485 -12.4933
33.3721 11.3842 9.5348
55.9431 18.8837 10.1964
67.7136 23.9825 7.7974
64.9 42.676 -7.4365
71.9169 25.679 5.6067
71.5286 37.3946 -11.875
5.5089 0.4599 7.214
7.6932 -3.3044 6.2432
23.7044 -1.319 5.313
33.6325 0.9357 -6.9412
33.4886 -1.0057 -11.159
78.4335 7.8248 -7.2342
79.9743 16.6796 -11.1827
ID=SMUtemUNKTANMfNB75060*

LM3=54
5.86029999999928 -0.18320000000007 -0.639900000000109
7.91039999999917 -1.55200000000012 -1.60840000000004
26.5432999999997 -2.08949999999993 -8.83420000000011
29.5363999999998 0.320000000000073 -10.6534000000001
29.6009999999994 8.35539999999998 -11.8545000000001
35.0943999999996 3.90900000000003 -11.9688000000001
34.6367999999997 5.68900000000007 -13.3190000000001
48.6845000000005 -1.03179999999971 0.75539999999979
46.3481000000002 14.1215000000002 -20.5596000000001
48.7975000000003 11.5501000000002 -10.8770000000002
51.8401000000006 9.9508000000003 -15.5194000000002
46.5367000000006 -2.09689999999966 -18.4653000000002
59.2996000000012 9.62810000000044 -17.5762000000002
63.4785000000015 5.05170000000055 -18.2185000000003
72.4954000000019 14.5766000000007 -19.1722000000003
77.9565000000023 15.0870000000008 -18.2773000000004

77.8883000000021 25.0848000000007 -21.2783000000004
85.038800000002 27.3042000000006 -11.2430000000004
83.8374000000023 13.7575000000008 -14.8721000000004
83.0024582940981 21.4451595633424 -20.8367395589646
5.11779999999919 2.96089999999983 -0.236500000000061
6.04699999999915 5.40869999999982 -1.27640000000007
20.922699999999 15.7422999999998 -8.74860000000003
24.8047999999991 16.0408999999999 -10.6959
31.5586999999993 15.0317999999999 -11.3098000000001
31.3384999999994 14.3543 -13.0528000000001
38.9464999999997 27.9104 0.918099999999906
46.5711000000002 17.6484000000001 -11.1339000000001
49.0110000000002 21.2083000000001 -16.2267000000001
36.4851999999998 27.4424 -18.4991000000001
54.3393000000007 25.4601000000002 -18.0116000000002
54.1568000000007 32.3259000000002 -17.9084000000002
67.2657000000015 30.4785000000005 -19.6874000000003
72.4557000000018 31.9788000000005 -18.2521000000003
75.8681000000018 37.8889000000005 -14.4859000000004
79.9739000000022 31.9943000000007 -20.6875000000004
6.20769999999919 1.76639999999982 10.2276999999999
6.20709999999907 6.76149999999977 8.37539999999995
23.3174999999991 15.5598999999998 8.20639999999994
30.2244999999993 20.8216999999999 -8.15970000000006
31.0941999999993 24.1266999999999 -13.2090000000001
39.4157999999997 11.696 11.1193999999999
64.1699000000009 19.8948000000003 9.40079999999978
76.4239466957889 23.7441139585566 6.30427754091418
71.6441000000014 43.4526000000004 -9.53740000000029
82.1057000000019 25.5070000000006 2.75819999999964
78.5375000000019 35.5956000000005 -12.5315000000004
5.99919999999923 0.539499999999829 9.82259999999995
8.50419999999932 -3.14930000000012 8.11449999999994
28.2532999999997 -1.49230000000002 7.44809999999987
36.5157 -0.959399999999849 -7.77510000000012
39.1446000000001 -1.6961999999998 -13.5546000000001
83.5225000000022 7.10020000000076 -9.99490000000038
86.2298609343571 17.3061809597542 -12.375981631827
ID=SMUtemUNKTANMfNB75120*

LM3=54
-0.148288847215849 -1.99883426130072 -8.76313981833555
1.14049999999849 -3.50269999999939 -10.5199999999995
22.2204999999999 -2.3711999999998 -16.3601000000003
24.1321999999998 -0.063699999999518 -16.6187000000003
23.8714999999992 7.54450000000005 -16.5151
29.6962999999993 3.5482000000002 -16.614
29.013800000001 4.6834000000007 -17.5502000000004
41.3609999999999 -1.88719999999946 -3.08170000000008
41.7001 14.0141000000004 -21.4806000000002
42.6799999999999 12.0902000000003 -13.9680000000001
47.2545999999997 11.4178000000003 -17.4377000000001
42.9444999999999 0.0301000000003622 -21.2327000000002
53.9663999999998 11.6662000000004 -18.8309

58.065 6.09210000000047 -18.8615
67.1751000000005 16.9867000000007 -17.8526
73.7587000000004 16.5989000000007 -15.6876
73.8502000000007 25.8652000000008 -17.5157000000001
79.3880000000004 28.6345000000008 -8.44220000000002
76.6541000000006 14.1764000000008 -12.9164
79.4535000000007 21.4408000000009 -17.899
-1.48060000000145 0.36520000000049 -9.88089999999978
-0.869800000001443 1.93160000000044 -10.5280999999998
15.564199999999 14.5021000000002 -16.2012
19.166199999999 14.2606 -17.0292
25.6712999999992 14.1723 -16.5373
25.7634999999993 12.8928000000001 -17.7233000000001
29.6968999999993 26.9855000000003 -3.13130000000001
40.2317999999997 17.7123000000003 -13.6207000000001
43.9761999999998 20.8480000000003 -17.3863000000001
32.5510999999996 24.9185000000002 -21.4564000000001
49.8452 23.8161000000004 -19.0113000000001
48.733 31.4792000000004 -18.7791000000001
62.5748000000004 29.2977000000006 -17.5761000000001
67.5117000000005 31.4440000000007 -16.4096
68.6144000000005 37.7177000000008 -13.7171000000001
75.3948000000007 32.8132000000008 -17.5191000000001
-0.0486000000014362 0.00910000000050504 0.0298000000001872
-2.32670000000144 3.40480000000045 -2.29649999999983
11.4531999999988 12.9939000000003 -1.14869999999989
23.2501999999991 19.1330000000001 -13.5984000000001
24.4113999999993 21.8465000000001 -18.6238000000001
27.8266999999992 10.2173000000004 5.13480000000004
51.1723999999997 19.0067000000006 10.2683
67.2974 24.9155000000007 6.66969999999998
63.3403000000002 40.3041000000007 -5.42790000000005
72.2624000000002 26.5192000000008 3.9906
71.1768000000004 37.2430000000008 -10.8835000000001
0.23676285696811 -1.03676637978827 0.131270224981692
1.98193581182198 -5.29068123722701 -0.997479148311865
18.2702999999991 -3.79219999999957 -1.08519999999993
31.5006999999996 -0.245499999999719 -12.7697000000001
33.3600999999998 -1.89889999999967 -17.4980000000002
74.4569000000004 9.98490000000081 -4.16570000000002
79.3795000000006 18.8479000000009 -9.87870000000001
ID=SMUtemUNKTANMfNB75122*

LM3=54
7.4683 0.2541 -1.1716
8.9264 -0.5022 -1.8098
29.5779 -0.4645 -9.9296
32.0583 1.9674 -10.6036
33.0271 10.2267 -13.2336
36.8038 5.3501 -11.206
36.0653 6.2872 -13.3629
50.5286 0.6383 -0.1187
49.2265 14.0537 -20.5055
52.848 12.9881 -13.5191

53.9614 10.5109 -16.3822
46.9744 -0.8595 -17.3735
61.5564 9.8962 -18.6674
66.3039 4.7082 -18.7766
76.2298 12.9753 -19.071
82.6583 12.7777 -17.7149
82.2601 23.3776 -21.0463
90.8514 25.7204 -12.0359
88.7456 11.0918 -14.3748
90.1765 18.781 -21.1897
6.3513 3.2708 -0.7668
7.4349 5.1619 -1.7799
24.6423 16.6892 -9.6735
28.4466 16.683 -11.018
33.7838 16.0256 -11.5164
33.17 14.6191 -13.1449
42.5374 27.0514 0.1831
51.9482 18.0669 -13.3333
50.7087 21.112 -16.1707
38.9592 26.3615 -17.0343
56.9769 24.3406 -18.672
57.6406 31.0709 -18.8193
70.8436 28.919 -20.2962
76.7344 32.2964 -18.5918
80.7813 36.9091 -14.8009
86.0307 30.427 -21.4672
7.891 2.9047 9.2884
7.1513 7.3173 6.9577
22.2254 15.3725 6.2352
33.52 22.009 -7.9871
32.2688 23.7578 -13.2252
41.6924 12.4021 9.8574
63.9095 18.7632 9.8317
77.3226 21.6306 7.6705
75.162 40.2302 -8.8727
85.919 23.8223 4.0246
85.2415 35.0711 -12.4527
7.9488 1.9775 9.2339
9.7258 -2.2863 7.2388
27.0551 -0.4535 6.0927
39.4821 0.8961 -7.6279
39.5447 -1.947 -13.2128
85.2701 5.6299 -8.4404
90.6896 15.1832 -12.2352
ID=SMUtemUNKTANMfNB75124*

LM3=54
4.38059999999987 -0.282800000000487 -0.828999999999706
6.72780000000012 -0.779100000000408 -1.48559999999984
23.9115000000006 -1.00440000000022 -8.6330999999998
26.4243000000007 0.754599999999821 -9.41349999999976
26.540100000001 9.43639999999993 -10.6305999999999
30.3365000000008 5.26199999999988 -10.4814999999998
28.7722000000009 5.7825999999999 -11.6591999999998

44.2244000000005 -0.274000000000141 -0.458399999999738
40.512200000001 13.8622 -17.2608999999998
45.0142000000009 12.6152000000001 -11.6232999999998
49.035300000001 10.5278000000001 -15.7772999999998
40.1684000000008 -0.34980000000002 -15.8864999999997
54.9160000000008 9.55960000000016 -17.3724999999997
57.0354000000006 3.3115000000001 -16.7303999999997
66.1046000000005 12.7906000000002 -19.0629999999997
71.3211000000006 14.0167000000002 -18.4144999999997
73.0073000000006 22.7446000000003 -20.6253999999997
79.0371000000008 23.9304000000003 -11.4637999999997
76.1416000000005 10.4798000000002 -14.8164999999996
77.7716000000005 17.7985000000002 -20.7012999999997
3.48330000000006 3.12259999999953 -0.827999999999818
4.51280000000021 4.71159999999959 -1.63439999999982
18.809700000001 16.8904999999999 -8.47519999999991
22.1052000000011 16.4300999999999 -9.76819999999992
27.3522000000011 14.9067999999999 -10.2901999999999
27.0335000000011 13.9499999999999 -11.3581999999999
35.2846000000011 26.2901 -0.310799999999917
43.773900000001 17.3563000000001 -11.3632999999998
46.0702000000011 20.6356000000001 -15.2280999999999
34.3427000000012 26.0745 -16.2855999999999
50.8012000000011 24.1363000000002 -16.4934999999999
50.0333000000013 30.8209000000003 -16.6662999999999
62.393600000001 27.3412000000003 -18.2859999999998
67.0007000000009 29.1787000000003 -18.0737999999998
69.8839000000009 34.6149000000003 -14.0647999999997
74.7701000000006 28.3507000000003 -20.2757999999997
6.98599999999981 3.15519999999949 8.26120000000013
5.13539999999992 7.45829999999949 6.13840000000014
19.7099000000003 16.5636999999997 4.65590000000015
26.3056000000011 20.2274999999999 -7.27979999999992
26.9202000000013 22.6605999999999 -12.3652999999999
34.6243000000003 11.7296999999997 8.65330000000018
54.9298000000005 17.6682 8.90090000000026
69.6214000000006 21.3160000000001 3.99050000000028
66.140600000001 37.3994000000003 -9.1996999999998
75.5505000000006 23.0182000000002 0.0959000000002881
73.6708000000008 33.5051000000003 -12.8662999999997
8.23259999999981 2.79529999999946 8.56640000000013
8.06249999999978 -1.35850000000054 6.75500000000019
24.4522000000001 -0.509300000000367 4.6063000000002
31.8559000000007 0.0485999999998497 -8.50589999999975
33.5197000000008 -1.48110000000011 -13.0710999999997
74.2527000000004 6.21180000000013 -9.19559999999962
78.5706000000007 13.5054000000002 -12.6343999999997
ID=SMUtemUNKUNKMfNB******

LM3=54
6.10010000000032 -0.0798000000001905 0.0323999999996977
9.49719999999837 -1.14770000000063 -0.871999999999732
27.3284999999984 -1.7893000000003 -7.7410999999999
31.8168999999986 0.573299999999663 -11.0147999999998

29.8667999999991 8.67019999999976 -10.5924999999999
35.5017999999986 4.48839999999967 -10.9194999999998
34.7148999999984 5.42349999999949 -12.4672999999998
49.6667999999996 -0.0640000000000659 1.90119999999993
47.0037999999995 14.7375999999999 -19.5464
49.0174999999997 13.0222999999999 -11.3654
52.6561999999999 11.1259 -15.2935000000001
46.6857999999995 0.480899999999856 -16.9746
59.0258999999994 10.5135999999998 -16.8994
63.2016999999998 5.21009999999985 -16.4452000000001
72.6161999999999 14.2584999999999 -17.1658000000001
76.4073000000001 14.7467999999999 -17.2880000000002
77.2637000000004 23.874 -20.2293000000003
84.3467000000004 26.0229 -10.2511000000003
82.4545000000003 11.9022999999999 -12.8703000000003
83.5711000000004 20.7901 -19.8164000000003
5.88900000000033 2.53749999999985 -0.0285000000002563
7.57090000000002 5.26869999999986 -0.866100000000192
21.4122999999994 15.9369999999999 -7.88309999999999
25.9066999999994 16.7566999999998 -10.6533
31.4849999999994 15.3968999999999 -10.9786
32.0786999999994 14.1535999999999 -12.4590999999999
36.9205999999999 26.4915 2.04829999999993
46.9027999999997 17.8715 -11.6277
49.2435999999996 22.1658999999999 -15.274
37.9476999999997 26.1982999999999 -16.4783
55.0623999999997 25.2731 -16.9323
54.1798999999999 30.9702 -17.3512000000001
67.2598000000001 29.4428 -17.1044000000002
70.8831000000003 30.9839000000001 -17.3203000000002
73.7554000000003 37.1175000000001 -14.9823000000002
79.4295000000005 29.6020000000001 -20.4342000000003
5.04110000000013 1.95649999999991 8.44509999999982
4.81580000000035 6.39549999999997 7.39139999999978
21.3666999999998 15.0314999999999 8.13259999999992
30.0964999999996 20.6715999999999 -8.45709999999998
30.5497999999996 22.1432 -13.3725
38.6698999999997 12.4134999999999 12.0188
62.5636000000001 20.9285 11.0601999999999
74.6736000000003 23.2925 7.1552999999998
72.8248000000004 39.8528000000001 -9.34840000000022
82.7163000000003 25.8431 0.580699999999771
79.3588000000003 33.8254 -12.1194000000002
5.21520000000008 0.912999999999874 8.48189999999982
7.18561414704719 -3.17823552724912 6.90702878456903
26.488799999999 -0.197700000000267 8.31470000000005
36.9640999999988 0.930699999999702 -7.50019999999988
37.8391999999988 -1.19230000000034 -13.4565999999999
78.8074000000002 5.18159999999991 -6.71430000000023
83.6059000000004 17.1290999999999 -11.4524000000003
ID=SMUtemFEMSAFAMNH168954

LM3=54
6.72479999999853 0.307499999999416 -0.831699999999804

9.24930000000118 -1.07130000000015 -2.17070000000031
27.6811999999997 -1.24280000000017 -9.12940000000001
31.3836999999995 1.1665999999997 -11.0870999999999
30.7452000000002 9.02410000000004 -12.0589000000001
35.4148000000002 5.09789999999994 -11.5410000000001
34.8398000000006 5.83240000000011 -12.9741000000002
52.1482999999999 -0.727100000000079 1.34949999999999
47.5773 14.2595 -20.0039000000001
51.5995999999999 12.4583 -11.3777
54.2947999999995 10.6880999999999 -14.7721
49.1214999999995 -1.10960000000024 -17.9008999999999
62.2010000000004 10.6205 -16.8715000000001
64.8571000000003 4.74399999999998 -15.9609000000001
74.2012000000009 13.7533 -17.6399000000002
79.0493000000008 13.2102999999999 -18.2100000000002
79.880000000001 22.7588 -20.5345000000002
86.251800000001 23.8508 -12.2437000000002
84.6821000000011 9.49629999999997 -15.7577000000002
87.8119000000011 17.7233 -20.4524000000002
5.82129999999937 3.13989999999955 -0.517700000000018
7.63869999999981 5.82569999999966 -1.98110000000009
23.2507000000002 15.53 -8.62560000000014
27.9519000000002 15.7874 -10.9723000000001
32.6046000000002 14.8140000000001 -11.6898000000001
32.5400000000002 14.0526000000001 -13.0363000000001
43.1403000000003 27.7823 0.818699999999842
49.0245999999999 17.1439 -11.7187
51.1468 20.3768 -14.5648000000001
41.2911000000002 27.8897 -17.2793000000001
58.0054000000005 24.1326000000001 -16.8847000000002
57.8999000000005 31.5256000000001 -15.6399000000002
69.3081000000007 27.9551000000001 -18.5453000000002
75.035400000001 30.7762000000001 -18.2711000000003
78.014100000001 36.7547 -15.5663000000003
84.190200000001 30.6957 -20.9937000000002
7.43400000000011 2.81809999999964 9.19399999999979
5.79180000000004 5.96639999999963 7.23039999999979
23.6075000000003 15.7944999999999 6.37519999999984
33.3016000000002 20.213 -8.06890000000011
29.9841000000003 22.1041 -13.3236000000001
40.8773000000002 12.1716 10.0370999999999
63.9194000000004 18.0767 9.44989999999983
76.2154000000008 21.2731 5.76119999999977
74.8605000000009 38.106 -7.25380000000027
82.480700000001 23.008 2.99709999999975
82.7391000000009 34.193 -14.4118000000003
7.19170000000014 1.74939999999965 9.32959999999977
8.36940000000027 -2.10730000000034 7.4673999999998
28.7772000000002 -1.34100000000014 6.34539999999987
38.4664999999998 1.44499999999981 -7.86419999999995
36.8891999999997 -2.26020000000024 -12.9337999999999
81.5442000000009 5.93849999999996 -8.02960000000019
87.5600000000011 13.7955 -13.7479000000002
ID=SMUtemFEMZOOUSNM314972

LM3=54
3.22729999999828 -3.36600000000104 -9.59669999999954
4.21380000000734 -0.123499999999462 -8.47130000000001
51.6095999999987 -3.39340000000062 -4.68049999999989
56.3047000000013 -2.51920000000004 -4.57339999999985
46.8077000000006 6.96419999999993 -4.21999999999999
64.6653000000021 4.59839999999992 -3.20330000000007
67.8323000000036 5.3941000000004 -4.41410000000009
70.9853000000011 -0.296900000000054 12.8183
87.7945000000014 14.6598000000003 -9.68920000000008
80.0100000000031 7.63700000000021 -0.971800000000102
84.4686000000018 5.0050000000002 -4.69330000000015
92.3655000000013 -4.27459999999986 -3.46780000000011
93.0453000000029 4.66590000000031 -7.72400000000015
104.268500000002 0.379700000000103 -5.8285000000001
109.670800000002 8.69720000000019 -7.2028000000001
116.207200000001 11.5292000000001 -7.22900000000012
114.161700000001 18.9826000000002 -9.93040000000011
120.654600000001 20.3115000000001 0.471699999999929
117.831500000001 8.61200000000009 -5.01160000000007
118.004500000001 13.5747000000001 -10.8671000000001
2.41560000000328 5.05459999999984 -9.21069999999983
4.51120000000452 1.64560000000007 -8.49199999999996
47.7323000000015 17.7338 -3.34289999999996
52.9832000000018 18.4012 -3.5029
62.5363000000024 16.0139000000002 -3.03830000000005
65.2930000000027 15.6982000000003 -3.92340000000006
66.623900000002 21.3739000000001 13.9422
78.4666000000023 17.6465000000002 -0.864000000000093
81.662400000002 21.7406000000003 -3.98690000000007
85.8924000000019 33.2366000000003 -1.62570000000008
89.1699000000015 25.3020000000002 -7.05910000000007
98.7347000000015 32.6041000000003 -5.01170000000008
106.977800000001 26.8755000000002 -6.0263000000001
113.910600000001 25.8362000000002 -7.1420000000001
114.514900000001 29.3289000000002 -4.61510000000007
116.198500000001 25.9050000000002 -10.6261000000001
-1.11759999999838 0.133099999999499 -0.266799999999801
0.462200000002272 3.49599999999965 0.122200000000169
37.9973000000008 10.4970999999998 12.0150000000002
59.0825000000022 21.1938000000002 1.14229999999999
53.3094000000021 21.3959000000002 -5.13350000000002
37.4231000000005 5.29959999999975 12.9675000000002
71.7093000000016 10.7905000000001 19.8345
105.112000000001 16.2231000000001 18.1742999999999
106.849100000001 33.5838000000002 4.10419999999992
117.081000000001 18.0121000000001 12.4517999999999
115.087300000001 27.8572000000002 -2.3648000000001
-1.36769999999863 -0.401200000000523 -0.93919999999976
1.1562000000014 -3.38430000000053 -0.284399999999798
38.9426 0.814399999999651 11.1487000000002
60.2242000000011 -3.57520000000005 -0.908799999999989
56.9189000000008 -5.04570000000009 -6.44709999999992

111.585400000001 0.942200000000068 3.29479999999994
117.046900000001 9.35050000000013 -2.77110000000008
ID=TAMmexFEMPANBMNH392642

LM3=54
4.45429999999754 -3.53440000000098 -8.47359999999997
5.97850000000094 -1.33600000000017 -7.32520000000064
48.4473999999966 -2.39430000000079 -3.14809999999999
56.7255999999974 -0.862200000000703 -4.50859999999999
44.9866999999975 7.22929999999946 -2.65630000000009
63.2788999999987 3.34989999999957 -3.3806999999999
64.7287999999996 4.46059999999971 -4.42999999999998
70.6316999999981 -1.22450000000046 12.3926
85.9714999999993 14.0367999999999 -9.31129999999989
77.7332999999975 7.48069999999954 1.34310000000019
80.9610999999989 5.23969999999971 -2.19699999999981
90.4493999999979 -5.25910000000042 -4.39499999999984
90.1954999999981 5.33199999999968 -8.58649999999987
104.102399999998 -0.573700000000413 -6.07859999999977
109.898399999998 8.15839999999959 -8.75529999999973
113.985099999998 10.4116999999995 -8.45829999999974
113.706599999997 17.8171999999995 -11.9452999999997
120.696599999997 18.1241999999995 -1.50169999999972
116.509799999997 7.61159999999948 -7.12729999999972
118.916999999997 12.2799999999995 -12.1717999999997
3.56539999999955 4.63719999999968 -7.98020000000033
5.2012000000003 2.60199999999981 -7.38660000000042
46.8168999999983 16.5873999999996 -2.83410000000005
54.3634999999986 18.1087999999996 -4.02560000000002
60.6960999999987 16.0822999999997 -3.50119999999999
62.4207999999988 13.5487999999997 -3.69319999999998
64.3860999999981 20.4303999999996 12.2837
75.8341999999983 17.5047999999997 0.817200000000085
78.7756999999986 20.7382999999997 -3.2952999999999
85.2196999999983 32.5182999999997 -3.98079999999987
88.2487999999985 23.0943999999998 -8.80919999999986
99.7986999999976 31.7066999999996 -6.03149999999979
107.518199999997 25.6133999999996 -8.47129999999974
111.727499999997 23.8869999999996 -8.17759999999974
114.645099999997 27.1574999999996 -6.72839999999972
117.067599999997 24.0710999999995 -11.9068999999997
-2.42510000000127 0.29469999999943 -0.566800000000248
0.0322999999992389 3.32789999999958 0.802099999999704
37.8804999999985 9.82869999999957 11.1223999999999
58.4026999999985 19.8290999999996 1.09119999999999
54.9528999999987 22.9612999999996 -6.88460000000002
40.5127999999983 5.81009999999953 13.8871999999999
83.2065999999976 11.9427999999995 20.8171000000001
104.303799999997 14.4311999999995 17.0323000000002
110.638499999997 30.6314999999996 1.89650000000023
115.495899999997 16.5141999999995 13.6355000000002
114.283399999997 24.8951999999995 -3.88049999999973
-2.40330000000135 -1.1968000000006 -0.651900000000242
0.850299999998856 -3.42110000000055 0.456599999999726

38.9754999999982 0.784699999999489 10.7446999999999
60.2881999999992 -2.98730000000033 1.10650000000001
59.5119999999991 -4.28710000000033 -7.67010000000008
113.655999999998 1.12429999999948 1.83010000000025
116.591099999997 8.4487999999995 -3.36449999999972
ID=TAMmexFEMBHOBMNH952628

LM3=54
4.38559999999521 -3.74290000000058 -9.43079999999955
5.55280000000536 -0.355899999999314 -8.93260000000018
55.4056999999974 -2.90400000000047 -4.95689999999975
58.9103000000006 -1.60809999999993 -5.60629999999981
52.4020999999996 8.5140000000001 -5.04519999999998
68.5822000000017 4.90939999999998 -4.63869999999994
72.9541000000033 6.13570000000046 -5.37379999999998
77.0577000000004 -0.976900000000002 15.0997000000002
95.2182000000009 15.0876000000002 -11.6844999999999
85.7284000000029 9.6858000000002 -0.928099999999956
89.4729000000014 4.7065000000002 -3.76969999999997
100.846400000001 -6.69639999999987 -4.01279999999995
102.391800000003 4.44580000000027 -9.4054
114.855500000001 -1.16199999999998 -5.43339999999994
121.364200000001 7.52160000000004 -7.20909999999995
125.788 10.9452 -6.84619999999994
126.4293 18.502 -9.47319999999994
131.7022 18.4754 -0.221799999999926
128.9892 7.09439999999995 -4.40709999999992
129.516 12.5134999999999 -10.3416999999999
6.37200000000097 4.76480000000006 -8.54099999999989
8.7046000000024 1.5827000000003 -8.7448
55.1462000000004 18.7301000000001 -4.25989999999992
59.703300000001 19.6719000000002 -5.30859999999992
69.9255000000018 17.3037000000003 -4.90419999999995
74.4762000000022 16.5660000000003 -5.03369999999996
71.7980000000012 23.7965000000002 14.0779000000001
87.4136000000018 17.3797000000002 -0.818099999999953
90.2843000000015 22.4140000000002 -4.46579999999994
94.862500000001 35.0153000000002 -3.07019999999995
100.479600000001 24.6686000000001 -8.58039999999994
109.675800000001 33.0794000000001 -6.57919999999994
118.6709 26.8155000000001 -6.91499999999994
123.8299 25.0547000000001 -6.57549999999995
124.7165 29.7511 -3.12519999999993
128.4348 25.4244 -10.0664999999999
-0.765700000000984 0.524099999999778 0.0335000000001834
1.08749999999986 3.65389999999993 0.572800000000162
41.2691999999995 12.4302 12.9101000000002
63.5528000000014 22.4258000000002 0.389500000000077
60.6094000000013 23.5597000000002 -7.34379999999993
40.2514999999992 6.16879999999992 14.3113000000002
92.3289000000008 13.7181000000001 24.6448000000001
113.8933 16.6349 20.7826000000001
117.9674 34.8245000000001 2.45980000000006
126.4374 18.0483999999999 13.6681000000001

123.0848 27.698 -1.35649999999992
-0.750300000001128 -0.809500000000253 -0.10789999999977
1.95379999999886 -3.37150000000025 0.574100000000199
42.5794999999986 1.35259999999985 12.9197000000003
66.2922000000005 -2.14999999999996 0.557300000000126
62.2511 -4.49189999999997 -7.47269999999982
123.1791 1.58829999999997 3.21490000000009
125.224 9.74639999999998 -1.68369999999993
ID=TAMmexFEMPANBMNH3313**

LM3=54
4.17010000000123 -4.10999999999968 -9.58910000000011
5.44749999999843 -1.34090000000043 -8.54289999999967
55.6167000000006 -3.94289999999959 -3.90589999999994
61.8503999999998 -2.40209999999999 -5.06369999999994
48.5165000000004 7.05380000000013 -4.15309999999989
67.3986999999998 2.56470000000005 -4.44759999999993
70.0669000000004 3.59460000000009 -4.57379999999985
74.4315999999996 -2.18040000000002 14.7799000000001
92.9803999999996 13.9246 -10.7840999999998
81.2674 4.89119999999993 -0.47279999999985
86.1241000000004 3.43150000000003 -4.14139999999983
95.4782000000001 -5.59319999999993 -4.74819999999988
96.4335999999999 4.57919999999998 -8.03579999999983
109.1776 -0.278899999999943 -5.62899999999985
115.7052 8.30840000000008 -8.55969999999982
121.254699999999 12.4925000000001 -9.71809999999981
120.441299999999 18.3063 -11.4263999999998
125.851099999999 19.3071 -1.60029999999978
122.890299999999 8.10630000000006 -5.4022999999998
126.876699999999 12.9039000000001 -11.7466999999998
2.97909999999978 3.86859999999997 -9.99499999999986
4.76519999999907 1.33409999999979 -9.03389999999981
51.6154999999998 18.3193000000001 -3.96299999999987
58.4382999999998 20.5207000000001 -4.91649999999986
64.7880999999995 16.7484 -4.29879999999984
66.9928999999997 16.6549000000001 -4.47769999999985
67.7416999999993 21.2025 14.2429000000002
81.1923999999995 15.8455 0.965100000000181
83.5448999999997 21.7243000000001 -3.19369999999983
89.6561999999994 33.5633 -4.0470999999998
94.1061999999995 24.5114 -8.02009999999981
104.488399999999 33.0134 -5.87479999999978
112.627799999999 27.1164000000001 -7.62109999999979
119.306799999999 25.0663 -7.95049999999978
120.139699999999 28.513 -5.20429999999977
124.859799999999 25.0734000000001 -11.5125999999998
-1.07139999999984 0.377300000000046 -0.50829999999996
-0.444500000000112 3.95560000000002 -0.403999999999929
40.5495999999998 10.5718000000001 12.8964000000001
62.5983999999995 20.6671000000001 2.25210000000015
59.8408999999998 23.4674000000001 -7.82899999999985
39.1957999999998 5.08220000000008 14.6538000000001
92.1926999999994 12.5073 23.1770000000002

110.637599999999 14.8904 18.4584000000002
114.996691676278 33.0151756671261 2.79703411126836
120.281399999999 17.2035 15.0512000000002
121.2749 26.9270000000001 -3.22249999999979
-1.4293999999998 -0.820899999999954 -0.246999999999953
0.33040000000005 -3.95579999999999 -0.361899999999933
41.2541999999999 0.636700000000118 12.4091000000001
65.1119999999997 -2.83290000000001 3.02650000000015
63.3195000000006 -4.64869999999985 -7.86989999999997
118.5726 1.52220000000007 1.34150000000018
122.737699999999 9.26690000000005 -3.77559999999979
ID=TAMmexUNKMEXBMNH79161*

LM3=54
2.8858000000002 -4.41079999999969 -9.41100000000033
4.43789999999915 -1.38330000000003 -8.52999999999999
54.2656999999997 -3.61089999999999 -4.68690000000008
60.406699999999 -1.91810000000004 -5.88649999999992
51.5623999999983 7.11739999999999 -3.68840000000007
67.7121999999964 2.48239999999953 -4.52369999999976
68.9388999999976 3.75879999999975 -5.54699999999993
74.0106999999985 -0.637100000000129 14.0294
91.876899999998 12.3711999999997 -9.61159999999985
83.5541000000003 5.88680000000013 0.0739000000000178
86.6381000000002 2.76200000000011 -2.89449999999992
96.3623999999991 -6.92060000000011 -3.33229999999992
96.995799999999 3.88649999999985 -8.05019999999988
110.116 -1.30160000000007 -4.58079999999992
117.5104 8.17709999999995 -6.54619999999995
120.3323 10.9973999999999 -6.24559999999994
120.437975009064 17.4879815426034 -8.74965286059305
125.529311151774 18.3618692316705 0.155653640507196
124.124 7.88629999999993 -3.10839999999994
124.3526 12.3111999999999 -8.72109999999993
2.37149999999898 3.10359999999993 -9.34220000000004
4.79889999999879 0.163499999999931 -8.59390000000002
50.3390999999976 15.6570999999998 -4.8568
57.6809999999973 17.3193999999998 -5.95529999999991
65.5771999999972 14.1699999999998 -4.55779999999988
67.8974999999972 13.0596999999998 -5.34129999999986
70.1540999999977 20.8876999999998 13.9176000000001
81.2123869365005 16.4045310417778 -0.65828589461722
83.8005999999982 19.5498999999999 -5.07939999999989
89.8197999999981 32.5044999999998 -3.6715999999999
93.5936999999984 22.3254999999998 -8.37499999999989
104.923599999999 31.1395999999999 -5.24549999999991
114.474499999999 25.0348999999999 -5.80919999999995
120.183599999999 22.6705999999999 -6.01389999999994
121.929599999999 27.3015999999998 -2.95289999999993
124.231699999999 25.3672999999999 -8.5553999999999
-1.06350000000039 0.100400000000021 0.0277999999999076
1.2102999999992 4.05449999999999 -0.303200000000079
40.8617999999985 12.5578 10.1973
65.6844999999974 18.9136999999998 -0.364399999999919

60.1712999999974 23.7592999999998 -8.62879999999991
45.4748999999986 6.10839999999998 14.2551999999999
91.355399999999 13.1388999999999 20.9462
110.473099999999 15.6777999999999 18.1588
114.939099999999 31.3770999999999 3.86780000000008
123.4117 17.4106999999999 13.5441
120.5291 25.8192999999999 -2.22019999999993
0.134799999999711 -0.388099999999956 -0.188700000000107
2.42489999999991 -3.4412 -0.118900000000114
43.2397999999989 -1.00160000000002 10.1703999999999
69.2514999999982 -0.505100000000132 -0.0990999999999818
64.4983999999981 -6.05670000000021 -7.77909999999992
119.1325 1.71579999999992 4.65550000000005
123.0581 9.07359999999994 -1.40449999999995
ID=TAMmexFEMMEXBMNH121826

LM3=54
2.96249999999806 -5.23730000000032 -10.5951999999996
3.33029999999837 -1.90470000000019 -10.9077999999996
55.1715999999981 -5.50170000000036 -4.65039999999995
64.1819999999994 -2.71360000000011 -7.18299999999995
49.738699999999 7.5752999999998 -4.66700000000003
70.4761999999999 2.21989999999996 -5.24639999999992
76.054299999998 4.72479999999967 -5.69509999999987
79.9651999999987 -1.30850000000028 12.4615000000001
97.6264999999973 15.5397999999995 -10.6234999999998
85.1441000000006 7.00529999999989 -1.46639999999987
89.2608 3.09949999999981 -5.45349999999991
102.078499999999 -7.29170000000036 -4.8907999999999
101.904699999998 4.54899999999951 -9.95669999999985
116.738199999999 -0.539800000000413 -8.39789999999988
125.467799999998 10.6013999999995 -8.65879999999983
130.337699999999 12.7945999999996 -6.91989999999985
129.684999999998 21.1359999999996 -9.87339999999984
136.026799999999 22.2189999999996 0.561000000000144
132.158499999999 9.00559999999958 -4.02609999999984
137.198999999999 16.4565999999996 -8.54129999999983
0.885499999998105 3.88289999999987 -10.9812999999999
2.72139999999812 1.33859999999989 -11.2002999999999
51.5994999999986 22.2826999999998 -5.45219999999993
59.8588999999987 22.2616999999998 -7.12039999999993
67.4924999999984 19.6299999999997 -5.84729999999988
72.8807999999983 19.4575999999997 -5.93089999999988
72.9114999999983 25.9661999999997 11.3204000000001
82.9415999999985 19.4393999999997 -1.35749999999985
85.307899999998 24.2828999999996 -5.52519999999984
94.7381999999979 38.7534999999996 -4.30489999999983
97.658299999998 27.4939999999996 -10.0965999999998
110.497999999998 37.9121999999996 -7.21519999999982
122.745499999998 29.8291999999995 -8.06169999999982
127.845899999998 28.6805999999996 -6.91699999999982
128.503799999998 33.9105999999995 -3.85349999999982
135.757799999998 28.5605999999995 -8.39629999999982
-2.13858290288762 0.516548840789578 -0.960562574730608

-0.619500000002041 4.07569999999983 -1.43339999999993
40.1501999999984 11.8130999999998 11.4434
65.8125999999984 25.3825999999998 -0.873799999999889
60.3204999999984 24.9515999999998 -9.8300999999999
40.6702999999985 6.69849999999977 13.7719
99.9336999999985 16.7988999999997 21.9627000000001
112.700099999998 18.5090999999996 19.6949000000001
120.302499999998 39.3490999999995 2.35130000000019
129.952499999999 22.0657999999996 15.2161000000001
129.038899999998 31.5859999999995 -0.523399999999823
-1.89831990144524 -0.968796975286978 -0.8556193096986
0.736799999997883 -3.82200000000015 -0.645599999999982
41.0322999999984 1.58819999999976 11.4764
70.7315999999993 -2.65680000000019 -0.884099999999953
65.136999999998 -6.20730000000028 -10.3647999999999
128.107599999999 2.58419999999955 3.97360000000014
132.648199999999 12.7623999999996 -0.276399999999851
ID=TAMmexUNKUNKMfNB102646

LM3=54
2.8645 -3.671 -9.8463
3.9471 -1.4122 -9.3682
55.1941 -4.2039 -5.9036
60.991 -2.1761 -6.9238
49.9908 7.6009 -5.0597
67.6713 3.0491 -5.9423
69.3124 3.7623 -6.2148
74.7209 -1.1433 12.1679
89.4585 14.0595 -11.2004
81.7503 5.8563 -0.142
85.5945 2.2076 -4.3783
93.6827 -6.0366 -4.1664
92.6625 3.646 -9.4177
106.6898 -0.4734 -5.6776
114.2289 9.0302 -7.3195
117.7038 11.1431 -7.9677
118.0091 19.0591 -10.8038
125.2445 20.4692 -0.8561
121.4549 7.8596 -4.6435
125.3917 14.2736 -10.7464
2.2261 4.4749 -9.5871
3.4876 2.3599 -9.9296
50.46 20.2324 -4.7667
57.457 20.0705 -5.8807
64.9925 17.0646 -5.4636
66.5026 16.9035 -5.8097
67.7551 23.4428 12.2857
79.0415 20.6451 -0.7584
81.058 25.1317 -4.8849
87.2651 34.9204 -4.2043
89.0237 26.0412 -9.3949
101.1744 34.2591 -5.2894
111.4686 27.3607 -6.8987
115.1748 26.5005 -8.015

117.7299 30.5985 -4.7778
123.0969 26.1376 -10.6202
-2.3552 0.4506 -0.8713
-0.2537 3.6025 -1.0009
38.8453 10.9327 10.8337
61.8547 22.3361 -0.0726
58.1414 24.1007 -7.4226
41.1605 6.1849 12.7134
86.2459 13.8745 21.2994
110.3996 17.3974 17.6988
113.405 33.5998 7.2311
120.818 19.5417 13.6237
118.3289 28.2908 -2.0195
-1.998 -1.4688 -0.9477
0.5007 -4.1844 -1.1951
40.0876 0.4926 9.7117
65.8115 -2.2731 -0.851
63.2276 -5.8037 -8.6467
117.9094 4.3151 7.3734
122.0056 11.6952 -1.093
ID=TAMmexUNKGUAMfNB102650

LM3=54
4.00909999999264 -3.33750000000167 -8.89199999999929
5.71369999999334 -0.761000000001557 -8.613299999999
49.4749999999953 -3.2038000000011 -3.16849999999939
56.9944999999956 -1.44960000000101 -4.04809999999942
47.8095999999954 7.93109999999892 -2.18099999999943
64.9236999999963 3.12599999999912 -2.55529999999942
68.0485999999964 4.71539999999914 -2.33849999999941
72.6519999999962 -1.05380000000098 13.6198000000007
84.3038999999981 13.7625999999994 -7.95989999999938
77.6245999999973 6.26049999999928 1.1379000000006
82.7719999999977 4.83979999999935 -3.85439999999938
89.5857999999978 -6.09130000000069 -3.51299999999929
90.7086999999984 4.98239999999946 -7.33509999999937
103.851199999998 -0.559300000000628 -5.32769999999922
110.634999999999 8.84639999999953 -7.04509999999919
114.018699999999 10.6914999999996 -6.02489999999915
115.559099999999 18.4286999999996 -9.31199999999916
120.275299999999 19.4842999999995 -2.1272999999991
116.664599999999 6.82459999999948 -3.33269999999911
121.590199999999 12.8682999999995 -8.27519999999908
2.19199999999309 5.08779999999852 -8.6768999999991
4.47409999999292 3.25779999999852 -8.33569999999915
45.9601999999955 19.476899999999 -3.3925999999994
53.664499999996 19.8965999999991 -3.98999999999943
62.7233999999966 17.2238999999992 -2.35209999999945
65.8421999999967 16.8801999999992 -2.51369999999944
68.3391999999963 23.6053999999991 13.0022000000006
74.9473999999976 19.0708999999993 1.80840000000059
80.0545999999978 22.7181999999994 -3.4638999999994
82.9578999999979 33.9768999999994 -3.14119999999932
87.0709999999983 24.2003999999995 -7.10689999999934

97.9858999999985 33.9132999999995 -5.26209999999924
108.364299999999 26.3258999999997 -6.38819999999924
112.167799999999 25.4064999999997 -6.38709999999919
113.230799999999 30.1941999999996 -4.00089999999915
119.721599999999 25.9011999999996 -8.3470999999991
-0.183200000007437 0.514099999998394 -0.0937999999990312
0.640299999992695 4.16929999999843 -0.0961999999990686
37.7253999999948 12.9564999999988 11.9470000000006
60.0829999999964 21.5568999999991 0.957000000000547
53.9935999999961 23.2656999999991 -7.21819999999943
42.4223999999948 7.38909999999875 14.4525000000006
86.545599999997 14.2004999999992 22.5706000000007
107.214699999998 17.3097999999993 18.5461000000009
108.799099999998 33.7910999999995 3.09480000000086
118.885999999998 19.3577999999994 12.7386000000009
115.861699999999 27.6565999999996 -0.85389999999916
0.0603999999924598 -0.331500000001615 0.0536000000009412
1.38019999999271 -3.47690000000159 0.656900000000928
39.2859999999947 0.199099999998728 12.0455000000007
63.549499999996 -1.76520000000095 1.3423000000006
58.9566999999958 -4.58480000000096 -7.22189999999939
113.893299999999 2.51549999999939 2.85690000000088
117.652099999999 10.9262999999995 -1.19029999999911
ID=TAMmexUNKUNKMfNB105446

LM3=54
3.70020000000096 -2.64540000000019 -8.11159999999982
5.01330000000296 -0.658699999999728 -7.67359999999953
49.9653000000012 -3.29079999999994 -4.04609999999982
58.3623000000019 -1.20139999999983 -5.12220000000001
46.3743000000008 7.27730000000014 -4.41199999999984
65.4340000000012 3.9915000000002 -4.25900000000005
67.9538000000011 4.98170000000024 -4.73880000000015
70.4829000000004 -1.92620000000002 13.5406
87.8478000000003 14.8263999999999 -10.7062
78.3016000000013 5.54050000000008 0.350899999999883
81.8208000000006 3.31079999999998 -3.11580000000008
90.9279000000001 -7.01940000000014 -2.50460000000002
87.9807000000007 3.81469999999993 -6.88750000000003
102.3431 -0.882300000000188 -4.80950000000002
108.648399999999 9.32719999999976 -7.9034
111.3562 11.2281999999998 -7.78950000000004
111.1148 19.1840999999998 -10.3425
119.942599999999 20.9242999999997 -1.58300000000002
114.429599999999 8.42969999999974 -5.4401
118.422099999999 14.9676999999997 -11.2704
3.25260000000335 5.23320000000029 -8.18279999999993
4.43050000000349 3.06490000000031 -7.76759999999993
45.5804000000014 18.7444000000002 -3.33079999999995
55.3169000000012 19.2342000000002 -4.78959999999999
62.824900000001 17.8325000000002 -4.83260000000003
63.759200000001 16.5041000000002 -4.94320000000006
67.6918000000009 24.6317000000001 13.639
75.6575000000006 19.295 -0.365700000000052

77.5142000000006 22.8789 -3.63420000000003
82.4001000000005 35.7042999999999 -2.52530000000001
83.6521000000003 24.4543999999999 -6.98210000000001
103.2597 33.8332999999999 -7.24760000000001
104.9887 27.1307999999998 -7.3505
108.3403 26.3837999999998 -7.07610000000002
110.4326 30.3487999999998 -5.25260000000003
115.2443 25.1888999999997 -10.4824
-0.655999999997574 1.02500000000001 -0.213499999999921
0.824800000002975 4.20940000000015 0.190700000000082
39.6013000000019 11.1090000000002 12.3014
60.4480000000012 21.7631000000002 -0.515900000000015
56.0261000000012 23.3236000000001 -7.59880000000002
39.1632000000018 5.69700000000015 12.3572
84.4368000000005 14.2851999999999 25.1348
107.2048 18.4971999999998 20.2618
108.7366 35.6496999999998 2.41589999999998
120.3552 20.8468999999998 11.9236
113.7849 28.6722999999997 -2.84290000000002
-0.52419999999767 -0.411900000000024 -0.1070999999999
1.84860000000203 -2.91160000000011 0.160800000000124
41.1423000000017 1.28960000000011 12.0407000000001
65.359300000001 -1.57039999999992 -0.175600000000048
61.0439999999998 -4.44750000000003 -7.88819999999993
114.932199999999 2.69519999999976 2.44669999999999
117.311099999999 11.9030999999998 -2.69060000000002
ID=TAMmexUNKGUAMfNB81440*

LM3=54
5.0609 -3.507 -9.0786
6.9804 -0.5568 -8.007
53.5818 -2.5787 -4.5636
64.0713 -0.9702 -5.7595
52.191 8.406 -3.3216
71.2008 4.4216 -4.6869
73.2008 4.7831 -5.965
74.9312 0.0222 13.5634
88.6252 14.8453 -10.6619
83.7383 6.8319 -0.1777
86.558 4.5408 -4.3242
94.8728 -6.4919 -3.7926
94.1121 6.2375 -9.5738
109.1853 0.1371 -6.5611
116.6575 8.9365 -8.4165
120.3095 11.4758 -8.1082
119.7171 19.4967 -10.8215
127.3406 21.0158 -2.3751
123.0044 8.4 -5.7111
128.0838 14.184 -10.7181
2.7775 4.8713 -8.5268
5.6347 2.8902 -7.911
48.5474 19.3905 -3.286
59.6767 21.6695 -4.9413
68.1037 19.6625 -4.3235

69.591 18.4907 -4.9353
68.9964 23.4431 12.278
81.0856 20.7299 -0.2556
82.8887 23.3522 -3.972
88.0518 36.3325 -2.9175
91.2171 24.5544 -9.5421
102.6325 35.2922 -5.5952
113.7892 28.973 -7.6962
117.8694 27.4489 -7.9029
120.0894 31.3946 -4.4639
125.3563 27.5935 -10.18
-0.5958 0.328 -0.0781
1.5977 4.1353 -0.177
40.0164 11.328 12.5166
62.5302 24.2625 -0.1662
59.8667 25.3047 -8.1769
42.3737 6.5973 13.2719
88.6994 14.7924 23.4642
110.7434 17.9149 19.791
115.9267 35.7996 1.944
122.6553 20.2318 14.1861
121.7868 28.9544 -3.5229
-0.5063 -0.677 -0.0117
2.8035 -3.693 0.262
40.398 1.9117 11.6762
68.1443 -2.3766 -0.608
65.3805 -3.9583 -8.61
120.9895 3.2451 1.3754
124.2318 10.6988 -3.8912
ID=TAMmexFEMGUAMfNB81441*

LM3=54
5.01140000000482 -3.64349999999877 -8.12390000000062
6.70830000000402 -1.35569999999895 -7.66260000000043
52.9923000000026 -1.44829999999947 -3.09580000000001
63.4527000000024 0.509500000000479 -4.42049999999999
50.7758000000028 8.75850000000055 -3.28120000000003
70.1051000000018 4.42340000000036 -3.75159999999991
73.6052000000017 6.15060000000035 -4.50929999999989
75.9570000000021 0.419100000000416 12.9080000000002
92.1156000000005 14.7216000000003 -10.7240999999997
84.0374000000009 8.38190000000027 0.196700000000224
88.2163000000007 5.36240000000026 -4.86839999999971
95.9291000000007 -4.78119999999966 -3.44789999999964
95.5579000000002 5.74770000000025 -8.9397999999996
110.5727 -0.339999999999657 -5.93879999999955
118.635200000001 8.04160000000034 -8.8060999999995
122.730100000001 10.2770000000003 -8.69929999999949
123.0867 18.2526000000003 -11.1186999999995
129.842300000001 19.0842000000004 -1.81709999999948
125.213100000001 6.95460000000041 -5.67809999999948
129.993600000001 12.4691000000004 -11.5593999999995
3.89500000000499 3.86850000000113 -8.6138000000003
6.34320000000472 1.92630000000112 -7.79470000000027

50.0538000000026 19.6154000000005 -3.52160000000002
59.6584000000023 20.6478000000005 -4.98929999999997
68.2514000000018 18.8689000000004 -4.7609999999999
68.0910000000016 17.5514000000003 -4.36599999999987
73.2509000000018 25.0091000000004 12.5839000000001
81.8208000000012 19.6500000000003 -0.997699999999765
86.185400000001 22.3509000000003 -5.45119999999973
90.886100000001 34.3985000000003 -3.88189999999971
94.0152000000004 23.9928000000002 -9.32809999999964
106.430300000001 33.7902000000003 -5.70989999999959
116.9998 27.1679000000003 -9.40519999999954
120.897 25.1841000000003 -9.28139999999952
123.600900000001 29.5500000000004 -5.73969999999952
128.979800000001 25.0678000000004 -11.3728999999995
-0.387999999994937 0.196300000001182 -0.20730000000025
1.67160000000499 3.54630000000118 0.207599999999724
41.9573000000035 12.7614000000007 13.0564999999999
65.4876000000021 23.6407000000004 -1.21139999999992
59.9145000000022 24.0144000000005 -7.72549999999995
45.1112000000033 8.90960000000066 14.2094999999999
90.7177000000013 14.7853000000003 22.0818000000003
112.553100000001 17.6100000000004 18.9318000000004
118.240500000001 33.9831000000004 2.05010000000045
125.822100000001 19.2817000000004 12.8104000000005
125.887100000001 27.3753000000004 -3.22129999999952
-0.256299999994974 -0.347299999998815 -0.0489000000002659
3.36190000000503 -2.87039999999883 0.623299999999745
43.1741000000035 3.81110000000068 12.9350999999999
68.5165000000021 -0.447499999999608 -1.40699999999993
64.1830000000024 -2.77399999999953 -7.02449999999995
121.998800000001 2.75110000000039 1.65680000000049
127.397900000001 10.7864000000004 -3.1170999999995
ID=TAMmexUNKGUAMfNB81442*

LM3=54
4.26839999999781 -2.7375000000002 -8.22549999999962
5.14060000000006 -0.244799999999535 -7.59999999999959
54.628799999999 -2.09419999999983 -4.48159999999972
61.6303000000004 -0.610299999999743 -5.29709999999987
51.1957999999988 8.97110000000038 -3.16169999999977
68.7822999999996 4.20440000000029 -3.65679999999989
72.0321999999997 5.68770000000034 -4.0237000000001
80.6880999999989 2.26830000000014 -0.532799999999931
88.0435999999997 14.3875 -9.23569999999984
84.3816000000002 7.39030000000019 -0.70389999999997
87.9406999999999 4.99630000000011 -5.18449999999996
94.3875999999994 -5.15110000000001 -3.61729999999987
95.5930000000003 5.15580000000003 -7.53329999999989
106.990799999999 0.556499999999949 -4.42519999999986
115.515199999999 9.79629999999989 -5.77659999999982
118.7704 11.5246999999999 -6.05929999999981
120.0927 18.5597999999999 -7.93349999999981
125.6243 19.6771999999999 1.00650000000014
121.727199999999 8.37379999999986 -3.21299999999981

124.998499999999 14.6045999999999 -9.03389999999979
2.68010000000035 5.22550000000067 -8.45579999999995
4.71310000000059 3.00640000000065 -7.71279999999993
50.9712999999996 19.8138000000003 -4.28999999999991
58.5101999999996 20.4638000000003 -5.14279999999992
66.6914999999997 18.8177000000003 -3.89379999999996
69.8153999999998 18.0987000000003 -4.05109999999996
68.9677999999996 24.9379000000002 13.7937000000001
82.3114999999997 18.5623000000001 -0.716499999999942
84.6008 22.3561000000001 -4.90299999999992
88.9612999999999 34.2794000000001 -3.5974999999999
92.6132999999999 24.1963 -7.87499999999987
103.5322 33.2577 -4.80019999999988
113.4476 26.1139999999998 -5.33219999999986
116.7477 24.1518999999999 -6.68719999999983
118.999 28.7453999999999 -3.40939999999984
122.1481 22.5630999999998 -9.17129999999983
-0.627000000000702 0.0271000000003963 -0.313199999999853
1.57039999999991 3.44850000000051 -0.0333999999998493
41.3386999999997 12.3273000000004 12.5859000000001
63.0486999999997 22.4443000000003 0.40910000000005
58.0912999999996 24.4612000000003 -7.95669999999993
46.8943999999995 7.71500000000034 14.5882000000001
90.0437999999995 14.1195000000001 23.4537000000001
109.5804 17.3291 19.3663000000002
113.4428 34.8440999999999 3.08600000000015
122.0065 19.0817999999999 14.6121000000002
120.4849 26.6344999999999 -0.311399999999823
-0.618000000000845 -1.37449999999965 0.0464000000001664
2.74469999999894 -3.68559999999973 -0.207199999999817
42.7056999999994 1.32560000000033 12.6463000000001
66.4959999999993 -1.75519999999978 0.667100000000042
63.0457999999981 -3.45649999999989 -7.96639999999986
118.577199999999 1.75369999999988 3.7403000000002
121.922599999999 9.77669999999987 -0.945699999999818
ID=TAMmexFEMCRIAMNH141988

LM3=54
4.33489999999478 -3.91800000000037 -9.03639999999995
5.50559999999773 -1.80530000000051 -8.47919999999996
53.884699999998 -3.54060000000031 -3.51559999999998
60.7008999999989 -2.37900000000016 -4.58629999999999
48.4835999999977 7.05279999999963 -3.59049999999985
66.0656999999981 1.6551999999996 -3.76989999999986
67.9359999999996 2.85079999999993 -4.12439999999993
71.2565999999978 -2.48550000000026 13.5516000000001
87.4377999999994 13.3848 -10.8755999999999
79.4660999999986 6.07619999999987 -0.738099999999784
83.1020999999985 3.38669999999984 -5.25439999999978
92.3525999999972 -5.9033000000002 -3.17459999999972
92.0534999999987 3.90829999999997 -8.64189999999978
105.593799999998 -0.350400000000095 -4.77599999999971
111.809499999998 8.42859999999994 -8.35579999999969
114.586699999998 10.3832999999999 -7.23469999999967

115.552399999998 18.3468999999999 -10.1581999999997
122.224099999997 19.5340999999999 1.82060000000034
118.011599999997 7.04189999999985 -3.75799999999965
119.766199999997 13.8745999999999 -10.2771999999996
3.34309999999768 3.26849999999968 -9.3149
4.75219999999792 2.07009999999966 -8.94980000000003
50.4259999999983 18.6642999999998 -3.98159999999991
57.3308999999986 20.3425999999998 -4.87979999999991
64.1287999999991 17.0435999999999 -3.72529999999992
65.4337999999992 16.1740999999999 -4.34329999999991
68.5522999999985 23.5344999999998 13.2807000000001
77.7850999999989 17.0903999999999 -1.12249999999987
80.3207999999992 21.6018 -4.88639999999986
85.6447999999988 33.0889 -3.39599999999984
88.686799999999 23.7088 -9.01889999999983
99.4752999999985 32.5764 -5.79679999999978
108.024099999998 26.3188 -7.69909999999974
112.402499999998 25.6605999999999 -7.29209999999969
114.079499999998 30.2035999999999 -4.33889999999969
117.097199999997 23.0636999999999 -10.9091999999997
-0.829100000003717 0.352799999999516 -0.428099999999915
0.406999999996943 3.71899999999961 -0.861999999999984
39.655399999998 11.3148999999998 11.5576
60.7252999999988 22.0540999999999 1.94860000000007
57.5059999999987 22.3494999999999 -7.72199999999993
42.2139999999977 6.84539999999976 13.7372999999999
85.7414999999978 13.5121999999998 22.8591000000002
106.311799999998 16.8073999999999 19.4007000000003
106.913799999998 34.4477999999999 2.24840000000028
118.571599999997 19.0923999999999 14.8466000000003
114.891399999998 27.9773999999999 -0.972399999999695
-0.571200000003963 -0.893400000000515 -0.0755999999999225
1.43339999999566 -3.45020000000058 -0.459499999999907
41.1571999999976 0.548499999999755 11.8431999999999
64.7169999999989 -3.71960000000014 1.67900000000007
61.9485999999971 -4.68750000000029 -6.87219999999977
113.760399999997 0.597199999999853 2.66060000000033
118.064799999997 8.90369999999987 -0.896799999999657
ID=TAMmexUNKCRIAMNH10087*

LM3=54
3.75659999999422 -3.7683000000006 -8.51450000000005
4.81029999999652 -1.49800000000014 -8.33410000000005
50.2991999999997 -3.66749999999996 -4.94630000000012
57.648199999999 -3.12260000000009 -5.92320000000011
46.4427000000017 6.45680000000033 -5.08000000000021
64.7675000000001 0.992300000000105 -4.93960000000014
66.5676999999993 3.1616 -5.25910000000009
68.3914999999995 -1.57879999999998 13.4866999999999
85.8720999999997 13.0392999999999 -10.3561
76.7402000000007 5.3682000000001 -0.460400000000144
80.2361999999997 2.75650000000008 -4.72040000000011
89.5197999999992 -5.50600000000007 -3.58310000000009
90.1728999999996 4.09290000000004 -8.33440000000009

102.652 -0.362000000000001 -5.06680000000014
110.590999999999 8.1495999999999 -7.55540000000011
113.803 11.0898999999999 -7.62780000000013
114.7046 18.4181999999999 -9.55120000000012
120.4216 19.3975999999999 1.79269999999986
117.7557 8.10119999999992 -4.57050000000014
119.327999999999 14.0012999999999 -10.4498000000001
2.86380174472122 4.11636986999421 -8.80376036817805
4.90506757304338 2.33260953327023 -8.31863300762792
47.226500000001 17.3377000000003 -5.23150000000015
54.3688000000008 18.8733000000002 -6.19500000000013
62.0817000000004 16.9442000000001 -5.60160000000009
64.0924000000002 16.0928000000001 -5.48860000000009
66.7458000000003 22.3684000000001 12.5840999999999
74.8004000000004 17.4657000000001 -1.00960000000009
77.1811000000002 21.0676000000001 -4.56240000000008
82.8316000000003 31.6226000000001 -3.8289000000001
85.8231000000002 22.4689 -8.52810000000009
96.7659000000001 31.5094 -5.39800000000011
107.897 26.1747 -7.21070000000011
112.1853 24.4881 -8.04550000000012
113.793 29.1234999999999 -3.97510000000012
116.7132 23.4590999999999 -10.7593000000001
-0.518600000004386 0.663399999999758 0.625299999999925
0.604999999996105 4.18309999999985 0.110599999999925
36.9905000000001 11.6668000000002 11.3306999999999
60.9994000000007 20.4303000000002 0.854499999999877
55.5204000000006 22.0685000000002 -8.24460000000012
38.9801 6.21440000000018 13.3294999999999
84.3760999999999 13.2321 21.3040999999999
103.317 16.6766 18.4584999999999
108.8068 32.3254 2.84969999999987
118.598699999999 18.7177999999999 12.2936999999999
115.8108 27.0415999999999 -2.26270000000013
-0.194000000004428 0.138999999999741 0.378699999999928
1.73529999999548 -2.94830000000029 0.0766999999999486
38.2406999999997 0.147600000000145 11.7306999999999
64.4036999999997 -2.13289999999992 1.06249999999989
60.2388999999984 -5.62180000000009 -7.65950000000003
111.634599999999 1.62439999999993 3.64339999999987
118.3697 9.92749999999992 -1.61640000000014
ID=TAMmexFEMHONAMNH128128

LM3=54
4.5905000000016 -3.68439999999914 -9.33710000000057
5.74428515945703 -1.63020070838275 -8.4825115297989
56.580200000003 -2.07579999999969 -3.69440000000012
63.4675000000034 -1.73949999999959 -4.15700000000013
53.2432000000039 7.46560000000019 -3.68600000000017
70.5458000000016 3.39329999999995 -3.05980000000003
73.5571000000022 5.00820000000021 -3.73940000000011
76.8092000000032 -1.32279999999965 14.4831999999998
96.1067000000022 14.9659000000001 -9.55400000000014
85.8425000000021 6.78210000000016 1.09649999999985

88.6843000000013 4.46960000000009 -2.27200000000017
100.054000000003 -5.16349999999961 -3.28800000000025
98.768300000002 5.31860000000018 -7.53760000000014
113.102800000002 0.71350000000022 -5.27580000000016
121.696200000002 9.09610000000018 -7.59970000000019
124.883700000002 11.1388000000002 -7.66140000000014
125.751800000002 19.0833000000002 -9.89280000000016
131.979000000002 20.6335000000002 -0.746100000000191
129.187000000002 9.02140000000021 -5.47340000000018
131.706900000002 14.1759000000002 -10.7333000000002
3.46150000000299 3.54500000000015 -9.0307000000002
5.36870479094486 2.13580413706916 -8.60506825767432
53.2421000000035 19.0117000000002 -3.74520000000017
60.436700000003 20.2129000000002 -4.50270000000015
68.5098000000027 18.0124000000002 -3.39890000000013
71.5008000000028 16.9197000000002 -3.00620000000015
72.8183000000034 23.7075000000003 14.7302999999998
83.5972000000024 19.3527000000001 1.27359999999984
85.5885000000024 21.9009000000002 -2.67340000000017
94.1913000000025 34.3967000000002 -3.99330000000016
96.1220000000021 23.9769000000001 -7.86420000000014
108.301100000002 33.5465000000001 -5.40270000000015
118.652600000002 27.5481000000001 -8.10320000000015
122.584100000002 26.8955000000001 -7.02840000000015
125.987500000002 30.2512000000002 -5.49370000000017
129.485800000002 25.7509000000002 -10.8519000000002
-1.47289999999732 0.57510000000027 -0.410000000000235
0.837300000002867 3.99370000000021 -0.707200000000225
43.5224000000034 12.0930000000002 13.6721999999998
67.8403000000032 22.4613000000002 2.58069999999985
60.863000000003 22.7578000000002 -6.14320000000017
48.3510000000034 6.99580000000029 17.2445999999998
93.5694000000031 15.1648000000003 23.1787999999998
115.724400000003 17.6628000000002 18.9793999999998
119.948900000002 34.6108000000002 3.25079999999982
130.188000000003 19.9798000000003 13.3325999999998
127.057000000002 28.2345000000002 -1.89550000000016
-1.15259999999745 -0.645299999999709 -0.165700000000236
2.15510000000246 -4.00219999999972 0.217999999999751
45.2779000000033 0.819900000000272 13.7171999999998
71.9318000000021 -0.840199999999844 2.98659999999992
64.9584000000026 -3.84989999999969 -6.31980000000022
125.943700000002 3.46530000000024 3.0567999999998
129.595500000002 11.0956000000002 -2.54340000000017
ID=TAMmexFEMHONAMNH128140

LM3=54
2.84019999999385 -2.92490000000064 -9.48910000000037
4.71449999999448 -0.774700000000496 -8.65859999999972
54.9040999999966 -2.5114000000004 -4.67559999999999
61.322699999997 -2.04900000000039 -5.59969999999998
47.5719999999972 7.42589999999969 -5.11619999999997
68.4831999999975 3.01499999999967 -5.34109999999997
71.9860999999976 5.19669999999969 -5.63339999999996

78.3247999999975 -0.774500000000414 13.0293
92.334099999999 14.2179999999997 -11.5255999999999
83.2251999999983 7.26399999999969 -1.95639999999995
87.2189999999984 4.98549999999971 -6.79179999999993
94.5099999999985 -5.43040000000033 -3.96589999999987
96.1824999999987 4.32949999999969 -9.87089999999989
107.451099999999 -0.814000000000316 -6.54219999999989
116.1656 8.12599999999972 -7.55639999999991
120.5359 9.93499999999972 -6.77039999999992
120.2805 18.6629999999997 -9.75459999999993
126.0006 20.1385999999996 4.89990000000008
123.5889 6.71129999999965 -3.0682999999999
126.467351644229 12.8906252893902 -8.281422923528
2.09099999999433 3.26399999999933 -9.31899999999987
4.17688590444817 1.91248637374506 -8.95930932886191
51.6447999999972 18.1082999999997 -4.87549999999999
57.8639999999977 19.4904999999997 -5.75000000000002
66.502499999998 17.3698999999997 -5.28
70.056799999998 16.7790999999997 -5.65780000000001
71.8701999999981 22.4346999999997 14.321
80.7548999999988 18.8825999999997 -1.96649999999997
83.9799999999989 22.4172999999997 -6.48449999999997
89.1945999999989 33.6752999999997 -4.75949999999997
93.6497999999994 25.3013999999998 -10.1881
103.92 33.5859999999997 -6.58889999999995
113.3355 26.8826999999997 -7.22799999999993
118.479 25.8868999999997 -7.15989999999994
120.180248602553 30.3534810796462 -4.51621061526733
124.506915519738 25.6141493408449 -8.71734495036324
-0.653600000005718 1.57349999999932 0.308600000000173
1.6192999999944 4.43729999999932 1.31620000000014
42.9833999999967 12.6303999999996 11.9094
64.7246999999981 21.1647999999997 -0.286200000000016
58.0983999999979 22.4182999999997 -7.63940000000003
44.0969999999966 6.40349999999954 13.1248
91.4708999999984 14.0189999999996 21.2125
110.053499999999 16.8274999999996 19.3399000000001
113.9639 34.1644999999997 3.14470000000005
124.2261 18.5781999999996 14.9033000000001
121.7216 28.2040999999996 -2.03159999999993
-0.356700000005794 -0.382600000000674 0.142400000000115
2.73999999999438 -2.8793000000007 1.05020000000012
44.8735999999963 0.335399999999506 11.343
67.9610999999973 -1.79190000000037 -0.814199999999966
62.2972999999971 -4.66760000000039 -7.56249999999996
119.5009 1.40899999999962 3.3674000000001
125.6525 9.02569999999966 -1.14469999999991
ID=TAMmexFEMCOLAMNH14222*

LM3=54
3.15279999999196 -3.90560000000044 -8.67830000000002
4.25910000000066 -1.5518999999994 -8.46960000000024
52.0387999999983 -2.73330000000002 -4.28480000000003
61.5517999999994 -2.48610000000008 -5.44790000000023

48.5341999999989 7.02720000000008 -4.35610000000015
68.4218999999985 2.54309999999987 -5.02180000000015
70.2176999999989 4.84659999999988 -5.47950000000019
75.2896999999979 -2.00920000000016 14.2522999999999
91.358099999998 14.3727999999997 -11.3626000000001
82.2454000000001 5.8032000000001 -0.800700000000253
87.2377000000002 3.74210000000015 -5.47380000000036
95.2084999999988 -6.09500000000003 -4.52650000000022
96.3623999999988 4.83819999999991 -9.68150000000019
110.234999999998 -0.399100000000184 -6.63810000000018
117.727299999999 8.90749999999986 -8.06410000000021
120.809399999998 12.2350999999998 -8.21750000000018
121.092799999998 19.7436999999998 -10.5785000000002
127.969299999998 20.5134999999997 -1.8198000000002
124.582099999999 9.00699999999983 -5.83440000000021
126.204199999998 14.9281999999998 -11.6592000000002
1.02193255988649 4.30907217388422 -9.38366628683812
3.27348628803009 2.52117978710945 -8.74761219863873
48.832799999999 17.7688000000001 -4.26700000000015
58.2760999999991 19.7392 -4.97620000000017
65.8897999999991 17.53 -4.37750000000018
67.7897999999992 16.7314 -4.95890000000018
68.0743999999987 22.9530999999999 14.5330999999998
80.0982999999992 19.2024999999999 -0.682400000000195
83.7757999999988 22.9883999999999 -4.67160000000017
87.8788999999987 34.5073999999998 -4.30890000000018
92.4230999999984 24.7041999999998 -9.90560000000013
103.881599999998 34.7794999999998 -6.46970000000017
114.016299999999 28.1308999999998 -7.55630000000017
118.295099999998 25.9374999999998 -8.42070000000017
120.432099999998 30.7895999999998 -5.48100000000019
124.125499999998 25.5568999999998 -11.4895000000002
-0.792300000004334 0.530899999999954 -0.0540000000001102
0.0718912640684266 3.80369019170819 -0.407444199749053
41.6579999999977 11.6440999999999 12.8125999999999
61.6659999999991 22.1881 0.489099999999817
58.4623999999991 23.3909 -7.05940000000018
43.6742999999976 6.25639999999991 15.3210999999999
90.9120999999981 14.4684999999998 22.8908999999998
111.302499999998 17.5594999999998 20.0672999999998
116.916899999998 34.7472999999998 2.82289999999978
126.267499999998 20.6965999999998 12.6455999999998
121.384199999998 28.5146999999998 -2.48940000000018
-0.409400000004653 -0.617100000000075 -0.530700000000097
2.35599999999454 -3.72810000000019 0.359599999999993
44.0666999999972 0.205099999999853 12.8463999999999
67.1242999999978 -3.16680000000019 0.487299999999988
62.9950999999984 -5.05649999999998 -7.77420000000009
123.123699999998 3.09839999999984 3.2545999999998
125.121699999999 11.6082999999998 -1.56320000000021
ID=TAMmexFEMMEXAMNH143970

LM3=54
4.0922 -2.0552 -8.2869

5.491 -0.1856 -8.2093
53.1544 -2.2301 -5.6978
59.4214 -0.7956 -6.427
48.2888 8.0578 -4.8607
66.5719 3.358 -6.4341
68.3161 5.6679 -6.5521
71.0688 -0.671 10.3334
89.2986 15.0229 -11.9837
80.5657 6.7744 -2.1936
83.5062 4.4228 -6.5886
92.3175 -5.1812 -4.9522
91.9531 5.88 -9.8922
105.7222 -0.1254 -6.1831
113.1101 9.2167 -7.7153
116.8219 11.4559 -7.4848
118.0218 19.0173 -10.2544
122.1592 19.3785 -0.5282
119.7457 8.1075 -5.6865
123.645 13.2997 -10.3479
3.0611 5.342 -8.2564
5.084 3.526 -8.1103
50.3331 19.3012 -4.8158
56.5508 20.5986 -5.6127
63.3833 17.1019 -5.4205
65.814 16.643 -5.8152
67.348 21.5158 11.1676
78.2333 17.8665 -2.5286
80.4954 22.6003 -6.1356
85.4947 33.806 -4.1783
88.9528 23.6361 -9.753
100.1701 33.7483 -4.4079
110.072 27.2393 -6.7799
114.6713 26.158 -7.4023
116.6246 30.0541 -5.0445
121.8237 25.6057 -10.0675
-0.4306 1.3654 0.3432
1.4615 4.2744 0.8419
39.3185 10.9226 10.0101
62.9482 20.9699 -1.0858
57.725 23.7472 -7.7957
41.6909 6.7827 11.6868
86.6499 14.0092 19.2706
105.4188 16.7497 18.0833
112.7941 33.1246 2.0246
119.1639 19.1717 12.3422
116.4287 27.246 -2.9075
-0.2551 -0.1573 -0.2172
2.6094 -2.619 0.364
40.6194 1.5751 9.5916
66.0717 -0.5798 -1.369
61.9366 -4.1114 -8.7346
116.1218 2.4612 2.5476
120.1384 9.9464 -2.2423
ID=TAMmexFEMMEXAMNH148801

LM3=54
3.27220000000117 -3.56909999999999 -8.74400000000006
5.31690000000299 -1.67720000000004 -7.89130000000001
53.3364999999998 -2.37620000000016 -5.35199999999999
61.0016999999994 -0.640800000000215 -6.52669999999992
41.5279000000001 6.89779999999982 -4.33959999999986
68.3676000000021 3.72470000000019 -5.31239999999998
70.5968000000017 6.20700000000025 -5.56870000000004
74.4161000000025 -1.04539999999974 12.6705999999999
90.8409000000004 15.2394000000001 -11.2380999999999
82.4208000000023 8.36170000000025 -0.85360000000004
86.5387000000015 5.19560000000017 -4.85869999999997
95.6105000000016 -4.08849999999988 -5.1475
95.5542000000007 6.20480000000007 -9.50169999999999
109.065000000002 0.530000000000182 -6.40070000000006
116.938600000002 9.10120000000024 -8.02980000000008
121.723900000002 12.1397000000003 -7.99140000000011
122.476600000003 19.3385000000003 -10.2618000000001
127.870500000003 20.3131000000003 -1.44300000000009
124.444600000002 8.32200000000027 -5.2640000000001
128.266800000003 12.7687000000003 -10.7352000000001
2.37337491884404 5.17014054412096 -9.07477133576152
4.83239123115495 3.14652082425339 -8.2951179516179
50.3172000000005 20.0593999999999 -5.99049999999988
58.2686000000008 21.1656 -6.62169999999991
65.9501000000012 19.1126000000001 -5.71859999999995
68.7031000000012 19.6030000000001 -5.86029999999995
69.7495000000018 25.3096000000002 12.2694
81.7425000000017 18.7287000000002 -0.749400000000011
83.7462000000012 23.4053000000002 -4.87979999999997
90.4002000000015 35.4903000000002 -5.48060000000001
92.6832000000014 25.1233000000002 -9.2935
104.737600000002 34.3630000000003 -6.97890000000005
115.311000000002 27.7804000000003 -8.04110000000009
120.358700000002 26.6700000000003 -7.61970000000008
121.708800000003 30.9988000000003 -5.70760000000009
126.968800000003 26.8975000000003 -10.8675000000001
-1.08679999999808 1.32149999999986 -0.626999999999992
0.365600000001868 4.94389999999991 -0.537899999999978
41.2349000000008 14.3326 11.5199000000001
62.9388813948595 23.3931288519409 -0.0548519972428949
58.0131000000009 24.1422 -9.08949999999991
42.395900000001 7.51790000000001 13.4676000000001
88.2305000000024 15.2137000000002 21.8259999999999
109.273100000002 17.6019000000002 19.2826999999999
116.455800000002 36.1661000000003 1.33769999999993
123.799600000003 19.6917000000003 13.2826999999999
122.697100000003 29.2013000000003 -1.4303000000001
-1.00509999999809 -1.06280000000016 -0.422900000000005
1.330600000002 -3.79160000000008 -0.791499999999984
42.9189000000008 0.301400000000002 11.2725000000001
66.5092186848648 -1.59331262799998 0.230435339443769
62.1411000000027 -3.48929999999975 -8.73540000000003

119.322100000002 1.11120000000022 1.14209999999992
124.553000000002 9.15530000000028 -2.33590000000011
ID=TAMmexFEMMEXAMNH172180

LM3=54
5.0819 -3.9966 -8.4892
7.0482 -1.9895 -7.6362
51.8822 -2.2209 -3.5825
61.7537 -0.8902 -5.2665
47.4982 8.0617 -3.1541
67.8191 3.6232 -4.1849
70.0635 5.4756 -4.5687
72.7813 -0.5692 14.2366
90.7231 14.8235 -10.3823
82.766 6.7654 -0.3022
84.2522 5.1089 -3.0136
94.9502 -5.0937 -5.0215
94.7013 5.6034 -9.4386
109.9588 -0.103 -6.1728
118.1433 8.7338 -6.9853
121.0591 11.8019 -6.9544
123.0916 19.5419 -9.1569
127.8159 20.1432 0.919
125.664 8.3713 -4.3875
127.7227 14.3833 -9.7789
3.9362 3.5891 -9.6163
6.6124 2.4523 -8.3074
49.8196 18.5581 -5.184
59.0495 21.5686 -6.6596
66.0592 19.1853 -5.0358
67.4956 17.9387 -4.9567
68.9608 25.3932 13.0443
81.3193 19.5402 0.5203
84.8637 23.4691 -6.4977
89.4325 35.4894 -5.2979
92.1272 25.6256 -9.475
105.2488 35.2365 -6.237
115.8315 27.9152 -7.6222
119.2032 26.0677 -7.0612
122.0166 30.8423 -4.3935
125.8221 25.6957 -9.6727
-1.3783 -0.012 -0.814
0.4221 3.6984 -0.9186
38.965 12.9451 12.2762
62.8095 23.6483 0.2678
60.3152 24.5421 -9.3292
41.1449 8.497 15.2346
90.5573 15.3923 21.9938
110.1484 17.648 19.2046
118.0972 34.813 3.7737
123.0366 20.0641 14.3856
122.5562 28.1998 -0.7814
-0.6169 -1.5595 0.0392
1.7817 -3.7036 0.1121

40.774 2.831 13.0407
66.2954 -1.308 1.7802
63.2908 -3.8595 -7.6171
121.0367 3.3168 3.0472
124.7793 11.0486 -0.3082
ID=TAMmexFEMMEXAMNH17269*

LM3=54
4.00520000000188 -3.23609999999888 -9.0772000000001
5.03869999999815 -1.12349999999957 -8.56769999999978
52.8003000000009 -3.04139999999958 -3.67740000000007
60.3122000000004 -1.8839999999997 -4.63410000000006
47.4321999999987 7.32800000000012 -3.74049999999984
67.0922 2.9711000000002 -3.27960000000002
69.2492000000009 4.29440000000027 -3.80600000000017
70.8646000000003 -2.57799999999966 14.6200999999999
88.2902000000006 14.5230000000003 -10.1697000000003
80.6021999999998 6.13790000000018 0.305599999999756
83.8218999999988 4.04400000000011 -3.91460000000017
92.7261000000004 -5.75529999999961 -4.25490000000029
92.7344000000006 4.95400000000031 -8.75980000000031
106.2444 -0.120399999999621 -6.65240000000035
114.8285 8.84420000000036 -9.39170000000038
118.5029 12.0129000000004 -9.50160000000042
119.6507 19.9442000000003 -11.7532000000004
125.026 20.4502000000004 -1.74720000000044
122.758700000001 8.63040000000043 -6.92960000000043
123.9973 14.3134000000004 -12.6737000000004
2.02017697961548 4.77718249062708 -9.13131806253862
4.27417564663317 2.93560174047751 -8.58877190985091
49.4309999999993 18.7611000000001 -3.25919999999993
57.0959999999994 19.6929000000001 -4.02959999999999
64.9248000000001 17.3984000000002 -3.22830000000006
67.2604 17.2231000000001 -3.61610000000008
66.4860999999999 22.8007000000002 15.0989999999999
78.0422000000001 19.0646000000001 1.14109999999982
80.4839000000003 22.9441000000002 -3.34200000000021
86.3647 35.0379000000001 -2.91570000000022
89.5474000000001 25.2394000000002 -8.28120000000024
100.523 34.5567000000001 -5.80400000000029
111.8735 28.2073000000002 -8.85950000000037
115.928 26.6873000000003 -9.1403000000004
118.9028 31.3860000000002 -6.13540000000038
122.8692 26.1084000000003 -12.3531000000004
-0.999199999999631 0.2881000000008 -0.137299999999935
0.101100000000031 3.80940000000069 0.0892000000000983
40.427 13.1328000000003 12.5001000000001
60.1963999999997 21.1961000000001 2.04069999999997
57.3903999999995 22.7183000000001 -6.75749999999999
44.2325000000001 6.3319000000003 16.5336000000001
89.6663 14.1197000000003 23.3407999999998
108.8029 17.0375000000003 18.7749999999997
112.5065 35.0133000000002 1.30999999999963
122.9929 19.9428000000004 12.7058999999996

118.8937 28.9928000000003 -2.97690000000042
-0.975299999999505 -1.01529999999916 -0.268999999999951
1.3159000000009 -3.80809999999913 -0.0968999999999676
43.0291000000005 -1.71209999999963 12.5222000000001
63.9106000000004 -3.07309999999972 2.15049999999995
61.8064 -4.42109999999967 -7.06219999999992
117.9186 2.42850000000043 1.7820999999996
122.1939 10.8770000000004 -2.77110000000045
ID=TAMmexMALMEXAMNH176663

LM3=54
3.47319999999976 -4.12100000000222 -9.28459999999962
4.80209999999931 -1.63840000000154 -8.36199999999969
51.3028999999996 -4.89730000000126 -6.19760000000004
58.3477999999994 -3.00980000000117 -6.92450000000005
43.4810999999995 5.99809999999874 -5.37510000000006
64.4161999999993 1.89429999999891 -6.05530000000009
66.0642999999995 3.57049999999897 -5.9603000000001
70.6123999999993 -2.04930000000109 13.5610999999999
87.2130000000002 13.0208999999993 -11.6942000000001
79.1441999999993 4.67189999999912 -1.06350000000012
82.9987999999993 2.54669999999917 -4.6075000000001
93.1021999999992 -6.81750000000084 -4.45190000000009
91.8339999999995 4.1993999999993 -9.9950000000001
106.3604 -0.0648000000006336 -6.50680000000012
113.6504 9.53889999999957 -9.05720000000017
116.9135 13.2068999999997 -8.94590000000019
117.306400000001 19.8616999999997 -10.5967000000002
122.544800000001 21.3671999999997 -2.11640000000025
119.4954 9.4928999999996 -6.94010000000021
121.288200000001 15.1937999999997 -10.9747000000002
2.45879999999957 4.05099999999827 -10.0557999999997
4.53099999999973 2.2806999999984 -8.98919999999968
47.3719999999999 19.0064999999988 -6.50370000000004
54.8528999999999 19.0657999999989 -7.28000000000007
62.0052999999997 16.615299999999 -6.52520000000008
64.3219999999998 15.570299999999 -6.3002000000001
67.6148999999999 22.933299999999 13.0040999999998
76.6313000000001 17.3938999999992 -0.436600000000131
78.8676000000003 20.2395999999993 -5.96930000000013
84.4305000000007 33.8047999999993 -4.53660000000015
87.2446000000005 22.5526999999994 -10.0706000000001
98.4575000000013 32.9600999999996 -6.20850000000021
109.382100000001 27.7065999999998 -8.57790000000024
113.883800000001 26.0844999999997 -8.32080000000025
114.912700000001 31.0461999999998 -5.88370000000026
118.292800000001 26.0400999999997 -11.5584000000003
-0.339300000000445 0.517299999998319 0.0595000000002881
0.707499999999626 3.87719999999834 -0.243299999999694
38.7043999999997 12.1078999999987 9.95619999999993
60.2917999999998 20.3502999999989 -1.00080000000009
55.5933 23.7177999999989 -10.9317000000001
42.2612999999995 6.4506999999987 12.8471999999999
88.5235999999998 13.7776999999992 22.0498999999998

106.0772 16.6101999999994 18.5816999999998
109.545700000001 35.9164999999997 2.69779999999974
121.499300000001 20.5272999999996 11.9788999999997
116.981000000001 28.9205999999997 -2.20340000000025
-0.236300000000465 -0.849900000001685 0.0519000000002971
1.89979999999948 -3.72860000000165 -0.156799999999725
41.1455999999993 -0.61310000000134 10.2525999999999
63.8681999999993 -2.66320000000113 -0.293000000000083
59.6016999999996 -6.74390000000111 -9.8984000000001
117.3053 2.7179999999995 2.50099999999982
121.3488 12.2052999999996 -3.2497000000002
ID=TAMmexUNKMEXAMNH176664

LM3=54
4.61379999999379 -4.43259999999955 -8.75129999999927
5.57468292966054 -2.14808892437869 -8.38567851469942
56.2860999999967 -3.0654000000003 -4.34409999999982
60.0709999999974 -1.24520000000032 -4.85349999999981
50.5186999999966 7.90509999999972 -3.70689999999977
68.7446999999978 3.40789999999968 -4.37669999999981
71.9390999999979 4.61009999999962 -4.39839999999981
76.0820999999973 -1.25620000000035 13.9411000000002
90.2644999999987 14.1867999999997 -10.3166999999997
83.7599999999984 7.30499999999967 -0.678699999999761
87.4929999999986 5.03579999999965 -6.20939999999974
94.7576999999981 -5.6764000000003 -3.54049999999966
96.3663999999987 5.0506999999997 -8.81159999999966
108.841499999998 -0.797000000000275 -5.07659999999954
116.020599999998 8.72009999999975 -8.08709999999946
120.245599999998 11.5510999999998 -7.82059999999942
120.175799999998 19.4316999999998 -10.7788999999994
126.401599999997 19.9789999999998 -0.313199999999342
123.836499999998 8.2856999999998 -6.05349999999939
124.572299999998 14.0333999999998 -11.5739999999994
3.53349999999335 4.65280000000004 -8.6366999999994
4.97560054834542 2.7920364657601 -8.65473006800173
52.0334999999969 20.0986999999998 -4.21089999999976
56.8499999999977 19.2827999999998 -4.45809999999981
66.4657999999979 17.3167999999997 -3.93729999999979
70.180999999998 16.2461999999997 -4.17329999999979
72.4174999999973 23.6638999999997 14.7164000000003
81.4981999999983 18.6738999999997 -0.973099999999739
83.8752999999985 21.6206999999997 -6.0074999999997
88.0420999999979 34.2496999999997 -3.52659999999961
92.0987999999985 24.6173999999997 -8.85319999999964
101.526399999998 33.4795999999997 -6.45259999999948
113.752299999998 27.0667999999998 -7.96839999999943
117.583199999998 25.2469999999998 -8.42769999999941
119.908099999998 30.3813999999998 -6.28739999999939
122.965999999998 25.0191999999998 -11.3325999999994
-0.583000000006926 1.28310000000003 0.303100000000616
0.743299999993142 4.29500000000004 0.537900000000604
41.1036999999957 12.7748999999998 13.7142000000002
62.6363999999976 22.1263999999997 -0.0507999999997922

58.0819999999978 22.7632999999998 -6.3553999999998
40.8028999999958 6.60709999999974 14.4738000000002
92.6160999999974 14.0257999999997 21.9979000000003
110.453499999997 17.0629999999997 17.8135000000005
113.275399999997 35.5461999999997 4.12650000000057
124.428199999997 19.3854999999998 11.6590000000006
120.357799999998 28.2367999999998 -2.35039999999938
-0.253400000006749 -0.154699999999924 -0.405299999999382
2.2674999999931 -2.99220000000001 0.220100000000589
43.4582999999955 0.484799999999703 13.6280000000002
66.0167999999974 -2.66600000000035 0.0721000000001493
61.6257999999974 -4.4063000000003 -6.88849999999982
119.092199999998 0.495899999999779 3.53150000000058
123.009699999998 10.3138999999998 -2.61879999999994
ID=TAMmexFEMPANAMNH18884*

LM3=54
4.57609999999528 -3.69890000000093 -8.15649999999972
5.2163999999988 -1.6220999999999 -7.55120000000007
56.9306999999976 -3.47120000000006 -5.79229999999973
64.874899999999 -1.63269999999989 -6.61819999999995
52.4637999999974 7.53610000000006 -5.6902999999999
70.6882999999986 3.1186 -6.2563000000001
72.8923999999987 4.26200000000004 -6.79100000000015
74.8646999999979 -1.37170000000017 13.4204
94.5606999999992 13.5871999999999 -12.6355
85.3386999999995 6.52319999999994 -1.83620000000009
89.1463999999992 2.61389999999988 -6.19280000000009
98.260999999999 -6.62020000000029 -4.68429999999999
98.5932999999999 4.66009999999984 -10.5213
112.9659 -0.813200000000287 -6.35539999999998
120.5512 7.75839999999969 -7.97809999999996
124.2898 11.1249999999997 -8.82359999999998
125.6237 18.4969999999998 -10.8509
130.2047 19.0839999999997 -1.21919999999994
127.947 7.42659999999965 -6.14919999999995
129.9582 12.8875999999997 -11.2276999999999
2.97119999999813 3.64899999999998 -8.86709999999991
4.87439999999852 2.00310000000001 -7.95599999999995
53.4048999999979 19.1273 -5.96449999999997
62.1463999999981 19.3321 -7.00280000000002
68.8125999999984 17.3733 -6.62080000000006
71.3358999999986 17.2274 -7.01160000000008
73.2503999999984 23.1265999999999 13.4034
83.6929999999989 18.1872999999999 -1.54340000000006
86.0940999999992 22.4747999999999 -5.80450000000005
92.4622999999992 34.5558999999999 -5.03790000000003
95.7879999999994 23.5153999999999 -10.8303
107.692 33.3159999999999 -6.1548
117.8243 27.2050999999997 -7.74579999999997
121.4089 25.2709999999997 -9.16659999999998
123.7479 30.0302999999998 -6.13529999999997
128.2233 25.1772999999997 -11.3237999999999
-1.99680000000322 0.235899999999696 -1.08419999999978

0.283799999997573 3.89579999999982 -0.883299999999855
40.651199999998 13.9636 10.3234
66.1032999999984 21.7074 -1.25230000000004
62.4823999999981 23.8329 -9.73550000000003
42.0420999999979 6.28869999999996 13.6902
91.617599999999 13.7289999999998 21.636
111.0727 16.8920999999997 18.6123
117.8504 33.6631999999997 3.65900000000001
125.8056 18.5047999999997 12.0677000000001
123.8612 27.7745999999997 -2.20039999999998
-1.8110000000033 -1.27580000000034 -0.892299999999761
1.16659999999642 -3.91580000000053 -0.0987999999997094
41.2682999999978 -1.28030000000012 10.6536000000001
68.4925999999982 -2.69150000000008 0.368699999999934
67.3114999999968 -5.21730000000017 -8.91939999999992
121.6768 1.15689999999964 3.90170000000006
126.7208 9.91629999999968 -1.80269999999994
ID=TAMmexFEMMEXAMNH190192

LM3=54
4.98349999999875 -3.36370000000018 -8.70669999999992
5.70239999999845 -0.980199999999621 -7.84890000000061
52.3858 -2.5151999999997 -4.09240000000033
59.025199999998 -0.764200000000151 -4.64500000000015
48.5896999999997 6.89760000000026 -4.15210000000038
66.7870999999994 3.29120000000005 -4.56640000000015
67.6331999999987 5.69279999999989 -4.82470000000014
71.7854999999986 -1.53150000000006 12.3841999999998
84.2520999999991 12.6787999999998 -11.2030000000001
79.8652999999983 7.04649999999982 -0.941800000000013
82.8796999999993 3.32189999999987 -4.50110000000012
91.9520999999984 -5.61560000000019 -4.0809000000001
91.5217999999987 3.40589999999985 -8.7135000000001
104.580299999999 -0.701000000000154 -5.20180000000015
111.979899999999 8.81959999999981 -8.06280000000018
115.972499999999 11.0915999999998 -8.38200000000017
116.314299999999 18.1215999999998 -10.1509000000002
121.569299999999 18.8109999999998 -1.03910000000016
118.863299999999 8.04049999999983 -6.00170000000017
120.599299999999 13.5249999999998 -10.7099000000002
3.58059999999818 4.72410000000029 -8.47600000000025
5.22149999999804 3.15130000000028 -8.02740000000033
49.249399999999 18.4039000000001 -4.48060000000028
55.7604999999989 18.749 -5.16350000000026
63.6459999999988 16.8087 -5.1523000000002
65.3809999999989 15.4392999999999 -4.85810000000019
67.8558999999988 23.7276 11.9813999999998
78.3179999999987 18.4885999999998 -1.21790000000015
79.4996999999989 21.8781999999998 -4.70770000000016
85.5133999999988 33.6417999999998 -3.68360000000019
87.3407999999989 24.1325999999998 -8.85240000000017
99.680899999999 33.0926999999998 -4.23710000000018
108.889899999999 25.4785999999998 -8.39510000000018
113.275699999999 24.1557999999998 -8.44910000000018

114.326999999999 28.0152999999998 -6.1005000000002
118.886599999999 23.6815999999998 -11.0180000000002
-0.77630000000173 0.241300000000304 -0.283700000000157
1.33329999999808 3.90360000000025 -0.094400000000214
39.2545999999988 11.8379000000001 11.5418999999998
61.2708999999989 20.6338 -0.13880000000022
57.3661999999989 23.3536000000001 -7.61440000000025
41.2836999999989 6.72660000000013 12.2954999999998
88.1392999999986 13.5915999999999 21.9837999999998
106.178999999999 16.2070999999998 18.8759999999998
112.413099999999 31.5755999999998 3.69099999999979
118.491299999999 18.3042999999998 13.1742999999998
115.112899999999 26.4125999999998 -2.69290000000018
-0.779500000001753 -1.06059999999971 -0.290700000000129
2.46169999999825 -3.8088999999997 0.303099999999882
40.869099999999 0.359900000000175 11.8021999999998
64.6465999999982 -1.67470000000009 0.0818999999998853
62.0362999999984 -4.49780000000003 -6.90460000000021
116.960899999999 4.19169999999982 3.76629999999984
118.808799999999 9.80949999999984 -2.63370000000017
ID=TAMmexUNKMEXAMNH207419

LM3=54
3.06512325789864 -4.11157380943702 -10.3264202226766
4.83419999999838 -1.63319999999965 -9.89170000000039
55.2015000000008 -2.5613999999992 -7.55320000000038
59.1063999999982 -1.78449999999979 -7.7447
49.7868999999988 8.20610000000036 -6.99710000000017
69.5053999999992 3.58620000000043 -7.6288
71.8573999999967 4.68699999999986 -7.28799999999987
76.5930999999987 -0.899199999999784 13.784
92.4849336754147 15.8009406714219 -12.7111498723799
83.7559999999976 6.78050000000007 -1.28989999999992
88.3912999999968 4.21279999999975 -5.37899999999984
97.4471999999997 -6.39029999999982 -4.3746
97.9175999999996 4.00240000000018 -10.8894
111.6086 -0.88379999999981 -6.40110000000002
118.8541 9.21340000000018 -10.0607
123.659 12.5038000000001 -8.6815
124.4775 20.4595000000001 -10.8899
129.6442 21.2168000000001 -0.313700000000005
127.195200000001 8.46110000000017 -4.81140000000003
130.3222 14.3610000000001 -10.4725
1.95869999999988 4.91270000000078 -10.0134000000004
3.33140000000018 3.20030000000085 -10.0182000000004
51.2197999999986 20.9170000000004 -7.35870000000012
55.1936999999978 21.7992000000002 -7.75460000000003
66.8100999999972 19.5821 -7.53139999999993
68.4287999999971 18.2321 -7.4640999999999
71.5903999999978 25.6079000000001 13.6963
81.0756999999975 21.9547 -1.1915999999999
85.1797999999976 26.5731 -5.94889999999991
90.5803999999983 38.0817 -3.79519999999996
94.4574999999984 28.499 -10.3210999999999

105.754099999999 37.0833999999999 -6.24219999999996
116.211999999999 29.2572 -9.50619999999997
121.3405 27.1255000000001 -8.27209999999999
123.3801 33.1165000000001 -5.21009999999999
127.2363 27.5033000000001 -10.9975
-0.502399999999911 0.391200000000825 0.0461999999997043
0.114500000000358 4.0522000000009 -0.436100000000355
39.8897000000003 14.0291000000008 11.8174999999997
64.7360999999974 23.8987000000001 -1.70209999999999
53.4392999999978 23.8898000000002 -8.69280000000003
42.7821000000004 7.52020000000076 14.9441999999997
92.9373999999988 15.6928000000001 23.4201
113.8923 18.7241000000001 20.2701
117.299299999999 37.7791000000001 3.92660000000002
127.5406 20.6895000000001 14.2138
124.973 31.4729 -1.78630000000001
0.282600000000153 -0.582799999999145 0.178399999999725
1.76930000000053 -4.19959999999914 -0.232800000000247
42.450800000001 0.42120000000083 12.2592999999997
68.7973999999985 -2.04039999999977 -2.6814
58.7764999999996 -3.98109999999959 -8.46330000000011
122.755500000001 1.40220000000021 3.51249999999997
127.777100000001 9.38450000000015 -2.26610000000002
ID=TAMmexUNKCOLAMNH207929

LM3=54
3.4496 -3.0493 -9.1281
5.0059 -1.0939 -8.5161
49.7311 -4.0158 -5.0737
57.2069 -1.8274 -6.5367
46.9402 7.3696 -4.3112
64.386 3.4332 -5.8277
66.3688 4.2685 -5.791
67.7584 -1.3475 11.6612
83.7731 14.8875 -10.7463
77.4135 8.2719 -1.229
80.9801 4.2338 -6.296
87.8148 -5.2179 -4.5193
89.6644 5.0024 -9.5916
101.3662 0.2618 -5.5778
108.9757 9.379 -8.0178
112.7614 11.3225 -8.3487
113.3564 19.0823 -10.1748
118.949 19.7179 -0.5924
115.1805 8.2133 -4.8404
117.8638 14.1244 -10.3893
2.5604 5.535 -9.8704
4.6828 3.9166 -9.2563
45.8254 19.7022 -5.5977
54.7365 20.3284 -7
62.4347 17.9914 -5.7959
65.0135 16.9227 -5.6133
64.7162 23.2101 11.5736
75.6698 19.4716 -1.3624

76.3946 23.0345 -6.2221
81.8216 34.433 -3.869
86.1849 25.557 -10.0624
96.3498 33.2031 -5.8911
106.1123 26.902 -7.9595
110.3494 25.5459 -8.261
111.9164 30.255 -4.8656
115.4216 24.5447 -10.2831
-0.1095 0.8383 0.2779
0.7419 4.1867 -0.6604
37.2937 11.9087 10.6326
59.9774 21.4276 -1.0918
54.6234 23.7466 -9.6356
40.7035 6.9015 12.3049
84.74 13.8536 21.8737
101.9439 16.9391 18.7974
106.4194 34.4344 2.8029
115.9959 19.4019 12.7177
113.2232 27.6231 -0.9901
-0.004 -0.4166 0.1278
1.823 -3.5401 -0.9858
39.6823 1.2987 10.7031
63.3594 -1.2321 -1.4918
59.4574 -5.1759 -8.9625
111.746 2.1959 2.9124
115.5922 10.8145 -1.956
ID=TAMmexUNKCOLAMNH23416*

LM3=54
5.26249999999807 -3.89650000000028 -9.98919999999983
6.48649999999937 -1.18529999999976 -9.13819999999986
55.5420999999991 -2.22430000000012 -5.4297000000002
60.8967000000006 -0.897499999999869 -5.77380000000012
52.2545999999991 7.93910000000005 -4.96890000000024
69.0119000000021 3.73890000000045 -4.75150000000019
70.7874000000004 5.56570000000005 -5.17730000000022
74.3545000000005 -0.931599999999914 13.4641999999998
87.2883999999999 14.5124000000001 -10.3552000000001
82.5074000000007 7.13370000000022 -0.408800000000112
85.0934999999998 4.78380000000001 -4.80760000000011
95.3767000000001 -4.81409999999996 -3.80950000000012
95.6357000000004 4.73320000000016 -9.23130000000012
109.0826 0.221900000000107 -5.69270000000014
116.556588257148 8.99115065318906 -8.12831561018205
119.5372 11.1404000000001 -7.43680000000016
119.9171 18.7284000000001 -10.1112000000002
125.3437 20.2823000000001 3.5403999999998
122.936111897039 7.35679712944623 -4.31252186220993
125.429793987356 13.3476006276751 -9.22690164451404
3.47929999999912 5.28340000000006 -9.54369999999999
5.78299999999961 3.28180000000013 -9.29239999999997
51.8026999999997 20.7612000000001 -5.18280000000017
58.3952 20.8966000000002 -5.84860000000018
66.5111000000004 18.6489000000002 -4.8921000000002

68.6743000000003 18.0727000000002 -5.2081000000002
70.1013000000004 24.4431000000002 13.0399999999998
80.1114679296536 19.4411011214731 -0.954028997661031
82.5289000000003 23.0245000000002 -4.98520000000017
88.7623999999999 35.4498000000002 -3.68170000000018
91.9710999999999 25.9623000000002 -9.41840000000014
104.8087 34.4423000000003 -5.64260000000019
113.9481 28.0888000000002 -7.69810000000018
117.4972 26.0624000000002 -7.12330000000019
119.62203437996 30.9607398536255 -3.9752889049594
123.517780265137 25.894591106098 -8.85345979180248
-0.749200000001138 0.490199999999926 -0.438199999999916
0.752699999999208 4.29700000000001 -0.0816999999999836
39.7941000000002 12.7286000000002 11.7523999999999
64.7528000000002 23.8352000000002 -0.372200000000188
59.5804 25.1062000000002 -8.47670000000016
43.2246000000002 7.18310000000011 13.6762999999999
89.6559000000004 14.8512000000002 22.0982999999998
110.7808 17.7376000000002 19.0440999999998
114.9175 34.6867000000002 2.4240999999998
124.486 19.0124000000002 13.3065999999998
120.8012 29.0147000000002 -0.743600000000212
-0.567700000001235 -0.797100000000098 -0.247299999999908
1.81739999999918 -4.08560000000008 0.10190000000012
41.2689000000002 0.22460000000009 12.0871999999999
68.3611000000008 -1.82759999999987 0.519499999999844
63.521994370222 -4.40648556144578 -8.54363122010997
119.2474 2.39730000000011 3.30989999999986
123.4319 9.35700000000011 -0.834400000000184
ID=TAMmexFEMNICAMNH28975*

LM3=54
5.54459999999708 -2.32510000000071 -9.10520000000046
6.39429999999727 0.0827999999992399 -8.42640000000035
54.5573999999981 -2.4530000000006 -4.94490000000031
60.5405999999981 -0.960600000000621 -5.9402000000003
50.0648999999984 7.87919999999951 -5.01840000000032
66.6831999999984 2.72309999999953 -5.21040000000022
68.3119999999985 4.9652999999995 -5.66510000000024
67.1366999999989 -1.25360000000041 13.3510999999997
86.0458999999989 13.4694999999996 -10.4857000000002
80.2174999999986 6.48579999999956 -1.14720000000023
81.7646999999986 3.47609999999953 -3.79380000000022
89.3207999999988 -5.12790000000047 -2.30390000000029
90.2978999999987 3.51349999999952 -7.71520000000026
103.108599999999 0.0114999999995971 -5.11410000000035
110.376099999999 9.03969999999958 -6.85590000000038
113.594599999999 10.9655999999996 -6.92380000000044
115.317899999999 17.3652999999996 -8.64620000000042
120.620499999999 18.2799999999997 0.954799999999529
116.623299999999 6.89619999999955 -3.90260000000047
119.320999999999 12.6200999999996 -9.53010000000047
4.26429999999735 5.07739999999937 -8.80590000000047
5.93359999999724 3.15449999999939 -8.13850000000045

51.4962999999982 19.8205999999995 -4.7604000000003
57.7347999999983 19.9538999999996 -5.50480000000026
65.1608999999985 18.2625999999996 -5.16350000000024
67.3393999999987 16.3732999999996 -5.50410000000024
66.456499999999 23.2705999999997 14.1583999999997
78.8257999999989 18.8071999999996 -0.651000000000246
79.7291999999989 22.5319999999996 -3.05040000000024
84.6161999999989 32.6772999999996 -2.9974000000003
87.9067999999988 24.5554999999995 -8.22060000000027
100.061699999999 31.8898999999997 -4.70660000000036
108.527999999999 25.3917999999997 -6.85440000000041
112.419499999999 23.4241999999997 -7.16130000000045
114.344799999999 29.1399999999997 -4.09450000000048
118.4838 24.0231999999997 -9.34270000000049
0.143699999997287 1.3260999999993 0.440899999999498
1.9475999999973 4.58639999999936 0.0955999999994888
39.4897999999983 11.3660999999995 12.6264999999996
61.0465999999987 22.1567999999996 -0.0650000000002657
58.2938999999983 23.3898999999995 -7.60310000000027
41.4094999999984 6.29989999999952 14.1324999999996
84.8308999999992 13.0146999999997 22.6780999999997
105.219699999999 15.7837999999997 19.7050999999996
107.9686 33.0069999999997 3.95629999999953
118.7673 18.1669999999997 12.9559999999995
115.088099999999 26.4160999999997 -0.560400000000468
0.024699999997439 -0.277100000000792 0.279399999999477
2.75539999999741 -2.8840000000006 0.311699999999512
40.0646999999984 1.41539999999954 11.8605999999997
64.7985999999983 -1.83730000000053 -0.684200000000255
62.3046999999983 -4.28280000000056 -7.80750000000026
112.260299999999 1.28799999999964 3.24019999999957
117.269799999999 9.52779999999966 -0.704900000000463
ID=TAMmexFEMPANAMNH36704*

LM3=54
4.09789999999937 -3.31200000000171 -9.0169000000002
5.60870000000794 -0.910100000000023 -8.44800000000054
53.2054999999992 -2.37930000000096 -3.80640000000011
58.5666000000018 -1.16850000000038 -4.82530000000014
48.6925000000011 8.01969999999951 -3.5079000000003
65.9624000000027 3.45059999999962 -3.92650000000019
67.1404000000042 5.42410000000012 -4.17820000000018
69.9867000000017 -0.927500000000352 13.2261999999999
85.2914000000019 13.6838 -8.41780000000002
78.9206000000037 6.88189999999996 0.488399999999871
85.5084000000023 3.89219999999991 -4.6291000000001
91.0893000000018 -5.07440000000013 -3.09720000000008
92.5018000000034 5.01840000000006 -7.35740000000008
103.847500000002 -0.707000000000146 -4.91609999999999
111.445700000002 9.03709999999996 -6.48969999999997
115.161200000001 10.8750999999999 -5.64379999999995
116.095300000002 18.6028999999999 -8.75029999999994
122.372200000001 19.7599999999999 2.02610000000003
118.109600000001 7.31919999999985 -3.93279999999995

121.221000000001 12.6930999999998 -8.02819999999994
2.62090000000382 4.92189999999929 -8.91660000000029
4.67540000000506 3.41089999999955 -8.37110000000036
48.974000000002 18.8671999999996 -3.75830000000024
55.4089000000023 19.7813999999997 -4.7866000000002
63.7724000000029 17.7277999999998 -3.80600000000018
65.2420000000031 15.9911999999999 -3.75170000000016
66.3613000000024 22.2626999999998 13.9291999999998
76.7259000000028 18.5929999999999 0.82709999999988
82.2172000000025 22.7509999999999 -4.68700000000007
84.6857000000023 33.3615999999999 -3.04230000000007
88.835400000002 24.0154999999999 -7.20710000000003
97.912100000002 33.7478 -4.60410000000001
108.046700000002 26.8051 -5.99379999999997
112.583400000002 25.4743 -6.06059999999997
113.888000000002 30.7281999999999 -4.39439999999997
117.976100000001 26.0882999999999 -8.30369999999995
-0.767799999997868 0.586199999998977 -0.263600000000219
1.11220000000282 4.11859999999912 0.0718999999997387
37.6463000000013 11.5514999999994 11.5528999999998
60.9963000000026 22.1370999999998 2.1987999999998
56.7012000000026 23.2949999999998 -6.62150000000019
39.788900000001 6.21069999999938 13.8361999999998
86.627800000002 13.8632999999998 23.7597999999999
107.630400000002 17.3275999999998 19.2736
109.256500000002 34.8048999999999 3.1325
121.092400000001 20.0236999999998 12.6803
116.701600000001 28.3572999999999 -0.766299999999982
-0.556399999998055 -0.776100000001069 -0.209700000000248
2.15850000000194 -3.45250000000106 -0.238100000000206
39.2845000000006 0.555699999999282 11.3856999999998
64.3413000000017 -1.69570000000037 2.03599999999984
60.7796000000014 -3.57440000000039 -6.76210000000008
114.415300000002 2.19569999999984 3.21980000000001
119.475600000001 10.9654999999999 -0.57899999999996
ID=TAMmexMALPANAMNH37630*

LM3=54
4.30410000000322 -2.12949999999972 -8.80600000000034
5.86328350352518 -0.323695684738329 -8.2277756190621
49.2416 -1.35159999999991 -3.94059999999992
56.415499999999 -1.17370000000001 -4.45599999999993
45.4246999999993 8.04040000000009 -3.03059999999995
64.166100000001 4.49460000000008 -3.66949999999994
67.3700000000001 5.24750000000009 -3.68149999999988
66.3949000000002 -1.20629999999992 13.0887000000001
81.316600000001 13.5280000000002 -9.64639999999994
76.0939000000014 7.62510000000018 -0.133299999999963
79.4845000000005 4.77680000000018 -3.20899999999994
87.8053000000006 -3.9025999999999 -2.61419999999992
87.1270000000011 4.14210000000016 -7.24039999999995
99.6006000000017 -0.078299999999837 -5.0463
107.575100000002 8.1828000000002 -7.55909999999996
112.681300000002 10.6067000000003 -8.19339999999998

112.400400000002 18.7873000000003 -10.4463
119.263800000002 19.6997000000002 0.995100000000043
115.818400000002 5.63900000000026 -4.90189999999997
117.537500000003 11.7136000000003 -9.87449999999999
3.05630000000095 3.7568000000004 -8.29469999999996
5.24788168275991 2.72492940518155 -8.01624781715341
45.3669999999994 18.2421 -3.1257999999999
52.9787999999995 19.4716 -3.74209999999987
61.4019999999998 17.2396000000001 -3.07529999999988
64.5831 16.8337000000001 -3.43479999999988
64.4064000000003 22.6657000000001 14.0365000000001
74.2053000000006 17.6927000000001 0.482200000000094
76.6063000000008 21.2231000000002 -3.17009999999991
81.6829000000011 31.8584000000002 -2.21559999999992
83.9872000000013 24.2475000000002 -7.12199999999994
95.4587000000017 33.0131000000002 -4.28459999999994
103.936200000002 27.5322000000002 -7.47759999999994
109.583100000002 26.1119000000002 -8.14859999999996
111.018500000002 32.4157000000003 -4.77319999999995
114.159100000002 26.6118000000003 -10.6369
-0.0347999999980608 0.310500000000468 0.0989999999999293
1.1474000000014 4.00010000000044 0.382799999999977
35.5586999999997 10.8715000000001 11.5954
56.7642999999998 21.0523000000001 1.38290000000013
53.1961999999998 22.6368000000001 -5.1124999999999
36.7886999999998 5.81920000000008 12.9258
81.3086000000009 13.3384000000002 22.3965
102.313400000002 16.9510000000002 18.7723
107.778800000002 34.1282000000003 1.94470000000006
115.279300000002 18.8334000000002 14.3792
113.009600000002 29.2357000000003 -1.28819999999995
0.0742000000019974 -0.196899999999532 0.231299999999913
2.36120000000198 -3.34889999999956 0.331599999999882
37.6929999999999 1.25280000000009 11.5639
61.5002000000002 -0.928499999999905 -0.0270999999999576
58.1780999999999 -3.39559999999999 -6.50259999999989
112.799700000002 1.69190000000021 1.68860000000002
116.580900000002 8.35690000000023 -1.53349999999998
ID=TAMmexMALECUAMNH62867*

LM3=54
4.21519999999582 -3.32449999999999 -10.4196999999997
5.94260000000224 -0.670499999999242 -9.58920000000049
54.4091999999987 -2.88229999999987 -5.77010000000002
61.9165999999998 -1.4441999999999 -6.2905999999998
49.6964000000006 8.05160000000024 -4.60280000000015
67.8547999999988 3.8925999999998 -4.82019999999977
70.0904000000005 5.45700000000011 -4.80219999999989
74.6287000000005 -1.21909999999989 14.9653000000002
91.1079999999998 15.2184999999999 -11.6601999999998
82.4997000000003 7.56749999999993 0.879500000000216
85.5084000000008 4.50219999999995 -3.93919999999978
93.713499999999 -5.7444000000002 -3.32699999999973
95.5988999999998 4.07499999999985 -8.52979999999972

107.604099999999 -0.240300000000214 -5.56279999999972
115.1294 8.97759999999983 -8.50039999999969
119.989099999999 11.2582999999997 -7.32639999999965
121.045999999999 19.0037999999997 -10.1283999999996
126.581799999999 20.0918999999997 -0.702399999999634
124.043099999999 6.4833999999997 -4.41709999999965
126.010599999999 12.9496999999997 -9.64109999999964
2.7484 4.89210000000039 -10.0872000000001
5.24000000000073 2.96860000000045 -9.38290000000022
50.2555000000006 20.9465000000002 -4.74369999999999
57.7545000000005 22.1604000000001 -5.67599999999993
64.8348000000004 18.8165000000001 -4.17409999999986
67.1468000000005 18.7906000000001 -4.48089999999986
68.3174000000008 24.8522000000001 15.2118000000001
79.7980000000006 20.648 0.901300000000177
82.7152000000003 24.3672999999999 -3.72559999999979
87.0996 35.9318999999999 -2.93739999999978
91.6913999999997 27.4372999999998 -8.82949999999976
103.245 35.2176999999999 -5.33949999999972
112.485099999999 28.8086999999998 -7.94579999999966
117.694099999999 26.9408999999998 -7.43469999999965
119.873099999999 32.6047999999998 -4.79039999999963
123.862699999999 26.3518999999998 -10.3767999999996
-0.964500000001346 0.559400000000294 -0.700100000000028
1.49379999999929 4.16460000000036 0.190199999999888
39.9685000000003 14.1416000000002 13.0406
64.8166000000007 22.6165000000001 0.558600000000104
59.2346000000005 27.0419000000001 -8.76119999999992
43.2084 7.47560000000019 15.0091
88.4258000000003 15.4169 23.8745000000002
110.1181 18.6293999999999 20.3480000000003
113.507699999999 36.7497999999998 3.04540000000034
123.758199999999 20.0841999999998 14.1360000000004
120.712199999999 29.1490999999998 -0.973199999999663
-0.819300000001523 -1.05999999999971 -0.706700000000037
3.11709999999827 -3.70839999999975 -0.246900000000053
41.6634999999997 -0.86859999999981 12.5838
68.4953000000002 -0.28769999999994 0.0497000000001354
64.5713000000001 -5.11069999999989 -9.64979999999995
118.549699999999 1.06159999999975 3.23010000000034
122.722999999999 9.73049999999972 -0.427699999999633
ID=TALmexMALECUAMNH62868*

LM3=54
6.199600000001 -2.42850000000104 -10.3277999999998
7.21120000000293 -0.548200000000361 -9.61599999999927
54.8151000000009 0.445199999999712 -2.98249999999974
61.3279000000018 1.95519999999986 -3.6408999999999
48.1456000000004 10.5463999999998 -2.68249999999976
67.3461000000012 7.57999999999986 -1.52989999999998
69.5432000000012 9.42689999999993 -2.13420000000013
72.7093000000007 3.23229999999963 16.2874
87.8660000000005 20.2435999999996 -7.21539999999993
80.8708000000017 12.0890999999999 3.30029999999996

86.3860000000011 10.2333999999997 -2.62730000000004
94.3807000000008 0.0255999999995984 -0.125399999999948
93.5922000000013 11.2492999999997 -5.14189999999999
108.847200000001 7.06309999999959 -2.03469999999997
115.3788 16.4423999999996 -4.25369999999996
118.251700000001 21.0499999999996 -4.51579999999996
119.0787 28.5700999999996 -7.82029999999995
124.633400000001 29.8357999999995 2.29640000000004
123.232 18.4296999999996 -2.48439999999993
125.4713 24.1160999999995 -7.97199999999992
4.52130000000322 4.8281999999999 -10.2906999999997
6.3865000000034 3.55799999999994 -9.29109999999967
50.135200000001 23.5229999999998 -3.53109999999985
56.7777000000009 24.2236999999998 -3.8212999999999
64.1910000000009 21.6080999999998 -1.93369999999997
66.774800000001 20.9945999999998 -2.08219999999999
68.102400000001 30.1586999999998 16.7091
77.7712000000009 24.3753999999998 2.52159999999999
81.2839000000009 28.3048999999998 -2.34359999999998
83.8183000000007 41.7506999999997 0.179200000000038
88.3223000000006 30.8255999999997 -5.25479999999996
100.211200000001 42.6161999999997 -1.35779999999997
109.1151 37.7785999999995 -4.35289999999996
114.6627 34.4249999999995 -4.66509999999996
115.956700000001 39.0509999999996 -2.49299999999997
122.8486 36.4200999999995 -7.67039999999994
-1.2400999999977 0.260399999999561 -0.459199999999642
0.302800000002876 4.41379999999977 -0.0556999999996627
37.2750000000016 17.1137999999998 12.5423000000002
62.688600000001 26.5917999999999 2.69150000000006
58.0020000000009 28.9260999999998 -6.65709999999993
40.8970000000015 9.78719999999978 17.4953000000002
89.2347000000011 21.2108999999997 27.1746000000001
110.315800000001 26.3943999999996 22.9074
111.479300000001 44.1585999999995 5.22690000000004
124.390800000001 30.0982999999996 13.0142000000001
118.686900000001 36.9995999999996 -0.732199999999955
-0.679099999997794 -0.692400000000519 -0.226899999999623
2.27000000000194 -3.66340000000058 0.210700000000383
40.5148000000015 1.64169999999972 13.1695000000002
67.925800000001 2.77879999999974 3.26690000000001
64.5923999999997 -1.15760000000048 -6.66659999999988
119.461600000001 11.1290999999996 6.01050000000006
122.531400000001 20.7776999999996 0.327700000000046
ID=TAMmexMALMEXUSNM073502

LM3=54
4.5219 -2.4056 -9.0804
6.0371 -0.3351 -8.4441
51.1849 1.0011 -1.1739
59.6751 2.7508 -1.8125
46.2468 10.4277 -1.7043
66.0199 8.3708 -0.4514
67.738 9.7112 -1.2298

72.6019 3.2563 16.5496
89.5217 18.9492 -6.8327
81.4371 11.7159 3.2364
85.0854 9.0559 -1.1315
93.5323 0.2797 -0.3972
92.4053 10.5571 -5.479
106.7698 5.8823 -2.1295
114.0425 14.4336 -3.0243
118.4301 18.0301 -3.7091
117.9644 24.3195 -6.2767
123.3974 25.4975 3.5078
121.3704 14.6356 -2.0189
124.2487 20.1081 -6.5787
3.0683 4.6298 -8.9885
4.9605 3.2505 -8.4924
47.9628 21.0847 -1.2802
55.183 22.2843 -1.6393
63.0694 19.6452 -0.6492
65.5591 19.6566 -1.5293
68.0922 26.8763 15.6587
78.9591 22.6096 3.4639
81.0652 26.6566 -1.3644
86.1176 38.1673 -0.4367
89.7644 28.9528 -5.4872
101.8419 38.7029 -1.0423
110.6688 32.7799 -3.5557
116.0674 31.1728 -3.9144
117.8875 34.9746 -1.8569
122.4991 30.8456 -6.3768
-0.4802 0.3295 -0.0315
0.1451 3.7243 -0.2976
37.5043 13.631 14.179
60.3886 24.6101 5.4591
55.1562 25.1454 -3.1825
37.8579 8.9455 16.44
88.496 18.7916 26.2335
106.7233 22.4826 22.1874
112.4521 39.4045 5.5071
119.6505 24.5409 16.9045
118.7035 32.7051 1.2412
-0.4803 -0.623 -0.2784
1.8512 -3.2114 -0.2008
40.3302 3.5364 14.1495
64.6902 2.1313 5.4781
60.8414 -0.3706 -3.2459
118.2782 7.9056 5.9357
121.414 16.4062 1.6795
ID=TAMmexFEMMEXUSNM076198

LM3=54
5.86259999999484 -2.84890000000131 -8.32669999999982
6.08750000000353 0.923200000000444 -7.90459999999999
52.0846999999965 -0.158100000000878 -2.23299999999994
58.9907999999996 1.4031999999999 -2.71879999999989

46.9633999999986 10.6502999999997 -3.19160000000009
65.5277000000007 6.61089999999993 -2.09569999999994
67.4466000000022 8.49810000000048 -2.48049999999989
69.4241999999994 2.64249999999986 16.6322000000001
89.5789000000006 19.3006000000002 -6.88179999999986
81.0301000000021 10.7264000000003 2.46410000000016
84.8088000000007 8.09940000000016 -1.47929999999988
95.1284000000002 0.311100000000053 -0.447899999999858
94.3457000000021 9.54110000000033 -5.21709999999983
107.331500000001 5.6818 -0.71529999999978
114.867900000001 14.5349000000001 -2.56379999999976
119.504 17.6367 -1.45099999999976
118.9908 25.2902 -5.49279999999974
124.5803 26.7994 5.27630000000026
122.118 14.8798999999999 1.17190000000026
125.9123 20.0555999999999 -4.92809999999974
4.74509999999948 4.87109999999963 -8.71079999999989
6.14620000000086 2.13549999999993 -8.18299999999995
47.4659999999994 21.2794999999999 -1.6668
54.4101000000001 22.589 -2.04019999999997
61.9855000000009 20.9012000000002 -1.41029999999994
64.7580000000012 19.4495000000003 -1.77959999999992
66.1176000000002 27.1363 15.2769000000001
77.2620000000011 22.7999000000002 2.89380000000012
79.8646000000009 26.8833000000002 -1.50799999999988
86.3100000000007 38.7266000000001 -0.329699999999849
89.3215000000006 29.2621000000001 -5.25329999999984
99.1937000000006 38.8483000000001 -1.2528999999998
110.157500000001 33.5122 -2.15099999999976
115.506100000001 33.4444000000001 -2.21319999999975
116.3141 36.7972 1.10190000000025
122.716 33.1815 -4.98729999999974
-0.527100000002428 0.614699999999203 -0.0512999999999063
1.49449999999836 4.04009999999941 0.290200000000095
36.9360999999982 13.8116999999997 13.6102
59.7693000000005 24.7353000000001 4.13220000000005
54.6025000000004 26.4185000000001 -3.91959999999996
34.8002999999979 7.82229999999958 15.7021
86.3287000000001 18.0272 25.2690000000002
105.282 22.6902999999999 23.7912000000002
109.6455 39.7184 7.58340000000023
120.5702 26.2870999999999 18.1600000000003
116.4924 35.1753999999999 3.07300000000025
-0.419200000002595 -0.651500000000831 -0.137999999999904
3.03279999999745 -2.88840000000082 0.699900000000099
38.8987999999974 2.77559999999946 13.1661
64.2291999999995 1.91889999999989 3.92170000000007
60.5334999999991 -2.04110000000015 -4.64009999999987
118.1594 10.0197999999999 8.09720000000025
121.3178 17.4380999999999 4.01270000000027
ID=TAMmexFEMMEXUSNM077867

LM3=54
4.48309999999918 -2.92960000000081 -9.40669999999959

5.48829999999898 -1.08800000000061 -8.68809999999966
47.3097999999998 0.192799999999254 -4.47769999999981
54.3928999999997 2.31459999999924 -4.46729999999986
42.3502 10.8540999999993 -3.82839999999979
59.8209999999999 7.81929999999929 -2.44899999999984
61.7822999999999 8.65399999999931 -2.72569999999983
64.3831999999994 1.92759999999927 14.1167000000001
79.4544999999997 19.6061999999995 -6.11739999999994
71.0987999999997 10.8704999999994 2.39170000000012
77.8613999999994 9.32679999999936 -2.28409999999992
85.7176999999989 0.0406999999993256 0.389700000000132
86.3448999999994 10.1697999999995 -4.74969999999989
99.1958999999983 5.83119999999932 0.305200000000025
105.706799999999 15.4395999999995 -1.17659999999988
109.630699999998 19.6892999999995 -0.807799999999997
109.246699999999 26.3791999999995 -3.23259999999997
115.025899999998 27.7022999999994 6.62000000000001
113.372499999998 15.2301999999994 2.60920000000005
115.299199999998 21.8299999999994 -3.28359999999999
2.91223220070792 4.91532757291421 -9.11463224863919
5.12993357894362 3.15389209878238 -8.48306146161698
41.9673000000003 21.8012999999994 -4.49289999999974
49.0376000000005 22.8509999999994 -4.40279999999978
56.3567000000004 21.4804999999994 -3.04039999999986
58.8132000000002 21.1093999999994 -2.71689999999988
57.9800000000002 27.3686999999995 13.7690000000001
67.8195000000002 22.7844999999995 2.38820000000016
72.8712000000001 26.8212999999995 -1.98429999999991
76.0709999999998 39.2970999999995 -0.0483999999998952
81.8667 30.1649999999996 -4.87979999999988
90.4602999999994 40.6747999999995 0.555800000000061
100.831599999999 34.5382999999995 -2.05669999999996
106.164199999999 32.7775999999995 -0.709499999999984
107.309199999999 38.2450999999995 2.78750000000004
111.663899999998 33.1186999999994 -3.57539999999994
-0.582600000000845 0.51089999999936 -0.487599999999659
0.426199999999107 3.96369999999934 -0.546399999999716
32.5669999999999 14.5901999999994 10.4534000000003
54.1561000000004 25.0096999999994 1.25830000000023
49.9596000000003 27.0826999999994 -7.30139999999979
33.5545999999999 8.1157999999994 13.1641000000003
77.5231999999993 18.7937999999994 25.9931000000002
97.1208999999984 23.7092999999994 23.6024
102.741499999999 41.0493999999995 10.1327
110.369499999998 26.8718999999994 19.3347000000001
108.491099999999 36.0232999999995 5.58570000000005
-0.00160000000083696 -1.07650000000065 -0.0147999999996609
2.02439999999909 -4.09450000000066 -0.687499999999656
35.5859999999997 1.87299999999933 10.8440000000003
59.5826999999997 2.48539999999927 1.37400000000018
57.3553999999994 -1.1441000000008 -7.38769999999984
109.806799999998 10.3253999999993 10.4929000000001
112.975799999998 17.9713999999994 5.75560000000006
ID=TAMmexUNKCOLUSNM123523

LM3=54
3.92559999999065 -2.95540000000182 -8.99259999999952
5.02889999999073 -0.339700000001546 -8.37779999999966
54.7971999999948 0.890299999998722 -0.935199999999602
64.5918999999954 3.23319999999878 -2.2378999999996
50.7497025347512 10.6900094763615 -0.496736371589746
70.9535999999962 8.90179999999889 -0.8874999999996
72.1020999999966 10.302799999999 -1.8391999999996
75.6009999999962 4.48829999999898 16.1414000000004
90.1766999999975 19.8320999999993 -7.31679999999958
84.5459854679262 12.955350042614 2.8899075053893
87.8138999999976 10.3524999999992 -0.751599999999601
96.4785999999974 0.259799999999234 0.15140000000044
95.5045999999978 11.9461999999993 -5.06559999999958
110.347899999998 6.02859999999934 -0.946799999999509
115.507599999998 14.7798999999995 -3.04859999999949
119.046199999998 17.4978999999995 -3.41369999999944
119.629599999998 25.2903999999995 -6.32349999999944
126.569099999997 26.2922999999995 1.72090000000065
122.054599999998 13.4855999999995 -1.62069999999942
126.848199999998 19.8046999999994 -5.83599999999939
2.45709999999104 4.46939999999846 -9.19849999999963
4.91069999999086 2.35649999999843 -8.06249999999967
51.0346999999948 21.7685999999988 -1.0828999999996
60.8601999999955 23.4126999999988 -2.17219999999959
67.5012999999961 21.6641999999989 -1.68789999999958
69.8156999999962 20.4855999999989 -1.78019999999958
71.5461999999962 27.614199999999 16.0620000000005
82.2962999999972 24.1393999999991 2.99960000000043
84.2061999999972 27.0829999999991 -0.23029999999956
88.7837999999969 39.4140999999992 -0.0417999999995029
92.5556999999973 29.0449999999992 -4.64309999999954
104.391599999997 40.0008999999994 -0.577199999999471
112.301499999998 33.4902999999995 -3.11909999999947
116.379299999998 32.1981999999995 -3.27799999999943
118.611799999998 37.3087999999995 -1.36279999999939
123.838699999997 32.5781999999994 -6.61589999999939
-0.852700000009545 0.521299999998403 -0.0875999999995745
0.700699999990627 3.97829999999853 -0.349499999999605
40.3637999999939 14.0262999999987 14.4386000000004
64.9536999999958 26.3063999999989 3.83750000000043
61.8089999999954 27.6434999999988 -5.2502999999996
40.6463999999941 8.87989999999873 15.4340000000005
89.8417999999966 19.4768999999991 26.8964000000005
113.181599999997 23.8893999999993 22.9650000000006
115.792499999997 40.6631999999994 5.3952000000006
125.662999999997 26.7113999999995 14.7731000000007
120.844699999998 34.2666999999995 0.761100000000615
-0.588800000009593 -1.19140000000158 -0.201499999999577
2.39239999999053 -3.75000000000153 0.109300000000406
42.3677999999939 3.62329999999872 14.4567000000004
69.7551999999959 2.72619999999882 4.10730000000041
67.6120999999953 0.486599999998748 -5.27639999999959

121.748499999998 10.0266999999994 5.84240000000059
123.900299999998 17.6400999999995 0.890100000000595
ID=TAMmexUNKGUAUSNM251157

LM3=54
2.0918 -3.8631 -10.3216
4.9349 -1.5048 -9.8202
49.7394 -4.062 -6.0695
55.8004 -1.9983 -6.42
46.7602 6.4986 -5.3308
63.3477 3.5909 -5.4618
66.2907 4.2362 -6.3292
70.896 -1.6616 10.7098
85.9424 13.307 -12.8896
77.1591 6.2006 -1.386
83.1385 3.8076 -6.1867
91.0153 -5.8382 -4.4631
90.5817 4.4318 -9.1343
105.167 -1.2591 -4.3807
111.4816 7.9895 -9.4799
115.5435 11.3715 -8.5776
115.8759 18.5273 -10.4275
121.715 19.5827 -1.3312
118.6532 7.7199 -5.0688
122.3049 13.7654 -11.0888
1.0217 4.1721 -10.3808
4.5004 2.4748 -9.6001
45.8381 18.69 -5.9175
52.3517 18.567 -5.9622
60.6331 16.2205 -5.3707
63.4185 16.0339 -6.0026
67.3716 22.9367 11.3803
75.2438 17.5965 -1.1402
78.5045 22.0068 -5.8674
83.7717 33.452 -4.474
86.8722 23.3797 -9.3563
98.6583 33.7556 -5.1157
108.0339 26.954 -8.8547
112.6889 25.3612 -7.8294
114.4557 29.8064 -4.7679
119.7083 25.3484 -10.6587
-1.4766 0.4646 -0.4237
0.1274 3.8026 -0.3183
37.5084 11.6254 11.2734
59.5301 20.8154 0.4757
53.2011 22.9373 -8.4399
37.8907 5.4596 11.8985
84.0239 12.6811 20.5808
106.2804 16.6894 18.081
108.6973 34.0136 2.2498
119.8369 19.1225 11.4289
115.6824 27.4149 -2.0704
-1.4453 -0.7908 -0.5903
0.9834 -3.2151 -0.6323

39.7491 -0.0494 11.0404
62.1755 -3.1078 0.1435
57.8573 -5.4939 -8.7493
114.2512 1.8533 1.4823
118.9361 10.6202 -2.5649
ID=TAMmexFEMCOLUSNM281322

LM3=54
3.3141 -3.5224 -9.6123
4.7646 -1.3619 -9.2387
49.0905 -0.8138 -4.2584
55.2178 2.2481 -4.6847
42.5733 9.6368 -4.623
62.3462 7.3312 -3.1887
64.6491 8.7317 -3.5247
67.196 3.0608 15.9607
83.4139 19.7558 -7.0313
75.119 11.7713 2.3213
79.616 9.5335 -1.9849
88.4534 -0.5657 -0.4362
89.1938 11.9834 -4.4836
101.6438 6.0311 -0.3029
107.4759 16.1159 -2.6336
111.7854 19.1978 -1.7884
111.6289 26.3163 -4.7738
116.5489 27.3919 4.752
114.7693 16.1515 0.5987
118.1336 22.0173 -4.1518
1.6344 4.7905 -10.2291
3.8921 2.7705 -9.5454
43.3124 22.0573 -5.0553
50.6238 22.0963 -5.1443
58.5823 20.5847 -2.9906
61.8824 20.672 -3.3485
62.7088 27.5719 15.5639
72.6185 22.6376 2.5373
75.7166 26.7943 -1.5617
78.8391 39.0747 -0.0261
84.6957 29.253 -4.1117
93.4433 39.9993 0.3643
103.1335 33.7188 -2.1123
108.6104 33.048 -1.0686
109.2914 36.6557 1.0261
115.8076 33.6536 -3.6318
-0.1521 0.3123 -0.0128
0.3248 2.3402 -1.9596
35.0828 14.2985 11.8561
55.4527 25.7301 2.1858
50.6261 26.3709 -7.3498
35.7301 8.7384 13.3607
81.8422 19.1434 27.6009
99.4274 23.5328 24.497
103.5615 40.6865 7.8934
114.6209 26.6852 17.483

110.3566 35.2466 3.5935
0.0043 -1.1221 -0.0549
1.6553 -3.9061 -1.297
38.0734 2.3972 12.3681
60.9135 0.8643 2.7678
56.9206 -1.7822 -6.7151
110.3119 8.9477 8.2891
114.5232 17.5708 3.3444
ID=TAMmexFEMCOLUSNM281344

LM3=54
5.93499999999986 -2.82210000000058 -9.41590000000003
7.72140000000189 -0.764700000000124 -7.96859999999971
55.6081000000001 0.763099999999942 -2.28059999999988
64.8245000000011 2.98840000000007 -2.79470000000002
53.6058999999998 10.8397 -2.25689999999994
72.0239000000003 8.81690000000003 -0.79600000000013
73.6954000000004 9.9848000000001 -0.970700000000249
77.3729999999997 2.52629999999982 17.0288999999999
94.1205000000002 19.1617999999998 -6.82320000000002
86.0480000000008 11.2307 3.87629999999988
88.6165000000004 9.22499999999988 -0.962600000000142
98.1460000000001 0.251899999999703 -0.0947000000000633
98.6191000000008 10.3717999999998 -4.67380000000005
111.8176 5.81399999999968 -0.919900000000038
118.6028 13.3926999999997 -2.5329
122.098900000001 16.7418999999997 -3.10300000000001
123.904700000001 24.2357999999997 -5.18319999999999
128.383400000001 25.3464999999996 4.16039999999999
125.985500000001 13.5116999999996 -0.124299999999995
130.507000000001 19.2710999999996 -4.37479999999999
4.69250000000229 4.86410000000014 -9.61679999999989
7.20410000000251 3.48280000000015 -8.1970999999999
51.7469000000006 21.208 -2.22230000000002
60.6071000000005 22.2995 -2.44960000000006
69.0559000000004 20.3447 -1.16730000000011
71.3921000000004 19.781 -0.952700000000128
72.2297000000004 26.4277999999999 16.7566999999999
83.4785000000003 22.4180999999999 3.73849999999989
85.4675000000005 26.0973999999999 -0.532700000000089
90.6632000000006 38.1116999999999 -0.0114000000000563
95.0645000000004 28.1932999999999 -4.43540000000004
105.888400000001 38.0285999999999 -1.06880000000003
115.814100000001 31.8920999999997 -3.60820000000001
119.811200000001 30.2630999999997 -3.37900000000002
122.078400000001 34.6302999999997 0.00859999999998706
127.316200000001 30.6652999999997 -5.27210000000001
-0.383599999998721 0.277099999999852 -0.220999999999867
1.33750000000188 4.20420000000002 0.190200000000091
41.3906000000009 14.0304 14.3664
66.6654000000005 23.6873 3.58339999999991
59.9946000000005 26.5498 -5.24470000000008
43.5472000000007 8.0285 16.9614
90.3660000000004 17.3304999999998 26.4756999999999

113.171300000001 22.4511999999997 22.7255999999999
117.900900000001 39.3134999999997 6.06219999999997
127.443100000001 24.8712999999996 14.8843
122.221600000001 34.1494999999997 1.56979999999999
0.067000000001164 -1.27010000000019 -0.474799999999842
2.82990000000086 -4.1839000000003 -0.658499999999852
44.0392000000006 1.74829999999995 13.4681
71.4409000000001 3.36939999999992 4.25099999999988
65.7866999999989 -0.943000000000227 -5.04820000000006
122.769100000001 8.73389999999962 5.60109999999999
125.748100000001 15.7676999999996 1.54379999999999
ID=TAMmexFEMNICUSNM337708

LM3=54
4.28437653167947 -2.72829795341261 -9.49226920926612
5.76849999999151 -0.40700000000123 -8.91000000000002
57.8331999999951 0.855799999999059 -0.450199999999947
61.2863999999956 2.6932999999991 -0.567699999999982
47.8467999999952 10.9468999999992 0.742600000000096
66.4872999999959 7.39339999999916 0.69810000000004
69.847899999996 9.20709999999913 0.581600000000053
76.0984999999958 3.91729999999916 18.8644000000001
88.3560999999971 19.2047999999993 -5.62700000000001
80.9945999999964 11.6026999999992 4.77020000000007
84.4156999999965 8.4672999999992 0.626100000000024
95.0868999999962 -0.0461000000008276 -0.556200000000034
93.492599999997 9.8813999999993 -4.27140000000004
108.494499999997 5.86049999999927 -0.669800000000058
114.307399999997 14.8151999999993 -3.05470000000015
118.893799999997 19.1875999999993 -3.97830000000014
118.338299999997 25.8430999999994 -6.13370000000016
126.202299999996 27.2255999999993 2.91309999999987
122.114899999997 14.3957999999992 -0.618800000000138
126.123199999996 21.1176999999993 -5.52410000000016
3.0114999999919 4.84909999999871 -9.42230000000006
5.09889999999215 3.06819999999874 -8.76460000000005
52.8576999999953 23.4776999999992 -0.0254999999999539
57.2212999999958 23.4492999999992 0.0275000000000727
64.0922999999961 21.4669999999992 0.733000000000072
67.2044999999961 20.6384999999992 0.637600000000094
69.942299999996 29.0995999999992 18.0281000000002
78.3315999999964 23.4299999999992 4.53700000000009
80.2515999999965 27.5302999999992 1.16440000000005
86.9312999999964 40.2411999999992 0.164300000000046
89.2362999999967 30.6357999999992 -4.5457
101.165799999997 40.1824999999993 -0.705200000000026
110.794899999997 34.3990999999994 -4.11400000000009
116.455499999997 32.6843999999994 -3.52710000000013
116.519899999997 38.0839999999994 -0.889200000000117
123.561299999997 33.3502999999993 -5.32010000000014
-0.801700000008378 0.638799999998703 0.0182999999999702
1.46319999999186 4.52269999999873 0.346299999999979
38.5702999999949 15.0835999999992 16.9456000000001
62.4938687709486 25.8081204270786 5.28129530630804

58.2049999999957 27.2253999999992 -2.55689999999994
41.3826999999949 9.34299999999918 18.5631000000001
89.4867999999961 19.3735999999992 27.2971000000001
111.863099999996 23.9676999999992 24.0881
113.715999999997 41.8740999999994 5.83369999999993
125.007899999996 27.1661999999992 15.8878999999999
119.364299999997 34.9743999999994 1.75859999999989
-0.538700000008367 -0.324700000001281 -0.0964000000000411
3.04949999999186 -3.18740000000125 -0.148400000000031
40.9511999999949 3.05179999999913 16.6246000000001
66.4613999999957 2.30439999999911 5.10100000000006
63.4549999999955 -0.428700000000918 -3.14140000000001
119.208099999996 9.69959999999919 6.1264999999999
122.725099999996 18.5118999999992 1.88209999999987
ID=TAMmexFEMPANUSNM339036

LM3=54
6.17749999999762 -4.41780000000059 -9.4318000000002
7.95329999999968 -1.5514000000002 -9.42850000000027
58.5179999999988 -3.4344000000001 -4.41529999999991
66.3825000000001 -1.39410000000003 -5.27660000000002
54.0617999999985 6.56939999999998 -4.2129
74.7424999999997 3.27640000000001 -3.59480000000003
77.8737999999997 4.87150000000006 -3.56710000000013
80.1090999999989 -2.04580000000016 14.7403
94.1932999999996 13.6906999999999 -9.61049999999973
88.7110000000004 6.39970000000003 0.778900000000054
91.7667000000001 3.60509999999996 -1.53789999999994
100.145599999999 -6.48480000000016 -3.72919999999979
102.6701 4.19829999999995 -7.67749999999978
116.2544 -0.764800000000129 -5.22099999999968
123.9379 7.52759999999989 -6.48009999999959
129.0845 11.1013999999999 -7.0889999999996
129.6206 18.3228 -9.94189999999958
135.569999999999 19.4528999999999 -0.900599999999602
133.2184 7.37529999999988 -4.89729999999959
136.933799999999 13.3572999999999 -10.4192999999996
4.94109999999985 5.08849999999994 -10.3463000000004
7.11590000000015 2.35329999999993 -9.57990000000044
54.8606999999992 18.9191999999999 -5.05750000000011
63.1141999999993 19.421 -6.05180000000008
72.3428999999995 16.8132 -4.36170000000003
76.6932999999996 16.6398 -3.87140000000005
77.5895999999993 23.9113999999999 12.8792
87.5473999999996 16.1703 0.674300000000082
89.8740999999998 21.2348 -3.15449999999987
94.2422999999996 33.1701 -5.05199999999983
100.0383 22.8321 -7.81779999999977
110.8083 32.2747000000001 -5.4601999999997
120.955099999999 26.4705 -7.35629999999962
126.84 24.6947 -7.68449999999961
128.8419 29.5058 -5.25709999999964
134.558099999999 24.4270999999999 -10.6288999999996
-0.635400000001337 0.316599999999662 -0.0957000000003536

1.49179999999931 4.11369999999981 0.178399999999582
41.6012999999993 11.8133999999999 13.1271999999998
70.1821999999995 21.6772 -1.13010000000006
64.9227999999993 23.9819 -8.61600000000008
43.1437999999991 6.11209999999988 15.0080999999998
97.3730999999994 13.4539999999999 22.9793000000002
118.124599999999 15.7841999999999 19.4408000000003
123.041799999999 32.7556 3.28620000000033
133.081599999999 18.8602999999999 12.8654000000004
130.966499999999 26.1904 -1.21439999999962
-0.439600000001571 -1.00190000000038 0.137999999999688
2.54659999999819 -4.29800000000048 0.654799999999721
43.6798999999991 0.0165999999998417 13.5717999999998
72.9621999999993 -1.71850000000009 0.124599999999944
68.3993999999978 -5.83160000000022 -7.77329999999996
126.785499999999 1.82549999999986 3.66660000000037
133.1102 9.8149999999999 -1.21359999999959
ID=TAMmexFEMPANUSNM362297

LM3=54
5.0894000000021 -3.54259999999935 -8.77510000000012
5.92310000000177 -0.71699999999917 -8.63050000000008
48.1090000000023 0.442700000000607 -2.425
55.8005000000014 2.84020000000041 -2.36719999999997
45.0351 11.0055000000001 -1.62929999999993
62.8209000000023 8.22920000000035 -0.634000000000039
64.9636000000004 9.22840000000001 -1.02959999999986
68.4122999999998 2.96650000000005 17.2033000000001
79.3965000000004 18.9198 -6.41939999999991
74.5025000000012 11.5079000000003 4.76570000000008
78.4151000000011 8.76740000000031 0.735300000000041
87.7755000000005 -0.0444999999998695 0.821300000000099
87.0723000000009 10.0391000000002 -4.78439999999989
99.8644000000007 6.22160000000018 -1.51409999999989
106.211300000001 15.7002000000002 -4.19319999999988
110.584400000001 19.2162000000003 -4.69149999999987
109.279700000001 25.9921000000002 -7.53989999999987
117.1845 27.6487000000002 2.45300000000015
114.375000000001 15.5528000000002 -1.45779999999985
116.568214336421 21.5656286535577 -7.60787195405186
3.23830000000037 4.05520000000044 -9.04410000000006
4.86010000000045 2.79610000000044 -8.68160000000007
43.5770999999995 21.9088 -2.29809999999991
50.9312999999998 23.2882 -2.64879999999991
59.2696000000002 21.3926 -1.23369999999991
61.3486000000002 21.9189 -1.5915999999999
62.0909999999995 28.1031 18.0809000000001
71.7922000000001 23.502 4.62850000000011
73.8666000000001 28.1148 -0.241499999999881
78.6791999999997 40.0554999999999 -0.306499999999871
82.8988 30.9962 -4.77309999999989
92.6578999999999 39.9285 -1.38449999999987
102.4563 33.9904 -3.79339999999986
107.2972 33.0337000000001 -4.71199999999987

109.1229 37.8157000000002 -1.53639999999986
114.3602 33.5704000000002 -7.53809999999986
-0.312899999999087 0.533900000000623 0.137399999999927
0.975800000000377 4.09610000000049 -0.00010000000006577
33.5039999999991 15.9728 13.7363000000001
54.0070999999997 26.6741 3.3425000000001
51.5509999999997 27.2694 -5.3010999999999
35.2466999999994 8.69270000000008 15.8294000000001
81.6221999999997 18.8647000000001 27.9456000000001
104.274 24.6320000000002 22.2246000000002
105.1024 41.3573000000001 5.59430000000014
115.8765 27.3723000000002 14.6020000000001
111.0213 35.0337000000002 1.28540000000014
-0.0390999999989536 -0.73419999999933 -0.114000000000068
2.65300000000123 -3.72789999999927 0.315799999999915
36.9684999999998 1.36480000000016 13.6106
59.8450000000002 1.32300000000007 3.63390000000005
58.1541000000011 0.0562000000002358 -4.74719999999992
111.168400000001 9.39990000000025 5.90140000000012
114.533000000001 17.7959000000002 0.536200000000139
ID=TAMmexFEMVENUSNM443249

LM3=54
2.71090000000708 -3.71559999999826 -10.3224000000003
4.34340000000717 -1.05609999999834 -9.61980000000006
51.1516000000043 -0.229899999999335 -3.95910000000016
57.6717000000038 2.20960000000053 -4.0976000000002
46.5570000000043 10.2425000000006 -3.27760000000015
64.7370000000033 7.60770000000044 -2.22340000000019
67.625800000003 8.6025000000004 -2.1745000000002
67.8833000000033 2.46430000000056 15.5316999999998
82.7108000000015 18.7811000000003 -6.20040000000024
77.3004000000023 10.4656000000003 3.17949999999976
79.9314000000024 8.33140000000045 -0.148000000000243
89.5493000000028 0.111900000000657 -0.281200000000301
89.9876000000019 10.5882000000005 -4.85510000000028
102.717000000002 6.2602000000008 -0.81490000000035
109.199200000002 16.0233000000008 -3.10440000000033
112.535000000001 18.8007000000009 -3.62590000000036
112.531300000001 24.4588000000009 -5.60900000000036
118.630500000001 27.647200000001 3.60739999999959
116.709800000002 15.600800000001 0.386499999999615
118.896496891589 20.9265042000863 -5.70086703058283
1.37980000000705 4.26060000000182 -10.6116000000002
3.58090000000706 2.5769000000018 -9.72750000000016
45.7115000000041 22.2385000000006 -3.65530000000013
52.9420000000033 22.8762000000004 -3.99300000000014
61.7360000000029 20.7900000000003 -2.65080000000017
65.0751000000027 21.2064000000003 -2.43500000000018
63.479700000003 27.7761000000005 15.2876999999998
74.021300000002 23.3573000000002 3.33279999999978
76.3205000000015 27.2112000000002 -0.257000000000238
81.2560000000018 38.4647000000004 -0.0416000000002452
86.2205000000014 29.1730000000003 -5.01120000000026

96.1200000000011 38.5079000000006 -0.0275000000003003
105.972900000001 33.1206000000007 -3.58080000000032
109.488600000001 32.3574000000008 -3.21310000000036
111.411200000001 36.1689000000009 0.527699999999642
116.101283344293 32.0490082125367 -5.43577045146745
-1.1170999999925 0.354500000001901 -0.269100000000151
-0.38069999999259 3.62300000000187 -0.450800000000159
36.8961000000049 14.7725000000008 13.4247999999999
58.898800000003 25.1841000000003 2.80169999999985
53.5293000000033 26.4448000000004 -6.90240000000014
42.322300000005 9.5655000000087 15.7564999999999
83.6403000000028 18.5129000000007 26.1956999999997
103.176500000002 23.0915000000008 23.8597999999996
105.017800000002 40.7546000000009 7.27989999999966
115.589800000002 26.2475000000011 17.3508999999996
112.178200000001 34.7876000000009 2.98039999999962
-0.738099999992495 -0.719499999998041 -0.0317000000001812
1.31440000000742 -3.13329999999811 -0.0135000000001256
39.0067000000053 2.37090000000093 13.2932999999999
62.8993000000035 1.7820000000005 3.56929999999979
60.296700000004 -0.636599999999387 -6.50450000000022
111.931900000002 8.19040000000098 8.0156999999996
116.156300000002 17.783700000001 3.94749999999961
ID=TAMmexFEMVENUSNM443250

LM3=54
6.92740000000514 -3.31979999999968 -9.38460000000085
8.66840000000503 -0.408199999998737 -8.02630000000098
55.4391000000021 0.380699999999868 -0.832900000000464
63.2650000000019 2.00159999999988 -1.44780000000044
52.8969000000025 11.2951 -0.839900000000447
70.5265000000012 7.36969999999975 0.287299999999591
74.5642000000009 8.57559999999969 -0.106200000000402
78.9073000000017 3.25969999999987 20.2858999999995
92.6821000000001 20.0755999999995 -7.14870000000034
86.8188000000007 11.6841999999997 4.45039999999963
90.7246000000006 9.53459999999963 0.505099999999628
99.4504000000005 0.209199999999649 0.20289999999959
100.4642 10.4934999999996 -4.10190000000037
114.6711 6.55329999999955 -1.88210000000043
122.2921 16.4099999999996 -5.05650000000042
126.4306 19.3613999999996 -4.76930000000046
125.0507 26.8078999999995 -7.92660000000047
133.3396 22.4927999999997 -8.61170000000056
131.653 29.0134999999996 0.348099999999483
129.4082 16.0460999999996 -3.11290000000052
5.05600000000529 5.75150000000074 -9.3267000000009
7.59950000000474 4.08940000000058 -8.25700000000088
50.4670000000026 22.4814000000001 -1.17570000000049
58.6280000000021 23.3254999999999 -1.35080000000044
67.3336000000017 21.7451999999998 -0.000800000000419576
71.8783000000017 22.2014999999999 -0.524600000000408
74.3188000000017 29.2463999999998 18.3978999999995
84.0116000000011 23.8216999999997 4.40189999999962

85.9473000000008 28.4973999999997 0.284799999999629
90.0645000000009 40.1427999999996 -0.327600000000416
94.8552000000002 30.5024999999995 -5.12520000000037
106.3534 41.0954999999996 -2.20810000000043
117.3683 35.5667999999995 -5.24930000000043
123.0351 34.2198999999996 -5.29030000000047
124.3389 39.1212999999996 -3.69270000000052
129.7546 35.1170999999996 -9.43980000000052
-0.56299999999479 0.111700000000504 -0.166300000000961
1.74200000000535 4.45910000000065 0.441499999999123
43.4295000000037 15.6542000000002 15.9301999999995
66.1541000000019 27.4449999999999 5.99389999999958
59.787400000002 27.9201999999999 -3.97970000000046
45.8765000000037 9.49040000000025 19.3164999999995
96.2585000000016 19.9933999999998 27.7352999999995
115.696000000001 25.0383999999997 23.2542999999994
118.883700000001 42.9440999999996 5.80219999999947
130.325200000001 28.8054999999997 14.7176999999994
125.7096 37.1686999999996 -1.14930000000053
-0.339899999994892 -0.97479999999945 -0.270500000000893
3.91740000000495 -3.87489999999946 0.861799999999111
45.7642000000036 2.16290000000023 16.1261999999995
71.6914000000016 0.369199999999837 5.54299999999956
66.4535000000018 -1.3804000000001 -4.08330000000046
125.75 10.1268999999996 6.99179999999951
129.6743 17.3408999999996 -0.240300000000517
ID=TAMmexMALPANUSNM449541

LM3=54
5.9009 -3.2722 -8.3491
6.7357 -0.6757 -8.0804
53.2679 1.1356 -2.2485
61.484 3.689 -2.3354
48.9309 10.9668 -2.1607
69.3493 8.1979 -1.1364
72.3904 8.6955 -0.7431
74.0739 3.1846 16.2147
91.8082 20.0522 -6.983
81.9538 12.5186 3.3906
86.2918 10.0325 0.013
94.7591 0.2556 -0.228
95.6901 11.1875 -3.9813
108.6307 5.8393 -0.2379
115.5219 14.8899 -2.3033
120.6211 17.3976 -3.3062
119.8915 24.9552 -5.7421
126.7024 26.232 4.0006
122.7704 13.513 -0.3162
126.8495 19.2742 -5.7974
4.2091 4.858 -8.9012
5.7993 2.9187 -8.2658
49.1278 21.0942 -2.446
57.7608 22.3218 -2.636
67.0123 21.0282 -1.1893

70.9643 21.8473 -0.9611
70.2471 27.2805 15.9829
79.6925 22.9673 3.5631
82.8818 27.5482 0.3767
87.8739 39.1662 0.0795
93.0131 30.1123 -3.7069
102.7023 38.8887 -0.3873
112.5057 32.9479 -2.6995
117.6765 31.9018 -3.775
118.5195 36.3046 -0.4745
124.3836 32.6497 -5.61
-0.6089 0.299 -0.4117
0.625 3.9094 0.2588
39.3561 14.5203 13.8168
61.2656 26.1645 2.9573
57.7946 25.18 -5.0334
45.6902 10.0724 16.6042
89.5057 18.7783 26.7406
110.8493 23.0967 23.9053
113.2521 40.0727 6.7645
125.0877 25.1593 15.7387
119.4922 34.2913 1.3984
-0.2601 -0.8986 -0.1061
2.4148 -3.6346 0.2837
41.561 2.7016 13.8648
65.9198 0.2984 3.1886
62.6402 0.9878 -4.7725
119.3927 7.969 7.1293
124.0881 16.5451 2.0379
ID=TAMmexMALPANUSNM575605

LM3=54
6.09540000000278 -3.49859999999978 -8.91260000000027
6.64460000000178 0.0584000000002707 -7.70470000000042
51.6792000000012 -0.542199999999934 -0.761000000000194
57.4491000000026 0.847800000000531 -1.88210000000022
46.4500999999991 10.4098999999997 -0.703900000000018
64.4389000000013 7.26100000000032 -0.856000000000269
67.8143000000009 8.2642 -2.09710000000017
72.4414000000006 3.35620000000002 16.6816999999998
85.1919000000011 19.5067 -7.69420000000027
77.6196000000016 10.2960000000004 3.56809999999982
82.0775000000022 8.97330000000039 -1.38250000000024
91.0616000000017 0.0653000000001382 0.0800999999997067
89.212400000001 9.55450000000004 -4.95960000000026
103.253500000001 5.33980000000001 -1.82760000000037
110.212100000002 14.3193 -5.8228000000004
113.901400000001 19.6909999999999 -6.45430000000043
113.503100000001 26.0872 -8.53760000000041
120.303100000001 27.786 0.165799999999519
116.945200000001 15.4897 -3.93050000000044
119.785200000002 21.1844 -9.76730000000048
4.05140000000068 5.07879999999972 -8.74910000000025
5.81900000000032 2.38159999999965 -7.07440000000022

47.0884999999989 22.3109999999997 -0.634600000000073
52.6289999999998 23.7872999999999 -1.59270000000013
60.9671000000004 20.9067 -0.896500000000161
64.9940000000006 21.0648000000001 -1.78700000000018
67.0027000000001 27.7423 16.9212999999998
74.4524000000007 24.0694000000001 4.04419999999979
77.0692000000008 27.3616 -0.655000000000229
82.1847000000006 38.7730999999999 0.45079999999973
84.7427000000009 30.0969 -5.34420000000026
95.4662000000009 39.9845999999999 -1.88250000000036
105.863400000001 35.1157999999999 -5.29240000000039
111.108200000001 33.0624 -5.57850000000044
112.071000000001 36.9214 -2.41720000000045
116.787900000001 33.4427999999999 -9.17390000000047
-0.444799999998431 0.30669999999979 -0.109000000000265
0.441400000000848 4.1175999999997 0.238899999999747
36.6726999999987 14.0559999999996 14.757
59.1196 25.6119 5.73899999999985
53.3485999999997 27.3168999999999 -3.63990000000012
39.013699999999 8.40619999999967 17.9768
86.2318000000008 19.2979 26.0069999999997
107.016200000001 24.0928 21.4245999999996
109.584900000001 41.0828999999999 4.80319999999958
120.245900000001 27.4816 12.8981999999995
114.507800000001 35.8284999999999 -0.90110000000045
-0.0649999999982438 -0.109700000000165 0.195599999999724
2.2617707972164 -2.77266011138253 -0.437273190685596
38.7817999999992 2.79519999999967 14.7147
64.466 2.09539999999993 5.26499999999983
60.1756000000002 -1.96170000000016 -3.79790000000017
115.774600000002 10.0976 3.80189999999958
118.133200000001 17.1252 -1.27930000000044
ID=TAMmexUNKMEXUSNMA13857

LM3=54
1.46340000000198 -4.52669999999954 -10.2848999999998
2.96700000000378 -1.8404999999993 -10.1999999999999
45.339400000003 -4.11599999999967 -7.37530000000026
51.6976000000015 -2.79799999999981 -8.09930000000029
43.2445000000021 7.09920000000034 -5.32830000000007
59.0168000000024 2.89340000000042 -6.21680000000029
62.3372000000013 4.29690000000017 -6.12980000000022
64.9022000000007 -2.51839999999995 10.9677999999998
84.1967000000009 14.9783000000001 -10.1163000000004
74.8473059207436 7.23093893625406 -1.68992961775129
78.6960999999977 4.55119999999968 -4.63100000000017
88.2403000000002 -5.67259999999998 -5.58910000000029
91.031500000001 5.18750000000017 -8.53470000000036
102.080800000001 1.24950000000007 -7.88620000000039
109.731000000001 8.70550000000007 -6.44430000000042
115.054500000001 12.9214000000001 -4.99200000000043
115.106900000001 18.739 -7.96320000000041
119.972700000001 20.1807 0.894099999999564
116.660900000001 9.28550000000005 -2.64340000000042

118.809700000001 14.0618000000001 -7.93430000000045
-0.752299999997789 4.0626000000002 -10.5221999999999
2.47020000000222 2.04200000000031 -9.99899999999988
41.9219000000014 18.2932000000002 -7.62850000000011
48.8487000000012 19.4200000000002 -8.57020000000015
57.3145000000012 16.2987000000002 -6.5680000000002
60.620200000001 15.9691000000001 -6.09270000000021
62.125800000001 24.7979000000001 10.4552999999998
74.3311000000005 17.0306 -1.70370000000024
76.6749000000009 21.7422000000001 -4.78240000000029
82.455900000001 34.4273000000001 -6.30900000000032
87.5294000000008 25.2792000000001 -8.50900000000034
97.9674000000008 32.6145000000001 -6.63070000000037
106.684200000001 26.9498000000001 -6.61560000000042
113.665200000001 24.6565000000001 -4.89700000000042
113.629700000001 28.6433 -2.74190000000043
116.825200000001 24.0659000000001 -8.50630000000044
-0.383699999998182 0.338100000000106 0.274100000000033
0.904800000001587 4.22450000000012 -0.870599999999992
30.615300000001 10.5395000000001 10.2048999999999
53.965914276787 21.6552309758274 -2.18666329157693
50.3697000000012 21.6336000000002 -11.4783000000002
31.3491000000012 5.65370000000009 11.7853999999999
83.4074000000003 13.0899 22.1972999999997
101.3086 16.1125 20.0408999999997
110.094300000001 34.6311000000001 2.9946999999996
115.925400000001 18.9932 14.8688999999996
114.748200000001 26.6883 0.381799999999561
-0.186099999998174 -0.329799999999892 0.205400000000029
2.31460000000158 -3.43899999999994 -0.291599999999966
31.9227000000013 0.667100000000107 10.1411999999999
55.4218836033311 -3.30081756320019 -1.60650046446688
54.5007000000016 -4.5759999999998 -11.1235000000002
113.517700000001 1.80000000000004 2.73699999999958
116.094400000001 10.2613000000001 0.371799999999574
ID=TAMtetFEMBRABMNH111394

LM3=54
3.13059999999969 -4.05180000000036 -10.0846000000002
4.1756999999992 -1.77530000000031 -9.81519999999964
45.889199999998 -5.89390000000048 -4.19419999999999
51.1124000000006 -5.72960000000011 -5.2270000000001
45.9570999999989 7.00259999999957 -3.07899999999997
58.7836000000012 1.08369999999993 -3.23770000000033
61.9432999999995 2.42969999999965 -4.21390000000004
64.2064999999998 -2.45800000000029 14.4949999999999
86.442499999999 14.4752999999997 -11.3977
74.1578999999997 6.83629999999958 1.17239999999997
79.2341000000009 1.47499999999981 -3.73490000000013
87.2906999999999 -6.52430000000036 -3.14690000000008
88.5694999999998 2.56379999999972 -8.71450000000008
102.5248 -1.0518000000003 -6.7613000000001
108.8573 9.18519999999967 -9.4432000000001
113.7406 11.9516999999997 -8.39760000000011

113.0255 19.3954999999997 -11.0290000000001
117.9678 19.9236999999997 -1.97310000000011
117.1177 8.58319999999966 -7.2786000000001
118.3077 13.9742999999997 -12.0403000000001
1.71369999999974 4.53879999999968 -9.99319999999988
3.48679999999979 2.49419999999978 -9.84439999999984
41.3840999999998 19.0741999999998 -3.99560000000006
46.8718999999997 20.6311999999997 -4.46870000000006
56.425 16.9367999999998 -4.05210000000011
59.1580999999999 16.4078999999998 -4.57180000000009
59.0108999999997 22.3598999999998 14.9270999999999
72.4767999999995 17.8449999999997 0.756799999999922
75.3532999999993 23.6817999999997 -3.79630000000005
82.756893810494 35.8201320538095 -3.26224946795443
84.5871999999992 26.9168999999997 -8.87880000000004
96.4201999999994 35.0282999999997 -6.40820000000007
105.7124 28.1386999999998 -9.33250000000012
111.7457 26.7545999999997 -8.18090000000011
113.9865 30.9396999999997 -6.29620000000009
115.9827 26.2334999999997 -11.9955000000001
-1.05020000000009 -0.51470000000034 -0.834099999999964
-0.367100000000042 2.90619999999972 -1.44069999999995
32.3355999999995 11.0566999999997 13.0919999999999
52.6943999999998 21.7060999999998 2.30739999999992
47.3924999999998 23.6629999999998 -8.0247000000001
35.0076999999993 5.24209999999965 16.2074999999999
80.4317999999998 12.7911999999997 19.1364999999999
101.6358 17.5345999999997 17.7981999999999
108.9965 35.7545999999997 2.35299999999989
114.9725 20.2619999999997 12.7451999999999
113.6004 29.9459999999997 -4.78560000000013
-1.23050715101847 -1.59718296074973 -0.757489439315769
-0.127400000000026 -3.86180000000028 -1.01329999999998
34.3910999999991 -1.69730000000035 12.9057999999999
57.3647999999998 -4.29020000000032 1.74619999999987
53.1232000000005 -6.97060000000014 -7.81380000000027
113.6901 3.22029999999963 1.7982999999999
116.5318 10.3890999999997 -3.93380000000012
ID=TAMtetFEMBRABMNH111397

LM3=54
2.6469 -2.9455 -9.8394
5.0064 -0.7055 -9.3746
53.0785 -3.8121 -6.9525
55.7658 -3.0481 -7.4767
45.4855 6.4351 -4.4384
67.9914 2.5267 -6.5028
70.0809 3.3962 -6.152
70.582 -2.6966 11.1906
89.3695 13.402 -11.407
79.3081 6.1244 -0.2693
84.5469 2.0476 -4.0466
93.6618 -6.1123 -4.5562
95.7639 3.4253 -8.9868

107.5406 -0.1631 -6.0921
112.9389 8.4121 -7.8205
118.9264 11.9891 -8.1263
116.6775 18.4334 -11.1273
121.782 19.1499 -0.2355
120.257 8.6329 -6.1726
121.97 12.3212 -11.164
0.8996 4.0693 -9.5101
4.2997 3.0402 -9.3451
48.7595 18.1909 -6.2563
52.107 18.1148 -6.3012
64.3724 16.7642 -5.9796
67.0756 15.881 -6.1544
66.6246 20.9356 11.6668
77.6199 16.9536 -0.9495
80.8545 23.3059 -4.496
87.1115 33.4972 -3.7061
92.0032 25.0042 -9.8953
102.3993 32.698 -5.5926
109.6635 26.6634 -8.1549
116.8473 25.1357 -8.3467
117.4666 28.5621 -6.3654
119.4001 26.1248 -11.0566
-1.861 0.0439 -0.292
-0.0561 4.0291 -0.0147
37.352 11.8572 10.9435
57.832 21.0734 -0.2013
55.5616 22.3772 -9.0682
40.9935 4.5822 12.311
84.4998 11.4661 19.2224
105.7193 15.5066 16.9606
111.2622 32.8369 1.8653
118.6588 17.2708 11.907
117.041 26.5938 -4.4829
-1.6876 -1.1013 -0.4308
1.3195 -4.5596 -0.2451
40.2713 -2.8426 10.1676
62.112 -5.1539 -0.4916
60.1458 -4.2652 -10.3831
116.7973 1.3031 2.9627
119.8111 9.0616 -4.3621
ID=TAMtetUNKBGUBMNH105444

LM3=54
2.52789004586404 -3.20656553289743 -9.79415389362149
4.03026169334148 -1.0097878746328 -8.99250440268651
47.1666999999994 -3.5159999999999 -4.60850000000014
52.2246999999999 -3.30979999999996 -5.54230000000016
43.8605000000005 6.6335000000002 -4.39670000000009
62.2249000000002 2.76909999999999 -3.77720000000021
65.8166000000009 4.17580000000006 -4.25560000000012
63.6919999999997 -2.6159 14.5796
83.8444999999999 13.1814999999999 -10.3910999999999
74.8998000000001 6.8743000000001 0.615300000000104

78.9459000000001 2.01290000000024 -2.90759999999974
88.1213999999999 -7.09789999999998 -2.61899999999991
87.2270000000008 1.71020000000014 -8.38699999999995
101.9774 -2.00779999999997 -5.10049999999998
109.1209 7.82380000000004 -7.27179999999988
113.0675 10.0229000000001 -6.41289999999987
112.5759 17.5752 -10.0257999999999
119.2349 18.8325 0.181800000000099
115.9314 7.164 -5.68099999999989
116.3545 12.3100000000001 -10.6367999999998
1.68226726866198 4.92671958003768 -9.53049026183206
3.78044431336696 3.21697880852349 -8.80836330298104
44.6038000000006 17.2556000000002 -5.33100000000017
49.5810000000005 18.5814000000001 -6.91360000000008
59.2200000000003 16.8017 -4.52060000000009
63.6611000000003 15.2869 -4.37460000000007
59.4740999999999 21.7855 14.6323
73.7075000000001 16.3545 0.513900000000024
75.859 21.853 -2.87009999999997
81.5763999999997 33.1302 -3.09599999999992
83.7458999999998 24.4900999999999 -8.31069999999992
96.4518999999999 32.6362 -5.79019999999995
106.0281 25.439 -7.74149999999988
111.635 24.248 -6.62379999999984
113.0351 28.3351 -5.44979999999987
114.2726 23.5724000000001 -10.5615999999998
-1.15186352600441 0.980071553590466 -0.402907999148097
0.673103080599356 4.31625974660039 -0.261783769365912
35.7161000000002 11.8590000000002 11.9724999999998
56.4071000000003 20.9538000000001 0.732899999999887
53.1390000000005 21.9502000000001 -8.43810000000013
38.7135000000001 6.50520000000015 15.2664999999999
85.3363999999999 12.074 21.4968
106.9087 15.4885 18.5492000000001
109.0065 31.57 3.45800000000008
106.7576 15.9531 18.4329000000001
114.4992 26.3397 -3.25059999999985
-0.978899999999977 -0.208699999999783 -0.419900000000358
1.43850000000006 -2.7290999999997 -0.443300000000271
36.7817 0.508000000000164 12.1265999999998
58.7585999999998 -3.22620000000003 1.48419999999997
55.5614999999993 -4.68890000000004 -7.33630000000014
113.0651 2.15680000000005 3.22420000000009
118.0929 18.4126 13.3683000000001
ID=TAMtetMALBRABMNH242420

LM3=54
3.16800000000466 -3.23799999999838 -10.2436000000011
4.57000000000448 -1.21679999999803 -9.8713000000007
51.3073000000022 -5.67159999999911 -5.98530000000057
57.2538000000021 -4.88679999999917 -6.56200000000058
46.4117000000025 6.41560000000094 -5.52700000000054
64.5380000000016 1.57240000000064 -5.87870000000055
68.1037000000016 2.93780000000061 -6.00690000000055

72.6230000000018 -1.20319999999931 12.1343999999993
90.4366000000012 13.9964000000003 -12.6069000000006
77.9332000000015 5.93950000000046 -0.424800000000601
82.4052000000015 1.44780000000044 -3.58500000000058
90.3028000000014 -7.75679999999948 -3.99850000000062
92.7213000000012 2.79910000000039 -10.6357000000006
105.409700000001 -0.600999999999562 -7.06340000000066
114.010000000001 7.96310000000034 -10.4977000000007
119.226800000001 11.6277000000003 -7.66240000000074
118.746700000001 17.6858000000003 -10.2050000000007
122.588400000001 18.1272000000003 -0.907900000000806
121.303600000001 6.29370000000041 -4.74980000000078
124.629000000001 12.0250000000004 -10.3859000000008
1.98570000000447 4.78890000000184 -10.5118000000007
3.9311000000045 2.98470000000183 -9.98130000000067
47.3169000000026 20.0918000000009 -5.83310000000057
53.1719000000022 20.6340000000007 -6.61070000000056
61.7995000000019 17.6746000000006 -5.85710000000055
65.6419000000019 16.7712000000006 -5.81880000000056
68.907900000002 21.7919000000006 12.3600999999994
76.3006000000015 16.7095000000004 -0.970600000000589
79.2854000000015 22.1371000000004 -3.52610000000061
83.9649000000016 34.6452000000003 -3.96130000000063
89.4785000000012 25.7997000000002 -10.4345000000006
100.773300000001 31.4339000000002 -6.48790000000066
111.229600000001 25.9822000000002 -10.1605000000007
117.899600000001 23.4123000000002 -7.28930000000072
118.012000000001 28.4900000000002 -3.64790000000076
123.389600000001 24.3690000000003 -10.0752000000008
-0.0837999999951999 0.144700000001906 -0.125500000000795
1.30550000000468 3.81650000000186 -0.162700000000757
37.1096000000034 11.4662000000011 11.6974999999994
57.9163046364927 22.3297382848019 0.263823265023763
53.5769000000022 24.7735000000007 -9.16000000000055
39.7120000000033 5.77130000000113 14.5571999999994
88.0896000000017 11.9131000000005 19.0939999999993
107.285100000001 14.8440000000005 17.5422999999992
110.454100000001 32.2730000000002 3.45989999999926
117.496600000001 16.6083000000004 13.9391999999992
119.116200000001 26.0300000000002 -1.96630000000077
-0.0356999999952585 -0.478399999998041 -0.493800000000808
2.22520000000451 -3.30499999999812 -0.450300000000741
38.9676000000033 -0.298699999998824 11.8603999999994
61.6822899843041 -4.94487534812187 0.0278430619736337
58.0951000000019 -7.5350999999992 -9.01980000000058
115.105200000001 0.621300000000442 3.06289999999925
121.455600000001 8.89630000000038 -2.29380000000077
ID=TAMtetFEMBOLBMNH611216

LM3=54
3.11190000000465 -4.06129999999874 -10.8746000000006
4.10760000000445 -1.89779999999838 -10.2312000000006
49.0363000000025 -4.75299999999945 -5.75920000000034
54.3718000000025 -3.62199999999948 -5.9622000000003

42.1474000000027 6.70990000000058 -5.24020000000037
63.1049000000018 1.13890000000036 -4.78480000000024
66.6167000000017 3.23730000000035 -5.58760000000022
71.407300000002 -2.48989999999954 13.6619999999997
89.5938000000008 14.0154000000002 -12.8653000000001
78.3388000000015 6.58590000000027 0.68169999999981
81.6512000000014 1.50630000000029 -2.89880000000017
90.4009000000011 -7.73829999999956 -3.37230000000018
93.4568000000008 1.13150000000034 -9.74710000000008
106.8273 -1.15889999999956 -5.42950000000012
113.1458 8.27940000000039 -9.69120000000011
117.9033 12.9598000000004 -8.15800000000013
116.7796 19.1727000000004 -11.5970000000001
122.5383 20.1539000000004 -1.33100000000016
120.1172 8.93030000000048 -6.00860000000016
121.599 14.7019000000005 -12.0403000000002
1.65000000000434 3.97220000000135 -10.7196000000006
3.30820000000443 2.44700000000134 -10.2695000000006
45.5117000000026 19.9242000000005 -5.59090000000035
50.5056000000022 19.6897000000003 -5.86290000000031
59.9766000000019 16.5646000000003 -4.81590000000025
64.0537000000019 16.4224000000003 -5.25840000000025
66.3611000000019 23.6458000000003 13.5150999999997
75.9656000000012 17.8108000000002 0.353699999999813
77.7538000000011 22.7906000000002 -1.8804000000002
82.694900000001 35.7523000000003 -3.11380000000018
88.2502000000006 27.3167000000002 -9.35660000000014
100.2455 33.9838000000002 -5.67750000000014
109.6894 27.3048000000003 -10.0987000000001
114.2184 26.9898000000003 -7.14070000000014
116.1178 29.6333000000003 -5.56830000000016
119.5777 25.2732000000004 -12.1355000000002
-1.19409999999527 0.460200000001435 -0.541000000000666
-0.157099999995356 3.56060000000139 -0.901900000000678
34.4814000000035 11.4860000000008 12.6214999999995
55.3713792648514 21.8344573377297 0.44039005016719
52.2993000000021 23.8911000000003 -8.45910000000031
34.8572000000036 5.48800000000078 13.6108999999995
87.2916000000014 13.3032000000004 22.3995999999998
107.397500000001 16.7142000000005 19.2185999999998
111.8054 34.6796000000003 2.22579999999981
119.746 19.1047000000005 13.8822999999998
116.8776 27.8827000000004 -2.27230000000013
-0.947699999995344 -0.751499999998554 -0.331700000000658
1.27600000000449 -3.18909999999862 -0.941100000000644
36.0940000000036 -0.395899999999174 12.4557999999996
60.0759443888371 -4.56450491550772 0.167426875016566
57.1597000000023 -5.80929999999948 -8.5644000000003
116.7157 2.3415000000005 1.8002999999998
119.9168 10.8052000000005 -2.90810000000016
ID=TAMtetMALBOLBMNH612480

LM3=54
1.4319 -4.9349 -10.7359

2.7643 -2.5596 -10.7196
53.7843 -5.1921 -5.5976
59.8368 -4.4152 -6.6497
46.802 6.5879 -5.4937
68.2627 2.0689 -6.0577
69.5224 4.4801 -6.1241
72.6551 -0.033 12.5275
93.5026 13.9165 -13.2432
81.1229 7.1582 -2.1657
86.1124 2.4876 -5.2688
96.8757 -6.9021 -4.883
96.2083 2.5741 -10.7613
110.1182 -0.767 -7.8691
115.7366 8.4869 -8.3022
118.706 12.1852 -7.9479
118.1487 18.8993 -9.7048
124.5947 20.2923 -0.3587
121.7348 8.8577 -4.7287
122.651 13.3603 -10.2061
1.2118 3.44 -11.2819
2.9529 1.9773 -10.8522
52.2079 20.3984 -7.3292
57.4285 22.5191 -7.9281
65.8535 17.643 -6.6978
68.0508 16.9545 -6.7571
69.2806 22.5524 11.2581
80.2524 17.7691 -2.4436
83.0261 22.8942 -5.7609
89.5231 35.3765 -5.4861
91.9983 25.6424 -10.5382
104.3429 33.7283 -7.3522
111.7508 27.7593 -8.23
116.4689 25.3692 -6.8422
118.1192 28.8692 -4.8565
121.0637 25.7231 -10.238
-0.2821 0.7472 0.0082
1.6702 3.779 -0.3378
37.2088 10.7963 11.3265
60.595 22.1411 -1.4378
57.452 24.7425 -10.8931
40.2114 6.2743 13.2389
88.7093 14.0172 18.8086
108.0424 16.3473 16.8509
113.572 34.5578 3.029
119.85 18.4054 13.7851
119.2967 26.5671 -2.0707
-0.1302 -0.6627 -0.0339
2.4611 -2.8803 -0.3528
38.8157 0.8735 11.9412
63.5749 -4.0745 -1.0352
61.6162 -6.8495 -9.1972
119.0469 1.6744 3.2884
121.5248 10.6751 -1.9106
ID=TAMtetFEMPERBMNH711156

LM3=54
2.87340000000649 -2.60909999999958 -8.61920000000099
3.57930000000566 -0.761399999999412 -8.39190000000077
48.0294000000035 -3.23199999999976 -4.01230000000031
57.964500000003 -2.58499999999988 -6.18690000000027
48.627500000004 7.22470000000032 -4.25540000000029
68.7266000000022 2.4215 -4.80250000000024
72.4937000000021 4.68149999999997 -5.14420000000025
77.5012000000022 -1.37939999999999 12.1651999999997
91.0039 13.6973999999997 -11.0721000000002
82.531100000001 7.06019999999982 -1.21680000000024
86.6885000000007 2.77039999999982 -4.55670000000023
94.5617000000001 -5.71940000000023 -4.83350000000029
97.0476999999996 3.23039999999968 -9.72390000000022
108.302399999999 -0.286800000000423 -6.30740000000027
114.976499999998 7.35899999999952 -7.08720000000026
119.323199999997 11.0215999999994 -5.88810000000023
118.796599999997 17.8343999999994 -9.27820000000021
124.180799999997 18.3364999999995 0.747499999999719
121.768099999998 7.36749999999949 -2.9823000000003
123.298399999997 11.5182999999994 -8.18900000000027
1.49211625663388 5.01824796672476 -9.34990421183561
3.38453886900964 3.36158623189009 -8.87229419778221
46.4593000000042 17.3675000000003 -3.98970000000033
56.1298000000035 20.4560000000002 -5.83400000000031
66.0789000000027 17.2682000000001 -4.36340000000025
69.6519000000024 16.6323 -4.73510000000024
71.5131000000028 21.6198000000001 12.4712999999998
80.7295000000016 17.1415 -1.25740000000023
83.3713000000012 21.9475999999999 -3.63180000000024
88.8677000000007 32.3644999999999 -4.35380000000026
93.8512000000001 24.6820999999998 -9.22260000000022
104.081399999999 30.9341999999997 -5.59430000000022
112.073299999998 25.9250999999995 -7.10090000000021
117.694799999997 24.3474999999994 -5.86770000000021
118.519799999998 27.3310999999995 -3.52790000000026
121.512099999997 24.3107999999994 -8.19770000000025
-0.529699999994056 0.498600000000744 0.0185999999992131
1.07530000000589 3.69160000000071 0.00199999999924683
41.4096000000053 11.0387000000005 13.2272999999997
64.5821000000032 20.4216000000002 0.793799999999723
57.8436000000034 23.1708000000002 -7.52310000000031
43.2468000000052 5.69400000000048 13.6864999999997
89.1086000000012 11.4540999999999 20.3551999999997
110.074299999999 14.3175999999997 17.0182999999997
112.765899999998 32.0799999999995 3.34119999999976
120.900999999998 16.8527999999995 12.9349999999997
118.734199999997 25.7922999999994 -0.823000000000254
-0.743299999994042 -0.652199999999234 0.0977999999992349
1.30800000000582 -3.34839999999927 -0.350800000000748
43.765100000005 -0.72329999999953 12.9782999999997
66.9614000000028 -2.69499999999992 0.747999999999729
61.2195000000028 -3.75199999999992 -7.83220000000032

117.147799999998 0.655099999999601 2.2881999999997
121.173499999998 8.19739999999948 -1.25630000000028
ID=TAMtetMALBRABMNH781153

LM3=54
1.6357 -2.2649 -9.3398
1.8628 -0.0296 -9.4537
52.4958 -2.9395 -7.0325
56.3765 -2.1706 -7.2384
42.9622 6.8882 -4.4756
64.8187 1.9545 -5.3915
68.139 3.9248 -5.6616
72.3089 -1.9454 11.8088
91.124 13.0466 -10.5798
79.0017 5.9845 -1.5969
83.4863 1.9881 -4.8912
93.4895 -6.1225 -6.6099
94.8612 3.3998 -10.3484
108.0695 -1.3345 -8.5951
114.3461 7.053 -8.8176
117.7551 10.9494 -9.1665
117.8358 16.5075 -10.4482
123.2255 17.2718 -2.424
121.1726 6.3897 -4.8985
122.0283 11.3583 -10.9779
0.2183 5.6441 -8.3119
1.5301 3.8059 -8.3926
49.4192 18.6057 -5.8519
54.2863 18.5746 -6.2418
62.8182 15.9422 -4.9408
66.143 15.5328 -5.3285
68.5853 19.5403 12.7237
77.9713 16.5862 -1.3243
81.0641 21.158 -4.4628
88.6981 31.6732 -5.5523
92.4433 22.7743 -9.9194
104.3931 30.6405 -7.3958
112.1806 24.4895 -7.8714
116.8677 22.1443 -8.2593
118.3083 26.6494 -4.9415
121.3785 23.3833 -10.3794
-1.2964 0.8316 0.7779
0.1057 3.4201 0.6767
39.0108 10.7607 11.0992
58.8164 20.7276 1.3288
54.7933 20.5722 -7.4621
37.908 3.9578 12.3146
86.6278 10.5607 17.9433
105.9141 12.6252 15.9685
110.3332 30.6257 4.7823
120.2386 16.0295 11.6236
118.2619 25.0657 -2.6303
-0.3645 -0.4019 0.1032
1.4092 -2.6571 -0.2118

40.9786 -1.8354 10.0997
61.498 -4.29 -0.7769
59.0815 -3.7945 -8.1805
115.2996 0.0857 3.767
120.41 6.8293 -2.8837
ID=TAMtetMALBMNH781155

LM3=54
2.47860000000118 -4.0734999999994 -8.55120000000022
2.24650000000051 -2.29289999999977 -8.72950000000022
51.5313000000003 -4.71219999999993 -4.81140000000016
59.8956000000001 -3.72909999999997 -6.29400000000015
45.2538000000003 6.42530000000005 -4.70480000000011
65.2518999999999 1.4749 -5.36160000000016
72.6040999999995 4.27279999999994 -5.14480000000017
73.1495000000002 -1.32029999999991 12.8787999999998
91.0117999999991 12.8456000000001 -10.3381000000002
79.6184999999999 5.02920000000008 -0.960600000000229
83.6852999999998 2.04630000000006 -4.14100000000024
92.9216999999994 -6.55809999999988 -3.31940000000032
94.8041999999992 3.10060000000011 -8.64510000000029
108.015699999999 -0.257499999999781 -5.86070000000039
113.682399999999 6.91470000000023 -6.92110000000042
118.758399999998 9.38520000000024 -5.48620000000043
117.458999999998 16.7441000000002 -8.25470000000041
123.519099999998 17.4042000000002 -0.217900000000495
120.633399999998 7.03700000000027 -4.14110000000049
122.717599999998 11.5853000000003 -7.60050000000048
1.12020000000026 2.31670000000038 -8.59620000000025
1.5520000000005 0.331200000000372 -8.23390000000024
48.4685000000002 16.9019000000001 -5.38050000000014
56.0744000000002 18.6717000000001 -6.65190000000015
61.7757000000001 15.2507 -5.40930000000017
70.0984999999998 14.5672 -4.88530000000017
68.2451000000004 19.8966000000002 12.2161999999998
77.8163999999997 15.3774 -0.578800000000202
80.7035999999995 19.4230000000001 -4.87490000000021
86.822999999999 31.3422000000001 -3.97930000000025
91.363199999999 21.5978000000001 -8.71250000000026
101.925699999999 29.8990000000002 -5.68750000000032
110.377299999998 24.6425000000002 -6.66780000000038
116.460799999998 23.1684000000002 -5.7780000000004
115.968199999998 27.4433000000003 -3.54430000000043
120.214999999998 23.9186000000003 -7.70290000000045
-0.936199999999596 -0.0416999999995609 0.0123999999997131
2.66310000000038 3.1868000000004 1.02799999999974
37.1581000000008 10.1490000000002 10.0678999999998
61.6733000000003 19.0986000000001 -0.016300000001658
57.1104000000001 21.8654000000001 -8.45030000000015
35.2536000000012 5.44850000000017 10.2622999999998
87.5371999999996 11.0190000000002 19.7577999999997
106.324699999999 14.5990000000002 17.9109999999996
112.550799999999 31.6381000000002 2.97159999999959
119.812699999999 17.2880000000003 13.9553999999995

119.178399999998 25.3961000000003 -1.17580000000043
-0.538899999999437 -0.697999999999553 0.355799999999753
2.84450000000059 -2.48369999999958 1.08649999999972
37.9978000000009 1.14090000000018 9.95069999999981
64.0007000000003 -2.91919999999997 0.925599999999846
62.4851000000001 -5.47019999999999 -7.69480000000016
116.735799999999 0.688500000000231 3.19599999999954
122.261999999999 9.76530000000026 -2.43650000000048
ID=TAMtetFEMECUBMNH910281

LM3=54
2.64730000000138 -4.65340000000034 -9.7024000000001
3.6453000000014 -1.75510000000065 -10.0700999999997
49.0264000000002 -3.42290000000006 -6.85109999999992
53.1167999999998 -3.73900000000023 -7.65559999999994
41.3644000000005 7.10019999999987 -4.82919999999987
61.686399999999 2.21539999999959 -6.47249999999978
66.3565000000021 4.07900000000015 -6.2014000000001
70.163300000001 0.383699999999965 12.1269
88.2697000000001 14.781 -11.3047
78.8027000000001 7.98859999999996 -1.87719999999997
81.1804 2.84629999999994 -3.17979999999998
92.110600000001 -6.11689999999996 -6.58020000000006
93.3065000000001 2.28720000000004 -9.63020000000001
106.537 -0.884399999999996 -7.69850000000003
113.0905 8.31129999999999 -9.05489999999997
116.7545 11.8654000000001 -8.00130000000001
115.8477 18.8568 -10.4778
122.0331 19.6680000000001 -1.32500000000001
120.2167 8.33790000000002 -5.61520000000001
120.9643 13.3798 -10.7921
1.29250000000202 4.46499999999961 -9.76389999999986
2.9682000000022 2.20759999999953 -9.66379999999984
45.220000000001 19.3798 -7.04239999999994
50.2017000000009 21.3973 -7.91759999999996
58.933600000001 18.2901 -6.55630000000001
63.8507000000011 18.1249 -6.49360000000002
65.6361000000009 23.93 11.291
77.6336000000007 19.3609 -2.25390000000002
79.0632000000005 23.4198000000001 -4.07080000000002
85.5713000000002 35.4567 -6.2531
88.9223000000002 26.9754000000001 -9.67790000000001
100.1545 34.1050000000001 -7.75989999999999
109.6846 28.3482000000001 -8.8046
114.6141 26.1553000000001 -7.40179999999999
116.2378 29.9262000000001 -5.6981
119.3025 25.4304 -10.825
-1.02409999999784 0.326299999999611 -0.287099999999899
-0.608299999997629 3.74029999999964 -0.738099999999874
34.5886000000015 11.9757999999999 10.3145
55.2052943248548 23.239856479866 -1.29559044113943
50.680400000001 23.1877 -9.68449999999998
33.4928000000015 6.29459999999987 11.8808
84.2903000000004 13.2025 20.1425

104.8187 16.3252 17.0243
111.1158 34.8656000000001 2.8372
118.4308 18.8168 11.5945
116.5234 29.1833000000001 -3.3834
-0.686699999997883 -0.624900000000359 -0.165999999999878
0.531800000002338 -3.68890000000036 -0.622899999999876
37.0205000000015 0.762699999999875 10.3149
59.2682954169393 -3.04597391475759 -1.50654103227657
54.3978000000026 -4.07329999999994 -9.34350000000001
115.4129 1.49680000000003 3.30039999999997
119.275 8.87060000000003 -2.7893
ID=TAMtetMALBRABMNH47490*

LM3=54
2.01819999999874 -2.66789999999991 -10.1246000000003
3.29500000000141 -0.350599999999305 -9.59029999999994
52.8130000000004 -4.18629999999954 -8.15340000000023
55.7827999999987 -4.51570000000007 -8.8123000000001
41.4261000000003 6.05230000000025 -5.81280000000024
62.5273999999987 2.21689999999983 -6.63639999999998
69.1779999999999 4.65800000000015 -6.90140000000014
73.3497 -1.23559999999983 12.5156999999999
87.833299999999 14.1644999999999 -12.576
77.0854999999994 6.32720000000003 -3.04700000000005
83.8230999999994 2.64190000000004 -6.52080000000002
90.0454999999986 -6.65030000000011 -5.55859999999999
93.1874999999993 2.19499999999998 -11.1765
105.975799999999 -1.32590000000004 -7.99700000000008
111.201699999999 7.68769999999999 -11.1917000000001
114.771599999999 12.1562999999999 -10.2683
114.148999999999 18.3770999999999 -13.5847000000001
120.535499999999 19.1148 -3.87660000000008
116.501399999999 7.94679999999997 -7.93420000000008
118.924452076922 13.7394073290313 -13.9398925371224
1.09280000000131 4.19820000000048 -10.1350000000002
2.86400000000159 2.08900000000057 -9.61160000000018
48.3903000000006 18.4017000000002 -7.56100000000018
51.2271000000003 20.1245000000001 -7.98630000000015
59.9978000000001 16.0445000000001 -6.47550000000014
67.8199 16.0650000000001 -7.26370000000014
68.4357000000004 21.0507000000002 12.4004999999998
73.9599 15.8783000000001 -1.95430000000012
80.1517999999997 23.5515 -6.6463000000001
83.4096999999996 33.7388 -4.94510000000011
88.8263999999993 26.3485 -10.5882000000001
99.6925999999994 33.9702 -7.1081000000001
107.565899999999 27.0868 -10.4276000000001
113.032899999999 24.3146 -10.4545000000001
112.162999999999 29.0897 -7.48180000000008
116.012699999999 24.4215 -14.8318000000001
-0.342299999999351 0.251800000000401 -0.0153000000002363
0.312700000001202 3.94350000000045 0.461299999999797
36.4641000000012 9.80250000000033 9.88149999999981
58.6713000000003 20.3922000000001 -0.241500000000166

52.3241000000004 21.9816000000001 -9.39660000000015
40.451600000001 4.77710000000034 11.9334999999998
86.6539999999999 12.3427000000001 19.9774999999999
105.8787 15.5614 16.4682999999999
107.1278 33.6934000000001 2.19259999999991
118.118899999999 17.9118 11.6943999999999
114.153499999999 26.7379 -4.21450000000006
-0.324099999999396 -0.675199999999591 -0.228700000000233
1.25200000000055 -4.0965999999996 -0.261000000000208
38.0665000000009 -0.362099999999654 9.6781999999998
60.6826864002331 -4.37630091567553 -1.42410473687907
56.9900000000009 -4.83679999999968 -9.89720000000016
112.643199999999 0.79669999999997 1.28799999999992
117.045699999999 9.53649999999996 -5.15340000000007
ID=TAMtetUNKUNKBMNH331974

LM3=54
1.84840000000275 -4.33960000000053 -10.9143
4.74770748070564 -1.10953202450201 -10.1823544547488
49.9589000000032 -3.68629999999993 -4.33670000000017
56.2159000000031 -2.57460000000011 -4.44500000000025
44.9067000000029 7.09589999999994 -4.07970000000011
66.4083000000005 3.20199999999957 -2.28710000000001
71.7044000000007 4.59509999999981 -2.70180000000007
72.9509000000006 -0.0396000000002967 16.5459
89.7016000000017 14.2213000000001 -8.69470000000008
81.9250000000008 7.75649999999994 1.67580000000003
85.717100000001 3.63879999999987 -2.46210000000004
94.711800000001 -5.94120000000011 -3.13580000000002
95.1617000000015 4.85360000000005 -6.93400000000008
121.838600000001 3.68299999999987 -4.74339999999999
128.7373 11.9090999999998 -7.25969999999997
133.8832 15.0291999999998 -5.50409999999991
132.5981 21.7606999999998 -8.53619999999989
139.2603 23.2266999999998 -1.3915999999999
135.8063 11.3953999999998 -3.94599999999994
138.2643 16.3389999999997 -9.27389999999992
5.52332324334665 6.44854186515644 -10.7662161822019
7.46538130242044 4.65441843673739 -10.090883213566
59.0296000000021 23.9383999999999 -3.07570000000004
65.981900000002 24.9818999999999 -3.30800000000007
76.9173000000016 21.8110999999999 -1.72830000000006
81.7743000000014 21.4325999999998 -1.62220000000006
69.4632000000012 22.5923999999998 15.7828
93.5336000000012 21.7843999999999 2.51799999999998
97.1659000000013 26.4306999999999 -1.22190000000004
102.349500000001 38.4103 -1.46439999999999
106.248200000001 28.926 -5.77510000000001
116.978200000001 36.6739999999999 -3.73069999999994
126.6048 30.7896999999999 -6.15769999999992
132.4654 28.8852999999998 -5.14649999999992
134.1908 31.9174999999998 -3.4435999999999
136.1686 28.3578999999998 -9.24579999999989
-1.95889999999825 0.0091999999996234 -0.807799999999997

-0.413299999998617 3.51969999999963 -1.17279999999996
35.4938000000014 13.3404999999998 11.8061
66.6650927286084 24.1413919663006 3.6627443988988
67.5939000000018 29.3847999999999 -6.52530000000006
38.9153000000015 6.90639999999978 16.9236
92.0265000000009 13.4443999999998 22.5462
112.827800000001 16.6712999999998 19.8556
117.6591 34.0992999999998 2.09610000000008
126.5582 18.3426999999998 12.3004000000001
121.7208 26.4371999999998 -1.9313999999999
-1.96949999999811 -1.65320000000036 0.0084000000000034
0.850100000001684 -3.82800000000042 0.256100000000009
37.7450000000016 -1.45060000000022 12.0988
63.6484222712365 -2.9430241628946 3.05994013440154
60.2903000000015 -4.64860000000023 -7.5741000000001
120.3404 0.992599999999789 1.86630000000005
122.3929 8.77429999999979 -2.64059999999992
ID=TAMtetFEMECUBMNH54645*

LM3=54
2.10770000000393 -3.16120000000022 -8.7015000000001
3.08429999999966 -0.661200000000282 -8.32359999999966
47.914200000002 -3.28479999999984 -5.27370000000006
52.3928999999998 -2.87510000000017 -5.88580000000003
42.5012000000024 6.95220000000031 -3.6071000000002
61.8463999999997 3.18289999999976 -4.67650000000001
64.7432 4.18839999999987 -4.74299999999995
70.0517000000015 -0.885899999999899 13.2324999999999
88.5578000000006 14.1184999999999 -11.4959
78.1980000000024 7.42280000000034 0.113399999999827
80.5670000000022 3.59300000000024 -3.94040000000018
88.9630000000025 -4.89009999999978 -4.0424000000002
91.5274000000025 3.53830000000029 -9.44570000000015
103.983900000002 0.403800000000213 -7.55590000000014
111.649100000002 9.5207000000002 -8.50220000000011
116.141700000003 13.9302000000002 -8.15670000000013
114.693400000002 19.7896000000002 -10.9291000000001
121.337400000002 20.5887000000001 -1.05900000000011
119.487500000003 8.86820000000021 -4.76810000000012
121.114900000003 14.6731000000002 -11.8968000000001
1.47470000000072 4.76669999999973 -9.03719999999998
2.97910000000004 2.83449999999968 -8.74079999999993
44.5284000000011 17.4825000000001 -4.08310000000007
49.877600000001 19.7880000000001 -5.11470000000007
60.1016000000004 16.5068 -4.16220000000003
62.5019000000005 16.1357 -4.35180000000004
64.6951000000011 20.7495000000001 13.9012
76.4712000000008 16.8867000000001 0.958499999999959
77.3153000000008 22.3736 -3.23810000000002
82.5872000000008 32.7927 -2.56260000000002
87.7421000000009 25.3756 -8.6016
98.2824000000015 32.5382 -5.72580000000003
108.031800000002 27.4735000000001 -7.05010000000006
114.912100000002 25.5372000000002 -7.75870000000008

116.353100000002 30.9595000000001 -4.84110000000008
118.377300000002 27.0458000000001 -11.3435000000001
-0.653899999998291 0.394799999999975 -0.0331000000000028
0.713200000001031 3.2302999999997 0.41850000000001
34.3553000000014 8.53250000000001 12.1711
54.6626000000007 19.9523 0.803199999999953
50.6745000000007 22.2856 -7.22240000000006
35.3710000000017 4.46320000000002 13.2945
83.036700000002 12.4948000000002 19.8508999999999
105.135400000002 15.9176000000002 16.0445999999999
109.723100000002 34.1398000000001 0.540499999999993
117.308900000002 18.6524000000002 11.5703999999999
115.748100000002 29.0173000000001 -3.39760000000008
-0.276199999998168 -0.893700000000259 -0.192500000000018
1.4736000000018 -3.45760000000026 -0.477899999999973
35.9945000000018 1.31270000000003 12.2748
57.6459000000008 -3.53840000000006 -0.306200000000028
54.3255000000007 -4.45770000000009 -8.1758000000001
114.411900000003 1.46500000000021 0.523099999999832
118.308900000003 8.73020000000022 -3.92470000000012
ID=TAMtetMALBRABMNH712512

LM3=54
3.3143999999945 -3.45690000000048 -10.6223999999994
5.28179999999753 -1.30689999999984 -9.65919999999996
54.4277999999967 -3.25990000000014 -5.35199999999986
61.1857999999989 -3.41489999999954 -7.01239999999998
50.1743999999979 7.7269999999999 -3.51059999999983
70.6394000000003 2.58020000000035 -5.52539999999997
73.5688999999998 4.0580000000004 -5.81440000000003
79.0550999999994 -1.23459999999976 13.2272
100.285200000001 15.4074000000003 -11.7126
87.6464999999986 6.71790000000019 -2.05359999999987
92.0587999999993 3.73400000000011 -6.20589999999986
103.5515 -8.03019999999976 -5.50399999999991
101.7474 4.35340000000024 -10.8263
114.8577 -0.138799999999795 -8.63999999999993
123.422920839007 9.13522866319126 -9.74710478482059
128.6155 11.6275000000002 -9.54319999999996
127.1877 20.4216000000003 -12.1789
134.1243 21.2609000000002 -2.72789999999999
129.8124 8.79300000000023 -7.64489999999993
132.4988 13.8120000000003 -12.9887
1.74309999999748 5.07590000000018 -9.55510000000008
4.47839999999756 3.52760000000018 -9.06830000000006
51.0231999999992 20.3667000000001 -4.1681
57.7543999999996 21.4661000000002 -5.884
67.7102 18.4282000000003 -5.13130000000002
70.4718000000001 17.8614000000003 -5.10810000000002
73.7545999999999 23.8787000000003 14.454
85.2205 19.7159000000003 -1.47750000000003
87.6210000000003 23.9482000000003 -5.06980000000003
96.3323000000003 38.4232000000003 -5.05410000000006
97.6913000000004 27.0086000000003 -10.3049

111.0707 36.5865000000002 -4.733
120.618270853368 30.229161891602 -9.27895951717723
125.5238 29.2238000000002 -8.8002
127.4159 30.8184000000002 -7.15250000000003
132.1475 26.9860000000003 -12.5354
-1.60620000000385 0.196800000000053 -0.220999999999902
0.465199999996809 3.7882000000001 0.131699999999997
38.2570999999981 11.0154 11.4586
64.8251999999998 23.8922000000002 -1.57490000000004
60.6600999999998 25.7102000000002 -8.23710000000002
45.8944999999979 5.8608 13.4710000000001
95.9581999999996 14.3609000000002 21.4752
117.9696 18.8280000000002 19.148
122.7642 37.6598000000002 1.37959999999998
130.7579 20.4841000000002 13.8995
127.9706 29.4338000000003 -3.74220000000003
-1.58520000000401 -1.15829999999995 -0.657499999999895
0.919599999995609 -3.28529999999996 -1.53009999999984
39.8184999999974 -1.15640000000004 10.4555000000001
69.2218999999994 -2.46599999999976 -1.28589999999995
65.5020999999997 -4.88219999999974 -10.2426
126.162615029089 1.81350888069023 1.29075931121341
130.1137 9.98290000000024 -3.48039999999996
ID=TAMtetUNKBRABMNH293035

LM3=54
1.89548230005158 -4.73103861991855 -9.7108925221422
3.31286872695924 -2.36175870451272 -9.08402016479518
49.369500000003 -4.76439999999939 -6.27660000000028
58.8066000000026 -3.00919999999952 -7.98230000000028
45.8445000000031 6.67270000000067 -6.42020000000026
70.1673000000024 2.47440000000034 -7.22130000000027
74.5618000000022 3.70980000000027 -6.68510000000026
75.5085000000021 -2.53069999999966 12.6977999999997
89.153400000001 13.9373000000001 -11.6184000000002
83.4548000000018 6.18760000000016 -1.22430000000024
86.2847000000016 1.00650000000017 -4.35160000000022
95.7305000000018 -8.6200999999997 -4.77560000000023
97.1789000000013 2.34040000000021 -10.2333000000002
111.496400000001 -1.48779999999976 -7.54340000000018
118.677300000001 9.10340000000024 -8.95610000000013
123.375400000001 12.1420000000002 -8.68810000000007
123.1931 19.9718000000001 -10.8804000000001
130.4421 20.9755000000002 -0.958000000000099
125.878200000001 8.15110000000024 -5.02360000000011
130.2209 15.3389000000002 -10.6318000000001
0.426225542353071 4.50873268217643 -9.55893945161491
2.67040000000452 2.85830000000172 -8.9221000000002
45.6586000000028 18.1027000000005 -6.08230000000023
56.2926000000024 20.6129000000003 -7.92990000000023
67.6513000000019 18.2351000000002 -6.56960000000022
69.8939000000018 17.1180000000001 -6.54510000000021
71.8046000000016 24.1147000000002 12.3055999999998
81.4065000000014 19.0121 -0.609700000000205

81.747900000001 24.8513 -3.91560000000016
87.8650000000006 36.6929 -3.15690000000012
93.0117000000004 27.1417999999999 -9.74430000000013
104.5282 35.6590999999999 -6.42220000000008
115.7625 27.9382 -8.00590000000008
120.4547 28.2214000000001 -7.60630000000009
121.7581 32.0106000000001 -5.41960000000008
127.4477 26.4417000000001 -10.6982000000001
-0.172099999995227 0.256900000001836 0.365699999999761
1.18590000000465 3.97280000000179 0.504599999999776
39.8128000000036 13.9206000000009 11.6417999999997
63.2392599538242 23.2724030535089 -1.62296078286436
56.8994000000022 24.2766000000003 -10.3970000000002
40.8969000000037 6.39480000000093 13.0345999999997
91.9399000000015 13.8042000000003 24.5727999999998
111.889300000001 17.4767000000002 20.9926999999998
116.9169 36.2662 2.8908999999999
125.218600000001 19.6135000000002 15.1882999999998
123.925 29.7723000000001 -2.87180000000008
-0.0957999999951841 -0.740399999998229 0.501299999999801
2.23970000000486 -3.5692999999982 0.531299999999787
41.3901000000037 -2.25299999999908 11.6945999999997
67.4396807914079 -4.17994372982614 -1.7987499481065
62.0848000000026 -6.59129999999952 -10.1625000000003
123.728900000001 2.40520000000035 2.06909999999983
126.9955 11.7695000000002 -3.63080000000012
ID=TAMtetUNKUNKMfNB102648

LM3=54
2.27134071819805 -3.24785956096744 -9.78805468563295
3.60295274890026 -1.16579584882046 -9.2788694280593
47.3889000000002 -3.78599999999983 -5.99979999999994
55.3269000000003 -2.26139999999971 -6.98559999999963
42.2398000000004 6.95500000000039 -7.74900000000021
62.5479999999994 2.03830000000005 -6.21579999999986
67.3008000000015 4.17240000000036 -6.6088000000001
70.1975999999988 -1.4624999999999 12.6907
84.3922000000007 13.4413000000002 -12.7402
76.6888000000007 6.01760000000026 -1.1131
80.3540999999998 2.4545000000001 -4.8826
87.9631999999994 -5.56909999999995 -3.84249999999998
89.2620999999999 2.79830000000013 -9.63390000000002
102.215899999998 -0.0200999999999266 -7.57059999999994
110.436299999999 8.65260000000007 -8.37609999999994
114.210399999998 11.2516000000001 -7.85539999999992
113.410799999999 18.8316000000001 -10.2198999999999
119.113199999998 20.102 -1.37009999999991
117.594399999998 7.01410000000008 -5.75569999999993
121.145799999998 13.3449000000001 -9.87179999999991
0.795968973196412 4.02303395186622 -9.8169076444314
2.98352651689452 2.47690172283726 -9.28157594460045
43.8751000000006 18.5820000000004 -6.49670000000005
51.8362000000004 19.7730000000004 -7.6141
60.3227000000003 17.5595000000003 -6.4137

65.3455000000003 17.7302000000002 -6.92519999999998
66.3734999999995 23.1137000000002 12.5333
74.7385 17.9127000000002 -1.02659999999998
76.8988000000003 23.2697000000002 -5.03119999999998
81.7726999999999 33.4837000000003 -4.46319999999997
85.4041 25.8515000000002 -10.0626
96.9734999999992 33.1851000000001 -7.69969999999994
107.245699999999 27.7890000000001 -8.46369999999993
111.629299999998 26.5912000000001 -7.69899999999992
113.942899999998 31.5330000000001 -5.98409999999992
119.145799999998 26.3622000000001 -9.8350999999999
-0.285000000000202 1.05390000000065 0.137599999999992
0.233099999999795 3.02500000000065 -0.253600000000035
35.1550000000002 10.9772000000005 10.7708999999999
56.6912000000001 22.0881000000003 -3.13290000000001
53.7714000000005 22.9657000000004 -8.7503
35.4877 5.96290000000041 12.2902999999999
82.8003999999987 14.0066000000001 21.3333
102.557199999998 16.5568000000001 19.1057
108.674599999999 34.4918000000001 2.20290000000007
117.526899999998 19.3227000000001 12.3002000000001
115.292499999998 28.6946000000001 -2.48129999999992
-0.149820138070871 -0.145570083384545 0.176597843105527
2.11503303126693 -2.91878923002425 -0.075991888347567
36.6807 1.0478000000004 10.8499
60.8519999999995 -3.25819999999982 -2.3881
57.5657999999992 -4.9504999999998 -8.5816999999998
113.829399999998 2.28430000000007 2.11190000000005
118.525499999998 10.0447000000001 -1.65959999999994
ID=TAMtetUNKBRAMfNB29668*

LM3=54
4.21680000000956 -2.3514999999986 -9.46020000000087
4.82750000000882 -0.013999999997971 -9.08870000000075
50.2001000000053 -4.80689999999932 -5.95500000000059
58.6267000000049 -2.8936999999995 -7.83060000000059
45.4515000000055 6.4067000000007 -4.77390000000058
68.0913000000043 1.41670000000031 -7.25180000000057
70.1145000000042 2.6386000000003 -7.34600000000058
73.197200000005 -2.43819999999959 11.0087999999994
90.236600000004 13.5474000000002 -13.2350000000006
80.1054000000038 5.26650000000016 -2.60060000000058
84.827000000004 1.54870000000018 -6.07300000000062
94.9539000000046 -6.01309999999967 -5.60680000000066
94.8517000000042 2.01150000000024 -11.4602000000006
107.479700000004 -0.715799999999627 -8.12030000000068
116.043900000004 8.32920000000039 -8.89590000000071
118.629100000005 11.7937000000005 -7.96880000000075
118.428600000005 18.9349000000004 -10.0253000000007
125.490600000005 19.9836000000005 0.79939999999919
121.678400000005 6.34820000000049 -4.73960000000078
126.037600000005 15.1646000000005 -10.2337000000008
4.8524000000092 1.99590000000188 -8.81790000000068
3.00370000000885 4.53910000000185 -9.44920000000063

45.9931000000054 19.4482000000006 -5.15290000000059
54.218100000005 19.6310000000004 -6.81760000000061
64.9392000000042 18.8013000000003 -6.93120000000057
68.1286000000043 18.1358000000002 -6.76880000000058
69.5971000000052 22.8743000000004 12.0189999999994
76.3002000000041 18.5510000000002 -1.9900000000006
80.5204000000041 25.1208000000002 -5.68480000000062
87.8187000000047 34.9743000000003 -5.10700000000066
90.8992000000044 27.3207000000002 -11.4073000000006
102.659700000005 34.1861000000003 -7.51600000000067
113.152800000005 27.9281000000003 -8.72300000000068
116.955100000005 25.7111000000004 -7.91080000000072
118.027000000005 31.8414000000004 -4.66260000000075
124.549400000005 24.9500000000005 -10.2276000000008
0.0128000000094657 0.345600000001935 -0.0257000000007197
2.16230000000926 3.5263000000019 0.242399999999326
37.0819000000067 10.0677000000009 11.0702999999994
59.7357030308054 22.315419318192 -0.279819045459857
54.2954000000051 23.4791000000004 -8.39040000000059
40.5381000000067 4.93070000000096 12.3030999999994
86.5248000000052 13.2470000000004 21.3581999999993
107.031900000005 16.8598000000004 18.6560999999992
112.982300000006 35.1472000000005 2.19839999999923
120.255900000006 18.4294000000005 13.9877999999992
119.566700000005 29.2013000000005 -0.851700000000761
-0.100899999990497 -0.735699999998031 -0.581100000000715
3.11710000000921 -3.18629999999809 -0.298200000000691
38.9562000000066 -0.80959999999906 10.4379999999994
64.8761001248888 -4.02107926009448 -1.61451378131581
59.587100000005 -5.52619999999949 -9.1901000000006
117.830500000005 1.19500000000049 2.38199999999921
123.659200000005 9.16790000000047 -1.54650000000077
ID=TAMtetFEMBRAMfNB35312*

LM3=54
3.49360000000247 -2.63449999999909 -9.95970000000005
5.04699999999834 -0.575999999999566 -9.30420000000012
54.5852999999998 -3.54769999999953 -4.34150000000028
66.3545000000004 -2.46899999999952 -5.72050000000011
49.7003999999987 6.99380000000019 -3.83610000000014
73.0767999999993 2.06080000000033 -4.26220000000007
75.2362999999986 3.36740000000022 -4.16050000000008
80.0240999999987 -1.1603999999997 14.2778999999999
93.2971999999991 13.6145000000003 -9.94310000000016
85.3714999999988 6.3692000000002 0.964499999999908
89.0692999999987 2.72940000000028 -2.64990000000011
97.5929999999988 -6.61299999999964 -2.79700000000015
98.8289999999993 1.85800000000036 -8.15690000000016
111.915699999999 -0.133799999999567 -6.9870000000002
119.6117 8.48890000000046 -7.79980000000019
122.266099999999 11.5883000000004 -7.71320000000017
121.3647 18.9133000000004 -10.4849000000002
128.8696 19.6181000000005 -2.81830000000018
124.524599999999 7.39560000000043 -6.84820000000018

127.456999999999 14.6522000000004 -10.9966000000002
2.73619999999882 4.96960000000043 -10.0901
4.78219999999864 3.14980000000043 -9.55559999999997
50.7122999999988 18.3939000000002 -4.0569000000001
62.8970999999987 20.8093000000002 -5.42080000000007
71.6051999999987 18.3439000000002 -4.15620000000007
74.0390332666779 17.7674647453787 -3.95110247339987
74.8186999999987 22.5537000000003 15.4781999999999
83.2941999999987 18.3238000000002 1.07659999999992
85.8233999999989 22.7143000000003 -1.98190000000009
90.659699999999 33.9892000000003 -2.14910000000012
94.5555999999992 26.4294000000003 -7.26080000000012
106.585199999999 33.4390000000004 -5.55460000000013
115.9996 27.9472000000004 -7.67840000000014
119.6432 25.1678000000004 -7.64650000000015
121.0113 30.4472000000004 -6.32680000000017
125.6704 24.3056000000004 -10.7230000000002
-0.123800000000065 0.783100000000564 0.499599999999898
1.08139999999935 4.04930000000053 0.485099999999962
42.4492999999989 10.4017000000003 14.0987999999998
67.8462999999987 23.0032000000002 2.96639999999993
65.0935999999987 23.9594000000002 -7.89930000000007
41.021599999999 5.32380000000037 14.4639999999998
94.663799999999 12.7996000000003 22.8187999999998
113.1231 16.8197000000004 18.0721999999998
115.6673 35.2979000000004 1.66579999999985
127.305099999999 19.4510000000004 8.75609999999979
122.5729 27.6217000000004 -3.06540000000016
-0.253499999999979 -0.594799999999423 0.260699999999901
2.01690000000033 -3.05559999999938 0.18379999999988
43.9806999999993 1.0563000000004 14.1367999999998
71.3192999999992 -3.77049999999968 0.60649999999987
69.3431999999974 -4.26539999999985 -8.3433000000001
121.860699999999 0.960000000000468 1.8855999999998
125.5312 11.6020000000004 -3.73770000000019
ID=TAMtetFEMBRAMfNB35484*

LM3=54
0.900199999998154 -4.77680000000029 -10.8461000000005
2.7078000000002 -1.92460000000029 -10.2483
51.8857000000008 -3.61160000000008 -6.17129999999991
58.5531000000032 -1.78169999999975 -6.94340000000016
48.3513999999997 7.206 -4.29979999999982
67.7894999999999 3.14259999999999 -5.57590000000002
72.1683000000001 5.07469999999999 -5.54209999999985
70.8252999999994 -0.352200000000084 12.4026000000001
90.7832172851066 14.4915452976295 -10.3017862018256
80.9808 6.49159999999997 -0.37279999999992
83.7367999999991 3.48489999999992 -3.08519999999995
96.059499999999 -5.11210000000017 -5.48439999999988
95.6818000000002 4.46939999999997 -9.78739999999989
108.603399999999 -0.0921000000001669 -7.73439999999987
116.6438 8.87579999999993 -9.17799999999987
119.7335 12.0745999999999 -7.57599999999988

120.1326 19.1037999999999 -9.77459999999988
126.1618 19.5651999999999 -0.565699999999856
122.2576 7.56699999999989 -5.96639999999986
126.5909 14.9545999999999 -10.0195999999999
-0.789000000000202 2.81449999999998 -10.7292000000002
2.35349999999993 1.48189999999999 -10.2808000000001
48.7914000000002 19.0712000000002 -6.07109999999992
55.6997000000004 19.6443000000002 -6.98429999999992
65.9805000000003 17.8591000000001 -5.77639999999992
70.1383000000003 18.0132000000001 -5.77779999999993
69.9060000000002 22.6066 11.5855000000001
79.0593000000002 18.6103 -0.185699999999899
80.4891000000004 22.8535000000001 -3.36569999999991
89.9521000000006 34.2234 -5.13429999999992
92.4415000000004 25.6116 -9.23529999999989
103.503700000001 33.5096000000001 -7.31329999999991
114.124600000001 27.2815 -7.90899999999989
117.514200000001 26.0806 -7.84639999999988
119.029400000001 30.4535 -4.43439999999989
124.582300000001 24.8816 -10.0704999999999
-0.186000000000897 0.633099999999911 0.112299999999822
-0.182400000000558 3.58899999999996 -1.01220000000015
35.1033648395923 11.0159404236836 10.3353540489587
60.4863737901138 22.0539952513116 -1.08224412896032
56.1599000000003 21.8150000000001 -10.0822999999999
36.2363999999999 5.53160000000005 12.039
87.9215 13.7288 19.4447000000001
110.6191 17.1025999999999 16.6837000000001
115.155400000001 34.1639000000001 2.27480000000009
120.6557 18.5467999999999 13.6515000000001
120.225000000001 27.8201 -2.76899999999989
0.126699999998994 -0.69010000000015 0.0975999999997877
0.957299999998757 -3.54350000000017 -1.37010000000019
37.4935999999998 0.542399999999993 9.81540000000005
63.7392000000001 -2.4392 -1.69899999999995
59.6010999999982 -3.97940000000011 -10.3865999999999
119.2948 2.80069999999986 3.16630000000014
122.9793 10.4170999999999 -2.72409999999988
ID=TALtetFEMBRAMfNB38395*

LM3=54
4.43209999998812 -3.56880000000214 -10.979
6.18619999998768 -0.984500000002165 -10.5392999999994
47.3349999999921 -3.07240000000147 -4.03839999999969
61.2205999999928 -2.08150000000138 -6.00499999999971
43.8854999999921 6.91739999999847 -5.32799999999973
69.8017999999938 2.49479999999874 -5.44149999999975
74.8754999999941 4.96759999999879 -4.79819999999978
76.2021999999943 -2.21370000000123 13.5891000000003
93.702099999997 15.0056999999993 -11.9844999999998
82.8883999999955 6.53239999999904 0.225100000000218
87.9698999999958 3.21159999999909 -3.56979999999976
97.2289999999961 -6.38540000000085 -4.68389999999974
99.848699999997 3.83009999999932 -9.24809999999978

113.777799999998 -0.828000000000598 -6.87909999999974
121.141699999999 9.43309999999961 -8.92999999999976
124.815899999999 11.9357999999996 -7.93359999999974
125.304199999999 20.5329999999997 -11.1283999999998
131.330999999999 21.2576999999996 -2.22549999999976
128.365099999999 8.10609999999951 -5.19349999999974
131.341399999999 14.7508999999996 -11.3339999999997
3.43519999998753 4.00109999999777 -10.8588999999995
6.07299999998759 2.1870999999978 -10.4850999999995
44.9150999999925 17.7310999999985 -4.13699999999972
57.7200999999932 21.4068999999987 -6.73089999999975
67.1927999999943 18.8639999999988 -4.75159999999978
72.4171999999945 17.7908999999989 -5.32339999999978
71.5435999999944 25.1131999999988 13.6728000000003
80.0630999999958 19.3166999999991 -0.154899999999801
83.4620999999963 25.0519999999992 -3.4570999999998
90.1475999999965 36.3010999999993 -4.28779999999977
95.7140999999975 27.8880999999995 -9.44429999999982
107.902699999998 37.1847999999996 -6.7700999999998
118.123599999999 29.5424999999998 -9.3307999999998
122.470999999999 27.8456999999997 -7.58029999999979
124.588699999999 32.4032999999997 -4.97449999999977
128.965699999999 27.1964999999997 -11.4572999999998
-0.586500000012638 0.452299999997734 0.0916000000005641
1.92429999998752 4.25759999999773 0.111200000000531
36.7898999999911 11.4594999999983 12.5004000000003
62.4501999999938 23.8345999999987 -0.937899999999764
58.3245999999934 23.9796999999987 -9.13849999999975
36.5540999999909 5.45349999999828 14.6849000000003
91.5787999999957 14.595699999999 24.9591000000003
117.196499999997 19.0048999999993 18.8876000000003
121.265299999998 37.4836999999996 3.33830000000022
129.335399999998 20.6600999999994 12.6034000000003
125.871699999999 30.3250999999997 -2.72229999999978
-0.476700000012596 -0.766700000002305 -0.594899999999439
3.29329999998757 -3.91020000000224 -0.319599999999455
38.4360999999908 -0.426800000001752 12.0367000000003
67.6939913445 -3.11615324923353 0.423339111708565
62.6642999999929 -5.08510000000138 -8.8451999999997
127.519599999998 3.04349999999938 1.72290000000029
129.340499999999 10.6594999999995 -2.38179999999974
ID=TAMtetMALBRAMfNB38396*

LM3=54
3.0485208775216 -3.43361998586061 -9.85935231668333
4.49779999999978 -1.16819999999984 -9.21159999999997
52.6438000000017 -4.32579999999949 -4.9862000000003
61.2200999999996 -3.03549999999997 -6.21980000000006
46.6002000000002 7.04250000000028 -4.39230000000016
67.9053999999983 2.77059999999974 -4.5823000000001
71.7948999999987 4.57479999999995 -3.93150000000006
76.3552999999999 -1.90859999999992 13.7210999999998
90.4011999999997 14.2019999999999 -9.96750000000033
82.553299999999 6.28269999999993 1.04569999999985

85.8464999999995 3.41090000000001 -2.03270000000028
95.8663999999993 -6.71910000000004 -3.81430000000034
96.622499999999 3.34489999999985 -8.51170000000027
110.279199999999 -0.183100000000048 -7.24420000000038
118.360899999999 9.28700000000005 -8.36410000000046
121.352099999999 11.1374 -7.66160000000045
122.0714 19.1395000000001 -9.59860000000046
128.2088 19.8351000000001 -0.926800000000488
124.750999999999 8.01550000000007 -5.00800000000048
127.884799999999 14.8667000000001 -10.6868000000005
3.30849999999907 5.06170000000009 -9.00980000000005
3.78609999999897 2.73960000000006 -8.94670000000003
47.7086999999996 19.9864 -4.02400000000013
56.6836999999998 21.7958 -5.16160000000015
65.1040999999999 18.2406 -3.40970000000017
68.3949999999998 17.0981 -3.39440000000015
72.8509 23.8656 15.6078999999998
79.2863999999999 19.2182 1.20889999999978
81.6818999999999 23.2264 -0.888400000000247
89.1323 36.0327 -3.01880000000031
92.9823999999999 26.7337 -7.94440000000032
104.531 34.3168 -6.32480000000039
115.1765 27.5610000000001 -8.27110000000042
118.7918 25.9929000000001 -7.26520000000047
121.1692 30.6654000000001 -4.34030000000046
126.0312 24.8498000000001 -10.5130000000005
-0.703000000000976 0.744000000000119 0.110499999999936
0.344399999998745 4.29880000000009 -0.347400000000073
39.9170999999992 12.3439000000001 12.4909999999999
63.6562900569345 23.6268906015847 2.20456183072685
58.4724999999998 25.2708 -7.49760000000015
41.6805999999994 5.60230000000011 13.7516999999999
88.4482999999998 13.5921 22.6427999999997
112.1685 17.0136000000001 18.8764999999996
115.8658 36.3571000000001 5.66049999999956
125.0027 19.1113000000001 14.3606999999995
122.6237 28.9082000000001 -2.54650000000046
-0.476500000001012 -0.675899999999908 0.294899999999928
1.3357311282025 -3.16490791140609 -0.248896205787305
42.6248999999995 -0.817299999999848 12.0762999999999
67.7462714100842 -3.47078318870797 0.565405777109845
63.6967000000015 -5.9171999999997 -9.00700000000019
121.571599999999 1.04160000000003 4.77879999999954
126.279699999999 10.6735 -2.76730000000046
ID=TAMtetFEMBRAMfNB39556*

LM3=54
1.47148669968777 -3.37003711401069 -8.54744724988846
2.7233000000011 -1.04799999999961 -7.55620000000003
46.1346000000013 -3.97399999999971 -5.39269999999988
55.8455000000011 -2.57919999999991 -6.45610000000007
42.6709000000011 6.74790000000023 -3.98079999999973
63.3081000000018 2.36910000000032 -5.47980000000003
64.7641000000016 2.98490000000036 -5.82699999999998

69.4564000000004 -1.25339999999985 13.0409000000001
82.8432000000004 14.1304000000001 -11.3603
77.2116999999996 6.6715000000001 -1.42629999999987
79.8716000000006 2.84800000000019 -3.23309999999991
88.495500000001 -5.71829999999975 -4.38909999999995
89.1400999999997 3.06460000000005 -9.79519999999991
102.0236 -0.116399999999909 -6.65349999999992
108.9797 9.08170000000007 -8.3421999999999
111.9872 12.3120000000001 -7.32309999999987
111.8335 19.6882 -10.1092999999999
119.5914 20.846 -1.20529999999984
115.5792 8.7327000000001 -4.11549999999988
117.4655 15.5273000000001 -10.2042999999999
1.38990000000079 4.14130000000015 -7.98989999999982
2.22870000000086 1.43530000000016 -7.53039999999986
42.4919000000008 18.6340000000002 -5.2909999999998
52.0103000000007 20.1016000000002 -6.66139999999983
60.1286000000008 18.0231000000002 -5.71019999999988
62.2404000000006 17.8826000000002 -5.76819999999988
65.3467000000002 22.8786000000001 12.6554000000002
74.2027000000002 19.4405000000001 -2.12869999999988
75.6913000000002 23.4932000000001 -3.52249999999988
81.1577000000001 35.2564000000001 -3.79369999999986
84.8115000000002 26.9357000000001 -9.44589999999989
94.9944999999998 34.9294000000001 -6.41049999999985
105.7948 28.219 -8.23409999999985
109.8862 26.3222 -7.24479999999985
111.7354 30.3179000000001 -4.12889999999985
115.295 25.2953 -10.0991999999998
-0.845699999999169 0.811500000000157 -0.284499999999853
-0.525399999999218 3.61010000000015 0.291400000000158
34.5491000000005 10.2369000000001 12.5200000000001
54.4034000000006 23.6498000000002 0.100400000000167
52.4580000000007 22.9867000000002 -9.63059999999984
34.8197000000006 5.57760000000018 12.8965000000001
83.1195999999999 13.6644000000001 21.5008000000001
102.6017 17.7942000000001 18.8987000000002
105.9417 36.468 2.75300000000018
115.4941 19.8666000000001 13.2227000000002
113.2889 28.4948 -1.72219999999984
-0.566399999999915 -1.18189999999984 -0.0485999999998514
0.767900000000086 -3.59299999999985 0.278200000000144
35.6612000000007 0.692500000000191 12.1553000000001
59.9763000000008 -4.67809999999981 0.166700000000053
57.656900000001 -5.10539999999981 -9.56759999999989
112.1326 0.795900000000012 2.54180000000011
116.5196 10.9269000000001 -1.98859999999988
ID=TALtetMALBRAMfNB47915*

LM3=54
2.72779999999548 -4.64090000000065 -11.1150999999997
5.12389999999596 -1.86920000000048 -10.5517999999996
52.6902999999978 -5.15390000000044 -5.65599999999973
63.243599999998 -3.12590000000039 -7.03609999999976

47.0239999999978 7.02849999999967 -4.15159999999982
70.3471999999982 2.09859999999963 -4.2323999999998
75.994399999998 4.37889999999958 -4.61909999999981
76.7858999999976 -0.726300000000487 12.6326000000003
95.3669999999979 15.3347999999996 -10.4876999999999
85.0761999999981 7.1688999999996 -1.21619999999985
91.1011999999982 3.62669999999957 -6.73309999999983
98.977099999998 -5.90620000000051 -4.58509999999974
101.142599999998 4.73719999999955 -10.1376999999998
113.866199999998 0.300099999999459 -7.46369999999973
122.930299999998 9.69229999999945 -7.79979999999976
125.988199999997 12.8063999999994 -7.41109999999977
126.105517622515 20.7036922822376 -9.5484758584132
132.853232002161 21.8031924158136 0.336997576191704
130.381264318679 9.03387479911482 -4.46697661308133
131.917736093724 15.2183667356323 -10.1486844549997
1.13569999999576 4.86099999999945 -11.0457999999997
4.3170999999958 2.83519999999947 -10.5639999999997
48.1475999999978 20.7829999999997 -5.52379999999992
59.0880999999979 21.9179999999997 -6.70189999999994
67.247899999998 19.9319999999997 -4.62389999999995
72.702599999998 18.8120999999997 -4.25239999999994
73.4775999999977 24.6132999999996 12.0387000000001
82.9777999999981 19.4613999999996 -1.19779999999996
85.3791999999978 23.8733999999996 -4.74149999999997
91.3299999999972 36.3626999999995 -3.68669999999997
96.3101999999975 26.8732999999995 -10.3372999999999
106.971799999997 35.9610999999994 -6.45879999999992
119.171899999997 29.8543999999994 -6.20869999999988
124.119944038461 27.9380239249557 -6.13948188948668
125.822439194446 33.0035141297107 -3.06280756942394
129.657929844651 27.7912390535043 -9.5699439048548
-0.620800000004476 0.423399999999414 -0.134399999999619
0.614999999995732 3.99729999999945 -1.06129999999965
37.867899999997 11.0354999999996 11.7219000000002
62.6124999999979 24.4155999999997 -0.142299999999968
59.0166999999977 24.4642999999996 -9.55479999999996
41.8170999999971 6.58299999999957 14.1600000000003
93.5818999999974 15.0589999999995 23.3734000000002
114.262399999997 18.7558999999994 20.3844000000002
119.067931675769 37.3878315920456 4.373378060724
130.019869871475 21.3902723382183 14.3648407513972
126.411668012099 30.6869021625603 -0.15818662975729
-0.344600000004454 -0.922500000000582 0.054300000000377
1.72659999999571 -3.71180000000059 -0.663899999999609
38.9034999999971 1.11859999999955 11.3038000000003
67.9563999999981 -4.07310000000041 1.19160000000025
63.707799999998 -5.15440000000043 -10.0649999999998
128.233499999997 3.13559999999939 3.08910000000028
130.356601844009 11.0477697892209 -1.32241906349724
ID=TAMtetUNKBRAMfNB56796*

LM3=54
2.85689999999752 -3.0314000000013 -8.47919999999939

4.42889999999829 -0.692700000000686 -7.75019999999948
48.8304999999986 -2.94510000000075 -4.30449999999973
55.9452999999988 -1.94310000000075 -5.99569999999975
45.8684999999986 7.64979999999928 -3.44829999999972
64.309999999999 3.28549999999931 -4.20249999999979
66.244199999999 4.74319999999928 -4.37029999999981
69.526699999999 -0.10180000000069 12.4028000000002
89.3026999999997 14.6956999999995 -11.2261999999999
77.4872999999995 7.55559999999935 -0.628699999999873
80.9858999999997 3.41969999999937 -4.9024999999999
89.7152999999993 -4.90230000000069 -3.90669999999987
91.6953999999999 3.42559999999941 -9.66279999999995
103.4971 0.378999999999423 -8.00279999999993
112.4098 8.30929999999952 -9.65169999999996
116.518931521584 10.8854492664642 -9.4077123012597
116.796966794531 17.2724671269783 -11.882657160103
122.18818225979 18.3760627168704 -2.75535501990046
119.238844182605 7.10636975197004 -6.66045503777614
121.975749491733 12.6173033557856 -12.2167571981045
1.57829999999804 4.12909999999916 -8.06639999999954
3.7374999999982 2.41179999999918 -7.61129999999955
45.232199999999 19.4915999999993 -4.14739999999979
52.3462999999989 20.2315999999993 -5.53719999999982
61.5359999999992 18.3132999999994 -4.41119999999987
63.3328999999992 17.6812999999994 -4.53429999999987
67.0264999999991 23.0144999999994 12.4933000000002
75.6453999999996 18.3139999999994 -0.686699999999931
79.2749999999998 21.9818999999995 -3.87859999999995
84.4803999999999 32.9315999999996 -3.70129999999996
88.9327999999999 25.2916999999996 -9.6109
99.1653000000002 32.1884999999997 -7.29559999999999
110.386929287592 26.2672970696238 -9.32204861188421
115.074172194311 24.0498992762007 -9.14816862623564
116.710657254504 28.8940735729834 -6.37552151739827
120.457796703761 23.5994262735784 -12.1702363978045
-0.891800000001889 0.270299999999135 -0.232899999999506
0.572799999998046 3.07759999999914 0.0525000000004991
33.1844999999985 11.1098999999993 11.3620000000003
58.622999999999 22.2547999999993 -1.15429999999984
53.980699999999 22.8361999999993 -7.32029999999984
37.3115999999984 6.25269999999929 14.4726000000004
85.3498999999994 13.7643999999994 20.3142000000001
104.3038 16.3365999999995 18.1710000000001
110.2954 33.9326999999996 0.906700000000024
118.413772889162 18.0398021960501 11.0034334830309
116.936874187008 26.8907331386519 -3.60485905477009
-0.781800000001908 -0.7397000000009 -0.173999999999531
1.45759999999809 -3.13710000000087 -0.0187999999995097
34.8468999999984 -0.16770000000073 11.1515000000004
62.0238999999987 -1.47440000000076 -0.997999999999754
57.8210999999989 -4.02430000000073 -7.60589999999976
113.8785 0.393099999999417 1.30980000000008
118.876912249645 8.64340859739941 -3.70891980455236
ID=TAMtetUNKBRAMfNB81444*

LM3=54
2.99960000000705 -5.02159999999886 -11.0562000000005
5.15699088690017 -1.79839071434986 -10.4179915405453
55.2522000000049 -3.88429999999936 -6.38060000000031
63.9387999999999 -2.56999999999997 -7.13080000000002
51.1928000000052 8.24110000000059 -6.28270000000021
72.0727000000021 2.97700000000021 -5.51330000000004
75.3632000000042 3.64450000000065 -5.6085000000003
78.4744000000031 -1.35949999999969 12.8681999999997
100.812000000002 15.7303000000001 -13.1575000000002
88.2027000000014 7.81610000000016 -0.79760000000017
91.9737000000031 5.10810000000034 -4.47840000000021
104.324200000003 -5.5828999999997 -4.13540000000028
103.591600000002 4.64860000000017 -10.1134000000002
117.441600000002 -1.09549999999978 -7.53300000000027
127.329700000003 9.77810000000023 -8.57360000000029
131.071900000003 13.0721000000002 -7.4725000000003
132.584700000002 21.0665000000001 -10.0023000000003
137.808300000003 22.0582000000002 0.930699999999681
134.140600000003 8.96450000000024 -3.19540000000034
137.550800000003 15.9662000000002 -10.6092000000003
1.74360000000514 5.20150000000068 -11.4759000000002
4.43000000000479 1.22330000000055 -9.99180000000014
50.443200000004 20.8000000000004 -6.18130000000023
60.0726000000033 22.3240000000003 -7.23620000000019
69.5091000000027 21.2106000000002 -6.12180000000017
73.2505000000026 19.6718000000003 -5.69740000000018
72.8366000000025 25.9316000000002 12.8927999999998
85.7469000000019 21.3863000000001 -1.04820000000018
88.664200000002 25.0358000000002 -5.14240000000018
97.1367000000022 38.1798000000002 -3.82340000000023
99.6065000000018 27.8515000000001 -10.3299000000002
110.570400000002 37.9216000000001 -7.59420000000026
123.615800000002 30.2620000000001 -8.13870000000031
128.426400000002 27.8333000000001 -7.83280000000029
130.268500000003 32.7399000000002 -4.22640000000033
136.187100000003 27.4250000000002 -10.8172000000003
-0.884999999994405 0.880000000000797 -0.103100000000322
0.38810000000507 4.87590000000066 0.436899999999703
38.8007000000042 12.1519000000004 13.1970999999997
65.4714000000029 25.5313000000003 -1.1550000000002
60.2122000000033 26.0311000000003 -10.1681000000002
41.2006000000043 6.98720000000047 14.5516999999997
95.7544000000025 16.3596000000002 23.1553999999997
115.594000000003 19.1658000000002 21.0494999999997
124.164600000003 39.3494000000001 1.61929999999973
131.082700000003 22.1190000000002 15.4718999999996
131.785900000003 30.6989000000001 -0.372800000000304
-0.799299999994348 -0.585399999999139 -0.0354000000003607
1.33120000000551 -4.05199999999921 0.0040999999996848
40.2890000000046 0.710300000000502 12.5288999999997
70.0558000000032 -3.56519999999961 -0.468700000000239
65.0962000000043 -6.09679999999952 -9.37960000000031

129.506400000003 1.36410000000023 1.70539999999967
134.808100000003 12.0470000000002 -0.312200000000321
ID=TAMtetFEMBRAMfNB81452*

LM3=54
3.29759999999802 -4.56539999999923 -8.87320000000022
4.02659999999817 -2.2722999999991 -8.61210000000042
57.5569999999992 -4.71739999999963 -5.84770000000001
62.1461999999991 -2.59089999999973 -6.653
47.689099999999 7.62830000000035 -5.25760000000002
69.8924999999992 2.68220000000025 -4.57639999999997
72.5422999999989 4.16320000000019 -4.84029999999995
77.1278999999996 -2.19839999999964 11.7999
96.8582999999989 15.7182000000003 -10.9837999999999
85.1740999999996 7.21820000000031 -0.976499999999977
89.3455999999995 3.64630000000028 -4.40319999999998
99.6552999999991 -6.55049999999966 -3.3545
100.832499999999 5.29820000000028 -9.34449999999999
114.393399999999 0.547900000000357 -7.5522
123.656399999999 9.4302000000004 -8.76390000000001
127.748499999998 12.8723000000004 -8.53250000000002
127.276899999998 20.9566000000003 -11.3566
133.277899999998 21.7924000000004 -1.46030000000003
129.722599999998 6.77900000000039 -4.69510000000004
134.370399999998 15.5354000000004 -12.0324
1.81429999999802 4.77000000000084 -9.19740000000026
3.08679999999831 2.54880000000083 -8.58310000000027
52.9988999999988 22.8679000000003 -6.90030000000003
57.924599999999 21.5621000000004 -7.10260000000003
68.0171999999991 19.4699000000003 -5.295
71.172999999999 18.8376000000003 -5.20379999999999
74.3201999999996 26.7327000000005 11.1681
83.1010999999992 19.5007000000003 -1.14739999999997
85.7839999999992 24.0561000000003 -4.02169999999996
92.0291999999987 37.2648000000003 -3.54889999999994
96.6068999999988 26.2737000000002 -9.77209999999995
107.968699999999 36.4595000000003 -5.95639999999996
120.273399999998 30.3486000000004 -7.88189999999997
124.887399999998 28.3238000000004 -8.31489999999998
125.964899999998 34.6745000000004 -4.3771
131.739399999998 27.9583000000004 -11.7777
-0.398500000001825 0.422800000000877 -0.237900000000266
0.563399999998153 3.84200000000085 -0.267500000000238
37.1343999999992 12.4493000000006 10.3938999999999
63.1250999999992 25.0429000000004 -0.311500000000019
58.2954999999989 24.3842000000004 -9.24890000000002
35.0122999999996 6.02020000000057 12.2530999999999
94.2943999999991 15.0165000000004 21.0894
114.203999999999 18.9067000000004 19.0037
120.537899999998 37.2631000000004 2.8279
128.659199999998 21.1405000000004 14.4389999999999
126.929699999998 32.4587000000004 -1.45290000000001
-0.230800000001755 -1.16209999999909 -0.123300000000271
1.71369999999839 -3.81439999999911 0.182899999999741

38.3610999999994 -0.456299999999472 10.5968999999999
66.3191999999996 -4.50219999999972 -0.561899999999971
63.7757999999992 -5.20929999999973 -8.2811
126.300399999998 2.6531000000004 2.75479999999996
131.249799999998 9.3828000000004 -1.91420000000005
ID=TAMtetMALBRAMfNB91288*

LM3=54
3.761 -3.1114 -9.4254
5.3581 -0.7336 -8.6484
46.1871 -1.9976 -5.8003
54.2229 -1.4023 -6.9857
44.5977 8.5794 -5.5017
63.6845 3.6496 -6.1598
65.4884 5.1756 -6.0259
71.7249 -1.0768 12.837
85.503 15.6594 -11.3357
76.8175 7.2448 -2.8117
80.361 3.6959 -5.2703
91.1505 -5.9181 -6.0721
90.9912 5.5692 -11.56
104.6763 0.0858 -8.2805
113.1783 8.7665 -9.0556
116.6963 11.6657 -9.2226
116.8414 19.9141 -12.1712
123.7023 20.7152 -2.0157
120.8119 7.0389 -6.6958
124.7777 13.6571 -10.1341
2.2267 4.8681 -9.0105
4.5969 3.3607 -8.4963
42.5677 19.5815 -5.3874
50.3973 21.3277 -6.9089
61.4622 20.116 -6.6077
63.769 19.1158 -6.6168
63.0049 25.9014 12.5378
74.3242 20.4403 -2.935
77.1471 25.0565 -5.5567
84.201 37.654 -5.3835
87.9784 26.7917 -11.2231
99.1361 36.3537 -8.1426
109.9661 29.7503 -8.7837
114.312 28.1673 -9.2365
116.5356 33.9578 -5.6413
121.6881 28.4581 -11.1156
-0.5925 0.5245 -0.1591
0.8899 4.0358 -0.4744
32.9026 11.6569 9.6981
57.1705 24.1862 -0.7595
51.7011 24.411 -9.499
34.3636 6.7321 12.0307
86.0735 16.1463 20.8898
105.3463 18.4175 18.0883
112.1887 37.7873 3.9641
120.6924 20.9225 12.1558

117.9539 30.6425 -2.8775
-0.4152 -0.6448 -0.0055
2.1764 -3.6348 -0.6111
34.5364 1.1086 9.2682
61.351 -1.7031 -1.0548
56.153 -3.9144 -9.6618
117.6724 2.9167 4.4999
121.1582 11.3497 -3.721
ID=TALtetUNKBRAAMNH133477

LM3=54
1.93630000000152 -4.24109999999978 -10.2829999999998
2.72190000000527 -2.01099999999936 -9.96200000000004
50.756500000003 -3.38299999999969 -5.96660000000003
57.1996000000033 -1.68079999999951 -6.3277000000001
44.1987000000035 7.95390000000038 -5.24030000000016
68.8965000000019 4.06230000000031 -5.30590000000004
72.5534000000016 6.25950000000024 -4.6694000000001
74.1709000000015 -0.280499999999772 14.7478
87.3772000000007 14.4806000000001 -9.60440000000018
83.5990000000011 8.8961000000002 0.315899999999928
85.4663000000012 4.96030000000022 -2.95450000000015
94.3054 -5.48929999999992 -2.90920000000008
94.9580000000005 4.69330000000009 -8.47960000000013
108.253 -0.0853999999999097 -6.52140000000012
116.4608 9.35620000000005 -7.82820000000013
120.5675 11.5634000000001 -7.81860000000016
121.2905 19.3914000000001 -10.1718000000002
127.7176 20.2071000000001 -1.79850000000014
123.293999999999 8.11900000000002 -5.10500000000011
125.492799999999 13.5886 -10.6431000000001
0.470500000004519 4.18620000000059 -10.7268
2.07240000000501 2.09180000000067 -10.072
46.5315000000036 19.7848000000005 -6.12630000000012
53.6739000000033 20.4128000000005 -6.44140000000013
66.7537000000026 18.6123000000004 -5.32100000000011
69.2070000000023 18.8186000000004 -4.99790000000011
68.6148000000023 24.6003000000004 13.9586999999999
81.7909000000015 18.7195000000003 0.23429999999989
82.8026000000013 23.5495000000002 -3.40650000000013
88.2316000000014 35.9216000000003 -3.05760000000017
91.8369000000007 26.4258000000001 -8.75600000000014
103.026700000001 34.7804000000002 -6.59950000000016
113.7658 27.4593000000001 -7.31200000000016
118.4464 26.3104000000001 -7.77430000000017
120.7516 30.7514000000001 -4.74560000000015
124.1175 26.2272000000001 -10.6731000000002
-1.862299999996 0.44530000000057 -0.190599999999928
-0.144699999995547 3.57640000000061 -0.418099999999955
34.8459000000041 11.6865000000005 10.309
61.1796000000028 23.0177000000004 1.08009999999988
54.4586000000032 23.4023000000005 -8.63350000000013
35.6783000000038 6.87190000000049 12.0437
89.1413000000011 14.1255000000002 23.4222

112.5705 18.2305000000002 19.5134999999999
114.7145 36.3134000000002 2.29539999999984
127.3168 21.2839000000001 12.1074999999999
121.4899 29.5301000000001 -2.40300000000017
-1.54469999999613 -1.17819999999944 -0.129299999999906
1.03590000000381 -3.63009999999946 -0.00509999999992133
36.4962000000037 1.35930000000047 10.3078
66.3722000000025 -0.933499999999711 2.51419999999992
58.835500000002 -4.2249999999997 -8.30739999999998
120.501099999999 2.12920000000007 2.42639999999991
124.436299999999 10.4895000000001 -2.34740000000012
ID=TAMtetMALBRAAMNH133499

LM3=54
2.9414000000063 -3.34449999999845 -10.3850000000001
4.2196000000063 -1.16279999999852 -10.0352
54.7084000000036 -3.82059999999936 -6.24920000000008
64.1262000000036 -2.05379999999943 -7.8159000000001
43.5172000000042 6.97990000000067 -5.07790000000013
72.8228000000033 2.8932000000005 -6.01050000000013
74.8983000000032 4.44800000000048 -6.17830000000013
79.3730000000031 -2.35679999999949 12.5614999999998
99.2727000000023 15.9946000000004 -12.6513000000001
88.4124000000026 7.29830000000038 -1.13850000000016
90.7449000000025 3.46850000000041 -5.75030000000012
100.300500000002 -6.66669999999957 -4.87530000000009
100.242000000002 4.36660000000035 -10.4458000000001
116.164700000001 0.482800000000317 -6.50040000000006
122.6841 10.3505000000002 -8.56490000000002
126.3472 13.3738000000002 -8.54690000000003
126.9995 20.9541000000002 -11.301
132.9726 21.8398000000002 -2.44600000000009
130.0513 10.0711000000002 -5.98820000000002
130.7985 16.0331000000002 -11.8564
1.89620000000632 3.67790000000152 -10.6672000000001
4.01140000000616 2.22560000000146 -10.4922000000001
50.689500000004 20.4425000000006 -5.52260000000017
60.0627000000037 22.0043000000005 -7.3547000000002
68.7086000000034 20.7045000000005 -5.98490000000018
72.3818000000033 19.5094000000005 -5.90330000000019
74.1776000000034 27.8535000000005 12.2631999999998
86.1018000000029 21.0653000000004 -1.30320000000019
86.5130000000029 26.0720000000005 -5.6145000000002
92.2357000000027 38.7175000000005 -4.9716000000002
95.7152000000024 27.2407000000004 -10.2212000000002
109.150800000002 36.5108000000003 -6.62170000000013
119.553500000001 28.9427000000003 -9.0330000000001
123.969300000001 27.2767000000002 -8.43500000000006
126.225100000001 31.9550000000003 -6.48020000000007
130.112900000001 26.2060000000003 -11.6466
-0.218099999993496 0.555000000001546 0.0271999999999533
2.78040000000662 4.94410000000153 -0.0202000000000829
40.5993000000049 14.2033000000009 13.2437999999998
66.3637000000035 25.6026000000005 -0.626500000000213

61.2094000000038 25.5710000000005 -11.2633000000002
44.4856000000048 7.68290000000087 16.8992999999998
95.3591000000025 15.9656000000004 24.2018999999998
114.429100000001 18.9515000000003 22.2068999999999
121.440800000001 38.5102000000003 1.78209999999986
131.510700000001 22.0741000000003 13.0413999999999
128.345000000001 29.6045000000002 -3.43080000000007
-0.0991999999934983 -1.12129999999846 -0.00830000000004011
4.48300000000633 -3.95169999999848 0.253999999999965
42.1195000000047 -0.935099999999129 12.7435999999999
71.4604231896884 -3.52997959740375 -1.36626814675212
65.7619000000034 -4.3143999999994 -11.3749000000001
126.927012951143 3.05617791221588 2.92187115014598
130.3795 11.9407000000002 -3.36710000000004
ID=TAMtetUNKBRAAMNH133506

LM3=54
2.97640000000393 -3.04279999999957 -9.29219999999993
4.87600000000087 -0.443800000000082 -8.39939999999996
45.3496000000005 -3.03680000000003 -5.4774999999999
53.4207000000014 -1.12069999999992 -6.25880000000006
39.1780000000001 6.61389999999978 -5.50799999999998
61.9486000000007 1.98860000000021 -4.91670000000007
65.4123000000009 3.7878000000001 -5.52320000000001
67.9400000000004 -1.2748999999999 12.2053999999999
82.9047999999984 13.2264999999998 -8.80370000000001
75.5350000000001 5.86160000000016 -0.338300000000116
79.0835000000003 3.66230000000024 -4.15010000000011
88.6861999999993 -5.98229999999994 -4.48290000000006
89.5826999999999 3.83140000000007 -8.40910000000012
105.0493 -0.00949999999988288 -6.76380000000016
111.3253 9.49950000000005 -8.84170000000013
115.186199999999 12.9619 -8.06620000000012
115.3248 19.3570000000001 -10.0819000000001
121.1537 20.5535 -0.370700000000141
118.3612 8.09730000000008 -4.52840000000014
121.0679 14.7558 -10.8798000000001
1.84340000000172 4.27860000000003 -8.8197999999999
3.98130000000127 1.71309999999995 -8.44649999999994
41.7783000000005 17.2997 -4.87399999999996
50.3297000000003 18.7861999999999 -5.84439999999996
59.3518000000004 17.616 -4.72749999999999
63.5254000000004 17.4916 -5.22590000000001
64.7963000000003 23.6716 11.2656
73.1949999999999 18.4854 -0.185300000000031
76.3726999999997 22.2817999999999 -3.23570000000002
81.9742999999997 34.0530999999999 -3.48750000000003
85.8312999999993 26.0252999999999 -8.37600000000001
99.0317999999998 33.9697999999999 -6.44310000000005
108.6385 27.1883 -8.17490000000011
113.1271 25.9195 -7.4609000000001
115.1096 31.0195 -5.23760000000012
119.2808 25.3926 -10.8399000000001
-1.5749999999977 0.261600000000218 -0.623299999999952

-0.477499999998186 3.58270000000012 -1.05319999999995
34.3842000000003 11.0661999999999 11.8168
55.1241000000004 22.1040999999999 -0.423499999999981
51.0316000000004 21.9196999999999 -7.77979999999997
36.4180000000004 6.14629999999994 12.3969
83.07 13.9509 21.3187999999999
104.5024 17.8624 18.5063999999999
109.1236 34.9191 2.65369999999991
118.5546 20.6521 12.4746999999999
114.4488 29.6635 -2.03370000000012
-1.26039999999762 -0.687099999999737 -0.820599999999946
0.740000000002317 -3.35829999999974 -0.651899999999982
36.1190000000005 0.650399999999972 11.9878
58.7333000000005 -3.01929999999991 -0.0164000000000062
55.3182000000013 -4.53159999999973 -8.56319999999996
114.3967 3.14090000000008 2.55279999999987
118.215799999999 10.6553 -1.04300000000013
ID=TAMtetFEMBRAAMNH133508

LM3=54
3.20809999999685 -3.1746999999995 -10.4257000000001
3.77380000000099 -0.900499999999195 -9.89250000000001
52.5926000000003 -5.06119999999947 -4.4355
58.5344000000008 -3.68139999999932 -4.90920000000004
45.767200000001 6.40370000000058 -3.04170000000018
69.1243999999999 2.23760000000047 -3.22550000000003
69.7487999999999 3.12910000000043 -3.76250000000008
75.1035999999996 -1.72949999999961 14.6595
90.5104999999993 12.9093000000001 -9.25570000000015
83.0872999999998 6.4357000000003 0.347899999999974
85.4752999999999 2.50320000000032 -3.12360000000014
94.2950999999986 -7.02899999999983 -4.85540000000005
97.4299999999993 2.62260000000015 -9.00650000000011
111.173799999999 -0.0202999999998907 -5.13560000000008
119.545999999999 8.27360000000005 -6.46280000000009
122.707099999999 10.9496 -6.32590000000013
123.452499999999 18.3235000000001 -9.21340000000014
128.470499999999 19.1735000000001 1.23779999999991
126.130699999998 7.1657 -2.5275000000001
128.463499999998 13.1668 -9.00240000000012
2.16570000000002 3.60780000000081 -10.5362999999999
3.80890000000061 2.34150000000086 -9.91859999999995
48.7131000000012 18.7852000000006 -4.00420000000012
54.5364000000011 19.2744000000006 -4.28230000000011
66.3489000000008 15.8103000000005 -2.9389000000001
67.2826000000006 15.0172000000005 -3.2093000000001
72.3422000000004 21.0868000000005 14.7476999999999
80.2193000000001 16.7879000000003 0.6652999999999
82.4910999999999 22.0157000000002 -3.21650000000011
87.6755 33.1549000000003 -4.40630000000015
93.6376999999994 25.7203000000001 -9.32060000000013
106.059699999999 33.2540000000001 -4.90120000000014
116.631599999999 27.3212000000001 -6.76670000000013
120.354599999999 25.5834000000001 -7.02100000000014

122.487499999999 29.9087000000001 -3.70770000000014
125.928199999999 24.8820000000001 -9.97050000000013
-0.460900000000678 0.708100000000782 -0.265199999999906
1.6330999999999 4.18520000000084 -0.0575999999999206
36.5824000000011 9.98980000000074 12.3633
63.0339000000009 21.0135000000005 1.27269999999989
57.705900000001 22.4948000000006 -7.5652000000001
40.5545000000008 4.99010000000071 14.6684
92.4912999999995 13.1467000000003 22.8762999999999
110.321799999999 16.2989000000002 19.8776999999999
115.632399999999 33.7248000000001 4.23629999999986
124.918999999999 18.9682000000001 14.7010999999999
122.595299999999 27.9325000000001 -0.541500000000123
-0.235000000000822 -0.717099999999207 -0.322999999999884
3.05079999999923 -3.4724999999992 -0.196099999999899
38.4259000000007 -1.15509999999931 12.2652
66.3476000000003 -3.89939999999953 0.439599999999927
61.9007999999996 -6.11829999999948 -7.86869999999997
120.491599999998 0.956100000000065 4.52789999999993
125.604599999998 8.77870000000004 0.626399999999902
ID=TAMtetFEMCOLAMNH136242

LM3=54
3.04759999999542 -3.91839999999969 -11.8359000000002
4.79719999999607 -1.0058999999996 -10.9521000000001
55.7225999999976 -2.95679999999982 -4.90869999999997
62.6428999999976 -1.3136999999999 -5.24709999999998
47.0627999999973 7.94060000000016 -3.34679999999995
70.7297999999978 3.56980000000009 -3.44509999999998
73.6264999999976 5.37680000000004 -3.76619999999998
75.8426999999983 -1.46479999999982 14.5664
91.4462999999979 15.2032000000001 -9.92640000000006
82.9613999999985 7.83950000000016 1.92739999999996
87.4357999999984 3.92220000000012 -2.74760000000005
97.5045999999981 -6.35439999999984 -3.18310000000012
97.6920999999981 4.80360000000016 -7.6841000000001
114.949099999998 0.533000000000226 -5.57240000000018
121.667999999998 9.76970000000026 -8.30200000000021
125.627299999998 13.2413000000002 -6.69210000000025
124.857399999997 20.8803000000002 -10.0855000000002
131.639199999998 21.0678000000002 -0.780700000000266
129.015699999998 8.89290000000024 -4.90820000000026
131.007399999998 14.4051000000002 -9.56650000000026
1.48839999999555 4.93970000000042 -11.9853000000001
4.4451999999959 3.27950000000044 -10.8350000000001
52.0617999999972 20.4917000000002 -4.71829999999998
58.9736999999975 21.4643000000002 -5.25789999999999
68.1439999999978 19.6714000000002 -3.85329999999998
71.0414999999977 19.0583000000001 -3.91999999999999
70.1203999999982 25.8144000000003 14.5219
80.956099999998 19.0529000000001 1.24669999999999
83.8622999999979 24.5580000000001 -2.60640000000003
90.2604999999974 36.8974000000001 -3.59900000000005
94.0393999999977 25.7389000000001 -8.40330000000008

108.198099999997 35.9574000000002 -5.61180000000014
118.042499999998 29.7746000000002 -8.24920000000019
122.112099999998 27.8782000000002 -6.76410000000021
125.287299999997 32.6169000000002 -5.79150000000024
128.579999999997 28.5062000000002 -9.71090000000024
-0.824000000004348 0.290900000000473 -0.31590000000011
1.1969999999957 4.00820000000044 -0.502400000000088
35.5159999999974 12.1745000000003 12.2714
63.8825999999978 23.9146000000002 1.35000000000003
59.9863999999974 24.2400000000002 -7.5663
44.8418999999978 7.29760000000031 16.9978
92.5411999999979 15.2262000000002 24.6752999999999
113.928099999998 18.3445000000002 21.0984999999998
118.280299999997 37.7691000000002 1.7936999999998
130.165999999998 21.5989000000003 12.6451999999997
125.179599999997 31.1147000000002 -1.95830000000024
-0.805300000004286 -0.746599999999509 -0.321700000000105
2.10409999999592 -3.54309999999952 -0.46020000000011
37.7593999999976 -0.819199999999722 11.8147
68.0090999999981 -1.85149999999987 1.37190000000004
64.0250999999977 -4.08549999999989 -7.9261
124.174699999998 1.76290000000021 2.71039999999977
128.294199999998 10.0099000000002 -1.97690000000026
ID=TAMtetFEMCOLAMNH136243

LM3=54
0.864154135432583 -3.96222221450749 -11.5167776150115
2.1552459937814 -1.79572013464911 -11.0611425886391
52.7398999999956 -4.65560000000034 -8.34169999999981
60.8447999999963 -3.27030000000051 -8.7574
47.8888000000002 7.27510000000014 -6.7669
68.8417000000005 2.23300000000001 -6.33650000000004
72.0326000000014 3.92370000000013 -6.03200000000005
76.9676000000002 -1.76459999999993 11.8372999999999
95.6443000000016 14.3523000000002 -11.8227000000001
84.4124000000021 6.72780000000033 -0.854500000000091
88.5026000000012 3.68510000000021 -3.69040000000003
98.7244000000014 -5.47179999999974 -5.03800000000004
100.518800000001 4.36200000000013 -10.4043000000001
114.500300000001 0.0375000000001974 -7.03550000000007
121.853800000001 9.64510000000016 -8.56140000000008
125.262800000002 12.7532000000002 -7.05190000000011
123.766000000002 19.6784000000002 -9.43130000000012
131.047100000002 21.3768000000002 -0.364700000000114
128.455400000001 10.1661000000002 -4.64400000000011
130.533800000002 15.4923000000002 -10.8041000000001
-0.272099999999064 3.75059999999997 -12.0826
1.7776146839382 2.04507827492098 -11.2743197957992
49.3483000000011 18.8227000000001 -8.54990000000009
57.1546000000013 20.6105000000001 -9.39250000000012
66.6333000000016 17.4025000000001 -7.08220000000013
69.5453000000016 16.5065000000001 -6.44100000000014
68.9759000000012 22.5683000000001 12.5350999999999
82.2887000000018 18.2113000000002 -1.28950000000013

85.1562000000019 22.2167000000002 -3.69400000000012
91.721200000002 34.5122000000002 -5.52960000000012
95.603800000002 25.5636000000002 -10.7004000000001
108.954000000002 35.2918000000001 -7.96810000000013
118.606100000002 28.7247000000001 -9.3433000000001
123.483700000002 26.9531000000002 -7.07470000000012
126.385300000002 31.1512000000002 -3.86410000000014
128.802000000002 25.9985000000002 -10.8479000000002
-0.963799999999365 0.720099999999954 -0.184600000000014
1.06290000000075 4.11479999999996 -0.389900000000018
35.9916 12.3609 8.21800000000002
63.3858000000014 23.3772000000001 -1.80720000000013
58.2423000000017 22.5687000000001 -10.9815000000001
41.5132999999995 5.75609999999997 12.2099
91.5818000000009 13.5798000000001 21.6647999999999
113.901900000001 17.7038000000001 18.8820999999999
119.787300000002 36.8680000000001 2.19779999999986
127.641700000001 20.6014000000002 13.0851999999999
126.029800000002 28.5898000000002 -1.76290000000012
-0.857999999999404 -0.797000000000048 -0.554000000000025
1.96550000000048 -3.46630000000007 -0.277000000000008
38.329299999999 -1.56750000000008 8.46370000000009
67.2852000000001 -4.57639999999995 -2.11729999999998
62.8116000000008 -5.07309999999984 -10.3202000000001
126.244200000001 2.7071000000002 2.50229999999992
128.119800000001 12.7276000000002 -1.19910000000009
ID=TAMtetFEMCOLAMNH142249

LM3=54
4.13220000000673 -3.0079999999987 -9.85780000000044
5.13420000000629 -1.06079999999887 -9.27850000000025
54.6286000000038 -2.24409999999959 -5.07420000000035
62.8239000000034 -0.823899999999665 -5.43720000000035
49.1317000000036 8.07270000000046 -2.97600000000035
71.2103000000029 3.20170000000023 -3.31140000000035
75.3023000000029 4.89610000000022 -3.53830000000036
76.2882000000038 -1.70389999999967 14.1263999999996
96.0379000000032 14.3785000000004 -11.5003000000004
85.3582000000027 6.07700000000019 1.74199999999963
90.1700000000031 1.85990000000025 -3.28460000000038
96.9574000000041 -6.0179999999996 -3.6850000000004
99.2246000000037 2.95200000000037 -8.3683000000004
113.727300000004 -0.213699999999437 -6.36130000000039
121.606900000005 8.93670000000066 -9.26240000000041
124.461100000005 11.5757000000008 -10.0492000000004
124.408800000005 18.6919000000008 -12.5436000000004
131.463500000005 19.5843000000008 -3.35090000000045
127.933500000005 7.77550000000077 -6.82820000000043
129.433800000005 14.0455000000008 -13.7198000000004
2.77010000000656 5.07330000000117 -9.10340000000031
4.38800000000619 3.54070000000111 -8.71010000000028
52.9088000000034 18.8719000000004 -4.12410000000038
60.2868000000031 20.0505000000004 -4.9519000000004
69.1433000000026 17.9523000000002 -3.07140000000038

73.1341000000028 17.0329000000002 -3.30140000000038
73.8399000000037 23.5276000000004 15.0179999999996
83.6665000000029 18.5209000000002 2.05059999999959
86.001800000003 23.4039000000003 -1.9806000000004
91.0212000000034 34.3811000000005 -2.93250000000042
96.2744000000034 26.2388000000004 -8.2958000000004
109.035400000004 33.9232000000006 -6.00310000000042
118.935000000004 27.2832000000007 -9.11880000000042
122.501100000005 25.4391000000008 -9.72090000000043
125.085900000005 30.2518000000008 -6.95670000000045
125.904700000005 23.9139000000008 -13.8224000000004
-0.58249999999323 0.120300000001198 -0.339700000000317
0.860300000006572 4.36990000000114 -0.16320000000033
38.3523000000046 10.6795000000006 13.7871999999996
65.5116382692695 22.3985231928809 1.7181183771407
61.9627000000031 23.1182000000004 -7.5007000000004
39.4558000000047 5.4827000000006 15.9007999999996
94.5091000000042 14.7304000000005 22.3809999999996
117.059700000005 17.5513000000006 16.4443999999995
118.893900000005 34.9412000000008 2.79089999999953
129.469300000005 19.7694000000008 10.4127999999995
125.235400000005 28.5041000000008 -3.23000000000046
-0.613699999993218 -0.538499999998811 -0.369100000000335
2.09520000000657 -3.98909999999881 -0.760200000000312
40.6910000000047 -0.0462999999993905 13.0128999999996
68.9222683532121 -2.47310983378573 0.808307749484037
65.8530000000035 -3.73069999999966 -8.53220000000035
123.217600000005 0.826100000000701 2.19539999999955
127.703900000005 9.24080000000076 -3.45310000000045
ID=TAMtetUNKBGUAMNH142987

LM3=54
5.42060000000146 -2.61189999999931 -9.41220000000014
6.56080000000086 -0.283999999999648 -8.90790000000043
56.5617000000005 -3.19060000000017 -4.64279999999998
60.3434000000002 -1.64320000000027 -4.87079999999992
48.7745000000005 8.51189999999989 -3.54870000000006
73.7829000000001 4.60239999999972 -3.05459999999997
75.3364999999996 5.18739999999965 -3.52899999999996
77.8466999999995 -0.830300000000349 15.4449000000001
93.2883999999987 15.3200999999997 -8.86330000000012
84.1289999999994 7.24149999999964 1.74460000000004
87.2901999999992 3.55929999999967 -1.27849999999993
96.6134999999988 -5.58980000000026 -3.5452999999999
97.4444999999987 4.61699999999978 -8.34009999999999
112.308699999998 -0.117000000000174 -6.8178
119.395699999997 9.8218999999999 -8.44420000000013
123.652299999997 13.1459 -7.96670000000018
122.433299999997 19.7936 -11.2489000000003
129.503899999997 20.3823 0.0786999999997193
127.683699999997 9.59260000000002 -5.98460000000016
127.775599999997 14.6306 -11.9559000000002
3.73360000000076 5.46290000000037 -9.06760000000042
5.15390000000072 3.35930000000035 -8.12670000000039

53.0893000000003 21.2636999999999 -4.79560000000021
57.0609999999997 20.6840999999998 -4.83000000000021
71.2695999999996 19.5234999999998 -3.01030000000016
73.6216999999994 18.9198999999997 -3.49400000000016
73.4836999999991 25.3058999999997 15.5814999999998
82.2606999999988 19.7751999999996 1.79299999999985
83.8151999999987 23.8946999999997 -1.24320000000022
90.5382999999983 35.2893999999997 -3.76640000000035
94.2459999999982 26.2703999999997 -8.08890000000026
106.249299999998 34.5602999999998 -6.61490000000039
117.369199999998 28.0959999999999 -8.87920000000037
121.709599999997 26.0318 -8.58790000000037
124.353099999997 31.1385 -7.0559000000004
126.222599999997 25.7217 -12.2189000000004
-0.960699999999227 1.58970000000036 -0.0156000000004105
0.728400000000854 4.67300000000039 0.180599999999608
38.5537000000005 12.5763 13.2899999999999
62.6789999999995 24.7608999999998 1.82249999999979
57.0184999999998 23.6408999999999 -6.50230000000025
32.9622000000007 5.35739999999996 12.5215
90.7522999999987 14.8694999999997 24.2059999999999
114.302499999998 18.1960999999999 20.7830999999998
115.642099999997 36.6899999999998 3.07219999999961
127.155999999997 20.4056999999999 12.3778999999998
124.494699999997 28.7637 -2.78470000000037
-0.814099999999235 -0.916199999999613 -0.325900000000361
1.96610000000073 -3.48109999999963 -0.167400000000343
40.2718000000006 0.644599999999942 13.1353
66.9649000000001 -3.4381000000003 2.7970000000001
62.099324220127 -3.51206138210056 -7.82989623814045
121.751399999997 1.6913999999999 2.96629999999994
126.280899999997 12.1537 -2.34640000000016
ID=TAMtetFEMVENAMNH16935*

LM3=54
3.11619999999574 -3.82560000000105 -10.276399999999
4.1771999999959 -1.8774000000008 -9.80799999999912
49.4106999999974 -2.89850000000083 -4.81619999999953
55.5112999999978 -2.18560000000076 -5.55449999999958
43.1343999999972 7.32819999999917 -4.51029999999948
64.3937999999979 3.37979999999927 -4.56249999999961
66.3682999999979 5.39169999999928 -4.39689999999965
69.892599999998 -2.2295000000007 12.9436000000004
84.0813999999995 14.3668999999996 -10.2547999999997
76.2813999999984 6.77709999999937 0.335600000000329
79.8315999999988 3.13079999999945 -4.11329999999968
88.8816999999996 -4.44250000000041 -4.79839999999968
90.6490999999997 4.66739999999964 -7.9739999999997
102.9761 0.920399999999744 -6.32979999999967
109.951300000001 9.3267999999999 -7.65569999999968
113.677400000001 12.2703999999999 -6.92649999999965
113.773900000001 20.1149999999999 -9.34519999999962
120.492 21.4038999999999 1.76740000000043
117.493200000001 7.94619999999986 -4.45769999999962

118.417900000001 14.5972999999999 -10.5288999999996
1.52969999999578 3.52309999999915 -10.5083999999991
3.63039999999555 2.06519999999911 -9.99389999999912
46.1263999999976 18.8504999999993 -5.18459999999949
51.8687999999979 20.2153999999992 -5.84479999999955
61.366599999998 17.0098999999993 -4.0961999999996
65.074799999998 16.8792999999993 -4.17269999999961
64.0301999999979 24.6378999999992 12.8755000000004
73.4796999999986 18.6733999999994 -0.0485999999996551
75.8797999999989 23.3236999999994 -3.35949999999965
82.106499999999 34.3184999999995 -3.98619999999958
86.4588999999996 26.6702999999996 -8.42959999999964
97.6168999999999 35.0878999999998 -5.41549999999959
106.5495 28.8896999999999 -7.19339999999961
111.2436 27.1908999999999 -6.99449999999959
113.5143 32.8051999999999 -3.49069999999955
116.2606 27.2842999999999 -10.0030999999996
-0.868900000004561 0.363999999999095 -0.33359999999902
0.357111049623998 3.52268737136928 -0.743722405424344
31.8242999999972 11.2441999999992 10.3988000000006
56.3125999999979 22.2494999999992 -0.191299999999558
53.7614999999979 24.0773999999993 -7.66499999999956
31.2744999999971 5.75449999999919 12.5814000000006
82.9830999999983 14.3898999999994 21.1931000000004
103.142499999999 17.8100999999996 18.3558000000004
107.7159 35.2934999999998 2.77100000000047
119.4406 20.7402999999997 11.1996000000005
114.1556 30.2640999999998 -1.33769999999956
-0.55960000000456 -0.511600000000892 -0.245999999998996
1.32489999999553 -3.4416000000009 -0.386999999999059
34.059499999997 0.118699999999196 10.7887000000006
60.6699999999978 -2.80140000000075 0.802500000000381
58.8686999999979 -5.09710000000074 -7.4290999999996
113.6259 3.16089999999975 3.0203000000004
116.8891 10.2758999999998 -1.51109999999961
ID=TAMtetUNKVENAMNH16936*

LM3=54
3.5037 -2.4199 -9.8401
5.4239 -0.4645 -9.1797
52.2035 -2.5383 -3.7571
59.9237 -1.75 -4.545
44.8668 6.5568 -2.6742
65.6787 1.7576 -2.5015
67.8375 2.8271 -2.7127
72.6977 -1.4458 14.3517
85.7907 13.9487 -7.6025
80.328 6.3642 0.9374
82.8803 2.307 -2.3966
91.7154 -5.4419 -2.942
91.8871 2.8495 -7.6564
104.7366 -0.2574 -5.8048
112.9535 9.1997 -8.0671
115.7241 11.7065 -7.6932

115.6458 18.2785 -9.8909
121.5389 18.9082 -1.4968
119.0758 7.9676 -5.0467
121.1079 13.9749 -10.14
2.6438 4.625 -9.5762
4.5243 3.2844 -9.1357
49.0564 18.4519 -2.6731
56.2501 19.6003 -3.3016
63.7716 18.0394 -1.9801
67.7368 17.3595 -1.9355
67.6973 22.5433 14.6199
77.9852 18.5854 1.4439
78.855 23.3832 -2.1961
85.7819 33.6239 -3.2158
88.3917 25.9045 -7.8014
99.9844 33.5029 -4.8932
110.5495 26.8118 -7.8956
113.9195 24.3419 -7.9365
116.8527 29.4611 -4.8127
119.046 23.8013 -10.2035
-1.1381 0.4077 -0.7882
0.3836 3.3615 -0.4605
37.0604 11.8469 13.8575
61.5399 22.6333 3.4742
57.8771 23.3534 -5.5858
42.0471 5.7694 15.9689
90.3262 14.7586 22.5189
109.3443 16.6036 18.8806
111.7541 33.6343 2.2857
120.0705 18.8186 12.369
116.3134 27.2699 -1.6039
-0.8036 -0.5422 -0.5543
1.2068 -3.1517 -0.4697
38.6017 -0.9908 12.9093
65.2135 -3.4784 2.7453
62.1869 -3.9007 -6.6152
115.8699 1.7545 3.0179
118.3699 9.3724 -1.5064
ID=TAMtetFEMVENAMNH17561*

LM3=54
1.74850000000184 -2.88899999999925 -10.1533999999998
3.77319999999868 -0.633599999999958 -8.83489999999962
51.0853999999991 -3.17509999999991 -5.47829999999964
56.7083000000011 -1.91749999999975 -5.59039999999984
43.565499999998 7.69440000000003 -3.14999999999961
64.5866999999991 3.4174000000001 -4.14959999999969
67.1966 4.11930000000019 -4.19969999999982
71.4084999999994 0.768100000000146 14.1541000000002
90.2123999999992 14.9405000000001 -11.0392999999997
77.777799999999 6.69880000000015 0.317800000000239
82.458599999999 3.13750000000007 -1.99539999999979
92.4619999999991 -5.8510999999999 -4.5417999999998
93.6691999999993 3.09850000000008 -9.49929999999979

108.8315 0.000700000000140477 -6.90769999999983
114.3228 9.47550000000015 -9.20839999999983
117.9691 12.7085000000002 -9.00989999999983
116.6082 19.2482000000002 -11.7058999999998
124.7856 21.1341000000002 -1.92179999999981
120.9806 8.2812000000002 -6.40079999999982
122.708895945964 14.3537446276371 -12.1238262635884
0.991699999999158 3.88770000000025 -9.86979999999968
3.56379999999886 2.53030000000016 -8.41109999999966
47.6090999999984 20.3801000000001 -4.95589999999962
53.6159999999988 20.5048000000001 -5.07859999999962
62.4474999999992 18.3116000000002 -3.58629999999969
64.7536999999992 18.0456000000001 -3.68789999999971
69.0707999999991 22.4784000000002 13.9171000000003
76.4898999999992 18.4377000000001 0.293400000000287
78.8867999999992 23.3388000000001 -2.76759999999972
85.5232999999994 34.4949000000002 -4.06379999999971
88.8173999999993 25.9902000000001 -9.46349999999972
102.222 34.6414000000001 -6.00369999999975
110.9094 28.2498000000001 -8.86979999999979
115.0928 26.2799000000002 -8.84459999999981
116.1596 31.4819000000001 -6.45889999999978
120.094096792338 26.0697298306966 -12.2867378712538
-0.117899999999627 0.349700000000459 0.239800000000159
1.56189999999973 4.23200000000038 0.457900000000249
35.8863999999987 11.7704000000002 12.0282000000004
57.8043999999989 23.7583000000001 1.57690000000033
55.3375999999988 23.5002000000001 -7.08469999999962
35.4722999999989 6.4008000000002 13.2652000000003
87.8483999999993 14.0344000000001 21.3501000000002
108.8493 18.0778000000002 17.8352000000002
111.8254 35.4228000000002 1.21360000000026
123.1417 20.7531000000002 9.24380000000023
117.3942 28.7130000000001 -3.04699999999979
-0.370499999999456 -0.342299999999541 0.391200000000136
2.49060000000088 -3.1360999999995 0.356400000000096
37.5254999999991 0.935100000000225 11.8837000000003
61.6925999999997 -3.79749999999981 1.27790000000022
58.8393999999986 -3.86739999999997 -7.67539999999965
117.796 2.07530000000015 1.13700000000019
120.4908 10.7617000000002 -2.38339999999981
ID=TAMtetMALPERAMNH188191

LM3=54
2.8976 -4.1985 -10.7775
4.9315 -1.4992 -10.051
46.5576 -2.1487 -6.7285
55.6363 -1.1575 -8.2164
42.4262 7.0732 -5.6526
62.5043 3.4949 -5.6817
68.1265 5.9544 -5.8047
69.3945 -1.6299 11.6175
85.291 14.9784 -11.3412
76.8572 6.754 -2.2118

80.9635 4.2695 -6.1818
89.9426 -4.5296 -4.9499
92.6566 5.7286 -10.1277
103.672 0.766 -6.4211
112.3376 10.9238 -7.7705
116.527 14.2482 -7.5911
115.9711 20.9901 -10.3863
121.3311 22.7573 -1.5571
118.8579 9.9824 -4.0767
120.9437 16.257 -11.4152
1.6804 5.0252 -10.8615
4.6881 3.1692 -10.0567
42.9699 18.0482 -6.9484
51.4611 20.224 -8.2749
60.1314 18.5302 -5.9921
65.4718 18.5387 -6.1397
65.2125 24.9124 11.7835
73.8712 19.9603 -2.0324
76.6907 24.02 -6.7715
82.1911 35.7424 -4.6062
88.2734 26.7762 -10.4449
97.3893 36.1214 -6.2077
108.6161 29.6514 -7.6901
113.7809 27.3547 -8.4398
113.4981 32.5056 -5.0551
118.6718 27.0763 -11.5416
-1.0979 0.534 -0.5262
0.981 4.1625 0.0457
33.6425 12.6902 8.6458
55.5763 22.9179 -1.5404
52.5069 23.3003 -11.3985
34.1194 6.0532 10.2381
83.5646 15.025 21.4585
103.0361 18.8121 19.0147
107.8264 37.4745 3.6319
116.289 20.8039 13.0192
114.3672 30.8709 -1.8009
-0.7433 -0.6942 -0.3014
2.2434 -3.1911 -0.2236
35.7176 -0.4743 8.5477
60.5939 -2.7261 -1.2968
57.7979 -3.6717 -11.0728
114.6172 1.7852 3.3506
118.9311 11.3975 -1.7603
ID=TAMtetFEMBOLAMNH211660

LM3=54
3.10909999999951 -3.60600000000016 -10.3877000000002
4.70829999999949 -1.78740000000017 -9.79369999999998
52.4932999999994 -3.63250000000031 -8.2247
57.2370999999994 -1.59860000000027 -8.32510000000001
42.7470999999995 7.77339999999978 -5.65180000000003
66.7754999999995 3.10329999999977 -6.4594
68.9283999999995 4.42099999999977 -6.53810000000002

72.5555999999994 0.337499999999746 14.0848999999999
88.4513636716751 15.8904462668983 -10.4209032457564
77.8840999999998 7.11069999999985 -0.456400000000054
83.3404999999998 4.70439999999982 -4.27400000000005
92.3653999999997 -4.33440000000018 -5.35310000000005
92.8012 5.41669999999991 -8.63450000000003
105.9183 -0.186800000000122 -5.76320000000003
112.7431 10.0080999999999 -9.04760000000002
117.2874 12.9354999999999 -8.45320000000001
116.6321 20.8250999999999 -10.8943
123.673699999999 22.4975999999998 -2.22050000000005
121.529 9.79539999999986 -5.73790000000002
121.2795 16.1866999999999 -11.3834
1.38599999999928 3.99479999999989 -10.216
3.81659999999926 2.87269999999982 -9.42439999999997
47.5207999999997 21.9247999999998 -8.16320000000002
52.6258999999996 21.7101999999998 -8.27430000000001
62.9758999999997 20.5586999999998 -6.72170000000002
65.4421999999996 19.6982999999998 -6.58360000000002
69.0007999999997 25.1389999999998 14.3460999999999
75.0724999999999 20.8370999999999 -1.43350000000005
79.0865999999998 25.1430999999999 -4.21850000000002
85.2905999999997 36.2607999999999 -5.1441
89.4402519746268 27.0827185329728 -9.69028129337697
99.0179999999997 36.2516999999999 -7.2911
109.2406 29.6767 -9.39600000000002
113.872799999999 28.5748999999999 -8.63
116.785499999999 33.1046999999999 -5.47159999999999
119.207799999999 26.8494999999999 -11.4982
-0.446200000000774 0.475199999999858 0.0300999999999968
0.572299999999219 3.55739999999985 -0.447600000000016
34.9457999999996 14.0881999999998 10.1375
58.3591243717594 24.4539106047561 -1.23862736929869
53.2947999999997 24.5869999999998 -10.9373
39.1612999999996 7.15359999999976 12.5472999999999
84.2894999999994 16.0415999999998 23.3092999999999
107.568099999999 19.3843999999998 20.3959
110.264999999999 39.7957999999999 1.28629999999999
122.760899999999 22.5858999999998 12.3924999999999
118.208499999999 31.0502999999999 -2.62850000000003
-0.384900000000805 -0.769500000000172 -0.0526000000000067
1.70619999999922 -3.3819000000002 -0.405699999999999
35.5280999999995 1.51119999999977 10.3083
63.7568324177531 -2.83617018798517 -1.57447002669919
58.9086999999994 -3.80480000000025 -11.1543
117.870899999999 2.36739999999979 1.66599999999996
121.1975 11.5374999999998 -3.17480000000003
ID=TAMtetFEMBOLAMNH211662

LM3=54
1.89559999999989 -3.27620000000007 -11.7136000000004
3.8261999999996 -1.0619999999999 -10.4333000000005
53.9988000000002 -3.32470000000001 -6.49300000000023
63.4982 -1.86860000000009 -7.84390000000022

51.4595000000002 7.29749999999991 -3.88920000000015
68.8715999999999 2.43679999999995 -5.53770000000022
73.0064999999996 4.42279999999993 -4.57750000000021
73.2737 -2.18799999999991 11.5368999999997
93.8759999999991 13.5676000000001 -10.9538000000003
83.7755 6.63640000000009 -0.445000000000292
87.6917999999997 2.83090000000008 -4.01390000000033
97.8427999999991 -7.06729999999985 -4.34730000000041
98.148299999999 2.7199000000001 -9.96510000000038
111.779599999998 -0.997099999999757 -5.97450000000048
119.264499999998 8.19300000000035 -8.18420000000054
122.366099999997 11.5810000000003 -8.02910000000054
122.708499999997 18.0642000000003 -10.7059000000005
129.710999999997 18.9909000000004 -2.64420000000061
126.208099999997 8.83920000000033 -6.29430000000056
126.738799999997 14.1184000000004 -12.3049000000006
1.11539999999956 4.2667000000001 -11.5157000000004
3.24339999999982 2.75830000000013 -10.4855000000004
50.1459000000001 18.6418999999999 -5.26880000000018
60.3438000000002 20.5124999999999 -7.00740000000021
67.3790000000001 17.6793 -4.25490000000023
71.0157999999999 17.556 -3.95210000000024
67.7467000000003 22.8442000000002 11.7436999999998
81.1628999999997 19.1019000000001 -0.564500000000278
84.4361999999996 23.3050000000001 -4.15670000000028
91.415699999999 34.9087000000001 -4.44710000000032
94.7588999999989 25.6731000000001 -9.44470000000033
105.998299999998 33.5286000000002 -6.64240000000041
116.471099999998 26.9217000000003 -7.90410000000047
120.152399999998 24.9826000000003 -7.62670000000053
122.826399999998 28.7165000000003 -5.39370000000054
125.715699999997 23.6754000000003 -12.0695000000006
-1.0061000000003 0.520500000000142 -0.141500000000412
1.27209999999973 3.91720000000011 -0.251100000000372
39.8379000000005 11.3314000000001 11.2420999999998
63.8697000000003 22.6258000000001 -0.612800000000234
59.6123000000001 22.4563 -9.78070000000023
39.8291000000007 6.07690000000013 12.7593999999997
90.5132999999992 13.5700000000002 20.3820999999996
113.746399999998 16.6236000000003 16.8658999999995
117.579599999998 34.2108000000003 1.49349999999949
127.704299999998 19.1424000000004 8.99929999999937
123.548799999998 26.6187000000003 -3.08040000000055
-0.928900000000215 -0.714499999999827 -0.16630000000042
2.32099999999995 -3.22559999999985 -0.41160000000039
41.4210000000006 0.62870000000003 10.8710999999998
67.1047000000003 -3.16150000000003 -0.651900000000222
63.7342 -4.07070000000006 -10.3193000000002
122.479299999997 1.4921000000003 2.17709999999944
125.636099999997 10.8659000000004 -3.09710000000059
ID=TAMtetFEMBRAAMNH203374

LM3=54
3.5709 -3.0394 -9.6258

4.5373 -1.2031 -9.2132
50.5911 -2.8144 -2.6095
57.9097 -1.0115 -3.6755
45.8742 7.9683 -1.7545
64.2699 2.9338 -2.5313
67.0201 4.4282 -2.6152
68.8977 -0.0894 13.942
85.5282 14.6456 -8.9419
77.8274 6.0743 0.4242
81.1997 3.1223 -3.0888
89.0579 -5.589 -2.5277
90.9265 3.8349 -7.949
102.9361 -0.1444 -5.7677
111.1611 9.2555 -8.6365
113.9351 11.964 -7.6369
114.4269 19.482 -9.8991
120.5867 20.4684 -2.1533
117.6796 8.5897 -5.0852
118.5384 15.1157 -11.0215
2.5146 2.9314 -8.8336
4.1854 2.2078 -8.9283
46.6254 19.0923 -2.0944
54.5281 19.7601 -3.8938
61.8829 17.8334 -2.4028
64.6928 17.8002 -2.8473
66.4855 22.3396 14.0491
75.1886 19.2976 0.0718
77.4355 23.3778 -3.1213
82.3951 34.06 -2.7779
86.7111 26.0874 -8.3684
96.6282 33.9569 -6.093
107.3172 28.1927 -9.1013
111.197 26.1251 -7.5921
113.5451 30.987 -4.6468
117.0738 25.4672 -10.7941
-0.253 0.3583 -0.3783
1.2212 3.8027 -0.8759
35.8339 9.387 14.117
59.0841 21.6444 1.6981
54.8232 22.687 -5.3533
40.3154 6.2783 15.2366
84.604 14.2113 22.6073
107.5145 18.5972 18.3575
107.9927 35.7829 3.2161
118.0216 19.9782 12.4899
114.2713 29.0492 -1.608
-0.0915 -0.4087 -0.1108
2.2689 -3.2423 -0.4429
36.2474 1.7288 13.9025
62.563 -2.3597 2.0998
59.6417 -3.1675 -5.6319
114.296 1.3215 3.5086
117.2402 9.4176 -1.5372
ID=TAMtetFEMBOLAMNH246456

LM3=54
2.98229999999895 -4.04710000000066 -10.3627000000002
4.19699999999975 -1.37000000000064 -9.48290000000005
49.2766000000001 -4.56440000000076 -7.03079999999998
55.7111000000001 -2.98310000000073 -7.33709999999996
45.2238000000003 7.31739999999935 -4.97560000000003
64.5029000000001 2.2618999999993 -5.11669999999988
67.3864999999999 3.36659999999928 -5.24579999999985
70.0744000000001 -2.43720000000067 12.9734000000002
86.2013000000002 13.9809999999996 -9.51249999999978
76.7725000000004 4.92929999999945 -0.13869999999981
80.5884000000001 2.90939999999945 -3.98749999999978
90.0900999999996 -6.25620000000053 -5.17779999999975
90.4181999999997 4.06199999999957 -8.71669999999975
104.834299999999 -0.323800000000393 -7.50139999999969
111.662899999999 8.86979999999972 -9.69349999999967
115.485999999998 11.9655999999998 -9.36579999999964
115.745199999999 19.5560999999999 -11.7264999999997
122.357899999999 20.8688999999998 -2.39869999999964
119.958199999998 8.87239999999973 -7.06549999999964
120.759699999998 14.7704999999998 -11.9330999999997
1.4333999999996 4.22489999999937 -10.2815000000001
3.59309999999967 2.4762999999994 -9.33460000000014
45.3502000000004 19.0491999999994 -7.1114
52.6794000000005 19.3680999999994 -7.30249999999995
60.8267000000005 17.5523999999994 -4.9103999999999
64.9299000000007 17.0588999999995 -5.59959999999989
66.5493000000004 23.6756999999995 12.6866000000002
73.9585000000007 18.5058999999996 0.25720000000017
76.7812000000007 21.6779999999997 -4.13179999999981
81.9666000000006 34.5787999999998 -4.14149999999979
86.2220000000004 24.2486999999998 -8.5084999999998
97.7314000000002 34.1474999999999 -7.05769999999976
107.4308 28.0666999999999 -9.23429999999972
112.317999999999 26.5335999999999 -8.7779999999997
114.734799999999 31.1723999999999 -5.6691999999997
118.065399999999 25.9730999999999 -11.9661999999997
-0.670000000000471 0.646199999999399 -0.353100000000101
-0.195000000000414 3.55309999999944 -1.13600000000011
34.3142000000002 10.9779999999993 11.1612
56.9090936228241 21.8185681655844 -0.966187087743907
53.3951454122997 22.2473104274377 -10.1552857615984
35.4575000000001 5.22149999999933 13.8124
86.1081000000002 14.5246999999996 22.1009000000003
105.5855 17.9961999999997 19.1795000000003
107.8052 36.6417999999999 2.32040000000029
121.491999999999 21.0673999999998 11.4186000000004
116.557799999999 28.5271999999999 -3.03689999999967
-0.574000000000475 -0.704900000000642 -0.0733000000001213
1.43089999999948 -3.48010000000064 -0.628000000000114
35.9891 -0.310900000000711 11.4071
60.7345556725462 -3.96444434445269 -0.867032438890156
58.0890000000002 -4.98500000000074 -9.81679999999995

114.128399999999 0.828499999999646 2.06340000000035
118.924999999998 11.0782999999997 -2.62979999999962
ID=TAMtetMALBOLAMNH255862

LM3=54
4.5618 -3.208 -10.2857
5.755 -0.9072 -10.0781
48.8983 -2.592 -4.9448
58.1395 -1.8326 -7.2042
46.3023 7.9844 -3.7187
65.1115 2.6996 -5.3354
67.9821 4.0457 -5.1677
73.8754 -1.0529 12.711
87.2449 14.527 -10.2171
79.6786 6.9131 -1.3025
83.4274 3.0354 -5.55
94.3207 -5.9728 -4.8607
93.1677 4.4876 -10.8908
108.3682 -0.8412 -6.351
115.511 8.9047 -7.6947
118.5869 11.85 -6.8163
119.2059 19.5134 -8.8463
124.9315 20.1955 -0.0061
121.9964 8.1036 -2.8679
125.2814 14.5018 -9.8582
3.1415 4.8812 -10.4585
5.1663 3.2584 -9.9501
45.505 18.7682 -4.7795
54.628 21.4135 -6.6442
62.5518 19.3573 -5.1046
65.4429 18.4062 -4.8362
67.9895 24.9566 13.0752
77.8473 18.9277 -0.7119
79.7992 25.0498 -5.2954
87.4444 36.5021 -4.8498
89.9183 26.483 -11.2859
102.8861 34.9767 -6.6935
112.6702 27.3936 -7.211
116.5273 25.7333 -6.501
118.6869 30.4366 -3.5375
122.9014 25.4392 -9.6591
-0.4232 0.6853 -0.1676
2.0158 4.1669 0.0339
36.1504 13.9566 11.5892
60.6274 23.5701 0.3065
56.4989 24.2289 -10.6992
40.5232 6.6948 15.3934
88.0606 14.2872 21.4331
106.3489 17.1849 18.824
112.9244 34.7734 -0.853
121.4855 19.7139 14.3131
118.7302 28.7052 -0.3888
-0.6425 -0.6088 -0.294
2.8094 -3.533 -0.2573

38.0319 -1.8147 11.3121
64.0111 -2.5598 0.6448
60.4048 -4.8487 -10.9714
116.717 1.0641 1.4316
121.3848 10.2947 -0.1404
ID=TAMtetUNKBRAAMNH368***

LM3=54
1.6357 -2.7634 -9.1271
3.6977 -0.5364 -8.0721
49.6934 -3.3471 -5.4248
54.6211 -2.449 -6.1911
41.7435 6.4366 -4.8382
60.7167 2.8219 -4.8056
64.0165 4.0179 -4.9385
64.2707 -2.7635 11.3886
82.3838 14.0409 -10.1683
75.2906 6.5755 -1.2888
77.6806 3.536 -3.7666
87.196 -5.71 -5.156
86.7888 3.5892 -9.7124
101.8895 -0.4285 -6.1712
108.4243 8.521 -8.0066
113.151 12.3341 -7.624
112.9381 19.3534 -9.9479
118.5367 20.412 -1.0654
115.9558 8.6748 -4.3976
119.7736 14.6619 -10.3522
0.7726 4.648 -9.2406
3.3707 2.8469 -8.4264
46.0242 19.6845 -5.9051
50.8271 20.3497 -6.2257
58.1919 17.4826 -5.3984
61.7208 17.1609 -5.083
59.6227 22.5973 11.639
72.8968 18.0765 -1.0791
74.0568 22.0851 -4.2592
80.5065 33.7643 -5.154
83.257 24.9039 -10.3043
95.3332 33.3926 -6.9573
105.8068 27.3759 -7.4871
110.8175 25.4673 -7.3759
112.4115 30.2715 -4.2461
117.1226 25.4637 -10.4913
-0.6494 0.7136 -0.0584
0.567 3.526 -0.5896
32.7077 11.0115 10.3306
54.7027 21.8748 -1.7368
51.9595 22.2485 -8.7949
36.3131 5.5595 14.1533
81.7055 13.1567 19.1105
103.1181 17.5485 16.8495
107.2354 34.9904 0.6722
116.815 19.4575 11.3722

112.6595 28.6232 -0.6379
-0.6577 -0.4476 -0.147
1.402 -2.7233 -0.359
33.5893 -0.0065 10.5477
59.6237 -2.7257 -0.7709
56.8019 -4.2146 -8.5646
112.9266 2.2126 0.6716
116.2688 11.6445 -0.9211
ID=TAMtetUNKBRAAMNH370***

LM3=54
2.1678000000004 -3.7548999999997 -11.2695999999998
4.21499999999986 -1.87330000000009 -9.74819999999973
52.8030000000014 -4.74049999999989 -6.16419999999983
58.7957000000039 -2.67529999999953 -6.77750000000009
45.6458999999999 7.40979999999982 -4.44669999999995
67.9787999999996 2.93269999999977 -4.94779999999983
70.5487999999996 3.70509999999977 -5.3358999999998
73.4266999999994 -1.45260000000017 14.0808000000002
94.7885999999993 14.9631 -9.84229999999982
81.8317999999995 6.73140000000001 -0.0572999999998096
86.0442999999988 3.4832999999999 -4.16629999999976
97.648199999998 -5.47410000000011 -4.08409999999972
98.4964999999985 4.18979999999995 -9.47639999999977
112.346799999997 0.23319999999992 -6.42669999999973
120.519099999997 9.93449999999996 -8.26869999999973
125.726599999997 12.5687999999999 -6.96239999999972
124.975399999998 20.1469 -9.44519999999975
132.102199999998 21.621 -0.749199999999721
128.668699999997 9.49549999999996 -3.59179999999971
130.246699999997 15.6493 -10.9541999999997
1.56803608203132 3.98700250945067 -10.4606564816602
3.92075724405766 2.29453763684174 -9.74675391279786
49.2489000000003 20.3869999999999 -6.13369999999988
55.7045000000002 21.3287999999999 -6.77849999999987
66.2398 18.7021999999999 -5.25879999999985
68.4652 18.2348999999999 -5.33799999999984
72.9422 24.7171999999998 15.0258000000001
80.1451999999997 19.6705999999999 -0.208999999999826
82.8726999999996 23.3261999999999 -4.60419999999981
90.9777999999993 35.7964999999999 -4.2820999999998
94.7251999999993 26.3069999999999 -9.9243999999998
106.962399999999 35.0469 -6.93259999999978
117.648799999998 28.2238999999999 -8.50049999999976
122.438499999998 27.7529 -7.21119999999974
124.804799999998 31.6491 -3.49569999999975
128.303999999997 25.7239999999999 -11.0918999999997
-1.59999999999899 0.618600000000113 -0.498099999999881
-0.0902999999985777 3.93260000000012 -0.629799999999853
34.3779000000013 10.4953 12.0011000000001
59.0442000000001 24.5267999999998 -0.348599999999853
57.2118000000001 23.9678999999998 -9.47049999999984
39.2897000000013 5.78310000000002 13.5692000000001
91.0584999999991 14.1809999999999 22.0056000000002

113.591099999998 17.935 18.4810000000002
118.282299999998 36.3667999999999 1.90910000000024
127.574399999998 20.2666 13.2178000000003
123.845599999998 29.7127 -0.965399999999742
-1.344399999999 -1.3946999999999 -0.412899999999901
0.828700000001113 -3.65989999999987 -1.41529999999989
35.6792000000014 0.17620000000003 11.3653000000001
63.8520999999999 -5.43260000000016 -0.490599999999854
61.1702999999987 -5.24340000000034 -9.51869999999972
124.105199999997 3.04969999999995 1.46230000000026
127.340899999997 11.5828 -0.850099999999716
ID=TAMtetUNKBRAAMNH372***

LM3=54
4.3413 -2.663 -10.3809
5.265 -0.131 -9.0441
53.5089 -2.8986 -5.5888
60.1373 -1.1106 -6.105
45.6122 8.6389 -4.6753
67.8858 3.921 -4.4614
71.4873 5.1879 -3.8797
74.7552 -0.7551 15.2281
89.7087 16.1168 -10.0309
80.4834 8.2114 0.4987
82.3769 4.5092 -1.7392
90.9307 -5.8296 -3.4431
93.9626 4.7839 -7.5372
106.0964 -0.1517 -6.6653
113.9123 9.6553 -8.4077
117.6778 12.4171 -7.1924
118.1211 20.0136 -10.5208
124.4204 20.6586 -1.5969
121.5884 7.9148 -4.6807
123.2162 14.7856 -11.1191
3.0906 5.3578 -10.281
4.8189 3.0217 -9.5372
49.7414 22.3485 -5.3232
56.4427 23.1966 -5.7117
65.1025 20.8079 -4.605
68.5322 20.5104 -4.0301
67.5182 25.4309 15.3065
78.744 20.1624 0.5544
79.0858 25.0911 -2.4454
84.392 36.7992 -3.2984
91.0978 28.3836 -8.3424
101.3929 36.0381 -6.9526
112.132 28.7595 -8.0559
116.7495 27.0593 -7.5955
119.6114 32.1422 -3.8944
121.7182 25.5247 -10.6541
-0.6951 0.4983 -0.409
1.2759 4.1147 -0.7292
34.6734 12.2757 11.6974
59.5015 25.2665 1.017

56.1244 25.2111 -8.057
39.1144 7.1819 15.4289
88.4113 15.4776 23.1566
106.299 18.0082 20.6602
112.5863 36.1982 1.1753
122.0383 20.487 12.2311
118.5214 29.098 -1.0023
-0.3858 -0.63 0.0793
2.7239 -3.0376 -0.6588
36.3796 0.5175 11.5451
64.2021 -2.5545 0.0773
60.7288 -3.3695 -8.6842
117.3905 2.4861 1.0671
120.5629 9.9678 -1.6967
ID=TAMtetUNKBRAAMNH373***

LM3=54
1.61727819774263 -3.6542672297438 -10.7411623443981
2.43770000000091 -1.29460000000046 -10.1748999999999
51.7574000000014 -5.26479999999998 -5.34509999999997
59.8311000000012 -3.9193000000002 -5.94200000000017
47.8038000000011 7.49870000000001 -4.09849999999982
69.2010000000018 0.798300000000169 -4.97050000000008
72.6886000000014 2.95550000000015 -4.84560000000004
74.8090000000002 -2.50889999999999 14.5942000000001
91.0486999999994 14.0903000000001 -10.5217
83.2942999999989 5.94179999999995 -0.535599999999862
86.8620999999999 2.15690000000008 -3.88129999999989
96.6746000000002 -6.2924999999998 -3.01839999999992
97.7314999999987 2.5588000000002 -9.19289999999988
112.793899999999 -0.255999999999873 -6.28039999999987
120.339199999999 9.8848000000002 -9.85999999999985
124.260499999999 12.5128000000002 -9.11409999999983
124.569899999998 19.8909000000002 -11.8044999999998
131.153199999998 21.4453000000002 -1.04099999999982
127.229399999999 8.58020000000023 -5.54939999999984
129.265199999998 15.6108000000002 -12.3336999999998
0.823500000000666 4.74869999999968 -10.7806
1.76860000000071 2.70119999999966 -10.0368
47.1790000000007 20.2695999999999 -5.5040999999999
55.6207000000006 21.9558999999999 -6.21659999999992
66.0453000000005 20.0753 -5.65499999999995
71.0305000000002 19.2695 -4.77969999999995
68.5526999999998 25.0699999999999 14.4940000000001
80.8444999999995 18.1579 -0.288999999999915
82.7178999999993 23.2268 -4.66939999999991
89.367099999999 35.1978 -5.19969999999989
93.3390999999991 26.0526000000001 -9.87029999999992
106.390599999998 35.5147000000001 -6.52639999999985
116.958699999998 27.9944000000001 -9.74919999999984
122.106899999998 26.4819000000002 -10.1212999999998
122.502199999998 31.3101000000002 -6.05059999999982
127.876199999998 25.9876000000002 -12.6672999999998
-0.780699999999328 0.607399999999637 -0.360499999999974

0.503700000000654 4.11589999999965 -0.801999999999972
34.9755000000006 11.5557999999998 11.0570000000001
63.1150000000003 23.6848999999999 -1.38899999999992
58.7840000000005 25.7880999999999 -9.19569999999994
34.5452000000008 5.73039999999986 13.3975000000001
92.4907999999991 15.4807 22.6153000000001
113.379099999999 18.6981000000001 18.3119000000002
117.034699999998 37.6100000000001 2.96780000000018
126.764899999998 21.3887000000002 14.6138000000002
123.120399999998 30.9743000000001 -3.89909999999982
-0.486499999999289 -0.603400000000395 -0.0273000000000062
1.81020000000076 -3.34310000000036 -0.604900000000002
36.8226000000009 -0.182100000000128 11.4539000000001
67.9960000000007 -3.35630000000002 0.0440000000000008
63.6115000000011 -6.3793 -9.0105
123.764199999999 2.67710000000002 3.55780000000016
127.281899999999 10.1413000000002 -2.28879999999982
ID=TAMtetUNKBRAAMNH392***

LM3=54
3.41149999999818 -2.98000000000007 -9.46339999999995
5.43249999999416 -0.759800000000216 -8.71839999999994
58.2469999999991 -3.29859999999985 -5.49709999999988
64.2560999999971 -1.73910000000018 -6.20029999999984
48.5821999999993 7.46630000000002 -3.898
71.2410999999973 4.39899999999983 -4.78379999999977
73.5368999999977 5.33129999999987 -4.74849999999981
77.2865999999992 -0.199999999999853 11.9958000000001
95.1873999999984 14.5885999999999 -11.3136999999998
85.2515000000006 7.83780000000032 0.0703000000000747
89.1929000000002 3.61490000000027 -3.16569999999997
97.6809000000005 -4.84349999999972 -4.03119999999996
99.2582000000006 4.44040000000032 -9.18789999999995
111.283200000001 0.338000000000313 -6.13309999999992
119.146800000001 10.1327000000003 -7.6430999999999
122.890700000001 12.4324000000003 -7.09059999999989
122.407 19.7173000000003 -9.22669999999986
127.8554 20.8222000000003 -0.611399999999861
126.143500000001 8.84580000000038 -4.31159999999989
127.722100000001 15.3610000000004 -8.84819999999987
2.40729999999514 3.73799999999978 -9.11249999999986
4.61259999999451 2.48859999999972 -8.59329999999979
53.8912999999982 20.6042 -5.6882999999999
60.1812999999983 21.0476000000001 -6.42959999999989
69.048199999998 17.7869999999999 -5.24489999999987
70.7422999999981 16.9794 -4.90439999999986
71.7737999999985 22.9155000000001 12.3552000000001
83.1621999999987 18.8079 0.0455000000001307
85.6030999999985 23.6833 -3.08019999999985
90.9014999999982 34.4494999999999 -3.75619999999984
95.4387999999985 26.1496 -9.16549999999984
105.214799999999 34.0267000000001 -5.75939999999985
116.0925 27.1137000000002 -8.04369999999987
120.5593 26.2007000000003 -7.76729999999986

121.9964 31.0965000000002 -5.16599999999986
125.9375 25.9622000000003 -9.54399999999987
-0.914000000004006 0.521299999999817 -0.343499999999808
2.71519999999544 4.11179999999978 0.885500000000197
41.1183999999978 11.9493000000001 12.9817000000001
64.8842999999981 24.4788 1.00450000000013
61.857099999998 23.4022 -8.37759999999987
43.9884999999981 6.10930000000009 14.6851000000002
91.4179999999998 13.8731000000002 20.7659000000001
113.8036 17.6010000000003 18.1526000000001
116.461699999999 36.1018000000002 4.01690000000015
126.3385 19.8837000000003 11.9183000000001
122.3947 28.8369000000002 -0.993099999999868
-0.795400000003887 -0.540000000000184 -0.465299999999814
3.80449999999623 -2.95270000000022 0.329000000000218
42.5667999999983 -0.333499999999925 12.6790000000002
69.5483999999984 -3.83060000000004 0.0242000000001706
66.8176999999981 -3.50480000000011 -8.34549999999993
121.693500000001 1.68710000000037 2.9625000000001
125.198400000001 11.6498000000004 -0.131299999999886
ID=TAMtetFEMBGUAMNH42862*

LM3=54
4.93220000000287 -3.23359999999933 -9.48830000000014
7.18019999999971 -1.48180000000023 -8.47230000000002
55.2490999999996 -2.80270000000006 -4.86730000000003
60.2918000000006 -1.40759999999993 -5.29140000000023
47.2405999999991 7.55259999999981 -2.07890000000012
67.9109 2.99650000000024 -4.32080000000025
69.9299000000003 4.7714000000001 -4.26540000000012
73.4259999999999 -0.736299999999867 14.3527999999998
90.3446999999984 13.9283999999998 -10.2690000000001
81.6139999999999 6.54990000000017 0.932599999999764
85.1306000000004 2.32260000000026 -2.71300000000021
95.0141999999996 -6.15249999999983 -3.69670000000018
95.8385000000002 2.70650000000013 -8.2414000000002
110.630500000001 -0.00159999999975918 -5.50630000000025
117.636400000001 9.14100000000021 -8.47940000000022
121.1769 11.9683000000002 -7.21360000000019
121.353000000001 19.6038000000002 -9.86990000000019
127.5591 20.8307000000002 -1.90020000000023
124.828000000001 7.65970000000025 -3.69500000000024
127.286600000001 14.3740000000002 -10.5565000000002
3.7399000000004 4.16369999999996 -9.81800000000002
6.03339999999994 2.97919999999989 -8.97810000000005
50.4114999999994 19.7176999999999 -4.93070000000007
55.9590999999993 19.4716999999999 -5.11350000000008
64.8444999999996 18.2379 -4.30290000000011
67.5274999999997 17.2444 -4.11980000000011
68.0154999999996 23.9563 14.4106999999999
79.0903999999995 19.1438 0.691499999999866
82.3799999999992 24.5660999999999 -4.00170000000011
87.7000999999993 34.8122999999999 -4.31790000000008
91.6272999999991 26.6036999999999 -9.10800000000008

105.3203 34.8201 -5.12480000000011
114.5416 27.9625000000001 -7.81220000000014
118.218 26.3843000000001 -7.74080000000016
120.6787 32.1542000000001 -4.40690000000018
125.079000000001 26.6782000000002 -10.7253000000002
-1.11249999999899 0.171200000000176 -0.473400000000105
2.10960000000049 4.18620000000005 0.0300999999999094
37.0598999999993 12.6047999999999 13.6456999999999
60.8361999999995 23.6866999999999 0.0642999999999077
57.7840999999994 23.7955999999999 -8.84040000000007
41.2493999999994 7.0649999999999 16.7360999999999
87.3459999999999 13.7055000000001 21.6018999999998
111.4613 18.2460000000001 19.4108999999998
112.6334 37.4981000000001 3.00799999999983
125.684700000001 20.5915000000002 13.1101999999998
121.9364 30.2496000000001 -1.06160000000017
-0.997499999998917 -0.904999999999807 -0.128700000000117
2.80940000000101 -3.28409999999978 0.276499999999864
38.2653999999994 0.0428999999999279 13.8417999999999
65.4970999999998 -2.6837999999999 0.157499999999868
62.9617000000005 -4.21889999999971 -8.39060000000013
119.250100000001 0.857000000000242 2.47959999999975
125.432800000001 10.1985000000002 -1.07610000000024
ID=TAMtetFEMBGUAMNH42864*

LM3=54
1.6390999999982 -3.98399999999967 -10.4333000000002
2.99450000000143 -1.9259999999996 -9.48309999999991
53.4005000000001 -3.96469999999975 -4.32869999999997
57.3776999999999 -2.4088999999999 -4.77149999999996
45.7066999999992 6.73080000000022 -3.26259999999987
67.8829000000006 2.74810000000024 -4.28719999999999
72.1158000000002 3.64950000000011 -4.36820000000005
73.9502999999996 -0.937699999999943 13.9842
87.5453000000005 13.5438000000002 -10.6132000000001
81.5060999999987 6.68389999999983 1.08880000000007
86.1364999999999 2.78370000000002 -2.75029999999997
96.4688000000001 -6.44929999999999 -3.86429999999999
95.0487000000002 1.70550000000012 -9.71370000000003
110.8028 -0.58099999999997 -5.55400000000001
117.7784 8.8094999999999 -8.13249999999998
121.1494 11.3242999999999 -7.43
121.7248 18.8466 -9.16029999999999
127.5148 19.6441999999999 0.404900000000024
124.0293 7.39189999999993 -4.70599999999999
125.0932 13.4291 -10.8363
0.892000000001114 3.3631000000005 -10.8068
2.67350000000134 1.79350000000046 -9.58559999999995
49.0073 19.5686000000002 -4.12989999999994
53.9348 20.1060000000002 -4.88379999999998
65.4642000000002 17.0199000000002 -4.17920000000001
70.3066000000002 17.9013000000002 -4.37000000000002
69.6568999999998 21.8302000000001 14.1686
79.5308891363903 18.4562288264376 0.51464134765953

82.5476000000001 22.9400000000001 -3.13160000000002
90.2290000000002 34.8006000000001 -4.20099999999999
90.7890000000003 26.1817000000001 -9.90430000000002
105.5294 33.5495 -5.74759999999997
115.3012 27.1170999999999 -8.27459999999998
119.3398 25.1033999999999 -7.94079999999998
120.6252 29.6031999999999 -5.08619999999997
123.6404 23.5197999999999 -11.1417
-1.04250000000005 0.772900000000425 -0.153699999999925
-0.107899999999487 3.52810000000045 -0.397099999999947
40.1470999999996 12.2087000000002 13.5797
62.5343 23.3044000000002 2.75500000000001
55.9252000000002 23.3396000000002 -6.6807
41.1878999999995 6.58600000000017 15.9176
89.9300999999995 14.4272 22.1027
111.2423 17.1676999999999 18.0161
115.35 35.0333999999999 2.63480000000003
124.8701 19.4207999999999 12.7246
121.4664 27.1116999999999 -1.63749999999997
-0.765300000000215 -0.892999999999592 -0.204299999999994
0.716199999999509 -3.08789999999961 -0.478199999999918
44.9161999999994 3.93470000000018 15.935
65.9268999999995 -3.99529999999985 2.86320000000002
60.5715000000015 -5.44989999999955 -6.67600000000008
120.6076 0.930899999999949 2.69800000000003
123.5311 9.22949999999989 -1.39989999999999
ID=TAMtetFEMBGUAMNH42865*

LM3=54
2.4094 -3.9575 -9.4424
3.2471 -1.8198 -8.8521
51.3026 -4.2529 -5.5794
57.9294 -2.6717 -5.7888
42.9878 7.1283 -4.7471
65.785 2.559 -4.9813
68.1558 4.134 -5.0098
70.2609 -1.6684 13.6325
88.7903 14.2357 -10.887
79.2382 5.674 -0.8908
82.5552 2.6827 -3.4219
90.4701 -6.8733 -3.7246
92.9803 2.8657 -9.8941
105.2058 -0.6047 -7.6385
112.5338 8.4797 -8.6802
116.6712 12.3718 -9.6074
117.7488 18.9655 -12.0286
123.3847 20.3147 -1.4204
119.2216 8.1748 -6.2917
122.4665 14.1225 -10.9681
1.2796 3.5358 -9.6787
2.7658 1.7225 -9.1369
47.1182 19.9187 -5.9948
53.7484 21.1779 -5.9154
62.1506 18.4721 -5.1905

65.3 18.2718 -5.3218
66.4914 24.3867 13.0136
76.4271 20.3376 -0.8348
78.8263 23.2331 -3.718
82.9208 35.5198 -4.0875
88.5588 26.068 -10.4198
100.0817 34.3746 -7.2393
109.8556 27.416 -8.5617
114.2686 25.8572 -8.3461
115.8025 30.4605 -6.4922
118.9549 25.1236 -11.723
-0.8898 0.3542 -0.0924
1.1931 3.8251 -0.7679
36.2452 11.7189 11.3702
57.8328 23.6959 -0.7085
54.6361 23.3699 -8.9715
36.3633 6.2097 12.6791
87.7646 15.4324 22.0539
109.4513 18.0904 16.8007
110.8019 34.8094 1.1523
121.4256 19.8379 11.3661
116.6002 28.198 -2.5652
-0.6148 -0.7496 0.067
2.3893 -3.406 -0.386
38.0946 1.0115 11.8673
62.6325 -4.0066 0.3477
59.2866 -4.8049 -8.757
116.1737 2.2483 1.4406
119.6296 11.2686 -2.4895
ID=TAMtetMALBOLAMNH61795*

LM3=54
2.09790000000342 -3.6445999999995 -10.3945999999999
3.82370000000664 -1.47649999999956 -9.69259999999995
47.3319000000033 -2.82079999999982 -5.90379999999997
54.5319000000036 -2.05469999999959 -6.95219999999996
40.513500000004 7.63070000000024 -5.38010000000016
60.5145000000021 3.4413000000002 -5.0374999999999
62.527500000002 5.01050000000018 -5.5806
67.8958000000021 -1.09349999999981 12.3495000000001
81.4927000000013 14.4577000000001 -11.0632
74.7704000000015 7.12020000000017 -1.14569999999993
77.6618000000017 3.9942000000002 -5.1912
85.5716000000009 -6.18489999999987 -5.17969999999988
87.1836000000013 4.27830000000017 -10.0025999999999
101.672900000001 -0.0394999999998181 -6.67079999999986
108.848100000001 9.80600000000021 -8.84349999999985
112.383900000001 12.7662000000003 -8.00289999999987
112.680800000001 19.9865000000003 -10.8232999999999
118.560400000002 20.7316000000003 -2.43819999999985
114.570200000001 8.71130000000019 -5.08839999999983
116.636000000001 15.0258000000002 -11.7513999999998
0.529000000005826 3.54900000000039 -10.3085999999999
2.72020000000625 2.08890000000047 -9.78249999999995

42.8083000000041 19.2848000000003 -5.78400000000005
50.7771000000037 21.0782000000003 -6.65070000000003
57.5849000000029 18.1365000000002 -4.6829
59.8726000000026 18.2442000000002 -5.41539999999998
62.4946000000029 23.8539000000003 13.1759
71.786100000002 20.1141000000002 -1.26599999999997
74.2746000000019 24.5319000000001 -5.06179999999996
78.0476000000023 35.9753000000003 -5.12739999999998
83.6298000000015 27.4408000000001 -9.80169999999992
95.6103000000019 35.3348000000003 -7.13709999999995
105.628800000002 28.4514000000003 -9.26369999999989
109.999100000002 26.6115000000003 -8.10639999999989
111.976300000002 31.1625000000003 -4.9842999999999
114.813500000002 25.7467000000003 -11.0983999999999
-0.76249999999458 0.448900000000357 -0.260199999999909
0.0227000000058121 4.02720000000045 -1.27649999999993
33.7682000000049 12.1595000000004 10.7262
55.1972000000033 22.7770000000002 1.85579999999998
50.9244000000035 23.7947000000003 -9.03500000000003
33.6215000000046 5.61510000000034 12.3021
84.0914000000022 13.6247000000002 22.9199000000001
102.545400000002 17.3195000000003 19.4625000000001
105.010200000002 36.7519000000003 3.26810000000009
118.858000000002 20.2650000000003 12.1329000000001
113.085600000002 28.0169000000003 -1.5149999999999
-0.299799999994708 -0.479199999999618 0.00810000000009647
1.6573000000052 -3.23089999999962 -1.15699999999991
35.0900000000045 -0.365699999999678 10.1702
60.5745000000028 -1.0707999999998 0.987699999999994
56.0110000000023 -4.0983999999998 -9.52419999999987
110.971900000001 0.718600000000195 2.32760000000016
115.408000000001 10.4337000000002 -1.68229999999986
ID=TAMtetFEMBOLAMNH61793*

LM3=54
4.26054641699491 -2.49672212931032 -8.79098327341508
5.70700000000219 -0.05599999999898 -8.26160000000091
54.7311000000006 -2.61619999999991 -6.25360000000041
60.7291000000006 -1.78149999999991 -6.68670000000042
48.8299000000011 8.04620000000014 -5.28730000000049
69.4686000000005 3.60670000000001 -5.35710000000032
73.1323000000005 4.5835 -5.60690000000033
73.9377000000008 -0.924899999999918 11.8879999999996
93.1933000000005 14.6151000000001 -12.6044000000003
83.0403000000005 7.75539999999998 -1.2274000000003
86.3624000000006 3.30510000000006 -5.08020000000031
96.7563000000006 -5.03249999999987 -4.24310000000033
97.6887000000006 2.40110000000009 -10.1626000000003
110.401600000001 -0.562199999999751 -7.0601000000003
118.422900000001 9.50990000000028 -8.25170000000028
122.382000000001 12.0905000000004 -8.6156000000003
123.868900000001 19.4723000000003 -10.4872000000003
128.673600000002 20.2146000000005 -1.7034000000004
125.304000000002 8.87080000000042 -6.15100000000036

127.826800000001 14.2137000000004 -11.1961000000003
3.32864334659353 4.92733787352166 -8.78676149373456
5.44420000000222 3.25770000000062 -7.93750000000074
51.1657000000008 20.0899000000001 -6.52220000000046
57.3190000000008 21.0256 -7.22950000000042
67.2992000000005 19.2571 -5.88220000000037
70.7748000000005 18.2981 -5.63730000000036
70.2017000000008 24.4516000000001 11.6959999999996
81.0079000000004 19.1801999999999 -1.63930000000032
82.4876000000005 23.5904 -5.18020000000033
90.918500000001 34.7371000000001 -4.52190000000038
93.7655000000005 27.9144000000001 -10.4067000000003
105.667500000001 34.5890000000003 -6.70000000000038
115.797200000001 27.3127000000003 -8.84530000000035
119.406000000001 26.0898000000004 -8.11560000000036
121.779400000002 30.3754000000004 -4.86820000000039
125.697900000002 25.2938000000005 -10.8728000000004
-0.234599999997509 0.317100000000679 0.00719999999921356
1.85360000000238 4.22440000000067 -0.368400000000777
39.3437000000015 15.5513000000003 10.4902999999994
63.2107000000007 24.4931 -0.116900000000417
59.7485000000007 24.5900000000001 -9.49390000000043
41.1012000000015 7.39230000000027 13.1053999999994
93.9202000000014 15.1667000000002 21.0262999999996
110.496600000002 17.8515000000004 18.7573999999996
116.090800000002 36.0013000000004 2.61479999999957
126.384300000002 19.9639000000005 11.1028999999995
121.806800000002 29.0080000000004 -2.34740000000039
-0.109999999997531 -0.201699999999333 -0.182800000000792
2.60390000000243 -2.92469999999935 0.0492999999992418
42.2211000000015 -1.26569999999972 10.2133999999994
67.6317000000007 -3.45879999999994 -0.441900000000378
63.9232000000007 -4.28719999999991 -9.07480000000041
120.764400000001 1.34570000000033 2.83549999999964
124.788100000002 10.7356000000005 -2.72560000000036
ID=TAMtetFEMBGUAMNH64117*

LM3=54
4.45930000000148 -4.11579999999928 -9.54309999999992
5.74549999999647 -1.4698999999996 -9.20439999999974
55.6810000000005 -3.06709999999935 -4.40779999999991
62.6515999999984 -0.866299999999734 -4.88779999999986
52.6342000000008 8.70770000000071 -3.59949999999999
69.3446999999985 3.46750000000022 -2.8410999999998
72.4593999999989 4.99380000000027 -3.62229999999977
76.1958000000006 -1.10569999999949 15.5804000000001
94.0154999999997 15.1394000000002 -9.36339999999971
83.9664000000018 8.23780000000065 -0.0998999999998613
88.5714000000015 5.87870000000057 -3.81949999999991
96.3280000000018 -5.46399999999949 -2.65159999999987
97.7716000000017 5.57440000000055 -7.68919999999987
111.087200000002 -0.243599999999541 -4.27359999999978
118.589500000002 8.61490000000043 -7.57449999999973
123.280200000002 10.6408000000004 -7.41329999999969

124.139900000002 18.5310000000004 -11.0581999999997
129.997400000001 19.8156000000003 -1.40859999999965
126.547600000002 6.76350000000043 -4.7580999999997
129.084100000002 11.8384000000004 -11.5775999999997
2.52019999999772 4.31870000000052 -9.8079999999999
4.76079999999703 2.60290000000043 -9.49289999999985
51.9854999999994 20.1954000000006 -4.18209999999992
58.5327999999995 20.3526000000005 -4.47529999999989
66.7185999999992 18.4136000000004 -2.89119999999984
69.5691999999994 17.7245000000004 -3.40569999999982
73.901 26.7801000000005 15.3656000000002
82.7494000000001 19.7904000000004 -0.054899999999797
85.5386999999999 22.9448000000003 -3.04659999999976
90.6373999999998 35.2598000000003 -2.49429999999971
94.6850999999999 25.0347000000003 -7.8098999999997
106.3047 34.6550000000003 -4.03489999999968
116.833500000001 27.6245000000003 -7.76819999999967
121.349700000001 25.9445000000003 -8.79939999999966
124.196000000001 31.0555000000003 -5.13969999999965
126.984700000001 26.2001000000004 -12.0102999999997
-1.7652000000013 0.231200000000534 -1.56979999999989
-0.387300000001946 3.89570000000051 -1.11409999999988
41.0121999999995 13.3225000000006 13.5835000000001
62.4655999999993 25.7328000000005 0.433200000000158
60.4779999999992 24.9082000000005 -8.49039999999986
41.4237999999998 7.58300000000062 15.6388000000001
91.1149000000013 14.8262000000005 23.9331000000002
113.281000000002 18.3224000000005 20.8010000000003
117.457500000001 37.0576000000003 3.15800000000034
128.074400000002 20.1483000000004 14.4724000000003
125.241400000001 29.2530000000003 -1.75929999999965
-1.68930000000117 -1.25319999999947 -1.0260999999999
0.612699999998816 -4.5578999999995 -0.711499999999865
42.2318999999999 0.124000000000607 13.3670000000001
67.4500999999997 -3.69039999999961 0.58730000000018
64.9691999999994 -3.93299999999967 -9.03159999999992
121.617400000002 0.377300000000472 3.60920000000026
126.998900000002 9.84480000000042 -1.4163999999997
ID=TAMtetUNKECUAMNH66243*

LM3=54
1.43290000000112 -4.76749999999955 -10.8933999999999
2.84749999999817 -2.35449999999956 -10.0654999999996
54.2156999999992 -4.00749999999978 -6.79880000000005
66.7592999999998 -3.15909999999968 -8.50979999999994
49.7228999999981 7.29690000000004 -6.0306
72.6265999999987 1.73940000000009 -6.53549999999997
74.640399999998 3.59369999999997 -6.0751
78.777899999998 -0.637099999999959 12.399
96.173199999999 14.7067000000001 -11.8732000000002
85.7548999999984 6.03469999999995 -1.54100000000005
90.5604999999982 4.47339999999998 -4.87570000000009
100.547899999998 -5.21949999999992 -5.78290000000011
101.357999999999 4.52980000000011 -9.44770000000013

116.657399999999 0.18160000000019 -5.04460000000019
123.7236 8.44530000000028 -6.97370000000021
127.835699999999 11.1233000000002 -6.59480000000018
127.7769 18.2839000000002 -8.80330000000019
133.874999999999 19.0624000000003 -0.508200000000185
132.032599999999 8.40620000000026 -3.9300000000002
133.099099999999 13.7994000000003 -9.90090000000021
0.339399999998179 4.13970000000035 -11.1926999999997
2.14879999999805 2.04040000000035 -10.3385999999996
51.4475999999982 20.1006000000001 -7.34919999999994
63.7719999999981 22.7864 -8.84189999999995
69.9539999999981 20.0038 -6.62319999999999
73.1115225646397 18.705988168344 -6.37210443190333
77.1323999999982 24.2066 12.141
83.7605999999983 18.6979999999999 -1.18650000000004
87.6906999999987 21.9541 -5.04960000000006
95.6080999999989 33.8152 -5.39940000000007
98.6290999999991 24.4374000000001 -9.74590000000011
112.412599999999 32.8122000000002 -5.08070000000013
121.710499999999 26.1364000000002 -6.73130000000018
126.6706 24.2685000000002 -6.09790000000019
129.635399999999 28.2628000000002 -4.20180000000019
132.2847 23.5234000000003 -9.32520000000017
-1.3372000000008 0.496300000000497 -0.251699999999759
1.28429999999866 3.79420000000043 -1.0821999999997
41.5863999999983 12.9040000000002 10.9301000000001
66.2676999999981 23.9266 -2.26709999999996
62.8873999999981 24.9233 -11.7192
48.9664999999984 7.74040000000022 13.7629000000001
94.4147999999985 14.1574000000001 20.8960999999999
115.252599999999 16.6616000000002 18.6089999999999
119.630399999999 34.4050000000002 3.65669999999986
130.706599999999 18.9562000000003 12.4291999999998
128.129199999999 27.2350000000002 -0.987300000000168
-1.29580000000069 -0.877099999999495 0.0420000000002366
2.55129999999962 -3.62119999999949 -0.325899999999782
42.5686999999986 0.60560000000028 10.6984000000001
70.1356999999984 -3.57569999999992 -1.8329
67.8066999999967 -5.97760000000007 -11.5274
124.219199999999 0.380600000000264 3.95499999999981
130.509399999999 9.28270000000027 -0.895100000000199
ID=TAMtetFEMECUAMNH71818*

LM3=54
1.2196999999992 -3.03099999999972 -11.6036
3.33189999999861 -0.981900000000308 -10.2693
54.7938000000015 -3.39879999999987 -7.45079999999985
64.5531000000001 -2.34539999999999 -8.65489999999984
48.8742000000008 6.87740000000001 -6.24690000000003
74.0928000000008 3.76570000000028 -6.84299999999975
75.6421999999995 4.99659999999995 -6.92519999999977
79.4903999999999 -2.04199999999994 9.8296000000003
97.3407000000008 14.4111000000002 -12.4837999999997
86.8751999999998 6.20969999999999 -1.86679999999977

90.4488999999994 3.6 -5.63569999999972
98.600099999999 -7.1952 -5.34979999999967
101.1185 3.28420000000008 -10.8333999999997
115.392499999999 0.606100000000076 -7.34849999999958
124.1307 9.40110000000012 -7.36839999999958
128.085 11.7027000000002 -7.58809999999958
128.5755 19.4293 -9.13989999999955
133.2527 19.8064000000001 -1.35209999999954
131.4271 8.04410000000012 -3.87909999999956
132.8059 14.0334000000001 -9.67659999999955
0.374199999999912 4.70079999999987 -11.4350000000001
2.76909999999964 3.47479999999977 -10.1698
50.9789000000008 19.9208 -6.38869999999991
60.7411000000006 22.139 -7.9990999999999
72.5774000000002 18.4383 -5.84049999999983
73.6307000000003 18.2253000000001 -6.6081999999998
75.0957000000006 24.1669000000001 10.7815000000002
84.3236000000004 19.7102000000001 -1.46829999999974
87.7680000000007 23.6979000000001 -5.47739999999975
93.1285000000008 35.8106000000001 -3.34829999999972
98.3040000000009 26.3492000000001 -10.9833999999997
112.113800000001 33.0117000000002 -6.13349999999967
121.4769 26.6239000000001 -6.50059999999959
126.3164 25.3173000000001 -7.38329999999959
128.7254 30.0522000000001 -3.61179999999957
130.9171 24.6447000000001 -9.63329999999955
-2.1614000000004 0.489699999999918 -0.527199999999965
0.0266999999999045 3.69069999999988 -0.453099999999964
38.364700000001 11.6595 10.3963000000001
66.1008000000006 23.2815 -2.51449999999984
59.4828000000006 23.9487 -9.85679999999987
40.400700000001 5.20390000000006 12.6938000000001
92.5703000000003 14.0795000000001 21.4657000000003
114.8859 16.5611000000001 19.5731000000004
120.918500000001 35.0799000000001 2.0302000000004
131.2957 19.2742000000001 12.6751000000005
128.1183 28.5296000000001 -0.186499999999579
-2.07780000000058 -1.04950000000009 -0.732499999999947
0.981499999999109 -3.39900000000012 -0.981499999999923
39.650800000001 -0.864099999999939 10.0647000000001
69.4671000000004 -2.93489999999981 -2.82699999999976
64.125199999999 -4.29490000000001 -10.7366999999998
125.8894 1.56090000000009 2.37430000000045
130.7757 9.75130000000012 -0.104299999999561
ID=TAMtetMALECUAMNH71819*

LM3=54
0.557800000001479 -4.54049999999975 -9.70790000000006
3.02689999999703 -2.71850000000016 -9.26979999999973
51.4904000000007 -4.26779999999975 -4.04749999999998
62.6231999999987 -4.40140000000001 -5.16629999999993
47.045700000001 6.69800000000036 -4.22220000000004
69.1274999999987 0.797199999999926 -3.47619999999987
70.1721999999992 2.03229999999999 -3.64499999999986

76.6691000000007 -0.771199999999749 15.3599
91.8551999999997 13.2609000000001 -11.1363999999999
83.0448000000019 5.70900000000046 1.86629999999992
85.8872000000016 1.58760000000041 -1.9862000000001
94.7077000000018 -6.69849999999961 -2.0642000000001
96.3964000000018 1.46900000000044 -7.62780000000008
110.726700000002 -0.980099999999599 -5.04500000000009
116.583100000002 9.06110000000039 -8.99580000000007
118.875700000002 11.6383000000004 -8.66610000000007
118.776700000002 17.9042000000003 -11.174
125.498500000001 19.4628000000003 -3.44450000000006
123.461500000002 8.4999000000004 -7.16560000000007
123.966500000002 14.4504000000004 -13.3071000000001
-0.583500000001834 3.48229999999993 -10.2144999999999
2.20089999999746 1.90719999999989 -9.28029999999983
49.6072999999998 19.7338000000001 -4.88949999999997
58.0841999999998 21.3591000000001 -5.96029999999996
66.6412999999995 18.9283 -4.21509999999992
67.9727999999996 17.3071000000001 -4.26609999999993
74.3906000000002 23.2320000000002 14.7112000000001
80.6390000000002 17.8161000000001 1.62450000000004
82.2456 22.7954000000001 -2.71699999999994
87.6503999999999 33.6827000000001 -1.85859999999994
91.7426 26.2277000000001 -8.06749999999994
104.4794 33.4274000000002 -5.87499999999997
113.600800000001 26.4310000000003 -8.90510000000002
117.092900000001 24.7360000000003 -9.08890000000004
119.383300000001 29.3808000000003 -6.29540000000004
122.366800000001 24.5153000000003 -13.0050000000001
-1.85520000000088 0.592600000000017 -0.579199999999902
0.645099999998512 4.01479999999994 0.391000000000114
37.5398999999999 11.7610000000001 12.2012000000001
65.3641999999996 22.9536000000001 1.43260000000006
59.1900999999995 24.0703000000001 -7.65579999999994
39.1766000000001 6.21990000000017 14.3449
91.9456000000012 13.2745000000003 23.5365
111.978800000002 16.8156000000004 18.8625
115.445300000001 33.9045000000002 2.72940000000001
124.335200000002 19.6291000000004 11.5472999999999
119.713100000001 27.7446000000003 -4.72630000000004
-1.66210000000074 -0.883999999999978 -0.748899999999915
1.80459999999926 -3.80310000000003 0.340100000000126
39.6807000000003 -0.0203999999998264 12.5206
69.1435999999999 -3.00929999999992 2.18410000000009
64.1288999999996 -6.26959999999996 -6.76160000000001
121.184800000002 2.87290000000041 1.83119999999991
123.063600000002 9.09910000000039 -4.57590000000007
ID=TALtetFEMPERAMNH74114*

LM3=54
2.80899999999891 -3.96189999999981 -11.5287000000003
4.16629999999789 -2.03070000000016 -10.1702000000003
55.6571999999969 -4.18200000000075 -5.81829999999995
68.6283 -3.01060000000019 -7.94940000000013

51.6090999999986 7.78739999999978 -4.8431000000001
77.9435999999988 2.7602999999997 -5.42540000000015
79.8921999999957 4.47399999999944 -5.34349999999996
86.7024999999982 -0.234400000000298 14.8267999999999
102.821599999999 15.5525999999997 -11.9817000000002
92.6381999999985 6.86219999999988 0.0561999999999172
99.1939000000002 3.30760000000001 -5.57060000000014
107.203199999998 -5.74340000000025 -4.92290000000011
109.347699999998 3.85519999999965 -9.46320000000012
124.153599999999 -0.01400000000027 -6.02200000000014
130.898699999999 9.42059999999971 -9.90720000000014
134.678099999998 11.9812999999997 -8.15340000000012
134.468699999999 19.9068999999997 -10.8067000000001
140.836999999999 20.7452999999997 -2.44930000000017
137.884799999998 7.50619999999971 -4.97760000000014
139.313399999998 14.2380999999997 -11.9621000000002
0.854299999997887 2.92339999999962 -11.9123000000002
3.20689999999801 1.66739999999972 -10.4077000000002
51.8721999999983 19.6655999999997 -6.42590000000012
64.686299999998 22.7156999999997 -8.4826000000001
75.2218999999979 20.2959999999997 -6.22280000000009
77.4742999999977 19.2314999999997 -5.95710000000007
83.4496999999984 25.7151999999997 14.6336999999999
90.4205999999983 20.8713999999997 -0.577200000000098
95.7551999999985 26.2668999999997 -5.39850000000012
101.562599999999 37.5069999999997 -5.10720000000014
105.962299999999 28.7926999999997 -9.69460000000014
119.585099999999 36.6944999999996 -6.02010000000015
128.755699999998 28.5557999999997 -9.36820000000014
133.003199999999 26.3022999999997 -8.99640000000016
134.997599999999 32.4727999999997 -5.65800000000016
137.987699999998 26.5453999999997 -12.4387000000001
-0.355600000001919 0.590199999999696 -0.0165000000002706
1.10229999999816 4.4419999999997 -0.520500000000237
44.0942999999986 13.7920999999997 11.9255999999999
68.8536999999982 25.6017999999997 -0.474200000000104
66.1326999999981 25.0574999999997 -11.0487000000001
44.0706999999984 6.63109999999971 14.7730999999999
101.015299999999 14.7845999999997 22.5894999999999
126.235199999999 19.1743999999997 17.4140999999999
128.326399999999 37.0457999999997 4.14209999999984
137.807199999999 20.4369999999997 12.6750999999998
135.702799999999 29.8258999999997 -2.25400000000017
-0.371100000001849 -0.660700000000293 -0.00420000000028198
2.39389999999846 -3.93670000000022 -0.0821000000002992
45.6319999999983 -0.816200000000343 11.7941999999999
75.1118012441513 -3.6912436612703 -0.634323871819568
70.4530999999974 -5.38940000000037 -10.1843
132.534799999999 1.2156999999997 3.96239999999986
137.843499999998 10.5785999999997 -1.70520000000015
ID=TAMtetUNKPERAMNH75984*

LM3=54
4.8082999999951 -2.84680000000058 -10.0782999999993

6.05139999999547 -1.32580000000042 -9.27409999999932
54.864399999997 -1.79380000000023 -3.59799999999961
66.0735999999975 -1.13130000000021 -4.85959999999967
47.4774999999973 8.07139999999969 -2.47649999999963
71.7575999999982 3.96629999999981 -2.90939999999974
74.0190999999982 5.20769999999982 -3.11179999999974
75.0384999999982 -1.38090000000023 15.0106000000004
93.7599518357897 15.4157867807308 -8.86040308030233
84.8546999999989 7.55019999999993 1.03310000000022
88.2561999999994 4.34839999999997 -2.3417999999998
98.0339999999996 -6.03519999999993 -4.16009999999974
99.3104000000003 4.70560000000011 -8.69069999999983
113.1821 0.000600000000129136 -6.10149999999978
120.510200000001 9.17390000000021 -8.78389999999985
123.742800000001 11.9837000000001 -9.07709999999985
123.191100000001 19.0811000000001 -10.6781999999999
131.1512 20.1747 -3.20089999999979
128.155400000001 9.36310000000008 -6.77299999999978
128.868500000001 14.8077000000002 -13.1626999999998
3.58809999999548 4.21539999999947 -10.0600999999993
5.37859999999562 2.94919999999948 -9.04049999999935
51.1283999999973 19.6884999999996 -2.76289999999966
61.2050999999977 21.9967999999996 -3.67109999999972
68.7857999999981 19.8277999999997 -2.43399999999977
70.9886999999982 19.2306999999997 -2.46739999999978
72.4313999999983 24.8013999999997 15.8131000000003
82.846099999999 19.8616999999998 1.32900000000019
84.5899245515075 24.5776748235128 -1.64637002418909
91.5483999999996 36.7955999999999 -3.86609999999984
95.7469000000003 27.2142000000001 -8.26899999999993
107.6 35.0453 -6.5127999999999
117.757600000001 28.0061000000001 -8.72639999999989
121.644100000001 26.3647 -9.03949999999988
125.2479 30.3353999999999 -6.71529999999981
127.0919 24.8956 -13.1385999999999
-1.16580000000481 0.521599999999381 -0.553699999999288
0.936899999995595 3.67239999999952 -0.0214999999993304
40.610599999997 12.3532999999996 14.7026000000005
65.6174999999979 23.7914999999997 3.03220000000026
61.5651999999977 25.0276999999996 -6.76959999999971
41.0213999999971 6.66369999999962 16.4232000000005
93.1698999999989 14.4012999999999 22.7197000000003
116.394399999999 17.8153999999998 18.5144000000003
119.9798 35.5278999999999 1.80610000000019
130.5234 19.8848999999999 8.7109000000003
125.9521 27.5116999999999 -3.6639999999998
-1.10600000000477 -0.560800000000578 -0.588199999999287
1.99749999999523 -2.92300000000057 0.252200000000704
41.970099999997 0.916199999999573 14.0729000000005
69.3706999999977 -1.68260000000018 2.17690000000035
66.4644999999975 -3.59530000000027 -7.70179999999966
124.3826 1.91060000000005 1.36420000000028
127.622000000001 10.9728000000001 -3.99619999999979
ID=TAMtetFEMPER****76629*

LM3=54
1.6623999999933 -3.71339999999989 -10.5311
2.8363999999945 -1.61389999999959 -9.8174000000001
54.7509999999968 -2.10229999999995 -4.67389999999995
60.070599999997 -0.801100000000015 -5.46219999999994
45.6555999999966 7.83690000000001 -2.68269999999989
67.8256999999973 4.95949999999998 -3.96159999999993
71.6454999999971 6.13769999999994 -3.88399999999992
73.0232999999976 0.254800000000092 12.3079000000001
91.1964999999975 15.0632 -10.1036
82.235699999998 7.02710000000007 -1.05899999999998
84.5672999999979 3.65980000000005 -3.97090000000001
94.0290999999975 -4.90389999999989 -4.86460000000005
94.9444999999977 4.48800000000006 -9.75690000000003
110.682499999997 0.175500000000171 -6.66470000000009
117.836899999998 10.0459000000002 -8.45310000000014
120.894799999997 11.9327000000002 -8.46690000000011
120.926999999997 19.4792000000001 -10.9328000000001
125.994199999997 20.2119000000002 -0.993800000000148
123.141999999997 8.74870000000019 -5.49120000000012
124.105799999997 15.1238000000002 -11.9653000000001
0.229099999994046 3.85720000000025 -10.7770000000001
2.55219999999438 2.04790000000026 -9.76450000000013
50.7836999999966 20.3057 -4.54709999999994
56.035499999997 20.6535 -4.82549999999994
66.3436999999973 17.9464 -3.74249999999994
70.7136999999973 18.2482 -3.63869999999993
70.6501999999976 23.7944000000001 12.4839000000001
80.1698999999976 20.1543 -0.396999999999963
81.1608999999976 23.9945 -4.08869999999996
88.233999999997 35.1319 -4.45909999999997
92.1361999999974 26.5621 -9.8065
105.058599999997 34.827 -7.31670000000004
115.212599999997 27.6990000000001 -8.79580000000008
118.879599999997 26.1793000000001 -8.8993000000001
119.982799999997 30.5515000000001 -4.87630000000011
122.815499999997 24.7139000000001 -11.6874000000001
-1.19860000000588 0.308200000000283 -0.416700000000113
-0.0666000000058059 3.86830000000025 -1.26470000000011
38.0462999999964 11.5784000000001 12.1698000000001
60.5146999999972 24.1671000000001 0.939600000000056
58.0827999999968 24.0378 -8.27109999999996
39.5426999999967 7.13780000000017 13.164
87.7485999999973 14.4412000000001 20.3928
108.202699999997 18.1307000000001 17.3114999999999
112.169999999997 35.6241000000001 4.22109999999992
122.713199999997 20.5194000000002 11.3282999999998
119.465099999997 29.1833000000001 -2.75300000000012
-0.970900000005878 -0.890499999999697 -0.409500000000119
1.18059999999442 -3.86049999999969 -0.881500000000116
39.6734999999966 2.41470000000016 12.2671000000001
65.0857999999974 -2.29099999999997 0.152400000000073
62.946399999997 -3.9218 -8.49519999999995

117.913899999997 2.06070000000017 4.20069999999988
122.657399999997 10.6063000000002 -2.62970000000015
ID=TAMtetFEMVENAMNH76902*

LM3=54
2.95970000000001 -4.0215000000007 -11.3863000000003
3.40701389507042 -1.70797521515678 -10.9913875551231
51.7635000000017 -3.04820000000052 -5.11060000000025
59.583700000003 -2.41240000000004 -5.78960000000016
48.1025000000019 7.46789999999969 -4.3479000000003
68.9860000000017 2.63789999999988 -4.99300000000019
72.0979 4.4703999999996 -5.35439999999998
78.2581000000007 -1.65090000000034 12.7667999999999
92.0804999999997 13.9509999999997 -11.7303999999999
83.2945000000013 6.08130000000004 -0.146300000000097
88.7464000000005 1.73089999999976 -5.02699999999998
96.3427000000003 -8.44210000000039 -4.18169999999996
99.2449999999998 2.59899999999962 -9.5256999999999
112.816399999999 -1.07720000000057 -6.5655999999998
119.372899999999 9.13089999999941 -9.3991999999998
123.422799999998 11.4028999999993 -9.03029999999975
124.117599999999 18.4304999999994 -11.3283999999998
129.880199999998 19.4690999999993 -1.45399999999977
127.989999999998 7.64679999999932 -6.14599999999978
130.011799999998 13.3857999999993 -11.2306999999997
1.0514837295154 3.71345604374439 -11.22036934718
3.07833630247118 1.99975742710704 -10.8596505612133
49.3625000000016 19.9398999999998 -5.84540000000026
56.4855000000013 20.3169999999998 -6.4542000000002
66.4640000000008 18.3934999999998 -5.66450000000011
70.0101000000006 17.4349999999998 -5.43880000000008
74.1789000000007 24.3024999999998 12.7513999999999
80.4917000000003 19.2132999999998 -0.194600000000024
84.6250000000001 24.3361999999998 -3.96339999999999
89.8841999999998 36.5571999999997 -4.27099999999992
95.1315999999996 26.7554999999997 -10.1262999999999
107.044799999999 34.5612999999996 -6.58409999999983
117.120799999999 26.9543999999995 -9.07299999999977
122.244199999999 25.5648999999994 -9.00219999999977
124.285099999999 29.9658999999994 -5.91959999999977
128.837499999998 25.4002999999994 -10.9428999999998
-0.246299999999301 0.770299999999239 -0.0973000000001417
1.44270000000097 3.84149999999925 0.26849999999984
38.7833000000016 12.2852999999996 12.3120999999997
64.913500000001 23.7929999999998 -1.72860000000015
58.2220000000011 23.7544999999998 -8.67070000000017
42.2627000000016 6.75489999999957 15.4055999999997
93.4467000000001 15.2194999999997 20.4334
113.141599999999 17.2656999999995 17.2838000000001
118.691699999999 34.3834999999995 1.9546000000002
126.469699999999 19.5578999999994 11.4761000000002
124.524199999999 28.3241999999994 -3.14919999999977
-0.308899999999327 -0.688400000000782 -0.0965000000001108
2.36550000000066 -3.19960000000077 -0.112300000000108

40.1459000000016 0.568399999999519 12.1482999999997
68.5828000000013 -3.88430000000024 -1.19230000000012
62.473600000001 -5.32560000000043 -8.43390000000017
123.079699999999 2.12369999999937 2.15160000000019
126.723299999999 10.6021999999993 -2.85559999999977
ID=TAMtetMALBRAAMNH95100*

LM3=54
3.3898 -3.342 -11.0658
4.9305 -1.3211 -10.5981
52.1133 -5.2404 -4.1275
60.3153 -3.6296 -5.2883
45.9774 6.8579 -4.5288
69.2798 2.0161 -4.8879
72.3584 3.7382 -5.8072
77.4401 -1.7238 15.7134
94.1587 15.1426 -10.9532
84.4734 7.4828 0.2743
87.5388 2.9498 -3.1055
97.3078 -7.0084 -4.1536
97.5569 3.2453 -9.048
112.2993 -0.228 -8.3052
119.9781 9.1477 -9.3915
123.9282 11.7919 -8.8486
123.7697 19.9934 -10.9831
130.6043 20.6405 -2.6565
127.8298 8.8627 -6.066
127.7393 14.5029 -12.8389
1.7709 5.1311 -11.205
4.4899 3.4672 -10.5095
47.9829 20.2905 -4.1348
56.3407 22.3608 -5.1022
66.9991 19.297 -5.4263
69.7831 18.4527 -5.8332
71.1689 23.7959 15.4251
82.1111 18.2762 0.2468
84.067 23.7175 -2.5964
90.3263 36.8679 -3.676
93.1822 27.6088 -9.0619
106.3574 35.8578 -7.5198
116.7161 29.2278 -8.8906
121.5174 27.0204 -8.7021
124.2781 32.3873 -5.2072
126.7626 26.4743 -12.4407
-0.8524 0.517 -0.1398
1.8411 3.9681 -0.2429
41.7866 11.8498 13.75
62.5485 25.0108 1.0642
57.0006 25.7252 -6.8091
38.771 5.5025 13.7213
93.3315 14.7662 24.8279
113.1734 17.0171 20.2162
120.0017 36.6522 3.0774
129.0139 20.0756 12.6033

125.4082 29.1298 -2.0639
-0.7788 -0.7586 0.0082
2.8575 -3.001 0.5441
42.9946 0.6034 13.7859
67.5286 -5.0215 0.132
62.3937 -6.4791 -6.8064
125.494 2.4835 2.0653
127.8357 10.7898 -2.1896
ID=TAMtetMALBRAAMNH96264*

LM3=54
2.4781 -3.1973 -11.3041
4.5581 -0.1604 -9.9783
58.6688 -3.6364 -7.4778
67.5159 -3.4641 -8.3361
49.468 7.4013 -6.3157
76.3863 3.014 -6.8658
79.6332 4.1443 -7.1045
89.7557 -1.6738 13.6581
97.9667 14.7602 -11.4607
90.1976 8.4512 -2.2871
92.9058 4.9057 -6.7901
100.4525 -5.8154 -5.8133
103.4904 5.4358 -11.1362
117.6987 -0.4449 -7.8354
125.112 9.3195 -9.7696
129.2189 11.426 -9.2344
127.8615 19.7462 -12.3002
134.8151 20.4521 -0.0854
132.6795 7.3542 -6.8072
134.0716 13.7787 -12.9313
1.3561 5.4115 -11.2768
4.0104 3.5371 -10.145
54.6169 20.9693 -6.7911
64.0484 23.6109 -8.2746
73.371 19.3955 -6.9284
77.0669 19.2351 -7.169
85.2461 26.0017 13.9996
88.5842 18.6278 -2.0397
90.4522 22.5968 -6.5517
94.3099 34.6097 -4.4815
100.3707 25.0429 -10.4916
112.8393 35.6096 -5.6673
122.2664 28.1758 -8.9447
126.5846 27.063 -9.1192
128.1182 32.1225 -5.7489
132.6256 27.0308 -12.5287
-0.9869 0.4298 -0.4115
1.1431 3.6857 -0.7093
44.0303 10.7123 10.7775
70.4414 23.2051 -0.645
65.2591 25.5055 -9.7529
41.3118 5.8518 10.5503
95.8226 13.8156 22.244

119.0579 17.4904 17.6541
121.2802 35.6887 2.2007
133.5406 20.18 10.2909
128.7671 30.2438 -3.1217
-0.8144 -0.7263 -0.3823
1.8563 -3.2049 -0.2529
44.5215 1.4125 10.4033
74.6131 -2.0279 -0.6819
69.8279 -4.7209 -10.7135
127.6549 1.503 1.3868
132.1245 7.9849 -3.3874
ID=TAMtetMALBRAAMNH96278*

LM3=54
2.07789999999742 -3.20840000000006 -10.5759000000001
3.63120000000173 -0.697099999999732 -9.70080000000008
49.1230000000006 -2.8402999999999 -3.6789000000001
59.294500000001 -2.36319999999959 -5.54740000000017
46.7013000000011 7.68720000000018 -4.7047000000002
65.5466000000001 2.68740000000023 -5.0066000000001
68.0651999999999 3.83210000000019 -4.91040000000015
73.7584 0.306700000000159 15.3771999999999
88.4625999999999 14.4545000000002 -10.0746000000001
81.7103 7.60940000000021 1.25789999999991
84.9284000000002 3.52140000000021 -3.62260000000018
93.4828999999993 -7.15639999999992 -3.21270000000009
95.2941999999999 3.46400000000014 -9.4908000000001
108.6352 -0.170199999999894 -6.45500000000009
116.4201 8.87990000000012 -7.36400000000009
119.7508 12.6255000000001 -7.4640000000001
120.6091 19.4634000000002 -10.1279000000001
126.7779 20.5538000000002 -2.11880000000009
124.239299999999 9.62260000000007 -2.92510000000008
126.0008 14.8290000000001 -11.2355000000001
0.545400000001088 5.37940000000011 -9.68680000000013
2.73390000000158 3.52390000000017 -9.54630000000014
44.7124000000014 18.3690000000003 -3.11170000000019
55.2428000000012 21.9026000000003 -5.34860000000019
63.2477000000008 18.6781000000003 -4.84000000000016
66.0625000000006 19.0074000000003 -4.83270000000014
69.3871000000008 23.4735000000003 15.7209999999999
79.3657000000004 19.9620000000003 0.482699999999873
81.0666000000003 24.1479000000002 -4.21070000000012
86.4988000000006 36.5947000000003 -3.40470000000015
90.8544000000001 26.7934000000001 -10.1138000000001
103.465400000001 34.7494000000002 -6.92650000000013
113.5211 28.6895000000002 -7.59670000000011
117.0867 26.1875000000001 -7.53150000000012
120.1396 30.6277000000002 -4.3138000000001
123.9555 25.5899000000001 -11.3982000000001
-0.644599999999473 0.420700000000047 -0.199200000000126
0.596400000001023 4.36780000000013 0.0161999999998685
38.5956000000016 13.6983000000003 13.3466999999999
62.899700000001 23.1157000000003 3.31089999999983

56.2120000000011 24.7681000000003 -6.67020000000018
36.6252000000014 6.42820000000021 14.4999999999999
90.4814000000003 14.8242000000002 22.5489999999999
110.4044 17.8711000000002 18.0573999999999
115.420700000001 36.6007000000002 3.08419999999986
125.165 21.2474000000001 12.0757999999999
120.793200000001 29.3681000000002 -1.29170000000012
-0.619599999999605 -0.970299999999954 -0.290300000000119
2.46340000000034 -3.95499999999996 -0.360500000000115
41.2760000000012 -0.198899999999814 13.0915999999999
66.4040000000005 -1.40339999999983 3.67679999999986
61.0649999999997 -5.32659999999984 -7.17000000000004
119.9716 2.2233000000001 3.93769999999993
123.9894 10.4307000000001 -1.03970000000009
ID=TAMtetFEMBRAAMNH96280*

LM3=54
2.30509999999642 -4.88900000000076 -11.5404999999997
4.50950000000744 -2.31039999999925 -10.8924000000002
55.7699000000003 -4.0893000000002 -5.75389999999984
65.3191999999981 -3.41990000000039 -6.16539999999984
50.9372000000017 8.19850000000003 -4.75149999999996
72.5778999999998 3.75619999999992 -4.27879999999999
74.6478 5.10619999999995 -4.99919999999995
74.0580000000012 -1.82439999999987 13.2200000000001
97.5478999999997 15.4007 -11.2120999999999
85.9946000000022 7.1184000000002 -0.307900000000002
90.2284000000016 4.03550000000009 -3.43179999999993
100.703000000001 -5.23509999999985 -5.61859999999993
101.497900000001 4.68690000000013 -8.51939999999996
116.130400000001 0.251600000000085 -6.3441999999999
123.3517 9.73240000000001 -9.15279999999986
127.573 12.8014 -8.73359999999987
127.6888 20.5318000000001 -11.0848999999999
133.3955 21.8154000000001 -2.04219999999984
132.1759 9.5142 -5.40079999999988
132.5498 15.3061 -11.9815999999999
0.315500000002201 4.29680000000003 -12.0585000000001
3.7079000000041 2.31690000000024 -10.9193000000002
51.5283000000012 21.2217 -5.42239999999997
59.7747000000009 22.5189 -6.75969999999996
70.0847000000006 18.5539 -4.17279999999996
72.2362000000005 17.6985 -4.84459999999996
73.9746000000009 24.6402000000001 14.4950000000001
83.3235000000008 20.1436000000001 -0.441599999999947
86.3217000000004 25.0254000000001 -3.65859999999992
93.3093 36.0728000000001 -5.49369999999989
97.9317999999998 27.2216 -8.94339999999988
110.1997 36.6197000000001 -6.19469999999986
120.2538 29.1475000000001 -8.54609999999989
124.6571 27.3337000000001 -8.24119999999987
127.0899 32.7261000000001 -6.04719999999988
130.5627 27.2706000000001 -11.9816999999999
-1.05579999999911 0.820199999999807 -0.467600000000071

0.465700000001695 4.17159999999992 -0.671200000000111
39.3688000000013 13.3274 11.5656
65.8719000000008 24.0265 0.578900000000047
59.6159000000009 23.8272 -8.87649999999995
44.1039000000012 7.38049999999997 15.8329000000001
94.6342000000011 15.3319000000001 22.6458000000001
116.446700000001 18.8100000000001 19.4852000000001
120.0313 37.9035 2.62120000000013
130.7241 21.5256000000001 12.4674000000001
127.4014 30.6067000000001 -2.97239999999986
-0.569599999999256 -0.957000000000231 -0.768700000000067
1.63850000000113 -3.35470000000022 -0.716100000000079
40.971900000001 -1.11370000000009 11.1489000000001
69.8683000000007 -2.87349999999999 -0.00469999999994952
64.9641000000018 -4.21539999999981 -8.91780000000005
126.7558 2.74170000000002 2.18040000000011
130.5587 12.1865 -2.89239999999986
ID=TAMtetFEMPERAMNH98575*

LM3=54
3.91969999999561 -3.81080000000027 -11.0376000000003
6.47589999999993 -0.557100000000439 -10.3456000000002
58.9837999999994 -0.474400000000064 -4.26800000000008
67.7578999999999 2.21330000000017 -5.45679999999999
52.3802999999999 11.9312 -3.06060000000021
75.7234999999989 7.72 -2.39750000000005
78.5637999999989 9.42709999999993 -2.47350000000015
84.4194999999987 4.9508999999999 19.2730999999999
102.130199999999 22.8790999999998 -6.86430000000021
91.7014999999989 13.0664999999999 2.94719999999992
94.7338999999992 10.6376 -0.846200000000177
106.595299999998 0.0637999999997084 0.014099999999837
107.773599999999 12.5771999999998 -4.42540000000017
119.743499999999 7.87409999999977 -0.838800000000142
126.758699999999 17.6522999999998 -0.87130000000014
132.439199999999 21.9214999999998 -0.819800000000154
133.101999999999 28.8311999999998 -3.07100000000017
137.837199999999 30.1117999999998 5.67429999999986
135.474299999998 16.4865999999997 2.64949999999983
137.480799999999 25.6307999999997 -4.82660000000017
2.18469999999894 4.93969999999973 -11.1418
5.73089999999959 2.73029999999984 -10.1292
53.3374000000003 25.9622000000002 -3.86130000000013
62.2075000000003 27.1833000000002 -5.39650000000015
71.1514999999999 24.8239000000001 -2.57970000000013
76.0628999999997 25.0091000000001 -2.14490000000014
79.9307999999996 31.6345000000001 17.9889999999999
87.8141999999993 26.4815 2.73359999999986
90.4957999999993 30.1668 -0.698700000000133
97.2671999999998 45.4100000000001 0.144599999999857
102.782599999999 34.3973999999998 -4.45880000000017
112.7714 43.9250999999999 0.0276999999998611
122.7359 36.8979999999998 -0.450400000000129
129.1992 36.2200999999998 -0.0163000000001463

129.9306 40.8166999999998 3.27299999999984
135.857199999999 35.2793999999998 -4.41700000000016
-0.132900000001851 0.861199999999671 0.0875000000000496
2.52649999999877 4.86789999999979 1.63160000000004
37.0030000000001 15.0359000000001 13.3018
66.1543 30.5023000000002 1.01859999999986
62.7747000000002 30.8846000000002 -7.79100000000012
40.4385999999998 8.89100000000004 16.5259999999999
100.563999999999 20.6214999999999 29.1133999999999
118.863299999999 25.3688999999999 28.1572999999999
122.9153 45.3956999999999 10.2122999999999
136.859199999999 29.5890999999998 19.3929999999998
132.549 38.8615999999999 6.60379999999986
0.0638999999980628 -0.675200000000333 0.0580000000000725
3.92159999999808 -3.3641000000003 1.12390000000006
43.1752999999996 0.617599999999992 12.817
72.6849999999993 0.173699999999968 0.844999999999868
69.7777999999986 -1.02330000000011 -7.75430000000006
130.467199999999 9.5848999999997 9.93539999999984
136.251399999999 18.8632999999997 6.32119999999984
ID=TAMtetUNKBRAUSNM114839

LM3=54
5.00540000000764 -3.52279999999868 -9.59060000000047
6.06780000000728 -0.709899999998291 -8.66550000000053
52.6393000000049 -0.794399999999172 -1.88300000000037
61.3473000000047 0.826800000000777 -2.37600000000039
44.4081000000052 9.89280000000077 -1.49930000000039
68.358200000004 7.01140000000067 0.569499999999646
71.3520000000037 8.56040000000066 0.295299999999648
74.1249000000036 4.05800000000071 18.7742999999996
89.5660000000017 19.8801000000004 -4.79300000000034
81.9042000000027 11.9053000000006 7.51829999999966
85.4770000000024 8.00760000000057 2.90579999999964
95.9400000000022 0.0283000000006377 -0.0539000000004384
97.9398000000017 9.76720000000055 -2.64090000000038
109.878000000001 6.92690000000049 -0.03130000000046
116.0952 15.3802000000004 -1.69840000000048
119.451199999999 19.1771000000003 -2.30910000000052
119.936299999999 26.3393000000002 -3.83950000000049
126.030099999999 28.2464000000002 4.32589999999944
123.4329 16.4021000000004 0.993399999999438
126.256799999999 23.2113000000003 -4.72040000000055
2.88780000000727 5.58170000000137 -9.85080000000056
5.05610000000714 3.67820000000132 -8.64120000000052
47.7356000000045 23.3941000000007 -2.22080000000035
56.1926000000041 24.6499000000006 -2.59250000000034
65.3045000000036 22.9986000000005 0.0386999999996696
67.7443000000031 22.4041000000005 0.522499999999675
69.5971000000033 28.3321000000006 18.4103999999996
79.0331000000025 23.0251000000005 6.92489999999968
80.4622000000023 28.8717000000005 2.97939999999968
86.6957000000014 40.3394000000003 0.30019999999963
92.9370000000013 32.1974000000004 -2.27470000000034

101.8833 39.7656000000002 -0.219800000000423
111.815899999999 34.7454000000001 -1.21950000000045
115.914199999999 32.7520000000001 -1.71890000000049
117.664299999999 37.0857000000001 1.33659999999947
123.081399999999 32.6163000000002 -4.35110000000054
-0.849299999992478 0.339700000001438 -0.398200000000576
0.540300000007384 4.43180000000142 0.0241999999994478
34.2805000000056 13.9281000000009 14.1512999999996
60.1822046436117 26.6711851433994 4.42000985401518
56.9484000000039 27.6512000000006 -4.36880000000034
37.7161000000056 8.27620000000092 16.6781999999996
91.0422000000022 20.3784000000006 28.0430999999995
107.827000000001 24.1370000000004 24.3459999999995
112.179699999999 42.5340000000001 7.07689999999952
124.2446 27.5505000000004 15.7251999999994
118.883899999999 35.4676000000001 3.12509999999949
-0.619799999992527 -0.262699999998582 -0.390500000000573
2.67960000000738 -3.6358999999986 -0.0124000000005814
36.1071000000058 2.12570000000099 13.9311999999996
65.6963423555413 1.01348063917274 4.27263785919374
63.7233000000043 -0.532399999999293 -3.76170000000038
118.9905 9.19470000000045 7.82139999999947
123.8785 19.1253000000003 2.86079999999946
ID=TAMtetFEMZOOUSNM256759

LM3=54
0.47729999999756 -3.8486999999997 -11.7412999999997
2.60505968835896 -1.22178239047613 -10.8786535673534
50.1603000000009 -4.0794999999996 -7.07840000000012
60.2599 -3.46069999999996 -8.94139999999999
50.1715000000013 8.3925000000006 -7.72350000000015
69.054900000001 1.57950000000024 -7.78360000000003
71.1777000000002 2.54800000000011 -8.36590000000005
74.3016999999998 -1.79379999999989 11.5484000000001
89.9357999999999 13.6168000000001 -13.6368999999999
82.4701999999996 6.03649999999997 -1.52609999999988
86.0947999999996 1.47049999999997 -5.31849999999986
96.8177999999987 -6.70660000000011 -5.41019999999983
97.3555999999992 2.25090000000002 -10.2051999999998
110.140299999999 -1.15500000000005 -7.0436999999998
116.637799999999 7.70909999999997 -8.55239999999976
120.454699999998 11.6837999999999 -7.79909999999973
120.969199999998 17.8152999999999 -11.3488999999997
127.179099999998 19.2725999999999 -1.1696999999997
122.986399999998 7.71799999999992 -5.33349999999974
126.443699999998 12.9165999999999 -11.4746999999997
-1.07809999999949 4.93700000000044 -11.8780000000001
2.24450000000112 3.2094000000005 -10.3568000000001
45.6079000000011 19.0650000000004 -7.37920000000008
55.8562000000009 21.3846000000004 -9.28150000000004
66.3975000000004 18.6146000000002 -7.51969999999999
68.6033000000003 18.6858000000001 -8.06009999999999
71.2398000000002 24.6048000000002 11.7479000000001
80.7476 18.9560000000001 -1.7553999999999

82.2155999999998 23.6042 -4.40279999999988
90.7673999999998 35.6960000000001 -5.03609999999985
93.2256999999997 26.8405000000001 -10.0305999999999
105.091699999999 34.3397 -7.46189999999976
113.489699999999 28.3049999999998 -7.78749999999973
118.015299999998 24.5068999999998 -7.85569999999971
119.759899999999 28.9580999999999 -6.2528999999997
123.992599999998 23.7407999999999 -11.3010999999997
-2.06099999999998 0.697500000000379 -0.641300000000073
0.289000000000728 4.38480000000046 -0.554300000000109
37.0325000000018 13.4852000000005 9.77999999999985
63.1962000000006 23.4492000000003 -2.93900000000001
59.9928000000007 25.3599000000003 -11.585
39.9501000000018 6.77890000000054 11.8841999999999
89.9816999999994 13.4584 22.4185000000002
112.820099999999 17.5737 19.1840000000002
115.772399999999 35.0021 1.54500000000029
127.377299999998 19.6024999999999 9.7248000000003
119.936699999999 28.0654999999999 -2.75049999999971
-1.97340000000009 -1.00729999999963 -0.445300000000058
1.57440000000011 -3.72439999999961 -0.0248000000000642
38.8586000000017 -1.24619999999946 9.84109999999986
66.6584035305112 -3.53609465069596 -2.06311589085279
65.1303999999998 -6.23149999999995 -11.3921
121.534799999999 2.17749999999995 1.85920000000025
123.150599999998 9.87799999999992 -2.18699999999973
ID=TAMtetMALGUYUSNM339664

LM3=54
4.8009 -3.3271 -11.0969
6.8372 -0.65 -9.5827
57.2322 0.0533 -3.552
62.3774 1.6776 -3.9906
52.7657 10.6569 -2.4867
71.1582 6.8478 -2.4207
72.8073 7.9694 -2.7312
78.7905 4.247 15.9837
97.1944 20.0134 -7.8259
87.8682 12.3429 3.3428
91.2567 7.214 0.1007
101.8578 -0.2699 0.1605
101.2379 8.341 -5.3517
116.3597 6.7355 -1.5429
123.5049 14.4881 -3.0861
127.7462 19.4469 -2.129
127.6774 26.2906 -5.3171
133.1229 27.1597 4.0697
130.8058 14.8577 0.715
134.7154 21.948 -5.1651
2.8283 5.313 -10.6878
5.8797 3.9816 -9.5958
51.6376 22.1725 -2.9167
57.527 23.1559 -3.6547
67.625 21.5935 -2.1238

69.5161 21.4163 -2.3961
72.921 27.7831 15.5915
85.3834 23.9486 3.0684
86.5541 29.1784 0.4748
93.4296 40.3633 0.3688
96.1185 32.374 -5.2706
109.4463 39.9052 -1.4407
118.9675 36.3471 -2.9404
124.8454 33.277 -1.4867
126.2457 37.8083 0.3352
131.8115 33.0513 -5.3725
-1.2681 0.3379 -0.1619
1.5332 4.1296 -0.5634
39.9167 15.5912 13.5578
64.8415 26.7009 2.5071
59.0088 26.1705 -5.1335
42.9619 8.901 15.6545
92.8817 19.6012 25.6447
115.9518 23.8993 23.6966
121.0462 42.2836 8.0107
130.2093 27.0167 16.0479
127.6881 35.9144 3.5016
-0.7236 -0.8715 -0.1984
3.3073 -3.0239 -1.0191
43.7066 1.7151 13.4058
70.7447 1.993 3.0331
64.8813 -0.6688 -5.5827
126.7758 8.8364 7.7643
131.9867 17.9446 4.1121
ID=TAMtetMALBGUUSNM361029

LM3=54
1.2431 -2.9327 -10.1808
3.0998 -0.8264 -9.8348
50.5906 -2.6001 -7.5249
58.0875 -1.4838 -7.9245
44.8212 8.1912 -5.8506
63.8923 3.2517 -5.7677
66.4155 4.5475 -5.5944
69.5072 -0.2401 11.3681
87.6837 14.7817 -10.9483
76.3814 6.8851 -1.1869
81.3198 3.3427 -5.0076
88.4562 -5.1196 -5.352
90.6461 5.2021 -9.4922
103.5139 -0.1011 -6.2591
110.8498 8.3837 -8.248
114.4852 11.6892 -8.0133
113.894 17.8816 -10.9085
120.4097 19.5428 -1.7722
117.9828 8.4299 -5.1799
120.0489 13.5466 -11.5776
0.0454 3.8713 -10.0075
2.3033 2.2624 -9.3886

46.6883 19.0351 -7.0505
53.8822 20.4209 -7.9671
60.8282 18.686 -5.7974
63.5328 18.1947 -5.7937
66.3529 23.0073 11.6515
74.2211 19.2835 -1.2362
77.4813 24.2409 -4.7805
82.2928 33.8589 -5.2139
87.1018 25.218 -10.9456
99.043 33.6813 -6.1524
108.1596 27.568 -7.9823
112.6428 25.2164 -8.3109
113.7763 30.1795 -4.8655
117.9734 25.2015 -12.1871
-1.0253 0.4134 -0.2761
0.2706 3.4217 -0.4834
33.493 12.2306 9.238
56.2 23.2247 -1.6431
55.0657 23.8255 -9.6107
37.2498 6.2947 12.3286
85.3792 14.8925 20.5061
103.573 17.1844 18.0272
107.4719 34.9082 2.9986
119.6063 19.3388 10.3358
115.155 28.3318 -2.9064
-0.7473 -0.6886 -0.1291
1.3854 -2.9312 -0.2722
35.6327 -0.4157 8.9567
60.8273 -2.954 -1.824
59.6914 -3.2678 -10.4188
112.3036 1.8449 3.1633
117.9446 9.4252 -3.2474
ID=TAMtetMALVENUSNM406688

LM3=54
3.7435000000034 -3.46419999999979 -9.62429999999994
5.08859999999582 -0.740900000000951 -9.33600000000018
52.0132999999979 -0.421400000000689 -4.21239999999996
57.9488000000016 1.39660000000004 -4.23099999999999
44.7516999999988 9.66999999999942 -3.14429999999997
65.8433000000018 5.99589999999975 -2.16479999999993
69.2143000000002 7.03909999999967 -1.98329999999997
74.9394999999999 4.25569999999961 16.7980000000001
90.3247000000008 20.8168999999999 -6.4926999999999
80.3796000000001 11.8530999999998 3.53680000000012
83.7919000000007 7.51919999999983 0.702600000000101
93.5894000000008 0.234099999999861 0.581100000000123
95.0324000000006 9.72109999999993 -4.7655999999999
108.764200000001 7.02929999999989 -0.706299999999853
116.276600000001 15.1451999999999 -2.07929999999986
119.084600000001 19.6864999999999 -1.18549999999985
119.707100000001 26.5510999999999 -4.04449999999986
125.140700000001 27.8303999999999 6.54360000000017
123.054800000001 14.8461999999999 2.14200000000015

126.809400000001 21.6719999999999 -3.17549999999985
1.65359999999785 4.45589999999929 -9.82650000000005
3.46979999999703 2.49059999999917 -9.22050000000007
46.2578999999992 21.6421999999995 -4.46569999999997
52.1879999999996 23.4814999999996 -4.71569999999995
61.8186 23.0182999999997 -2.18709999999995
65.8408000000001 23.0734999999997 -1.75209999999993
68.6705999999995 28.4566999999996 16.0500000000001
77.2138999999999 23.9050999999997 3.3580000000001
79.4018000000002 29.3873999999998 0.589700000000104
85.3185000000002 41.4507999999998 -0.460999999999904
90.1794000000005 33.1190999999999 -4.28919999999991
102.2314 41.4292999999998 -0.8824999999999
111.149700000001 36.9620999999999 -2.66709999999988
116.155100000001 33.4867999999999 -1.62189999999986
117.433500000001 39.578 1.00290000000016
123.545600000001 34.0170999999999 -4.38959999999984
-0.589900000000331 0.267399999999513 -0.351899999999989
0.600499999998716 4.18039999999939 -0.192500000000006
35.3624999999987 15.6082999999994 13.3876000000001
57.1046999999996 28.7280999999996 2.79430000000006
53.4847999999996 26.0818999999996 -5.78809999999995
34.2198999999989 7.8490999999994 15.1275
85.3023000000001 19.3195999999997 24.5098000000001
107.867200000001 23.6842999999998 22.9169000000002
111.523400000001 43.4419999999999 7.29220000000015
123.653500000001 27.3086999999999 17.8571000000002
117.727500000001 37.8397999999999 2.56340000000016
-0.381000000000117 -0.748600000000453 -0.187699999999991
2.35080000000015 -3.69340000000041 -0.492099999999982
38.1528999999989 1.18749999999938 13.3981000000001
63.7232000000006 -1.68800000000026 3.30220000000007
60.9938999999996 -2.16010000000046 -5.70379999999977
118.450500000001 7.8197999999999 7.41270000000016
122.798500000001 16.0390999999999 3.20190000000017
ID=TAMtetFEMBRAUSNM461730

LM3=54
4.69840000000087 -3.22859999999899 -9.97260000000011
6.57240000000095 -0.437799999999282 -8.81230000000022
49.3453000000006 -0.514999999999739 -4.10760000000007
59.3989000000004 1.40390000000015 -5.54870000000008
45.1934000000006 10.2881000000003 -3.64040000000004
67.2737000000002 7.25400000000004 -2.86850000000007
69.3837999999999 9.74259999999993 -2.65950000000008
73.9450000000006 4.03080000000015 14.6437999999999
92.0989999999998 21.0794999999999 -7.86300000000011
81.2417000000005 12.4966000000001 2.58029999999994
84.1531000000003 8.9837 0.3935999999999
94.8577 0.114300000000028 -0.0898000000001606
96.3480999999999 10.2401 -4.94980000000013
108.2759 6.95330000000003 -0.57500000000019
115.2568 15.6736 -1.12480000000024
119.004299999999 19.3954999999999 -0.481500000000242

119.587499999999 26.5718999999999 -3.48100000000019
123.963299999999 27.8149999999999 7.81069999999971
121.680299999999 16.3785 2.60109999999975
124.616599999999 21.0289999999999 -2.46330000000027
2.83230000000081 4.99870000000091 -10.1359000000003
5.61410000000099 3.1176000000009 -9.04750000000023
43.4895000000005 21.2080000000002 -4.31230000000005
53.8346000000005 24.6796000000002 -5.71260000000007
63.8652000000003 22.8568000000001 -3.33510000000007
65.7523000000002 22.3391 -3.13400000000008
67.8770000000008 28.6192000000002 14.5457999999999
78.1618000000001 24.4135999999999 2.41649999999991
79.6997000000001 28.6331999999999 0.0152999999999155
85.8907999999999 41.5745999999999 0.10359999999991
91.5399999999999 32.8793999999999 -5.1035000000001
100.5903 40.2314999999999 -0.575000000000132
110.9325 35.5015999999998 -0.885000000000171
116.021099999999 33.9627999999998 -0.637600000000241
117.2099 37.5576999999999 2.26109999999979
121.833399999999 33.7734999999999 -2.41570000000022
-0.335099999998931 0.513900000001028 0.140699999999785
0.969700000000975 4.02740000000096 -0.317200000000214
37.5532000000011 15.3691000000005 13.1965999999999
62.5172000000006 26.8498000000001 1.04359999999993
55.1664000000004 27.4061000000001 -7.65140000000008
44.6860000000012 10.1774000000005 16.3399999999999
87.3321000000002 19.5720000000001 25.5912999999999
105.5477 23.5251 25.1301999999998
111.918 42.0437999999999 8.42579999999979
123.900999999999 27.4435 17.5184999999997
117.690799999999 36.4118999999998 4.70239999999976
-0.118799999998941 -0.777299999999005 0.119999999999747
2.64810000000118 -3.33549999999898 0.0317999999997869
40.9638000000012 2.45040000000058 13.1413999999999
65.9438000000006 1.04660000000016 2.43569999999996
61.3618000000005 -0.675399999999852 -7.88600000000008
117.496399999999 8.50939999999998 9.14759999999973
122.009199999999 16.8007999999999 5.17899999999972
ID=TAMtetFEMBRAUSNM546272

LM3=54
14.9834999999874 -2.43680000000139 -2.96909999999933
27.634199999989 -7.78340000000143 -4.3676999999994
102.101099999993 -8.13260000000119 -11.0333999999992
131.341099999993 -6.10560000000126 -19.6933999999991
100.976299999993 16.401799999999 -18.8046999999994
125.679799999994 9.49969999999895 -23.0209999999993
138.085199999994 6.35639999999893 -27.9450999999993
152.312999999994 -5.34240000000118 17.6112000000007
138.110199999995 22.0237999999992 -27.2778999999995
161.130799999995 18.060299999999 -9.10309999999946
171.689399999994 15.1447999999988 -16.9648999999994
155.971999999994 -17.3801000000013 -12.2950999999991
187.138199999993 10.6195999999987 -19.1727999999993

201.652399999991 0.405899999998491 -15.7860999999992
208.698399999991 18.3458999999985 -16.8996999999993
215.43089999999 19.5917999999984 -20.6763999999993
210.856499999991 33.7544999999986 -24.5204999999995
225.96709999999 36.0529999999984 -11.8223999999995
220.34169999999 10.9802999999983 -16.4072999999992
220.39159999999 21.4446999999984 -28.3951999999993
12.9472999999887 7.50009999999855 -2.91449999999941
23.9331999999892 17.0065999999987 -4.52109999999949
94.8145999999926 40.556999999999 -10.4327999999996
122.661399999994 47.1472999999992 -19.4267999999997
124.225699999994 31.0666999999991 -22.3927999999996
135.043499999994 36.2501999999991 -29.2799999999996
142.961599999993 52.8611999999991 18.4509000000003
157.923299999996 33.5628999999992 -9.22629999999964
167.491399999995 40.4914999999991 -16.9495999999997
142.632099999994 65.9105999999991 -11.9922999999998
180.316499999993 48.8279999999988 -18.5812999999997
192.513399999992 64.0153999999987 -15.0669999999997
204.039699999992 48.6691999999987 -16.2144999999996
211.227399999992 49.0598999999986 -20.6252999999996
212.623399999992 58.2244999999986 -16.3551999999997
216.555499999991 48.9514999999985 -28.1003999999996
17.5202999999884 4.04369999999847 19.8778000000006
14.421199999988 9.01349999999846 16.5816000000006
87.1232999999917 36.2934999999988 18.9766000000005
129.257499999995 47.5725999999993 -4.12769999999973
123.941099999994 57.3039999999991 -24.2611999999997
104.849499999992 17.1979999999988 27.0996000000006
166.482499999993 27.5425999999988 26.2716000000005
216.65949999999 35.0409999999984 17.8941000000005
214.258299999991 54.2964999999986 -4.34259999999963
221.77689999999 36.0245999999983 13.9719000000006
218.887899999991 51.3141999999985 -15.5943999999996
17.6630999999879 2.34439999999841 19.6890000000006
16.1645999999878 -3.67210000000158 16.8151000000006
93.2970999999912 -7.14740000000141 19.6025000000008
137.216899999994 -4.65410000000115 -3.08659999999923
136.626299999993 -15.4496000000013 -25.3554999999991
219.96639999999 15.4510999999984 -4.71109999999927
222.400099999989 21.2872999999983 -15.2189999999993
ID=ORYafeUNKUNKMfNB1533**

LM3=54
15.343500000004 -5.64619999999915 -5.45369999999903
32.7836000000031 -11.3204999999991 -8.24269999999918
118.233600000001 -11.5087000000003 -15.2789999999991
145.677600000002 -9.67060000000015 -24.4091999999991
108.654100000001 14.9532999999998 -26.2824999999995
139.082500000001 6.42439999999962 -26.9861999999993
154.111600000002 6.49539999999976 -34.3455999999994
162.483200000003 -1.16740000000004 18.6815000000007
148.376300000002 21.2646999999997 -35.0470999999997
172.494000000003 19.2752999999998 -11.0447999999997

181.222500000003 16.0758 -17.2872999999996
169.372000000004 -17.1691 -15.2472999999991
200.085500000003 10.4554000000001 -19.9222999999995
217.407500000004 -0.602499999999735 -18.8891999999993
225.580100000004 17.2681000000005 -16.8911999999996
232.528200000005 18.7050000000006 -23.9121999999996
225.498200000005 34.1233000000006 -27.3458999999998
244.072000000004 37.9040000000006 -15.4855999999997
236.318200000005 10.2620000000006 -19.0133999999995
239.117900000005 23.1154000000007 -32.0112999999996
14.2354000000022 6.57070000000084 -6.30739999999918
27.0852000000022 16.6282000000008 -8.8903999999993
107.713200000001 44.5486999999998 -17.0233999999999
135.279400000002 50.1100999999998 -27.2167000000001
134.505600000001 33.3601999999996 -28.9600999999998
146.281500000002 36.7590999999997 -35.7103999999999
155.280800000002 51.0052999999998 16.9608999999999
169.796900000002 31.4228999999997 -11.4256
177.105700000003 37.2658 -17.8408000000001
155.154800609381 68.1235511120005 -15.3060413730176
194.459300000004 49.7303000000003 -20.7936000000002
205.769500000004 64.6930000000005 -20.7553000000003
221.288100000005 50.1565000000006 -17.7234000000001
226.355900000005 51.7552000000007 -24.3575000000001
229.061200000005 59.6431000000006 -21.4410000000001
235.026700000005 48.4169000000006 -33.1915
19.6591000000026 3.19020000000092 22.6703000000008
14.7922000000033 10.231600000001 16.9004000000007
93.2207000000009 37.2140999999999 15.9198000000003
138.756800000002 48.6257999999997 -7.91410000000014
136.075700000002 59.4409999999998 -32.6616000000002
109.763400000002 16.8214999999999 26.0516000000004
173.339100000003 28.2272 25.5357000000003
233.300300000005 37.6986000000006 14.4118000000003
231.757000000005 59.2945000000006 -5.06730000000006
238.106400000005 38.4816000000006 11.2240000000003
232.535600000004 55.8170000000007 -17.9195
21.3542000000029 1.32430000000091 23.1052000000008
17.2955000000033 -6.96859999999899 16.8390000000008
101.501400000003 -8.51709999999986 15.1613000000007
148.400100000002 -2.90010000000028 -8.06259999999924
149.080700000002 -16.7173000000002 -31.5255999999991
238.548300000005 16.0572000000006 -5.03409999999949
238.866500000005 20.4707000000007 -17.4814999999996
ID=ORYafeUNKTOGMfNB18470*

LM3=54
15.0196999999988 -3.7147000000003 -5.15130000000013
24.9235999999997 -8.72910000000109 -5.53160000000048
101.936399999999 -10.4042000000012 -13.9797000000002
123.278000000003 -7.20339999999991 -24.0735000000007
93.7123000000008 15.9760999999996 -22.3513000000005
112.6223 9.19139999999972 -25.5413000000004
128.667700000001 7.70719999999998 -33.1064000000007

138.779899999999 -3.08160000000073 12.8606999999996
125.285600000001 21.7487999999999 -31.3643000000006
146.152599999999 18.0932999999996 -10.7607000000004
154.9196 16.1069999999997 -18.8956000000006
144.544800000001 -14.9615000000003 -14.0000000000006
168.524500000002 12.2291 -21.6029000000007
185.029100000002 1.5837999999998 -19.5696000000007
190.915600000001 18.6218999999998 -18.9954000000006
196.322600000002 19.6992 -22.7081000000007
191.243600000001 33.6912999999999 -28.2498000000007
205.525900000001 36.0332999999998 -13.2476000000007
200.045100000001 13.9928999999998 -18.7768000000007
200.634400000002 23.7026 -31.1077000000008
13.3082999999999 5.80879999999935 -4.8873000000002
19.439600000001 13.7191999999995 -5.18220000000033
90.7968000000012 42.4250999999998 -13.8640000000005
112.063900000001 48.1084999999998 -22.9509000000006
109.094900000001 29.8052999999999 -25.2219000000006
123.671000000001 35.9189999999999 -32.1089000000006
129.3243 50.8855999999996 13.4134999999995
143.0925 32.3391999999998 -11.5450000000005
151.4943 37.5023999999999 -19.1532000000006
130.126200000001 63.8782999999999 -15.4248000000006
162.374100000001 45.9367999999999 -21.3328000000006
174.2164 60.4923999999997 -19.1460000000006
186.9234 46.5058999999998 -17.6705000000006
191.547800000001 47.8179999999999 -23.4412000000007
193.209000000001 55.3075999999999 -19.3785000000007
196.594700000001 45.6099999999999 -30.7066000000007
19.9851000000002 3.46129999999903 17.9922999999997
15.3221000000003 7.0403999999991 15.2986999999997
81.4467000000003 35.4361999999994 12.6473999999995
117.375200000001 46.0989999999998 -7.81690000000055
114.188600000001 57.2271999999999 -31.2692000000006
95.9239999999997 16.8936999999993 21.7902999999996
148.301599999999 27.2603999999994 24.7259999999996
197.3732 35.9324999999996 13.3965999999994
196.0619 55.1682999999998 -7.87580000000064
203.1136 37.5843999999996 9.5971999999994
199.463800000001 51.3512999999999 -18.2570000000007
19.6352000000002 2.29149999999902 17.6358999999998
18.0541999999999 -2.94260000000102 15.0339999999998
87.1116999999991 -5.30260000000098 13.2840999999997
125.732400000001 -2.33990000000017 -7.44310000000059
128.634100000002 -15.0881000000001 -29.8702000000007
203.0756 16.0182999999997 -7.14600000000062
203.997600000001 20.0590999999999 -18.3606000000007
ID=ORYafeUNKETHMfNB36755*

LM3=54
18.1762602054048 -3.24599021901505 -4.99376427033861
28.8654000000083 -8.8086999999983 -6.80569999999978
107.531099999998 -11.1987999999996 -13.3367000000002
134.911599999997 -9.1020000000002 -23.8368

104.219499999999 16.0966000000004 -20.1342
128.569699999997 8.20679999999976 -27.0316
138.483600000001 4.19060000000052 -29.9032000000002
153.156600000001 -7.37559999999981 19.3594999999999
137.291299999999 21.7014000000001 -29.3025000000001
163.977600000001 18.2621000000005 -6.65860000000009
175.072200000001 17.0872000000003 -13.0217000000001
155.296899999999 -19.1565999999999 -14.3592000000001
190.748500000001 10.2326000000003 -15.2793000000001
205.413700000001 0.160600000000075 -13.2194000000001
212.618500000003 17.3845000000004 -10.0131000000001
219.039200000002 18.7463000000003 -13.2162
213.449700000002 33.8882000000002 -18.7826000000001
227.680000000003 34.8232000000003 2.62949999999992
223.752100000003 10.8693000000003 -9.29170000000008
225.265600000002 24.1318000000003 -21.7499000000001
14.9740000000028 7.74650000000104 -4.26199999999973
25.0108000000031 15.5985000000011 -3.67969999999975
98.5587999999997 42.4782000000003 -9.72549999999998
125.2583 49.5317000000004 -19.8207000000001
126.057899999999 32.1404000000001 -25.3122
135.079599999999 38.0517000000002 -28.7861000000001
144.1367 51.2388000000004 21.784
161.5118 31.6367000000003 -5.45920000000009
171.0292 37.6668000000003 -12.6335000000001
142.6961 65.2498000000005 -10.4263
184.659100000001 48.8870000000004 -14.5131000000001
195.880500000002 62.7286000000004 -11.0669000000001
207.756300000002 49.5473000000003 -8.69140000000009
213.723000000003 49.7826000000004 -12.6665000000001
215.376800000003 57.8689000000003 -9.75030000000006
219.45405692908 48.9332465425528 -20.7499939747416
19.7089000000024 1.44740000000095 20.3017000000002
17.8692000000018 6.26900000000096 18.5392000000002
89.5772000000008 33.7972000000006 20.4058
132.4626 44.6127000000003 -4.39900000000005
124.6494 58.2788000000003 -27.4768
112.5278 16.0828000000005 30.5206
166.257300000001 24.9039000000003 30.2926
209.996000000003 31.9976000000004 26.5147
216.485100000003 55.7540000000003 5.12459999999994
219.462200000003 33.9252000000004 23.0296999999999
219.913200000003 53.5992000000003 -4.56900000000006
20.7269000000025 0.0563000000009888 20.2715000000002
19.8113000000029 -4.57119999999893 17.0508000000002
94.6964000000008 -7.18709999999941 19.7588999999999
140.560299999998 -1.98640000000002 -5.33650000000007
136.279899999998 -16.9584 -29.8547000000002
223.481000000003 15.2639000000004 4.26599999999991
226.926800000003 17.9151000000003 -5.21340000000009
ID=ORYafeMALETHMfNB39336*

LM3=54
15.7133000000184 -2.8294999999986 -2.42670000000131

30.2395000000163 -7.88649999999906 -3.81130000000086
104.035600000007 -9.57320000000012 -11.6082000000001
128.738600000004 -6.77970000000043 -20.6546999999999
97.8937000000073 16.0682999999998 -18.7682000000004
122.495600000004 8.90329999999952 -23.8606000000001
136.307000000003 6.99539999999948 -29.9188
148.008500000004 -4.16230000000021 17.7589
134.984600000004 21.8518999999996 -29.4086000000003
155.784200000001 17.2602999999995 -8.23380000000009
168.739600000001 16.0520999999996 -15.4083
149.410000000003 -16.8385000000003 -13.9897999999999
183.1366 10.0847999999997 -18.3196999999999
198.396999999999 0.562699999999757 -17.5215999999997
205.931699999998 16.4082999999997 -16.4732999999998
211.349499999998 16.7109999999998 -19.9802999999998
206.951299999998 32.7551999999997 -25.5058999999999
221.642899999999 35.2252999999999 -9.6712
217.944814876641 10.2679044264123 -15.5659722645663
215.987099999999 22.0898999999999 -29.4844999999999
13.9364000000176 8.28470000000114 -2.01470000000108
25.883100000017 17.2190000000011 -4.26140000000107
96.9781000000084 43.2007000000001 -12.1404000000009
119.950900000006 47.4669999999999 -20.8028000000008
120.268700000005 30.5204999999997 -23.7924000000005
133.286300000004 36.0413999999996 -30.6152000000004
136.954800000006 50.7009 16.3210999999992
153.248400000002 33.3382999999996 -8.36460000000037
164.057800000001 37.9846999999996 -15.4173000000003
137.886400000005 64.0715 -14.1459000000009
177.345600000001 47.4931999999998 -18.9571000000003
188.500400000002 62.1988 -18.2157000000005
199.8656 47.8480999999999 -16.7152000000002
205.832499999999 48.7783999999998 -20.8382000000001
210.4936347395 57.1924786145717 -15.9434467069332
212.1927 46.1942999999999 -29.6230000000001
15.0069000000177 3.41160000000114 22.404099999999
16.1199000000182 9.29330000000125 18.9188999999989
87.1551000000106 39.8186000000004 18.0716999999991
125.889000000006 42.4805999999999 -5.96120000000079
118.105800000006 53.6938999999999 -29.6773000000009
102.781700000009 17.8139000000002 27.5904999999995
163.523200000003 28.3884999999997 27.5226999999998
209.767700000001 34.7603000000001 19.8994999999999
212.386 54.3164 -5.37470000000021
219.217000000001 36.0178000000001 14.8490999999999
213.748099999999 51.2236999999999 -13.4510000000001
15.5390000000175 1.81970000000116 22.022399999999
18.8720000000183 -3.48099999999876 18.993799999999
94.9391000000096 -8.93339999999981 20.2747999999996
133.192800000004 -2.00020000000038 -3.05730000000001
130.659300000004 -16.3319000000004 -26.7529999999999
218.788899999998 14.1429999999998 -3.75589999999983
218.958299999998 17.1017999999998 -12.2653999999998
ID=ORYafeUNKSUDMfNB4259**

LM3=54
12.3611999999938 -3.4371000000011 -3.34239999999966
24.5073999999949 -7.57230000000102 -4.74109999999957
96.3680999999955 -9.683200000001 -11.9999999999993
123.746799999995 -7.0763000000011 -20.3458999999991
93.7553999999959 15.8348999999991 -19.0199999999995
114.936499999996 7.58969999999903 -22.4089999999994
128.539999999996 6.769399999999 -29.4475999999994
138.414999999996 -3.54300000000106 16.2483000000007
125.027399999997 21.4792999999992 -28.0770999999996
146.521999999996 18.687499999999 -8.82449999999953
153.674099999996 16.1963999999989 -15.0252999999995
140.497099999995 -15.6980000000011 -11.9565999999991
170.579399999995 11.7840999999987 -17.5015999999993
184.954699999994 2.13499999999857 -14.5006999999991
191.615199999993 18.9274999999985 -14.1106999999992
196.809499999993 19.6484999999984 -18.4135999999992
191.349999999993 33.3099999999985 -22.0165999999994
209.279499999993 36.7040999999984 -9.24749999999937
201.849099999992 13.3477999999983 -14.7349999999991
203.287499999992 23.9700999999984 -26.5492999999992
11.1419999999949 5.99599999999892 -3.16929999999963
20.5059999999951 14.175699999999 -4.72339999999971
87.8939999999956 41.5322999999991 -12.4272999999998
113.292699999996 48.2901999999992 -19.6100999999999
110.191399999996 30.4306999999991 -23.3018999999997
121.797199999996 35.6685999999991 -28.3046999999998
128.532799999996 49.670699999999 16.0010000000002
142.838199999997 31.7202999999992 -9.12159999999974
148.767899999996 37.542799999999 -15.0575999999998
126.507499999996 62.155699999999 -11.4917999999999
164.208099999995 47.4072999999987 -16.9392999999997
173.974199999994 60.8112999999986 -14.8496999999997
185.973399999994 47.2408999999986 -14.4238999999996
191.964399999994 47.3742999999985 -18.2278999999995
193.539699999993 55.4071999999985 -14.5814999999996
199.058499999993 46.6753999999985 -25.9827999999995
16.9869999999947 2.50029999999884 16.6153000000003
13.0646999999944 7.16809999999883 13.9813000000003
77.7981999999953 34.138399999999 14.8579000000003
118.020499999997 43.9963999999992 -4.76059999999987
110.542999999996 53.6546999999991 -27.1447999999999
97.9158999999954 16.363799999999 24.1371000000005
150.932699999995 26.4622999999988 26.4318000000005
196.658099999993 34.7868999999984 17.7415000000006
198.294399999993 53.6716999999985 -2.80469999999956
204.208699999992 36.0822999999983 12.4329000000006
198.903099999993 52.0572999999985 -12.2379999999995
16.6411999999942 1.1122999999988 16.3584000000003
14.3885999999942 -5.25590000000117 14.4990000000004
87.0526999999946 -8.52090000000118 15.4743000000007
125.535799999996 -3.57390000000102 -6.46839999999928
123.802999999995 -14.2969000000012 -26.6105999999991

205.002299999992 15.9730999999984 -2.81989999999915
205.423699999992 17.9327999999983 -12.5285999999992
ID=ORYafeFEMSUDMfNB4488**

LM3=54
14.7983 -1.8576 -4.1634
24.8665 -6.9348 -6.385
103.0835 -11.1901 -13.5035
121.0162 -8.7779 -15.1729
96.4626 15.7547 -20.6036
116.9861 8.5885 -24.1557
130.088 7.3249 -29.5831
140.7542 -3.1807 16.7878
127.6343 21.0688 -28.5031
147.9009 19.2546 -7.8656
157.5975 14.9113 -15.2176
146.137 -15.4347 -12.6066
173.0141 11.194 -17.9493
189.2332 -0.0262 -13.6672
194.0704 15.8743 -13.6688
199.1263 17.9297 -18.4315
194.6147 32.0641 -23.1608
209.1539 34.7486 -13.3118
204.6021 12.4777 -14.5395
202.3542 21.329 -26.2419
13.0143 5.5588 -3.3722
20.5811 13.7768 -5.1194
93.3989 43.3454 -12.7353
113.3036 46.8137 -17.63
113.6841 29.8647 -24.5439
126.3748 34.4863 -30.4316
131.0008 48.6655 16.4803
145.7108 30.9392 -7.9765
153.6043 36.8799 -15.7405
133.8759 62.4029 -12.5855
167.7578 46.4327 -17.5433
180.4793 61.4635 -14.1506
189.7198 46.9961 -14.603
194.7004 46.2161 -19.7726
197.946 53.7697 -14.437
199.9347 44.3321 -26.7577
18.8746 4.846 17.9461
13.9813 7.7083 14.6079
80.4321 32.6716 17.4537
120.4635 43.6511 -5.2292
114.8236 53.7695 -26.6559
97.2308 16.6168 24.3815
150.2589 25.9888 25.4755
201.3661 32.6055 15.7905
200.9602 50.8988 -2.6814
205.5665 33.5392 12.9515
201.6083 48.0253 -15.856
17.4091 2.1786 18.0934
15.9652 -2.4119 14.8599

86.9096 -4.8309 16.3721
127.9812 -1.7293 -4.7839
126.8908 -13.3741 -27.2822
204.4975 15.5382 -4.3143
205.1667 20.71 -14.5941
ID=ORYafeFEMETHMfNB47494*

LM3=54
18.3627999999942 -3.26740000000044 -4.25349999999939
31.8750999999955 -8.65350000000066 -6.15579999999941
114.483899999995 -11.3314000000007 -14.2037999999996
141.335299999995 -10.4974000000009 -22.9444999999996
109.700199999996 16.7613999999993 -24.2776999999999
133.184999999996 6.80099999999921 -27.5201
146.240999999996 4.99809999999918 -32.1334
163.580099999997 -3.85710000000092 14.4328000000001
144.182799999997 21.4863999999993 -33.1166000000003
169.307699999997 17.7395999999991 -11.7085000000003
180.447199999996 14.751899999999 -20.2627000000003
169.354599999996 -17.685100000001 -12.9049999999998
197.509599999996 9.92219999999887 -23.3814000000003
214.428399999995 -3.06760000000128 -19.8397000000001
220.553299999995 16.5163999999986 -19.0697000000004
226.522799999995 16.9258999999986 -22.1880000000004
220.852599999995 31.2445999999986 -28.1605000000006
236.552399999995 33.9958999999985 -13.9806000000006
230.617099999994 10.5827999999985 -20.5287000000003
231.365099999994 21.8333999999985 -30.6833000000004
16.3014999999954 8.90179999999938 -3.74869999999943
27.8506999999956 18.6131999999994 -5.41009999999953
106.724499999995 45.3625999999992 -14.0903000000002
132.671499999997 52.3155999999993 -23.5662000000005
130.238199999996 32.8762999999992 -27.5190000000003
141.690299999996 37.8502999999993 -33.0158000000004
155.650099999997 52.5466999999992 14.4154999999994
167.069199999998 32.9109999999992 -12.4741000000006
177.326699999997 38.6283999999991 -20.4436000000007
157.136699999996 67.5444999999991 -14.0444000000007
192.256599999996 46.9884999999988 -23.5671000000007
205.532499999995 63.3246999999987 -21.0450000000009
216.285799999996 46.7839999999987 -19.7007000000008
222.114999999995 46.8729999999986 -23.9324000000008
225.899799999995 54.3474999999986 -20.1459000000009
228.324299999995 43.8968999999986 -31.3383000000007
26.1378999999952 4.9090999999993 17.8604000000007
20.727599999995 10.5058999999993 15.1150000000007
101.255599999996 40.6097999999992 15.1392000000001
135.981499999997 50.0619999999993 -7.84710000000052
132.163599999996 60.7236999999992 -28.9789000000006
120.865499999996 19.6671999999992 23.6606000000001
176.761399999996 27.7456999999989 23.4864999999997
225.023699999995 33.4796999999985 14.1810999999995
225.269199999995 53.5019999999986 -8.60790000000081
233.786999999994 34.5124999999985 10.4048999999995

230.432599999995 47.2556999999986 -18.1349000000008
26.6439999999948 3.95369999999927 17.3487000000007
22.3838999999947 -2.50520000000071 15.3831000000008
106.829499999995 -6.70270000000091 15.5098000000005
144.374499999996 -5.56890000000088 -7.61629999999984
144.961699999995 -18.0286000000009 -29.2308999999997
230.617499999994 13.0292999999985 -7.99350000000029
233.606499999994 19.4625999999985 -18.4549000000004
ID=ORYafeMALTANMfNB84691*

LM3=54
15.6846000000061 -1.94029999999871 -3.59400000000177
27.651300000007 -6.83929999999835 -4.83380000000172
101.290800000007 -10.1176999999991 -11.878000000001
124.847800000008 -7.35759999999887 -20.325300000001
95.9090000000059 16.618800000001 -20.0511000000013
119.589100000008 8.77160000000112 -23.7734000000012
132.483000000008 8.34810000000123 -29.6595000000011
144.120200000011 -3.78379999999844 17.0820999999987
130.086700000007 22.7185000000011 -29.2976000000014
151.528000000009 19.2426000000015 -9.42500000000135
160.258900000009 14.2527000000015 -18.3339000000013
149.28380000001 -17.4192999999985 -11.8739000000011
174.816100000011 10.9588000000019 -19.7409000000013
190.783200000013 0.978700000002411 -18.7588000000015
196.167100000012 17.9940000000024 -17.3557000000016
201.580000000013 18.9836000000026 -23.1360000000017
196.602700000012 33.6143000000025 -26.0828000000018
211.966100000014 35.4177000000028 -13.738000000002
208.200100000014 11.8686000000028 -19.2977000000018
204.280200000014 22.7325000000028 -31.4382000000018
13.3989000000064 7.71070000000161 -3.38170000000189
22.974200000006 16.2665000000015 -5.06220000000182
93.0634000000061 44.606000000001 -11.8294000000017
114.681200000007 49.3375000000012 -19.3186000000018
117.095200000007 32.4752000000011 -24.1312000000015
128.307100000007 37.7797000000011 -30.1446000000016
133.657300000008 52.2680000000014 17.036999999998
149.047500000009 33.9366000000014 -8.49440000000154
156.571500000009 39.8702000000015 -15.4007000000016
133.207900000008 66.1900000000014 -11.7449000000021
168.55040000001 49.6433000000018 -18.4578000000018
180.120100000011 63.3156000000021 -19.1382000000022
191.771000000011 48.0992000000022 -16.876600000002
197.020200000012 49.1296000000025 -22.964000000002
200.523400000013 57.4796000000026 -18.8553000000022
201.486200000013 46.6195000000026 -31.4478000000021
18.1397000000072 3.73390000000174 16.2000999999981
13.5702000000068 8.37160000000169 13.4072999999981
81.4988000000065 36.7729000000011 17.1309999999983
120.543400000007 48.8539000000011 -3.53290000000169
113.995300000007 56.8026000000013 -23.8244000000019
99.1468000000073 16.9691000000011 25.2521999999985
153.806400000011 27.0394000000018 27.4587999999982

206.878400000014 35.5887000000027 15.015399999998
202.825100000013 54.2549000000026 -8.37240000000219
211.726100000014 36.5297000000028 12.0982999999979
207.034600000013 50.8658000000026 -16.9697000000021
18.3113000000072 2.29000000000172 16.1769999999982
16.1899000000075 -4.12859999999824 12.6892999999981
88.8485000000077 -6.82659999999885 17.2153999999987
129.956200000009 -3.97479999999882 -5.26850000000108
124.918700000009 -13.3521999999987 -24.814200000001
208.621000000014 16.2033000000027 -8.93360000000176
211.472800000014 21.3100000000027 -17.7203000000018
ID=ORYafeMALTANMfNB84692*

LM3=54
21.0156235067857 -2.20357777054082 -3.35978529294248
32.4829485903099 -6.92194651366857 -4.46792366282786
124.981199999999 -7.98260000000059 -14.0414999999997
150.390999999996 -3.34100000000062 -20.7751999999999
115.363799999998 19.5067999999996 -22.857
149.012199999998 10.2446999999995 -25.7201
160.530299999997 7.56809999999943 -31.0091999999999
178.337300000002 0.3165 16.1609999999999
158.8249 26.0878999999999 -30.3070000000001
185.563799999999 22.3785999999997 -8.6492999999999
193.058900000001 17.8248 -16.5311000000001
176.704799999998 -16.7484000000004 -9.65539999999986
209.446 13.8722999999999 -19.6564
225.8976 4.43509999999978 -17.5279999999999
235.783 20.7247999999999 -16.2246999999999
243.2757 23.7008999999998 -20.6198
237.1051 40.7959999999998 -24.7743999999999
251.534239420978 43.5771735883645 -10.1894535272989
248.151899999999 15.5163999999999 -16.2418999999999
250.543699999999 29.7894999999997 -29.9911999999999
19.4683999999957 8.73549999999962 -3.46030000000011
30.110799999996 20.0284999999996 -6.42240000000009
112.716999999999 46.7723999999999 -16.6858
142.4086 51.778 -24.5929000000001
145.141499999999 39.6812999999999 -26.3148000000001
155.821 43.105 -30.4690000000001
165.311500000001 58.4426999999999 14.5765999999999
182.1629 39.3792999999998 -9.23190000000001
187.8334 46.6406999999998 -16.1873
160.3997 73.6375 -11.9041000000001
202.5789 54.9902 -19.2296
213.6554 70.5306999999999 -17.3106
228.101 58.2179999999999 -15.3126
236.7586 58.1609999999999 -21.1249
238.6332 66.9125 -17.3287
245.1025 56.0586999999998 -30.5117
21.933699999996 7.63779999999962 24.1827999999999
19.797799999996 11.5975999999996 20.6760999999999
103.556899999999 44.2668999999998 17.0780999999999
146.0536 53.7368 -11.2684000000001

145.460599999999 60.7369 -28.3379000000001
117.4719 22.2214999999997 27.9791999999999
180.599200000001 31.6757 26.1939999999999
237.475163427515 40.6260038933359 18.6579638187841
239.4572 65.4059999999999 -4.1679
245.549980015471 42.1927949908247 14.576617198647
242.3387 61.4227999999999 -13.3803
25.0595999999959 5.10609999999964 24.6368999999999
20.3946999999959 0.075299999999618 19.8484999999999
107.955899999999 -4.7466000000004 19.5178000000001
154.167799999999 -5.28000000000034 -8.51069999999994
155.355199999997 -11.4183000000006 -25.6451999999998
247.9043 16.8751999999999 -2.94139999999997
251.0975 24.7443999999998 -13.2213999999999
ID=ORYafeUNKCAMMfNB84694*

LM3=54
17.9593999999957 -4.84970000000003 -6.54740000000011
34.6245999999868 -11.7535000000011 -8.3803
115.0064 -10.0795999999989 -18.2880999999998
148.447399999999 -5.68949999999982 -24.0658999999999
114.748199999999 17.8524000000005 -23.4885999999999
149.310099999998 5.4218000000001 -28.1651999999998
159.0501 5.8052000000004 -31.0221999999999
167.674899999998 -4.74339999999995 14.7527000000001
161.0769 26.1991000000004 -31.8685999999998
185.269399999999 20.4085000000003 -8.62269999999984
194.342 16.2655000000004 -16.4681999999998
175.803299999999 -19.8213999999997 -13.4056999999999
208.822899999998 13.0832000000003 -19.3538999999998
226.607199999998 1.34190000000033 -15.4227999999998
235.282899999998 18.0935000000005 -13.9626999999998
243.808499999997 21.6053000000005 -17.0773999999997
238.660519551217 38.817983316558 -20.4814489553026
251.352720690467 40.4255512056242 -4.70211484441596
248.867799999997 12.8152000000005 -12.7459999999998
250.891391398005 25.2629510477912 -24.473872123998
15.8967999999945 9.18390000000004 -6.45610000000019
29.7776999999928 20.6706999999998 -7.26100000000016
105.076299999999 43.8596000000006 -16.2997
138.606 48.8790000000006 -21.3665999999999
142.5644 39.6910000000005 -26.8145999999999
150.3935 43.5035000000005 -28.9589999999998
161.768999999999 55.3070000000002 16.6656000000001
182.2392 37.9430000000003 -7.95439999999986
189.145 45.4637000000005 -15.5504999999999
160.147 74.0540000000003 -10.4601999999998
203 52.8474000000004 -18.0899999999998
216.096799999999 69.0180000000003 -14.1286999999998
227.902899999999 55.3625000000003 -12.3921999999998
237.891699999998 57.4884000000004 -13.9730999999997
240.796699999998 65.0155000000005 -10.5244999999998
246.754472393469 55.2847433340008 -22.6646408419072
26.1053999999928 4.30989999999993 23.5236999999998

21.8517999999936 9.63039999999988 19.8496999999997
98.0644999999972 39.9896000000002 18.895
141.7773 53.8931000000005 -8.80119999999991
145.9666 61.9582000000006 -28.0837999999999
116.426899999998 17.6826000000001 26.7257
181.167099999999 27.6849000000002 26.0049000000002
234.028399999997 36.3110000000004 25.5116000000002
237.153699999998 62.7930000000004 0.428100000000236
241.669699999997 37.4726000000004 23.9144000000002
244.626299999998 59.6205000000004 -9.28219999999975
27.036499999993 1.86249999999976 22.8672999999998
24.3019999999928 -4.22980000000031 18.3707999999998
109.566999999997 -10.8143 17.3911000000001
152.435999999999 -9.11509999999973 -9.04659999999988
156.767199999999 -14.5063999999997 -29.9509999999999
245.915799999998 13.2658000000004 -0.791499999999791
251.593199999997 20.9626000000004 -9.34679999999976
ID=ORYafeUNKCAMMfNB84695*

LM3=54
15.7564999999923 -4.69410000000294 -5.83539999999935
34.4004999999936 -12.3608000000027 -10.2519999999998
117.102799999995 -15.424900000002 -17.9205999999993
143.655799999995 -10.779900000002 -26.6945999999992
110.527699999996 17.1485999999982 -26.1195999999996
138.617699999996 5.39479999999816 -32.9662999999995
147.718799999995 4.93509999999815 -37.0312999999994
169.352799999995 -4.10100000000201 15.4819000000006
145.035199999997 23.3029999999984 -38.6955999999997
175.451499999995 21.0266999999983 -13.7502999999996
185.555299999995 17.5927999999981 -20.7767999999995
175.394699999995 -20.6369000000022 -11.7209999999992
201.403999999993 11.4167999999979 -27.0981999999994
217.357299999992 0.0464999999976099 -22.6194999999992
226.409199999991 20.8955999999977 -20.4696999999994
233.75329999999 21.4457999999976 -25.7291999999994
227.424399999991 38.2855999999977 -30.3112999999995
245.37349999999 39.9552999999975 -17.7948999999995
239.01049999999 14.5994999999974 -21.8945999999992
239.52879999999 28.3405999999975 -34.1847999999993
14.3419999999936 8.44539999999721 -4.11419999999984
28.2335999999939 20.8380999999974 -7.7106999999999
107.533699999995 50.2159999999982 -14.3154999999999
131.842899999996 55.5772999999984 -20.9443
132.848099999995 37.1080999999983 -31.8104999999998
142.904499999996 42.5652999999984 -35.3365999999998
159.768499999995 55.7018999999983 18.0388
172.870699999997 35.0258999999985 -13.1267999999998
179.396399999995 42.7644999999983 -19.9715999999999
158.040099999995 74.3531999999983 -6.84820000000003
192.969999999993 53.461099999998 -25.4158999999998
204.611899999992 69.8183999999979 -19.3237999999999
219.791899999992 54.2456999999979 -19.4022999999997
227.304299999991 55.6816999999978 -24.2446999999997

230.465999999991 63.7866999999977 -18.8497999999998
234.959899999991 52.2018999999976 -33.5866999999996
23.4033999999933 3.09629999999717 20.4248000000001
19.407899999993 9.62969999999716 15.3435000000001
97.6688999999945 45.171099999998 19.6505000000001
139.338899999996 50.6403999999986 -8.981
136.421699999995 65.6802999999983 -33.1064
117.147799999995 17.627699999998 32.4414000000003
179.068299999994 28.127299999998 26.9428000000004
232.150699999991 37.1312999999975 20.3824000000005
229.669999999991 59.9417999999977 -5.89619999999974
237.21749999999 37.4956999999974 16.8420000000005
232.145599999991 60.0949999999977 -16.8010999999997
22.2307999999928 1.25829999999711 18.1172000000001
21.2156999999927 -6.3246000000029 12.7573000000002
108.035799999994 -15.9326000000024 16.8859000000005
147.816799999995 -5.23190000000191 -9.29849999999935
151.496099999995 -20.0836000000022 -35.5196999999992
237.39929999999 16.0482999999974 -6.29339999999929
238.429599999989 18.5198999999974 -16.1829999999993
ID=ORYafeUNKCAMMfNB84696*

LM3=54
17.2779000000039 -4.7225999999994 -4.82100000000078
29.9072000000081 -10.3212999999984 -6.76860000000048
111.974800000006 -12.6972999999987 -13.4262000000004
133.930300000001 -9.79859999999908 -20.8604000000001
105.450500000004 15.4305000000007 -21.7258000000002
125.766199999998 8.7351000000004 -24.8176999999999
139.925900000004 4.54460000000127 -31.2619000000001
154.5035 -5.27569999999954 14.5199000000002
136.836300000002 20.4620000000007 -30.1891
161.8037 17.0303000000004 -9.32489999999981
172.8068 13.1144000000005 -17.3080999999998
158.794300000003 -18.540599999999 -13.8207000000001
186.063600000001 8.58580000000052 -19.7582999999998
201.5492 -1.76959999999942 -17.7856999999998
207.170099999999 15.7207000000004 -17.0102999999997
213.8209 16.5266000000005 -22.2448999999997
208.5252 31.0759000000004 -26.5949999999997
225.6031 33.5993000000004 -13.5903999999997
218.8741 9.90050000000052 -18.7642999999997
217.2893 21.4780000000005 -30.8376999999997
15.6001000000036 5.97030000000065 -4.79080000000047
25.0462000000028 14.9662000000005 -6.26520000000039
102.724700000002 44.3426000000005 -13.2522000000001
125.467400000002 47.7053000000005 -21.8237
122.963700000002 27.9173000000006 -26.7187
131.736200000001 37.4831000000005 -30.0299999999999
144.838500000001 50.9327000000003 14.6511000000001
159.7573 31.4993000000004 -9.87119999999984
169.3153 38.0324000000004 -16.7763999999998
144.65686084983 63.3653163742737 -13.7285764355946
179.8204 45.4204000000003 -21.1223999999998

192.1122 60.3778000000003 -18.3726999999997
203.2054 46.7557000000002 -19.0863999999997
209.4814 46.9679000000003 -22.2066999999997
213.3373 54.7094000000002 -19.0385999999997
214.1085 43.2518000000003 -30.7134999999997
19.5363000000044 1.92910000000071 17.6302999999995
16.8050000000041 6.96690000000061 15.5912999999995
89.2045000000023 33.5624000000005 15.9586999999999
128.879300000001 45.3240000000004 -9.49079999999994
125.586600000002 55.4465000000005 -29.6569
107.558600000002 15.9024000000006 22.4352999999999
164.1482 24.2668000000004 23.8484000000002
216.7184 32.0915000000004 13.9572000000003
215.1205 51.9704000000002 -8.06649999999964
222.2402 33.5646000000004 11.0193000000003
217.5026 47.2540000000002 -13.8438999999996
19.9405000000045 0.339400000000745 17.3560999999995
17.6785000000051 -6.60979999999919 14.4433999999995
95.2517000000045 -8.55349999999917 15.3067999999997
137.920000000001 -7.33099999999926 -8.45750000000003
137.629200000003 -18.030999999999 -29.2688000000002
221.2021 12.1386000000005 -7.16039999999968
220.9737 18.0535000000005 -13.8339999999997
ID=ORYafeUNKTANMfNB84698*

LM3=54
15.4338000000021 -2.41739999999833 -5.74750000000147
30.9432000000032 -8.22449999999795 -7.60850000000126
115.348300000005 -11.1983999999989 -12.6288000000005
137.495700000007 -10.4344999999988 -14.7290000000004
108.212200000004 17.0830000000011 -20.3549000000008
132.984100000007 6.24810000000122 -24.8942000000006
145.278000000008 5.4799000000013 -29.4116000000007
152.88040000001 -6.69299999999835 18.7234999999994
143.741600000006 22.5741000000011 -30.4196000000008
164.856500000009 18.4842000000015 -7.61740000000073
173.783800000009 14.4102000000015 -14.1313000000006
160.042300000009 -18.1965999999984 -12.4822000000004
191.97240000001 9.74730000000188 -18.9941000000007
208.943900000012 -2.41229999999755 -15.1672000000007
215.936500000011 16.1787000000024 -14.2510000000008
221.619700000012 16.9442000000026 -18.9700000000009
216.805300000011 32.6391000000024 -23.428700000001
233.366100000012 34.5172000000028 -10.9904000000012
224.772700000012 10.1637000000028 -17.4093000000009
227.235700000012 22.0833000000028 -27.453400000001
13.3313000000022 10.359300000002 -4.85720000000139
28.2459000000021 20.9809000000018 -5.52900000000136
106.858500000004 46.2693000000012 -12.4158000000012
129.513700000005 52.4994000000012 -14.3712000000013
128.667400000006 33.8567000000012 -24.290200000001
141.421000000006 39.5554000000012 -29.660200000001
149.269400000007 54.7691000000015 20.1487999999986
162.396200000008 34.1379000000015 -7.37440000000098

170.869000000008 40.0351000000015 -13.666700000001
148.146400000007 66.2147000000015 -12.3261000000015
186.674900000009 49.9580000000018 -17.7395000000012
200.884600000009 64.3508000000022 -15.0678000000014
211.75460000001 48.3083000000022 -14.3897000000012
217.18470000001 48.8936000000024 -18.8920000000013
222.50830000001 57.2703000000026 -15.3570000000014
223.717800000011 45.8304000000026 -27.2564000000013
17.9216000000033 4.39240000000212 22.3994999999986
16.0334000000028 10.9225000000021 18.1603999999986
95.4176000000041 42.8150000000013 15.7842999999988
135.457100000006 46.4491000000012 -3.10680000000123
129.948300000005 60.1818000000013 -26.6406000000014
105.112100000005 17.2725000000013 27.4125999999991
166.88170000001 26.3201000000019 30.8212999999989
220.986300000012 33.0463000000027 20.6593999999988
222.388300000011 55.1957000000026 -0.593600000001409
228.079800000012 34.4773000000029 17.0498999999988
223.836000000011 52.0412000000026 -10.3156000000014
18.2141000000034 3.80390000000216 22.3233999999986
17.9299000000037 -2.7037999999978 17.7879999999987
103.069600000006 -10.1356999999986 15.1308999999993
141.456700000008 -3.59179999999874 -4.78340000000049
141.388100000008 -17.5505999999986 -26.2454000000004
227.274600000012 12.8529000000027 -1.65040000000088
227.993500000012 16.7948000000027 -10.3262000000009
ID=ORYafeMALTOGMfNB84700*

LM3=54
17.4724000000026 -4.34960000000039 -6.3137000000008
32.6157000000022 -9.68990000000031 -6.96860000000063
111.5154 -14.1621999999997 -13.3036999999999
134.8304 -10.8385999999996 -17.0963999999997
104.510400000001 13.8845000000003 -23.2915
128.692600000001 4.84870000000041 -25.9141999999998
141.937900000001 3.75380000000048 -30.3829999999997
154.842600000001 -4.51199999999966 18.9840000000001
138.055500000001 19.1065000000005 -31.4065999999998
160.478900000001 16.1787000000004 -9.25509999999988
168.992500000001 11.8770000000002 -17.6870999999997
158.390019011307 -19.7846927732927 -10.1226757034729
187.284599999999 7.20089999999992 -21.4911999999996
203.7025 -4.22250000000012 -15.3539999999996
210.5605 14.4131999999996 -17.1051999999997
215.595799999999 15.7057999999995 -20.9796999999997
210.8659 31.0473999999996 -26.5433999999997
228.878800000001 34.7891999999995 -14.8919999999998
219.929599999999 9.47169999999954 -16.6119999999996
220.5216 19.9585999999995 -28.7204999999997
16.0791000000028 5.83229999999976 -6.17220000000085
27.9974000000027 15.6283999999998 -7.57770000000079
102.742900000001 43.5874000000002 -14.1433
126.500600000002 48.1248000000004 -20.2552999999999
125.537100000002 30.6692000000004 -25.6349999999999

138.134500000001 35.4088000000003 -31.3764999999998
144.319200000003 51.1529000000004 18.2721
156.786500000001 32.2836000000003 -8.8931999999999
163.573200000002 38.5186000000003 -16.8991999999998
140.097800000002 64.0069000000003 -12.6812999999999
179.408100000002 48.7262000000001 -21.7823999999998
191.545900000001 64.9969999999998 -16.9199999999998
204.433800000001 47.8577999999998 -16.8829999999998
208.371700000001 47.9821999999997 -22.2318999999998
212.261000000002 56.4461999999997 -18.2848999999998
215.818400000001 45.8259999999996 -29.8016999999998
19.4712000000041 3.21539999999984 19.122599999999
15.6908000000033 8.17279999999975 14.7777999999991
86.196000000002 34.8452000000001 19.7317999999997
129.695300000002 45.6364000000005 -4.74809999999996
129.659600000002 58.4280000000003 -25.1971999999999
105.317600000002 15.4295000000003 28.1090999999999
164.065100000001 25.6295000000002 28.5243
214.753900000001 32.9578999999998 17.9135000000002
216.640400000002 53.2432999999998 -4.81069999999978
222.378800000002 33.9564999999997 12.3163000000002
218.502200000001 49.6771999999997 -16.2386999999998
19.8682000000038 -0.00950000000019813 18.461899999999
17.7679000000032 -6.71770000000029 12.6587999999991
94.0227000000015 -9.50729999999974 18.5043999999998
137.55 -6.31639999999959 -4.90579999999982
143.5577 -17.8178999999997 -25.8013999999997
222.2904 12.6277999999996 -4.85379999999973
222.4471 17.2866999999995 -16.4748999999997
ID=ORYafeUNKTANMfNB84701*

LM3=54
16.2953000000113 -2.58439999999931 -4.4027999999995
30.3497000000111 -9.00339999999941 -5.07379999999941
115.98260000001 -14.7255999999994 -13.0464999999995
142.068600000009 -10.0989999999994 -24.6720999999994
113.815300000009 18.1416000000005 -25.7275999999998
136.487500000009 7.54790000000051 -28.3842999999996
148.971700000009 3.37420000000061 -33.5414999999996
161.900200000007 -6.02689999999942 15.0036000000008
145.487800000009 22.1718000000006 -33.9888999999998
172.018100000007 19.6365000000005 -12.7970999999995
183.751700000007 14.1801000000005 -21.4863999999993
166.960500000009 -20.7234999999993 -14.2788999999992
198.986200000007 9.02820000000054 -22.5955999999991
213.520200000007 -1.09439999999951 -18.7628999999988
219.767900000007 16.0446000000005 -19.3245999999988
226.155400000007 17.1958000000005 -22.0350999999988
221.138700000006 32.9095000000002 -27.9647999999988
237.216000000007 35.1265000000003 -14.7343999999986
229.641700000007 12.0909000000005 -19.3915999999986
230.999800000006 21.2096000000003 -30.0445999999987
14.475700000011 8.45870000000057 -3.9156999999994
24.0704000000104 18.2030000000004 -6.48439999999947

103.344500000008 47.2481000000004 -14.6947999999999
132.413800000008 52.8231000000005 -25.9027999999999
131.132600000008 32.5143000000005 -30.5961999999999
142.712000000009 40.4997000000006 -34.8283999999999
152.842700000008 56.0332000000006 14.4432000000003
170.290000000006 34.6668000000005 -12.9715999999996
179.465100000006 41.8694000000004 -22.0279999999995
153.168700000007 69.9819000000005 -16.3613999999997
192.704500000006 49.8087000000003 -23.8105999999994
204.338300000006 65.2051000000002 -20.0856999999992
215.658200000006 49.6534000000002 -18.3829999999989
221.870100000006 50.4450000000002 -22.3962999999989
226.787600000006 57.1754000000002 -16.8169999999988
228.452500000006 47.2040000000003 -31.3008999999988
23.7837000000108 5.91350000000053 20.6830000000006
17.4830000000114 11.3992000000005 17.0916000000005
97.6274000000083 39.9241000000004 17.4394000000002
141.033500000007 50.3265000000005 -8.11929999999986
137.653400000008 62.9730000000005 -30.9884999999999
118.690600000008 19.6956000000005 26.8345000000004
175.590800000006 27.8878000000005 27.0905000000007
227.816200000007 35.3629000000005 17.8331000000013
226.249700000006 54.3652000000003 -6.85069999999881
235.682900000007 35.4996000000005 12.4267000000014
231.060300000006 49.9653000000003 -15.1895999999987
24.1346000000109 3.94720000000057 20.1513000000006
19.4259000000115 -2.79479999999946 17.4898000000006
103.113700000009 -7.84399999999948 17.6376000000005
148.755100000008 -4.67039999999938 -9.23849999999944
147.349500000009 -20.5565999999994 -30.2196999999993
231.351300000007 14.8207000000005 -6.46009999999862
235.715000000007 20.7610000000004 -15.4119999999986
ID=ORYafeUNKTANMfNB84702*

LM3=54
14.7720999999992 -2.24549999999946 -5.85439999999997
26.5142999999994 -7.94519999999957 -7.40559999999996
105.220200000001 -9.21789999999995 -15.2759999999998
125.837700000001 -6.68699999999995 -22.3119999999998
97.7788000000002 17.1722999999999 -22.5453
117.081300000002 9.28160000000004 -26.1475999999999
130.397500000002 8.6573000000001 -31.5807999999999
140.696400000002 -3.47939999999979 15.0441000000002
127.427900000002 22.5030000000001 -31.3197
149.842500000003 19.1674000000002 -10.1784999999999
158.722300000002 15.6136000000001 -19.5234999999998
149.167600000002 -16.3287999999998 -12.0233999999997
174.379600000002 11.2331000000002 -21.2788999999998
191.170700000002 0.035600000000187 -17.2469999999996
196.716300000002 17.4172000000002 -17.1464999999997
201.157100000002 20.5752000000002 -20.5652999999997
197.352600000002 33.2769000000002 -25.9185999999998
212.278900000002 36.0192000000002 -13.5554999999999
207.708700000002 12.4510000000003 -16.8989999999997

207.469700000001 23.3825000000001 -29.0906999999997
12.4498999999994 7.07560000000042 -6.11500000000004
20.7469 15.6757000000005 -8.12920000000008
96.158200000001 44.7620000000001 -15.5628000000002
116.065300000001 49.5064999999999 -21.6419000000001
112.4893 31.5403999999998 -25.7989
125.632600000001 36.7819 -31.1295000000001
131.076600000001 51.2145 15.0582999999999
147.669400000002 33.8809000000002 -10.2040000000001
154.528200000002 40.5948000000001 -19.7828000000001
134.482600000001 65.9364000000001 -12.5191000000002
168.199600000002 48.2643000000001 -21.3824
180.807100000002 63.7103000000001 -16.7191000000001
192.532800000002 47.3046000000002 -16.2661
198.455900000002 47.8585000000002 -21.9119999999999
200.446900000002 56.1376000000002 -17.9992
203.881700000002 45.9388000000002 -28.8894999999999
15.6770999999995 4.62150000000039 15.1344
12.3508999999996 9.42780000000041 12.502
81.7806999999999 38.3204 15.6728
119.258700000001 46.7623 -6.10090000000015
118.775900000002 57.8556000000001 -27.2182000000002
95.6583000000007 16.8149000000001 22.7559
155.603700000002 27.7985000000002 24.2529
203.461000000002 34.6797000000003 15.8029000000002
203.575300000002 53.9768000000002 -3.7069
210.193300000002 35.1424000000003 12.0121000000002
206.062400000002 47.4185000000002 -15.1198
15.6381999999995 3.1709000000004 15.3493
14.143899999999 -3.11309999999968 12.8556
89.5957000000007 -7.89899999999983 15.4276000000001
127.928400000001 -3.18899999999992 -6.33899999999982
129.566700000001 -14.4097999999999 -27.3790999999998
207.794900000002 15.9198000000003 -5.47609999999971
210.231200000002 21.6109000000003 -16.5504999999997
ID=ORYafeUNKNAMMfNB84704*

LM3=54
16.9539999999988 -1.67780000000172 -5.22659999999955
27.1574000000068 -5.40919999999971 -5.93520000000025
102.881999999999 -9.94530000000044 -11.6252000000004
130.171299999999 -7.76580000000039 -17.4726000000002
105.3689 17.5461999999993 -20.0985
126.735099999999 8.05679999999928 -24.6939
139.1244 6.92269999999963 -31.6194000000001
153.777400000002 -4.51640000000016 16.9601999999997
135.9748 22.0130999999995 -29.8437
160.49 19.5317999999996 -8.95570000000007
172.075400000002 17.5976000000001 -18.2428000000003
153.417200000002 -17.7015999999999 -10.9229000000002
187.0074 12.8977999999998 -22.5671000000002
204.6726 0.574100000000029 -15.7918000000002
209.2721 18.0543 -17.4693000000003
215.211700000001 18.6719000000002 -22.1642000000003

209.491800000001 33.5790000000002 -27.2081000000003
227.340000000001 36.0074000000002 -15.8092000000002
221.6951 13.5074000000002 -18.3551000000002
219.331000000001 22.8918000000002 -31.0638000000003
15.3941000000009 10.0120999999991 -5.75780000000015
25.0191000000025 17.7251999999995 -7.68060000000018
94.8335999999999 43.9813999999992 -14.6065
121.7243 50.1166999999993 -18.5500000000001
124.8623 33.1698999999993 -25.2527
132.6494 36.7680999999995 -30.6690000000001
141.957 54.9386999999995 15.1067999999999
158.4015 33.0484999999996 -8.95930000000013
168.8258 39.5087999999997 -18.5383000000002
139.1915 67.2555999999995 -13.5583000000001
181.1349 46.6870999999998 -22.4281000000001
195.351400000001 64.6034999999999 -16.4476000000003
204.3015 49.2829 -18.0479000000002
210.594 49.5824000000001 -22.0986000000003
215.133700000001 57.8271000000001 -18.0309000000003
216.006300000001 47.0468000000001 -31.3115000000003
19.293200000003 8.3158999999997 19.3202999999997
15.6463000000026 12.5736999999996 15.4757999999996
87.700600000001 38.4454999999995 13.7018999999999
128.4009 47.5446999999994 -6.64380000000008
122.9574 57.0845999999994 -27.5292000000001
104.929400000001 19.7814999999996 24.9461999999999
166.4542 29.4541999999997 27.1108999999998
216.264500000001 36.2409 15.1542999999997
215.927300000001 56.4673000000001 -5.61770000000023
223.348600000001 37.3200000000001 10.1783999999997
217.845700000001 50.8314000000001 -14.7829000000002
18.62084201458 6.74682366595118 19.4277541239324
16.9368000000031 1.37689999999969 15.4041999999997
96.2507000000015 -5.34290000000022 15.9788999999998
136.1976 -2.36890000000038 -4.85250000000014
133.62 -16.5168000000002 -26.2834000000002
223.718600000001 15.4215000000001 -4.38990000000032
223.004500000001 21.5143000000002 -15.4237000000003
ID=ORYafeUNKNAMMfNB84708*

LM3=54
-2.61659999999968 -6.53019999999795 -26.5551999999999
10.5792000000008 -11.5285999999983 -25.0337
99.2007000000022 -12.0753999999991 -21.6581000000001
127.559100000002 -8.86709999999905 -24.335
98.1315000000014 16.2931000000009 -31.9839000000001
125.789100000002 9.85190000000094 -34.3006000000001
136.885000000002 5.02440000000095 -35.4194
146.696200000002 -6.26919999999909 14.6523000000001
133.742700000001 23.0481000000007 -35.5222
159.288400000002 20.2973000000007 -10.9904999999998
170.927300000002 14.6680000000008 -17.7825999999998
153.039100000002 -17.3466999999991 -16.1279999999999
187.492600000002 11.4703000000008 -18.6154999999998

203.348000000002 0.00730000000083741 -13.5526999999997
210.212800000003 17.0009000000008 -13.6519999999998
216.792800000002 18.7870000000008 -15.1733999999998
212.315400000002 35.1624000000007 -20.8917999999998
226.224300000002 37.3091000000007 -5.85469999999974
221.868500000003 12.698200000001 -12.8017999999998
224.233300000002 23.8221000000008 -24.7286999999997
-4.81009999999932 4.39860000000147 -27.1671999999999
3.82460000000081 12.0687000000014 -26.5683
90.7515000000017 43.239500000001 -22.4258000000001
119.045000000001 49.7578000000008 -26.9073
121.082500000001 31.8497000000007 -33.523
131.130000000002 39.6320000000009 -35.5063000000001
135.801800000002 54.9846000000009 14.4484000000001
157.642400000002 33.9367000000008 -9.70169999999986
166.623600000001 42.0010000000007 -17.4029999999998
138.893000000001 66.7962000000008 -17.1348
181.292900000002 50.4335000000008 -18.5087999999999
193.781900000001 65.3689000000007 -13.8478999999998
204.660300000002 51.3736000000007 -11.9810999999999
211.182800000002 52.2638000000008 -15.0718999999998
214.397100000002 59.4869000000007 -11.3716999999998
218.160500000002 49.2229000000008 -23.7472999999998
-1.98509999999924 2.69270000000144 0.113200000000081
-8.60109999999935 5.32960000000147 -5.20909999999993
79.692500000001 38.851200000001 6.33640000000005
125.625500000001 49.9888000000008 -11.8923
123.340800000001 58.3870000000009 -31.8722000000001
91.4542000000017 16.4401000000009 19.0887000000001
155.790200000001 27.2284000000008 27.6480000000003
213.117300000002 34.9798000000009 25.5878000000002
214.835700000002 57.5983000000008 3.63520000000013
220.736700000003 36.819000000001 21.6979000000002
221.530200000002 51.8465000000008 -8.54779999999981
-1.03429999999925 -2.31119999999855 -0.0715999999999095
-5.622699999999 -7.07809999999849 -5.01809999999995
88.7021000000018 -9.90729999999896 7.34320000000004
135.117400000002 -4.46649999999914 -10.5871999999999
137.897300000002 -16.0034999999991 -30.7497
221.273800000003 13.0544000000009 5.95470000000024
224.998700000003 20.0325000000009 -9.86599999999977
ID=ORYafeUNKTANMfNB84712*

LM3=54
17.3554999999988 -1.83750000000062 -5.07629999999922
25.6649999999983 -7.791500000002 -5.97089999999921
112.083100000003 -11.7290999999999 -14.0283
131.070499999999 -6.89830000000062 -21.8866999999999
104.980600000002 18.5348 -23.6851
127.807400000003 9.39109999999994 -27.2517000000002
138.1321 8.41989999999966 -31.6371
149.028900000001 -2.30860000000033 17.7154999999999
136.570000000001 24.4613999999999 -30.2824000000001
157.6939 23.0981999999997 -10.3391000000001

168.243400000001 17.6327999999997 -18.6346000000002
154.846699999999 -17.3262000000006 -12.7203
182.533099999998 12.6532999999996 -21.0433000000001
202.070699999998 3.03879999999943 -16.5201
207.879399999998 19.3212999999996 -16.7566000000002
212.917999999998 21.8098999999997 -21.3814000000001
207.615299999999 36.6624999999998 -26.4325000000002
223.579699999998 38.9268999999998 -13.1963000000002
217.517299999998 14.7998999999997 -16.9573000000001
217.425999999998 25.9831999999997 -30.0089000000002
15.0769000000012 7.97999999999961 -5.00979999999939
21.2005000000011 16.2480999999995 -6.33259999999939
100.049600000001 48.3610999999997 -14.0481
121.056900000001 52.4694999999998 -21.5043000000001
125.016700000001 36.5222999999999 -26.6788000000001
135.416900000001 39.3998999999999 -31.9948000000001
140.5809 55.3971999999995 17.3792
155.6722 34.1171999999997 -10.5939000000001
164.0363 41.2623999999997 -18.0510000000002
139.796200000001 70.2600999999999 -12.9287000000002
176.083 51.6149999999998 -21.2795000000002
190.7823 67.7040999999998 -18.1253000000002
203.514199999999 53.0080999999999 -17.1916000000003
208.348499999999 51.8673999999998 -22.5365000000003
212.757299999999 60.8279 -16.7906000000003
214.072799999999 50.9593999999999 -30.9495000000002
20.3494000000023 5.15039999999955 17.4584000000006
15.2805000000027 10.1185999999997 14.6914000000005
85.9532000000016 40.3490999999996 15.7845000000002
126.870300000001 50.5020999999996 -8.00480000000006
121.329600000001 62.6967999999999 -26.1678000000001
100.236000000001 19.5977999999996 25.0596000000001
162.642500000001 30.6573999999996 27.1505999999999
213.9512 38.3719999999999 17.5668999999998
212.580799999999 56.5805999999999 -5.95180000000025
220.036299999999 39.2083999999998 14.2810999999998
216.766199999999 54.1036999999999 -15.2545000000003
19.8544000000024 3.56329999999955 17.4954000000006
17.900900000002 -2.23770000000053 15.1150000000006
94.2116000000018 -6.79790000000038 16.0570000000002
135.281199999999 -3.92310000000055 -7.93129999999996
136.343999999999 -16.2534000000004 -26.8762999999999
217.671199999999 18.3798999999998 -6.01690000000018
220.696099999999 22.5247999999998 -16.4277000000002
ID=ORYafeUNKNAMMfNB84713*

LM3=54
17.8873000000001 -1.58040000000015 -5.87689999999964
29.8404000000004 -7.52680000000124 -6.6856000000006
108.683400000001 -9.52679999999973 -14.4080000000002
132.466000000001 -6.2022000000003 -21.7739999999998
100.8401 18.6857999999996 -22.243
128.599000000001 9.02299999999991 -27.1529999999999
141.007700000001 6.53070000000004 -32.3092

152.108400000001 -1.96200000000019 15.3392000000002
136.904 24.5781999999996 -31.9746999999997
158.807800000001 19.5227999999999 -10.1841999999997
168.703900000001 16.3566999999998 -19.2510999999996
155.4187 -18.3267000000003 -13.6466999999997
183.936900000001 14.0303999999999 -25.7672999999997
201.764000000002 0.352599999999915 -22.3585999999997
209.887600000001 20.6929999999998 -22.3257999999996
213.771600000001 21.2614999999997 -29.0144999999996
206.735300000001 36.3750999999998 -33.1787999999996
223.222900000001 39.1691999999998 -18.8173999999995
220.0143 13.9010999999997 -25.9649999999995
216.708200000001 26.0111999999997 -38.4163999999996
16.3983999999987 9.51009999999894 -4.60299999999991
24.5079999999991 18.8597999999989 -5.55169999999993
99.4294999999997 47.1537999999995 -14.5936999999999
123.012699999999 52.6293999999995 -21.9375999999998
124.0119 35.6731999999996 -27.4128999999997
134.7726 43.1427999999996 -32.1382999999998
143.2197 55.6657999999996 14.8119000000003
154.848600000001 36.1571999999999 -11.9031999999998
163.7728 42.8033999999998 -20.4872999999997
139.8103 72.0799999999996 -13.4934999999997
177.408 50.9627999999998 -26.8041999999996
189.6902 68.4075999999997 -22.2982999999996
202.9626 53.6442999999997 -24.6145999999995
208.247900000001 52.7033999999998 -29.4130999999995
213.331700000001 62.7582999999997 -25.3696999999995
212.038900000001 49.9636999999997 -37.7011999999995
19.9918999999985 4.73589999999856 19.5886999999999
15.2776999999982 10.0053999999985 15.5231
90.9958999999998 42.1158999999995 14.205
126.6104 50.9021999999997 -7.29419999999978
123.213099999999 63.9480999999995 -27.9662999999998
107.4441 19.7950999999996 23.5339
161.9518 29.8280999999997 24.4070000000003
215.438 38.6098999999997 10.0067000000004
214.558300000001 59.9377999999998 -12.8079999999995
223.3331 39.2402999999997 4.75600000000048
217.298900000001 54.5081999999997 -23.7137999999995
20.6088999999985 3.6261999999986 19.0842999999999
16.9259999999986 -3.49480000000139 14.7264999999999
99.6973000000004 -7.46700000000026 14.1881999999998
136.856000000001 -2.62750000000021 -6.48629999999986
136.184500000001 -17.8420000000002 -27.4871999999998
221.2439 16.9657999999997 -14.2999999999995
221.9827 23.2687999999997 -24.1762999999995
ID=ORYafeUNKTOGMfNB*****1

LM3=54
0.547699999999143 -5.91460000000051 -27.1942000000007
10.7832999999985 -10.6719000000003 -27.2085000000006
91.5981000000009 -12.8797999999999 -26.3640000000003
118.815000000001 -9.76199999999992 -26.6228000000002

87.9734000000002 17.9482 -33.3632000000002
113.009700000001 8.2006000000002 -34.0679000000002
126.2893 6.23110000000009 -37.5760000000001
131.702700000002 -5.97249999999973 13.5981999999997
123.0948 24.4376000000001 -36.4112000000001
142.297300000001 19.9054000000002 -11.6078000000002
154.182300000001 18.3444000000002 -17.9956000000002
139.102300000001 -20.4824999999997 -15.3288000000002
170.236900000001 12.9539000000003 -19.7196000000002
185.364200000001 1.56300000000033 -14.8081000000003
191.733400000002 19.9935000000005 -12.7089000000003
199.218500000002 21.3856000000005 -15.2186000000003
194.679700000002 37.2320000000004 -20.5074000000003
205.445300000002 37.8768000000004 -1.62760000000035
202.285400000002 14.4403000000004 -11.8114000000002
205.079200000001 27.3999000000004 -24.1854000000003
-2.14140000000049 6.53429999999983 -26.3668000000006
5.9698999999994 14.9422999999997 -25.2579000000005
81.3601999999997 47.0394999999998 -23.3889000000003
107.194900000001 53.8665 -24.6766000000003
108.6457 36.0531 -32.1475000000002
119.748 41.5541 -35.3435000000001
119.3225 51.3734999999999 16.3412999999997
139.865100000001 34.4686000000002 -10.4081000000002
149.386700000001 41.4184000000002 -16.3091000000002
122.174000000001 69.952 -11.0161000000003
162.977500000001 50.8883000000003 -18.2452000000003
174.034000000001 67.1104000000003 -11.3193000000004
188.219600000002 51.3183000000005 -10.6030000000004
194.197100000001 52.1652000000004 -15.5402000000004
195.794700000001 61.0742000000004 -9.49520000000038
201.007700000001 50.9188000000004 -22.4562000000004
-1.2500000000007 1.64929999999969 -0.30620000000057
-5.49030000000041 7.40679999999976 -4.71610000000066
65.4774 38.6029999999998 6.48939999999961
109.4245 48.2238999999999 -9.34670000000023
105.9135 63.371 -33.7560000000002
81.5096999999999 15.7896999999998 17.3351999999996
141.941300000001 26.8638000000001 28.3702999999997
190.550069027278 34.3352172680749 28.9371807118507
193.824800000001 57.6680000000004 3.1710999999996
197.407700000001 35.5280000000003 26.0110999999996
200.496700000001 52.2669000000004 -3.82150000000036
-0.904200000000327 0.0387999999997962 -0.634300000000635
-3.03070000000076 -6.27250000000025 -5.10890000000061
73.0064000000003 -10.958 5.69919999999958
118.559900000001 -5.67969999999986 -11.2346000000002
122.489100000001 -20.0410999999998 -35.3056000000002
200.904800000002 14.9640000000004 3.56889999999972
206.142400000002 21.4783000000005 -4.80710000000032
ID=ORYafeUNKUNKMfNB*****2

LM3=54
17.3728000000023 -5.68219999999976 -5.52080000000031

34.070899999996 -11.8563999999996 -5.99899999999975
112.561799999998 -14.0786999999991 -16.1632999999998
136.863999999998 -11.0305999999994 -26.9678999999999
104.413899999998 15.6304000000007 -24.1955999999999
133.890099999997 6.11230000000022 -29.0610000000001
143.294699999998 4.70330000000035 -36.0307999999998
153.6811 -5.02969999999928 13.1747999999998
140.2305 21.4693000000007 -34.1081000000002
162.325000000001 17.7972000000008 -12.4947000000003
173.345200000001 14.6059000000007 -21.2007000000003
159.019699999999 -19.3600999999995 -15.0628000000001
189.242700000002 11.2176000000008 -25.2906000000004
203.665000000001 1.22510000000082 -21.1481000000004
209.917400000002 17.3858000000008 -22.5678000000005
217.255400000002 19.1204000000007 -26.7174000000005
212.441800000003 33.0099000000008 -32.0640000000006
228.670000000003 35.9422000000007 -19.6946000000006
222.120300000002 11.2086000000007 -22.7517000000005
221.798600000002 23.0313000000007 -35.1332000000005
15.1062000000022 6.56620000000058 -5.01900000000032
24.7756000000014 15.6929000000007 -5.92520000000022
101.637099999999 45.0633000000005 -15.1317000000001
126.7851 51.1098000000006 -25.5327000000002
127.8346 33.6807000000006 -29.0981000000002
139.4939 38.6871000000006 -36.1874000000001
145.2719 51.3341000000004 14.1201999999997
159.097500000001 31.6229000000006 -12.4946000000003
169.448100000001 38.0358000000005 -21.3931000000003
145.417100000001 65.9439000000007 -14.0711000000004
183.046100000002 47.3249000000007 -24.4420000000005
193.668900000003 61.4723000000007 -20.9515000000006
206.209800000003 48.1565000000006 -22.5054000000005
213.697100000003 48.3822000000006 -26.1935000000006
216.849700000003 56.9209000000006 -21.9283000000006
219.362400000003 45.8364000000007 -34.8270000000006
19.6311000000013 1.8802000000008 19.5031999999997
15.9016000000018 7.1734000000008 15.8736999999997
88.1079999999995 36.3338000000006 14.7042999999999
130.6062 47.1539000000006 -8.4204000000002
124.914300000001 59.3747000000007 -30.9475000000003
106.807399999999 15.8749000000007 22.0143
164.895800000001 25.1353000000006 20.5959999999997
218.514400000003 33.0256000000008 10.1134999999994
217.722200000003 55.1618000000006 -13.0569000000006
226.322000000002 34.8953000000007 5.9123999999994
221.318700000003 48.9392000000006 -21.1945000000006
20.2171000000011 1.24850000000082 19.1563999999997
17.8527000000009 -4.87999999999925 15.0012999999997
96.043799999998 -9.25059999999924 14.2087000000001
138.858199999998 -3.74369999999949 -10.4411999999999
138.530699999998 -19.5919999999994 -32.5878999999999
224.207500000002 13.8349000000007 -10.4190000000006
225.296300000002 18.2998000000007 -22.1253000000006
ID=ORYafeUNKUNKMfNB*****3

LM3=54
15.1692000000055 -3.75909999999944 -4.14729999999992
29.1514000000051 -11.4879999999993 -4.33019999999997
112.885000000006 -11.5659999999996 -11.4761
138.807300000006 -10.0072999999996 -20.0985000000002
105.825100000005 15.5728000000003 -19.3486
131.937500000005 6.84250000000033 -23.5804000000001
146.527500000005 4.68860000000035 -28.7046000000002
160.339700000006 -4.42229999999936 21.5647999999998
142.993400000005 21.2194000000004 -29.3586000000001
169.657100000005 17.4411000000006 -6.69450000000022
178.391400000006 14.9314000000008 -13.1656000000003
162.313000000006 -20.5508999999994 -8.77490000000021
196.356700000006 8.1596000000009 -17.7112000000003
214.617900000006 -2.41209999999894 -17.2209000000004
221.278000000006 15.5843000000012 -15.8425000000004
226.352800000007 17.8617000000014 -21.3105000000004
220.348000000007 33.0129000000013 -23.8353000000005
236.360065732495 36.9920225504053 -10.1385642101293
232.844100000007 10.8092000000015 -18.2844000000005
231.752500000008 22.1317000000015 -29.1503000000005
13.5575000000055 6.50390000000064 -3.99219999999976
24.608100000005 16.9754000000006 -4.49839999999972
106.790500000004 43.8571000000003 -11.6123
129.578400000005 49.6423000000004 -19.1635000000001
126.758900000005 32.1778000000003 -23.8221000000001
141.929300000005 38.1375000000004 -29.8989000000002
153.620300000005 49.4983000000006 22.0054999999998
167.196400000005 31.6667000000005 -6.59130000000025
174.116000000005 36.9642000000007 -13.1855000000003
147.127400000006 66.0755000000007 -9.01840000000027
190.135200000007 47.720800000001 -16.2260000000004
203.392200000007 63.8995000000012 -16.3458000000005
216.414000000007 49.3592000000013 -15.1739000000005
220.826500000007 49.0138000000014 -19.7070000000005
224.198900000008 57.5363000000015 -17.2425000000005
228.278900000008 46.5813000000015 -28.9163000000005
14.7158000000052 2.43010000000074 22.4227000000003
11.5172000000054 8.36640000000075 18.6587000000003
96.4501000000045 39.1982000000004 21.6257000000001
135.912100000005 45.7633000000006 -4.12440000000016
130.658400000005 58.3146000000005 -26.3382000000001
110.212600000005 16.0776000000004 31.4446
173.191600000006 25.6774000000008 30.4056999999998
223.217800000007 32.7902000000014 21.5040999999996
226.184597432929 56.6339125912517 -2.93376278943172
235.129900000007 34.3636000000015 14.6735999999995
228.306900000008 53.7272000000016 -12.7833000000005
14.9652000000055 1.10040000000076 22.3302000000003
13.8316000000058 -5.20289999999921 18.5038000000002
104.335500000005 -10.1088999999995 21.2162
143.533100000006 -4.64999999999951 -3.42880000000015
141.443300000006 -17.5906999999995 -26.6010000000001

233.813800000007 14.1601000000015 -3.67820000000046
234.489735734861 20.509096768039 -14.6081612382968
ID=ORYafeUNKUNKMfNB*****4

LM3=54
-4.54179999999907 -6.47639999999838 -23.7876000000011
4.18159999999931 -10.1439999999985 -24.0541000000001
83.5956999999965 -6.43940000000009 -25.7518999999996
101.327700000001 -4.56849999999916 -29.3885000000002
76.4240999999979 17.8284000000002 -35.9125999999999
95.5038000000009 11.0568000000005 -37.4528000000003
108.576299999997 10.6262 -40.9031999999996
119.929999999999 1.39510000000045 2.72519999999998
105.719799999999 24.3708000000004 -43.203
125.4527 22.9689000000005 -18.6145
136.4686 22.7520000000006 -25.8041
127.8037 -10.0004999999993 -23.9182000000001
153.3563 18.6770000000009 -29.5715000000002
166.1657 8.51970000000095 -26.2532000000002
175.270200000001 26.3105000000011 -22.9448000000003
180.5609 29.5213000000011 -28.0177000000003
172.403200000001 40.9662000000012 -32.7837000000004
186.925800000001 44.4844000000013 -21.6446000000004
186.1356 22.6885000000011 -24.7027000000002
182.2087 30.5637000000012 -36.6126000000003
-6.50339999999956 3.22260000000134 -23.739200000001
0.0491000000004738 11.1115000000013 -24.3435000000008
72.2505999999991 42.4633000000003 -25.5200000000001
89.2056999999998 48.8093000000004 -28.3881000000002
90.8183999999993 33.1168000000003 -36.5916
101.8106 37.6457000000005 -40.4618000000002
107.481599999999 52.7079000000003 3.21219999999978
122.1379 34.7340000000005 -19.2923000000001
132.397100000001 41.1861000000007 -25.1882000000002
109.5593 65.4471000000004 -24.3483000000003
144.8841 52.4146000000008 -29.2490000000003
152.4646 68.009200000001 -25.2607000000004
168.690600000001 54.9150000000011 -22.9966000000004
173.8116 56.1084000000012 -28.3357000000004
177.0525 63.1000000000012 -24.3071000000005
176.4282 54.5855000000013 -36.6786000000004
-1.09660000000022 0.266200000001117 0.0921999999993388
-4.21810000000011 4.53280000000107 -2.56890000000077
59.1821999999988 34.1235000000004 2.01529999999984
97.4689999999997 44.7362000000003 -17.9335000000002
92.7177999999998 56.6829000000005 -39.9475000000003
76.2025999999984 18.8902000000002 11.0687
127.279899999999 30.5462000000004 12.3482999999998
179.5092 42.7862000000011 3.44019999999967
177.9156 62.1326000000012 -14.2873000000005
186.9786 44.3214000000011 -1.49120000000039
180.6445 57.0420000000013 -26.1978000000004
-0.454200000000257 -0.880099999998905 0.197799999999364
-1.92190000000026 -5.59009999999886 -2.36990000000062

67.2941999999976 -3.91249999999977 2.72130000000016
107.0139 3.37720000000052 -18.2998000000001
108.9384 -10.8662999999992 -39.5751000000001
185.376 23.5307000000011 -13.2445000000003
185.6443 30.7669000000012 -25.9067000000003
ID=ORYafeUNKUNKAMNH119504

LM3=54
-5.31849999999216 -6.893199999997 -22.9183000000012
4.09449999998679 -10.9200000000008 -24.594499999998
88.8445999999998 -7.63269999999905 -28.3588999999995
109.715299999999 -4.32309999999956 -32.6550999999995
80.8383999999971 20.8217000000001 -36.2801999999994
104.824599999997 12.3310000000002 -39.0993999999995
117.097499999999 14.1663000000003 -45.2336999999997
127.263599999997 2.34459999999976 3.94360000000039
112.140899999998 28.9732000000001 -44.0855999999998
134.687899999998 28.0007999999999 -22.7778999999997
146.862399999999 26.3344 -30.9886999999999
137.568799999999 -11.4705999999997 -25.2532999999997
162.8247 22.3967000000003 -32.5962000000001
176.0694 10.0395000000002 -30.7564999999999
184.5785 31.4812000000002 -27.8873000000002
190.541300000001 34.0221000000003 -33.4421000000003
185.040600000001 47.2705000000003 -38.4144000000003
195.362800000001 49.9598000000004 -23.1256000000004
197.102800000001 28.7135000000003 -29.4427000000002
194.978000000001 38.3078000000004 -41.2459000000003
-7.98879999999569 3.05720000000232 -22.6406000000004
-0.910299999998793 11.8690000000016 -23.7290000000001
73.8754999999979 50.1281000000002 -28.0979999999996
91.9459999999986 58.3061000000003 -29.6182999999999
98.0177999999981 39.2695000000002 -38.6558999999997
107.335499999998 43.4165000000001 -44.0080999999998
112.443899999999 59.2043000000005 3.54200000000008
130.892499999999 40.5189000000002 -22.5478999999999
141.234199999999 48.2875000000003 -30.4902
114.6167 76.4940000000004 -26.2765000000001
152.8823 59.0007000000004 -32.1396000000002
159.281800000001 75.5816000000005 -31.1683000000003
174.7574 60.6844000000003 -30.1771000000003
182.563600000001 61.8758000000004 -33.8281000000004
185.489300000001 69.4781000000004 -30.6189000000004
188.487200000001 60.0914000000005 -41.8876000000004
-1.6808408660953 0.624995589254734 0.276006375969077
-6.76669999999906 3.45060000000176 -2.87079999999997
60.6107999999979 39.3213000000004 1.15080000000044
98.5819999999987 56.1191000000003 -21.0824999999998
94.5490999999989 65.6551000000003 -43.9605999999999
76.4715999999976 20.1086000000003 7.23220000000046
134.890699999999 34.9671000000001 11.0339000000001
186.5279 49.1585000000002 2.09739999999981
182.646700000001 67.4559000000005 -20.4681000000004
194.5908 50.7078000000003 -0.467000000000261

189.100300000001 63.7868000000004 -29.0257000000004
-1.57170000000069 -0.580599999998546 0.391400000000301
-3.5565000000011 -6.2328999999986 -3.01699999999964
72.0715999999967 -4.70539999999967 1.01050000000073
112.191699999999 0.0506000000003874 -21.6634999999997
115.168599999999 -10.8696999999996 -44.1524999999995
193.185400000001 29.1596000000003 -21.4930000000002
196.732000000001 36.7281000000003 -28.4408000000003
ID=ORYafeUNKUNKAMNH119524

LM3=54
-6.64610000000627 -6.61650000000217 -29.2229000000005
4.35319999999445 -13.4203000000021 -29.7431000000003
102.600599999996 -5.67270000000144 -35.0663000000001
131.284799999996 -1.14920000000104 -38.0225000000001
94.2060999999958 20.2516999999987 -43.5033000000001
129.975099999997 10.215199999999 -44.0852000000001
139.850399999997 8.22049999999901 -44.8006
150.514799999997 -0.489600000000936 0.188800000000008
137.599499999997 29.5305999999991 -50.972
162.195199999998 27.0588999999995 -24.748
175.044599999997 21.2323999999992 -30.9023999999999
157.007499999996 -12.986800000001 -29.5002000000001
190.886399999996 17.752699999999 -31.6895999999998
208.204199999995 8.4669999999987 -24.5100999999997
218.606599999997 25.912199999999 -21.0135999999997
227.367299999996 28.5466999999989 -25.0445999999997
224.904399999996 45.6614999999989 -34.3808999999996
237.516599999996 46.9260999999987 -11.4347999999995
233.404699999996 21.3039999999988 -18.2322999999997
234.259199999995 32.7515999999987 -33.6896999999996
-10.2080000000065 5.06699999999764 -29.4086000000004
-1.25840000000578 15.4802999999979 -29.7298000000004
91.699099999996 48.3125999999987 -36.0612
120.434599999997 55.988999999999 -38.9675999999999
121.039399999997 44.4239999999991 -44.652
131.654299999997 48.2145999999991 -44.7945999999999
141.588599999997 62.7232999999993 0.452500000000262
158.589299999998 41.9590999999995 -24.6057999999999
169.388899999998 50.3325999999994 -30.4246999999997
139.730999999998 76.5564999999994 -29.2968999999998
182.279999999997 58.7573999999993 -31.4281999999996
195.801599999997 74.4325999999992 -25.2297999999995
211.541299999997 61.7464999999991 -23.4062999999995
219.718699999996 60.8771999999989 -26.0759999999995
224.225299999996 71.8934999999988 -19.4450999999994
229.138699999995 59.6232999999987 -34.3874999999994
-1.77940000000604 0.533399999997799 0.234499999999585
-4.65590000000601 5.55769999999776 -2.97830000000039
78.0614999999956 39.9066999999985 3.85989999999992
128.532299999997 54.9145999999992 -25.7826999999999
123.723499999997 66.3871999999991 -46.0741
97.1702999999969 20.7391999999989 9.26749999999982
162.517499999998 34.1436999999994 17.4196000000001

219.633399999996 45.1348999999988 16.1694000000005
220.835599999996 69.3559999999989 -9.44389999999946
227.256599999996 46.6524999999988 12.8195000000005
227.413199999996 62.8454999999989 -20.3039999999995
-0.774700000006172 -1.55820000000222 0.844499999999667
-2.16320000000642 -6.8593000000023 -1.93680000000034
87.7902999999956 -3.26740000000151 4.03819999999977
138.391199999997 0.00489999999903773 -25.3713
140.488699999996 -10.9694000000012 -46.5985
228.538799999996 18.9033999999988 -8.9265999999997
234.174199999996 29.6357999999987 -20.0904999999996
ID=ORYafeUNKUNKAMNH146722

LM3=54
-6.37449999999511 -7.4901999999975 -31.2040000000008
7.09419999999361 -13.8698000000006 -31.4680999999995
106.488300000002 -6.40049999999905 -34.3697000000002
128.763400000002 -4.72469999999938 -36.7537000000001
101.829300000001 21.8046000000007 -40.5737999999999
126.354000000003 9.26740000000075 -43.2209000000002
141.259400000003 11.9348000000012 -45.8446000000002
154.074899999999 3.40720000000014 4.00680000000007
137.3288 29.2821000000004 -47.2025999999999
163.357800000001 26.4567000000006 -19.0731
175.2847 22.3803000000005 -24.7469
161.425700000001 -12.6197999999994 -27.3085
193.542900000001 21.0323000000007 -26.9711000000001
209.144500000001 7.6872000000008 -20.8065000000001
218.128500000002 27.099200000001 -16.5858000000002
226.296400000002 30.457700000001 -20.1785000000002
220.292800000002 45.9756000000009 -24.2556000000001
233.079700000002 48.4346000000009 -8.75170000000017
231.777700000002 23.333600000001 -12.7668000000002
234.098500000002 34.518100000001 -26.8460000000001
-8.56349999999696 5.16760000000171 -30.8893000000005
1.85120000000091 16.5331000000012 -30.1872000000003
97.2665000000009 50.6764000000009 -32.5135
116.476700000001 57.1830000000009 -35.9698
121.190300000001 44.4449000000008 -41.7975
131.931800000001 48.7332000000006 -43.5733
144.265100000001 61.9238000000008 4.91569999999993
160.615700000002 43.5021000000008 -18.8451
170.386900000001 52.3772000000006 -25.0119
143.418300000002 78.7130000000011 -27.1237000000001
185.935800000002 60.4502000000008 -26.0755000000001
194.479900000002 77.057300000001 -17.5004000000001
210.775500000002 62.4039000000009 -17.5025000000001
220.637500000002 63.794600000001 -19.3836000000002
223.046700000002 71.8093000000009 -11.8421000000002
228.589500000002 62.905000000001 -26.8559000000002
-3.35169999999915 0.50370000000105 0.268399999999805
-6.88939999999833 6.68670000000131 -5.89130000000026
77.3748000000004 42.3414000000008 -0.0533000000000179
124.681800000002 57.9136000000009 -24.2222

125.435100000001 66.946300000001 -43.6824000000001
99.0517000000004 22.7020000000006 14.677
166.341100000001 36.2484000000006 20.0299999999999
215.789000000001 45.2916000000008 21.3131999999999
218.648900000002 68.8451000000009 -2.50870000000015
223.194100000001 46.2924000000009 20.2633999999999
226.042700000002 64.8227000000009 -13.3070000000002
-0.674199999999248 -0.622599999998994 0.309599999999791
-4.31809999999937 -7.39349999999893 -4.99660000000016
86.6102999999996 -4.85969999999958 0.71400000000007
136.153100000002 -0.368799999999201 -22.7004000000001
142.254600000002 -7.32239999999937 -44.1762000000001
226.620800000002 24.2915000000009 -5.40820000000013
232.702500000002 31.966200000001 -14.1867000000002
ID=ORYafeMALUNKAMNH148200

LM3=54
0.224500000006857 -3.98509999999698 -26.1039000000004
11.2094000000087 -9.64599999999999 -28.3873999999999
101.283200000005 -5.21769999999874 -31.3145000000005
123.410800000003 -4.97019999999937 -33.1519000000001
98.4291000000041 22.1234000000008 -38.4152000000003
119.565700000003 12.3425000000011 -40.9484000000001
135.079500000005 12.372100000001 -44.5781000000005
146.780200000003 0.830600000000946 4.69179999999985
130.423000000002 29.3881000000005 -45.777
155.343000000003 25.7277000000006 -21.6329000000001
168.303200000003 24.8708000000005 -29.0302000000002
157.153700000002 -11.8030999999995 -25.0352000000001
186.169600000002 21.1415000000006 -32.0207000000001
201.343900000004 9.60380000000085 -29.7200000000002
212.134900000003 30.4352000000006 -24.3760000000001
218.054900000003 29.6522000000006 -30.9265000000001
211.608700000003 47.2011000000006 -36.8428000000002
229.182900000002 49.8185000000006 -21.6612000000001
225.119800000003 23.9335000000007 -26.1691000000002
224.794300000003 34.7248000000006 -39.1748000000002
-1.62309999999467 4.63990000000191 -26.0020999999999
7.39220000000556 14.6050000000014 -27.4717999999998
87.0033000000036 48.0639000000007 -31.1630000000001
110.730900000003 57.0703000000007 -33.5734000000001
113.810000000003 40.6203000000007 -40.2178000000001
124.896400000002 46.4299000000006 -43.6607000000001
133.688100000003 61.9716000000008 5.2860999999999
152.419300000003 43.5776000000007 -21.6994000000001
163.330000000002 50.0896000000005 -28.2179
138.338100000003 76.6455000000008 -23.5955000000001
178.822900000003 61.4528000000007 -29.9554000000001
187.103000000002 76.7661000000006 -28.9204000000001
202.918100000002 62.6312000000006 -25.4655000000002
210.845000000003 65.9013000000006 -30.4434000000002
213.778700000002 74.2722000000007 -26.0503000000001
218.388300000002 64.3610000000005 -38.7666000000001
0.0765000000051651 0.275800000000997 0.332700000000266

-2.18639999999522 6.05440000000115 -3.09699999999977
77.7261000000047 42.6075000000008 1.34299999999989
116.951000000003 55.8603000000008 -21.3266000000001
116.784200000003 67.2311000000007 -40.4316
98.7477000000048 22.507800000001 14.3597999999998
157.574800000003 35.2994000000009 16.3828999999998
214.362400000003 46.8586000000008 10.7257999999999
214.746400000002 69.6243000000007 -11.9748000000001
221.912000000003 48.4900000000008 8.51769999999985
220.450700000002 65.9393000000006 -23.5284000000002
-0.756099999994817 -1.47979999999899 -0.00519999999974652
1.13080000000546 -7.29569999999896 -2.8216999999998
86.9748000000056 -6.16189999999909 1.43479999999965
130.421600000003 0.273200000000855 -20.7171000000002
132.583500000002 -11.7036999999995 -41.3674
223.347300000003 26.3487000000008 -12.7840000000002
227.257100000003 35.3278000000007 -23.4173000000001
ID=ORYafeFEMDRCAMNH51235*

LM3=54
-1.6654 -6.9472 -28.094
9.927 -11.8862 -29.0267
99.8797 -6.8949 -30.9639
124.3276 -6.0822 -35.17
94.756 20.805 -41.3255
119.3701 12.1964 -42.5303
132.6032 14.6622 -47.5405
148.0589 1.2023 3.9592
129.734 28.7551 -48.7256
155.4503 25.5505 -23.148
164.2945 23.6494 -29.8022
149.4639 -13.7348 -28.006
181.8204 19.4346 -32.7017
195.802 7.8116 -29.8886
205.9808 28.2054 -27.4725
214.7976 29.5647 -31.1695
208.9619 44.7618 -36.5029
222.1152 47.2492 -21.3644
221.9466 20.8296 -26.8014
222.584 32.615 -40.0541
-4.2388 5.5883 -27.4523
3.18 15.7001 -28.8833
90.1559 49.3349 -30.9175
113.0371 58.8057 -35.7168
114.1583 40.3025 -42.4129
128.1702 44.7913 -47.6837
135.3002 62.2283 3.6553
151.525 43.4931 -22.5026
159.4163 49.0842 -29.3678
132.1226 75.3203 -27.9226
174.3071 59.37 -31.8355
182.4751 72.1953 -27.6443
200.4613 59.7497 -27.5507
208.7054 61.205 -31.089

213.0017 72.3539 -26.226
216.5478 61.1399 -40.265
-0.5639 0.412 -0.1826
-4.124 6.2319 -3.0059
76.9753 44.5559 -0.2925
121.8267 55.0154 -25.0037
112.7148 64.816 -48.6921
94.2207 21.4037 14.059
157.6982 36.3553 16.3634
210.3198 45.5147 8.3005
210.5664 68.6975 -16.5244
216.5338 47.1281 4.7703
215.8255 64.0263 -27.3161
-0.2559 -0.8057 0.2034
-0.6794 -7.7283 -2.748
88.8657 -7.6093 -1.0029
132.8114 4.5355 -25.9558
128.4282 -10.7861 -48.8514
219.2214 23.3288 -14.9571
221.2397 31.8765 -25.125
ID=ORYafeMALDRCAMNH51372*

LM3=54
-6.1085 -8.0166 -24.7084
2.8721 -13.5837 -25.6865
97.1927 -7.6661 -28.2453
113.6741 -7.207 -34.2951
83.1918 16.5364 -35.8908
115.8311 7.8741 -39.4681
122.1383 9.6479 -43.3306
139.851 0.6463 2.4948
115.309 23.9561 -44.7484
144.9212 22.1091 -23.3304
154.2713 19.3998 -30.1527
145.0446 -11.2017 -25.1004
170.3797 17.2204 -32.801
183.2367 7.1133 -31.0501
192.1831 24.9353 -29.2617
199.4287 25.5697 -34.2529
190.947 41.7594 -36.8959
204.5578 45.411 -22.536
205.0132 19.8595 -30.7335
203.8622 32.0286 -43.0386
-8.3209 3.534 -25.9979
-1.6561 11.7788 -27.3752
83.935 44.4963 -29.5038
102.3979 51.639 -35.2641
106.0094 36.71 -38.6944
112.5551 38.8579 -41.2299
125.8329 54.9805 2.0558
140.8536 37.295 -23.6858
147.6855 43.9812 -30.0208
125.1896 68.5824 -24.39
161.0333 54.7135 -31.5878

168.6764 69.6569 -31.7284
184.3989 57.4961 -29.4733
191.2746 58.7098 -34.3911
193.7606 67.9956 -29.1568
198.2533 57.6215 -42.576
-0.2113 0.4356 0.1542
-5.3827 4.523 -3.5842
68.2309 36.8819 1.7652
111.5917 47.792 -24.1779
106.6217 58.0844 -42.3173
92.5071 19.1018 12.5154
147.3523 31.0655 12.3308
191.4863 40.6774 6.9136
193.0153 63.5001 -14.3368
199.6217 42.6217 2.8417
199.9247 60.5157 -26.7812
-0.4707 -1.2887 0.3799
-2.8782 -6.7337 -2.1471
79.5531 -7.7425 2.1875
123.2271 0.5602 -23.5311
120.7684 -11.5306 -43.3781
203.3791 23.3332 -16.1262
207.6523 30.3711 -28.0243
ID=ORYafeUNKDRCAMNH51906*

LM3=54
-11.1610000000226 -9.94060000000697 -27.9484999999968
-0.543300000021824 -13.1790000000068 -29.3907999999964
90.0761999999938 -10.3552000000023 -34.630199999999
113.198499999997 -6.56710000000138 -37.2648999999996
85.1871999999943 20.055799999998 -43.7521999999991
108.606999999999 10.4280999999991 -46.1913999999998
121.3403 9.25289999999943 -49.6093
134.633199999999 -1.42210000000097 0.958600000000314
117.0989 26.8339999999996 -50.3267
143.555400000003 22.8395000000002 -25.0732000000004
155.473800000004 20.7406000000004 -33.4907000000005
138.9236 -14.4254000000008 -28.5090999999999
172.786800000005 18.5002000000005 -36.2995000000006
187.015900000003 5.46849999999991 -32.9607000000003
197.147900000004 26.8558000000002 -31.6378000000003
203.024300000005 29.5876000000004 -35.8576000000005
196.713400000005 44.0941000000006 -38.6603000000005
211.979000000004 47.1184000000003 -26.1098000000004
209.937300000004 22.2070000000001 -31.5335000000004
208.830100000005 34.3602000000004 -44.4405000000005
-14.8396000000228 3.164799999993 -27.6276999999963
-6.79690000002227 12.2146999999932 -29.2200999999964
77.0611999999933 48.3633999999979 -34.7497999999989
99.3111999999976 56.4246999999991 -38.1715999999996
100.990599999998 37.5404999999991 -45.7980999999997
113.6046 44.0723999999996 -49.3067
120.307199999999 57.6943999999994 1.66180000000038
139.114700000003 41.0558000000003 -24.7802000000003

149.100900000003 47.3285000000004 -31.3894000000004
120.6313 72.9984999999998 -27.9232999999999
163.511300000004 57.9846000000006 -35.2429000000004
171.609700000004 76.4095000000005 -32.4882000000004
189.395000000005 59.7155000000007 -30.2552000000005
196.843900000005 59.7812000000007 -35.8316000000005
199.443900000005 69.8135000000006 -31.1503000000005
203.990800000005 59.4838000000006 -43.9689000000005
-5.63592723469976 -0.295423428929033 -1.3997193853721
-11.7959000000232 5.30669999999284 -5.45369999999613
64.6905999999896 43.8659999999967 -3.17829999999814
108.427799999999 54.0850999999994 -25.6604999999997
109.040399999997 66.254199999999 -45.2224999999995
84.1630999999912 18.8863999999971 10.1827000000016
143.6072 32.0815999999994 13.0491000000003
201.925300000003 43.7489 5.84749999999989
200.488100000004 65.9057000000004 -16.1608000000003
208.989100000004 44.9131000000001 1.09609999999984
203.537100000005 64.0392000000006 -26.4560000000005
-5.55244360716895 -2.55561511100492 -1.2186247893594
-5.80914406904556 -10.1205581134199 -5.46048282618142
74.3724999999896 -11.4849000000035 -3.16599999999819
120.730899999999 -2.40880000000093 -26.0124999999998
125.900399999998 -12.0866000000014 -46.1050999999997
207.201200000004 26.0406000000001 -20.4777000000003
211.304000000004 30.2017000000003 -27.0996000000004
ID=ORYafeFEMDRCAMNH51910*

LM3=54
1.2091 -4.7352 -23.3694
11.1397 -9.1562 -23.8012
86.7207 -8.5816 -25.4503
107.3327 -6.2273 -29.607
84.3312 18.602 -36.6591
106.2623 8.9604 -38.028
116.9817 10.2465 -43.6313
128.4695 0.8979 3.6655
112.5645 24.9401 -43.6691
134.4273 21.8675 -21.0754
145.1844 20.0834 -29.7184
133.4621 -12.1795 -25.918
160.9882 16.4949 -31.9249
175.9969 2.9949 -27.6717
180.5895 23.4355 -27.3312
187.0394 26.24 -31.3717
182.5478 39.5478 -36.7225
195.2734 43.2152 -22.4619
194.3624 19.6658 -27.0035
191.9561 29.4153 -38.6354
-1.1989 4.9467 -23.3909
5.8695 13.205 -23.9449
76.386 45.2701 -26.1218
96.0176 51.7914 -29.945
99.3443 37.0261 -37.7617

108.6814 39.3338 -41.6315
117.7887 54.4413 2.8703
130.4987 38.3708 -21.1338
139.2144 44.1906 -29.5541
116.2059 67.6361 -26.2574
153.7929 53.6072 -31.6455
161.796 70.494 -27.629
174.7239 54.1706 -27.4978
181.2052 54.3474 -31.335
186.5771 62.6303 -26.1151
187.4446 52.6959 -38.3094
-0.5191 0.6772 0.2464
-1.993 6.0319 -3.9396
63.5393 35.3084 1.9743
102.206 47.7254 -18.3847
99.6699 60.5416 -40.935
82.9689 18.9814 10.1622
136.9841 31.504 11.8038
184.2309 40.2561 3.1579
185.9217 61.4925 -15.0314
193.8837 41.9936 -1.2839
187.8308 56.0263 -24.5507
-0.2427 -0.8825 0.4445
0.7145 -5.8058 -2.376
72.3491 -4.9269 2.0992
112.6249 0.4118 -18.5602
112.5903 -11.0571 -40.7361
192.0975 20.288 -17.9055
193.8199 27.3556 -25.4337
ID=ORYafeUNKSAFAMNH69549*

LM3=54
-4.3058 -7.5341 -24.14
4.428 -11.274 -24.2309
80.6289 -8.3846 -27.2909
101.3631 -5.8084 -31.7929
72.1037 16.357 -35.2565
99.783 9.7382 -37.8945
110.4384 10.1775 -44.3991
117.8852 1.1153 2.5058
105.4013 24.7466 -45.2387
124.317 23.1763 -21.5667
136.7279 19.1427 -29.3001
126.6496 -12.3376 -22.0569
150.8079 18.0068 -31.939
166.4536 6.7432 -30.0005
172.3244 26.2978 -29.6029
177.9533 29.4065 -34.3274
169.5644 40.4885 -37.9548
183.6817 44.6606 -24.3298
184.5939 22.6599 -29.4384
180.5097 30.9089 -41.9883
-7.3688 3.5703 -24.2742
-1.2046 10.5325 -24.9085

69.2506 43.1704 -29.13
88.0878 49.2237 -33.1662
93.0671 33.912 -39.8827
102.6701 40.5784 -45.1135
106.4429 52.9256 2.0316
121.6741 34.2766 -21.9115
131.4493 41.6075 -29.6176
107.4704 68.3249 -23.7692
142.0371 51.6062 -32.3007
150.4848 69.2215 -30.1685
163.7038 54.1296 -29.3215
170.4718 55.0896 -33.9204
172.9745 63.4322 -29.5443
174.3252 54.1939 -42.4472
-0.9552 0.6122 -0.0259
-6.4635 4.2486 -4.4452
56.767 34.7276 -2.2369
93.4308 48.1529 -20.3161
90.6939 61.0664 -40.4557
70.2147 17.4967 4.6697
125.9681 30.6575 10.385
176.2658 42.7486 -0.5035
174.0149 61.3022 -19.2739
181.5673 45.3638 -4.3993
178.6743 57.6039 -29.0692
-1.2448 -1.0716 0.7112
-3.7482 -6.6684 -3.8489
65.3352 -4.9993 -1.0203
106.5127 0.6454 -18.633
107.9582 -12.6746 -40.2844
183.3948 25.1114 -19.9155
186.052 32.8921 -28.4857
ID=ORYafeUNKZOOAMNH70036*

LM3=54
-1.848499999995 -5.64860000000056 -23.6151000000006
7.05790000000772 -10.5017999999997 -24.6624000000009
91.3549999999998 -7.49520000000117 -28.9824999999995
113.397000000002 -5.56060000000011 -32.9388999999999
87.967100000002 20.9805999999995 -38.6381
111.705600000002 11.9911999999998 -40.0045999999997
123.185700000002 11.2262999999997 -44.2359999999998
131.834000000002 1.16539999999967 4.37980000000018
118.370500000002 28.2939999999998 -46.1873999999998
143.390000000002 24.8542999999998 -22.6623999999998
154.801800000003 21.7023000000001 -31.0236999999999
140.054700000002 -12.3491000000004 -25.2445999999998
172.055800000003 20.5863000000001 -31.3455
184.492400000003 7.72679999999993 -27.2729
193.133500000002 28.9606999999998 -26.4305999999998
199.348600000003 31.5307999999998 -29.1784999999999
192.560300000003 45.0352999999999 -34.7343999999999
207.756200000003 48.5721999999998 -19.7775999999999
204.961800000003 24.2423999999998 -24.1652999999999

204.708700000003 35.0365999999998 -37.2151999999999
-3.67179999999545 5.1324999999994 -22.7162000000001
2.72500000000477 14.1282999999995 -24.0438000000002
78.2122000000023 48.3202999999997 -30.2594
99.2624000000018 55.6364999999997 -34.7676999999999
106.244700000002 41.1619999999998 -40.5867
114.629000000002 44.5394999999997 -43.8934999999999
117.793100000002 58.4372999999997 3.7988000000002
139.482800000001 42.2430999999997 -22.7839999999997
147.470800000001 49.1184999999997 -30.8874999999998
118.957900000002 75.2572999999997 -26.5558999999999
162.022900000002 59.1798999999998 -31.8784999999999
168.787500000002 77.4307999999997 -28.0605999999999
184.624600000002 59.5638999999997 -25.8393999999999
191.931600000003 61.6702999999997 -29.5366999999999
193.862100000003 70.4851999999997 -24.8102999999999
198.364400000003 60.0020999999997 -37.4229999999999
-1.77189999999495 0.855099999999483 0.420499999999785
-4.04659999999515 6.20859999999946 -2.52980000000022
65.6596000000024 42.4944999999996 2.48790000000003
106.903700000002 54.8153999999997 -22.0579999999999
103.184300000002 66.0734999999997 -40.1493999999999
87.4475000000019 22.9310999999995 11.4915000000002
141.959500000002 34.2880999999996 13.8487000000003
192.718100000003 46.7003999999997 9.12910000000017
192.157000000003 68.9435999999997 -15.1907999999999
197.998300000003 46.9405999999997 6.92230000000012
199.522700000003 62.9025999999998 -22.0044999999999
-0.655799999994921 -0.685100000000471 0.795899999999763
-0.864899999994743 -6.21210000000048 -3.09260000000032
78.3832000000019 -6.78730000000066 4.57210000000016
119.484600000002 -1.41110000000028 -21.2729999999997
121.218600000002 -13.4772000000003 -38.2325999999998
201.790400000003 25.5597999999998 -14.1910999999999
204.021700000003 33.0998999999998 -20.8733999999999
ID=ORYafeFEMETHAMNH70189*

LM3=54
-2.26449999999081 -6.87689999999789 -26.7112000000006
10.6136000000099 -11.398699999998 -27.8587000000002
96.1052000000089 -6.0299999999983 -31.0822000000005
116.127100000008 -3.71469999999851 -31.9327000000004
88.2237000000081 20.2429000000014 -38.8741000000007
108.749800000008 11.7593000000013 -40.1542000000005
123.529400000008 12.9496000000014 -44.0002000000005
138.756900000007 2.08280000000143 5.12839999999983
117.595900000008 27.8866000000014 -47.4660000000006
144.913700000006 25.7292000000012 -20.5646000000003
157.928200000006 25.1568000000013 -28.5061000000002
148.729000000008 -10.5608999999983 -25.5771000000003
173.201200000006 21.9498000000014 -32.0186000000001
191.008700000007 10.4501000000016 -25.8721999999999
196.418500000006 29.2736000000016 -25.8122999999999
203.304700000006 32.6915000000016 -30.3068999999998

195.781000000005 46.7154000000013 -35.3257999999998
211.451200000005 50.9254000000015 -19.3829999999998
209.060900000006 27.0511000000016 -26.5873999999997
208.595200000005 37.5493000000014 -38.6028999999997
-3.5613999999903 3.69950000000195 -27.8204000000002
5.10950000000923 13.0227000000018 -28.2747000000002
83.5339000000071 50.2582000000013 -29.9526000000006
99.8580000000072 55.1253000000014 -31.1503000000007
102.374200000007 38.9015000000013 -40.4883000000006
114.810100000008 45.0391000000014 -44.2527000000007
124.090600000007 58.3576000000014 4.87079999999942
140.274600000006 40.4066000000011 -21.8114000000003
151.704100000006 48.1879000000011 -28.4180000000003
127.706700000007 75.8204000000013 -24.1097000000006
163.207500000005 57.5418000000011 -31.5499000000002
172.649100000005 75.3875000000012 -25.2365000000001
188.123400000005 61.0070000000012 -24.8432999999999
194.614400000005 62.8435000000013 -29.7589999999999
196.895600000005 71.3830000000014 -24.9757999999999
201.147100000005 61.8763000000014 -38.3240999999999
-2.38929999999039 -0.314999999998003 -1.19480000000025
-7.20169999998991 4.04840000000206 -5.07260000000034
64.3840000000078 38.5485000000014 -2.78460000000051
108.840300000007 55.1314000000012 -19.7242000000006
101.923400000007 65.8105000000014 -41.9730000000007
86.1343000000076 20.8445000000014 8.87349999999955
143.331700000006 33.2802000000013 15.3826999999998
198.209700000006 48.0826000000016 9.08620000000017
193.403300000005 67.4705000000014 -13.4155999999999
206.002400000006 49.8356000000017 3.18210000000021
197.578700000005 64.7456000000014 -22.2569999999999
-1.5259999999903 -0.56459999999803 -0.847900000000235
-3.95749999998983 -7.11639999999795 -5.65590000000027
76.0530000000082 -5.65719999999841 -1.92220000000038
122.381200000007 1.52780000000145 -18.9435000000004
121.277000000008 -11.3129999999984 -43.0452000000003
205.256300000006 29.0122000000016 -15.4845999999997
207.371200000006 35.2561000000016 -22.8729999999997
ID=ORYafeUNKZAMAMNH89852*

LM3=54
-2.17470000000436 -4.19680000000149 -26.1959999999981
10.0255999999963 -8.86180000000127 -26.3573999999982
96.618899999998 -4.5131000000001 -29.4194999999991
120.338899999998 -2.32270000000003 -31.7956999999991
94.3102999999985 22.5780000000001 -35.9982999999989
119.373799999999 12.0810000000002 -38.2154999999992
126.865299999999 12.0186000000002 -42.2847999999991
141.196499999997 1.15129999999964 2.91520000000099
124.0796 28.5579000000003 -43.8619999999991
150.970799999998 26.2847999999999 -21.623399999999
162.178499999998 25.3925999999997 -28.7744999999989
147.518699999998 -9.73520000000026 -26.0229999999992
179.311699999998 20.7229999999995 -31.9207999999988

193.046699999997 10.2564999999993 -27.1793999999988
200.192799999997 27.707199999999 -26.6779999999985
207.483499999997 31.265999999999 -32.5330999999985
202.498499999998 45.4569999999992 -37.7523999999984
215.977099999997 48.0626999999987 -20.6423999999982
212.257599999997 22.7185999999989 -26.2607999999984
214.847299999997 34.6174999999989 -39.1468999999984
-4.23370000000424 5.08899999999854 -26.146399999998
5.283499999996 14.6812999999987 -26.415999999998
84.9229999999976 47.8262999999998 -28.5414999999985
108.564299999998 55.0847999999999 -31.0477999999985
111.143499999998 40.4889000000001 -37.6493999999988
121.204199999999 43.3738000000001 -41.8829999999988
128.072899999997 60.0041999999996 4.13880000000162
147.172599999998 41.7048999999999 -21.1232999999988
157.171299999998 48.3563999999998 -28.6529999999986
129.953399999999 73.7312999999999 -25.6317999999984
171.023499999998 58.0924999999995 -31.5000999999984
178.897599999998 73.4578999999993 -24.9340999999981
194.308499999998 60.8979999999992 -25.5127999999983
201.467299999998 61.1151999999991 -31.2405999999983
203.064799999998 70.7575999999989 -24.5516999999981
207.638226215161 60.1008267826082 -38.8909597223874
-1.06240000000468 0.336999999998466 -0.152299999997957
-6.67800000000463 5.04779999999843 -3.39649999999792
72.0242999999964 38.6677999999994 0.978900000001637
114.860099999998 54.8592 -18.3230999999986
111.007999999999 63.0624999999999 -40.1651999999985
89.491299999997 21.1550999999996 10.5416000000013
149.857199999997 32.9358999999994 14.4752000000014
204.013999999997 45.0333999999989 8.40160000000172
202.614399999998 64.8527999999989 -13.7229999999981
209.738499999997 45.9948999999988 5.09300000000182
207.521399999998 62.7080999999989 -23.8135999999981
0.0620999999951231 -0.352400000001592 0.433900000002057
-4.20380000000459 -7.28370000000155 -3.740999999998
81.4660999999973 -3.53500000000039 1.54890000000113
126.980199999998 1.14750000000001 -18.3521999999992
126.594699999998 -10.1233000000001 -39.9391999999991
209.723199999997 26.1483999999989 -13.7340999999985
213.545199999997 31.0625999999988 -23.8904999999983
ID=ORYafeUNKZAMAMNH89853*

LM3=54
-0.179899999987752 -6.33619999999724 -25.1486000000024
9.71530000001177 -10.5474999999973 -25.0890000000022
92.5125000000072 -5.2167999999979 -28.3063000000014
115.514900000006 -2.68619999999817 -32.7532000000011
88.181100000006 21.3102000000018 -36.6525000000009
113.858200000005 13.1304000000016 -40.1096000000009
123.704700000006 13.4613000000016 -43.3570000000009
136.903900000006 3.72260000000172 4.70419999999898
120.962000000005 29.2677000000014 -43.9651000000006
143.275100000006 28.1617000000014 -21.8088000000007

153.935200000006 24.5009000000013 -28.8342000000008
143.468600000007 -9.61519999999796 -22.4222000000013
171.891100000007 22.3308000000014 -30.4100000000009
187.045000000008 11.8771000000016 -24.8552000000011
189.883800000007 29.8120000000014 -26.4855000000009
195.932800000008 33.4562000000014 -29.305200000001
190.979100000007 46.4404000000012 -34.0168000000007
205.098900000008 50.4262000000014 -18.4041000000008
204.194900000009 26.9867000000016 -22.7855000000011
201.382800000008 37.2684000000015 -36.764500000001
-3.28379999998713 4.85950000000285 -25.5529000000024
4.32470000001224 13.5527000000027 -25.1684000000022
79.9832000000059 46.8981000000015 -29.1030000000007
102.240700000005 55.6077000000013 -33.3193000000004
107.304900000005 41.4247000000013 -39.5105000000005
115.237300000005 44.8649000000012 -43.2228000000004
120.031500000005 60.5773000000012 4.3574999999996
139.633900000005 41.7156000000011 -21.8914000000004
147.346100000005 49.3762000000009 -28.7148000000003
123.059900000005 75.933200000001 -22.7580000000002
163.191800000005 60.6638000000007 -30.0538000000003
171.645900000006 76.2067000000009 -25.0490000000003
183.206000000006 62.020000000001 -25.6961000000005
189.914100000007 62.1872000000011 -29.4677000000005
193.139500000007 70.5264000000011 -23.6034000000005
195.740900000007 60.3606000000012 -36.2764000000006
1.46950000001239 0.671200000002723 1.01909999999772
-2.07879999998753 4.88000000000274 -2.7113000000023
68.989000000007 39.4559000000018 1.83739999999901
110.534000000005 52.8893000000013 -19.5693000000004
105.941600000006 65.6137000000012 -41.4377000000004
88.5885000000065 21.6084000000018 11.2308999999991
142.908600000005 35.6144000000012 13.9140999999994
192.622300000008 47.1826000000015 7.77619999999919
193.925600000007 65.0391000000012 -12.2627000000005
203.078300000009 49.2861000000015 3.44839999999918
196.588200000007 64.3717000000012 -19.4859000000005
1.09400000001247 -0.273199999997265 0.873099999997656
0.707000000012496 -5.96919999999726 -2.70570000000231
81.2006000000074 -4.43159999999793 1.18319999999865
122.177500000006 2.16750000000174 -17.664100000001
125.020000000007 -10.759999999998 -41.3623000000012
201.879900000009 28.2979000000016 -13.5636000000011
203.850000000009 35.2924000000015 -19.468400000001
ID=ORYafeMALBOTUSNM470175

LM3=54
1.4588 -5.2696 -22.4155
7.9292 -8.7433 -24.2479
91.1527 -6.6249 -27.3051
114.9413 -3.1774 -32.0331
86.8299 20.9812 -36.0871
107.732 12.6158 -38.6375
119.8689 12.3627 -42.4779

136.8553 1.062 2.9275
116.6024 27.7768 -43.4565
142.8688 26.5885 -22.9595
152.0013 23.4485 -31.0636
138.3858 -10.6929 -25.379
165.9028 19.0299 -34.2076
183.5514 7.7642 -28.1825
189.2795 27.1979 -29.5452
193.2771 31.714 -33.6329
187.6589 44.0765 -37.7316
203.4926 48.5846 -23.927
199.6089 23.8661 -28.8243
197.0795 33.6333 -40.3918
-0.7387 5.6583 -22.2559
3.4093 11.5917 -23.2383
78.6221 46.2344 -25.6871
101.9071 54.506 -30.3155
102.8895 37.6295 -36.7659
115.2437 42.1692 -42.1837
121.983 59.8011 3.4916
139.3477 40.8131 -22.9694
145.8701 48.2008 -29.9588
119.7014 72.1712 -24.5944
156.5808 58.2416 -33.1503
167.2049 75.9619 -28.0654
180.2268 58.3685 -28.1941
187.4692 58.6062 -32.6849
190.0129 67.4907 -27.5308
191.1036 58.5143 -39.3788
-1.1975 0.4882 0.3655
-1.9939 5.5496 -2.3326
68.4221 37.1684 3.4089
109.5201 49.5837 -17.794
101.5575 61.617 -37.2471
88.6509 20.7206 11.3007
145.3989 33.9818 14.507
191.8435 45.7386 4.882
190.5429 65.7967 -16.1263
200.3114 47.0684 -0.3857
192.2895 63.1117 -24.5816
-0.8725 -0.815 0.2252
0.6542 -6.2581 -3.1992
78.8887 -3.4657 2.6414
119.775 3.0274 -18.2554
118.7277 -9.7587 -39.184
201.0156 25.0837 -18.5287
200.2058 29.8011 -25.1261
ID=ORYafeFEMZIMUSNM470533

LM3=54
-1.97879999998278 -7.10849999999496 -27.1643000000032
14.6895999999934 -13.2341000000004 -29.1012999999993
97.1437999999977 -8.25710000000007 -32.7240000000003
127.6252 -5.50229999999957 -36.9706000000005

95.209699999999 21.9202000000003 -41.5586000000001
125.513299999998 12.9379000000002 -44.1651000000003
136.233799999998 12.8865000000001 -48.6756000000001
150.038599999997 0.461300000000022 2.96119999999993
133.762599999999 29.6627000000001 -49.8911000000002
158.335699999997 25.2759999999998 -25.1644
172.150199999998 23.9403 -32.5809000000003
155.537199999999 -14.4851999999996 -29.4795000000005
186.236999999996 20.1223999999997 -36.5107000000002
204.556699999997 7.1065999999999 -33.4214000000003
211.772799999998 27.8894000000001 -34.3684000000005
217.424599999998 32.2014000000002 -37.7100000000006
211.849999999997 47.0462000000001 -41.8041000000005
225.564699999997 49.8761999999999 -28.7986000000005
225.052399999997 24.7504000000001 -33.5232000000005
223.385799999998 34.8645000000001 -47.6435000000006
-4.58449999998826 5.90010000000386 -26.9708000000023
9.44790000000776 19.0940000000028 -29.4813000000015
82.4457000000004 48.4700000000008 -32.9908000000003
112.5249 60.0916000000005 -35.1777000000003
118.116499999999 42.6326000000003 -42.5775000000002
127.360799999999 45.7753000000002 -47.4635000000002
136.330199999999 63.4980000000006 3.53769999999966
154.172099999998 44.6993000000001 -24.6869000000003
165.342099999999 51.6568000000002 -31.9953000000003
134.6931 79.6208000000006 -29.0567000000005
178.485999999998 61.9705000000002 -35.4507000000004
189.848199999999 80.9615000000004 -32.2458000000006
203.677299999998 64.8024000000001 -33.9620000000005
210.429299999998 63.4322000000002 -37.6135000000005
215.098299999998 72.9007000000002 -32.8001000000006
218.056899999998 63.3317000000001 -47.5772000000006
-0.29659999999446 0.979500000002473 0.837099999998775
-2.26899999999287 6.86580000000283 -2.03370000000148
78.3078000000001 42.2981000000009 4.17519999999969
120.043899999999 58.1932000000004 -23.1244000000002
117.3163 69.8994000000006 -46.7721000000003
92.7047999999983 20.6215000000004 8.20419999999981
160.234799999998 35.5834000000001 15.2651999999997
215.567899999997 46.4315000000001 8.4394999999995
214.543699999998 70.9389000000002 -16.0425000000006
223.239099999997 47.8912000000001 3.88709999999948
218.023699999997 67.7049 -28.7074000000006
0.572800000005398 -0.54309999999753 1.10009999999882
0.352200000005216 -6.81489999999756 -2.29000000000116
87.2212999999973 -3.50459999999986 4.78440000000003
134.006099999998 0.0657000000001979 -23.1843000000003
134.175199999999 -13.2453999999997 -47.3638000000005
223.135299999997 24.7302 -17.4355000000005
225.282799999997 30.7555000000001 -27.3746000000005
ID=ORYafeUNKCIVUSNM477345
