# Supplementary material for: Flexible conservatism in the skull modularity of convergently evolved myrmecophagous placental mammals
Source: BMC Ecol Evol. 2022 Jun 30;22:87. doi: 10.1186/s12862-022-02030-9 (PMC9248141; doi:10.1186/s12862-022-02030-9)

Figure S1 – Homologous anatomical landmarks used across the skull in (A) ventral, (B) dorsal, and (C) lateral views. A detailed list of landmarks, as well as their affiliation to the ten a priori architectures tested, is given in Table S2. The architecture presented here corresponds to architecture 1.

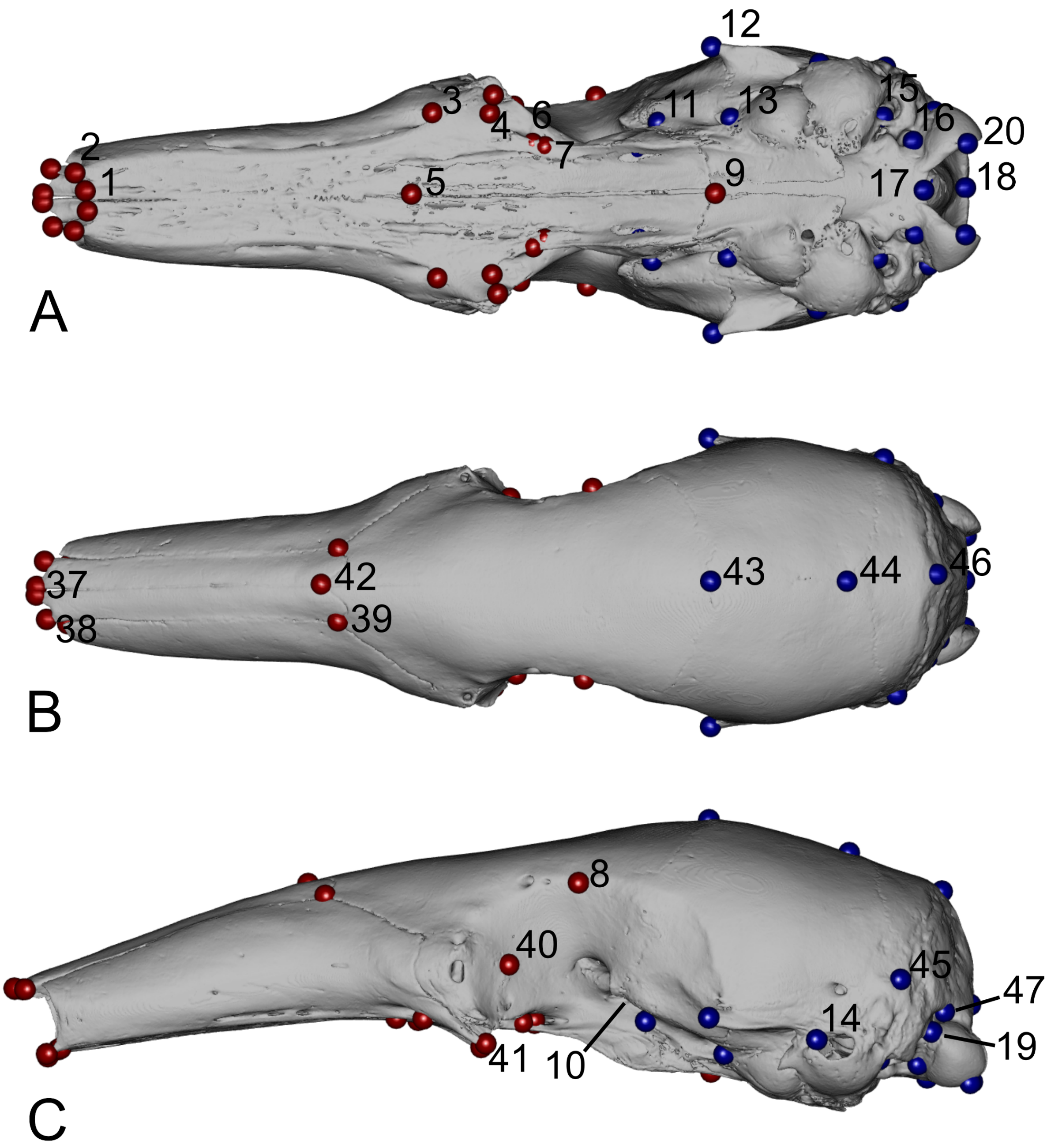

Figure S2 – Clusters retrieved with EDMA for *O. afer* (A) and *S. gigantea* (B). Colors of landmark configurations do not correspond homologous anatomic clusters (left). Gap statistics estimation (*y*-axis) according to the number of defined clusters (*x*-axis) for *O. afer* (A, right) and *S. gigantea* (B, right). Black arrows indicate the lowest *k* value for which the Gap statistics stabilize.

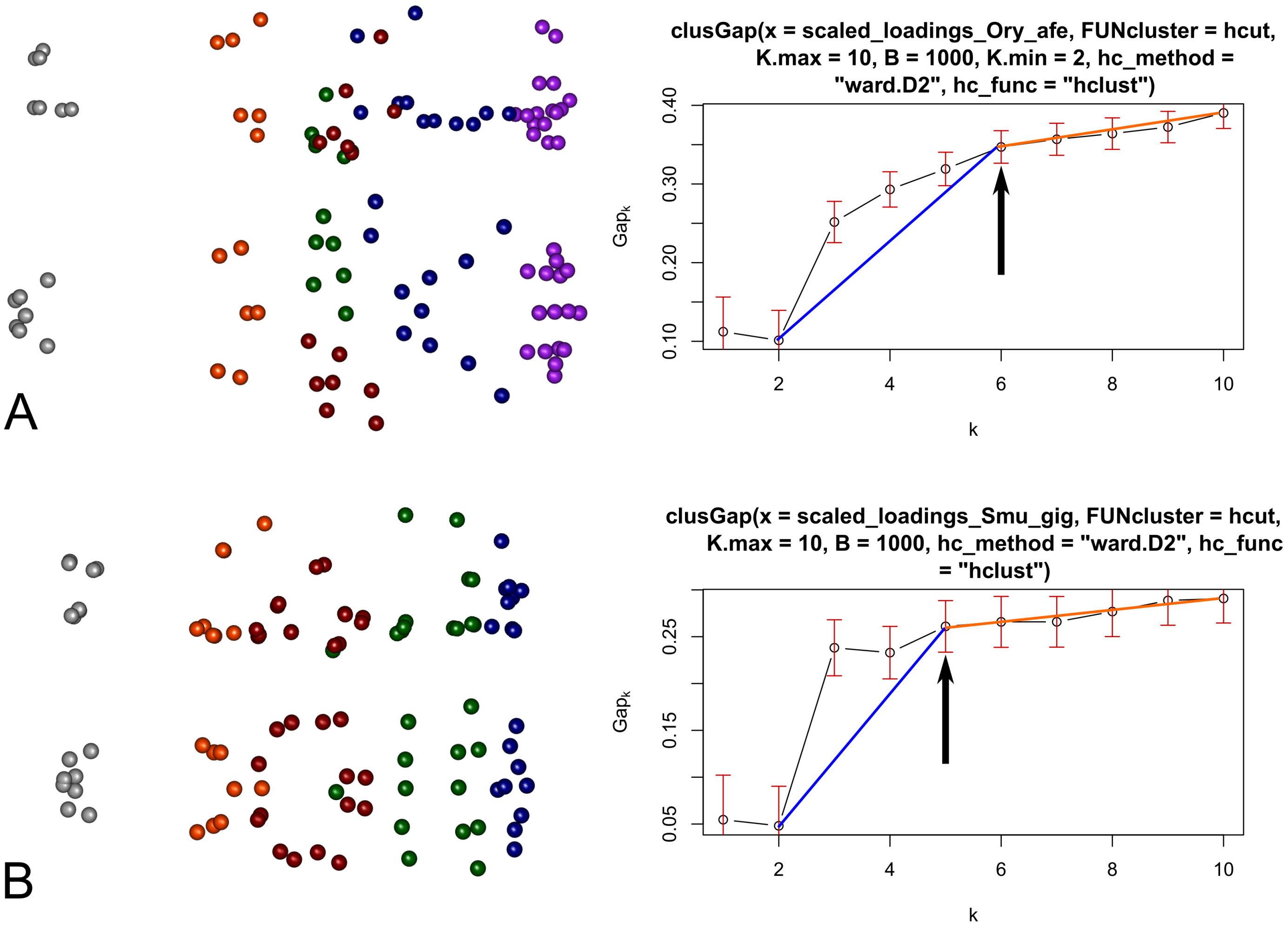

Figure S3 – Results of EDMA for all 13 myrmecophagous placentals included in this study. Spheres (left) represent the landmark configuration for each species, colored according to the recovered hierarchical structure (right). Colors do not correspond to homologous units between species or to the colors adopted in the main text figures. The species name and the number of cluster identified by the Gap statistics is given below each item.

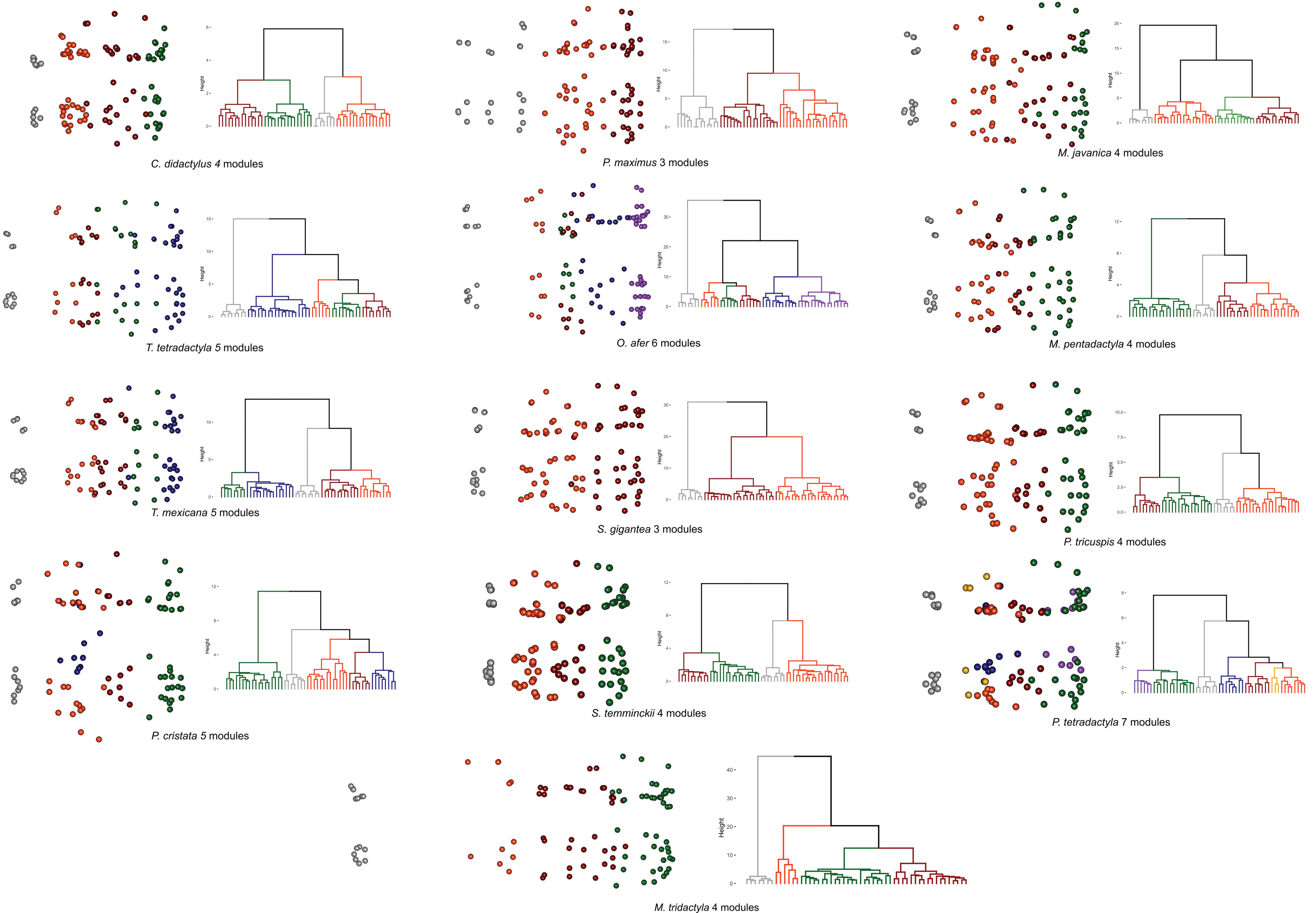
C. didactylus 4 modules
P. maximus 3 modules
M. javanica 4 modules
T. tetradactyla 5 modules
O. afer 6 modules
M. pentadactyla 4 modules
T. mexicana 5 modules
S. gigantea 3 modules
P. tricuspis 4 modules
P. cristata 5 modules
S. temminckii 4 modules
P. tetradactyla 7 modules
M. tridactyla 4 modules
Height

Figure S4 – EDMA results (above) and the naso-palatine module (below) in *T. tetradactyla* (A) and *O. afer* (B). Landmark color corresponds to the therian six-module (Goswami, 2006; A) and Hallgrimsson (2004; B) six-module architectures to which the naso-palatine was added, resulting in architectures VII (A) and VIII (B), respectively.

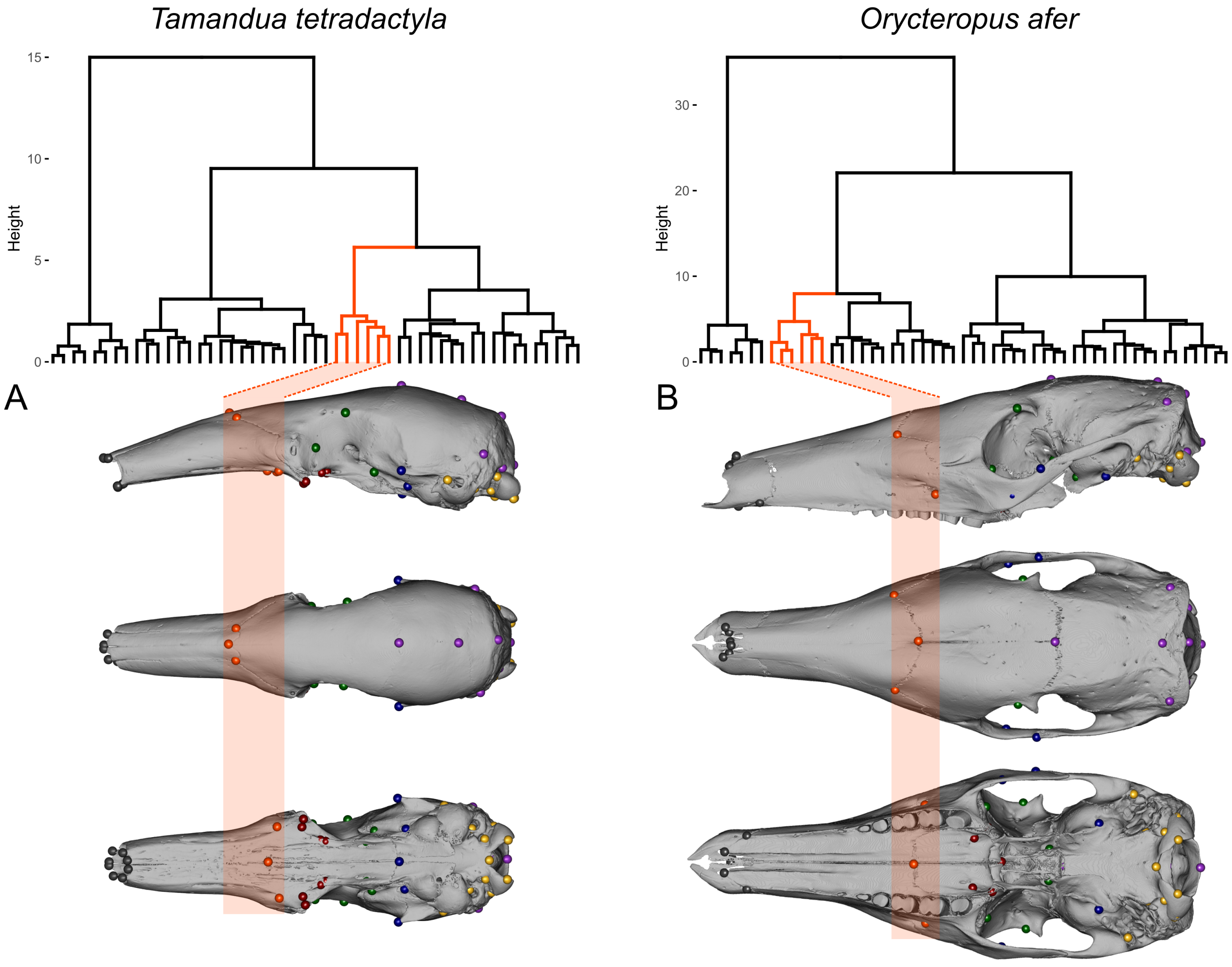

Supplement: Supplementary file 2 — Additional file 2: Figure S1. Homologous anatomical landmarks used across the skull in (A) ventral, (B) dorsal, and (C) lateral views. A detailed list of landmarks, as well as their affiliation to the ten a priori architectures tested, is given in Table S2. The architecture presented here corresponds to architecture 1. Figure S2. Clusters retrieved with EDMA for O. afer (A) and S. gigantea (B). Colors of landmark configurations do not correspond homologous anatomic clusters (left). Gap statistics estimation (y-axis) according to the number of defined clusters (x-axis) for O. afer (A, right) and S. gigantea (B, right). Black arrows indicate the lowest k value for which the Gap statistics stabilize. Figure S3. Results of EDMA for all 13 myrmecophagous placentals included in this study. Spheres (left) represent the landmark configuration for each species, colored according to the recovered hierarchical structure (right). Colors do not correspond to homologous units between species or to the colors adopted in the main text figures. The species name and the number of cluster identified by the Gap statistics is given below each item. Figure S4. EDMA results (above) and the naso-palatine module (below) in T. tetradactyla (A) and O. afer (B). Landmark color corresponds to the therian six-module (Goswami, 2006; A) and macaque phen-gen (Hallgrímsson, 2004; B) six-module architectures to which the nasopalatine was added, resulting in architectures VII (A) and VIII (B), respectively. [file 12862_2022_2030_MOESM2_ESM.pdf]
