# Supplementary material for: Flexible conservatism in the skull modularity of convergently evolved myrmecophagous placental mammals
Source: BMC Ecol Evol. 2022 Jun 30;22:87. doi: 10.1186/s12862-022-02030-9 (PMC9248141; doi:10.1186/s12862-022-02030-9)
Supplement: Supplementary file 3 — Additional file 3: Table S1. List of myrmecophagous species included in this study. Number of specimens (N). Table S2. List of homologous anatomical landmarks used across the data set. Columns I to X show the affiliation of each landmark to the ten a priori architectures tested in this study (Table 2). Table S3. Number of clusters resulting from the EDMA for each myrmecophagous species and minimum Jaccard coefficient per number of modules (k) calculated from a 10,000 bootstrap. Number of specimens (N), value of Gap statistics (Gap) for the optimal number of clusters (Cluster), and final number of clusters after removing biologically meaningless cluster (Final). Table S4. Modular architectures of 13 myrmecophagous species. Number of specimens (N), most likely modular architectures recovered with EMMLi (MLi), covariance ratio (CR), within-module absolute correlations (ρ), correlation between oro-nasal and molar-palate modules (ρ abs) and mean within-module correlation (Mean ρ). (1) Oro-nasal/rostrum, (2) molar-palate, (3) orbit, (4) zygomatic-pterygoid, (5) vault, (6) basicranium, (7) naso-palatine. All CR values were significant. Table S5. Static allometry-corrected modular architectures of two myrmecophagous mammals with three different sample sizes. Number of specimens (N), most likely modular architectures recovered with EMMLi (MLi), covariance ratio (CR), within-module absolute correlations (ρ), and correlation between oro-nasal and molar-palate modules assuming a therian six-module (V) architecture (ρ abs). (1) Oro-nasal/rostrum, (2) molar-palate, (3) orbit, (4) zygomatic-pterygoid, (5) vault, (6) basicranium, (7) naso-palatine. All CR values were significant. Table S6. Static allometry-corrected (when detected) effect size (ZCR) for the most likely architecture retrieved by EMMLi. For each most likely architecture, the p-values of the modular signal strength comparison are given. Most likely modular architectures (MLi), effect size calculated with the compare. [file 12862_2022_2030_MOESM3_ESM.pdf]

Table S1 – List of myrmecophagous species included in this study. Number of specimens (*N*).

Species name	Common name	*N*
T. tetradactyla	Collared anteater	74
T. mexicana	Northern tamandua	47
M. tridactyla	Giant anteater	35
C. didactylus	Pygmy anteater	60
P. maximus	Giant armadillo	14
O. afer	Aardvark	40
M. javanica	Sunda pangolins	28
M. pentadactyla	Chinese pangolin	27
S. temminckii	Ground angolin	15
S. gigantea	Giant pangolin	12
P. tricuspis	White-bellied pangolin	72
P. tetradactyla	Black-bellied pangolin	17
P. cristatus	Aardwolf	24

Table S2 – List of homologous anatomical landmarks used across the data set. Columns I to X show the affiliation of each landmark to the ten *a priori* architectures tested in this study (Table 2).

#	Landmark	I	II	III	IV	V	VI	VII	VIII	IX	X
**1**	Most antero-medial point of the maxilla	1	1	NA	1	1	1	1	1	1	1
**2**	Most antero-lateral contact of the maxilla	1	1	NA	1	1	1	1	1	1	1
**3**	Ventral margin of the infraorbital foramen	1	1	2	2	2	1	7	8	2	1
**4**	Ventral margin of the maxillary foramen	1	1	2	2	2	1	2	1	2	1
**5**	Intersection between maxilla and palatine sutures	1	1	2	2	2	2	7	8	2	2
**6**	Ventral margin of the sphenopalatine foramen	1	1	2	2	2	2	2	2	2	2
**7**	Ventral margin of the posterior caudal palatine foramen	1	1	2	2	2	2	2	2	2	2
**8**	Most dorsal point of the orbit	1	1	3	3	3	3	3	3	3	3
**9**	Intersection between palatine and pterygoid sutures	1	3	4	4	4	2	4	2	4	2
**10**	Optic foramen (most postero-ventral point)	2	1	3	3	3	3	3	3	3	3
**11**	Foramen rotundum (most postero-ventral point)	2	2	3	3	3	3	3	3	3	3
**12**	Anteriormost point of the zygomatic process of the squamosal	2	2	4	4	4	4	4	4	4	4
**13**	Most posterior point of the foramen ovale	2	3	4	4	4	4	4	4	4	4
**14**	Concavity at contact with the tympanic bulla	2	3	6	6	6	6	6	7	6	7
**15**	Anteriormost point of the jugular foramen	2	3	6	6	6	6	6	7	6	7
**16**	Posteriormost point of hypoglossus foramen	2	3	6	6	6	6	6	7	6	7
**17**	Most anterior point of the foramen magnum	2	3	6	6	6	6	6	7	6	7
**18**	Most postero-dorsal point of the foramen magnum	2	2	5	5	5	5	5	6	5	6
**19**	Most lateral point of the occipital condyle	2	3	6	6	6	6	6	7	6	7
**20**	Medial limit of the occipital condyle	2	3	6	6	6	6	6	7	6	7
**21**	Most antero-medial point of the maxilla	1	1	NA	1	1	1	1	1	1	1
**22**	Most antero-lateral contact of the maxilla	1	1	NA	1	1	1	1	1	1	1
**23**	Ventral margin of the infraorbital foramen	1	1	2	2	2	1	7	8	2	1
**24**	Ventral margin of the maxillary foramen	1	1	2	2	2	1	2	1	2	1
**25**	Ventral margin of the sphenopalatine foramen	1	1	2	2	2	2	2	2	2	2
**26**	Ventral margin of the posterior caudal palatine foramen	1	1	2	2	2	2	2	2	2	2
**27**	Most dorsal point of the orbit	1	1	3	3	3	3	3	3	3	3
**28**	Optic foramen (most postero-ventral point)	2	1	3	3	3	3	3	3	3	3
**29**	Foramen rotundum (most postero-ventral point)	2	2	3	3	3	3	3	3	3	3
**30**	Anteriormost point of the zygomatic process of the squamosal	2	2	4	4	4	4	4	4	4	4
**31**	Most posterior point of the foramen ovale	2	3	4	4	4	4	4	4	4	4
**32**	Concavity at contact with the tympanic bulla	2	3	6	6	6	6	6	7	6	7
**33**	Anteriormost point of the jugular foramen	2	3	6	6	6	6	6	7	6	7

**34**	Posteriormost point of hypoglossus foramen	2	3	6	6	6	6	6	7	6	7
**35**	Most lateral point of the occipital condyle	2	3	6	6	6	6	6	7	6	7
**36**	Medial limit of the occipital condyle	2	3	6	6	6	6	6	7	6	7
**37**	Anteriormost point of the nasal	1	1	NA	1	1	1	1	1	1	1
**38**	Anteriormost point of the suture between nasal and maxilla	1	1	NA	1	1	1	1	1	1	1
**39**	Intersection mx/nasal/frontal	1	1	3	3	3	3	7	8	7	8
**40**	Intersection between palatine/lacrimal/frontal	1	1	3	3	3	3	3	3	3	3
**41**	Zygomatic process of the maxilla	1	1	2	2	4	4	2	4	2	4
**42**	Intersection between inter-nasal and inter-frontal sutures	1	1	3	3	3	3	7	8	7	8
**43**	Intersection between inter-parietal fronto-parietal suture	2	2	5	5	5	5	5	6	5	6
**44**	Intersection between inter-parietal and supraoccipital	2	2	5	5	5	5	5	6	5	6
**45**	Intersection between squamosal/supraoccipital/parietal	2	2	5	5	5	5	5	6	5	6
**46**	Posteriormost point of the skull roof on the supraoccipital	2	2	5	5	5	5	5	6	5	6
**47**	Concavity located above the condyle	2	2	5	5	5	5	5	6	5	6
**48**	Anteriormost point of the nasal	1	1	NA	1	1	1	1	1	1	1
**49**	Anteriormost point of the suture between nasal and maxilla	1	1	NA	1	1	1	1	1	1	1
**50**	Intersection mx/nasal/frontal	1	1	3	3	3	3	7	8	7	8
**51**	Intersection between palatine/lacrimal/frontal	1	1	3	3	3	3	3	3	3	3
**52**	Zygomatic process of the maxilla	1	1	2	2	4	4	2	4	2	4
**53**	Intersection between squamosal/supraoccipital/parietal	2	2	5	5	5	5	5	6	5	6
**54**	Concavity located above the condyle	2	2	5	5	5	5	5	6	5	6

Table S3 –Number of clusters resulting from the EDMA for each myrmecophagous species and minimum Jaccard coefficient per number of modules (*k*) calculated from a 10,000 bootstrap. Number of specimens (N), value of Gap statistics (Gap) for the optimal number of clusters (Cluster), and final number of clusters after removing biologically meaningless cluster (Final).

	N	Gap	Clusters	Final (J>0.70)	Final (>0.60)	Jaccard
T. tetradactyla	74	0.49	8	5	8	0.73
T. mexicana	47	0.53	9	5	9	0.75
M. tridactyla	35	0.73	8	4	7	0.90
C. didactylus	60	0.42	7	4	7	0.82
P. maximus	16	0.48	9	3	6	0.91
O. afer	40	0.11	1*	6	6	0.76
M. javanica	28	0.35	9	4	6	0.73
P. pentadactyla	27	0.35	4	4	4	0.73
S. temminckii	15	0.41	8	4	8	0.75
S. gigantea	12	0.06	1*	3	4	0.90
P. tricuspis	72	0.36	8	4	7	0.74
P. tetradactyla	17	0.30	7	7	7	0.74
P. cristatus	24	0.46	7	5	5	0.78

Table S4 – Modular architectures of 13 myrmecophagous species. Number of specimens (*N*), most likely modular architectures recovered with EMMLi (*MLi*), covariance ratio (*CR*), within-module absolute correlations (*ρ*), correlation between oro-nasal and molar-palate modules (*ρ abs*) and mean within-module correlation (Mean *ρ*). (1) Oro-nasal/rostrum, (2) molar-palate, (3) orbit, (4) zygomatic-pterygoid, (5) vault, (6) basicranium, (7) naso-palatine. All CR values were significant.

	N	*MLi*	*CR*	*ρ 1*	*ρ 7*	*ρ 2*	*ρ 3*	*ρ 4*	*ρ 5*	*ρ 6*	*ρ abs 1-2*	Mean *ρ*
T. tetradactyla	74	VII (7)	**0.60**	0.76	0.18	0.51	0.34	0.25	0.20	0.40	-0.49	0.38
T. mexicana	43	VII (7)	**0.68**	0.73	0.19	0.46	0.30	0.31	0.21	0.27	-0.42	0.35
M. tridactyla	35	IX (7)	**0.60**	0.74	0.62	0.43	0.39	0.28	0.29	0.50	-0.52	0.46
C. didactylus	60	VII (7)	**0.71**	0.68	0.16	0.43	0.21	0.42	0.18	0.21	-0.25	0.33
P. maximus	14	V (6)	**0.74**	0.64	-	0.32	0.23	0.19	0.29	0.24	-0.21	0.27
O. afer	40	VII (7)	**0.72**	0.43	0.15	0.26	0.36	0.18	0.24	0.34	-0.15	0.28
M. javanica	28	VII (7)	**0.67**	0.56	0.28	0.36	0.37	0.26	0.23	0.26	-0.31	0.33
M. pentadactyla	27	VII (7)	**0.71**	0.49	0.26	0.25	0.28	0.14	0.17	0.20	-0.12	0.26
S. temminckii	15	VII (7)	**0.76**	0.64	0.23	0.33	0.32	0.20	0.22	0.21	-0.27	0.31
S. gigantea	12	VIII (7)	**0.82**	0.58	0.28	0.74	0.29	0.37	0.31	0.26	-0.38	0.40
P. tricuspis	72	VII (7)	**0.62**	0.46	0.23	0.40	0.22	0.31	0.16	0.19	-0.20	0.28
P. tetradactyla	17	VII (7)	**0.79**	0.59	0.24	0.35	0.29	0.35	0.26	0.24	-0.28	0.33
P. cristatus	24	IX (7)	**0.65**	0.46	0.49	0.28	0.29	0.24	0.15	0.27	-0.17	0.28

Table S5 – Static allometry-corrected modular architectures of two myrmecophagous mammals with three different sample sizes. Number of specimens (*N*), most likely modular architectures recovered with EMMLi (*MLi*), covariance ratio (*CR*), within-module absolute correlations (*ρ*), and correlation between oro-nasal and molar-palate modules assuming a therian six-module (V) architecture (*ρ abs*). (1) Oro-nasal/rostrum, (2) molar-palate, (3) orbit, (4) zygomatic-pterygoid, (5) vault, (6) basicranium, (7) naso-palatine. All CR values were significant.

	N	*MLi*	*ρ 1*	*ρ 7*	*ρ 2*	*ρ 3*	*ρ 4*	*ρ 5*	*ρ 6*
T. tetradactyla	74	VII (7)	0.75	0.18	0.49	0.32	0.27	0.19	0.40
T. tetradactyla	30	VII (7)	0.75	0.16	0.53	0.29	0.27	0.24	0.42
T. tetradactyla	15	VII (7)	0.78	0.27	0.52	0.33	0.20	0.35	0.58
P. tricuspis	72	VII (7)	0.41	0.22	0.40	0.22	0.31	0.17	0.20
P. tricuspis	30	VII (7)	0.46	0.24	0.39	0.23	0.34	0.22	0.21
P. tricuspis	15	VII (7)	0.52	0.28	0.47	0.29	0.44	0.16	0.27

Table S6 – Static allometry-corrected (when detected) effect size (Z_{CR}) for the most likely architecture retrieved by EMMLi. For each most likely architecture, the *p-values* of the modular signal strength comparison are given. Most likely modular architectures (*MLi*), effect size calculated with the *compare.CR* function (Z_{CR}, Adams & Collyer, 2019), *p-value* of the comparison with non-modular hypothesis (0), *p-value* for the comparison with the remaining modular hypothesis (I-X). The lowest Z_{CR} values are in bold**.** Significant and marginally significant *p-values* are in red and orange, respectively.

	MLi	Z_{CR}	0	I	II	III	IV	V
T. tet.	VII (7)	**-9.65**	$3,38x10^{-24}$	$2,84x10^{-3}$	$2,20x10^{-2}$	$5,74x10^{-1}$	$5,74x10^{-1}$	$1,76x10^{-1}$
T. mex.	VII (7)	**-8.76**	$6,67x10^{-20}$	$3,37x10^{-3}$	$1,88x10^{-3}$	$3,87x10^{-1}$	$3,87x10^{-1}$	$1,72x10^{-1}$
M. tri.	VII (7)	**-5.92**	$1,56x10^{-9}$	0,56	0,36	$8,70x10^{-1}$	$8,70x10^{-1}$	$5,87x10^{-1}$
C. did.	VII (7)	**-9.04**	$1,29x10^{-20}$	$3,52x10^{-4}$	$4,75x10^{-3}$	$7,02x10^{-1}$	$7,02x10^{-1}$	$6,61x10^{-1}$
P. max.	V (6)	-5.22	$1,15x10^{-7}$	0,97	0,45	$9,40x10^{-1}$	$9,40x10^{-1}$	1,00
O. afe.	VIII (7)	**-8.47**	$1,30x10^{-18}$	$2,90x10^{-5}$	$5,40x10^{-3}$	$4,85x10^{-1}$	$4,85x10^{-1}$	$4,82x10^{-1}$
M. jav.	VII (7)	**-8.37**	$3,48x10^{-18}$	$2,60x10^{-3}$	$5,61x10^{-2}$	$6,31x10^{-1}$	$6,31x10^{-1}$	$8,14x10^{-1}$
M. pen.	VII (7)	-6.51	$2,70x10^{-11}$	0,19	$3,63x10^{-1}$	$8,10x10^{-1}$	$8,10x10^{-1}$	$7,88x10^{-1}$
S. tem.	V (6)	-5.85	$2,43x10^{-9}$	$2,83x10^{-3}$	0,02	$7,90x10^{-1}$	$7,90x10^{-1}$	1,00
S. gig.	VIII (7)	**-7.07**	$3,75x10^{-13}$	$3.52x10^{-4}$	$3.22x10^{-2}$	$1.76x10^{-1}$	$1.76x10^{-1}$	$1.69x10^{-1}$
P. tri.	VII (7)	**-8.04**	$7,66x10^{-17}$	$5,82x10^{-2}$	$8,16x10^{-2}$	$7,79x10^{-1}$	$7,79x10^{-1}$	$8,55x10^{-1}$
P. tet.	VII (7)	-7.28	$6,66x10^{-14}$	$3,13x10^{-2}$	$2,45x10^{-2}$	$5,10x10^{-1}$	$5,10x10^{-1}$	$6,73x10^{-1}$
P. cri.	VII (7)	**-7.40**	$2,54x10^{-14}$	$4,59x10^{-3}$	$5,70x10^{-3}$	$6,04x10^{-1}$	$6,04x10^{-1}$	$7,24x10^{-1}$

	MLi	Z_{CR}	VI	VII	VIII
T. tet.	VII (7)	**-9.65**	$3,68x10^{-1}$	1,00	$7,33x10^{-1}$
T. mex.	VII (7)	**-8.76**	$3,72x10^{-1}$	1,00	$8,48x10^{-1}$
M. tri.	VII (7)	**-5.92**	$7,77x10^{-1}$	1,00	$6,35x10^{-1}$
C. did.	VII (7)	**-9.04**	$1,83x10^{-1}$	1,00	$5,21x10^{-1}$
P. max.	V (6)	-5.22	$9,16x10^{-1}$	$6,26x10^{-1}$	$7,82x10^{-1}$
O. afe.	VIII (7)	**-8.47**	$7,74x10^{-1}$	$6,48x10^{-1}$	1,00
M. jav.	VII (7)	**-8.37**	$8,57x10^{-1}$	1,00	$8,42x10^{-1}$
P. pen.	VII (7)	-6.51	$7,40x10^{-1}$	1,00	$4,76x10^{-1}$
S. tem.	V (6)	-5.85	$6,87x10^{-1}$	$3,46x10^{-1}$	$3,43x10^{-1}$
S. gig.	VIII (7)	**-7.07**	$5.40x10^{-1}$	$6.67x10^{-1}$	1.00
P. tri.	VII (7)	**-8.04**	$7,01x10^{-1}$	1,00	$9,44x10^{-1}$
P. tet.	VII (7)	-7.28	$5,69x10^{-1}$	1,00	$8,90x10^{-1}$
P. cri.	-	**-7.40**	$3,62x10^{-1}$	1,00	$9,10x10^{-1}$

Table S7 – Between-modules correlations of *C. didactylus* and *M. tridactyla*. Between-modules absolute correlations (ρ) for the most-likely architecture in *C. didactylus* (upper triangle; values in black) and *M. tridactyla* (lower triangle; values in blue). (1) Oro-nasal/rostrum, (2) molar-palate, (3) orbit, (4) zygomatic-pterygoid, (5) vault, (6) basicranium, (7) naso-palatine.

	1	*7*	*2*	*3*	*4*	*5*	*6*
1		0.17	0.28	0.24	0.17	0.19	0.14
7	0.37		0.15	0.13	0.17	0.11	0.10
2	0.49	0.10		0.18	0.27	0.12	0.15
3	0.31	0.20	0.22		0.21	0.17	0.17
4	0.10	0.08	0.10	0.15		0.13	0.22
5	0.40	0.15	0.30	0.22	0.14		0.17
6	0.52	0.26	0.34	0.32	0.16	0.28	

Table S8 – Between-modules correlations of *T. mexicana* and *T. tetradactyla*. Between-modules absolute correlations (ρ) for the most-likely architecture in *T. mexicana* (upper triangle; values in black) and *T. tetradactyla* (lower triangle; values in blue). (1) Oro-nasal/rostrum, (2) molar-palate, (3) orbit, (4) zygomatic-pterygoid, (5) vault, (6) basicranium, (7) naso-palatine.

	1	*7*	*2*	*3*	*4*	*5*	*6*
1		0.31	0.44	0.39	0.17	0.3	0.28
7	0.3		0.15	0.12	0.1	0.13	0.13
2	0.48	0.12		0.19	0.11	0.16	0.21
3	0.4	0.1	0.24		0.19	0.2	0.21
4	0.16	0.07	0.08	0.17		0.25	0.14
5	0.24	0.1	0.18	0.18	0.14		0.16
6	0.36	0.12	0.26	0.28	0.15	0.18	

Table S9 – Between-modules correlations of *S. gigantea* and *S. temminckii*. Between-modules absolute correlations (ρ) for the most-likely architecture in *S. gigantea* (upper triangle; values in black) and *S. temminckii***.** (lower triangle; values in blue). (1) Oro-nasal/rostrum, (2) molar-palate, (3) orbit, (4) zygomatic-pterygoid, (5) vault, (6) basicranium, (7) naso-palatine/naso-frontal.

	1	*7*	*2*	*3*	*4*	*5*	*6*
1		0.33	0.38	0.32	0.29	0.18	0.32
7	-		0.37	0.20	0.14	0.14	0.28
2	0.22	-		0.15	0.17	0.20	0.25
3	0.26	-	0.15		0.24	0.23	0.25
4	0.29	-	0.17	0.22		0.26	0.24
5	0.25	-	0.13	0.15	0.19		0.21
6	0.22	-	0.14	0.18	0.17	0.23	

Table S10 – Between-modules correlations of *M. pentadactyla* and *M. javanica*. Between-modules absolute correlations (ρ) for the most-likely architecture in *M. pentadactyla* (upper triangle; values in black) and *M. javanica* (lower triangle; values in blue). (1) Oro-nasal/rostrum, (2) molar-palate, (3) orbit, (4) zygomatic-pterygoid, (5) vault, (6) basicranium, (7) naso-palatine.

	1	*7*	*2*	*3*	*4*	*5*	*6*
1		0.16	0.2	0.31	0.19	0.16	0.16
7	0.18		0.12	0.15	0.11	0.12	0.14
2	0.29	0.11		0.12	0.13	0.1	0.14
3	0.27	0.08	0.14		0.18	0.17	0.13
4	0.21	0.13	0.14	0.2		0.16	0.11
5	0.18	0.12	0.14	0.19	0.2		0.15
6	0.24	0.09	0.18	0.17	0.17	0.18	

Table S11 – Between-modules correlations of *P. tricuspis* and *P. tetradactyla*. Between-modules absolute correlations (ρ) for the most-likely architecture in *P. tricuspis* (upper triangle; values in black) and *P. tetradactyla* (lower triangle; values in blue). (1) Oro-nasal/rostrum, (2) molar-palate, (3) orbit, (4) zygomatic-pterygoid, (5) vault, (6) basicranium, (7) naso-palatine.

	1	*7*	*2*	*3*	*4*	*5*	*6*
1		0.18	0.19	0.18	0.17	0.14	0.13
7	0.21		0.12	0.1	0.11	0.06	0.11
2	0.25	0.16		0.17	0.17	0.11	0.12
3	0.27	0.12	0.16		0.23	0.14	0.12
4	0.39	0.16	0.13	0.2		0.14	0.1
5	0.25	0.12	0.2	0.22	0.21		0.16
6	0.22	0.17	0.11	0.16	0.19	0.18	

Table S12 – Between-modules correlations of *O. afer* and *P. maximus*. Between-modules absolute correlations (ρ) for the most-likely architecture in *O. afer* (upper triangle; values in black) and *P. maximus* (lower triangle; values in blue). (1) Oro-nasal/rostrum, (2) molar-palate, (3) orbit, (4) zygomatic-pterygoid, (5) vault, (6) basicranium, (7) naso-palatine.

	1	*7*	*2*	*3*	*4*	*5*	*6*
1		0.16	0.18	0.2	0.16	0.15	0.23
7	-		0.14	0.12	0.15	0.1	0.1
2	0.24	-		0.11	0.14	0.13	0.14
3	0.26	-	0.18		0.16	0.18	0.22
4	0.16	-	0.16	0.16		0.13	0.16
5	0.30	-	0.24	0.17	0.13		0.12
6	0.11	-	0.17	0.15	0.17	0.18	

Table S13 – Between-modules correlations of *P. cristata*. Between-modules absolute correlations (ρ) for the most-likely architecture, (1) Oro-nasal/rostrum, (2) molar-palate, (3) orbit, (4) zygomatic-pterygoid, (5) vault, (6) basicranium, (7) naso-palatine.

	1	*7*	*2*	*3*	*4*	*5*	*6*
1		0.13	0.17	0.3	0.24	0.13	0.2
7	-		0.13	0.06	0.12	0.16	0.1
2	-	-		0.17	0.19	0.11	0.15
3	-	-	-		0.24	0.12	0.21
4	-	-	-	-		0.14	0.25
5	-	-	-	-	-		0.14
6	-	-	-	-	-	-	
